# Pharmacophore modelling based virtual screening and molecular dynamics identified the novel inhibitors and drug targets against *Waddlia chondrophila*

**DOI:** 10.1038/s41598-024-63555-1

**Published:** 2024-06-12

**Authors:** Sidra Aslam, Hossam M. Aljawdah, Mutee Murshed, Geidy E. Serrano

**Affiliations:** 1 Banner Sun Health Research Institute, Sun City, AZ USA; 2 Department of Zoology, College of Science, King Saud University, P.O. Box 2455, 11451 Riyadh, Saudi Arabia

**Keywords:** *Waddlia chondrophila*, Drug targets, Phytochemicals, Molecular dynamic simulation, Molecular docking, Computational biology and bioinformatics, Immunology

## Abstract

*Waddlia chondrophila* is a possible cause of fetal death in humans. This Chlamydia-related bacterium is an emergent pathogen that causes human miscarriages and ruminant abortions, which results in financial losses*.* Despite the years of efforts, the underlying mechanism behind the pathogenesis of *W. chondrophila* is little known which hindered the development of novel treatment options. In the framework of current study, computational approaches were used to identify novel inhibitors (phytocompounds) and drug targets against *W. chondrophila*. At first, RNA polymerase sigma factor SigA and 3-deoxy-d-manno-octulosonic acid transferase were identified through subtractive proteomics pipeline. Afterwards, extensive docking and simulation analyses were conducted to optimize potentially novel phytocompounds by assessing their binding affinity to target proteins. A 100ns molecular dynamics simulation well complimented the compound's binding affinity and indicated strong stability of predicted compounds at the docked site. The calculation of binding free energies with MMGBSA corroborated the significant binding affinity between phytocompounds and target protein binding sites. The proposed phytocompounds may be a viable treatment option for patients infected with *W. chondrophila*; however, further research is required to ensure their safety.

## Introduction

Outbreaks of Re-Emerging Infectious Diseases (REID) and Emerging Infectious Diseases (EID) have increased since the turn of the millennium which are communicable threats to public health^1^. Another Chlamydia-like abortigenic agent, *Waddlia chondrophila,* has also been linked to poor pregnancy outcomes in humans^2,3^. *W. chondrophila* is regarded as a potential cause of embryonic death in humans because it causes poor reproduction in females who have had infrequent recurrent abortions^4^. Additionally, lung samples from people who had pneumonia also revealed the involvement of *W. chondrophila*. Despite years of research, nothing is known about the biology or pathogenicity of *W. chondrophila.*

Recent studies provide evidence that *W. chondrophila* is responsive to azithromycin, doxycycline, and fluoroquinolones in cell cultures and resistant to lactams, but there is currently no approved therapy against *W. chondrophila*^5^. Regrettably, bacteria often develop resistance to antibiotics, making them difficult or even impossible to treat^6^. To tackle this issue, computational approaches are widely used to understand infectious diseases by establishing the latest research trends that seize advantages from the advancements made in both structural and molecular biology. Recently, subtractive proteomics has proven to be an efficient approach for the identification of species-specific vaccine candidates as well as prospective therapeutic targets against a variety of harmful bacteria^7,8^. Subtractive proteomics is thought to be the panacea for identifying novel therapeutic targets in pathogens^9^. The confirmation of target proteins acquired via subtractive proteomics underscores the pivotal role these identified targets play in the disease pathology. Furthermore, molecular dynamics (MD) simulation surpasses docking by integrating a spectrum of physiological parameters, crucial for accurately predicting the authentic mode of molecular interactions. This advanced computational approach provides a nuanced understanding of the dynamic behavior and structural changes within biological systems, offering invaluable insights for drug design and therapeutic interventions.

Understanding the mechanisms of drug resistance in Waddlia chondrophila is essential for devising effective treatment strategies. Current studies reveal that resistance mechanisms include genetic mutations altering antibiotic targets, efflux pump activity expelling antibiotics, and phenotypic changes like entering a dormant state. Furthermore, bioinformatics analysis of protein expression differences between drug-sensitive and drug-resistant strains could unveil potential therapeutic targets. Targeting proteins upregulated in resistant strains or unique to sensitive strains may offer avenues for novel treatments, enhancing antibiotic efficacy and combating resistance. Overall, elucidating resistance mechanisms and identifying potential targets through proteomic analysis are pivotal in developing strategies to tackle W. chondrophila infections.

Phytochemicals are compounds that are primarily produced by plants and have biological function. Plants are a good source of many active compounds in the pharmaceutical sector. They have pharmacological properties that can be used to treat bacterial and fungal infections, as well as chronic-degenerative disorders like cancer and diabetes. Many studies in recent years have shown that phytochemicals exert antibacterial activity via various mechanisms of action, such as suppression of virulence factors and bacterial membrane damage, including inhibition of toxin and enzyme activity, and bacterial biofilm formation^10^.

To effectively manage the pathogenesis of infectious diseases in the wake of all these changes, computational approaches are being developed. The study uses a multi-target strategy to conduct virtual screening utilizing molecular docking and dynamic simulation to find potential phytochemicals that can reduce the pathogenicity of diseases. Regarding this, a subtractive proteomics process was utilized to identify the target proteins, which were then further explored with a library of phytochemicals using molecular docking and dynamics simulations. Furthermore, we anticipate that this work may open the way for the development of promising and effective drugs candidates against *W. chronophilia.*

## Methodology

### Sequence retrieval, filtering and identification of paralogous sequences

The complete proteome of the *Waddlia chondrophila* was collected from UniProt in FASTA format^11^. UniProt is a central hub of protein sequence and functional information. It offers a user-friendly interface for interconnecting and storing valuable protein-related information from large and disparate sources^12^. One copy of each protein is sufficient to carry out an optimal function since redundant proteins are not necessary. The entire proteome was then subjected to CD-Hit suit for removal of non-redundant sequences^13^. The cutoff criteria for screening of non-redundant sequences was set to 80%. Only those proteins which met the precise criteria of screening were used for further analysis.

### Non-homologous proteins identification

Non-homology with host proteins is required for the anticipated non-redundant proteins with unique metabolic pathways. It is important to remember that predicted proteins may trigger immunological reactions if they are comparable to the host.^14^. To discover the non-homologous protein sequences of *W. chondrophila*, essential proteins were submitted to BlastP against the human proteome with standard parameter settings^15^. BLASTp was performed against non-redundant proteins, and proteins with query cover > 30% and identity > 70% were considered as non-homologous proteins.

### Protein subcellular localization prediction

Identifying the protein subcellular localization is crucial to grasp the structure and function of the cell as a whole as well as the function of specific proteins. For a large variety of proteins, bioinformatics predictors of localization can swiftly offer this information^16^. Regarding this, CELLO^17^ and pSORTb server^18^ were used for the identification of cytoplasmic proteins from the pool of predicted non-homologous proteins. The proper functioning of certain proteins is determined on their subcellular localization. Both PSORTb and CELLO server used support vector machine algorithm to expand the prediction of bacterial protein subcellular localization.

### Comparative analysis of metabolic pathways

Screened cytoplasmic proteins were processed to compare metabolic pathways. This research is carried out in order to identify therapeutic targets based on pathway enzymes that are both common and essential to bacteria^19^. The metabolic pathways of *Waddlia chondrophila* were discovered by comparing *Waddlia chondrophila* with *Homo sapiens pathways* in the Kyoto Encyclopedia of Genes and Genomes (KEGG) database^20^*.* Those metabolic pathways that are unique to *Waddlia chondrophila* and not found in humans were chosen. The purpose of this study is to identify potential therapeutic targets based on the similarities and differences between the metabolic pathways that bacteria and humans share. Therefore, only those proteins with unique metabolic pathways were taken into consideration for the rest of the investigation.

### Drugability analysis

The druggability of predicted cytoplasmic proteins was also investigated. The nature of the target, or the target's "druggability," essentially limits the effectiveness of many drug design endeavours. Here, we define "druggability" as the capacity of a target to be altered by strong, little molecules that act like drugs (which are often suitable for oral delivery). Early target assessment can therefore be a potent portfolio management tool by allocating resources to "druggable" targets that are more likely to produce clinical candidates^21^. BLAST heuristic search with an e-value of 10^−5^ is utilized by the user-friendly cheminformatics program known as DrugBank. This search is used to merge qualitative drug data with in knowledge of therapeutic strategies^22^.

### Prediction and evaluation of 3D-structure of targeted proteins

After successful completion of the protein sequence analysis, and evaluation, the targeted proteins were sent to the structure prediction tool for further consideration. AlphaFold was applied to forecast the 3D structure of targeted proteins^23^. AlphaFold is a neural network-based approach for precise protein structure prediction. The quality of enhanced pharmaceutical targets was evaluated using four distinct technologies (ERRAT, ProCheck, Verify 3D, and ProsA-web)^24–27^.

### Library preparation of phytochemicals

To investigate the potential repressive effect on the targeted proteins, 1,000 identified phytochemicals were gathered using an in silico technique from numerous databases, including PubChem, MPD3, and Zinc^28–30^. The plant-based compounds were chosen for their medicinal potential in accordance with the results of the literature review^31^. Phytochemicals belonging to classes sterols and alkaloids are considered as medicinally key active compounds of plants. The phytochemicals alkaloids and sterols were the most frequently selected. Chem Draw was used to create a visual representation and predict the stereochemistry of compounds^32^. To perform, virtual screening using molecular docking, current study relied on Molecular operating environment (MOE) software (written in a scientific vector language) for energy minimization by picking the MMFF94x force-field to create a fully prepared library of compounds^33^. At 310 K and pH 7, the target protein structures were refined to add partial charges using the Protonate3D tool. The target proteins' active sites were found by the Site Finder tool in the MOE software. In order to make use of the compound in the MOE ligand database were optimized with the energy of Protonate3D was reduced prior to accessing the database^34^.

### Molecular docking analysis

Molecular docking techniques are commonly used in current drug design to analyze ligand conformations within macromolecular target binding sites. This method calculates the free energy of binding between the ligand and receptor by evaluating critical components of the intermolecular recognition process^35^. The MOE (Molecular operating environment) tool was utilized to screen a library of 1000 phytochemicals against the targeted proteins using a molecular docking approach. In order to acquire accurate findings, all docking experiments were carried out with the default parameters^36^. To rescore simulated poses, MOE's London dG scoring system was employed. Protonation and energy minimization was also done. After docking, the best and most favorable phytochemicals were selected on the basis of binding affinity^37^. LigX is a tool for visualizing the best-docked complexes and examining the 2D structure of ligand-receptor interactions^38^.

### Druglikeness and ADMET profiling

Drug-likeness evaluation is a vital stage in the drug development process that should not be disregarded^39^. Chemical and physical parameters such as molecular weight, the logP coefficient (miLogP), hydrogen bond acceptors, hydrogen bond donors were all considered^40^. In order to determine the druggability of the top-docked ligands, we used the Molinspiration online tool (retrieved on May 8, 2021))^41^. It is generally recognized that ADMET profiling of candidate compounds are among the most important major shortcomings of drug development; therefore, it is imperative that ADMET studies be performed as early in the drug development process as possible^42^. Evaluation models to find the adverse drug-drug interaction have been established as an alternative tool to aid medicinal chemists in the creation and optimization of lead compounds. The ADMET lab 2.0 server was used to evaluate the ADMET properties^43^.

### Molecular dynamic simulation

Molecular Dynamic simulations of the top hits for target proteins were carried out in order to have a better understanding of their dynamics at the time scale of 100 ns^44^. The simulation studies were carried out with the assistance of the desmond software^45^. MD simulations were used to evaluate the stability of complexes after a number of stages, including preprocessing, optimization, and minimization. The minimization procedure was carried out using the OPLS_2005 force field^46^. A periodic box measuring 10 Å was used to solvate the compounds, which contained water molecules from TIP3P^47^. In order to replicate physiological conditions, counter ions and 0.15 M NaCl salt were added as needed to neutralize the systems. The NPT ensemble was calibrated to operate at 1 atm of pressure and 300 K of temperature. The systems went through a relaxation phase before the simulation started. Trajectories were recorded and saved at 40 ps intervals during the simulation, enabling subsequent analysis of the obtained results.

### Binding energy analysis

The binding free energy of drugs toward receptors was assessed to assure the stability of the compounds' binding. This was accomplished by using the molecular mechanics with generalized born and surface area solvation (MM/GBSA) method. This technique is a well-known, reliable, and potent analytical approach. The binding free energy of specific MD snapshots was calculated using the Amber tool 20 MMGBSA script (py)^48–50^.

## Results

**Figure 1:**
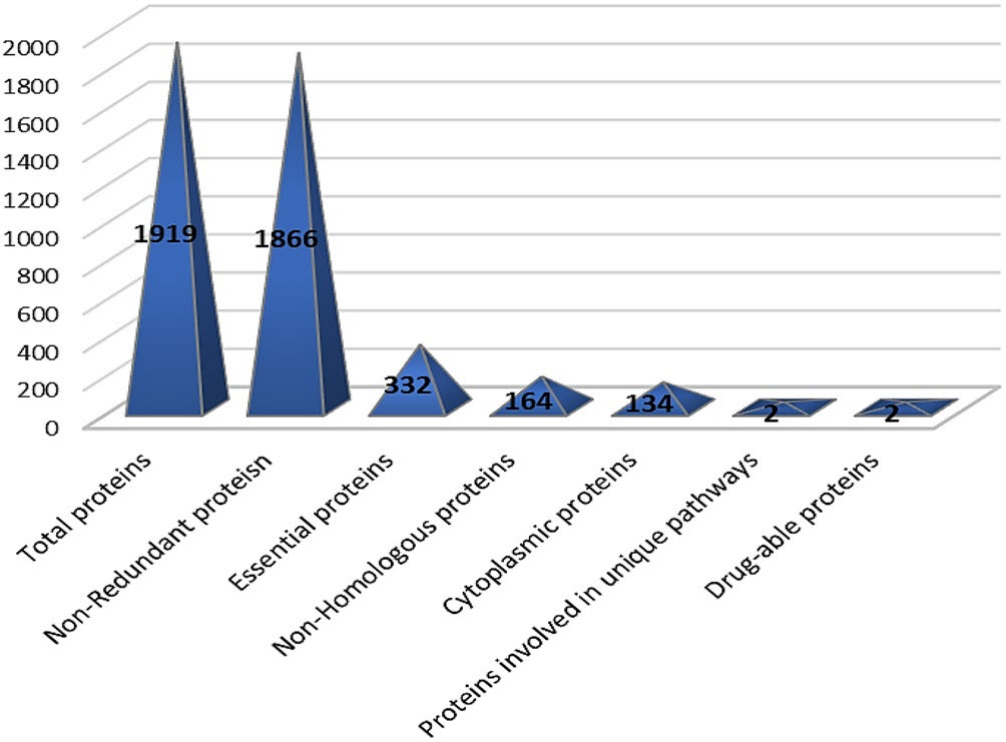
Graphical synopsis representing the outcome of subtractive proteomics approach.

This research focuses on the discovery of new potential targets for the creation of innovative and potent drugs to treat *W. chondrophila* infections (Fig. 1). The subtractive proteomics analysis performed in current work was basically relied on various computational databases and tools.

### Screening vital proteins

The whole proteome of W. chondrophila (Strain: ATCC VR-1470) were consisting of total 1919 proteins (Supplementary file S1). These 1919 proteins were then analyzed to identify the putative inhibitors for infection of W. chondrophila. After eliminating paralog sequences from these proteins using CD-HIT at the aforementioned threshold, a total of 1866 proteins were retrieved. sBecause duplicated proteins are not required for an organism survival therefore they must be removed from subsequent analysis, otherwise these duplicated proteins lead to erroneous results. Further, these 1866 proteins were screened for identification of essential proteins and total 332 proteins were identified as essential proteins, which were then used for further analysis.

### Prediction of non-homologous proteins

To be an effective pharmacological target, a protein must be essential for the survival of disease-causing organism in the host body but non-homologous to human proteins and this criterion is required for preventing drug cross-binding to human proteins, as well as the avoiding the side effects of drugs. Then, using BlastP, only those essential proteins that are not similar to human proteins were predicted. When 332 essential proteins were exposed to BlastP, then total 164 were identified as non-homologous proteins because these proteins satisfied the exact criteria of identity ≤ 30%.

### Subcellular localization prediction

It is critical to understand the function of a particular protein while designing a therapeutic drug. Simple and reliable approaches for figuring out a protein's function are provided by subcellular localization prediction. Furthermore, studies have shown that localization is a crucial factor for creating any therapeutic choices due to the fact that proteins can localize in a variety of locations. As a result, 30 of the targeted proteins were predicted to be membrane proteins and were not included in the rest of the investigation. In contrast, 134 proteins were determined to be cytoplasmic proteins that could be used as therapeutic targets (Supplementary file S2).

### Comparative analysis of metabolic pathways

A comparison of the metabolic pathways of *W. chondrophila* and *H. sapiens* was done in order to monitor the pharmacological targets engaged in the pathogen-specific pathways. Twenty-five non-homologous proteins were found to be involved in numerous metabolic pathways when the metabolic pathways of Homo sapiens and *W. chondrophila* were compared (Table 1). It has been demonstrated that ribosomal protein S3 causes caspase-dependent apoptosis in humans, whereas ribosomal protein S4 is involved in the development of the gonads^51^. On the other hand, ribosomal protein L22 is a novel p53 activator that is widely mutated (mainly deletion mutation) in a variety of human malignancies, while ribosomal protein L32 encodes a ribosomal protein that is a component of the 60S subunit. These points provide some important clues that ribosomal proteins (L32, L22, S4, S3) has key role in human, thus not suitable to be used as therapeutic target^52^. From these 25 proteins, only 2 proteins (RNA polymerase sigma factor SigA and 3-deoxy-d-manno-octulosonic acid Transferase) were named as bacteria-specific as neither protein has any similarities to human pathways.

**Table 1:** Predicted protein pathways in the body's metabolic processes.

Protein name	Common in human	Unique
Thioredoxin reductase	Selenocompound metabolism	
Pyruvate kinase	Pyruvate metabolism Glycolysis/gluconeogenesis Biosynthesis of amino acids Carbon metabolism Metabolic pathways	Biosynthesis of secondary metabolites Microbial metabolism in diverse environments
Riboflavin synthase, alpha subunit	Metabolic pathways Riboflavin metabolism Biosynthesis of cofactors	Secondary metabolites biosynthesis
30S ribosomal protein S8	Ribosome	
d-Alanine-d-alanine ligase	Metabolic pathways d-Amino acid metabolism	Peptidoglycan biosynthesis Vancomycin resistance
Chorismate synthase	Metabolic pathways Biosynthesis of amino acids Phenylalanine, tyrosine and tryptophan biosynthesis	Biosynthesis of secondary metabolites
Glycerol-3-phosphate dehydrogenase	Glycerophospholipid metabolism	Biosynthesis of secondary metabolites
Malonyl CoA-acyl carrier protein transacylase	Fatty acid biosynthesis Fatty acid metabolism Metabolic pathways	Biosynthesis of secondary metabolites
2-amiX-4-hydroxy-6-hydroxymethyldihydropteridine diphosphokinase	Folate biosynthesis Biosynthesis of cofactors Metabolic pathways	
Dihydropteroate synthase	Folate biosynthesis Metabolic pathways biosynthesis of cofactors	
cell division protein	Metabolic pathways	Peptidoglycan biosynthesis Beta-Lactam resistance
DNA-directed RNA polymerase subunit alpha	RNA polymerase	
Phenylalanine–tRNA ligase beta subunit	Aminoacyl-tRNA biosynthesis	
50S ribosomal protein L32	Ribosome	
DNA-directed RNA polymerase subunit beta	RNA polymerase	
50S ribosomal protein L22	Ribosome	
30S ribosomal protein S4	Ribosome	
Riboflavin biosynthesis protein	Metabolic pathways Riboflavin metabolism Biosynthesis of cofactors	Biosynthesis of secondary metabolites
30S ribosomal protein S3	Ribosome	
EXyl-[acyl-carrier-protein] reeducates	Fatty acid metabolism Fatty acid biosynthesis Metabolic pathways Biosynthesis of cofactors Biotin metabolism	Biosynthesis of secondary metabolites
RNA polymerase sigma factor SigA		Flagellar assembly
3-oxoacyl-[acyl-carrier-protein] synthase 3	Fatty acid biosynthesis Fatty acid metabolism Metabolic pathways	
3-deoxy-d-manno-octulosonic acid transferase		Lipopolysaccride biosynthesis

### Druggability analysis

Another essential factor for identifying effective targets is the analysis of druggability. Druggability is probability that compound may affect the working of specific protein. The DrugBank database was utilized to predict the similarity of proteins to their respective drug targets in this case. It has been noteworthy that our predicted proteins named were found to be significantly similar to drug entries of the database. Hence strengthened our findings that these proteins might act as novel and putative drug targets against *W. chondrophila* infections.

### Structure prediction and evaluation

**Figure 2:**
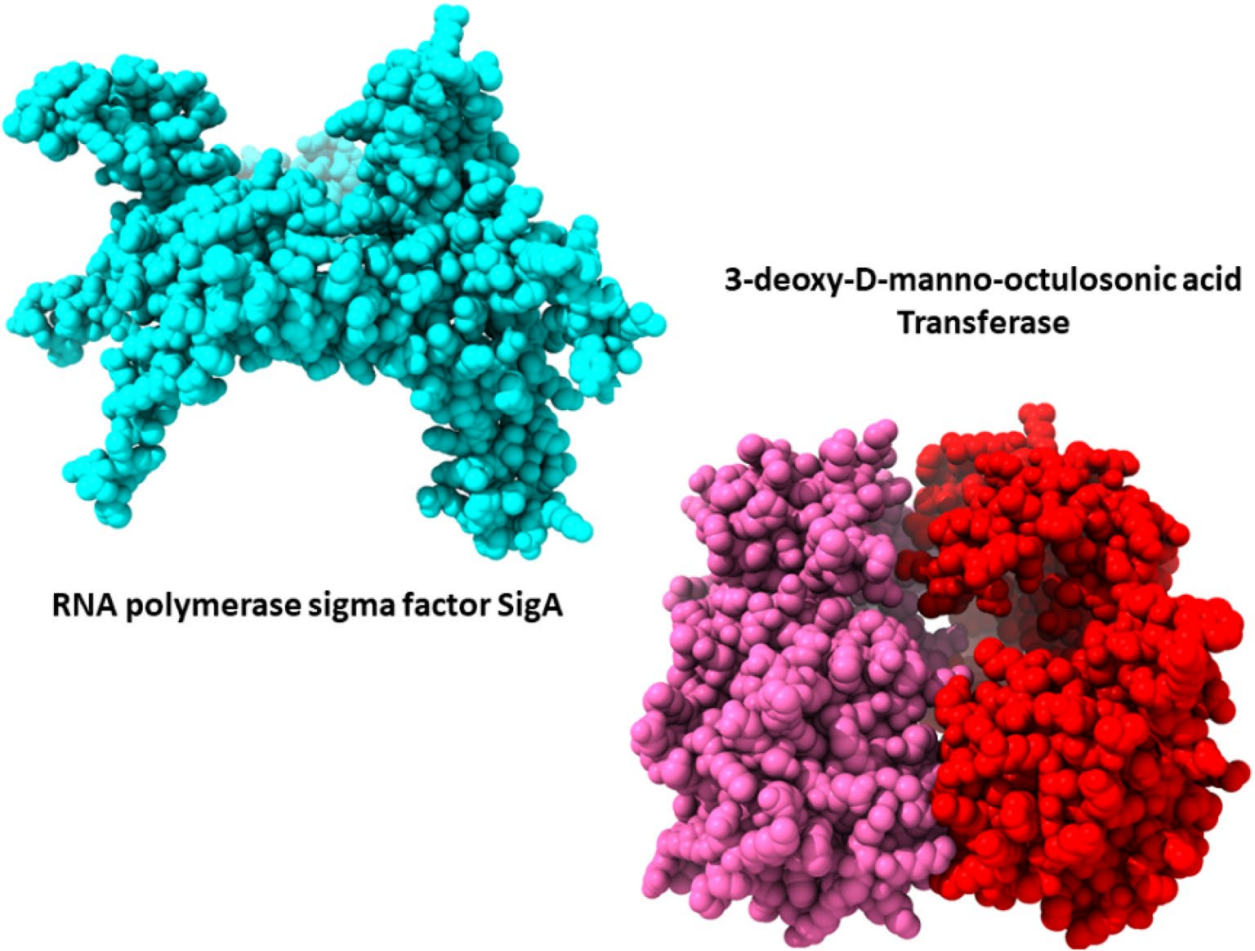
3D Structure visualization of the unique proteins.

The AlphaFold predicted the best anticipated 3D structure of target proteins. 3D structures of the predicted proteins were shown in the Fig. 2.

Using revised pharmacological targets, the protein structures quality was further evaluated. Several methods were used to validate the 3D models. PROCHECK server was used to crosscheck modelled structure's quality. Finally, an examination of the proteins' 3D models revealed that 85–90% of the residues were discovered in the favored and authorized regions, meaning that all projected models are of excellent quality. The Z-scores predicted by the ProSA web tool are a parametric quantity that measures the overall quality of the model. All the predicted structures quality score was mentioned in the Table 2.

**Table 2:** Several computer approaches are used to depict the level and improvement of the projected structures.

Receptors	RNA polymerase sigma factor SigA	3-deoxy-d-manno-octulosonic acid transferase
C-score	− 4.67	− 5.87
Estimated TM-score	0.25 ± 0.08	0.28 ± 0.09
Ramachandran plot statistics (%)		
Core	90.9%	85.9%
Allowed	6.3%	11.9%
General	1.9%	1.0%
Disallowed	0.9%	1.3%
Verify 3D		
Compatibility score	76.65%	81.68%
ERRAT		
Quality factor	85.7461	78.6861
ProSA		
z-score	− 7.09	− 9.28

### Docking analysis of targeted proteins

**Figure 3:**
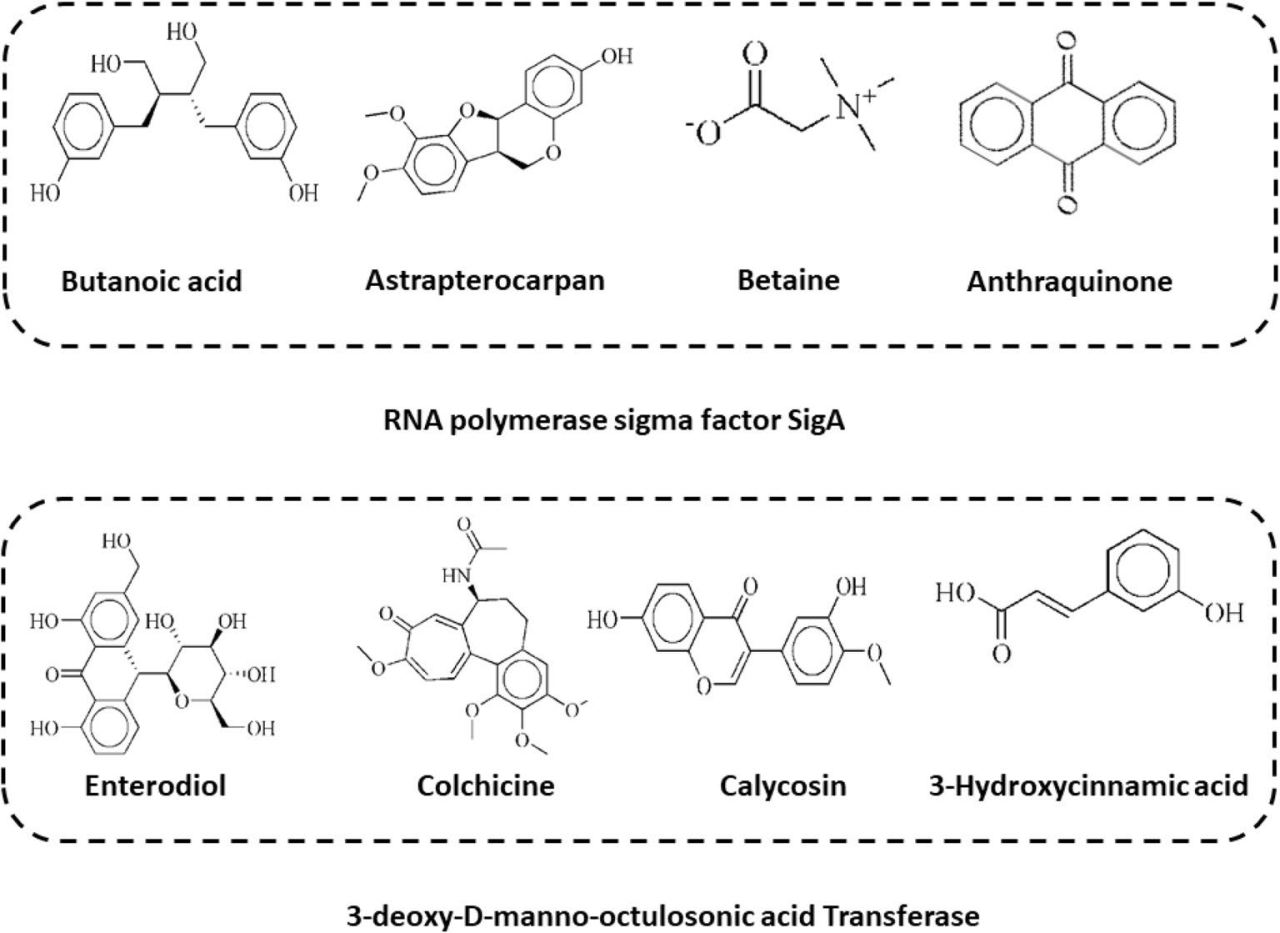
Top four drug candidates 2D structure visualization obtained after virtual screening.

The results of docking the receptor protein structures with the phytochemicals library using MOE software were presented in this section. Ten different conformations were determined for each molecule. Binding affinity score, RMD values, and bonding interactions with both proteins' active sites (RNA polymerase sigma factor SigA and 3-deoxy-d-manno-octulosonic acid Transferase) were used to sort the conformations of these compounds. The top four molecules from each receptor protein were chosen for further examination based on the lowest docking score value, as shown in Fig. 3.

As shown in Table 3, these chosen substances interact strongly with the protein binding pockets and have the lowest binding energies when compared to the scoring functions of each docked ligand. In our docking process, we meticulously identified the binding site through comprehensive protein–ligand contact analysis. Specifically, we pinpointed key residues such as Asn242, Trp283, Arg286, Arg290, and Arg301, which actively engaged in hydrogen bonding with the ligand. Notably, our analysis revealed that Arg290 displayed a remarkable binding affinity, evidenced by its presence in 57% of the total frames. This meticulous identification underscores the precision of our docking methodology, providing crucial insights into the ligand–protein interactions.

**Table 3:** The most promising medicinal candidates Affinity for binding as well as interacting residues.

Compounds I’D	Compounds name	Binding affinity (kcal/mol)	RMSD	Interacting residues
RNA polymerase sigma factor SigA				
1489	Butanoic acid	− 9.29	0.98	Arg 300, Tyr 299, His 69, Phe 297, Glu 65, Glu 298, Glu 72, Arg 168, Glu 107, His 171
14,077,830	Astrapterocarpan	− 8.84	1.87	Arg 300, His 69,Tyr 299,Arg 168, Glu 65, Glu 72, His 171,Glu 107
247	Betaine	− 8.80	1.04	Glu 65, Arg 300, Glu 72
6780	Anthraquinone	− 7.76	1.59	His 171, Arg 168, Glu 72, Gln 68, Glu 107
3-deoxy-d-manno-octulosonic acid transferase				
115,089	Enterodiol	− 10.53	1.26	Glu B27, Leu B46, Thr A71,Val B25, Thr B24, Trp A74
6167	Colchicine	− 10.06	1.89	Lys A108, Phe A109, Pro A107, Phe B45, Glu B27
5,280,448	Calycosin	− 9.90	0.81	Phe B45, IIe B67, Gln B65, IIe B19, Ser B23
637,541	3-Hydroxycinnamic acid	− 8.75	0.99	Glu B27, Asp A72, Tyr A73, Thr A71, Val B25, Phe B45

**Figure 4:**
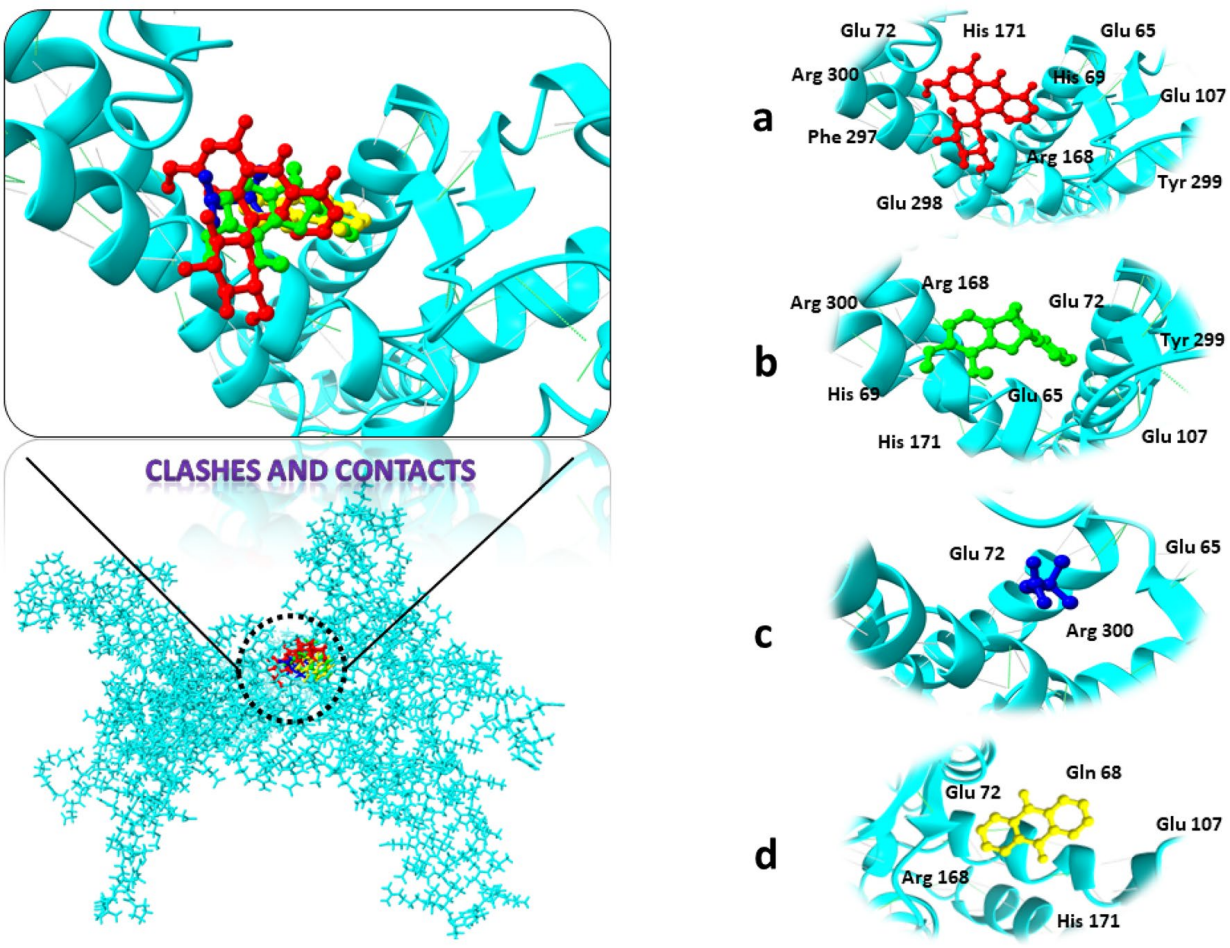
3D visualization of receptor and ligand indicating interacting residues. (**a**) Butanoiic acid in complex with RNA polymerase sigma factor SigA. (**b**) Astrapterocarpan/ RNA polymerase sigma factor SigA. (**c**) Betaine/ RNA polymerase sigma factor SigA. (**d**) Anthraquinone/ RNA polymerase sigma factor SigA.

Target protein 3-deoxy-d-manno-octulosonic acid Transferase showed strong binding affinity with compounds Enterodiol, Colchicine, Calycosin, and 3-Hydroxycinnamic acid. Enterodiol inhibitor in complex with target protein 3-deoxy-d-manno-octulosonic acid Transferase ranked as top having the least binding affinity of − 10.53 kcal/mol with strong hydrogen bond and other interacting residues. All the compounds with receptor proteins were showed in the Fig. 4.

**Figure 5:**
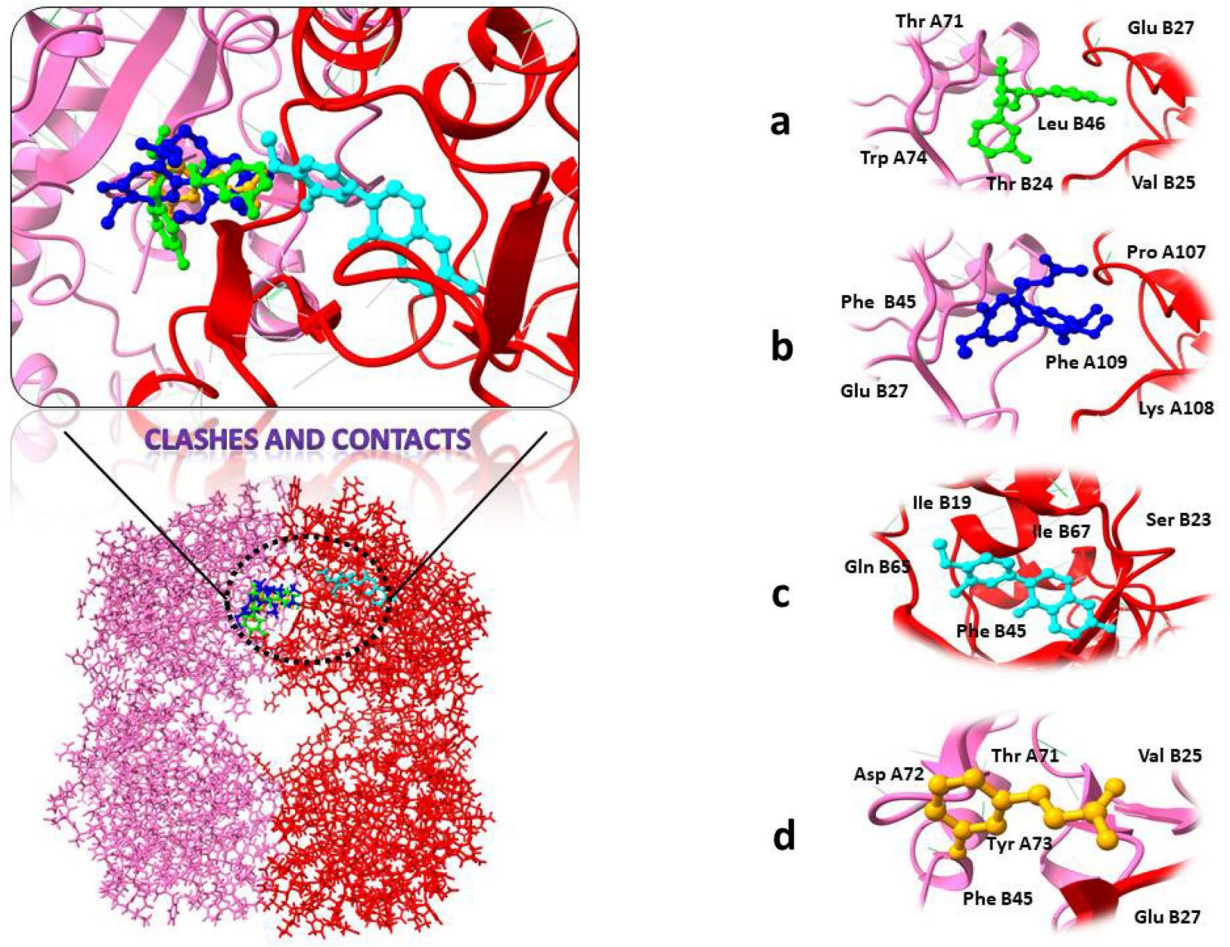
3D visualization of targeted protein with attached ligand indicating interacting residues. (**a**) Enterodiol in complex with 3-deoxy-d-manno-octulosonic acid transferase. (**b**) Colchicine/3-deoxy-d-manno-octulosonic acid transferase. (**c**) Calycosin/3-deoxy-d-manno-octulosonic acid transferase. (**d**) 3-Hydroxycinnamic acid/3-deoxy-d-manno-octulosonic acid Transferase.

Secondly, RNA polymerase sigma factor SigA in complex with the ligands Butanoic acid, Astrapterocarpan, Betaine, and Anthraquinone showed the binding affinity scores between − 9.29 and − 7.76 kcal/mol. Although Butanoic acid in complex with protein RNA polymerase sigma factor SigA ranked as top compound due to least binding affinity and showed good interactions along with the receptor protein. 3D visualization and interacting residues of the protein structure along with attached ligands were shown in Fig. 5.

### Druglikeness

A drug scanning was performed utilizing the molinspration service to assess the top Compounds' drug-likeness properties. Lipinski's five-rule has become the standard. This rule highlights important drug characteristics in the human body, such as pharmacokinetics, interactions, and metabolism, as well as their excretion. Selected compounds didn't break any of Lipinski's five requirements and possessed drug-like properties like molecular weight (Table 4).

**Table 4:** Compounds with a high probability of becoming medicines, according to the Lipinski rule.

Compounds I’D	Molecular weight	Hydrogen bond donner (HBD)	Hydrogen bond acceptor (HBA)	mi-LogP
RNA polymerase sigma factor SigA				
1489	418.40	5	7	0.18
14,077,830	300.31	5	1	2.55
247	117.15	3	0	-5.41
6780	208.21	0	0	3.4
3-deoxy-D-manno-octulosonic acid transferase				
115,089	302.37	4	4	2.39
6167	399.44	1	7	1.10
5,280,448	284.27	5	2	2.38
637,541	164.16	3	2	1.41

### ADMET profiling

ADME and AdmetSAR were used to evaluate a number of pharmacokinetic variables. The ADME and toxic effects of the top potential candidate agents can be evaluated using pharmacokinetic characteristics. Table 5 shows the ADMET properties of derived phytochemicals for both targets. Because of cytotoxicity and poor pharmacokinetic properties, many medicines do not exploit this mechanism in their development. Early drug discovery focuses on high-performance and quick ADMET profiling research to identify active lead components. The ADMET Lab was also used to confirm the drug likeness of potential compounds by testing their ADMET profiles (Table 5).

**Table 5:** Potential compounds’ ADMET profiling of top drug candidates (X: no, ✓: yes).

Parameters	RNA polymerase sigma factor SigA				3-deoxy-d-manno-octulosonic acid transferase			
	1489	14,077,830	247	6780	115,089	6167	5,280,448	637,541
Target proteins								
Absorption/distribution								
Blood brain barrier	X	X	X	X	X	X	X	X
Aqueous solubility (Log S)	− 3.289	− 3.810	0.086	− 5.143	− 1.590	− 2.680	− 3.628	− 2.386
Caco-2 permeability	− 6.059	− 4.718	− 5.795	− 4.631	− 4.807	− 4.712	− 4.686	− 4.812
Pgp-inhibitor	X	X	X	X	X	X	X	X
Pgp-substrate	X	X	X	X	X	X	✓	X
Metabolism								
CYP1A2 inhibitor	X	X	X	✓	✓	X	✓	X
CYP1A2 substrate	X	X	X	X	X	✓	✓	X
CYP2C19 inhibitor	X	X	X	✓	X	X	X	X
CYP2C19 substrate	X	✓	X	X	X	✓	X	X
CYP2C9 inhibitor	X	X	✓	X	X	X	X	X
CYP2C9 substrate	X	X	X	X	X	X	X	✓
CYP3A4 inhibitor	X	X	X	X	X	X	X	X
CYP3A4 substrate	X	✓	X	X	X	X	X	X
Toxicity								
Rat oral acute toxicity	X	X	X	X	X	X	X	X
AMES toxicity	Non-toxic	Non-toxic	Non-toxic	Non-toxic	Non-toxic	Non-toxic	Non-toxic	Non-toxic
Carcinogenicity	Non-Carcino genic	Non-Carcinogenic	Non-Carcinogenic	Non-Carcinogenic	Non-Carcinogenic	Non-Carcinogenic	Non-Carcinogenic	Non-Carcinogenic

### Molecular dynamic simulation

Molecular dynamic modeling was used to determine the structural stability of the docked complexes combinations with targeted receptors (RNA polymerase sigma factor SigA and 3-deoxy-d-manno-octulosonic acid). The MD simulation was run for 100 ns for the docked complexes (Butanoic acid/ RNA polymerase sigma factor SigA and Enterodiol/3-deoxy-d-manno-octulosonic acid Transferase). Furthermore, in our molecular dynamics (MD) simulations, we went beyond mere docking and actively compared the behavior of our candidate ligands with known active ligands or original ligands. This step was pivotal in establishing a robust evaluation criterion for identifying active candidates. Through this comparative analysis within the MD process, we gained invaluable insights into the dynamic behavior and stability of our ligand candidates, laying a solid foundation for evaluating their potential activity. Our approach not only enhances the reliability of our findings but also contributes to advancing the field by setting a benchmark for evaluating ligand activity through simulation methodologies.

#### Enterodiol/3-deoxy-d-manno-octulosonic acid transferase

**Figure 6:**
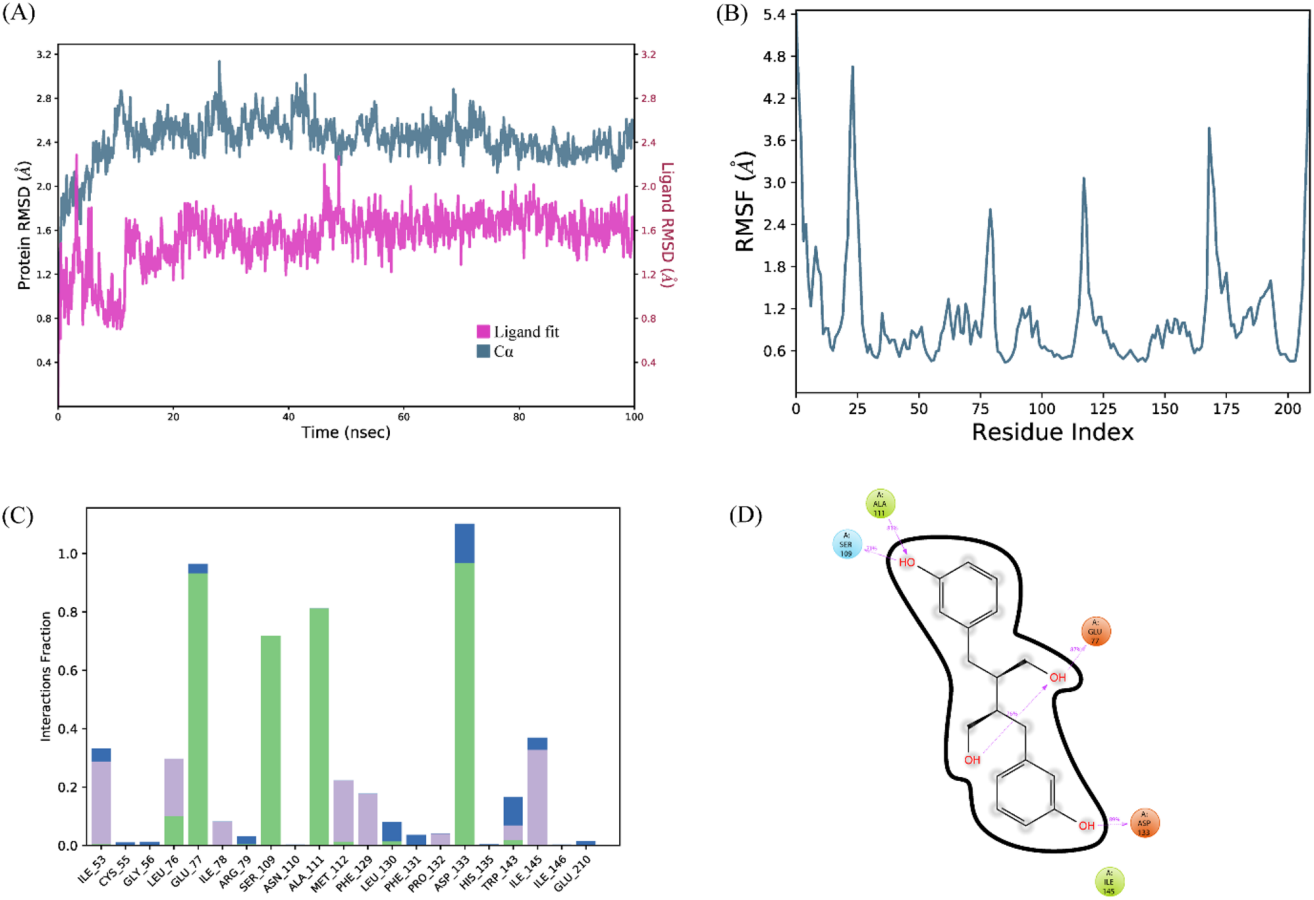
The MD trajectory analysis of the 3DAT complex. (**A**) RMSD of C-alpha atoms of proteins and ligand atoms fit on protein. (**B**) RMSF analysis. (**C**) Protein–ligand contacts calculated during simulation. (**D**) Tendency of the interacting residues with the ligand during simulation.

**Figure 7:**
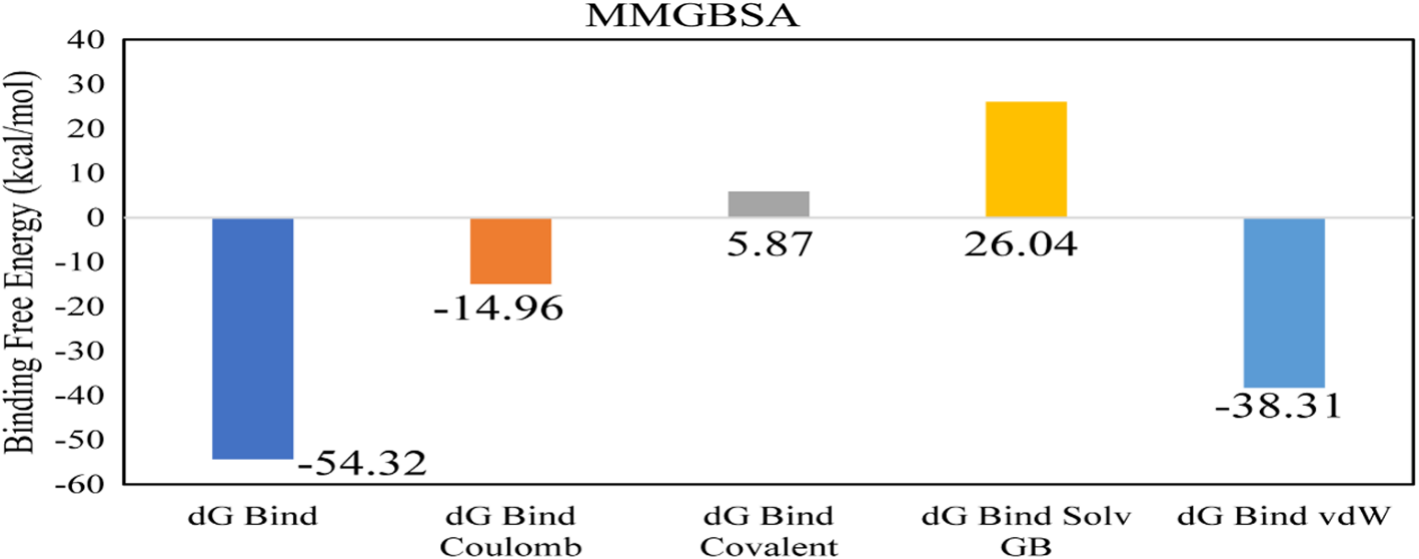
The contribution of the energy components in total binding free energy in Enterodiol/3-deoxy-d-manno-octulosonic acid transferase complex.

The stability of the complex was analyzed by conducting a 100 ns simulation. The RMSD of C-alpha atoms of protein and ligand fit on protein was calculated to find the stability of the complex. It was observed that the C-alpha atoms of protein maintained the RMSD values in the range of ~ 2.4–2.8 Å throughout the simulation after being equilibrated at 15 ns, while the RMSD of ligand was in the range of ~ 1.6 Å throughout the simulation (Fig. 6A). The structural dynamics of the protein residues were analyzed by calculating the RMSF values which show the flexibility of protein residues in response to the binding of this ligand during simulation. The higher RMSF values show flexibility and lower values indicate the rigidity of the residues. From the RMSF values, it was observed that most of the residues remained rigid during the simulation except for the loop regions whose values were ~ 4.8 Å. The loop regions contain the residues ranging from 20 to 30, 75 to 80, 120 to 125, and 165 to 180 (Fig. 6B). During MD simulation analysis, the most significant interactions between the protein and ligands were found to be ionic bonds, hydrogen bonds, and hydrophobic interactions. These interactions are critical in stabilizing the protein–ligand complex and regulating its functional characteristics. The specific residues involved in hydrogen bonding were Leu76, Glu77, Ser109, Ala111, and Asp133 (Fig. 6C). Among these interacting residues, Asp133 exhibited the highest tendency for binding, with interactions observed during 89% of the total frames (Fig. 6D). Furthermore, the binding free energy of the complex was calculated by prime-MMGBSA module. The binding free energy was the total of Coulomb, Van der Waals, Solvation, and Covalent energies. The van der Waals energy contribution was − 38.31, solvation was 26.04, covalent energy was 5.87, coulombic energy was − 14.96, and total binding free energy of the complex was − 54.32 kcal/mol as shown in Fig. 7.

#### Butanoic acid/RNA polymerase sigma factor SigA

**Figure 8:**
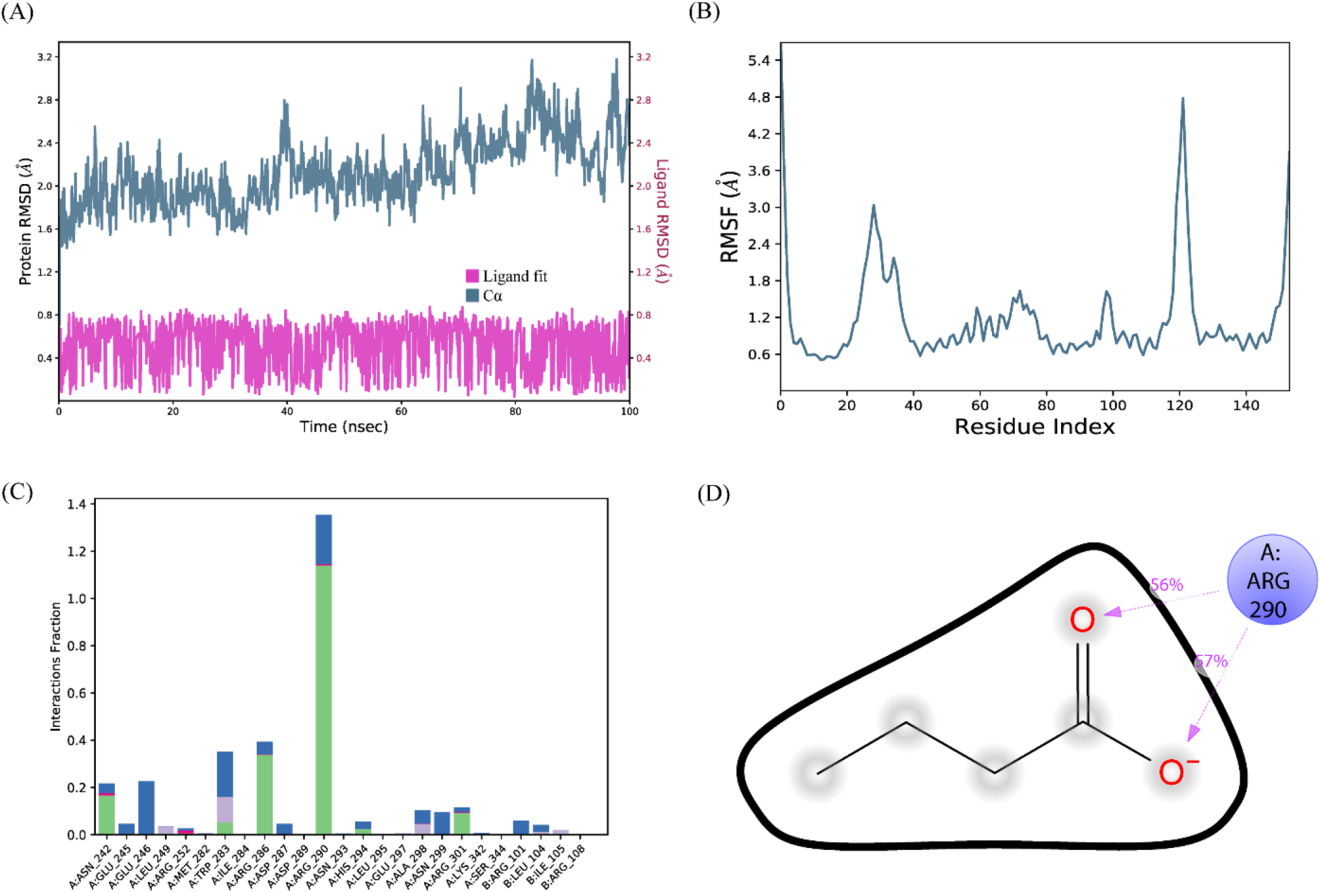
The MD trajectory analysis of the SigA complex. (**A**) RMSD of C-alpha atoms of proteins and ligand atoms fit on protein. (**B**) RMSF analysis. (**C**) Protein–ligand contacts calculated during simulation. (**D**) Tendency of the interacting residues with the ligand during simulation.

**Figure 9:**
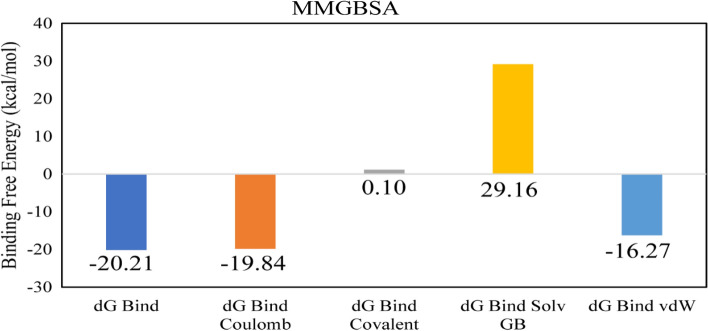
The contribution of the energy components in total binding free energy in Butanoic acid/RNA polymerase sigma factor SigA complex.

The RMSD of C-alpha atoms of protein maintained the RMSD values in the range of ~ 1.6–2.8 Å throughout the simulation, while the RMSD of ligand remained in the range of 0.8 Å, which indicates the stability of the complex (Fig. 8A). The RMSF analysis showed that that most of the residues remained rigid during the simulation except for two loop regions whose values reached ~ 3 and 4.8 Å (Fig. 8B). In protein–ligand contact analysis, the specific residues involved in hydrogen bonding were Asn242, Trp283, Arg286, Arg290, and Arg301 (Fig. 8C). Among these interacting residues, Arg290 exhibited the highest tendency for binding, with interactions observed during 57% of the total frames (Fig. 8D). Furthermore, the binding free energy of the complex was shown in Fig. 9. The van der Waals energy contribution was − 16.27, solvation was 29.16, covalent energy was 0.10, columbic energy was − 19.84, and total binding free energy of the complex was − 20.21 kcal/mol.

## Discussion

*W. chondrophila* is a new pathogen that causes sickness in both humans and cattle^4^. According to a research done by Baud et al.^53^ sixty nine women who experienced occasional miscarriages, two hundred women with history of abortions, and sixty nine command females having uncomplicated maternities due to *W. chondrophila*. In overall other investigations have confirmed the link between *W. chondrophila* and unfavorable pregnancy outcomes^54^. The real epidemiology is still unknown owing via lack of accessible purchase of legally uniform techniques for detecting *W. chondrophila* within victims^55,56^. Exposure to zooxtic organisms, as well as consumption of poisoned food or milk is also a plausible mechanisms of transmission^57,58^. The discovery of *W. chondrophila* in well groundwater sources prompted the development of a water network^59,60^. Due to the general mechanism of transmission, mass-order abortions are possibilities, with agricultural and dairy workers being the most vulnerable^61–63^. To combat such diseases, prior clinical preparations are required, which is the overall purpose of the current investigation.

For the study, the proteome was first purchased from UniProt. The effectiveness of plasmid proteins for the purpose of this research was questioned because they can be eliminated by the organism while under selective pressure, thus they were previously disregarded. Most large protein datasets contain largely identical patterns (paralogous), and removing them reduces the state space, saving time and processing resources. Only repeated sequences that were > 60% similar in structure and function to other proteins were removed from the proteome using CD-HIT. The essential *W. Chondrophila* proteins were identified using several databases. Protein sequences that have been empirically proven to be crucial to the organism are included in the database of vital genes. Even though they are essential to bacteria, not all of them can be used as therapeutic targets because some of them can be connected to the metabolic system of the host. In order to compare those important proteins to homo sapiens, BlastP was used.

Only 164 of the 332 essential proteins were remained, according to the results study. Then, only 134 proteins were found when cytoplasmic proteins were fetched from the cello server. Generic similarity to a previously recognized pharmacological target would be highly helpful in the medication search process, assisting in logical cure discovery and repurposing/repositioning. So, using the BLAST method, proteins were compared to the DrugBank database. The Drug Bank is a large online database that contains a lot of information about pharmaceuticals and remedy targets^64^. The database's high resemblance to FDA-approved medication targets enables despite a considerably shielded and further targeted therapy regimen. Just twenty-eight proteins met the criteria for the cut-off. The drug searching process would be greatly aided by sequence homology to a previously identified pharmacological targets, assisting in rational medicine discovery and repurposing/repositioning. As a result, proteins were compared to the DrugBank database using the BLAST algorithm. The DrugBank is a large freely available database that contains a lot of information about pharmaceuticals and controlled substance goal. The database's upraised resemblance to FDA-approved medication targets enables despite a considerably shielded and spare targeted therapy regimen. At most 28 proteins met the standard for the cut-off.

Targeting certain proteins could result in toxicity and cross-reactions with the host KEGG pathway. To get over this problem, Annotation from the Annotation Server or KAAS be employed. KEGG is a database usefulness since deciphering the peak-level functionality of organic structure using molecular-level data such as datasets from high-throughput sequence analysis or other methodologies. The KEGG database contains information on the metabolic pathways of *W. chondrophilia* and its host Homo sapiens, as well as their corresponding kids^65^. To discover pathogen-specific pathways, the routes were manually obtained and verified from the information. Following that, RNA polymerase sigma factor SigA and 3-deoxy-d-manno-octulosonic acid transferase were utilized to identify proteins that are only found in one route.

Initiation factors known as sigma factors help RNA polymerase connect to particular initiation sites and are then released. The main sigma factor during exponential growth is this one. 3-deoxy-d-manno-octulosonic acid transferase is involved in biosynthesis of lipopolysaccharide (LPS) The current study shown that we can suppress the production of target proteins by finding their active regions. Some substances have significant interactions with these three *W. chondrophila* related proteins. The "Lipinski Rule of Five" was wised to estimate the molecular characteristics and drug like properties of the selected complexes. When compared to typical drugs, selected molecules had low docking score values. ADMET analysis is a difficult technique in drug development^66^. This was accomplished using the ADMETsar database, which revealed that certain drugs have favorable pharmacokinetic features^67^.

After that structural stability of the docked complexes combinations with targeted receptors (RNA polymerase sigma factor SigA and 3-deoxy-d-manno-octulosonic acid) were further accessed by molecular dynamic simulation. The fundamental elements of protein–ligand interactions can be studied well with the use of MD simulations. They expand our understanding of the molecular mechanisms behind protein–ligand interaction, enhance docking predictions, rank ligand candidates, and coordinate subsequent optimization efforts^68–70^. According to MMGBSA analyses and MD simulations, these compounds were stable as effective inhibitors within the binding pocket of protein.

Same technique was used by researchers to identify potential targets against *Streptococcus pneumoniae* Strain D39^71^, *Fusobacterium nucleatum*^72^*, Streptococcus pyogenes*^48^*, and shigella sonnei*^73^*.* Hence, the novel drug targets discovered in this work should be very helpful in the drug therapeutic industry for finding inhibitors and devising new drug formulations to combat *W. chondrophila* infections, albeit more experimental research are required to confirm these drug targets.

## Conclusion

In conclusion, the urgent global challenge of antimicrobial resistance has spurred intensive research efforts to identify new therapeutic avenues. Through the exploration of metabolic pathways specific to pathogens and leveraging successful targeting strategies in other bacterial species, this study has unveiled two promising targets within W. chondrophila. This discovery marks a notable advancement in our understanding and potential treatment of infections caused by this pathogen. Moving forward, it is imperative for future research endeavors to delve deeper into these identified targets, assessing their impact on the longevity and virulence of W. chondrophila. By doing so, we can pave the way for the development of effective antibacterial agents, offering hope in the ongoing battle against antimicrobial.

### Supplementary Information

Supplementary Information 1.

Supplementary Information 2.

## Data Availability

All data generated or analyzed during this study are included in this published article and its supplementary information files. Complete detail of the whole proteome of *W. chondrophila* (Supplementary file S1). Total Cytoplasmic proteins (Supplementary file S2).
