# Supplementary material for: Pharmacophore modelling based virtual screening and molecular dynamics identified the novel inhibitors and drug targets against Waddlia chondrophila
Source: Sci Rep. 2024 Jun 12;14:13472. doi: 10.1038/s41598-024-63555-1 (PMC11169463; doi:10.1038/s41598-024-63555-1)
Supplement: Supplementary file 1 — Supplementary Information 1. [file 41598_2024_63555_MOESM1_ESM.docx]

**Complete detail of *Waddlia chondrophila* proteome**

>tr|D6YTC9|D6YTC9_WADCW UDP-GlcNAc pyrophosphorylase OS=Waddlia chondrophila (strain ATCC VR-1470 / WSU 86-1044) OX=716544 GN=glmU1 PE=4 SV=1

MEPFSPLELFDLSSYPHKALLENCTYAWEALLFLKKYLTQQNLGNLQGTISSNAYLVNKN

QIFIGKGTVIEPGAYIEGPCWIGENCTIRHGAYIRGNVITGHGCILGHDSEFKHSILLNG

SQAAHFAYVGDSILGNHINLGAGTICANLKLDKQPVEVKFESHRIHTGLRKLGAILGDNA

QTGCHAVLNPGTVFGKAALCYPCLNVSGYLSPGTKMKMEK

>tr|D6YS65|D6YS65_WADCW DNA topoisomerase (ATP-hydrolyzing) OS=Waddlia chondrophila (strain ATCC VR-1470 / WSU 86-1044) OX=716544 GN=parE PE=4 SV=1

MAKKYDETTIQTLDALEHIRKRTGMYIGRLGDGSHYDDGIYILIKEVIDNCIDEFIMGNG

NQILITKDQDLGKITVRDFGRGIPLGKVIECVSQINTGAKYNDDVFQFSVGLNGVGTKAV

NALSSRFLVRSHREGKFVEAEFSKGRLQKDKKGKTQEKDGTYVEFIPDSEIFKSFKFDDE

YIVKRLWNYVYLNTGLTINYNGERIRSDHGLLDLLNQEVSEEGLYEPLHYKSKQLEFAFL

HTQSYGESYFSFVNGQYTSDGGTHLSAFREGLLKGVNEYAKKNFQGVDVREGVFGTVAIR

IQDPVFESQTKNKLGNTEIRAPLVQEVKEAVVNLLFKHPSTAKKIIERIAFNEKVRKELS

AVKKEAKEKQKKISFKIPKLRDSKYHFGDGSKYAEKTMIFLTEGDSASASIVSSRDPLTQ

AVFSLRGKPLNVFGMKMDQLYKNEEMFNVMSALNIEEDISNLRYEKVVLATDADVDGMHI

RNLLITLFLTYFEGLVLNGHLYILETPLFKVRNKEKTIYCYSEKEKEKAVKELKKGVEIT

RFKGLGEISPQEFKQFIGPDIRLIPVAVNSFSDIKPTLQFYMGKNTPERKKFIMNNLVNE

DQELL

>tr|D6YVV2|D6YVV2_WADCW Uncharacterized protein OS=Waddlia chondrophila (strain ATCC VR-1470 / WSU 86-1044) OX=716544 GN=wcw_0901 PE=4 SV=1

MKIKCLVALFLVIGIFEFSYSESVAESLDFSIEDRLDRVENFLNRQVPGLEVSYHAVIET

LINEGWDVYIRGGAMRDLLAPIPNEPKDIDFDYSGTTEELKAILDRHQWQYTQLPGRSTI

VIGEHRGIHMEAVPLSVAFGHGESSLEFTINNIFYHCNQKSLVPGTEVGVVDLSYQRLNI

LTPNWKTWLYRKGRHPFERIFRSWNMIGKGFVYSVDFANFLNREAVKKMHRFPDIFREEM

LHYLGAHYESFDDIYYGSIAIMGYDWAQEQVLSLKDEAEKKNIAGQDLRDQFTFFSRP

>tr|D6YVN8|D6YVN8_WADCW Acetylornithine aminotransferase OS=Waddlia chondrophila (strain ATCC VR-1470 / WSU 86-1044) OX=716544 GN=argD PE=3 SV=1

MNSYEFINDPRVKEAKKLLLEAVREQQEKITGIRLPDPALEKEYEEIIERFSQNRGGPLW

FPFIGAGIGKGPFVQLLDGSIKYDFISGIGVHHFGHSHPKIIESSIDAAISDTVMQGHLQ

QNIDQVELIELLVKESGLDHCFLSTTGAMANENGLKIALQKNFPANRVLAFERAFCGRTW

AMAQITEKNDVREGLPVNLAVDYIPFYDHNHPEKSITLAVAALKKYLKRYPKSHAAMICE

LIQGEGGFYPGSREFFTEIMTILKEHEIAVIIDEVQTFGRTSRLFAFQHYALEEYADIVT

IGKLSQVCATLYKTDYKPRPGLLSQTFTSSTQAIHAAKSIIRLMIEEGFFGKNGKNNQVH

RQFVKRFEEIRNISGPYGLGAMVAFTPLNGDAKKVKAFVDRLYHAGVLSFICGKEPTRTR

FLVPVGAVSNLDIDNVCTIVEEMLQCS

>tr|D6YV39|D6YV39_WADCW Putative transposase OS=Waddlia chondrophila (strain ATCC VR-1470 / WSU 86-1044) OX=716544 GN=wcw_0632 PE=3 SV=1

MNPSCPKCESTAVKKNGHIHNGKQNHRCLVCGRQFVLDPQQKIITDQTKSEVRQALLERV

SLEGICRIFSVSMPWLLSFIQQIIYELPDDLNATVVKDYKDFEVAIIELDEQWSYVGNKK

NQQWLWLAFHSASRQVLAMHVGKRDKRAAEALLAKLPEDLKKKPSFTLIGSLCTTKSFLG

SNTRQSEKIQEKRVTLKDLTTP

>tr|D6YUL9|D6YUL9_WADCW Uncharacterized protein OS=Waddlia chondrophila (strain ATCC VR-1470 / WSU 86-1044) OX=716544 GN=wcw_0458 PE=4 SV=1

MAVELRVLGDPPEEIGSAGRPARYLTAKTTSSKKRSSTP

>tr|D6YWG9|D6YWG9_WADCW ATP synthase subunit c OS=Waddlia chondrophila (strain ATCC VR-1470 / WSU 86-1044) OX=716544 GN=atpE PE=3 SV=1

MDVNTAFALATPLAVGIAAFGSGIGLGLAVKGAMEAIGRQPEASGKVLTTLIIGAALIEA

LTIYALIVFFVIIGRSG

>tr|D6YTD5|D6YTD5_WADCW Uncharacterized protein OS=Waddlia chondrophila (strain ATCC VR-1470 / WSU 86-1044) OX=716544 GN=wcw_0019 PE=4 SV=1

MRNKMTPQYLYKILPVENWKKSRGMDSAKLTDADQDFIHFAREDQLDRIVGKYWDKVPEY

VILKIDIAKLPGKLIFEANPGGENKYYHLYGGSIPLEAVIESKIIKK

>tr|D6YWE9|D6YWE9_WADCW Uncharacterized protein OS=Waddlia chondrophila (strain ATCC VR-1470 / WSU 86-1044) OX=716544 GN=wcw_1103 PE=4 SV=1

MNPLFEKKGYLIALIAFIATFALFYNWTSELLYCLIASIINASIIWGTYLIIRMCYLASR

>tr|D6YSG4|D6YSG4_WADCW Tail-specific protease OS=Waddlia chondrophila (strain ATCC VR-1470 / WSU 86-1044) OX=716544 GN=tsp1 PE=3 SV=1

MMKRFLYFLVAVLIPVSVFSEQEELLTKKDVHEVMEKILSQHVDYHQVSGEIFTKAFQLY

IDQFDPNRVYLLQEEVDKAVNVSPIQMKRIISQYRKGQLDKFEEIDQLIQNGIHRTREYR

KEILKNGDALFQSSSNKSRLNFQEEKSSFPKNSSELKNRIKEQLVNYINVERLRYGDKTV

MKNRERILQSFMETTEHWENQYLYVDSQGKKLNSLEQENLFAMHVLKALAGSLDSHTSFL

DQNEALDMKIRLEKGFKGIGVVLQQKPEGIVITRMIKDGPAMKSGVIQVGDRIVNINGNN

VEYVSLEELVGKLREQKGSSVSIELKRTVDGKDILIKANLPRELITVEEGRVKAEYEPYG

DGIIGKISMDSFYQGDHGVTSEKDMRRAIKDLEKQGKLEGLIIDLRENSGGFLNQAVKVA

GLFITNGVVVVSKYSNGEERFYRDMDGKVSFDGPMIILTSKATASAAEIVAQALQDYGVA

LVVGDEQTYGKGTIQSQTVTDGSSASHFKVTVGKYYTVSGKTPQVSGVKADIIVPSYYSK

ELIGEEYLEYPIASDTIPSSFQDQLADVDPGLKPWYLRYYMPSLQKKKTSWSSHLNFFQA

NSRKRLAQNAVYQKFLKGELDWDPLQKNEELPENLDFQMQEAVSIMRDMIRVDKPLFNKG

IADD

>tr|D6YRI5|D6YRI5_WADCW Queuosine biosynthesis protein QueC OS=Waddlia chondrophila (strain ATCC VR-1470 / WSU 86-1044) OX=716544 GN=queC2 PE=4 SV=1

MRCKQALVVHSGGMDSSLCLKLAIDRHGADQVLSISFRYGQRHSNELVQAAKICRDWGVD

HCVIQLDCLQEITDNALMNTEIAIEHSQGRPPNTLVLGRNGLMARLAAIHANHLGAEKIY

MGVIEVEGANSGYRDCSRDYMDLMEKILRIDLDNPRFEIVTPIVQMTKCQTMALADQLGV

LDYLLKETITCYEGIGGRGCQHCPACKLRNEGADRYKRLSIGYVLTKN

>tr|D6YTI2|D6YTI2_WADCW Putative NAD-dependent epimerase/dehydratase family protein OS=Waddlia chondrophila (strain ATCC VR-1470 / WSU 86-1044) OX=716544 GN=wcw_0067 PE=4 SV=1

MNILFTGASSFTGMWFAKELSSQGHAVTCIFQKSQNTYQELRGKRVAAVLTRCISHFNCK

FGSDSFLELVKSSRWDLLCHHAAEVSCYKSSDFDVSNAVKKNTWRLEKILQSLKERGCCR

IMLTGSVFEQNEGEGDNIEQAVSPYGYSKGLTSEIFRFACEQNKIQLGKFVIPNPFGPYE

EQKFTTYLAKSWLSRKIPEVSFPNYVRDNIHVSLLAKLYAKAAASIDRNTSYQQWNPSGY

SESQAKFTERFAKEMEKRLSVPCAFTLKEQTEFPEPKKRINTQPINIETWSEKEAWDQLA

SFYSHYYG

>tr|D6YST9|D6YST9_WADCW Uncharacterized protein OS=Waddlia chondrophila (strain ATCC VR-1470 / WSU 86-1044) OX=716544 GN=wcw_1795 PE=4 SV=1

MLLNISSFHLQFPFIYQWIIEIKKYLSMKNF

>tr|D6YTD0|D6YTD0_WADCW Uncharacterized protein OS=Waddlia chondrophila (strain ATCC VR-1470 / WSU 86-1044) OX=716544 GN=wcw_0014 PE=4 SV=1

MLFVNNLQLINVPSKSYLPKFSQGNSEELSKFPQPLMKKINTYI

>tr|D6YUN7|D6YUN7_WADCW Putative membrane protein OS=Waddlia chondrophila (strain ATCC VR-1470 / WSU 86-1044) OX=716544 GN=wcw_0477 PE=4 SV=1

MNNLFYVHMERKNRKEISYSFILIPTFLWIGFVCSISFMEAWLKFRAPNVSLSIGLGIGK

LIFSALNIVEWFFLITTLMLILFNKKILKFRKTKILIIPILILLSQTFFLLPLLNERTDI

ISQGMIAPKSSIHVFYILVEFIKVISLFIFGLRIIYDSAYEISSN

>tr|D6YUW2|D6YUW2_WADCW Putative membrane protein OS=Waddlia chondrophila (strain ATCC VR-1470 / WSU 86-1044) OX=716544 GN=wcw_0553 PE=4 SV=1

MSTNLEGHSPFAPYLPYIIPPVSAGASIIPVFRGFIIKSAQQLGKPAPRMTVFRGLWEGL

RASPTIGAVIGAQLVIQEIAEKKLFTPPIEGQQPSLLSMLVSSAFVGTLSAPGLAVFNGQ

SMGKSLMQSLKGLSLLQTAAIVARETCFLLSIRISDPVSNHMKNKFGDHPSVIYGSTFFS

AAFGSLISHPADTALTCWQKGIQVNNLSHAMKGGPVKALAVGSFAVAYKITKEALTILLS

TQK

>tr|D6YTV3|D6YTV3_WADCW Tyrosine/tryptophan transport protein OS=Waddlia chondrophila (strain ATCC VR-1470 / WSU 86-1044) OX=716544 GN=tyrP1 PE=4 SV=1

MNSVQSNYRVFGATLLVAGCCIGAGMLGLPVLTALGGFLPTCLLFFFCWLFMAATGLLLL

EVNLWFKEEVNVVTMASRTLGPIGAFMAWFLFAFLFYSLMVAYISASGQLIADRLQTLTG

VIVAEWAGSLFLTLLFSVFLYLGTTMVDRVNRLLMLGLAASYLILVCVGSRHVRIDLLSH

VDWSASAYAIPAMIVSFGFHNLVPSLATYLDRDVKKLRFSIVAGSAIPLVIYLMWEWLIL

GLIPLDGENGFRQALGQGDMATRALRNIVGSAWVVDLAEAFAFFAIITSFLSVSLSFVDF

LADGLHIRKTGSGKVKLCVLSLTPPFLFALFYPGIFLKALSYAGAFGAVILFGVMPAAMA

YQGRYRKNLQGPLLIPGGKGVLALIFIVSIAIVCLEMVTTR

>tr|D6YU44|D6YU44_WADCW DsbD_2 domain-containing protein OS=Waddlia chondrophila (strain ATCC VR-1470 / WSU 86-1044) OX=716544 GN=wcw_0280 PE=4 SV=1

MSLLFTMLPVYLLGNLHCLGMCGPIVAFLGKHRFRYAYFLGRLLSFSFAGLAAGELGAVF

HIWLKEYNLSALVSIGFGFAMACIGLTILIGREIPGNRWLGSRLAQINHSLSLLLLKDSF

FSTALFGFATILLPCGQTLVVYSACALSQSGSAGLLNGFAFGLLTTPSLWLAMHASRVFG

KWKGIGNRVLGGCAMIVGLLAVLRGVAEFAWIPHFILNEKYHIALY

>tr|D6YUG4|D6YUG4_WADCW Uncharacterized protein OS=Waddlia chondrophila (strain ATCC VR-1470 / WSU 86-1044) OX=716544 GN=wcw_0403 PE=4 SV=1

MILMSNEVDNYKIYVDQLRDGKIESVGCFFPPEMMDVQDDALAFDARIHVKGEAYVSDHA

LILHLDLHASAVIPCSICNASVDTPVVVKGIYHLVPLDEIKGHIFDIREVVRENILLNTP

LFSECNEGNCSERQFLKKYLKSDEDLQQKPFEGQQPFKDLPFM

>tr|D6YVS7|D6YVS7_WADCW Cytokinin riboside 5'-monophosphate phosphoribohydrolase OS=Waddlia chondrophila (strain ATCC VR-1470 / WSU 86-1044) OX=716544 GN=wcw_0872 PE=3 SV=1

MTENKNFNYSTSHADSWRVFRIISEFVDGFESLMQIGPSVTIFGSARLQAVNPYYNMAID

VAQKIAERGFAVITGGGPGIMEAANKGAQSIGSMSVGIGIDVPYENDTNRFIDPRYRLKF

RYFFIRKVMFIRYAKACVFLPGGLGTLDEFFETVTLIQTQKIKPFPIYLMGTEYWSGLID

WLKKTVLEMGCMSEKDLDLFILTDDPVEVANGIERHYAQDQSLENF

>tr|D6YTB7|D6YTB7_WADCW Transposase OS=Waddlia chondrophila (strain ATCC VR-1470 / WSU 86-1044) OX=716544 GN=wcw_0001 PE=4 SV=1

MRDNWLANRFFDGYDSIVNACCDAWNWFVGTPNRIKNLCSRSWTKF

>tr|D6YSE2|D6YSE2_WADCW DUF202 domain-containing protein OS=Waddlia chondrophila (strain ATCC VR-1470 / WSU 86-1044) OX=716544 GN=wcw_1642 PE=4 SV=1

MSLEADHNTVLANERTYAAWVRTGLTALATGLGVERFLGGVIPDPVIRAISMSLLLFSVL

SFILGAQRYVHVGAIVSKNTKAGAPIPLILLLSILLVLVALLAVIGVWL

>tr|D6YTA7|D6YTA7_WADCW Uncharacterized protein OS=Waddlia chondrophila (strain ATCC VR-1470 / WSU 86-1044) OX=716544 GN=wcw_1969 PE=4 SV=1

MVWYLAVTILFALAGMLIYIYYLRNGQFDDMEDVKYQMFRDEE

>tr|D6YWW3|D6YWW3_WADCW Putative membrane protein OS=Waddlia chondrophila (strain ATCC VR-1470 / WSU 86-1044) OX=716544 GN=ompA7 PE=4 SV=1

MMSIKKTMAAIFLSVASLTTSVQANYWGSCNDCCPDVTVGVDWLYWSPCASDRHFALTTE

GIDSEVNVKTHYLCDEWDSGVRVYGKLGNLWNGFNGGLIYTYINPKTSGSVEQIGNNGVV

FSFGLPIPGESGGLLQGSRLDAKWELQYQTLDAILSYSIDVTQNRCFKIEAFSGLTWVDV

KQKASYTLDQVGGNEIEIRVDFDRNNDFWAIGPCFGLNTSFRFCDCFNIFGLVKTSLVVG

ESKNKDVIYGTRGSSETSTFVTVTAKDKCFCFPGLHLATGIDYELCLCNITLDLRLGWEY

VQWINAPTFPYYELDGLGVRSAPSTNDLTMQGIFLGLNTTF

>tr|D6YU46|D6YU46_WADCW Peroxiredoxin-4 OS=Waddlia chondrophila (strain ATCC VR-1470 / WSU 86-1044) OX=716544 GN=bcp PE=4 SV=1

MEAPDFTLKNQNNDSVSLKAFRGKWILLYFYPKDNTPGCTTEACDFSRLKKNFEDLNVKI

LGVSPDSVSSHQTFIAQKNLLIDLLSDPEKTSIQSYGAWGKKNNYGKVYEGLIRSTFLID

PEGKIAASFKNVRATGHAERVLKKLQELQHE

>tr|D6YTX8|D6YTX8_WADCW ADP,ATP carrier protein OS=Waddlia chondrophila (strain ATCC VR-1470 / WSU 86-1044) OX=716544 GN=ntt1 PE=3 SV=1

MNHNKDVKEFSFWRGVFFPVHLHELKFFAPMALMMLFILCNYTVLRNIKDTLVVNAKGSD

AEIISFLKLWGTMPAAFLFMLFYSKITDLLSRERIFYLCLAPFLIFFGSFAFLIYPNIDI

LHPDPEWVAGLKANYPNFKWFIAIWANWSYSLFYILAELWGSVILSLLFWQFANDTVKKE

QTKRFYPLFGMVANIGLVAAGSMVKYFSKVETAASDPWQVTLNGLMPIVVISGMIIAGLF

YRINRRLENRQAEEVVVKSEKKAKPKVSLAESFKILFTSPHLLCIAIVVLSYGVSINYVD

VIWKGQAKAYFAGDKNAFANYMGNFAITTGCLAIPIMLIGGNILRYLSWKSAALITPVIL

LVTGFGFFSLILTGYWHGDPNAALFTFMGVPVTFLGLGVQFGLFQNAVTKATKYSLFDPT

KEMAYIPLSKDFKTKGKAAVDVVGSRLGKSGGALTFFIIQTILPNVSLVQLIPLLAVMTV

LILCAWIAAVFKLSSSIKELEEQSSAVAKKGELEALGQAA

>tr|D6YSN2|D6YSN2_WADCW Uncharacterized protein OS=Waddlia chondrophila (strain ATCC VR-1470 / WSU 86-1044) OX=716544 GN=wcw_1736 PE=4 SV=1

MDKLFYEAIPPPIKWKAEQVEEWTSELLIFLKKEKIRSINIPEVVGESKEQERTVPLIAK

IETLDFIDRLQNHSFPITPIPNLITVHRSEIQLIEWVENAYRKGVRHLILVGGEKSSISY

PGLQVTTAARLLRNRFPDLKLGGISIFTRKNEPDRILRKMESGIEFFISQIIYETANMKC

VLLNLAKLCQAAKLPLPRVYLSLAPAAKLTDIAFLQWLGVEFPSALSSYFSGGGDECVEE

RVDEMIDFVLEELKYFISRKHFNLGFNVEQVMYHNFPSAQRLIKHMKQRLSGCL

>tr|D6YX06|D6YX06_WADCW Virulence plasmid integrase pGP8-D OS=Waddlia chondrophila (strain ATCC VR-1470 / WSU 86-1044) OX=716544 GN=wcw_p0001 PE=4 SV=1

MGKIMTLEVVQEHKIESQAQAKSLRDKIAWQKMEMLSLDSIIVEWLETLSERTKVNYKSG

ISKLTELGIINPFISLQSFSLVNHDAAIDRIKQLPDLKETTKQARAALYISFTRYLSRRF

KGMFSKATPSREGNEKTFFRVHEKVVTEAMNLQQWTAFFEELEKLNIRDALIGKIALQGG

KRIREVLSLHSEQIDWKKREITFHQSKTRGVSKQTIITYSESIMKQLKDLLGEGEGYLFI

TTTGNVVMLTQISRNFARAGKAAKIPFKVTPHVLRASAVTYLKQQGFSDSDIMKITGHSS

GEMIRAYDKSERADNPTKRVNLVM

>tr|D6YS89|D6YS89_WADCW Putative DNA repair protein recO OS=Waddlia chondrophila (strain ATCC VR-1470 / WSU 86-1044) OX=716544 GN=wcw_1587 PE=4 SV=1

METIRAEGIILQSLPVKDWDLLITAFTKEFGVMKFYYKKGQSKKRSKGALTSPLTCAEFI

CRKGNFDLIPLNEISVIDLHIDLRKTLEFLEYSCSWLKLIQRSQLPGKPAPNLYLLLKAF

LHSLPQFPKPETLHACFLLKLLRHEGLIELNPFCSVCSGPAAFQAGEPFCASHCSTNAIP

FNANELDLMQTLTFCRSLKEIENERIDFSFLEKISQLYRRIAEDFSTC

>tr|D6YWF5|D6YWF5_WADCW Putative pre-16S rRNA nuclease OS=Waddlia chondrophila (strain ATCC VR-1470 / WSU 86-1044) OX=716544 GN=wcw_1109 PE=3 SV=1

MKTRIIGIDYGMKRIGLAISDETHLIASAIETFEASKKMEETVAALVKRLEEHQKEKGYE

LKEIVVGFPLKMNGKAGFISDEVTLFAETLSKALSIPIIKWDERLTSVQAERAMMEGSYN

RKKRTKKLDQISAVIILQNYLDSKNLII

>tr|D6YWA6|D6YWA6_WADCW Urocanate hydratase OS=Waddlia chondrophila (strain ATCC VR-1470 / WSU 86-1044) OX=716544 GN=hutU PE=3 SV=1

MTLTSFMEKYAKHPSYKSPVGNQLHAKSWQTEAPLRMLLNNLDAEVAEDPNQLVVYGGTG

QAARNPEALTKIIDALLTLEEDQSLLIQSGKAVGIVKSHPEAPRVLIANSNLVPHWADWE

HFQKLKERGLIMYGQMTAGSWIYIGTQGILQGTYETFAEMARQHFGGSLKGKFVVSGGLG

GMGGAQPLAAVLNGAVYLGVDIDRSRIEKRLKTRYLDTIADNLEDAMAKITEAKKKGQAL

SIGLEADIGSTLHRLLEKGMVPDAATDQTSAHDPLYGYIPSGLTLSEAEALRKENPTDYL

LRSKESMASHVNALLAMKDQGTIVFDYGNNLREFAKEGGSSRAFEIRGFVPEYIRPLFCE

GKGPFRWAALSGDPMDIYKTDEALMRLFPENSHLHHWLKSARERVEFQGLPSRICWLGLG

EREQAGLLFNQMVESGELSAPLVIGRDHLDSGSVASPNRETEGMRDGSDAISDWPLLNLM

ANTAGGATWVSFHHGGGVGMGFSQHAGMVVLADGTKRAYRCLSRVLFNDPATGVMRHADA

GYETAQRLFQENQLG

>tr|D6YRY1|D6YRY1_WADCW Permease, major facilitator superfamily OS=Waddlia chondrophila (strain ATCC VR-1470 / WSU 86-1044) OX=716544 GN=wcw_1477 PE=4 SV=1

MNRVSHEGLSLAAFGICFGCVVIALVFTMVNTAIPSIQSHLNLPVSILQWMMTAFGLINC

ACLVTCGRIADIFGRKRIFLLGLTSTLIGISAAGLSNTAFCLILGMSFAGLGNAILLPVS

QAMLVMEFPQEKRGEAVGIWAGCIGAAMAAGPVLGGIITQVFGWRWVFLINAPVILVSFF

LTFFYSRESRNEKDPAKVDVKGMLYLALTVAAFVLVSTERRHFSSLFLSGLMALGVLSIY

RLIKHEKTFSAPIISDQLWKNRVFLSASLASFCLIFFVWSIFFLLPIFFQRELGLSPMFS

GLMMLWITLPVAFLSPLIARFEKYEKTWLMSLSGFLLLAFFVLLQIFFNTDTPLWKIAVS

CLIFGVGYSLIWGPTATAAISCFPSHQSGIASGTFVTIQELGGTFGLAIIATTFSHYSAL

KEGFYRSLWLLLVVSVIGCALSLVMKTRKRAVEAS

>tr|D6YTQ6|D6YTQ6_WADCW Uncharacterized protein OS=Waddlia chondrophila (strain ATCC VR-1470 / WSU 86-1044) OX=716544 GN=wcw_0142 PE=4 SV=1

MYCWKCQKQLDDPPSPRLPFRATCDHCSAWLHCCVNCRNYQPGLPNDCKIPGTEQIADRE

ACNFCDEFSLKETNDLSSSSIEEASKKLFGEETKFEKKNFNDLFND

>tr|D6YX16|D6YX16_WADCW DUF5681 domain-containing protein OS=Waddlia chondrophila (strain ATCC VR-1470 / WSU 86-1044) OX=716544 GN=wcw_p0011 PE=4 SV=1

MIMPENAGKKQGNTRFRKGQSGNPCGKAKGTKNKATLAAEVLLEGELEGICRSLIAQARE

GNIQAIKLVLDRVLPPKRNPTVSIELPQLKSSSDALEAITSITQAVGNGEISPAEGETLS

RIVDVYVKALEAHDFENRLNILEEKGK

>tr|D6YU19|D6YU19_WADCW Uncharacterized protein OS=Waddlia chondrophila (strain ATCC VR-1470 / WSU 86-1044) OX=716544 GN=wcw_0255 PE=4 SV=1

MKKIIVCSLFIILSIFPLFTGCWQMEFSSRQFKSKVGKEIYTGLSTPAHVKIEGMRTLTQ

SEIEKLQSFLLEDKGYIFNRTKKCLFIPEITVTFEGKEQVVAMISFVCKQIKLIHDDQTV

ILDIDPMAEVFSNYIQKELIK

>tr|D6YTR6|D6YTR6_WADCW Putative ribosomal protein L11 methyltransferase OS=Waddlia chondrophila (strain ATCC VR-1470 / WSU 86-1044) OX=716544 GN=prmA PE=4 SV=1

MDNFLIFEAELSAEEFFLFEETVGDLVESLNWEGGVVKGMACIDNRVELELRAGRPLAWS

PMESVDWLEEDEKKLPPITVGRLFICTSYYTGPIPKEKVLLKLHSSYAFGSGHHPTTEGC

LEAIQMLPNVNRALDIGCGSGILALAIEALFGAEVVGSEIDERSAEMARENACGRIKVVH

GNGVSSETIIERAPYDLIVSNIHSGPLIDMAQSIASLLSKEGTLILAGLLREQREEVEQA

YALQGLVFNGVHGNDPNWPALQFKKK

>tr|D6YRW3|D6YRW3_WADCW Diaminopimelate epimerase OS=Waddlia chondrophila (strain ATCC VR-1470 / WSU 86-1044) OX=716544 GN=dapF PE=3 SV=1

MQSSTPTKAFELRTSIPFSKYTGCGNDFILIDNRNQVYPSEESDLAEHLCDRQFGVGADG

VILLERSDRADFVMRIFNPDGTEAEMCGNGIRCLGKFLQELGIEGTRFTIEVMGKKYPLS

LHDDGSVSVSMQPAPEISWDIELSIGRKTFHVDFLDTGVPHATLSVEKLSAFDLDAIGPK

IRYHEKFMPQGTNVDVYHIDESDPSLIYIRTYERGVERETMACGTGATACAIIAWTKEQI

ANPIRIQVASGDILEFKISGSLGRIDQIVMKGPAEFIFRGNFSI

>tr|D6YSE8|D6YSE8_WADCW Uncharacterized protein OS=Waddlia chondrophila (strain ATCC VR-1470 / WSU 86-1044) OX=716544 GN=wcw_1648 PE=4 SV=1

MPIFRTLFILAALTIGFWTLWEHHSTFREIVQTYVENGEFLTLEARYSADQIMQAHEKEL

IPDAQYSYQDPALKFYPYLLMEVKYTQQGRTREGVILWSMVDGEMVLDTDSWEKTHGFED

AINAGANRSDFVLINTLAKYRGSLSSARLQKELNLDDQAFNQLIENSRKKYLIIIRGNEV

ALHFQNPNFLVPPQTRINQWLVTKPYHHAIRVGKRYSQKQIEKVAKAAFGYDFTVRSAKE

VFLPVYSIEVLNPDGSILTSYWNALNGHRIDNKYLSLSP

>tr|D6YT65|D6YT65_WADCW Methylmalonate-semialdehyde dehydrogenase OS=Waddlia chondrophila (strain ATCC VR-1470 / WSU 86-1044) OX=716544 GN=mmsA PE=4 SV=1

MTTMYPKIPKIGALKPAYLLINGEWIESVSPHRIKNLNPATQEVLGEIPMCTEKEVNQAV

QAAFNAYSYWREVSPSQRARTMFNYQQLIRENIEKLAHNISLEQGKTLADAKGDVTRGLE

VVEYAAGTPSLIMGESVENVSIGIDTYSLQQPLGVCAGITPFNFPAMIPLWMFPIAIASG

NTFVLKPSEQAATTANMLAELAQKAGIPNGVLNVIHGGKEVVNALLDHSLIRAISFVGSP

HAGHEVFARATSQGKRVQALLGAKNHAVVMPDANKEKTLNAIARAAFEASGQRCMALPIA

IFVGESKKWIPELIEKAKKLKVGPGLDPSSDLGPLISPEAKERVLSIFESALQEGAELLL

DGRQLQVPGHENGNFVGPTIFNKITRKMRIYEEEVFGPILLLFESTSLDHAISIINNNPF

GNGSAIFTESGHHARHFQHFVDTGQVGINIPIPVPLPFFSFTGSRGSIRGDLHAYGKHAI

RFYTQTKTVTAKWFGTESRNYEKIYEETPV

>tr|D6YS09|D6YS09_WADCW Putative membrane protein OS=Waddlia chondrophila (strain ATCC VR-1470 / WSU 86-1044) OX=716544 GN=wcw_1505 PE=4 SV=1

MNVSSVCAPQYPIENGTKKNLLETVEHGICSIAKNVFEGMRLLFVRSGQIRTNANAFGRV

VQLGVHLFSLAEIALMKPGSYAHISSRLGVTKNIIDTVQTLDGVHYFGCKISEKTAAKSL

GNAAMFVASVGLGMEILDKCKLLNLGRIAEKIGSVPVLGAATRLGVSFGQVCCGFAAFGY

IFFAVDAIERLIEAEDGDKRRQAWIDLAWLVAEIAATCFLIFASSCMVGVIGFGITACVL

GTASYLHGIAVRENAAALR

>tr|D6YTH4|D6YTH4_WADCW Putative membrane protein OS=Waddlia chondrophila (strain ATCC VR-1470 / WSU 86-1044) OX=716544 GN=ompA1 PE=4 SV=1

MMKRCFLFLSVFVCCCFFLSRVDAGNCCASQSYAPGFRETPLYRPSCCNYVDSCSYECDR

QCSIEIGAEFLYRKVCLDEFNWAFTRTETTEANVTSIDLSYERLCLDDEPGVRAWLGFQP

AGKESLGIYFGYVYLSGSKNDSIQQSFGNTIGTTVMHYGLFQESAGFTDIAVDWRNQYHE

GEVVLGSSAFCGKNHVFLPYFGAAGIYLEQKFALDLSDPDLVFAGDAGAVDWESQYWGVG

LRFGSHYKYQLGSCLAFIGRFNASLLVGENKFHNFQSVIFSSEAEELQLNISGKNKCHVV

PGYSLGAGFAIEPEICGCTLSLRVGYEFNEWYNIPKHRTFSGESTVDGEVAYSDSASNRT

WGSHGLFAGLSFAF

>tr|D6YWA1|D6YWA1_WADCW Uncharacterized protein OS=Waddlia chondrophila (strain ATCC VR-1470 / WSU 86-1044) OX=716544 GN=wcw_1053 PE=4 SV=1

MQMNELNDVLIRALEAIIQEGRSILDPTAYADLMVQINNAADSLEEFNSTALASMETIAI

NCFHKEGSFEECTHIYSFLTIRPRRGSTTKKSNVRNFAIAGF

>tr|D6YUD2|D6YUD2_WADCW Uncharacterized protein OS=Waddlia chondrophila (strain ATCC VR-1470 / WSU 86-1044) OX=716544 GN=wcw_0371 PE=4 SV=1

MYLEDFNEFKETLNSFHQTLRNGVENDSEYEGEADELLGSLERVQEKIKSIENIDQLKET

VGPFGWDFLQVYLAIDEIMAMDDEDFDFEENEENASV

>tr|D6YTL8|D6YTL8_WADCW Uncharacterized protein OS=Waddlia chondrophila (strain ATCC VR-1470 / WSU 86-1044) OX=716544 GN=wcw_0104 PE=4 SV=1

MSTKQKKKLCWNCEGRVSFEEENCPYCSVYLGPAIEKDGKQNVLAPPYQIIEEEEDLDAL

ESPYQIEGDSAAEEVPELDEAKNDMRQVVLPLGMLSAGSLFFLFGLMLLIFSDHGTLTLS

WSADYWYLYVLLALPALFFGWVSLGRSDLQGE

>tr|D6YTV4|D6YTV4_WADCW Tyrosine-specific transport protein OS=Waddlia chondrophila (strain ATCC VR-1470 / WSU 86-1044) OX=716544 GN=tyrP2 PE=4 SV=1

MIIKNKVLGGILLVSGTTIGAGMLALPVVTGLAGFGPTLFLFLLYWIYMTYTAFLLLEVN

LWLGENINMITMAKRTVGRVGELVSWAAYLFLLYLLTTAYLAGGAPVIVNCVKMAVGVRI

PDWVGALPLLLIFGFFVYEGTKYVDVINRILMFGLVIAYSVMTLFMVPHVDYRLLSYADW

GNFWLAVSVAATSFGFHIIIPTLTTYLHHNPVQLKKVILVGSVIPLVVYILWEMITLGVV

PIEGVSGIREGLREGVNGAALLSEQLGSSFVSAVAQMFSFFAIVTSFLGVTLSLSDFLAD

GLHIKKTHAGRFFLVALTFVPPLFFILLSPRAFLSALEYAGAFGVVILLGLMPPLMVWAG

RYRKGLKGSFTVPGGKPALAAVIVVSLIVIIGEAANKLGVFGG

>tr|D6YVZ4|D6YVZ4_WADCW Uncharacterized protein OS=Waddlia chondrophila (strain ATCC VR-1470 / WSU 86-1044) OX=716544 GN=wcw_0944 PE=4 SV=1

MVPLLHLFLFFIPDRRHMSTTLIAIRHAKPLSEGFADERLRPLHKEGISTQEMIVQRLAE

KGYMPEKIFSSPILRAVQTAAIIAKHFSLEVEEEEALGYNFNQSILLANLLQANQGKTLV

FVGHAPHLAEFVNDLTGYPALMHGLSKSEAAILRFDQEIELGKAIFLDNLKL

>tr|D6YU13|D6YU13_WADCW Superoxide dismutase OS=Waddlia chondrophila (strain ATCC VR-1470 / WSU 86-1044) OX=716544 GN=sodM PE=3 SV=1

MANTYTLPDLPYDLGDLEPVINAEIMDLHYNKHHKTYINNLNNLLEQLEEAQSKKDISKE

ISLQSGINFNGGGYINHSIFWTNLAPKGKGGGEAPSGPLADAIMKDFGSLDKLKETMTAK

TVAVQGSGWGWLGYNKSEDRLEIAICQNQDPLAAKGLVPLLGIDVWEHAYYLQYKNVRPD

YVKAIWEIVNWKNVSERYEAAL

>tr|D6YS63|D6YS63_WADCW Regulatory protein RecX OS=Waddlia chondrophila (strain ATCC VR-1470 / WSU 86-1044) OX=716544 GN=recX PE=3 SV=1

MNVEVIPDEQRKEILHLSVDDHFFREIHTAIFGKHPKFSFQEENLPEQFFRKEFERSRFF

VLKRLSQRSYSSFELRFQLQERLVSQETIERVIDDCHHLGYLDDKAWLEQFIRTQLLRKL

GPMMIEAKLYQKRVPKSFYEPILANLVTHEDQEKAIKRLMNTRFRSKDLRDFKERQKVFG

ALMRKGFNPDLIKEILALWLN

>tr|D6YSQ6|D6YSQ6_WADCW BPL_N domain-containing protein OS=Waddlia chondrophila (strain ATCC VR-1470 / WSU 86-1044) OX=716544 GN=wcw_1760 PE=4 SV=1

MARSFHRHEQCLFSWPAAVHPRRNLESDRSNNFHFQTAIIRELNTEVNSEPTLEQQSKQK

LKAIKSVQNQFSNSLQPMAILVYADEGAGPRSVRLLIKALKSLKLHEKYKLQRVNRKALA

ASGWEKDASLLIFPGGRDTPYHQSLKGKPNENIRRYVEEGGAYLGICAGGYYGSAEVEFE

KGHPLEVVGKRELGFFPGIARGSAFGPNLFKYEDESGSQAAHIHWMENSLLPIYFNGGCA

FVDAAAYTNTRVLATYAELPGNPAAVVECSIGKGKAILSGVHPEYAYPHIEHIPTIPSLV

LSTLKNHEPQREQFFRSLLETILASRDNLS

>tr|D6YS81|D6YS81_WADCW Transposase OS=Waddlia chondrophila (strain ATCC VR-1470 / WSU 86-1044) OX=716544 GN=wcw_1578 PE=4 SV=1

MKILLVAKSTSQHAVSMQFLTKQEREQLQAQHRFERDRRIRIINGIIERSL

>tr|D6YW89|D6YW89_WADCW Uncharacterized protein OS=Waddlia chondrophila (strain ATCC VR-1470 / WSU 86-1044) OX=716544 GN=wcw_1041 PE=4 SV=1

MIFFKENIVNVKQMPSPETLHSSLRKCDLYDNPYNTQMQAHPAIVKQRQETFAVQDSISS

SQKKLEKELFESFKQFSMQMFSGKFFIIGQAGKYAFLAIMLPTYLFFYGIPKWLLTEAAP

VVYDFTKRIVSHAGSKIGSAVSHLATAAFEIVRTVTDPILNFIQTRIEKSREFYHNVKQR

IEQIVKSFSKTLISPFQKITQPIVGFYRKLTKSVQLIREKISDFAVLIEKVFEKIKRIPD

RVAEALKIVQEKFKELKEKILQTCTPLLNLFKNLDHQVSKWFKKGLEFKDRWMNTLVYQP

FKAVNETYQAIRDRTVALVRRVSDPLINWVKPKFEKAVEVVDSIKESVSSTIRNLSEKIS

ETFKKYTEEVIKQILPQPVIAFFHSFVSTAGQVIKTPYRLFLYGKSSKRKIKRLKEKLRI

KLVHLNQQLLSFLKKGYQKAKPRCAAFTKKAAKTLMGFFRLIKEMLKGAFYMIRMIFAWL

KILFKQGMITVKETCAGLLPRL

>tr|D6YWV8|D6YWV8_WADCW Putative membrane protein OS=Waddlia chondrophila (strain ATCC VR-1470 / WSU 86-1044) OX=716544 GN=wcw_1267 PE=4 SV=1

MDRFIKKLMAGDIHLRDKWQIELKSEFLPSKHLKKNTYTQEFFFFIPNSLQINEETYPKE

HFYRDRTNFLRYKTPEFTLKELNNLANNRSPLSRLTCLIDSHPCEEAYEETEDELKLLGN

IVRSSIRESVKRFVLQIHSTPPKSPEIKNLCEEIARLRKTFLNLENRFQTEWNIDHLNSY

FYYADEFLSSTINYYLTGLLSEIRDKHPFVSNDTTAPLCQILEKERRHRKTVLHEPSITD

EDSISNEYILYRSSLLNKFILDALLLHTSGYTARKRFSHLIGSVSAGIAMLFFFFLFVHQ

AKVLIINSLPFILLTVFLYILKDRIKESLRDGSFKYLSKWFYDYSTEIYSQKMSKQLGKL

NESMSFLEEEDRVSDQIIDIRNREFHDILETIKRPEKVIYYKKVLTLFNGGLEKDGRRQA

LNLIFRYNIRDFLKKADNPTHDYLKVDPVSFNFIRATLPKVYHINIIIRNTYLNEEEKQA

VELKKFRLIIDKNGIKRIENP

>tr|D6YV07|D6YV07_WADCW Putative membrane protein OS=Waddlia chondrophila (strain ATCC VR-1470 / WSU 86-1044) OX=716544 GN=wcw_0600 PE=4 SV=1

MLKKFFASVITFALLCFTPGNVECYQGCGSSQQCCKDYGGYIPAAGRAYESACNTLCFSP

CLAVGVVAVASLATIIALTNNSGDTGVVIHAHSD

>tr|D6YRN8|D6YRN8_WADCW Single-stranded DNA-binding protein OS=Waddlia chondrophila (strain ATCC VR-1470 / WSU 86-1044) OX=716544 GN=ssb PE=3 SV=1

MVALNKVMIAGRLTRKPELRKTPNGASVTDLLIALNREFTTSAGEKQQEVCFVDVVVWGR

LAENCTNHLDISSPVLVEGRLQLDVWEGKEGDKRCKLRVAAERVQFLEKLDRKNSQEEND

ALAESYVLN

>tr|D6YUS1|D6YUS1_WADCW Hydrolase OS=Waddlia chondrophila (strain ATCC VR-1470 / WSU 86-1044) OX=716544 GN=wcw_0511 PE=4 SV=1

MVKSNEEKPKLVIRNAAVRDVPAIQKLSVKVYKEHYQYSQAELRGQIRHFPEGQFVAEYN

GKVIGYCASLIVHRQKAMRSHSWREITGNGYCSTHTRNGDFLYGVDIFVDPAYRRMRIGE

RFYRERISLCKFLRLKGIIFAGRLPLLKKKFKQVGSAEGYLQAVLDKKIRDPVINFQLRQ

GFEILGILEKYDPKDTESMGYAAHMIWYNPEMSSQDATESGNRSGSDALRVASVQYMQRE

IGSFDEFKKIITYYIDVVHDYRCDFVLFPEFFTLQLLSIESEKLSPEKAILRLAEYTEDL

KEFFKDLAIKYNTNIIAGSHPVRVSDTEVQNICFIFLRDGSVHEQPKIHPTPDEKYWWNI

TGGDKLEPIETDCGAIGVLICYDSEFPELARYLVDQGANILFVPFCTDQRQGYLRVRYCS

QARAVENQCYVVMSGNVGNLPKVENMDINYGQSCILTPCDFVFSRDGIAAECEPNVETII

FADLHLNDLFESRSSGSVINLRDRRHDLFSVVWHKKH

>tr|D6YVV6|D6YVV6_WADCW Uncharacterized protein OS=Waddlia chondrophila (strain ATCC VR-1470 / WSU 86-1044) OX=716544 GN=wcw_0905 PE=4 SV=1

MSNPLKMAQEEPWYAEGLAFECTGCGNCCTGSPGYVWVNEQEVEAIAEYLKIPIDKFRKT

YLRQKEGRFALVELKYKNYDCIFLKDNKCSIYPVRPVQCRTYPWWIANLSSKEAWENAAK

TCEGMHCKSPIVPKETIENELAKNP

>tr|D6YTE3|D6YTE3_WADCW Uncharacterized protein OS=Waddlia chondrophila (strain ATCC VR-1470 / WSU 86-1044) OX=716544 GN=wcw_0027 PE=4 SV=1

MKFFPFLRLRIQVAFLFVFHLSAIHADDAIPKWKQTLQSLTTTEKKSLEIYFKTMLEDSE

GGFVLFGSKPVCSEGIMHNPKCILEWIGGKRHEKSVNLWEGSLVWQRYFASLNSARLIIS

FKDHPDSLYPEWRHLIWINPDKLQEVVEKNLILFQYVLGPDVTETTLIDILRDNSKDFLS

DYALLGIALGFGTQNSLHYRRIECIARSLNSRELLPLKNRFERLQISDPFKGRYMGFDFE

PSFETNPAFNFSTLLQEYQFLCDKQLSSRKMGCEKIPLFSVFNKNDEETDQILKAYETHS

SKIQALLESDCFLEKILQEIFE

>tr|D6YSS5|D6YSS5_WADCW Putative Undecaprenyl phosphate-alpha-4-amino-4-deoxy-L-arabinose arabinosyl transferase OS=Waddlia chondrophila (strain ATCC VR-1470 / WSU 86-1044) OX=716544 GN=wcw_1781 PE=4 SV=1

MRRLCLFLGVKALFALFFIHSGLLGLGPDEAQYWTWSKALDWGYYSKPPGIAWQIWMGTQ

IFGDTELGVRFMAVVLNFLIPIAIYFLAKSCRLSESAAFWAAIAFAFSPLGILGSLLATT

DGGLVLFWTLGCLMIVQGRVLLLGFVIACGALFKWPIYLLWVFVPFFRKMRSLKLLWGVA

ISLLGLLPSVIWNWQHDWATFQHVFNTMKGGHAHTASSGNFWDFVGAQAALISPILFILL

ILAVISLFRRLDEMRPSLLFCGAVTAILLLIYSLMAIKQKMQGNWVVFAYPTGFVLLSWY

ALERAKKWLIGGVVLSVILTVVALWLPFSGWLPYKMNPFKHNLAWERMEEALLEAGYNPK

NDFLFSDKYQMSSLLSFYSPEKKRAYFLNLQGVRKNQFSYWPSMADEQLNQNGYYVAAEN

FPHLEQLDPKQHVERLSPYFSTIDLARIVPLIKQDGKVVKALLIVRCRGYRGQEPKDLAL

Y

>tr|D6YVY0|D6YVY0_WADCW Uncharacterized protein OS=Waddlia chondrophila (strain ATCC VR-1470 / WSU 86-1044) OX=716544 GN=wcw_0930 PE=4 SV=1

MNRPGTQGTFFGSFSRDILGDTSWRLGDYVCDN

>tr|D6YUF4|D6YUF4_WADCW Uncharacterized protein OS=Waddlia chondrophila (strain ATCC VR-1470 / WSU 86-1044) OX=716544 GN=wcw_0393 PE=4 SV=1

MKEFFKGFAASAAQVGAACVAKWAVVGAVTAVCSPAGAALGSALTAYSVGMTAYSVGKCA

YDNYDTLQEIGDAALAGDLGQIAAWGIDAVCSMSYEQLGSLAFDALSIAAPVARVKGAKA

ASVAAKVYMVVGTTKKSLPTKLDLDALSRAGQKWDRG

>tr|D6YSK9|D6YSK9_WADCW Putative chromosome-partitioning protein parB OS=Waddlia chondrophila (strain ATCC VR-1470 / WSU 86-1044) OX=716544 GN=wcw_1710 PE=3 SV=1

MSQEVVEVELSQIRENPYQPRKQFNREELEELAQSIQSVGVLQPPVVRKMENGGYELIAG

ERRFRAAEIAGLTTIHVLVSQKPGNVSAEAALIENIQRVDLNPLDIAQALRRLIVEFGLQ

QDELADKVGKKRSTVTNYLRLLSLPHKIQESLQFGEISMGHAKAILSVSGFEEQLYLHRM

VVEDGLSVRETEEAAAKLNVKPVKKKMRTLNENTVYLSDLEEKLQQRLGTKVTIASSGKR

GKILIDYYSLDDLERILEVISL

>tr|D6YS56|D6YS56_WADCW Uncharacterized protein OS=Waddlia chondrophila (strain ATCC VR-1470 / WSU 86-1044) OX=716544 GN=wcw_1553 PE=4 SV=1

MTITPFNNSSNSILHFEGTAQKVGAILPQSSKISFESDLDKKIEKARKHIDYLSTSLPHR

NTHGGKRYRARRASINQQIHLAKERLETLSRAKESIRNSASQPVHQETSFSYLNFLWKWM

K

>tr|D6YT64|D6YT64_WADCW Uncharacterized protein OS=Waddlia chondrophila (strain ATCC VR-1470 / WSU 86-1044) OX=716544 GN=wcw_1922 PE=4 SV=1

MGTQSLTDNIYYSVRNAASAGWEIAQSCVGVRQNASTTEKVYRIYNASVAALVAAGWAYE

IGSSVLDISSTSGPYESAWKTITIFLATGEYLSDAAFHAIASQVSENSSTEFKAATIALD

ILRLGIIGKLQMTQPPLSTIPPALNYTDIFNHLGNIAYNTGLISLFCPSKKEHNE

>tr|D6YRI3|D6YRI3_WADCW Uncharacterized protein OS=Waddlia chondrophila (strain ATCC VR-1470 / WSU 86-1044) OX=716544 GN=wcw_1326 PE=4 SV=1

MFAIFGALASCSTRMLTVHSDYVTRESLASFYVDTPDPLLNCPPFGQRLVISWSVPKQLM

ELDDPHLRLQLRFRNREQIDRKIPLRRAKGTTVFRILNDEYCKTGGFLTYRVQLIAGECV

YEESRHQLWEELIAIDRNDDESGTINFVDQPLKPYLLEHYLPLPF

>tr|D6YRZ2|D6YRZ2_WADCW 50S ribosomal protein L6 OS=Waddlia chondrophila (strain ATCC VR-1470 / WSU 86-1044) OX=716544 GN=rplF PE=3 SV=1

MTRKAKVPVDIPNGVEVKVDQEKISVKGPKGQLEQKIMKGINVIVEEGKVHVQLDQNAKD

MKNFQGLYHALIKNMVHGASQGFEKHLEMIGVGYRAAVQGNLLNLQVGLSHPTNLTIPQG

LEIKVEKNTKIIISGPDKQQIGEFASTVRSIRPPEPYQGKGIRYSGEYVRRKAGKSAAKK

>tr|D6YT76|D6YT76_WADCW Ribosome-recycling factor OS=Waddlia chondrophila (strain ATCC VR-1470 / WSU 86-1044) OX=716544 GN=frr PE=3 SV=1

MSIIDQTEQKMKASIDHLTDELKKIRTGQANPAMLENVTVEVYGTSMKLRDVASITTPEP

RQLLVTPYDANNTQVIGKGIEKANLGFRPIVDANVVRITIPQMDESVRQEMVKLCHKKRE

EAKVSIRNVRRDSNEAVRKQKTDGEIGEDMEKKFEKDIQILTDKYCKIADDVSSVKEKEV

STI

>tr|D6YT16|D6YT16_WADCW Uncharacterized protein OS=Waddlia chondrophila (strain ATCC VR-1470 / WSU 86-1044) OX=716544 GN=wcw_1873 PE=4 SV=1

MRILYLLILTAPFLLFAEDSWTQELPGGFRASVHVPKTRISIDEELILHLALEYPSTHLP

DLDTIRMNLLKYVGVTEPPFALIGEKVEEAGKGAMKITFRMEPQLAKIHFVSFYDIPFVP

LEKGPTKKIISEIFEIDVFLPKIDPAYHGHAIGLLSLTEPLPIGMDIKNTQRIEHPDRIQ

EEAKRSVSIVSSRSIPWTQMMGVLLFCIIVFIARMQPKKIPDPKKTIQKQAASAKLKALD

TIRTLDSAEKERFYIDLTNTVRTFIEEKYQIKATSQTTQEFLHAMANHPSFDNETQAMLS

DFLISSDRVKFADQKPSEDDCNKALQTAEQFISK

>tr|D6YSH3|D6YSH3_WADCW GBBH-like_N domain-containing protein OS=Waddlia chondrophila (strain ATCC VR-1470 / WSU 86-1044) OX=716544 GN=wcw_1674 PE=4 SV=1

MTALQIKEIRQKDNYHFSIEWTDGKVIDYRLSDLQKMCPCANCVDGSTGERKTNAKFVDE

QVRAVKIASVGRYAIRIDFTSGCSMGIYNFEDLRFI

>tr|D6YRU2|D6YRU2_WADCW N(6)-L-threonylcarbamoyladenine synthase OS=Waddlia chondrophila (strain ATCC VR-1470 / WSU 86-1044) OX=716544 GN=wcw_1438 PE=4 SV=1

MLTLVVDVCSDRGIAAFIRNGKVGYFAGLPLGLHNSKFLMPKIEEGLKVLEIQSSQIELI

AVGIGPGSYTGMRVGSMVAKALSFAHGIPLIGVSSLEGYLSDHQGIFAAMIDAKMAGCYL

MKGRKKGNEVNYLTGPEVWKMEHIGSVLKDVDVIVSPHCRSLQEKFQKNDLNRQWEWQET

AINVEHFASRAEKKYRDGEFSIDGSLDLLYMRG

>tr|D6YU52|D6YU52_WADCW Uncharacterized protein OS=Waddlia chondrophila (strain ATCC VR-1470 / WSU 86-1044) OX=716544 GN=wcw_0288 PE=4 SV=1

MKKNLFVFLLLAAGWVSVYGDHYCVPRGPLKPCTWNASLRSGVEWMWYPDRRQNDYVSSI

PLIETTNVSSNTSTEFTLGEEVLTVLISPIALVSGDANLVVEDSNGNVLLDGTVVNGNFE

GEIVLEGEPFVLELSLDLTVPNALSVGELVSSVRTPRFSDQFSIPWTVVGEFGYALTCNT

EIFADLHYGGASGRSLGYSIGFSAAEGSEELWKITESYTDLQYFGGTIGMRYYFNSICCN

FRWKRPSLLKHLIAREEEMYRRKRGLITIPITSFTAVFNWDLA

>tr|D6YRX4|D6YRX4_WADCW Uncharacterized protein OS=Waddlia chondrophila (strain ATCC VR-1470 / WSU 86-1044) OX=716544 GN=wcw_1470 PE=4 SV=1

MSLISSSLSLSSKAMIEFPSRWELDLQSPSQRAFLAAHPAIFNRLDDTVLKQIPHHPWLS

VLVYALEFPETLDKKAWNMLYADLSLKETHCILCYIFEHPELIQGMDLGSADTLSIQPWG

GAFHNFHQLGMDLMKKEGEFEENLVMNFSKGMARKVKKVYETETDWRDLKSTSVDFTKGK

IIKVLGRTVLFQPNDSDKIFACKFLRTGESISEFCREKAMIEIFRECSRFKSRLPIPISI

SNPKSLPFEEFPVDVEKDQPALYVQQVEEHYYHYLHQIKDDSTWNEARKKFLFDSGLQLS

LGQLPPFVELFHNEESVRKYQPLANLIPNINRGAGRLETPFLKTQYPNAGAWGARDVGDG

PHLSEVVKNPTLESFDLFKLKKGKVHYLLMNGLAKLMLVDSLLLLHRLRLQERLDWKDPS

LVQTVAGWLKEGQIEALCGFTQKSLDMCRAFIDQLPIDWDRQAKQIMFWVQDDEKGYPQY

LDAGKLPEGLYDSKMSSVIDLKKAQNYSQGVGFLSGSDADIGCYNGPLGWIESEKSWYWI

SAFAVGVHICQSKDVYSINEKNLCESQKKLKLRRLSFSEEI

>tr|D6YT91|D6YT91_WADCW Uncharacterized protein OS=Waddlia chondrophila (strain ATCC VR-1470 / WSU 86-1044) OX=716544 GN=wcw_1953 PE=4 SV=1

MNLSRYCYLCKYFFFLFLMLTYPLFSSDVRNSNEIQIRNGQLYLNEEPFTVKPVIAP

>tr|D6YV19|D6YV19_WADCW Uncharacterized protein OS=Waddlia chondrophila (strain ATCC VR-1470 / WSU 86-1044) OX=716544 GN=wcw_0612 PE=4 SV=1

MATSTLIHSISYDFKVVGSLRENERLLTNGRKFSKLQAPDRFFKNSQNPLLKLLHKIVTL

ALKILQALERAIRGEQSEINLHNVEELTHELSTVLENDLLLEREGELYTVLKDLGHLSHS

LHQSVSGSLENYKTTLLSRSACVDASSMDDEAWQMEKFKKITDQVANEILPKLGRVIEGL

ESKVAEEDRKQSEKFFSQLFSKDPLDLDIRVSAKSYTEILKSKFGVSAVERALRDYELEY

ELALSGSDMAALAIGVVAHFKEEDLKEYIPLFDQESEEALCWQLIDLRSSMNPHVFSMRV

SKPYRCQLEHDQKFLQYVNDIEDWVAVDEHVKSKGLKHYSYAEYLSRHIIYALFSESHAC

FPDGLLVPMYDEANALRLMTVHQLVSVKGLHGALFKSFRPVSEQNKMHVVFRGTYCRYSI

LRDISPTETMQNRLFDGPGRYSFTKHQENIYDKILEHASSVPHPVFEFGGHSLGASDAMR

AMEYFMYRQAESVERFPLTKFVLNAFNTPGIEPDVSRRFMQSLKILNVETDLRYFDVHHD

IVQELGAVRLGYWRSDETRPDFLKISIFKFNRRVEERLVALARNFFKKMSFNLKQALEAH

TFYCLKLHDTENPKRLNDTFIQEILSNHPDDLGVSYGKEGDKKSELASDDQLSDSLLTTS

CRIGRKLKQMSKKVATLFHRLTTFRATASHI

>tr|D6YTF7|D6YTF7_WADCW Uncharacterized protein OS=Waddlia chondrophila (strain ATCC VR-1470 / WSU 86-1044) OX=716544 GN=wcw_0041 PE=4 SV=1

MSVYKTDGLTNFQKCHRMIFLCPEGSLSHWGVKKIEYCYRNERTKIHIIWAHVIRRTIAL

AMPIFNLLDALHYVPMAAYKLVQLKPKDALHDLLKCFKCLQVFLCAVPTLVVAMIEPKWF

YRTEGMWLDVKNKQMKREITHAFEKMKEDSQTGVETVVHLKVACEKVIDKMVGNDQAIEI

LKKVLEKVTSEIPTDDPEKQEIIIEQYIEMFSELLVCSAHQQVELNKMTEHFTRILELII

KVRHPLLRLSLIHLIVTTAKNDPDTYEDIAKKKYENDNRQALPYMVARLITDDEDLLARL

MEISNDRYFKNRQTQTSFIATLHEIHQSELKDSDKEKALGSLLDSFDLDQRIAEAGKPVQ

RKKGEPKNAEERDQMIENKKRNIKQKQGEIQNGNKRVKRLESQIQIQNVKNEIASLEKMR

FISDSEREELLKLKKDKGSHFHSAQNILRMLLSLNDKNFVSKYLRRIGSEKKYPHLSDER

IVTDVFKEIFELENLPPDAFEKIQRLRAPWALVAFHGRLNEVKAKYRSDLIQCNKAIVQA

LLDGTYDELRHETDANPTLKKVFEVQPGLKDKWMHPSKDDYTVKGLYSEATRRFANFKIV

EANDPSDILLIGNDTHTCVHLGGRIERVSGMSAFIRDGKIHTILIKDEEGVTVAETQLQL

MWDEVNKKPVLFIEEANFLGGENNDYSLEHAIYSYARERAKELGLNLVSCYHMKDPKGKL

ISSRKYEGKVGSLGSSSPLEYVNRYFKNYSKPYDLGQTWYVTT

>tr|D6YUK2|D6YUK2_WADCW Uncharacterized protein OS=Waddlia chondrophila (strain ATCC VR-1470 / WSU 86-1044) OX=716544 GN=wcw_0441 PE=4 SV=1

MSCLTNNCYPWRKEFPEISKVLNNKEVESVESLFSKKGIDRAAERLEGVLFLPDGESRTV

QLIHLARIGDDESLRPLTEKQERIFEVGQRTDKVAVNWLNGGCGLYAVTSLVSFTTCGPT

YALSWIPASISTLFNVVSTGVSYLSTGCYPHMASVEGNKMQNTFHEAKRMYTELGSHLIS

IYEIDSKKAEKLAEKIDIQSIINRLEDFFSDREAVSLTEPLAAARRFVLKKEIPDGPVEI

RNSARLVLLSEEVEHLQKQNKKLKGKHL

>tr|D6YWK9|D6YWK9_WADCW Putative methyltransferase OS=Waddlia chondrophila (strain ATCC VR-1470 / WSU 86-1044) OX=716544 GN=wcw_1163 PE=4 SV=1

MWFKKWFNEDYLKLYSHRTMGEASKQVDFIRRSAGCSGSKTVLDLACGTGRHSIAFGLKG

HQVTGIDLSEALIAKARKRALEYEELNVSFLVADLFSLPDIGRFDLVANLFTSFGYFSDD

QKNSQVFFVVRERLKDDGRFFLDFLHPYSVKQNLVEEESLLVDGEEVEVKRFIKDDCVYK

QIQFPGRAYEERVKLYDRDVIEQMLSNAGLSVVNVWNDYLGNSWKKEGDRQLFLCIKNSG

>tr|D6YSS4|D6YSS4_WADCW Uncharacterized protein OS=Waddlia chondrophila (strain ATCC VR-1470 / WSU 86-1044) OX=716544 GN=wcw_1780 PE=4 SV=1

MKKIFLFFTLVSFLLKVEATVNVSERLQNISAEDQKKICYFFEKLIKQYGFGYTLFGEKP

VSMLYWLAIPEYDRKRPYFSVDEDFVEAYKTWKKHQGKFSSEKYFFEERSLVVGKEYVDL

ILINKSEFYKKIFLHSDLFPDHYDEKAFINNEKVELFSKEDVGGYHLRMGVLLGYGEGNA

SEFAKRTINDPKESPDWVVFKDLIRTKKNKNTPNPPVFRANPHTQETQKLIENYSQTQEK

LEDILNNENFLQIVLEEYCSAAAD

>tr|D6YSJ4|D6YSJ4_WADCW Uncharacterized protein OS=Waddlia chondrophila (strain ATCC VR-1470 / WSU 86-1044) OX=716544 GN=wcw_1695 PE=4 SV=1

MSFTPSVSHSHPHTKVEGCYHQEKTKFDFLINVIEKISAIALGIFSAYVNLELFVPFFFA

GVCIGVYSYIQDRTLCNQGHLSSSCAHGLLEQLTGVKLPPVISLAANIAVTVCHIDHHAT

VFVPIIGVSIGAWIGKTVSYYGDLNYMTLPHN

>tr|D6YTA1|D6YTA1_WADCW Uncharacterized protein OS=Waddlia chondrophila (strain ATCC VR-1470 / WSU 86-1044) OX=716544 GN=wcw_1963 PE=4 SV=1

MKDGIGIYGSIFNYSFLFVLFGSTLFMFLYLWYKGLLGMGEGPKYDMMHMDEEEEKK

>tr|D6YU62|D6YU62_WADCW Putative cysteine desulfurase OS=Waddlia chondrophila (strain ATCC VR-1470 / WSU 86-1044) OX=716544 GN=nifS1 PE=4 SV=1

MIYLDNSMAARPSKKGISAMMPYFTDYWASPSSPHAMGQQTTGMIRDAYQSLYDFLGAGK

SDAVVFTSCGAESANHVFHSIYTDIALPRGKNHFITGKTSEAPALMAMHQLEELGCVGKC

VDVDRQGQVTKELLGDVLSPRAALVSLSWGDGLTGVIQPVEEIAALCEERGILLHLDATH

VLGKRYFTLEEVKADLISFNGSQIHAPQGTGALYIRHGLKISPFIAGGMEQAGLRGGDLN

VPGLAALSVAAKEALDARDLVCTETARLRDRLEENVASQLEDVTIFYQDQERLPHLSCMG

FPGVANEALLFLLNKKGVMACIGGGSFQQIGLVLMAAGVEETLAHSSMSFSLSRETTESE

IDRASEMIVEAVKQLRKSSMGINL

>tr|D6YWT3|D6YWT3_WADCW FAD dependent oxidoreductase OS=Waddlia chondrophila (strain ATCC VR-1470 / WSU 86-1044) OX=716544 GN=wcw_1237 PE=4 SV=1

MTKEKDHIAIVGAGFSGLACAFYLSEAGYPVTLYDPFPIGENASGISAGLLHYYTGPRAT

PPSDAEEKLKASLELFEASSDALGSSVFKKTGLFRPALNANQEKHYRKRAEVSEDIRWIS

ENETLELLPQFSPLPGIWIANGYSVDTKRYLEGLWQACRNKGATWKKQGVKTLSEFEEDL

VIAATGASPLLRDFGLSIHPVKGQILEIEWDIQLPFPISANVYLVPGTLPTRCFVGGTFE

HHFDDSKPDPSTAQELLLPKMKALFPQFNGYKIVDCRAALRGSTPNRLPICGNIKGNMWA

LAGMGSKGLLHHAFYAKKLINEILCYNIP

>tr|D6YVC0|D6YVC0_WADCW Putative membrane protein OS=Waddlia chondrophila (strain ATCC VR-1470 / WSU 86-1044) OX=716544 GN=wcw_0714 PE=4 SV=1

MEHKDHQEIEKPERIYVETEDYDPGHEFGGFQKEFKSSYQRIGARSYPVSVRIACLFLTL

FLLFFLLMATPFVLLFFGVNVLTFFQWHKLWERTKIVWSTYSKMIVTAFGLLVGIFSPAL

GLSIIVVYLMMQGQKSSNTWVERIFKASKKRQGR

>tr|D6YWD9|D6YWD9_WADCW Mutator family transposase OS=Waddlia chondrophila (strain ATCC VR-1470 / WSU 86-1044) OX=716544 GN=wcw_1093 PE=3 SV=1

MFLKTYRDKYPKACACLEKDKAQLFTFYNFPAIHWQHVRTTNPIESTFATIRHRTRQTKG

CGSVAATKNF

>tr|D6YTD3|D6YTD3_WADCW ATP-grasp domain-containing protein OS=Waddlia chondrophila (strain ATCC VR-1470 / WSU 86-1044) OX=716544 GN=ddlA1 PE=4 SV=1

MNIKTAKCLGPVADLEKHLPVDWWRSIFNSIYLKTDADVVENDTATAHEIDIFLNFSKIE

PSDHILDLCCGQGRHSLELAKRGYRFITGIDRSRYLIRLARSRSQKLGYPIKFSEGDARK

IKLADQSMDCVMMMGNSFGYFEQERDDLKVLKEANRVLCEGGLLYLDVTNGAWMKENYEK

RSWEWISEELMVCRERHLATDLCRLISRELVTHAEKGVIADQFYAERLYSFEELKSLLLA

AGFENIEQQDSLKGESFRNQDLGMMANRILITAIAPKKRKPATRKNPFKIPCTVLLGDPR

IPDAIKRDGQFNLEDFHTINQMKEALKKIENFQFSCFDDHKNLIRKFSQNPPPFVMNLCD

EGWNNDPFMELHPTALMEMLKIPYTGAGPECLSICYNKSTVRAIAQSMDVHVPSEIWIDP

SNHSAAIPSIFPALIKPAYGDSSIGITQNAVVYSAEELVTYFDNLKAQMPNIPILIQEFL

DGREFSVGVIGNGVSLEVLPILEVDYSGLPENLPKILGYESKWHPESPYCTHIRYHQAHL

DESLARDLIDTSINLFQRLKCRDYARFDFRMDSQGEAKLLEANPNPGWCWDGKMALMAEF

AGIQYHELLEKILKAAAERYPYLEHAETDSFSNKAAQKLKKS

>tr|D6YTS1|D6YTS1_WADCW Uncharacterized protein OS=Waddlia chondrophila (strain ATCC VR-1470 / WSU 86-1044) OX=716544 GN=wcw_0157 PE=3 SV=1

MMMDAILSNKYAAALLPMAAGCCFIFVCSLVKVPFYPVPMTMHTFAIFYLGLMQSPRNAC

GSALLYLAAGTLNPSWMIGKCGGYFLSFPIAAYLISWSVQKISPYLAILAGQGVIYSLGF

LWLVPFVGIKIAFLKGVLFFLPSAVVKAALAVKLAEARS

>tr|D6YW37|D6YW37_WADCW Thioredoxin reductase OS=Waddlia chondrophila (strain ATCC VR-1470 / WSU 86-1044) OX=716544 GN=trxB PE=3 SV=1

MEIKMKKTKVAIIGSGPAGYTAAIYASRANLEPVLYEGFQTGPAGGQLMITTDVENFPGF

PEGVMGPELMMTMRKQAERFGTEVITDDVVSVDFSEYPYKIKGNKHEHEAFAVIIATGAS

AKRLNIPGTREGEFWQKGVTACAVCDGAMPIFRNQKLYVIGGGDSACEEAIFLTKFGSEV

FIVHRRDELRASKIMQERAMNHPKITILWDSVLTKVEGDHVVKKVTIQNVKTKEENVHDA

GGVFFAIGHLPNTSFLNEQIELHDNGYIKVSPGTTRTSKAHVYACGDVQDFTYRQAITAA

GTGCMAALEAERELAEKGHLD

>tr|D6YT73|D6YT73_WADCW 30S ribosomal protein S2 OS=Waddlia chondrophila (strain ATCC VR-1470 / WSU 86-1044) OX=716544 GN=rpsB PE=3 SV=1

MIERQDAVVNQEGQGLAEITIKNLLEAGAHFGHQTSRWNPKMKRFIFEERNGLYIIDLAK

TLQQVRNAAEIVKECVKKHKSILFVGTKKQAKGVVKELAEECGEFYVCERWLGGMLTNLS

TIRQSIKKLDRIEKQIAAGGEGLTKKELSLLTKEQLKLDKNLSGVRSMRKLPGLVVVVDP

SKEHIAVAEAKKLGIPVMGLVDTNCDPDPIDYVIACNDDALKSIKLIINTLTDVIAKEKN

EMRLVSRKEGSEEEDKKIGEEIAVGKVDLAKSGKEDAELKKTAGAAKEEN

>tr|D6YVR4|D6YVR4_WADCW Transcription termination/antitermination protein NusA OS=Waddlia chondrophila (strain ATCC VR-1470 / WSU 86-1044) OX=716544 GN=nusA PE=3 SV=1

MNKDLVAIFEYLEKEKGIQREIVIHAIEESLQAAARKSVSGASNVTVQIDPKTGNINVFS

EKEIVDEVEVPAQEILLEEAREIDPDCEIGQFIDILATPKDFGRIAAQKARQIISQKLRS

AERDVIYEEYRHRVNEVVSGTVKRFVKGANLVVDLGKVEALMPMRQYPKTEKYKIGDRVL

ALLMEVNDTDSGGAEVILSRSSPEFVRQLMIQEVPEIVEGIVVIDKIEREAGYRTKLTVR

ATDQKVDPVGACVGMRGNRVKNVVRELNSEKIDIIPYSDDPIELLQNALSPIEIRKISIS

EDDRVISIVIDDDDYAAVIGKKGMNARLNSRLIGYELEVQRMTDYNKTMAIQRTELAESD

DPTLDHPLTGIEGVNKLVFEHLVAEGFNTPRTLLLASPEQLSNAASISLEMADHILEQVR

KQRL

>tr|D6YWD5|D6YWD5_WADCW Uncharacterized protein OS=Waddlia chondrophila (strain ATCC VR-1470 / WSU 86-1044) OX=716544 GN=wcw_1089 PE=4 SV=1

MIKISNADKQARYRKKEHLKRLANNFFRDWQLKPWEGNSSSPKDVQRLLDKAIELPSGWT

DKDYEKSVQALEALKAELWCASNKLKNDVDAGWSSLDFMNSSDPRKFIRDNKEAIERARN

LASHLISALELSNCNNTDQAAALMEVVRYVGRSLASSNDVRRSQATAICLVSIGSQYKRP

DWFAEELANIIKCHVDSDVAHQVGILLITSQA

>tr|D6YU73|D6YU73_WADCW Putative 1-acyl-sn-glycerol-3-phosphate acyltransferase OS=Waddlia chondrophila (strain ATCC VR-1470 / WSU 86-1044) OX=716544 GN=plsC PE=4 SV=1

MSLLYKTTHTFLRIFFRIFYRHRILNADKFVQGPAIIAPNHISFLDPPLVGASCPEPVAF

LARETLFSKPFLGTLIRKLNAYPVSGKIKDLSSMKVVLNILKDNKKVMIFPEGIRSHDGE

LAEIKPGICMLAMRAQVPIIPVYIQGTFEIWNRQRKFPKLWGKTCCIFGEPIYPDTFKEM

DKKSAQEAMALEIKKRILLLKKSL

>tr|D6YV58|D6YV58_WADCW Uncharacterized protein OS=Waddlia chondrophila (strain ATCC VR-1470 / WSU 86-1044) OX=716544 GN=wcw_0651 PE=4 SV=1

MRFIFNFIFFGLLFFIIYKFLPETFETLVGWVDKLYDVLRDAVVWVFEKVQSVASNSGNA

A

>tr|D6YVN0|D6YVN0_WADCW Uncharacterized protein OS=Waddlia chondrophila (strain ATCC VR-1470 / WSU 86-1044) OX=716544 GN=wcw_0825 PE=4 SV=1

MENELQFKWERQPAAEKCLSDLLTLYREKNGKIRQFEEDLQKHTSTRLFDWIDYFAVVDQ

SAVKCFEEVGFELQAEGVYYHPGAQLPRIAVMQKEGVAVKVEQCADFLMVHGISSNIEGI

PYGRFRRALVEKENDCFLWVVERRCYPEVEPEEAEKNGTEKYLKALEIWKTRERAADDEE

EGMQNAFDAVKQMIALIGRDLAAWAVMEGERAYWQARNHAGQVQKNRQDRLGMGWANHDH

HTFRSSRKFFPQLIRLFELLGFHCRERFYAGEEAGWGAQVMENPGAGLVLFCDLDLSPEE

LHIDFAHEKLSELPELGTIGLWCALHGDSILKAGMHHLEAQFSFEELTNDLHQQGVGMMD

PFSNFSYLKQAFTHGEQWVIDPKRLDKLLVEGKISSEEKEKFLKFGAVGSHMENLQRREG

YKGFNQKNVSYIIKETDPRVR

>tr|D6YVQ9|D6YVQ9_WADCW Uncharacterized protein OS=Waddlia chondrophila (strain ATCC VR-1470 / WSU 86-1044) OX=716544 GN=wcw_0854 PE=4 SV=1

MKLPSLLPEDLAYAGAAKSARELYELLQKDYADYSRFFVSASDDETWCTQHSAFMRLSLQ

WFTVQFFKDKLQVDLAKKVGHAIRTHSRLMDEWLPRNLSIRMNHESLPINSLLWGTSSEW

LRQKIRSDCRDQQSTTLEFEGLTVRLFDLMNAYMSTGGIRDLWSKTEEEIIGVLELATRW

HLKDLSEYAQKGFVKYLDEFNVVNWLIRSQQSQWECLRSAAIEYVNKRELGVRIEDRSID

EFSFEFLDFGKKALEFFETVRHMVTKLVCGHHLIEEVMFSEVVNRCPSLNVLSVSESENF

SDRLLDLPHDLKGLDLSKCAWLTHQKLRQILEGNPSISTLSLSANIQLNYTAWGLLKLLK

GLLKLDLSSCHQINDHDLKVILQAGKELTHLNLARCVNLSDLSFFEIPKITKQLMELNLS

ECRIYDAGLIDILSKCRKMTHLIVRKCSTLSDRGVLEGIRNAPNLQTIDLSGCGFSNASI

RIIQEARPYLFLNV

>tr|D6YRU7|D6YRU7_WADCW Heptose adenosyltransferase OS=Waddlia chondrophila (strain ATCC VR-1470 / WSU 86-1044) OX=716544 GN=hldC PE=4 SV=1

MPLWVDDYQKKLLRPEELEDAVEGIKSRNRTIATLNGSFDLLHAGHLHIIYSASQTADCL

IVALNTDASIQRYKSYDRPIIPLPFRLQMMAALEFVDFVTYFDETDPIKILSIIKPDIHV

NGAEYGENCIEAETVKAYGGRVETVELVDGLSTSNIINKIASLKTHASSPHL

>tr|D6YT85|D6YT85_WADCW Uncharacterized protein OS=Waddlia chondrophila (strain ATCC VR-1470 / WSU 86-1044) OX=716544 GN=wcw_1947 PE=4 SV=1

MLLALRRRLQLSGLIIALYFASILIISIYPDASLLKFVFGLFIASVGSILAFLLLRQWEK

SKLAEQLPLPSSPEPMQNAPSFSSPENIGELEQALKDAQSKNHDLIQKQNQLEETLHKHE

QEKAQFELKLQDIQHQIEAQSLEADEEIRRKTTLLSEYQETINQQREVIKKKQEQISELE

SKVRDLNYEVKTLLQLAEIGNSKKESAYSEEKENEQPSSSYVYGGSMVRSPEEASVLLGR

CINIAQKITTSNRFNSNSRFHHMPLDSSALDFRRLYDSLRSETSSGIIVYSLKENRLIFA

NDQIEMIMGWNSETFTRNFSEIIREGYEEWNAALGQLSPHTESKARMLFKTKNGQNILIN

CHLKIIPTGIFRHYVIGTLYPA

>tr|D6YWA5|D6YWA5_WADCW Imidazolonepropionase OS=Waddlia chondrophila (strain ATCC VR-1470 / WSU 86-1044) OX=716544 GN=hutI PE=4 SV=1

MKLIGPFAQLVTMRNVRNAGPVEDVSLEILPHVGILIEDGKIAKIDNYENLRADDQITFD

SPVIAIPGLIDAHTHLCWGGSRTRDYALRVRGVSYQEIAKQGGGILDTVAQTREASLEEL

TNVVDQHLDQQLSWGVATTEVKSGYGLSVESELKMLKAIQIAARKHPVDVISTCLAAHTL

PPEFNDRKQYLNHLQSALFPRLKPGTHRIDIFIEEGAFSPEEALPYLQYAKKLGFEITVH

ANQFTHAGVNVACQVGAVSADHLEHLTKDEICLLKQSGIIAVALPGASLGLGERMAPVRK

MLDAGLSVAIASDWNPGSAPMGNLLAQAALLGAFEKLSLAETLAGITKRAAAALKLPDRG

VLEVGKRCDLTVFKASDWREIFYYQGSLQPSDTVIQGKRFHHVDRSR

>tr|D6YTI1|D6YTI1_WADCW Glycosyl transferase, group 2 family protein OS=Waddlia chondrophila (strain ATCC VR-1470 / WSU 86-1044) OX=716544 GN=rfbN PE=4 SV=1

MDEIGIAVITHQAKHHLKYCLPPLLKSPMKPKILVVNSSSHDGTVEEAETLGAETLVVPR

NSFNHGKTREAARKHLATDIVIMITPDAYAEDSSLVETLTQPLRDGTASIAYAKQIPHQG

ADLFASFSREYNYPEKSQLRGIEDLDTYGVYTFFCSNSCCAWKNSALDSIGGFSHVLLGE

DTVAAAKLLRKGHKIAYISEAKVRHSHSYSLWQEFQRHYDTGLARRQYSQLFSGAGGDNK

RGKAYALALLKKVYQTKKQLLPYAVLQTAAKLAGYYIGKLTLNAPITIKRTLSSQDFYWD

>tr|D6YRM0|D6YRM0_WADCW Putative rhs family protein OS=Waddlia chondrophila (strain ATCC VR-1470 / WSU 86-1044) OX=716544 GN=rhs29 PE=4 SV=1

MITEVVIAKNSKERNIKLVCDEGKTVEYHRSDFFRLDRVETNFSPPVSYQYDHEGDPWSP

LLISKSFPDHRYLNISYRGEKKKPKKVSALEAPIGTDEQPKRLYTFVYDDRKTYVTDARG

NRSTYRWNEEDRLTTIRHYCSDNTTLYRSEELRWGCHRLWDPTHFRSHYIVDAQGYVWAG

KQFYNDNNQKCVTKEEFYGNLSGQGKLYRSNGQGEKYIKTYTYYPNNLIESETFQGEGED

FKQRITYTYYGDSSLVKTKMVSDPGGIKERYYYAYDPCGEVAFEIVDDGSSSSINDLAGV

TRRTFKRIKLSSKGVPIEEAYSYLDLQTGAEKQLSRVVNHYSSRRLIVKKDYYDTENTYL

YSESWKYNDYELITEEIDRAGRVTTCSYDANGNCIRKSFPHFEYQILYTYDFSNRLIKEE

ELHDDGRSFVKSYAYDLKGNKIKEVDPCGHETLYTYDPYDRLIAKTLPPVEDGSGKLSTG

VERYTYNVLNHLSSREDPEGKITRILTTAYGKPYRIEYPDKTVEHVLYNKDGSLKMTTDA

QGTKTQFAYDYKSRKIKEEKISREGELLSIREWKYSAFDLLEEIDPEGHATTYTYNGAGQ

LTAKRKGDAETLIFYDTLGREKEKWEKCSDHTYRITVLEYNLLDQVVEEKIIDESGNLFL

LKQYAYDAWGNRTREIIGGHCPLSTDYNSSSQPVRTVTAEGFETLFHYDNNYLNTFGQTV

AAVTEIDPRGNVTFTVFDARGRPAVQERKNTYAETVQKTEYLYNQIGKPLLQKESVIVSG

EILRIHTTAFEYDSRDREMTVIEAKGEPEQKITRRTYTAFGEVEEIIKPSQVIVNHAYDP

LGRLSSLKTSDGAISYAYTYDRNNNILEVEDLISQSRTTRIYDIFGNIVSETLAHGSTLE

YAYDPLSRLTEVTLPDSSLISYSYDAGYLREIRRGRYAHKDNYGSHGKRLSSQMVQRLGE

IHYAYDQSLRPSLIESQYLKMEMAYDAIGNVETVDYTDDLGSESYSYQYDAMNQLISDNS

HQYTFDSLGNRVACDGVDNTHNALNQLLKQGKNTYRYDADGNLIAKNEESYVYDAFGRLT

EALKENLRTTYTYDPFHRRLSKTRFTLQEEQWVEISSEKYLYQGDKEIGVIGEHGELESL

RVMGVGVKGDVGAAVLMEFLGRAYLPLHDYRGNVSMLVDAVTAEVVESYRFDAFGVETVS

SQSKVPLNPWRFSSKRVDAETGWVYFGARYYDAEMGRWTTPDPMWFVNGTNLYCYVRNNP

TMYIDSEGLFFNEIVKFLKDTFRAIFKALVENVDVSADERGNIQVNFNTHPSLDHQSKST

SAKNDVYGSVHVMTVNGIFNSYEESEDLCGRMSELAGGCTSSFVYNASHGVFDLIESFLN

LLGIVTRPAQLLHKEWDHFFANCPEDGIIIMVCHSQGAILTRNALSTYDEELRKRIHVVA

VAPAAYIDKKFCGSVMHYVSKRDFVPWIDVAGRIRNRDTTVELDPHPDAPFFDHAVSSPT

YQDDIDKRIQWIRKHHGDYR

>tr|D6YSE6|D6YSE6_WADCW Dephospho-CoA kinase OS=Waddlia chondrophila (strain ATCC VR-1470 / WSU 86-1044) OX=716544 GN=coaE PE=3 SV=1

MLRLKKVAVTGGISSGKSLICQYFSEFGAYVIDADKIVHQLLNPDTEIGQKVVALLGERI

LDKQTISRSRVAKLVFLNPRLLKSLENLLHPLVYEEINRIYKKVAHEKNPPPLFVAEVPL

LFESGGEAYFDQTIAVVSIQEKCWERYRASTGNEREDFNRRTACQLPQHVKAEKADIVIH

NEGSIESLKKQTKTIYESLRQCT

>tr|D6YUT7|D6YUT7_WADCW YhcG_C domain-containing protein OS=Waddlia chondrophila (strain ATCC VR-1470 / WSU 86-1044) OX=716544 GN=wcw_0527 PE=4 SV=1

MVIQEGWSQNALLDAIKIKTYKRHGKAITNFHVRLPDPQSQLAHETLKDPYNFDFLELTR

EHVEKDLEDGLIEHVEKFICELGQGFSFVGRQVPLKVGNKDFYIDLLFYHLKLRCFVVVE

LKATDFKPEFAGKMNFYLSAVDDLMRYPADSPTIGILICRRKDNFIVEYALRDINKPMGV

AEYETKIISSLPKKLKGKLPSIDEIEAELSSLPVVQKKRSSKKTPK

>tr|D6YTY2|D6YTY2_WADCW Uncharacterized protein OS=Waddlia chondrophila (strain ATCC VR-1470 / WSU 86-1044) OX=716544 GN=wcw_0218 PE=4 SV=1

MGFSSDGKEGGKDNSEDTGFLPEVELELSNLDKKEIFEF

>tr|D6YVU8|D6YVU8_WADCW Uncharacterized protein OS=Waddlia chondrophila (strain ATCC VR-1470 / WSU 86-1044) OX=716544 GN=wcw_0897 PE=3 SV=1

MTFENLSWIFVILSLAGNVFVIKKNVIGQWLWAISNIGWVVYDLSIGNYSQALLFAVYVC

TSIWGIIAWTKDDQFLNKGLS

>tr|D6YU58|D6YU58_WADCW Permease, major facilitator superfamily OS=Waddlia chondrophila (strain ATCC VR-1470 / WSU 86-1044) OX=716544 GN=wcw_0294 PE=4 SV=1

MKTDLHHQRTRAAFMWSRILRTPFWAIYTMLPFILFRDLNASPFQIAVTVALKPMVSIFS

MYWSAAVRQRRDLLVSNIIWAGVLGLLPFFFFPFVDSPWFFIASFGFFMLLHRGVIPAWM

EILKLNIPNTSREKVFAWGSALGYLGDGILPFLFGALLDGYFQAWRWIFPLTALIGLIPI

LFQARILVRFEVAEQPKRIPFKQSLVQPWVSAWMLIKERSDFRSFQVGFMLGGAGLMIMH

AVLPAFFMGVLQLSYKELAIALTLCKGIGFAATSQFWAKWMSKVDIYRFSSVVTLMAFLF

PFCLLAAQMHLYWIYTAYIVYGVMQAGSELSWNLSGPIFSGDEDSSLYSSVNVVTVGLRG

CIAPGLGSYLVTLMSSASVLLIGGGFCLLAFIFMASYSKTQRQAKVYSGIV

>tr|D6YTZ9|D6YTZ9_WADCW Uncharacterized protein OS=Waddlia chondrophila (strain ATCC VR-1470 / WSU 86-1044) OX=716544 GN=wcw_0235 PE=4 SV=1

MAKRYRWRRNLSKDDVQLSDVYQAILAIHL

>tr|D6YT69|D6YT69_WADCW Uncharacterized protein OS=Waddlia chondrophila (strain ATCC VR-1470 / WSU 86-1044) OX=716544 GN=wcw_1928 PE=4 SV=1

MSPASLTLQIITNFFQGELLGRQGKLVSVGESENDLLREKEAARCMFS

>tr|D6YSW3|D6YSW3_WADCW Uncharacterized protein OS=Waddlia chondrophila (strain ATCC VR-1470 / WSU 86-1044) OX=716544 GN=wcw_1819 PE=4 SV=1

MNLQSDYSDYLSGAKFHNGLNVQISNRHELKDRLCKIEELVQNKNVLHAGCVDHLPLIKE

KIHSNRWLHKRLSLCASRCMGFDIDQPGIEFVKKLGYSNVIYHDLIKDKILPKEICEFE

>tr|D6YSB1|D6YSB1_WADCW Putative type III secretion system protein SctD OS=Waddlia chondrophila (strain ATCC VR-1470 / WSU 86-1044) OX=716544 GN=sctD PE=4 SV=1

MGAKLVVKDGESKGLTLPLEGRKEWVIGSDPEACQLLLEDPMTAQKHLICRTTPEGIELE

NLSEDNPVSVNEQIVNHPLLLKNGDSVKIGQNFFTFYAGEENGEEEKPSIVDEDAPEQDT

IYEEESDEDKGILAEIDFDLRESGRWLLKVIGGPNNGAEFSMQTGSSYMIGTDPTSCDVV

FHDTSVSRQHAKITVSKNDALSIEDLKSRNGTLVDGNPLEGQQTFEPNTLVTLGTTSFVV

YDREGEMQTIISPLLPSIVKVLQEEEETEEKTQAKESERKKLEEELATTKESKAQNALGA

FILIGIIAGLFAVVGIGTVSLFQSSPVQIREKIDYSAALAQAMQKFPQVRYSYNENSGRL

LLIGHVMTQSDKNQLMYNLQGLEFLKGVDDSGLIIDEYVWRETNQILARGNRFRGISVHS

PQAGKFVLSGYLKTRKQAEELSDYITANFPYLDILERRVIVEEDVLTTVETDLNNAGLRN

FNVQINNGELTISGGASQEQLAKVDRLVRKFREIPGVRNVKTYINEIAPEQTMVNISDKY

TVSGFSNQGGVNLNVVINGRILTRGDVLDGMTITSIRPDAIFLEKGGVKYRIDYNN

>tr|D6YWV1|D6YWV1_WADCW Histone deacetylase family protein OS=Waddlia chondrophila (strain ATCC VR-1470 / WSU 86-1044) OX=716544 GN=wcw_1260 PE=3 SV=1

MFNVGLVTDPIYMQHLTGAGHPESPARIASIAEAVRDLPLLAIDAREAREADLLRCHTKG

YIQTLIKDVAKCMQSRVHDGRYQLSTGDTQISPLSLLAAVKAAGAVIAGVDAVIREDVKR

VFCLVRPPGHHAESDQGMGFCLFNNAAVGARYAQTYKEIEKVAIIDWDVHHGNGTQAIFY

DDPTVHYSSTHQFPHYPGTGSREETGIGRGIGTTLNCPINAGKRSREEVLRAFRTEIRPA

LDKFKPDLIIISAGFDLLEEDPLGGFNATVEDIVELTHIVNKIADVHAEGRIISVLEGGY

DIKAIAAAAKAHIHALMS

>tr|D6YS92|D6YS92_WADCW Ribonucleoside-diphosphate reductase OS=Waddlia chondrophila (strain ATCC VR-1470 / WSU 86-1044) OX=716544 GN=nrdA PE=3 SV=1

MTINTLEQKISVRKRDKQITPFNPERIKNAISSAFRDVWNLQSYHTLDKAQQVIIGQVFE

AVIEKIRCEKPFDTPIDVERIQDYVELILMRQEHYKVAKSYILYRENRAKARQQKEEEAP

AIRVKLINGDSVAYSQEKLKLSLKRSCQDFEPLCSSEELMKAIEPQLFDRISSEQIRQAA

ILAAKSLIEKEPAYDKVAANILLQKIYREIESLPFSNFSSYIQKGVEAKRLHSDLLGFDH

RLLEEAMEHERDRQFSYQGLQTLYDRYLIRISEQVAELPQFFWMRVSMGLALNETDKNSK

AVEFYHVLSSFDYISSTPTLFNSGTCHPQLSSCYLSTVQDDLNDIFRVISDDAKLSKWAG

GIGNDWTNVRATQAHINGTNGKSQGVIPFLKVVNDTAVAVNQGGKRKGAICCYLETWHLD

LPEFLDLRKNTGDDRRRTHDMHTANWIPDLFMKRVEEDGEWTLFSPDEVPDLHDLYGRAF

DKRYEHYEREAKAGKIKQYRTFKAIDLWRKMLSRLFETGHPWITWKDPSNIRGNQKHAGV

IHSSNLCTEILLPTSKEETAVCNLGSVNLRNHIKEGKLDVSHLEKTIATAIRMLDNVIDI

NFYPTPEARESNLRHRPIGLGMMGFQDALYLMEIPYCSQEAIAFADSSMETISYFAIKAS

SLLAKERGSYPSFKGSNWDLGLFPHQTMALLEEERGIKIPIDQCSSRDWKTLLEIIRQNG

MRNSHVLAIAPTATISQIVGVSQSIEPQYTNLFTKSNLSGEFTSVNHYLVETLKSLNLWD

QEMLDELKYHDGSVQAIPRIPQEVRERFRTAFEVGAEWLIRCAAARQKWIDMGQSLNLYL

TEPSGRKLDEMYRLAWKLGLKTTYYLRTRAATQVEKSTLDINKFDIQPKWMKNKSASSSI

APRQGQCSIDDPTCEACQ

>tr|D6YS93|D6YS93_WADCW Ribonucleoside-diphosphate reductase subunit beta OS=Waddlia chondrophila (strain ATCC VR-1470 / WSU 86-1044) OX=716544 GN=nrdB PE=3 SV=1

MQQFHTKNTGRFRSDQKKLINCSEVDVNQLLPLKYKWAWEHYLNGCANHWMPSEVPMTKD

IETWKSDSISEDERLLIKRNLGFFATAESLVANNLVLAIFKHVTNAECRQYLLRQAFEEA

LHSHAFLYIVDSLGLEEQEIFNMYREISTISEKDEFEMQLTKGLIDGETKTLAGKQQFLK

NLVGYYIIMEGIFFYSGFAMVLSLHRRNIMPGIGEQFQYILRDETIHLNFGIDLINGIKA

ENPEIWNEDLTDEINGLVQQAVELEIAYAKECLPHGVLGLNAGLFVEYVQYVADRRLERI

GLSAIYKSSNPFPWMSETMDLTKEKNFFETRVTEYQSAAVLSWD

>tr|D6YV29|D6YV29_WADCW Uncharacterized protein OS=Waddlia chondrophila (strain ATCC VR-1470 / WSU 86-1044) OX=716544 GN=wcw_0622 PE=4 SV=1

MKDVVSNGGKARNVFPENPKNLLSDLPRDAKGHIYASDNIRIRPEQHLLRGCM

>tr|D6YRQ6|D6YRQ6_WADCW Uncharacterized protein OS=Waddlia chondrophila (strain ATCC VR-1470 / WSU 86-1044) OX=716544 GN=wcw_1401 PE=4 SV=1

MKDAKNHLKHLQKKVIQSARKEETKKNTGLLSKEERVVEFKRPKTVLNKRKIA

>tr|D6YTF1|D6YTF1_WADCW Uncharacterized protein OS=Waddlia chondrophila (strain ATCC VR-1470 / WSU 86-1044) OX=716544 GN=wcw_0035 PE=4 SV=1

MSKEMIKKLLQLLLFFLLSFNLEGASPPASFTFMIADLKHNDEEGIKICELQAGSLSAFR

GYDWLTNEPRVVSKKVMKILNQFGHSLWVFSGSIADPSIQKEMAEQKVNIVKNLSKLCQN

KTFIQQANLPVEDPYDLSSYPVLLIGSPKRFHNNLESFQEQFPNVLILDLPSYPYWKNKH

EMSLLLREPPELEQIKPHWELFSRAMTSEEIQSSHTHFSSEYVVIKPLNAFTGSGVILLE

RTKLPEILQLIWKSPKKLPDSSDKGYSYWRKAKDKHLIVEEFHFSDPVAVSHLEGREYDP

TLRAIFMLWHEHGEIQMDFVGLYWKLPEKSLSEEGSFNEKHKSCGKIPYFALVEPEKEEA

IKHQLKETMPLLYAKMLGWEKYEPIRIKNLELH

>tr|D6YT42|D6YT42_WADCW Transposase OS=Waddlia chondrophila (strain ATCC VR-1470 / WSU 86-1044) OX=716544 GN=wcw_1900 PE=4 SV=1

MITQEYFMEIQILRKQGKGIRQIANELCLSRNTVRKYLRSKKPPKYHSNQKRPSKLDPFK

AFLEKRIKNAGRHFIPATVLYREIKELGYQGGITILRDWVRKGKKQVELYCSPLRGCM

>tr|D6YTV5|D6YTV5_WADCW Tryptophan--tRNA ligase OS=Waddlia chondrophila (strain ATCC VR-1470 / WSU 86-1044) OX=716544 GN=trpS PE=3 SV=1

MAEKKRILTGDRPTGKLHLGHYVGSLKNRVALQDKYDCYFIIADLHTLTTKPEKEHIMEM

RGNIREMVLDYLACGIDPSKSTIYLQSATSAVYQMNLYFEMMISFNRLTGLPSLKEMARN

AHIDAESIPFGLIGYPVLQTADILMPRAHLVPVGKDNEAHIELSRGIARKFNQYYGEVFP

LPEVLLSDVPTLIGTDGKGKMSKSAGNAIFLSDDPKSVEKKVKGMFTDPNRISADVPGTV

EGNPVFIYHEIFNPDKEEVEALKTRYRAGTVGDVEVKQKLAAALNVFLDPIREKREQFAQ

DKGFVEQVIYEGTQKMIEVSNQTVKEMTSAMGMSGAWKKISKMAKERK

>tr|D6YVA8|D6YVA8_WADCW Acylglycerophosphoethanolamine acyltransferase / acyl-ACP synthetase OS=Waddlia chondrophila (strain ATCC VR-1470 / WSU 86-1044) OX=716544 GN=aas1 PE=4 SV=1

MKKLFLFAIALIFRLFLRLRYRIRVKGLETLTPEALNRKGGVLFLPNHPAALADPAIVAL

SVFPKFPIRPLIVEYMYFTPGVHAAMKYLNAIPVPNFESSNNSLKRKRHDMVLNEVIKGL

KEGQHFLLYPAGRLKHTQIEKIGGSSATQKIISETPEANVVLVRIKGLWGSSLSRAQTGE

VPDLLSTLKRHIWTVLKNLIFFVPKREVLVELEPAPKNFPYEASRLEMNRWLENYYNRPD

GLTEQKGEYPGESLVLTSYSFWKKELPAICDPVAETDHHIVHIEGIDYKIQDKVLQKLED

LTEIKKEKISPSMSLSSDLGLDSLDTSELAMFINDSFDTGPIPVTELTHVNKVMAIAAKQ

VKIKEKEEEAQADMNKWAFKGERKTAATFEGKTMIEVFLNASSKLKNQPIYADARSGILD

YSRMRLAVCLLAEQIRHMPGKYIGILLPASVGSFLCTFAIQLAGKIPIMINWTIGPRHIR

SIQELTDVQSVLTSWAFLERLDEVDLTGIDDKLVMLETMRRELSIGDKLRAFTRSKRSNQ

AIMKIFGVDKLTKDDIAVILFTSGSESMPKGVPLSHDNILSNQRSALTVVEMYTDDVIFS

FLPPFHTFGFGVTGTIGLLAGIRTAFYPDPTDGKGLAKNFETWNCTVSVGAPSFIKALFK

AATPKQLEKMRLCITGAEKTPQELIDLVAQFGKEKCLVEGYGITECSPIIAGNLYGRPLQ

GVGKPIPICDVIVVHPETYEKLPQGQEGLFLVRGPNVFKGYINKDIPSPFIEIDGKQWYN

TGDIGYLDDDGFMHISGRKKRFIKVGGEMVSLLAIETALVSSAEKKKWLIKDEGPALAII

AKENPGARPEVILISTFDTDVDEANQTLRVCGFSNLIRISEVVKVKEIPIMGTGKTNYRV

LEEKYLPPQLRG

>tr|D6YVH3|D6YVH3_WADCW Uncharacterized protein OS=Waddlia chondrophila (strain ATCC VR-1470 / WSU 86-1044) OX=716544 GN=wcw_0767 PE=4 SV=1

MCRQYSNLEKGSKIFSFLYFNFSNNKRYIDWLKIS

>tr|D6YWD8|D6YWD8_WADCW Transposase OS=Waddlia chondrophila (strain ATCC VR-1470 / WSU 86-1044) OX=716544 GN=wcw_1092 PE=3 SV=1

MKNDVISLDKFHATSEMNSLLEQTLREGARLLKQQAIENEECSEGYSSAILPKYLRRVPS

LDAVIPALYLRGISTSNFQDALEAIMGKDAKGLSAANNITSSSECEKGHSNLYGPYQRRL

TGGV

>tr|D6YSW8|D6YSW8_WADCW Glucose--fructose oxidoreductase OS=Waddlia chondrophila (strain ATCC VR-1470 / WSU 86-1044) OX=716544 GN=wblC PE=4 SV=1

MNGLRNIAVVGGGRWGRNLIRNFFEIGALHTICDMNEALLDSYLEKYPNINTTTNFNSIL

ENPLLSRIVIAAPAIQHYALAKKALLAGKDVYVEKPLCLDCGEARELIDLAQKSGRILMI

GHLLQYHPYVLKLQELVSTGELGRLQYIVSNRLNLGAVRTEENSLWNFAPHDVSVILSLC

GNQMPESVRCLGGDFVSQGVADKALMTLRFNGGIRSHIYVSWLNPYKEQKLILTGSHGMA

VFDDTKPWEKKLVFYRTQIKWNNGIIPQIESESSEPIIVPPAEPLKEECRHFIKCCDERI

RPKTDGEEALRVLKVLQAAQKSMDAEGTEKKPEDFQKAYHAHSTAEIDPKSSIGKGTKIW

HFSHLMADSIVGEGCNIGQNVVISPNVRLGRNVKVQNNVSIYSGVTCEDDVFLGPSMVFT

NVLNPRSEISRRDQYSKTLVRKGTTIGANATILCGIELGAYSFIGAGAVVTKNVKPFALI

TGNPGKQTGWMSRHGEKLNLPLQAPKGKTLTATCPATGEVYQLNGDTLEHDLLSRIGTIL

TS

>tr|D6YVZ1|D6YVZ1_WADCW Uncharacterized protein OS=Waddlia chondrophila (strain ATCC VR-1470 / WSU 86-1044) OX=716544 GN=wcw_0941 PE=4 SV=1

MYMKLLFFTPFFVKLFFTPLLVIMFLLSNCQNREEAPHVKHAHQEINKFKKTALEKYDVS

LIAQGRALGEKINGLSLTFHTREEKSLDEMRNLIVHLATDFLMQINSNNEIKKYLVEYPF

DIKNLNLIILSSDLKKNSPENLESSKEKIATAILIKNNLCYSTKNDDEENFQETFQEALE

KVQSTQSQLTK

>tr|D6YUG0|D6YUG0_WADCW Uncharacterized protein OS=Waddlia chondrophila (strain ATCC VR-1470 / WSU 86-1044) OX=716544 GN=wcw_0399 PE=4 SV=1

MTPFQGFKHGELTKIIHENPEVWSSLFFGPTIGKPREKEAVCHEVLAYLGYSLSEISTLH

DCDFDIQKFMDQKGDETVEKTNKKAASVFVSPKEIPIPPSNSYKKASSLLSYVPSSSLEN

LPQSEDLSEEKSPRKKVSDPQQEPGIPDKEWDEVFSFFED

>tr|D6YUJ3|D6YUJ3_WADCW Periplasmic serine endoprotease DegP-like OS=Waddlia chondrophila (strain ATCC VR-1470 / WSU 86-1044) OX=716544 GN=htrA3 PE=3 SV=1

MNHQPDSISYAKKWSMVAAFMAFVCFPISGFCKDQPLGRTSLDFTEVVKKATPAVVSVKV

KSKTKRNSFQFGDSDPFDFFNHDDFFGHFFGRRPRSPAHPQEPQYQTGQGSGFIISEDGY

ILTNGHVVNGAEEISVLFNDGREYNAELIGIDSSTDIAVIKINDKNLPYLNLGNSDDLEV

GQWVIAIGNPLGLQASVTAGVVSAKGRSGLDLARIEDFIQTDAAINRGNSGGPLLDLDGN

VIGMNTAIVTNMGSGGYMGIGFAIPSNMIQNIMEQLIANGSVTRGFIGINLQPIDNNLAQ

SFSLSKVKGALVSDVTPDSPAQKAGLKQGDIILDYNGKEVSTISSLRNAVSFMKPGSKIT

LKVLRDGKEIHIPLTVGEFPDEEEIASSKTNKLGIEVKPLTADIAHNLGYVDLKGVVVSK

ISAGSPAAMAGIKQGALITAVNHVKIDSVEAFNQQISKADPQKPILLLVKQGDYMHFVSI

KTG

>tr|D6YUI9|D6YUI9_WADCW Uncharacterized protein OS=Waddlia chondrophila (strain ATCC VR-1470 / WSU 86-1044) OX=716544 GN=wcw_0428 PE=4 SV=1

MVNSTDSIKQNYSQIEKQEKSNKDLNKIDKKTYSIARKIFEGTGLILLNILTVFTINFEL

LRGENSFLKREYGRVFHNKEISEIVSGRKDKPIQEAKNEGTDEKIEAPEDPPISSGENVE

PDTISPHNQDLGIVQTNPFIGQPFPLTPNIEQGRPLPEMTFTVLEMNLEIFGMSPEQVLH

SPPPLQIEDQANPLSERSLIPTQNSGYLSSLSSILSQGWNYIAGHEEPHLKADATILIVL

DRVEHLVKISLDTPLTGDRSPIDMVMSNLHQQLPFELQGAHKRVLQLEGGSSHGRLQLED

NPEDLSEDTEKDLINSTPAITLLASTALFEKALREELHLMASSNPQPSIIPPTSSINSIS

KAVGISSRLGFNPRMVGIAGVGVLFSVFALLHTNHSYQRRTASQDHTIAKVNPTKKTQGW

RGYLYDVFKNIISSANSFLGQQIGTARPIATKEFTTGSTRLRFAYMGTPMMNPLMFQPYG

IFPPRIVEEE

>tr|D6YRN2|D6YRN2_WADCW Probable nicotinate-nucleotide adenylyltransferase OS=Waddlia chondrophila (strain ATCC VR-1470 / WSU 86-1044) OX=716544 GN=nadD PE=3 SV=1

MKQIGFFGGSFDPIHFGHLKMAKELKEKKMLDEIWFSPARISPFKLDRCPESVENRLEML

RLALGGEPGFKIYEEESRRLGPSYSIETVEHLSEIPDCQFYFIISDESVPEFFHWKEAER

IVQLVPLIVGSRVGAEPPKKGNETICQAMERGWTPTQILDISSTQIRKFLKEGKDCTSYI

PRNVLDFIYQNHLYSSTNYE

>tr|D6YUU4|D6YUU4_WADCW Uncharacterized protein OS=Waddlia chondrophila (strain ATCC VR-1470 / WSU 86-1044) OX=716544 GN=wcw_0534 PE=4 SV=1

MKFPKYLLTLLLFLFVQLDAATFLKDRLQSSRDGDYIVTRIDNTYTVLLIKERSEHQISI

EEISIPVQRLHDKRFPWAGWKHWVENGANGHTSWLLYTIHVDSGMMREYFSYTSEQWHSM

SDVNNFLSTLLNLRFVKIPRENMKRVGVVPPSEKYGQDSRRIWTPKLVYEGETIYGAEFE

AWRTRWPRDCSELSGKTITVYLPEDEKKYPTYFPYWLEIQGMLGKAKISIVDSGHRMRSP

RSAPPRKVH

>tr|D6YTJ0|D6YTJ0_WADCW NAD(P) transhydrogenase, alpha subunit OS=Waddlia chondrophila (strain ATCC VR-1470 / WSU 86-1044) OX=716544 GN=pntA3 PE=3 SV=1

MRIFIPKETSSKETRASATPASVSKLISMGASVQIEQGIGNSLYLSDSEYLCKGAEIVES

RNLALRDADIVLKLNPLSCEEVGSMKKGAIYLGCMNPFQNPGLIEAFREAGVSAISVEMI

PRTTIAQKMDVLSSQASLAGYVAVMTAAQHLNKIFPMMATPSGTIQPARVFVIGAGVAGL

QAIATAKRLGARVQAFDTRPTVAEQVQSLGAKFVKVDLGETSQTEQGYAKELTSEQLLLQ

KEAMAKVCVQSDVVITTAQLFGRRAPLIIENRIIDMMAPGSLLVDLAVESGGNVEGALPD

QIIERNGVKIIGLSNFPGQVAYNASEMYSNNLTNFLEHFWDKESQSLRLNLDDEIISRSL

ITHCGEVVNSQFQSLQPA

>tr|D6YV37|D6YV37_WADCW Putative rhs family protein remnant OS=Waddlia chondrophila (strain ATCC VR-1470 / WSU 86-1044) OX=716544 GN=rhs23 PE=4 SV=1

MDLDGRQFAYDQNGNLESFETEHNCVYDALDRLIEVHTQECTCLYTYDPFHRRLSRTVLN

QEGSLQQHYLYSGDKEIGLLVQGEIRQLRILGNPVEEIGSAVLFEIDGIKYVPQHDLRGN

VALLLSPAGKAEVYRYSAFGEELFQESVSPWRFSSKRVDEETGWVYFGRRYYAPSWGRWT

TADPAWFADGPNLYAYVHNNPLKYVDPDGLSAIEHQQMNRPGTQGTFFGSFSRGILDDTS

WGASSWMLGDYVCDNWQSSLGYGMGTGVSMMAGLVYGGTEAKLLGAAGKGLNRAARWLNF

AEKEVKAACKMEGTAAKISRDIAPKIERNLPDVGKGSYVPGVGGSPKSMPEGHMWTSGSP

FKNKTAEELHQMFVDKG

>tr|D6YTG1|D6YTG1_WADCW Uncharacterized protein OS=Waddlia chondrophila (strain ATCC VR-1470 / WSU 86-1044) OX=716544 GN=wcw_0045 PE=4 SV=1

MDAHNPSDPFDDPSKHVRKKKKKIKRKSALEAPPSNMSAAGDSEIDTMLKKLRNMDDDLQ

NRMEKIAELSGMSTKEVKRFIENPDNFPSEEWSRMQRKKDDLEKKIYAGIGIEYQKRVQQ

KEKKENAKRKERENPRL

>tr|D6YWR5|D6YWR5_WADCW Uncharacterized protein OS=Waddlia chondrophila (strain ATCC VR-1470 / WSU 86-1044) OX=716544 GN=wcw_1219 PE=4 SV=1

MCKVALFLIFLVGTLCGESVYPVDMSFLIADIKYSQEHGIKVCEMQHGVVSTFRGDVFSH

DGEPVIAQNLFDTLALYVKEGWTVPQSFGDATLKKMFANQENWHAKNEKEEIFNDLKFLE

AARKAPFNSERMNGYHGFVYLRSPSPKELDELHEAYPGVIFIDRAISPYWANKAKMSALF

EQDPMLGLIKPKWNVYPKTYTPKLAERIKQELGTQLFVIKPKGGCSGKGVIIVSEGDLDA

VLKYILKKSRSLKKNRDKSYNYWYHTKETEFIVEQFFASDPIRVPHLKNELYQPTMRVSF

LFVYEGGEAKAHFLGAFWLLPKNSLEKGGSLNDTYKSCCGLPRYTAVDSELEEQVAAECH

DPFLLFYQKMLENQVD

>tr|D6YRR9|D6YRR9_WADCW Uncharacterized protein OS=Waddlia chondrophila (strain ATCC VR-1470 / WSU 86-1044) OX=716544 GN=wcw_1414 PE=4 SV=1

MNLPVNSYLLSPFIYWIFFCHSLKANLSRFGKKNDKEISQKMKKPEPLASNG

>tr|D6YSA1|D6YSA1_WADCW Uncharacterized protein OS=Waddlia chondrophila (strain ATCC VR-1470 / WSU 86-1044) OX=716544 GN=wcw_1601 PE=4 SV=1

MSSAPIGGEGTGGIIPVNSESEQTPQTQEIDPQVFIDQIEQMIESDDNIRLLAKGQIGLP

PGRISIAELTALINAVILKLKKEIQAAEQLDALISERFHRSIAQTAESLEGLHGELNDIV

VSWRNFTNENGETVHDAYDNLQQATDEYNQMMQNLWGEDRAAIQEYNQIIHQYNNLDSDG

VDDIYDSLSQAERDALIAGLTQSEIDAYGGTKEAVVAKYFDKKIDAFNQYAASRPNVQAA

INNLQQATADYNNVVDDYNLHIDQVNAERAEYGLGPLPYMVKTPTPNPNVAFTNLSQQPH

VQAPLLPTPNRQLLAVPNASQKGTVSEPTYPGDPPSVPSYEMMILHFIPIALTTFDYLAS

FNRALDLADAYVQWNRYALLTGAQLTLPYGVLNQINNVFLQSVNAIGGVGLAAHAIGLHS

RSLEIILSRALFQSLAQQLSLPISARLFSRLQFTALELLSRSALLSAYPSMRFLASRFGF

LAASSPSVQAAIALAFAGQIGSLVSSGIVRGIINGQINRLGFYARHNVRFSAAEVEYAER

NLANAIASGNPFLIASAVRRLTSAQVRLANAVRLSRTFGSLSIGGYAAFTNRVAAALNIS

LLGVSTSLFARTLGLPGLVAQIFAHVTHLSPIDILIASTAGSSIRAVLDNPLSVLFIKQN

LANQLVYRLGFSSSYAAAIVNNAINNAVFASVGLHTYSRLHNELYLQFRAEGLSPYHANR

LANETTSLIRGDLGVQFLNVVFGIHADTSLIASTVVNSIYGFDKGIAGVMLSNAIVRSLQ

YGGYGSRVRLQNELTEVFQSMGRRDGSELAAQYVRFIETVGSLVPLTRYPGLASVLLGNG

LLRNLALGGAFVRNEIRNDLIMRGLSPTQAAFVADQLIALSTGNFVRPSDQLLFELTLNA

AIERALIHSGSFETHREFRDQLINELRSVGFRLNDALFLANSIAAFAANGEALSLLGLSA

GQINGLNISLVNELTVSGISGSQAQTIVDRAYANTTAKAPFFSPDDFQNTLKEEIFRQTF

LASGRLDGHEIFDKAAAHAQDPSTILDLASLIEQISGAAQGVFRPDLGSELAQEVHNALL

NALLGGTTIDEISNEETRNPLSVLNQLNDQIDKLTKDEEIANLIKLLKKLQELLAALLTG

SPEGHLLGTSIMDAASGPFFGAANVSQGGQDYQAMQIPV

>tr|D6YTZ3|D6YTZ3_WADCW Uncharacterized protein OS=Waddlia chondrophila (strain ATCC VR-1470 / WSU 86-1044) OX=716544 GN=wcw_0229 PE=4 SV=1

MLKMWPKSYKDYPKDQPLKKERVTAGKGIVGCEMHEINGWGSTHHHPRPRKKGFFHHLLE

KLFQGRK

>tr|D6YUT0|D6YUT0_WADCW Uncharacterized protein OS=Waddlia chondrophila (strain ATCC VR-1470 / WSU 86-1044) OX=716544 GN=wcw_0520 PE=4 SV=1

MKVFDKFSLLIKVMLLSLLFLVNLIHGYFMKINKFILLSKILIVLFIFNLNTLCAARPHR

AYSIESSLEARHPNVRAVILQLKRMPESNEVIRQALGMGSITVEMSSKGMPFNAMWENYE

RRIVVDKRASKDQGSLLCHLLFELTNAVAEPRYQELCELAIDGLIDCDSYVEAVERIEYE

NMVRTVAIIEKGISSGIFPSTAGWEVIHDFDIHYKIQQLAGHSLLIAKEYQEITGRKRFS

SYQGTVKNLKRMSHSEKMSLIEYLSSQYFHSKRKISNA

>tr|D6YRX7|D6YRX7_WADCW 3-hydroxybutyryl-CoA dehydratase OS=Waddlia chondrophila (strain ATCC VR-1470 / WSU 86-1044) OX=716544 GN=crt PE=3 SV=1

MKYLEFEKRNRVGLLKINRPKALNALNLELLKELEHCLEEESEKLELVAMIVTGAGEKAF

IAGADIKEMQGFDKQQILSFISLGQRVSLLLEKAPFITIAAINGFALGGGLEMALACDFI

YAADSAKLGLPEVMLGIIPGFGGTQRLVRAVGVRQAKEMIATGKAISADEAHRIGLVNCV

CSKESLISECLAVAGSIAGHSQTAIYQAKDAINREDGLSIHEGLDLEKSNFAICFETPER

EKAMQAFLEKSMRGTSA

>tr|D6YUH8|D6YUH8_WADCW Tyrosine-specific transport protein OS=Waddlia chondrophila (strain ATCC VR-1470 / WSU 86-1044) OX=716544 GN=tyrP3 PE=4 SV=1

MLALPVLTSLGGFVPSMVIFLLCWLFMAGTGLLFLEVCHWMNGDTNIVSMAGKTLGKGGK

AFAWLLYLFMFYCLTLAYIVGCGNLVTEILPLPAWAGSLVFVAVFSPAVFFGARVVGRVN

SLLMVGLLVSYFVFVAIGIPYVNVENLKHIDWPSSLLALPVAFTSFAYQGIIPTLSRYME

FNIRKTRSAILLGSFLPFAAYVIWQWLILGIVPTFSENGLADTLEVGGNAVDPLKFFIKH

SSVYTVGQFFAFFALTTSFYGVTLGLLDFLADGLDIKKDAKGKFFLSMLVFVPPLFLAVS

YPRVFLTSLEYAGGYGSALLLGLLPILMVWSGRYCQKLPSKYSLFGGRITLVLMLLFVIF

EIIFETAHLIFH

>tr|D6YTI3|D6YTI3_WADCW dTDP-4-dehydrorhamnose 3,5-epimerase OS=Waddlia chondrophila (strain ATCC VR-1470 / WSU 86-1044) OX=716544 GN=rmlC PE=3 SV=1

MKMTPAPLEGVFLINLEKREDERGFFARTFCSEEFREKGLETAFVQANDSFSIEQGTLRG

MHYQLPPFAETKLVRCIKGSLYDAVLDLREDSPTFGQSFGTILSADNRTMMYVPRGFAHG

FLTLEPYTEVYYLVSAPYSPKFERGIRWNDPRFNISWPEPPRVISERDNSHPDFL

>tr|D6YV14|D6YV14_WADCW V-type ATP synthase subunit D OS=Waddlia chondrophila (strain ATCC VR-1470 / WSU 86-1044) OX=716544 GN=ntpD PE=3 SV=1

MAEIKFTKNELRAQEKKLGQLKKYLPTLQLKKAMLQAEIYEARLEIDHCEKELQKQRGKA

EEFSPLLSARLSLNLKDVARIDKVEKRYDNIAGVEVPYFESISFHKVEYGLFDTPAWVDA

AVILLRKLAEGKVRVMVAEEKKGALEEELRMVSIRVNLFEKILIPRAQGNIKKIKVFLGD

QELAAVSRAKVAKQKIEQKKTQLAT

>tr|D6YVV3|D6YVV3_WADCW Putative glycerophosphoryl diester phosphodiesterase OS=Waddlia chondrophila (strain ATCC VR-1470 / WSU 86-1044) OX=716544 GN=wcw_0902 PE=4 SV=1

MLNKTPKLIAHRGNSSQAPENTLAAFISAIQIPVDYIECDVQLSKDGVPVIIHDGTFHRI

TNETHPNKVNELLLEEIKLIDTGSWFDTSYSNQRVLTLEELLLFPKGKIGVMVEVKEETF

LACSMGKQIGDVIKRVTPRISQYGPILLGSLSPNVLLCLEAYLPQQALLPIVKDLEDLDD

FRPIHAKHYAFKHTLLNEEMILEFHQNGIEVWAWTIDDKDTAFQLAALGLDGLITNQPKK

MTGICHPSREMVDNVANVVFQGRLKKVN

>tr|D6YUJ2|D6YUJ2_WADCW Uncharacterized protein OS=Waddlia chondrophila (strain ATCC VR-1470 / WSU 86-1044) OX=716544 GN=wcw_0431 PE=4 SV=1

MRQGFDIVIVSTRNSSQEEFWHKRLRSSSKEICKPGALIITIAENWAGGAGTGLGTLYAY

QKAAEKAKFKYHCDLLQMHNAGASVALYHTAGDGKNLFPLTASENNSIANLRILSKNCYL

LETVIKQTNEHFSKNRERRLSVFHCNQIFYPSKTFSYSPDSHIDLFCQRLPQHQPGKKLI

VLKPAGARVFDMAKPIFLQTKSPMASNLSSFSLSWEMLQALLKEFKQELGKESGQMDANA

HFWMPLTLNYQTYASLIPYPEYQMHFHRMADLKKQLVKQDPSRSFFKPLDIGTDGYWWDF

RSVNSYYRTLLKLTGNTKESHYMRSLFNLNTPANYCLENRVIMDRNSYLIDSQIRGGSIK

NSVLIGVEAESLTIENCVLINSQLSSLETSRSFLYNVKENRPLRLASGTVRADVALEMEH

KAYHLYSHLSRNGASDWNTLLPQNPLSFSEIHQLVEQF

>tr|D6YRL8|D6YRL8_WADCW Uncharacterized protein OS=Waddlia chondrophila (strain ATCC VR-1470 / WSU 86-1044) OX=716544 GN=wcw_1362 PE=4 SV=1

MFIQDSYQEAHEQFGISEVYIIDGLDEATLKKLQLRAKLTLDSNDTIVKNNVSYDMLFIF

HKRVVDRSFMYRFIEFLRNNPSKEKDFRKLYELINPEEILETVKSKKYVLVSKKDLSR

>tr|D6YS29|D6YS29_WADCW Uncharacterized protein OS=Waddlia chondrophila (strain ATCC VR-1470 / WSU 86-1044) OX=716544 GN=wcw_1525 PE=4 SV=1

MIKLTLNPDQESKVITLDKEVIVIGHSNADHVDLPMDIPGICARHVVIEEQNERFVVINA

ANDPFTSLNGLPFGKKPLKNYDQIEIGENKIKFEFIEDDAPTTQTVEKQEDVPDEIDKLL

KEVEELSKPSLETAPPPSLPAIEDQKAEEEEEEAESLSSKKYYLRDFDDESEQWSEDRLE

ANNIYSPGRESFADSWKMLAGLLLAIIALGAVICSGVYFRASGKNSQEEKKIAAGIADIA

MAMTHAKLNHITPNKQNWSDPNFIRNNLAQVLSPNLHTQAQIDSEGQFTKYPYRLRVYTS

RSLDQFIVIAQPAPNLMQWLVHKKTLVVDSSTMEMRKISDLKALNRLLANPDPLEGSNGE

DIHHLIKEGGLMSLNSLAGHKNHWGFSPPKTLGFIRPGAENYIYNAPRYYPFGEELLRKA

IQLYKNSSSPSDVAIIQDEMDEISNFPNIVLYTSEGLQMAVEAQKALNTFAPNSKFLVAY

VKFNPKGFVASSHLLINEERREIAFLPSPRTELSTFFPTSSEMEQEPDFLSLSPIEDSLL

IAEKTASDDTDLQHPLYLQLKASHQERKHALNAISRKMLELLNQQNNALLPEFEPSFSEL

LSEYLKTSLHYQQKIIQKLAKLYQEHTDMPLEEFVKYVDQAQLSSFAQAALDDQPTQRLT

QEEIEEIFEKIDRAESLHELEHVSEQAAQTLTLENLPDTNSLIQFQEKVRTHTLNRLQFL

LFSPKSLYATEPLDERDRGVLINILENSWISDPGEKDYFLSEFDHLIEEFVKSG

>tr|D6YTP0|D6YTP0_WADCW Uncharacterized protein OS=Waddlia chondrophila (strain ATCC VR-1470 / WSU 86-1044) OX=716544 GN=wcw_0126 PE=4 SV=1

MILSMKTVTSYLLLICLLPVLSWAEQKVSFHQISKTINNEKIEISGYIYQSENGEAYLAP

QPDLKSCCIGSRQRSGEQIVLKDLESIPATKQPVTMRGTLRIDPETGQMFLTEGEAVPKE

NRLHLLFAAAAVGTAIFFFLLRKRI

>tr|D6YT94|D6YT94_WADCW Mutator family transposase OS=Waddlia chondrophila (strain ATCC VR-1470 / WSU 86-1044) OX=716544 GN=wcw_1956 PE=3 SV=1

MFLKTYRGKYPKACACLEKDKAQLFTFYNFPAIHWQHVRTTNPIESTFATIRHRTRQTKG

CGSVTVTLTNYSREKTEKTQGL

>tr|D6YUH3|D6YUH3_WADCW Uncharacterized protein OS=Waddlia chondrophila (strain ATCC VR-1470 / WSU 86-1044) OX=716544 GN=wcw_0412 PE=4 SV=1

MKAIPSPSGLPIYHRGAPLEEGKLPAFFYFALSGKDSLFLDPFNQPALFLESYPLRTFSF

TIPGHGEGLKNTEAMGFWAEKIEQGVNPLSSFLEKCLENIHFLIQNGIVDSERMAVGGLS

RGGFIALHLASRVPELSTVLGFAPLTTMATIQEFNKIHNHPITQSLEIEKSLLVGKKVKF

YIGNRDLRVGTDACYQFVRELTDLNYERQIRSPDVTLVINSSIGHKGHGTPPSTFQEGAE

WIASLLTAKTV

>tr|D6YU78|D6YU78_WADCW Isoprenyl transferase OS=Waddlia chondrophila (strain ATCC VR-1470 / WSU 86-1044) OX=716544 GN=uppS PE=3 SV=1

MTAANAVKSWKESPVISDKELEELHASPIPKHVALIPDGNRRWAKKHCILPEQGHKLGAD

SLLNIIKAGHQIGIETLTFFIFSTENWLRPKKEINAQMKLLEKSLTLQKPRMLENGVRFR

PIGDLSKFPPYLIELINRTEQETAHLDNINVVFAMNYGGRDDIKRAFIKMLNAYEHRIFT

KEEISENLISEYLDTCEWSDPDLLIRTSGESRISNFLLWQLSYAEIYLTKKHWPEFSPRD

FLEAVIEFQHRERRLGGS

>tr|D6YWT5|D6YWT5_WADCW Putative membrane protein OS=Waddlia chondrophila (strain ATCC VR-1470 / WSU 86-1044) OX=716544 GN=wcw_1239 PE=4 SV=1

MTFEQIQSIFNRALFNTFHKAKLILCYFVLAMCGVLVVFFKGVSMQAGQWLAQSLTFLPI

FLCAGVLFSLGILLIRIYHDEVKGKRISYRKTLWRSWEVVVGSSYVSIPIILCYLLVWMF

LGIFALLSEIPSVGPFFHAVLAFAPFLLNLSCLVLILFHLALLFYVAPILALKGFNRLHI

SKTLMERFRQDVFANGLMLLIAMLPLLLLLTILSLAAWMTGSLCVGCSSVTETVLLWFFI

MIPFTAILAPAVVFFFNFSAESHVLIMKRVRDSHPHKS

>tr|D6YTC2|D6YTC2_WADCW Uncharacterized protein OS=Waddlia chondrophila (strain ATCC VR-1470 / WSU 86-1044) OX=716544 GN=wcw_0006 PE=4 SV=1

MFNYHQIILFLITTLLFIPVSSYRISKSYEKLLSAQDKRKACSRMVNDILQTVFVLLAFF

AALAFTVIMNIIHRTNG

>tr|D6YUY1|D6YUY1_WADCW Uncharacterized protein OS=Waddlia chondrophila (strain ATCC VR-1470 / WSU 86-1044) OX=716544 GN=wcw_0572 PE=3 SV=1

MSQEKNPKSFGTHDGSFHADEVTACALLLLFELIEEEKIHRTRERSVLEKCEYVCDVGGI

YDPSKKLFDHHQVDYQGPLSSAGMTLLYLKDSGVISEKMYHFYNETLIIGVDDHDNGKDM

QPRGHSSYSYVVSNFAPIPYNPTEEEQNAAFREALHFAVGHLGRLRKRYEYVQSCRQVVE

ETMKSSDEVLIFDEGIPWLQLFFDLGGKNHPAKFVIMPSGPHWNLRGIPPSYEERMDVRI

PLPKEWAGLLEGELKKVCPIPGGVFCHKGRFISVWETKESALQALNYVLSQVKEKR

>tr|D6YVX1|D6YVX1_WADCW Spermidine/putrescine-binding potD protein OS=Waddlia chondrophila (strain ATCC VR-1470 / WSU 86-1044) OX=716544 GN=potD PE=3 SV=1

MNLRKTCFFFLLVILSSCEDNRQRTLHIFTWSEMLDTRLVHEFEKEFNCHVVIDLYDSNE

SMYAKVKLGNSSYDILFPSNYYLEIMSKQGMVKPLNFDLIPNHLYLDPHYFNSESDPYGI

PFMLSYSGLGYRSDQVAPPPSSYNIFGSKEYIGRMTMLNDTREALGAALRTLGHSVNSTD

KNAIESAADLVIKWKQNLAKFESEQYKNGLINSEFLICQSYSSDILQVQVEADLVKFSFP

EEGAILSIDYITISEHSPEPELAHAFINYMIAPKAAALNIQRTHALTPIPSSYQLLPKHL

RENPILFPSEDQLKAMEKIRDLGSDVRLYYDAWERVKGS

>tr|D6YUC1|D6YUC1_WADCW Uncharacterized protein OS=Waddlia chondrophila (strain ATCC VR-1470 / WSU 86-1044) OX=716544 GN=wcw_0359 PE=4 SV=1

MSFYPITSISQFFQLSDSPVDQQSASFIKLGFSEAQAEFLTDAGYKTDEVQQRINDGFTP

EEIYNAVIVKQLHDRVEIAAYRALQEGIETLQLIKGRTSTRISSTESDYLLHDCIRKAAH

ADMRSKDGYITMRGKYLNTKQITPVIENVSVITTDRDSKLTKATYARFEELLANLDEHNT

MVNTILFNAWGVPQKKEHIHLDEIKKKAEFTGPKPLD

>tr|D6YVL0|D6YVL0_WADCW 50S ribosomal protein L36 OS=Waddlia chondrophila (strain ATCC VR-1470 / WSU 86-1044) OX=716544 GN=rpmJ PE=3 SV=1

MKVKASIKADPSKGDKLVRRKGRLYVINKKDPNRKQRQKGPARKK

>tr|D6YX01|D6YX01_WADCW Uncharacterized protein OS=Waddlia chondrophila (strain ATCC VR-1470 / WSU 86-1044) OX=716544 GN=wcw_1310 PE=4 SV=1

MADTIRIFVTTCKPMWRVEKALEYTVHKYCTPPFKVTFLRSGDPDWLTNVDLSISHKDKD

AIKKAGCWNIGRDHPRPYSGEGWATPFTCFRFAIPELCSFEGRAIHMDADFIVQNDLRKI

FEIDMTHPIMSPHHRTDFMLIDCSRIAEMQMMGMWPSIEEMKVSGDNIDVYRKKLQNYMF

IGDAPREWESWDGKDLAENSFSIHYTEMRTQPWKPYPEYFDYPLYPDKNAGYLFWEEYAE

ALEAEARGEITLSAKDQTSPDATPLSRKA

>tr|D6YW72|D6YW72_WADCW Uncharacterized protein OS=Waddlia chondrophila (strain ATCC VR-1470 / WSU 86-1044) OX=716544 GN=wcw_1024 PE=4 SV=1

MKKVRECCHDSLPFKEIFSELTPRAFFKAIAYRILLKEKYFLYCKKTRIICINFSF

>tr|D6YT54|D6YT54_WADCW Putative general secretion pathway protein F OS=Waddlia chondrophila (strain ATCC VR-1470 / WSU 86-1044) OX=716544 GN=gspF PE=3 SV=1

MPLYAYQAIDSKGKKKNGLIDAHSEKDARSRLRDQGVMVASIEMKQGAVSKENMNGDQLL

AFTMQLAQLVGAGVPIYESLIALEEQCRHERYHRILLSLCEQIKAGKSLSAAMEDFPATF

DKLYYSMIRAGESSGSLDVVLEKLAELLAKQYKLRKDIMTAMIYPGILAGFCLVLIIILL

GFVVPMIEGIFQGRELNAFTAFVMGLSHAFRAWWWVYVPVVFTFATWAFFRLRTPEGKSW

LERQLLKIPVIKTLMVQAAIARFTRTMGTLLNGGMTMIDSLKIARNVMLNETLEKEVRKA

EGKIIEGSSLSKELSKSEWIPQMVSRMLAVGEDSGTTVVMLNKIADIYEENLEKTLDRLM

ALAQPIILIFMGLVIGTVLLAILLPLADMSSFSL

>tr|D6YSK6|D6YSK6_WADCW Glutamate dehydrogenase/leucine dehydrogenase OS=Waddlia chondrophila (strain ATCC VR-1470 / WSU 86-1044) OX=716544 GN=gdhA PE=3 SV=1

MKTLNKERNSQLKIKEIPIPGYEKVIEAKNAESGLHCFIAIHDTTLGPSLGGARIYPYSK

WEDALEDVLRLSKGMTYKSAVVENGFGGGKSVIIADPKKNKTDALLESFGQVIDTLKGKY

IVAEDVGSSVEDMIVIRRQTPYVAALPTEKSSGDPSRFTAWGVLRGMQAVAMKLWKSPSL

KNKSIAIQGLGHVGSGLANLLFWEGAKLFFSDIDEERLKTFTRKFGAGIIASGDFYTFPC

DILSPCALGGTLNEETIPNLKCQAVAGSANNQLFEKKDGRRIKDRGILYAPDFVINAGGI

INAAAEFDEGGYDPNRSRERVDKIYDTLIEIFVRSEVEDLPPSQCAIDLAKHKLMRKIGK

REQPIEFQR

>tr|D6YTJ8|D6YTJ8_WADCW SET domain protein OS=Waddlia chondrophila (strain ATCC VR-1470 / WSU 86-1044) OX=716544 GN=wcw_0084 PE=4 SV=1

MFFWNSNHETPLTQAVISGNTELVKRLAHHSVHRKAANYLGFSAEDLAIYLGREEMVDLL

GLQKNKVFRVLKKGGNGVVEMDVCEYEKFFHTKYMSSLRVTSYQDFCKIVKKCPKQVKVG

KVGASMRDLFESHKEKIKNGYVCESTIKWIDERTGYGLFTDRPINKGEFVGEYAGLLLIR

QILSRIRGDYCMRYPKLSFGLSYYTLDAEKMGNEVRFINHDYVPNLQPMSALENGFCHCV

LIALRDIKAGEQLTYDYGEDYWSRRDPPVDF

>tr|D6YTJ6|D6YTJ6_WADCW Transcription elongation factor greA OS=Waddlia chondrophila (strain ATCC VR-1470 / WSU 86-1044) OX=716544 GN=greA PE=4 SV=1

MSYLEEFQNQINNRDFHKFFQLWEEYCTNDEVDSDEFISLLDIIKNSDFNTLFGQFAETA

LPLWQCIKDDEESYQVLKRILDLQTSNSSLLASTAISMLKKRYGEDPKFKERLRQIGLRT

QANFQGAISNFELLNHMAKGKFVFHTSGWGTGEIMDISHIREQVAVEFENVTGIKHLSFE

NAFKTLIPLLDEHFLARRFADPDLLEKQAKKDPLEVLKALLHDLGPKTAGEIKDELCILV

IPEQEWSRWWQNARARLKKDTMVETPNSLKDPFILRKKELSHEEEMHREIEKQTSVDDIV

QTTYTYVRDLPHVLRRREVKDTLRDKLVSLLDEEQLSSAQELQICIFLETLFGHSIEGKS

VKDFIHSMENVEEVLNSMEIIAFKKRALTMIRENREDWAETFSSLLFSIHQNPLRDYLFQ

ELQSSESRELLMKKLNTLLRYPERHPEIFVWYFQKICKKNPGNIPFSNKEGQCQFFEAFL

ILLHRIEHENEWKDLVKKMYNTLTSKRFEVVRNLIENTTLEFIKEFLLLVAKVHVFSDHD

KKIMRSLAQVVHPSLAPSKSKSITDDASTLWTTEEGYRKTQERVKRIATVEMVENAKEVE

EARSHGDLRENSEYKFACEKRSRLQSEMKMLSKQLSAARVLTPEDVDSSIAGVGCIVEVE

EPSGAKEQFTILGPWEADIDNGIISYQSQIAQAMSGHKIGDSFTFKDGHYKITALKSVFE

G

>tr|D6YWX4|D6YWX4_WADCW Pyruvate kinase OS=Waddlia chondrophila (strain ATCC VR-1470 / WSU 86-1044) OX=716544 GN=pyk PE=3 SV=1

MLSAILSIVKRDRLMACRTKIICTIGPAVNTYDKICALIKAGMNVARLNFSHGSYEEHFS

VIEMLKRARKELQVPLAILLDTSGPEVRVGKIQDGEIKVNKGERLRLLDKEVLGGNGVIS

VNPPGILKGLSVGAQVLFDDGYISSRVKEVAEEWVELEIENYGVLKGGKGVNIPNVSLNL

PSVTEKDVKDIEFGCRNGIDWIAVSFVRTPENIITVKNLLESNRCSHVLVIAKIENHEGI

EHFDSILQISDGVMIARGDLGVEIPLSHVPRLQKEMIRKCYLAGKPSVTATQMLESMINN

PRPTRAEVSDVANAIYDSTSAVMLSGETAIGKYPIEAVEMMRDIISEAETDFDYRSLFEL

HSSISYNDVPSSVTLATVKTAYSSGAKAIFAFTSGGGTARLLSRLRPELPIVAMTPKENF

YHQLSLNWGVIPFLGKESKTFEEGFEQVSQFALKNHILSYGDLAIATAGSTFGIKGTTNM

MIVEHIGDVLVRGHLGEGEKVYGNVKFLRSPSEGLQPYHVRGALIVITKCDESYLPYIQE

SAGVILQNHIDDEASEMFAIEQAGLFHKPTIVRADAAAYILKEGQLVTLDPGKALIYKGV

VI

>tr|D6YSX6|D6YSX6_WADCW Uncharacterized protein OS=Waddlia chondrophila (strain ATCC VR-1470 / WSU 86-1044) OX=716544 GN=wcw_1832 PE=4 SV=1

MDKMTVVLLVALMFLASCSAYGGKMSEKRKMAYEMVDELTDPYEELYGLRLSGISEAAPD

GKYDNLGMDFHTYRRLSKDEGRRLILKIMDEFLDKINNTQAFRKYLTVHPFDSNHIVINI

FVNEKKRGERIFFPDIDSVSIYNEKIYYDFYLKELGNAVKDKQREMETIEEARRIVESQN

HKINNG

>tr|D6YSX0|D6YSX0_WADCW Putative outer membrane efflux protein OS=Waddlia chondrophila (strain ATCC VR-1470 / WSU 86-1044) OX=716544 GN=wcw_1826 PE=3 SV=1

MRSKGWLVLLLSMAGCTLGPHYTPPYVDNPEYWRVSLEDVRGSINADWWYQFDDDVLVQL

IGNSLEGNQDLIAAKYRVEEFLGLYRVTRSNLYPQIGGSAEYSRQKLSLGPAPLLPGMKN

PNDLYQLMLNGSWEIDVWGKLRRATEAACRDLLAAEENRLVVVQTLVSSVALAYINLLRL

DRQLEIAIETAKSRGKTLELFQKRFAAGVISEIDLSQIESQYREALATIPDFEQRIERQE

NAISVLLGRNPGPIPRGKTLHTLTLPEIPSEIPSHLLLQRPDLRAAEQELAAATARIAVA

RAAYFPSISLTGAYGTSSNELSSLFTAGTSLWNYGVPITMPIFTAGRISGEVKAAEAFRN

QLLATYRQRILEAFQDVNDSLIVFQKRREQEEEQRKQVESLQVYARLARLRYDEGYASYL

EVLDAERSLFNVQLEYSEVYANLFQALVALYRSLGGGWIYVADPLTECFPEEIENHLP

>tr|D6YU25|D6YU25_WADCW Uncharacterized protein OS=Waddlia chondrophila (strain ATCC VR-1470 / WSU 86-1044) OX=716544 GN=wcw_0261 PE=4 SV=1

MLAIFRKYQRFLYLVITCVIVISFSFFGTYSGLQNAQPADKTVFQAVDGSDVARSELEEM

AAFIGTDNEDKMLLGGRWGPNFLNDGVVKKDFLETGLGALLAKPFMNDLRSELLTRQQRE

RHYQSYKHPQAQFISAENAWNILAPEMNKRLADLRKVKDPATEEGFDARVNLFLAERKFP

HPALRMVLGHQQKQYKWVAPDPNLNYIDLALFGYHTADDWFGSRFIRLVSEFIINSAIIA

QEKGYTVSRKEALADLYRNAELSFQQNGSNPNLGVANAHQYFQEQLRRMGLDANRAAALW

QKVMLFRRLFHDLGNSVWIDPYTLANFQHFAKETVEGSLYQLPENLRLTDFRDLQRFEVY

LSAVADRPTSGMGMLELPKTWKSVDQIVQETPELVQRRYVLEIAQVDKKNLQAKVGVKDT

WDWQVQDHSWEKLKQKFPELGVKAGSNEQERYAALESLDQKTRSRVDAYSREQIVDEHSE

WLDQALVEAEPVETVVGIRKKGNVGIIAGLLNPEELITLLDQYPESRSKLEKYSADGQSF

YRIAVKEKASGYETLTFEAAKNDGTLDRLVDARLKAAYPQVREDDPKAFSERDGSWKPFE

QVKQKVAEYEFANILQAIRDDYTAAQPPEEKGNVVLNDFAATIRLYPYVRGLREQFKADP

ESIEEHVTARGRQEIASSVKDQWKLLKSSYTGDRSTPDLPMDEQEVFSLEKGEWTSVKKR

GNGNFSFFQLKEHQNAADEAGVVEKVMHAQRDFSFEAQRVYMQKLIDQFREKNALTLQYL

NPVQDSEE

>tr|D6YV74|D6YV74_WADCW HNH endonuclease OS=Waddlia chondrophila (strain ATCC VR-1470 / WSU 86-1044) OX=716544 GN=wcw_0667 PE=4 SV=1

MPKKRQPNEIWQITRVKVLERDEYRCQRCLTSLTVKTAHIDHIISGKRGSNHLSNLRALC

RRCHVLRADSRHRGMIASALRDGIIDVNWRDEVWDN

>tr|D6YSR8|D6YSR8_WADCW AAA domain-containing protein OS=Waddlia chondrophila (strain ATCC VR-1470 / WSU 86-1044) OX=716544 GN=wcw_1773 PE=4 SV=1

MDTMKRNLQKHILEDLPKKIVLISGPRQTGKTTISKQLCDQFDYFNYDSGEDRLAIRQKR

WDRSKLLIIFDELHKMKQWKRWLKGVFDTEGIPPQILVTGSAKLDIHKRVGDSLAGRYFQ

YRLHPLDLKEIHQFLNVKIEDGFNTLWHCSGFPEPFLEGSRTYYRRWRRSHIDIILRQDL

IDLSSVRDIESVQTLVLLLSQRTGSTVSYANLARDLDRDPNTIKRWLQLLENLYIIYRVT

PYSKNVARSLKKEPKFYFYDHALIEDEGARLENIVANALKKELHFLEDTQGIKGALHYLR

TKDGQELDFLVTLDGVPTHLIEVKMGDDKPAYGFRHFSKLFPDAMHFQVVKNLLRDTSLP

NGLFIKQVIPWLAELSLLTK

>tr|D6YUI2|D6YUI2_WADCW Exonuclease SbcC OS=Waddlia chondrophila (strain ATCC VR-1470 / WSU 86-1044) OX=716544 GN=sbcC PE=4 SV=1

MRILQVRFKNLNSLVGEWQIDLTHPVFASDGIFAITGPTGAGKTTILDAICLALYGRTPR

LNKVTKSGNEIMSRQTAECFAEVIFETQTGRYLCHWSQHRARRKLDGELQAPKHEIANAD

SGDLFESKIRGVADQIESVTGMDFDRFTRSMLLAQGGFAVFLQAAPDDRAPILEQITGTE

IYSQISICVHERQREEREKLNLLQAETVGIVILETEQEQEIGQTLETRLKEERDLAAKSA

DTGKAITWLANIDGLKKELANLADEASKLQGDIEAFKPDREKLGRALSAAALDGAYATLT

AIRKQQADDNAGLKAWKETLPELESSAKELAKALKSAEQQTALIKEELKAAVPTLQKVRS

LDQKLTDQKKAVLEGNEDCKKDAGKIDADKKARLEEERKRSKAYEALDLVDGYLKEHAKD

EWLVSGLAGVEEQIAGLLSKQNEILQQEATQETVTKALEQATKSFDDCQKLSGIRKQELK

GKSKQIQQGKDALNRLLGDRLLREYRTEKETLLREMAFLTKIAELKDHREKLEDGKPCPL

CGAIKHPFAEENIPAPDKTEQEIDALTRLISKAEDQEITIKKFEEAENLARKNLTEAEKL

ESVADNEKKAAEKILAEVRDCLVKLRSDFTERRQAITIKLQPLGIADIPETDISLLIGTL

KVRLKAWQAKVKKKVEIEKQIADIDSEVKRLDAVIEIQSTALAEKLERLGFLKKELATES

NERKALYGDKNPGDEERRLNKAISDAEGSEKQIRERHNELQQKWNTAKSHVESLKKRIEQ

REPELRRFEIEFSAALEPVEFSNEEQFLAAILPSEARAKLMATAKDLDERQTDLKARQKD

RETRLIAEMAREVADKPIEELETQLKEHEEVLKELRDIIAGLKHKLSENMAAKERIKEKQ

TAIEAQKKECRRWENLHELIGSADGKKYRNFAQGLTFEIMIGHANRQLQKMTDRYLLARD

NAQPLELNVVDNYQAGEIRSTKNLSGGESFIVSLSLALGLSHMASKNVRVDSLFLDEGFG

TLDEEALDTALETLASLQQDGKLIGVISHVPALKERISTQIQVTPQTGGRSQISGPGCNK

F

>tr|D6YV42|D6YV42_WADCW Uncharacterized protein OS=Waddlia chondrophila (strain ATCC VR-1470 / WSU 86-1044) OX=716544 GN=wcw_0635 PE=4 SV=1

MIISQAKLLGAAGKGVNRAARWLNFAEKEVKAASEMEETAAII

>tr|D6YVH6|D6YVH6_WADCW Uncharacterized protein OS=Waddlia chondrophila (strain ATCC VR-1470 / WSU 86-1044) OX=716544 GN=wcw_0770 PE=4 SV=1

MKTKQFLNVFLGLALFSMPLSADEVETEERIPVGDRAVVRAEQVKMYEDRLVQMEEADEE

ESQDEEANAKKIVKKDFLQATYVTTHEGAFHFPIAVSFLGDTVELEDGSIWKVCSNDAYK

TLNWLTSDMIIIVPNDSIFSSHDYKLVNLNTGAKVKVNLYLGPIYNGAYTHWIVAIDYLF

REIYLEDGSIWKMSSFDQSIVNTWLPNDTVIIGINDGFFSGTNPNILINVNMNNYSIGTC

LY

>tr|D6YX08|D6YX08_WADCW Uncharacterized protein OS=Waddlia chondrophila (strain ATCC VR-1470 / WSU 86-1044) OX=716544 GN=wcw_p0003 PE=4 SV=1

MGKIKTSSKHQTYLENLSKVDLDRVSGLHKAEPLKDLTDDRQISMAVFECLLNNDPEGAM

EVIEIYLEAMNKAKMRRKTKLPKSTMYSALKHRNPTIKTLAKIMYSSTH

>tr|D6YSW7|D6YSW7_WADCW Putative UDP-4-amino-4-deoxy-L-arabinose--oxoglutarate aminotransferase OS=Waddlia chondrophila (strain ATCC VR-1470 / WSU 86-1044) OX=716544 GN=wbpE PE=3 SV=1

MQFIDLKKQYQLYKKAIFQEIEKVLESGHFILGPQVKEIEQILADYVGIKHCLAVSSGTD

SLQIALMALNIGPGDEVITVPFTFISSAEVISLVGATPVFVDIEPDTYNIDIEKLEAAIT

PRTKAIIPVSLFGQMPDFTRINAIGEKHGIPVIEDAAQSFGATQNGKKSCSLTTISSTSF

FPAKPLGCYGDGGALFTNDDALAETMLAIHTHGSTVRNHHPLIGINGRFDTLQAAVLLAK

FPHFPDEVKAREQIGARYTELLGKCCQTPKIQPGNTHVYAQYTIRVPNREKVQEKLQEKG

IPTAVYYPVCLHEQPVYKILGYEKGSFPNAEKAANEVLSLPMHPWLTEEEQNHIVNTVKQ

VLMA

>tr|D6YV80|D6YV80_WADCW Uncharacterized protein OS=Waddlia chondrophila (strain ATCC VR-1470 / WSU 86-1044) OX=716544 GN=wcw_0673 PE=4 SV=1

MGKILVVTLTHELHQQCIESMSKNGIPFDGPLISDGEIHRFSRDSKRSQPDEWYVCYEGM

SIKDNPYLICCYGTWSGNQERFVYKSYENLPQSSPERHQLEEEYRKRKKHQESLLQKEQE

KRVARANEIWEESSNQDHCQGHSSYLEQKKIKPYDIKYRVEREGSPVIVIPLRNTEEELQ

GVQCIYEDGKKRIYGLKKGNFHVIGSLEGSSKVYVVEGYATGASVHEATKCPVIVAFDCG

NLKPVIASFRKKYPRAKITIAADNDDGNAQNPGKTKAEEAAKEYDCEVVLPAFTKEMQST

CRLTDFNDLHVHCGIDEVLKQLSSIKTSLQVLTANELLLMNIKPKKLIIDPWLPEKGLAM

IYAERGIGKTYLSLTIAYAVACGEPVCKWKIPEARKVLYIDGEMPGEALQERLRSISKMF

KNTPPEDSYFRIFSQDFQPDGIRDLGTFLGQEDVNELISDTDVIILDNLSTLIRSGKENE

AESWLLVQEWVLALRRQGKSVIFIHHAGKNGTSRGTSKREDVLDTVIKLKKPGNYAPEDG

ARFEIHFEKSRGFFGIDAEPFEARLKTNTDNSIEWSCSKVDGYEVEILAELYMDGMTKQR

DLAKEMGISVGKVNKLVKEAKENGLLK

>tr|D6YV36|D6YV36_WADCW Uncharacterized protein OS=Waddlia chondrophila (strain ATCC VR-1470 / WSU 86-1044) OX=716544 GN=wcw_0629 PE=4 SV=1

MIDFDNLPADITNHILLFREFLNISWDHVIYKIMDEHDWDDDGNFIYNWMQINWELLVER

ELLGKSFNLSQFSSTHLSDNILHPNVKPDFMVVGKSSRDLIDIRSGKEISKNQTLRLFTF

KTHTYESKGFAFGPPFEVAGLIDIETNELYHVLFEELSFWLEKFIS

>tr|D6YUZ1|D6YUZ1_WADCW Translation initiation factor IF-1 OS=Waddlia chondrophila (strain ATCC VR-1470 / WSU 86-1044) OX=716544 GN=infA PE=3 SV=1

MAKEDTIKLDGIVEELLPNMHFKVKLENGMNVTAHLCGKMRMRNIRVLAGDTVTVEMSPY

DLSKARIIYRQK

>tr|D6YX18|D6YX18_WADCW Phage/plasmid primase OS=Waddlia chondrophila (strain ATCC VR-1470 / WSU 86-1044) OX=716544 GN=wcw_p0013 PE=4 SV=1

MEVAVTLKNDKATDAGSRWLKNLTSNIKDIDMNNNNLKTEDNQEAKEEISLEKNAFKIKS

KGKSKLKFTPSIKKELKDVIGCQYVDILSAYVCPLGSKTEVSNLLEKNKINADLVEFHNP

LPKKSKKINGMETRLHILEKEVHKEDMELLVEAHKMKDEEGRHKSIQDFKDPPKEPGKDE

KSGDLRYRIELDLHERFKANDEKRKEAEELRRYVEQHEKEKTENEKVLERLNKNESGDAE

IFVELFEKKYLFDPTEGKNGAFYLWDGCQWTLDIHKERYKDFEKVSDTYLIATSDESIDE

SVSKELFKRSQQLRTSRRRSNVLETVSAYLSFKHSWDYCPNKLPCSNGIINLKTGDLETA

QRENYIKKVCPTSYEKNANCPKFLKFLDDITLGDKELSSFIGRVIGYALLGVPKEEKIFY

FYGNGRNGKGTLMHVIQHVLGALSKTFPSEMLLSQRNPPSSSSPNPELANLEGVRMAVFS

EINEGRKIDSAKVKNLSGRDIIPCRRLYSNVDLQITPTHTMILQTNYKPKAPSEDKALWS

RNILIPFKARFVKEPKDGENEREIKESLKDELLEEAKGILKWMVDGCLEYQEIGLKVPQS

VIDQTEGYRKENDGIGCFLEEMCFQDPAVSTQKSKMEAAIKNYCKANEMKEPTRNEISDY

LKIRFKEGRKSQGSYWKGIKIED

>tr|D6YVW5|D6YVW5_WADCW 50S ribosomal protein L35 OS=Waddlia chondrophila (strain ATCC VR-1470 / WSU 86-1044) OX=716544 GN=rpmI PE=3 SV=1

MPKLKTKKAVAARFKLTGKGKLLRQRPGLRHIMTKKTPKRKRQLAKPALVSDSQLKTYKR

LMCVS

>tr|D6YSH8|D6YSH8_WADCW Uncharacterized protein OS=Waddlia chondrophila (strain ATCC VR-1470 / WSU 86-1044) OX=716544 GN=wcw_1679 PE=4 SV=1

MIFFSGFLGGKKNRLPLYFRSSRFENVALNFGKINLQQQLL

>tr|D6YRW7|D6YRW7_WADCW Uncharacterized protein OS=Waddlia chondrophila (strain ATCC VR-1470 / WSU 86-1044) OX=716544 GN=wcw_1463 PE=4 SV=1

MANESGDTVINPYIAAARKKWQATKAILYTTGCLSKNDEIKQQFCSTKRLIDLENPVIPC

M

>tr|D6YVK4|D6YVK4_WADCW Putative rRNA methylase OS=Waddlia chondrophila (strain ATCC VR-1470 / WSU 86-1044) OX=716544 GN=wcw_0798 PE=4 SV=1

MYEQYPLFHSHIDLAHKHWKSLVKPGDIVIDATCGNGHDTLVLAQLALTVESGKLYACDL

QKDAINSTKQSLLEKLEKKIVERIFFVHGCHSSFPDEIQPNSVRLAAYNLGYLPGGDKAK

TTQTETTLQSIQKALEAVQDGGMVSITCYPGHTEGKREEKHILEFASKLDPKRWSLCHHR

WLNRSKAPSLLLIQKRMKSPLDSI

>tr|D6YT25|D6YT25_WADCW Uncharacterized protein OS=Waddlia chondrophila (strain ATCC VR-1470 / WSU 86-1044) OX=716544 GN=wcw_1882 PE=4 SV=1

MLRLILSYFLSLLFLLGMASTGHASILLSNEEAASLEIFFRHVLKEHEVGYVIEGVKPLC

ILGIAEQGDVYAGFPGHKTSLILKKGLRVWNAKLSQYNISSPIRIKAYDHPDSKASTYKH

LLFIHKKLFLNTVDQNLSLFRYVLGPHLTSHSFYDTLINSSKDFSETFGGHDNVLVGIAL

GYGTENSLIVSRQEALQHNSKNLNPSIGFKSLEEEENYLSELVTLPVDELTRVKPCFIYG

RRKNDPTSDAFDKKLIEAQKIIQRKLDHPDFLKNITFLLTGQDLVIQSNPITLTSSPHDA

IENIVAKNLLISLYFHGFSSEDALNLIKALGNPGKETKEPFFQIKAHDLSNFYMRSSTFD

LSKLIIAMERFRENPLELSPSEEAYLDDFYLNLYSAIPGTDKS

>tr|D6YWI6|D6YWI6_WADCW Putative ABC transporter, permease protein OS=Waddlia chondrophila (strain ATCC VR-1470 / WSU 86-1044) OX=716544 GN=wcw_1140 PE=4 SV=1

MFELSIAFKYLVPRWRQLSVSIISLISILVIALVVWLIVVFFSVTHGLERGWVDKLIALT

APVRITPTEDYYNSYYYLIDSISQESGYTVQTIGQKLGTRKTDPYDPDFDQEIPYTWTAP

DLNQDGEVKDLVKLAFKSINSIKGVSAKEYEMTITNLKLNMIRSEPLQLSGNPDSDNDQR

ALSQAIYLSSFDPSNPSLPKIMFPVASADIDNLIRTVENEQAGSPEQMRSSMKEILATVK

PLKTRTPAHGQLIPQKAIPQNCRLNVCVLNSANTIHALYLPQSAEELDPSYGTPAILEKE

NDTITVAMHEGHSIPLDSWVPVYLLGNAQGEIALDPSSLETAEFARDIKFNTQFAVQGVN

LSTTLGIDEILVDQFSIENGSRSFWVYDQTIPESLPSSPQWGEAILLPKSWRESGALIGD

RGYLSYQTPTASSLQEQRIPVYVAGFFDPGILPMGGRILLANDSIVSIIRSGNLMSDSSL

SNGINVRIPDLDDAEPVKAEIEKSFKEAGIDKYWKVQTYREFEFTKDFLQQLRSERNLFT

LISMVIIIVACSNIVSMLIILVNDKKMEIGILRSMGATSKSIAAIFGLCGIVMGLVGSLI

GIALALLTLKNLQMLIDFISRVQGFEMFNPAFFGDTLPNQVSLQALTFVLTSTAMISLIA

GIVPAIKASLLRPSAILRSE

>tr|D6YU61|D6YU61_WADCW 2,3-bisphosphoglycerate-dependent phosphoglycerate mutase OS=Waddlia chondrophila (strain ATCC VR-1470 / WSU 86-1044) OX=716544 GN=pgmA PE=3 SV=1

MSRLILMRHGESEWNKLNQFTGWVDVSLSKKGIEEAIEAGKEIKDIPIDVIFMSTLIRSQ

LTAMLAMSQHSEGKVPVVMHKTDEKLKEWGKIYSDEAKEKTIPCFVSWEINERMYGELQG

YNKKKTAEKFGADQVKVWRRSYSTPPPSGESLQMTAERTIPYFENTIVPFLREGQNVLVS

AHGNSLRSIMMDLDALSEEEVVSLEIPTGKPIIYSYENGNFIRK

>tr|D6YW19|D6YW19_WADCW Uncharacterized protein OS=Waddlia chondrophila (strain ATCC VR-1470 / WSU 86-1044) OX=716544 GN=wcw_0970 PE=4 SV=1

MEESIEEKIKRLKSELAVAKQAHQLWDILIEKREKLEEKIAFLRAEIEDEKNAQYQQAQK

AYEEIKNRGNKKEVLDTIKQSKQTIDEKLDEYEDFSDDIITYLQNQLVSNILKKHPGQES

SYRNLDNQFHQSMDLKDKLQALSTLTRDIDTLINKIVEERKRAGPFRLLQFFLGVSPYYE

ISQNVQGIKLLCGKALNLLHDIEEKMQDNHQAIECFEQLLGIFVKLQSFAQQRWSYGKID

KRLMPLKGTLTPLADQMEMFKKEAEKNASSQEEMLNLWIDQHS

>tr|D6YSH0|D6YSH0_WADCW ABC-type transporter, permease subunit OS=Waddlia chondrophila (strain ATCC VR-1470 / WSU 86-1044) OX=716544 GN=wcw_1671 PE=3 SV=1

MCLSAAWVGVIAYLKKQSLLGEALSHASYPGVIFGALLGSLALNGDEQWFLVPLMGMVGA

FATSLAALYCIHYLERSHKIKSDSAMCFILSAFVGVGITLASRVQFSHTSLYKQSINYLY

GQAATMTDIHIALYGILSVLVISLILLLYKELKVLTFDRDYAKSLGLNVKALDAIVFFLL

TIAIVVGIRSVGVVLMSAMLIAPAVAARQYTHRLIYLLLISGLFGIVSGLLGNILSVEVT

NHLQTLFPKDRLSIPTGPMIVLVATVICAISLLFAPERGLVIRLARIAYFRYLCLVENVM

KTMWRMGKGLWYSVNDIRCYQTVSYLSLRFVFYRLSKQGWVEQRGNRYRLTTDGIHRAAH

IVRLHRLWEVYLADYLGVGSERVHRSAEEMEHILTPELEKELTKLLEDPKIDPHRQPIPP

STVKEA

>tr|D6YSB5|D6YSB5_WADCW Uncharacterized protein OS=Waddlia chondrophila (strain ATCC VR-1470 / WSU 86-1044) OX=716544 GN=wcw_1615 PE=4 SV=1

MALEKIDKIKKVSTKLEPQTRIHEPNKDYFDALMQQQRVTAEKVEASSKDASEKAGATLF

DEVQNLNRRPDIATRSSPNELVAQAEDVIAQIDTLKTKLETPELNIKSSVQTLLRNKLNH

IDENLKVALDKTGTEVAKPERSDGMSKPIDRFLGLLTHSQNQLETLASDVKAMAVENEMS

PASMLLIQIKVAKVQQEIELFTSMLNKALESTKTIMNVQV

>tr|D6YVL1|D6YVL1_WADCW 50S ribosomal protein L34 OS=Waddlia chondrophila (strain ATCC VR-1470 / WSU 86-1044) OX=716544 GN=rpmH PE=3 SV=1

MVKRTYQPSKRRRKSEHGFRKRMETASGRKIINRRRRAGRKALTRV

>tr|D6YW36|D6YW36_WADCW Putative mechanosensitive ion channel OS=Waddlia chondrophila (strain ATCC VR-1470 / WSU 86-1044) OX=716544 GN=wcw_0987 PE=3 SV=1

MDRKRFFLFFISILFLLTSALIADQEKEKEPSTPSPLELKQNWWEFFDVSDETLKSRLDT

FKKENKALLSSLSDKEHEEIHRKMDQVFLMLDLYAKKKEEVQPSSAPQLQLLEFYTLDQL

VEVYERLQRSEMEIGILQSKIKIQQSRINRLQNTLDRSILLYQNLSPATYEKLKQGVLLI

SQRAEHATLDLELQRDKKALTSFLERRDLYRNELVAAKKRLKIDAQTLDELKKKSEKAKS

SYFEAEEKFYQLEKQSRLVDKDKQESKFTCCIQDSQILSQAISLENFKIKLLINEMKTTL

AKLALNHREIDSDDIREFLSDWKRRLGSIEEQRDFWDTEIKNYQAQVSQMTAQSMQEGSK

GQVDQEDLIGDIHFELDRSLAELELLKLHIENGEFLERLVGEQLVEKKSFMQTWLISLKN

SWSKFKNVVDHWIHVTLFHVNEQPVTLMTFISALLIFLGGIVFSHYLRKFLVKRKIVQRK

FSYSTEYIVLRVIHYAIVILAFLVALSFIGLNFTNLAIIAGALGVGIGFGLQTIVSNVSS

GFMLLLKKYLKVGDIIELSDKQLGTITAVNLQNTIIRTFDGAEIMIPNSQLSSQRLTNWT

MKDNAKRLKIPFGVAYGTDKNLVREAVVDSIKKLSFVYSDDFRYHDPQVWLMGFGESSVN

FELVAWINLNVPVPYETASSALFWELDTTLKNKGIEMPFSQRDLYIKSFPSGILSSID

>tr|D6YWM0|D6YWM0_WADCW FAD:protein FMN transferase OS=Waddlia chondrophila (strain ATCC VR-1470 / WSU 86-1044) OX=716544 GN=apbE PE=3 SV=1

MYKHLIFFLAAILLFSCSQSNENSVTHFNGIKMTVPYRITIGSPLSSRQKTEVQNIIEQT

FDEINNIYNKWNPHSEISALNRAEANKEHPLSPALYQFLVQVEELVNQTEGRFDPTIEPM

QKLWKNAFGKGVFPSQSEIDTLLPSVGWHHIHLTANGIWKDDSRSSLDLGGIAKGYAIDL

IAQRLEKAGYRNLYVEWGGDIAVKGQHPEGRPWRVLVTKWGAPDDTQTVAELKNLAIASS

GDYLQNWSIEGNVYTHIFNPCTGEPMKITRQSICSVTVVASNCLLADTIATTAMLFGTKQ

EAEEWLEHLKDRYPELQYWVYKR

>tr|D6YV62|D6YV62_WADCW Catalase OS=Waddlia chondrophila (strain ATCC VR-1470 / WSU 86-1044) OX=716544 GN=katA PE=3 SV=1

MKRDRPATTNDAGAFVPSDEYSLTVGPDGPILLQDHYLIEQMANFNREKIPERQPHAKGS

GAFGYFEVTQDVSAYTKASVFQPGQKTDTLIRFSTVAGEQGSPDTWRDPRGFALKFYTEE

GIYDIVGNNTPIFFIRDPMKFQHFIRSQKRRADSGLRDHDMQWDFWTLSPESAHQVTWLM

GDRGIPKTYRHMNGYSSHAYMWANAKGKRFFIKYHFKTDQGIDFLTQEEADRLAGVDGDF

HRRDLFEAIKGGDYPTWTLKVQIMPFEEAATYRINPFDLTKVWPHGDYPLHEVGRLTLNR

NPVDFHTEIEQAAFEPSNLVPGVGPSPDKMLLARLFSYADAHRARLGVNYKQIPVNQPKV

PVHSYSKDGAMRIQNVSDPVYAPNSKGGPKADGQHYPQVEVWNASGEFVHAAYTKRRDDD

DWSQAGDLVRHVMDDAQRDRLVSNVAGHIKQGVSEPVLQRVFEYWCNIDKEIGERIKKAA

GCSS

>tr|D6YW57|D6YW57_WADCW Putative low molecular weight protein-tyrosine-phosphatase OS=Waddlia chondrophila (strain ATCC VR-1470 / WSU 86-1044) OX=716544 GN=wcw_1009 PE=3 SV=1

MVSILFVCLGNICRSPCAEGVLKHFAKEDGLDLHVESCGLGDWHEGQLPDERMRKTAQAR

GIVLNSRAKGFRPEFFDVFDWILAADKSVLDELHKKTDSPTHKAKIHLMTKFSKSHLGKD

VPDPYYHEIAQFEYAMDIIEDACRGISERLKKKS

>tr|D6YVN9|D6YVN9_WADCW Putative carboxypeptidase G2 OS=Waddlia chondrophila (strain ATCC VR-1470 / WSU 86-1044) OX=716544 GN=wcw_0834 PE=4 SV=1

MVDQYFTYLEWLDSEKESMTEKLIDWVLIHSGSDHLEGLSLMNRTLIDAFTRLEGKIEEI

GLPPRKLMNKKGKIIQEPLGKALSIVKRPEANMQILLGGHMDIAFSKNHVLKKCSIKKKD

TLVGRGSVDMKGGLMVLLYALLSLERSPFANKIGWQVFITPDEEIGSPGSQSYWKQFASG

KKCALLYEPSFPDGNLASARKGSGNFTISITGKSAHAGRAFQEGENAILSAARIALSVEG

LNDLSNELTVNIGFIHGGGPVNIVPDSTLLKLNIRCQTLQEMQTALSKIKEIIKIENNRK

NLQIVIFQDSLRPPKIFDKRTRNLFESLKLSAKHLGIHLNWHTSGGVCDGNVLANQGLLT

IDTLGAVGGGLHTEEEYVKLQSIIDRAKLSARFLMQIAAGEIQL

>tr|D6YUR5|D6YUR5_WADCW Probable membrane transporter protein OS=Waddlia chondrophila (strain ATCC VR-1470 / WSU 86-1044) OX=716544 GN=wcw_0505 PE=3 SV=1

MNNAIKLIVATACLFVFFQILTHYRQMKKEPFSFKKTCALLFTGFISNISDTIGLGSFAV

VVALNNRFRTFDDKIVPGTLNAQSVLPSMLQSILFLNFVEVDLYLLLLFVASACFGGFLS

GYLVSKLDKKAIRQLMCAGFVGVVMLILSQKGGLLPNAGFATSLPPFKMAVGAVAMVFAG

MLPAIGAGIYVPIQTILFLLGLSPLAAFPIMTTAGAIVQTATASAFVMRGEFAVKESLFL

SFSGILGVAVAVPIISFVNLSSLHWLLLTIAAYNAFSLWKLINEENVKPARI

>tr|D6YWZ3|D6YWZ3_WADCW 3-phosphoshikimate 1-carboxyvinyltransferase OS=Waddlia chondrophila (strain ATCC VR-1470 / WSU 86-1044) OX=716544 GN=aroA PE=3 SV=1

MRLNAPPSKAHTLRALFLGALAQGKTVLQSPLLADDQKVAIQALKQLGADISIQRDSATI

QGTGGNRVQDSGSLFVGNSGVTCRFLSAIAPLLCKQSVAIDGDLAMRKRPLTQLLIALEP

LGIHSDSETGCPPLTLTCKRFTGGATSVAGNISSQYLSAILLAAPFAENDIVVSIDGELK

SGPYVEITLDMMRRFGAEVEHEGSTYRVTAGKRYKAVAPYEIEGDYSNASYFLAQAAITH

TRITIDRLMPNSLQGDRKILDLLKQFGCNVSREGSSVTVEGRPLSSICVEMSDTPDLVPT

VAVIAAFAKGTTKINGVGHLRYKETDRLKAIVSELKKMGIKAFSEEETLWIEGGTPQAAE

IDTYNDHRIAMAFSVAKRAIPKIVIRCPECVNKSFPDFFDLWNLQ

>tr|D6YST8|D6YST8_WADCW Peptidylprolyl isomerase OS=Waddlia chondrophila (strain ATCC VR-1470 / WSU 86-1044) OX=716544 GN=wcw_1794 PE=4 SV=1

MKNALCVSRLEELEDNVFSEETPPFKNLREVLNFPHLNVLDYICQRNHAKKARLLQPNLG

YSTIQEEYQELIKNIRLSPEPLRSEHPRFIFGYFKDDPASLVLIEKLKESQKEIQKQLQT

ESFLKDCVRAFAGIDIIIDQDDSLAKALTAISKDQWNHLVSKRLCYTLMEEGYSLDDQQA

FLEGFRETNATRELLDFRCAWPHFSENLQRALNNLKEANLFFSRLRNQAHLKELIPNSLF

IESSENEADQKNDRASKVLLDYVVYNPKEEVLREVKGEAVLLSDTIPGFSQGVRQMRVGE

TARLYIHPSLAYGVETVSEQGIYLIADVTLRKVEEFLKDSAPLPIPKNLSYFLDPDWLSQ

SMEKRRLAMKDRGKELRCFFKKSPLLNMEEIESQMRMHLSDVSREVRITEREKELLNRLY

WSICTKN

>tr|D6YTI8|D6YTI8_WADCW NAD(P) transhydrogenase subunit beta OS=Waddlia chondrophila (strain ATCC VR-1470 / WSU 86-1044) OX=716544 GN=pntB PE=3 SV=1

MNVLFDFAYIFASILFIIGLKMLSSAKTARKGNAISAVGMLVAITATLLYSGLSYTWIAI

GLATGAAIGALTAQSVQMTAMPEMVALFNGFGGIASLLVGWAEFHSKPSEDPFTRFAIFL

TVLIGGVTFTGSMIAYAKLAKKMDGKPTLFKGQQSVNALLMLIAVVCGILFSFSIGNSYT

LFLAFTALSLLLGILTVIPIGGADMPVVIALLNSYSGLAASMAGFIIYNTVLIVAGSLVG

ASGLILTNIMCKAMNRSLLNVLFSGFGKTASSQSSQGIKGEAKPINASDAYFLLEAARSV

IFVPGYGMAVAQAQHIVKELADMLEENGTEVQYAIHPVAGRMPGHMNVLLAEANVPYELL

VEPKDVNPTMGIVDVAVVIGANDVVNPAAKEDPSSTLYGMPVIDVEMAKTCLVLKRGLNP

GFAGMENALFFKENTRMLFGDAKQSLSSLVSEFKK

>tr|D6YWN2|D6YWN2_WADCW Acyl carrier protein OS=Waddlia chondrophila (strain ATCC VR-1470 / WSU 86-1044) OX=716544 GN=acpP PE=3 SV=1

MATEQEVIDIVVEQLGVDKGDVSLEKSFVEDLNADSLDLTELIMTFEERFGIEISEEEAE

KLKTVGDVVNYLEKTKS

>tr|D6YSD2|D6YSD2_WADCW Uncharacterized protein OS=Waddlia chondrophila (strain ATCC VR-1470 / WSU 86-1044) OX=716544 GN=wcw_1632 PE=4 SV=1

MTGDMNVNQQGISQTEFHHLLDHLELSEEKDLIDLMNDSTISKSLKEAVMMYYAIGIPFL

ERPEIGENGEFSWSVSNESVQSAVEAGFAKIGSDIWDRYAEYLEDQKKRIAEYLDSPQYR

EKVERQSPAYLAYIERNTPIDTQSQARNATGYQEWLSTLPPAARDIEINWTDEKIGHSKD

LWNNLIDATSHFLSDYKDEMSDAVPFMAASFVISSTFIGDYMNIVDVASTEMVTVNPVQD

AAKNVLALVEPAFHEQVTLAINFFAVGLVAYSNAEAIGNQQQGGQEAASKDAVLAFANSV

IDKVKSNEINYFLMALLVNSAEKGQLSPQDVQHLTRVVKAAMLSVALAALYKFEAGEISE

KEFRDLLAGKMEPRTEEERQLVLLLNSVYTEGMAANDGQYEGQWRSVMDSLVSFILSKPS

VEDLINPTRVWAQVGTFLRNPNPRG

>tr|D6YVP9|D6YVP9_WADCW 4-alpha-glucanotransferase OS=Waddlia chondrophila (strain ATCC VR-1470 / WSU 86-1044) OX=716544 GN=wcw_0844 PE=3 SV=1

MGNAKRYGISIPIHALHSKTTYGIGEFLELIKLFPWIKSMGFSAVQLHPLCDTRGMANPH

LPYSPFAFNPLFLSLTEIPHSNDKEELYLQKTINYTSAAKFKETLLIKFYYDHGETVFES

DAFIKFSEENPWLDNYVGLKNQDPRFYQFIQFLCHQQMKQVKTEAEKHGILLISEIPTQL

QAEAPDVKYHPSWFTIDNDKAGCNWSEMERDGYSWWKERFFHAAAYFDACKFDRPIDSNG

FFKEISTGLPLLLILNELQTTNLDCNSNLCPTVIYPNREQCADHSLTILDKPSKFVSREQ

CKQLIKHSHSTSSLLHMISLTTYLSLIPNLNWIASTEEDADQWSLSIKPSVEELILDPSL

REVMQECLVS

>tr|D6YUR8|D6YUR8_WADCW Uncharacterized protein OS=Waddlia chondrophila (strain ATCC VR-1470 / WSU 86-1044) OX=716544 GN=wcw_0508 PE=4 SV=1

MRIFHHYGIPTTEQRDDETLVEVGGFKFYSTPFEGNRWHIQWHRFPEGHGLPELVTQVPH

LAFQVDDLDREIEGANILFGPYSPLEGYRVAMIEEQGVPIELVETKLSDEELSELERKEF

NKGYYP

>tr|D6YTS8|D6YTS8_WADCW Uncharacterized protein OS=Waddlia chondrophila (strain ATCC VR-1470 / WSU 86-1044) OX=716544 GN=wcw_0164 PE=4 SV=1

MPYSRESPSPEYLRLIKEYRSLHLKGSETPPIPKKSIFAGMSTFKAAEKIRRCLIETPCL

TLLNYGCGKGQQYQKIFRSSEKPDELSTLKDYWRLKSIACYDPAYPPFEKMPKGTFDAVI

CIDVLEHVPREDLEWILQEIFSKANRLVYLAISCFPARKTLQSGENAHCIIEPPKWWHEE

VEKTAAAFPKIKRYIVCESS

>tr|D6YRM7|D6YRM7_WADCW Uncharacterized protein OS=Waddlia chondrophila (strain ATCC VR-1470 / WSU 86-1044) OX=716544 GN=wcw_1371 PE=4 SV=1

MRLSQPGEADGGIRDVRSPLREVFNAICDSSKDVNQTSYQKFNFKVKE

>tr|D6YSE3|D6YSE3_WADCW Peptidyl-prolyl cis-trans isomerase OS=Waddlia chondrophila (strain ATCC VR-1470 / WSU 86-1044) OX=716544 GN=mip5 PE=3 SV=1

MQTQASKYWRSCCTAATVSLILFSGCEKKDEEKHSVNEDEIKKISETMGHYVIENLNAQS

LTLDTESFIKGIEGAKAGQEPPLSKQKFLELLANYRKKAFEMKSSENLKMAEDYLGDNSN

SPNIVVLEKGKLHYQRLKPGNGETVSETSTPLIQYEGRLIDGQVFDSTGKRGKPAELPLK

STIPGFRKGIVGMKEGEKRRLFIHPDLGYGENSRLPPNALLIFDVEVIKADAAKKEAKAP

QKETSMSWLKRWAVPN

>tr|D6YVU1|D6YVU1_WADCW Conserved putative membrane protein OS=Waddlia chondrophila (strain ATCC VR-1470 / WSU 86-1044) OX=716544 GN=wcw_0890 PE=4 SV=1

MKDVLTFIFHNFFLVALGVACIWALIRQQGLLSYFMLLPIGLGGIWAFYLYAFHTELGAK

LAGWNVCNFQYSAAAAFLGLGINGIIAFKKGRDFRLSVILFASALFWGDLIAHFYQAYYI

GESASSNTAFIMYGDFLIPSALWIAFTLDL

>tr|D6YVH9|D6YVH9_WADCW Putative secreted protein OS=Waddlia chondrophila (strain ATCC VR-1470 / WSU 86-1044) OX=716544 GN=wcw_0773 PE=4 SV=1

MRAALIFFFSLLACAFVNASDQRLILQLSSGINAERGNAAYLLGMSKDPGVVGLLLRQLQ

EETVRENKLVIIESLKKLDSEEGYYGLSTFYQNESDSVVRRKVIQALGESGDRQYVPLIA

KNLEQAEAFSALSRIDHPEAAKVLLTAYGSARSRSQKERILAAIAAQGRIESVKPLISYG

QQAKDPLERVWIAETLADLGEGEVQDAIYQWFQDSNDSISKRRLVKSLKSIGSSKMVEKL

SKDLRTNDLSLQIEIVEVMVFIDKQAAAPYLRSYYEESMERQDPNMSKLEAVKTARLYNL

LRQNG

>tr|D6YSR3|D6YSR3_WADCW Coproporphyrinogen III oxidase OS=Waddlia chondrophila (strain ATCC VR-1470 / WSU 86-1044) OX=716544 GN=hemF PE=4 SV=1

MICTASAHKHQEIIAFLKSLREEIIQEFESLETADRFKRTFWNYDKGSGGGEMSVLRGEV

FEKAAVNWSGVSGENFPMEDSAGPFFATGVSLITHMSNPHAPTVHMNIRYIETEQGSWFG

GGYDLTPMGFPYEEDTRHFHSVAQNALTPFGKELYPQFMQQAKEYFYIPHRQKERGVGGI

FFDHFNSGDPHKDLQMWKTIGSTFIDSIMPIYHRRCSIPYSQEEKETQLKLRAHYVEFNL

VYDRGTKFGFHSGGNPEAILCSMPPVAKW

>tr|D6YSL0|D6YSL0_WADCW Uncharacterized protein OS=Waddlia chondrophila (strain ATCC VR-1470 / WSU 86-1044) OX=716544 GN=wcw_1711 PE=4 SV=1

MRHCFTLYNYLELLIIDIIHIFRKYKESKLMNKTKTSSNQTKEPIDWVDQIQQHPAFEWM

MQNLKYIPYLFIALLIMIIAGYQLISGSAAKTEANYQLAEGYWAKIQRSIGQEAIDAKQE

DALLQLCKIVDKHPELRAKYDGMIAQLLIAKGLSEEASVYTERVEKRTASTQNPVFKEFS

NISLLIAQGNHQEALQRSLSLKETLLKKEEPPLLLPYTLIRIAMLYQGMGEKNLEALAWD

EWQQYAQEKSLQKPDTQTLKTISALFSEGDFSLDQYIAERSLEKN

>tr|D6YRX9|D6YRX9_WADCW Citrate synthase OS=Waddlia chondrophila (strain ATCC VR-1470 / WSU 86-1044) OX=716544 GN=prpC PE=3 SV=1

MVETKTKQKSGGLAGIIAGDSSICLCGAKEESLLYRGYPIEELAQNGGFEETAWLLLRGK

LPNDKELTGYKQKLRALRELPDTLKKILEQLPKQCNMMDVMRTGCSALGNIEPETGKSDL

FQVADRLIACFGSMLLYWYHFHQSGKRIPLETGEDTLAGHILHLILQKEPSDLHRKCMDC

SLILYAEHEFNASTFTVRTIASTLSDFYSCICGGIGALRGPLHGGANELALSLIQQFDSP

KAAEQGIREALSKKERIMGFGHRVYTTKDPRSPIIKGWAYKLGQETEMAQWFPVAECIEK

MMWAEKRLFPNLDFYSALAYHFMGVPTPMFTPLFVMSRISGWSAHLLEQRANNKLIRPLS

NYIGPEKKTWTPLLNR

>tr|D6YT28|D6YT28_WADCW Integrase OS=Waddlia chondrophila (strain ATCC VR-1470 / WSU 86-1044) OX=716544 GN=wcw_1885 PE=4 SV=1

MVTPRAKKACVQILVKQHKRSERRACQLVGVHRSVVRYSSRKQDETALITKIKQIAYEKR

RFGYRRIHMILKREGVKVNHKKVYRIYRACGLKVLKRGGRKRAIGSRRAQDKPFARNQQW

ALDFVHDALANGRRIRLLTVIDTYTRECLRIFVDTSINGRKVTEILSEIMVANGKPKVVL

SDNGTEFTSNTVLKWSSDQGIDWQYIEPGKPYQNGNIESFNGVNNGLKLTHFF

>tr|D6YVM7|D6YVM7_WADCW Uncharacterized protein OS=Waddlia chondrophila (strain ATCC VR-1470 / WSU 86-1044) OX=716544 GN=wcw_0822 PE=4 SV=1

MGAEKTSSVVEESKIFTQESSSERPLSEIGVDTHLPSQVKPTADKADTVQQEKFFSKSEK

RQGRILDQRIVKEALEGGADVENLASLKKTDHVVQETQIFKQSSEESPPLSEKKVDPNPK

PELRRMPHQFFDKKVIGGIRAQSHENGGDVKDLSEPPKELKTAQRPSDENIKTISNALAS

LNKKTKLGVSLGKDGHVNISTKKRGRIGKAGKSQETKESMNTMLDVMEEIMRNGPENAKN

EVFKSLRSINSKSWSRDVLKQNPQMKARFQAIKNNFPHLTIKPSSSDLNTFSEMVGKVSG

DLESKDYKGLSDTLTEHSGTLKTILENSGMDPKKTVDISSRGRADLLGIPMEEFFGLVPS

EHSGSAIIQDIIDHTQFLKDNPEEGYALTSGQELAAQRQGDREISSFQIFGRVARTTGGG

PSVTTTMAKQLDRCVDGFCAKFIVDPERADKEGSDDLTPLQMTRNDMIQSRLEEISDFKD

FMEDRSNINKKIMKILRADTIDPKGLEDVIKDIKAFEEKYKPLAEKHQKELQKIRGEIPK

NIGFFTEKENIQDKPEYAEMLREYASKYTN

>tr|D6YVU2|D6YVU2_WADCW Uncharacterized protein OS=Waddlia chondrophila (strain ATCC VR-1470 / WSU 86-1044) OX=716544 GN=wcw_0891 PE=4 SV=1

MKIIITFFLLFAAPLIGTQSKPPSISKEIWSEVSPYLLPENHPLKPCLDLIFSTNCIANG

ISLYKAGFRYDSYNNPMKVIVAKHPKLEGYIIKLFLDNQPVGSEWPHWIKRIEGAKLIKK

IIEQKEYTASFKVPRKWIYFIPCKHNILSGRHFILIAEEMDIVQHRKTCSQWKNQATYEQ

IYMFWDLLETCGLSDSIYIQNIPYCKDGKYTFLDTEYYLDWPIEYHKFNKYLKKGKLKLW

KQLTNQE

>tr|D6YX27|D6YX27_WADCW mRNA interferase OS=Waddlia chondrophila (strain ATCC VR-1470 / WSU 86-1044) OX=716544 GN=mazF PE=3 SV=1

MALKKNNPLQGEVWLFDPDPVKGNEIGKKVRPALVVSNNLMNKGVSGLIIIVPITSKDKK

IPSHIRIEPPEGGVNLPSFAVCEQVRSISKSRLVKRLGKVQSATVLKEVGSWLNDLLWID

I

>tr|D6YW46|D6YW46_WADCW Oxoglutarate dehydrogenase (succinyl-transferring) OS=Waddlia chondrophila (strain ATCC VR-1470 / WSU 86-1044) OX=716544 GN=sucA PE=4 SV=1

MGFERLTLSGYGNLDLIEELYDRYQQDPSSVDPTWRAAFTSMISDEVPAAIVIDQSAPAD

LRIDRLIRSYRTWGHLKADINPIRTTPIKPPWQLDLGVIGFKESELKTTFPTNGLLHKPE

APLQEMIDVLEAIYCSKIGVEYMGIQNLEMQHWIQKKIEPNKFQIALTIDQKRSILQHLN

KSELFEVFLHTKYVGQKRFSLEGGETLIPILAALIDTGSGLDIEEFVIGMAHRGRLNVLS

NILKKSYSLIFSEFEEGYIPLSFEGSGDVKYHKGFSSKIDLNGKKIDIRLTPNPSHLEAV

NPVVLGQVRAYQDSIGDEKREHSLPILIHGDAAIAGQGIIYETLQLYGLEGYATGGSIHL

VINNQIGFTTTPENGRSTGYCTDIARAFDAPVFHVNAEDPEGCVAAIHLAIELRQKFQCD

VFIDLNCYRKYGHNEGDEPAFTQPHEYDLIRSKKSIREIYRDDLISQGVLERKMAEALEV

EFKEALQEALTGLRLPEKKFNQDEPPKRHQKKKGPFAPVQTGVDLKTLKFVAEKICTIPK

NVTVHRKLKKLIKERMEMVEGKKGVDWGMGETLAYATLLNENRTIRLSGQDSCRGTFSHR

HAVWMDQKEEKEYCPFDRLNGNFYVYNSPLSEYAVLGFEFGYSFARPDALVLWEAQFGDF

CNGAQIIIDQFISTSEMKWGQVNSVTLLLPHGYEGQGPEHSSARMERFLSLCGDWNMQIV

NPTLPVQMFHLLRRQLHKPMEKPLVIFTPKGLLRHPECVNDIGDFTKGHFQEVLDDPLKP

KNIETLVLCSGRMFYDLNAERRKRKNEKMAILRIEQLYPLYKEKLTELIESYKGFKTCYW

VQEEPENMGAWDFIRPQLLSLLPKGVQLEYIGRSQSASPAVGSFALHQKQHKKIIEALFG

KEEA

>tr|D6YRH8|D6YRH8_WADCW Multiple resistance and pH homeostasis system, subunit D OS=Waddlia chondrophila (strain ATCC VR-1470 / WSU 86-1044) OX=716544 GN=mrpD PE=4 SV=1

MILPVLPLILPLIAAIVLMATLGNAKAQKWISTVAAFSMLASSIYLFFYVYRLGIVAFQL

GGWEAPFGISIVLDRLSMIMLTATACVAVSVILYACGSLDEKREKSGFYPLVFCLLTGVN

GAFITGDIFNLYVWYEVMLTASFVLITLGKEKDQLSAGIKYLTINFVASIFFVSAIGILY

GMVGSLNMADIAKRLMDAGEVPYISGVCMLFFIAFSIKAALFPFFFWLPASYHTPPIVIT

ALFSGTLTKVAIYTLIRFQSLYFFSETAFWKPFFLVTAFLTMIVGVLMAASQYDTRKILS

FHIISQVGYMVMGMAISTIGSLAGAIYFVAHNIFSKTSLFFVAGLVNQRSHSYEIRDLGG

LYSAAPALAILFSIPALALAGIPPLPGFFGKFLLVRSSLEEGDYLIAAAAILVSLATLFS

MAKIWNEVYWKKKTYHAESGKISRRAVAATSFLMACVVLMGVFAGPLIALCQAAAEQILD

PQQYIQAVMERG

>tr|D6YWC6|D6YWC6_WADCW Peptidase_S9 domain-containing protein OS=Waddlia chondrophila (strain ATCC VR-1470 / WSU 86-1044) OX=716544 GN=wcw_1080 PE=4 SV=1

MQRYEERELVEFENQGIKIFGVLHKPLAQTKAPAVLFCHGLAGHRIGKHRMYVALSECLS

RVGIASFRFDFRGSGDSEGEFGEMTLEGEVSDAVKALEFLTIQEKIDPNRIGIFGRSFGG

AISIFAAQKFGNVKSIALWSSVFDAEQWEKQWEMLETGQIDEKTRHELMRINGQLPSLHF

YKELFNMDLKKELRALQNIPMQLIHGERDPRVGIEHSEKYANLRKDAPAQTEFIKLEHSD

HDFTYPEERVHAINMTCQWFAKTL

>tr|D6YSQ9|D6YSQ9_WADCW Uncharacterized protein OS=Waddlia chondrophila (strain ATCC VR-1470 / WSU 86-1044) OX=716544 GN=wcw_1763 PE=4 SV=1

MIRAALFTAFFSSALFSLQADDNFCRKCQVLREYHEKNPSKYKYYDDYLKDLEEKGEDAV

SPRLEEMPEEVRRIVDPDKKSD

>tr|D6YS95|D6YS95_WADCW Cell shape-determining protein MreB OS=Waddlia chondrophila (strain ATCC VR-1470 / WSU 86-1044) OX=716544 GN=mreB PE=3 SV=1

MNKKTETGLRESMNKMRTSLGNFKNFRGVFSNDIGIDLGTANTLVYVRGKGIVLAEPSVV

AVDSNTNEVLAVGHKAKAMLGRTPRKIHAVRPMKDGVIADFEVAEGMLKALIKRVTPSRS

LFRPKILIAVPSGITGVEKRAVEDSALHAGAQEVILIEEPMAAAIGVDLPVHEPSANMII

DIGGGTTEIAIISLGGIVESRSLRIAGDEFDECIINYMRRTYNLMIGPRTAEEIKMTIGS

AYPLGENELEMEVRGRDQVAGLPVTKRINSVEIRECLSEPIQQIIESVKLTLEKCPPELA

ADLVERGMVVAGGGALIKGLDKYLIKETGLPVILAQNPLLAVCLGTGKALEYLDKFKKRK

ATA

>tr|D6YTU4|D6YTU4_WADCW Uncharacterized protein OS=Waddlia chondrophila (strain ATCC VR-1470 / WSU 86-1044) OX=716544 GN=wcw_0180 PE=4 SV=1

MSFFCPPLGAAVSSAMTAYSVGMTAYSVENCAYDNYDTLKEIGDVAFAGDLGQIAACLTS

SWEAWLSMPCRSQLL

>tr|D6YTG0|D6YTG0_WADCW Uncharacterized protein OS=Waddlia chondrophila (strain ATCC VR-1470 / WSU 86-1044) OX=716544 GN=wcw_0044 PE=4 SV=1

MINRIDIFMPPVSQYGVLPYLTNELHQAFVRQGVVCKLLVAERNNPEPFLESIFRDPPDC

TFSINGLLPDEAGQFFCDMIKIPHVACLTEGFTEFSVLAHNKLNVITCPDAFGCHYFQGL

GCSQSIFMPQGVSRELKPDLLLSKKYEVLFVGSCIDYEYIREEWKKKYPKVIYHALENAQ

EIALSDYSTNYIEALVQALNAASQKEELDTNSFNMLEIIQELELFIKGRDRVELIRSIKN

APVTLFGGAIGIKGWDHYLKDRKNVNIHSPIPFESALEAMKMSKIVLNSSPASKNGAHER

IFSALGAESFLLTSQNTFMDHHFVNGQDLAYYIHGEWSDVDEIVSFYLENYQEREAVAKN

GREKAMQQHTWDHRVQELLKQLPPLIEKARASLK

>tr|D6YTN6|D6YTN6_WADCW Peptidoglycan-associated protein OS=Waddlia chondrophila (strain ATCC VR-1470 / WSU 86-1044) OX=716544 GN=pal PE=3 SV=1

MNKKSLVIIMQFNFFLLALTGCCRSSEDVWDDTKTAGRHMGRGVRTLAGKHGDSRQVRCR

EDFMNCREYEEDDFYTSAYSGLEFEAFPDQDYGRDVALAEPRYHPQKQRIEPGMSKQLPG

IEDFTDPRYDVELRTVFQTVYFPYNSGLIKGKGNMDRLRTIAAYMQKHPDTYLFVEGHCD

ERGAEAYNLALGAKRSNSVRELLIKEGVRPERIFTVSYGKERPADSGHNEESWSKNRRAE

FKIFNSDS

>tr|D6YVG6|D6YVG6_WADCW Putative membrane-associated protein OS=Waddlia chondrophila (strain ATCC VR-1470 / WSU 86-1044) OX=716544 GN=wcw_0760 PE=4 SV=1

MSNITFIFLKTSLNKVTSFIYETPLSIVATGVAGIAFSIFSFNLATPLLALTCAVTLTRM

IVKVLEKSDLDLFVQFNEKMDENDSKYRNLYYMAYTATILVSIVLPALGIVLGIGIGTYK

GLIVQIQIQKIKQDTREQEVRFNLFSPSSWPAGF

>tr|D6YUV7|D6YUV7_WADCW Uncharacterized protein OS=Waddlia chondrophila (strain ATCC VR-1470 / WSU 86-1044) OX=716544 GN=wcw_0548 PE=4 SV=1

MNKILLILIFTNFLLYADSSVDIQKELQGMPNAEQEKIEHFFRTLIKQYGFGYTLFGDKP

VSLLCWLAEPDYNKKRPYFTVNEDFIESYSVWKKYEKKFPSNKYILQERTLAMGKDYIDL

VLIKKTLFNESIKNNNDFFSPGYDTDTFLKSEKLENYVNCSDEQKSKYHIRMGILLGYGS

RAFS

>tr|D6YVD5|D6YVD5_WADCW Uncharacterized protein OS=Waddlia chondrophila (strain ATCC VR-1470 / WSU 86-1044) OX=716544 GN=wcw_0729 PE=4 SV=1

MYQVPRIFHKDPRLSVPNSVQNQEKSILGKIWHVARVILIGLAGAAMFATNPTLFTLGFI

SGIIWDAKVQEITDKIKSIWKSQTGGVLLLAGIASFLALQVTWAAGSIFYAANLGRCMVQ

RAQRAVNSWDDTDRMHFANEFG

>tr|D6YSU4|D6YSU4_WADCW Putative methyltransferase OS=Waddlia chondrophila (strain ATCC VR-1470 / WSU 86-1044) OX=716544 GN=wcw_1800 PE=4 SV=1

MRSLYTSFLLFILSVLSITNDSNAVESSSGRVIIEKDQHAERLNFLKQKGFDPKIIYDIG

AYHGLWSKNIQRVFPNAQFHLFEANASHQDLLRATQMPFYIALLGDSDKAAVFYSNDSTG

DSVLREQTKYYQDECCQSKVLPMTTLGSLVKKNNIPLPDLIKMDVQGAEKIILQGSPEVV

THAQVIILETKILEYNEDAPLILEIMNLMQNLGYRALDILELHYLPTGELNEMDVLFIKN

GSPLIKSGLLIK

>tr|D6YUH0|D6YUH0_WADCW DUF374 domain-containing protein OS=Waddlia chondrophila (strain ATCC VR-1470 / WSU 86-1044) OX=716544 GN=wcw_0409 PE=4 SV=1

MKLRKKEFFRKTVIPFFLGYTLKGVISLLAMTCRFRILGTEKLHQSAKNSKCILIAWHNR

LGIITEILKRTGPQYRYAAMVSNSRDGQFIAVIANSYKEGRAIKVPHNQRAQALQMMINQ

LKSSNEIGIVTPDGPRGPKYTLKPGVALAARKCNAQVIPLSWSANRFWKFNTWDGMMLPK

PFSTITVQWGNPVTLGKTSETTVEQDAQQLEHALLAITNDRDPNGTEKAAY

>tr|D6YS42|D6YS42_WADCW Uncharacterized protein OS=Waddlia chondrophila (strain ATCC VR-1470 / WSU 86-1044) OX=716544 GN=wcw_1538 PE=4 SV=1

MITKNTIDNLTSFIMDPSNNAREPLTQGIAWTTTIILGFGTIGIIQGLSALWRKLRHIDQ

NDTHEKISRLFQKIFSRNSANETFERTPNTHTATTLGSESLRSASEPRLIHEPVEMMHIQ

KIDPEFTEVKSIIEQREAHKNAKKYMQQEAERIGAEVFFVLPGKLSSCPKVDDVEIKHLD

AKVFTWQNTNGVQGDIQADGKNSDHVVLYGVASQFNSCEAVSRFTPEPGTAVETYRTDPT

QGPGAQLQFPDEQVEIINHAANLGFNGLCEVLDDSTKNAVKHGYLTPKTEKSASIVIEQL

KANGHKMEFPCIGNIPKGANTEKVYEILVAAPAFGIYSLGKITDQKKKKIEFLCALQSYR

AQFQQALKLAALHPQKQLIFKPTAPGLGVFGNRVDNVAKAFYVAAKEYESRLIDKKIQVR

LQVFQSRGSARMMANTLRLTQWIEGV

>tr|D6YW05|D6YW05_WADCW LysM domain-containing protein OS=Waddlia chondrophila (strain ATCC VR-1470 / WSU 86-1044) OX=716544 GN=wcw_0956 PE=4 SV=1

MDSDRNHHKGSAKSTSTVRKLQQRTTTIALSILVAVSFGVVGLQMLSASSKTSESHPRMN

GTKALTILAQEKEQNQAGVATNLSGAWNSQQNDAETVRELNKIQARKIKDLKRELNNANV

KLHKIKSELFTKGDPSDRARLAEVCQTLTEKERCNEEFQKKISELESERDLRNQKITRME

QTIDALAMMTDTQRDTKEKAIFNLQSQIERLEEDAKRERNELKKTLAELEETNHKLKENL

ADKAAAVKTLEEEISWQYGLMKEKDKDLQSQSKLYTLSENQLQKEINHLGESLELEMLKN

QSLAAELEIALAKEKAQEQYTRSLESQLDKNKNLSEKEKNQWNQNMVNFAKEYLELQSIL

DVYTHSHDHLTSKQTKLATLVKEEKSKVESLKEELDTALAAADAEQQKGLCIEEELHATA

HKILEMEDELKKKQVDIESKQQELDTLTYSNASLRDQLHARIDQLTTLLEESQKEVRRKE

ASVRDLAINLELERTRTQEFNDRQLQQSEQINAMEKDLKTHQEEVNRLQDKVLALTSEYE

QEKQHSNELQASLSDYESIYNDYDKLQKTFDENIDLLQAKLDKATTDLNNEQKKIQEASK

LISSLNDELAQKEDKIEKLHSSLASQENQVDALIAQLEEERNHSNFLEDRVYSIEETGPS

SLQIKEMQQTIAQLSKKVELERHRTLAYEKANKNQIGHTKFLEEKLENFSAQIAELQKQV

DKKDQLISEMRLKKENQPEIADNSGRSVSYQHTVQEGENLGIISTYYYGTPNRWIDIYNA

NRKAILDQNKLEPGIVITIP

>tr|D6YWL9|D6YWL9_WADCW Bifunctional protein FolD OS=Waddlia chondrophila (strain ATCC VR-1470 / WSU 86-1044) OX=716544 GN=folD PE=3 SV=1

MDHFNMDIDGKTLSKQIRNAIREEVKILKGRKPYLAVILVGKNPASQIYTNRKVKACKEA

GMQSKLIQLPDSISQQELIDHIEQLNKNSEVDGILVQLPLPPQIDPDRIAETVCPEKDVD

GFHPINLGKLLLGNGEGFIPCTPLGIRKLIEHYKIETSGKHIVILGRSNIVGKPLAALLM

HQASYGNATVTIANSRTKNLSKITQQADILIAAIGKPKFITKSMIKRNAIVIDVGINRLD

DSSLAGDVDYADAKGHCSMITPVPGGVGPMTIAMLLHNTLKSYRTRV

>tr|D6YUR1|D6YUR1_WADCW Uncharacterized protein OS=Waddlia chondrophila (strain ATCC VR-1470 / WSU 86-1044) OX=716544 GN=wcw_0501 PE=4 SV=1

MLKASSYLFIASTLFSAPVFSDGSPTDSECGFNKAAHHNSKAFTGRVSRDRVRLRLSAST

DSPIIKELNRGDMFLVTGEEDDFYAIKPLNGTKAYVYRTYILDGVVEGNKVNVRIEPHLE

APVIGQLNMGERIKGKISDKNSKWLEIDPPEMTRFYVSADFVEKIGDADYLAHFEKREND

VNDLLNGTYLISQQELQKPFSEIQPEKVVKNYQKIIEEYTEFPREVKQAKDSLAKFQETY

LHKKVNYLEDKASKADTDWKHRGQSETAMQDHQQPSANSSQADASQVYKKWVHEQAASDV

NARMALWIPVEIAYYEKWAKDNQNRPIQEFYKEQRGHAMALRGIVEPYDRPIRNKPGDYL

LVNRGNRLPIAYIYSTQVNLQDYVGQEVSLEAVLRPNNNFAYPAYYILKAE

>tr|D6YTW4|D6YTW4_WADCW Putative biopolymer transport protein ExbD OS=Waddlia chondrophila (strain ATCC VR-1470 / WSU 86-1044) OX=716544 GN=exbD PE=3 SV=1

MRFKSSLKPSSSLIDLTPLVDVVFLMLIFFIITSDILPLKSLNIENPTLEKDSAPLTTQL

IIVMDAQNVIYVGSRKSIVDLVSLKEHLQEELKKLKKQNFGMEPTVVLSIDQHVDYGSFL

HLFSICQECTSRLRLVYKPAESQQAEYF

>tr|D6YWM2|D6YWM2_WADCW Uncharacterized protein OS=Waddlia chondrophila (strain ATCC VR-1470 / WSU 86-1044) OX=716544 GN=wcw_1176 PE=4 SV=1

MKQKPLSNLETVARGSEFQRSVSDLTEAFVEDMEEMMLKDLEPYEAYANHIRHQVCGNLE

QFRSRFTRGYQALLNEMKEIESDKEH

>tr|D6YWC1|D6YWC1_WADCW Putative mechanosensitive ion channel OS=Waddlia chondrophila (strain ATCC VR-1470 / WSU 86-1044) OX=716544 GN=wcw_1073 PE=3 SV=1

MRFLVLAILVFAQLLNAQEAENASTPDPSLLTPKWWDVFVGEKAIVQQRSAELLNHLNPI

PEDLSGPHQEEARNLIDSIKVNLQAWINIFDQPLPLSKPATTFKDFYTISQFRQLYSRLQ

KQQIDLESKKKEIFGLQQQLNTNRERLDAAWQGYQFALDRSQEKILFGLKILKFRPAVEV

GKKKIQLVNQAIKIEEQQIKNDKEEIQTAKNRLISTHADWMNALNQLEILKRVWDDSKQE

RQRQEILYAEEHSEDPNEKDEAKNSLLTVNLINAFLKEMLAHFNYLRMEIIGKLSQLLED

PESVDIHELDVSLKHWKTQTRTFQLQLDDWKTQTERILQSASQVLTLEEINSPIGTTQLN

EVIQKAQGILLEIQKIRSEFDETNFLLNTLDEKSIFLRGEGEQWFKVFIDFVSTLWQSAN

SQLSKTLFYIGTHPVSVITMIEFVMIMVATWWFSRVVTGTINTFSKRRKGIRKALIYSLN

RLIHYFLLIIGGLIGLSWIGFDFSSFILIAGALGVGLGFGLQNIFNNFMSGIIILFQGHL

KVGDYIELDSGLRGEIREINVTSTVVTTNDGIEVIIPNSEMVSNRIVNWTLRDPYRRVHV

PFSVPFGSDVDKVTKIIVEAAKKVPSTLQRIGVREPQIFLVQLGDNGIQMELVVWVDEKW

TRRNRNTQSQYLFAIEKALRENGINIPLPQRDIHLRTTALDK

>tr|D6YT66|D6YT66_WADCW Putative secreted protein OS=Waddlia chondrophila (strain ATCC VR-1470 / WSU 86-1044) OX=716544 GN=wcw_1924 PE=4 SV=1

MFPPIDHTKPNVSAAVSLPETSSASQSKIQYFVINNFDFISTRTCRDLCITQIDSASELD

SKIQQLVRTHFEGGQRVYKMHEKIAALDFKNIVVQDGENTQVISAESIKIIFLNDEEYDL

MVDSFIEEHSTKVEEEADTRSIPLFSKQGLACIKAMTLVFRKNQEIVAFFTQVREQELTR

VDEEIRERQTKNRDKRWNQWMDYWLQKDENRREILVTVLKNSLL

>tr|D6YV83|D6YV83_WADCW Uncharacterized protein OS=Waddlia chondrophila (strain ATCC VR-1470 / WSU 86-1044) OX=716544 GN=wcw_0677 PE=4 SV=1

MPNKQYDKQNYLSILDAVETLSSIVDIDFEREIEVANDEEIKEQDEMVTAHTMHWLETED

RQNTVKHIKETFHVVHEYLKDFYEHEYSYVSKPKVMEGVKTIMVLVGEAAKKLDKFTTLF

KETKERSVKNLKEYRDLQEFYLTRIARKIDEGILGKWILELTKGSMAKRKAKAELKAKPM

ISVKHVFIDMESVKNDTEYELFFLRKEDGSRFFNPRLIRNIKLTADFGQKIRSKKSSDPL

EQINIWQDHLIHVAARNILQAQGGILDRFFREAKESWRRELASELYKAVVALMMCACPKN

LLSNAPTKACTEYFIDFHHFLRNAYVTGEYQRYLAYPPKKSSKMAHILMDLVHGLSRAMF

TRIKAYQQLVTVFEKMVEEANQMQSEEHQKAAEESNTLWSRLAGDYAALSKMMKTHPNGP

LVKVLEVLEKGAYHVFDPIAQLNLPGVMYSLFMGENRITNLHLPAPMNQGFIHQAEIVNE

FKGFLRAYMSGQFKRKHLLFNFQDRTSWKEHVRAVNLEQLSNQRLFSKYLTVVSLPKDTD

FYHQVSPYQDDHQAGLFIEHFKDNLSDESCGFVFPFYLSKKLFPNFINQLFEGVHKLFFH

ERNVLSVKDRLDFIEIFYMLLELKIIEEEQPATFSMTCKDGIDIGGCASAELFGFLKMIN

NQVLSNDEYMEYCTSLFMPAIISRERIIHTDRFGRMITAFKRIESTQQEMGKKAFAKAFD

ELLGSLFHSPILRSELAFPRQ

>tr|D6YRS7|D6YRS7_WADCW Uncharacterized protein OS=Waddlia chondrophila (strain ATCC VR-1470 / WSU 86-1044) OX=716544 GN=wcw_1422 PE=4 SV=1

MFRTTEIDKKIRQLIDKQKKEEFMLLANEKTRILEMVAAYKERRDLENQLKRAEQKAGEA

EAKVIGWNQLFQGLHHFTKVFARYAKADETSKQVAELTEKMKSGKEEISALRQRQEVIHG

KIERLKTYYKAQASISSAQAMAGLLERIGEFQTAQI

>tr|D6YTB1|D6YTB1_WADCW Putative membrane protein OS=Waddlia chondrophila (strain ATCC VR-1470 / WSU 86-1044) OX=716544 GN=wcw_1973 PE=3 SV=1

MMLILLIDRIFLVYMIMLFVRILGSWLPELNEYKFMQFVRYYTDPYLNVFRQIIPPLGMI

DFSPIVAFLCLGIIEHFTKWLAYTLFY

>tr|D6YVJ4|D6YVJ4_WADCW Uncharacterized protein OS=Waddlia chondrophila (strain ATCC VR-1470 / WSU 86-1044) OX=716544 GN=wcw_0788 PE=4 SV=1

MQQVKNFFSYFNPTTDKYEGAWEAFDSALTFGQQLQVITLTALAALVSLPILGLGGLAAF

RALVNHYVPYTADDLPDSFNGNTAGKVHDIGLNAFNPMGQAQFVENAKRLFNVASTFDAL

FDPELDTILEELFDKYKNGPILLTALKVGNPSERLEILKDAQKLDGSMDAAQEFLQLMKF

VSEIKAKRETAVPRLERFGEGISKAAELKSLHRALESIPVEEEEELLNAASIIFKNFKTA

LERINCLQVIRNHISIGKRADAIAYHQKVLSTITSPTILADVLPLIQEQTKENLRAVEEL

VLLLSNPEDDGTVLLKCLRCIEGVSDDVSISLLENIYKCLDGLAGENLKPVLQILHHISG

LTVERRTDVVAVALTHIRSSMHPTERRLIMSALCDLNDPAEVNKDELQDLIKTKKIIMEF

FGKISNALDKQKMMKEIQRIPENQRESLFSTISPVLSQVASVEQFKELITGIKMIAPQHR

NSVIANLSGKLSDPFDWLTIVRQALQDTQIKADSKQYLIELLRVETDQEIALDIANKIWD

NRNVFGVVDGDDGDELFILLLGIVPIADGHARSPYTLYGRLLKMDKEISTPEVLKPTRDL

QGVRVKMNQPHFEKMVRETKRVTFEELDSSVTFEFLEKLFSSFETRLSRLSSEERNKVWT

SGVANCVVVNGAITAGNVKNAYELLRHTFMGTGGYVNQILKIKGADHQVVPTGYLHVYAV

IKYVAACSIEIQSGSGLSSQEETLLRMLSSIQNCATGKSEGFAAFYNQLPHEYRKFGAAA

ASTPAEKAKAYLGGVVQKVIGSYLDHTSINRMAKELIGAPDDGQYVHTSKYLKNLIAKRV

GMPWDISFDAYPGCINDTLFNRELQDVLEIFYKHVTPKDFANKVYKEMLEEIVPNRDEIK

RIDEKLRQAKSALEKKMEEGNLPELRKSVNELNRQLSKVRIERNVEEVTRLTKELEEVRT

KLKNEEERLGVASLKEKVELIEEEFEGPHKKVKTELTTQFMQLLSEKNLDGCFCSIDDTE

MVHAITEKGVVLALIEAGYLAAS

>tr|D6YVA7|D6YVA7_WADCW Ribonuclease HII OS=Waddlia chondrophila (strain ATCC VR-1470 / WSU 86-1044) OX=716544 GN=rnhB PE=3 SV=1

MMKLFLQKTKSRKRLPQYQPLKRRLKLKLTKKKLLVATQRKKKTPLPDSEEFLLDFSHYE

NEARRMGYSHPAGIDEAGRGPLAGPVVASACILPAGYHLSGLNDSKKLTPLQRKRFFEVL

TQDPNVCIGIGIATHEEIDTVNVLEATKLAMCKAVDMLKPQPDFLLIDAVKLSEQPIPFL

NLIKGDCRVQAIAAASVIAKETRDRIMDEYDVKWPMYGFGRHKGYGTEAHREAIKRHGPC

PIHRKTFEPIKSMP

>tr|D6YUY0|D6YUY0_WADCW Uncharacterized protein OS=Waddlia chondrophila (strain ATCC VR-1470 / WSU 86-1044) OX=716544 GN=wcw_0571 PE=4 SV=1

MSITRPNIGIEGAFPFFKFYVKLSVMEIIVKEAAHFVALIAEFVAILVILLGVIKTTIFY

FKSPLPWRFVPEAFLKMRVQLGGALSLSLEFLIGADILKTAISPTWSEIGILGSVVVIRT

VLNYFLTQELSEGIGKVK

>tr|D6YRV4|D6YRV4_WADCW Putative rRNA methylase, SpoU family OS=Waddlia chondrophila (strain ATCC VR-1470 / WSU 86-1044) OX=716544 GN=wcw_1450 PE=4 SV=1

MNTKRKFLRLPSVSQHKKCAELLRCFYTDAQRVFIDNYNEIQDWMGQPSLEDLSPKSVSD

RFHKHLKYAEVRLREHSLLPRINKKDRPKPSANRIDVHLYLDQIRSAHNVGSILRTVEAF

QLGDVYFSKDTPLPTHPQVSKTAMGCSKEICFQRHASPSELPRPLIALETSSDGIPIDKY

TFPSSGCLAVGNEEYGLSDEVLSQADAVVEIPLYGKKNSLNVANAFAIAAAAISQQFRG

>tr|D6YTP6|D6YTP6_WADCW Uncharacterized protein OS=Waddlia chondrophila (strain ATCC VR-1470 / WSU 86-1044) OX=716544 GN=wcw_0132 PE=4 SV=1

MAANQGMSGFSPWVRLFFLFFIGGLAVAGFAYFSSDKSFDVVGDQLQDLRNHRVSKAYYE

HMSKEFQQTTSLPQFREFLSIYPVLYENLSYRLETSTVEDSRAHLTGILVSKGMEEMKVD

WGLVKEDGKWKVAGLRLTELKQDDEADKMIDFAKEQLQALRERDFVEAYYGFVSKDFQQQ

ISIGEFEAFVKENPILFNYRMIDSENSRVEDDRGYVSLHLENGKQTYLLNYTLSRESDGW

KIYSLKVILPPEVAIQKAETNPEALVPPIRELLEALEEHQISRAYQLTSKEFQGSTSFEN

FKKFIRSFPAFSVREMADIKKREVQNGTGWVRVNLHDEEGITAVDFRMGYEEGEWLIWGM

EVVDSPVKEKQESRNATVPPLADQLVSLLRQQRAYLHYEDINEAYEQIMSKEYRVHNSVE

DFQAFFAANPAFIEHRSSYFNRLLDQNSRAVLRGFITTKGAETLPVRFDFVKEDGKWKID

RQQLLKEPEPIAEAEEVFDKPEEDSPPKPLEFAKIVLGTEVNQQGIVTTPLDEVEGDEDF

LFFNVYIDNGQPETVVTLFLEHVDSGTSARPLSTKLDRKGKSTISFSYSSPKGGWPSGDY

IAKLTASSGQEYLFKFKVLK

>tr|D6YSA9|D6YSA9_WADCW Uncharacterized protein OS=Waddlia chondrophila (strain ATCC VR-1470 / WSU 86-1044) OX=716544 GN=wcw_1609 PE=4 SV=1

MVDLNLNMDLPPPSFFEELNQQTTQQQINTLSVAYPELSQDEIVAYVEFYSDLGIPFNTD

ALQNLLASDPILTEPRAANTALNEIVASTKNIWFSPNPFATFFILFLELQAKLTEIKVAE

AEVMSTMINLTLDMAEDLAGIIHEIYENEAWKAIASGICNFIGGLASVGFGIAGLSKIGG

GAAGIAQAQSLGAIGGGIGQISSAIDKFVTAGFDFRRADLEYSKTIIENALKVLQDRGMA

SASEAQRTADELIAQILQKLDKIIDEAYRAHGFQVH

>tr|D6YVA3|D6YVA3_WADCW Signal recognition particle protein OS=Waddlia chondrophila (strain ATCC VR-1470 / WSU 86-1044) OX=716544 GN=ffh PE=3 SV=1

MLGALTDKMQDLFSKITGKSKLTEENIAEAVSEVRLALLEADVNYSVAKVLVKRLKEKSL

GDAVLKSISPGQQFIKIVHDELIELMGGEEAPLDLKSKPAVVMMCGLQGSGKTTQCAKLA

NYLKKQKKCRQPLLAACDLQRPAAIEQLKTLGGKIGVPVFSIPNENSPVKVAKAALERAR

QEGFDLLILDTAGRLHIDDELMDQLQQIKQATKPHEILFVANAATGQDAVTVASEFNQKV

EVTGTILTMLDSNTRGGAAISIREVTGKPLKFEGVGEKIDDIQPFNPNSMADRILGMGDT

INLVRKAEEHIDEKEAKKLEEKIRKATFTYEDYLKQIQMVKKMGSFKSLLGMLPGMSKMK

EMEIDDKEFFKVEAIIQSMTLNERICKCDLSIPRRKRIARGSGTAIDDINRLVKSFKKAK

QFFKNMPNMKQLEKLMGGSQWR

>tr|D6YSL4|D6YSL4_WADCW Putative outer membrane efflux protein OS=Waddlia chondrophila (strain ATCC VR-1470 / WSU 86-1044) OX=716544 GN=wcw_1715 PE=3 SV=1

MRIRYVIFILLAAAGCRVGPRHSVPEVEISENWQSDSLSTLSCGSLLEVGELFDDCLLNT

YLEQLLHDNYDLKRASIKICEAFALRDISAAKLLPFIDGRIAYNSAKPAGGILQPGDFSS

GSVGIPLNIKQQTFISDFDAFWEIDLFGRRQREVESATASIQMERASYHDLIVSLTAEFS

RTYLKLRERQKCLELVRKERDVLKEIVEEYQKRLDQGLDSEFFLLDSEKDYEQLAAEVPM

LEGEILSLIYHLSILLGKPPEALVKELSSAAELPQMISVIPVGFPSDLLRRRPDIRFAER

QIAKATADVGVAVADLFPKFTLTGNYGFQNLHLGNTRGNGESWGYGGNLITPFFHGGSLK

ANVKRFQFIRMESLMAYEQAVLRALEESESTIASFLKSRESTQNREKGWMRADQLFAHGE

RLYEMGLSDTLRLLERKRRAIQAEREWATSYVETEIQLIALYKSLGSGWQNLSFL

>tr|D6YVG4|D6YVG4_WADCW UPF0056 inner membrane protein OS=Waddlia chondrophila (strain ATCC VR-1470 / WSU 86-1044) OX=716544 GN=wcw_0758 PE=3 SV=1

MTIFSISLVLFLIMDPIGNIASFLRMVEGIDPKKQKLILLREMLIALLAMIVFNYIGEGI

YSLLQISDPAVKLSSGVILFIISVQILFPNSTGLRENLPKEEPFIIPLAIPLIAGPSLLA

TIMLYAHMEQSLFVMLASILCAWMAALIVMLASKPLYKALGENGLMACEKLMGMVLVLIA

IQRFLDGIRQYVATF

>tr|D6YWE1|D6YWE1_WADCW Uncharacterized protein OS=Waddlia chondrophila (strain ATCC VR-1470 / WSU 86-1044) OX=716544 GN=wcw_1095 PE=3 SV=1

MYNKFMKKKDVEKQLKKLGWYLDREGGSHEVWTNGEAKTVVPIKAY

>tr|D6YVP3|D6YVP3_WADCW Uncharacterized protein OS=Waddlia chondrophila (strain ATCC VR-1470 / WSU 86-1044) OX=716544 GN=wcw_0838 PE=4 SV=1

MKSDQQGYQVKLWAIVGPLICLFSLFVISIKNAQVPFFLPFALLIGMPVCWRWRLWGWGG

ATLFLIACLAFEYDLIPLEERFWVVGISFSNSLALLITALSFEEVETQIESLGVESRSRL

ENLWKVDEKKQAIEQELAAKKEEVKNLKFKVRSFQKLIDLSTEEMHSARADHDKILQEFC

QIKDENEKLTELLAKSESDPPMEAKYRQLREQFKEKANVLVETRRDLFLANEKISRLQRE

LDEERWYTLSEVEELLEKHILELSREKEIQDEQHQREMEALLALVDKFILK

>tr|D6YWU2|D6YWU2_WADCW Uncharacterized protein OS=Waddlia chondrophila (strain ATCC VR-1470 / WSU 86-1044) OX=716544 GN=wcw_1246 PE=4 SV=1

MNQKYKEIVELEKKLSTENNQGKRKHLKKEMHKKKKEVNALRQKMIFGDPPPMGGMREIH

KWMKDVLEPEFLSTYPNLKTRETLVIFRKLLGSQLEIEGAALERNQEHLMFFALNKQLGV

ISAMNCKSGLDRTGFLFALFLGAQDLPEDKKMKIALNWESYTLELNQLYRNNGYDPAKVM

AVLDEPHRDHADELKHVFQMQQNVLQHLLTVSLPITGISTGMIGLKYGKGMKENLLPLYC

IPPVVKNESGEIIQLLRYKRSGRPKGLTTEGHRLITQLSLHRGA

>tr|D6YWM7|D6YWM7_WADCW SsrA-binding protein OS=Waddlia chondrophila (strain ATCC VR-1470 / WSU 86-1044) OX=716544 GN=smpB PE=3 SV=1

MMFAMGKKSSSSELVSNRKARHSYEILDTYEAGIALTGTEVKSLRDNGGSLQEAYIRVKG

GELWLLKCHIAPWKYGNIHNHEETRERKLLMHKKEIQRLKAATQEKGLTIVPLALYLKNG

KIKVSLAAAKGKTGLDKRKSLKEKDEKRRMQQAMKRDVSH

>tr|D6YS21|D6YS21_WADCW Ribosomal RNA small subunit methyltransferase A OS=Waddlia chondrophila (strain ATCC VR-1470 / WSU 86-1044) OX=716544 GN=rsmA PE=3 SV=1

MKSLMTSNPLRLSNPSQLRQYLEILGISPKKSLSQNFLIDGNIIDKIISLAEINAQDQVL

EIGPGPGALTDALHQHGARVLAVEKDRILAKALQERGGDIRVICEDVLKVDLEKELSERA

TVIANLPYNITSPILTSLLPKTHVFKRIVVMVQLEVAERLTASPGNKTYGSLTVFSNLFS

TPQWGFKVSRRCFFPEPNVDSAVVRFDLSPPPKEVEDEAFFQLIRTAFGQRRKMLKSSLK

KLYPSSSVMQALAGIGFQETARPEELSSNQFVEFYRHLIYMRS

>tr|D6YV17|D6YV17_WADCW Fumarate hydratase class II OS=Waddlia chondrophila (strain ATCC VR-1470 / WSU 86-1044) OX=716544 GN=fumC PE=3 SV=1

MFSGIGNYRVVCLICICLCPHAAVMEARIEKDSLGEVAVPADKYWGAQTERSLENFPIGS

HKMPIEVVHALAFVKKAAALVNFDFGLLEEKKCIVICSVCDEIIEGKLDGHFPLVVWQTG

SGTQTNMNVNEVISNRCIELLGGKMGSKIPIHPNDDVNKSQSSNDTFPTAMHISSVRMID

QLFFPKLRALRQSLEEKTVAFMEIVKIGRTHLMDATPLTLGQEFSGYVSQIDHALKAVEN

ALPHLRELALGGTAVGTGINTPPGFAEKVAAKIAELTGYPFITAQNKFEALAAHDAIVEM

SGAIRQTAVSLMKIANDIRLMGSGPRCGIGELILPANEPGSSIMPGKVNPTQCEALTMVC

TQVIGNDAAIAIAGMNGHFELNVFKPIMIYNLLESIRILADASLSFKERCIDGIQPNKKR

IKQLLDQSLMLATALNTEIGYDQAAQIVKKAFTEEITLKKAALSLGLLSEEAFDRIVDPS

KMINR

>tr|D6YU18|D6YU18_WADCW Uncharacterized protein OS=Waddlia chondrophila (strain ATCC VR-1470 / WSU 86-1044) OX=716544 GN=wcw_0254 PE=4 SV=1

MSIDRVLLICLILAAQSLFASTKTASLRYLPNTPVHLASDLKLDISQSLPGLSLSTKGVQ

RLEADLTLRNEQPEPSSSVLPLNLTFVLKKLSIDLQANDETLTFRSDEAGTSLYLTQLSK

LIDRPIQLKLDRHFQLSRDNEELRRAVSELPVLAEMNPDHLLVELFSSVFVPGNQELSVG

QVIRKDLSDWEIPSLPKEVVYTITKIDDYSVYAEICGEIEKKKFQLSGEVVIGGKGESVA

AALSGLMEGKVKWNRDNAMLYELELNYSYSTRLQLASWDWLMNVSLNLHNKTIP

>tr|D6YU49|D6YU49_WADCW Putative ABC-type oligopeptide transporter, permease subunit OS=Waddlia chondrophila (strain ATCC VR-1470 / WSU 86-1044) OX=716544 GN=oppB PE=3 SV=1

MFNYIVRRLILLPVTLFFIILVNFVMINLAPGDPVTVTEISPEGIATRKEDQSLAFGSDN

RYLQFREHYGLTLPILINLWPWLSLEDVIHSLEQLVEGKERLGVKKYDQLRILVGDQSPY

VMPLLLQIIENHKLSIPLRKMAIRFFVRGGTKQAHLGYRLTDAQKAYNRKIAVDNNFLST

FLGTDQPIDESAKKLRQWYDQNKEYYRLDPAGWKKLYLLTETRFTRYMGRVLTLDFGMMR

NDSSRSVIGEVAKRFKYSLTLAFLPMVMTFFLSQIFGFFMATHQNRWQDFSLNVVFLVMY

AIPVFVAAPFLIEKVALNYDFPVSGFTSSESVYEKMTSNERLWDVLRHIALPLVAVMYGG

LAASSRLSRTAVLEVMRQDYVRTARAKGVGRLAILGKHVGRNAAITIITSIAGSLGIVLG

GSLIVETLFEINGFGKFFYDAVINRDYNVIMFSALAGSFLTLYRS

>tr|D6YRH6|D6YRH6_WADCW Multiple resistance and pH homeostasis system, subunit F OS=Waddlia chondrophila (strain ATCC VR-1470 / WSU 86-1044) OX=716544 GN=mrpF PE=3 SV=1

MNRDKFMINFFPFAVFFSSACLTLSLFLGMYQLLTGPTLPDRIVVLDLVASLVMGLIIVY

AAKTGETVYLNAVVVIALLSYMSNIAFAKYLRRQIDD

>tr|D6YU88|D6YU88_WADCW Putative membrane protein OS=Waddlia chondrophila (strain ATCC VR-1470 / WSU 86-1044) OX=716544 GN=wcw_0325 PE=4 SV=1

MIDLPAVAILSFLFLLGAVFWKWGSSFQVPHLKFSQLQPLIEGPLSLKQKYAKLPRYLLI

AAGFFFSTAFLDPHYFVEKAPEVKKETPLQQVSIPSEGIAIYLVLDQSGSMSEEVKVFRK

TITKMDLLKEVTKGFVLGNKQEGLTGRPQDMMGLVTFARGAQVLAPLTLDHQAIIDQLSK

LQYTTDLEQDGTAIGYAIYKTANLIAATRHYAEELEGAGKPAYTIKNSIMILVTDGLQAP

NPLDQGKEFRNVELLDAAVYAKKLGVKVYIINVEPRIASEEFSAHRLLMKKITELTGGRF

YMVDNSLNLSSIYSEIDQLEKSPLPLETQYISPPKSMQPQLYRRVSFYPYLVAAGMLCFL

LSIVLETRVFRRVP

>tr|D6YT05|D6YT05_WADCW Enolase OS=Waddlia chondrophila (strain ATCC VR-1470 / WSU 86-1044) OX=716544 GN=eno PE=3 SV=1

MSRIRSVKAIEVLDSRGFPTVEVMITTDQNMMAKAVVPSGASTGTHEALELRDGDKSRYF

GKGVELAVQHVNGPIAQLLIGEHVFDQTRLDMLMIAADGSKNKEHWGANAILGASLALAR

VGALTAGLPLYRYIGGCHTYVLPCPMMNIINGGAHADNSLDFQEFMIRPVGAPTFKEAVR

WGSEIFHTLKALLKKKGHVTAVGDEGGFAPNLNSNEEALDLILEAVEKAGYKPGEQIALA

LDCAASEFYDKMAKKYIEKKKQERNQSYAERTSEEQIGYLKELCARYPIDSIEDGLDEAD

WSGWKLLTEQIGAKVQIVGDDLFVTNPDYLSKGIREKVANSILIKVNQIGTLTETLETVK

IAHTHGYTSVISHRSGETEDTMIADISVAVNSGQIKTGSLSRTDRICKYNRLLNIEAGLG

NVARYYDSNQR

>tr|D6YTB5|D6YTB5_WADCW 5-formyltetrahydrofolate cyclo-ligase OS=Waddlia chondrophila (strain ATCC VR-1470 / WSU 86-1044) OX=716544 GN=wcw_1977 PE=3 SV=1

MKRALRDHWISIRSQIQKSRREEAQSKLFKELTQQTENFRSVLSFFSFRNEISMDEFNDY

LAKNGKLCLPKIHEEHLDLYRVEEVDHQCVPNAWGIVEPDPALCEKIVEDEIFLAFIPGI

AFDSQYHRLGYGKGFYDRLLARLNDSTLTYGIGFKEQYSQTLLPIEAHDISLNRLLLF

>tr|D6YUX9|D6YUX9_WADCW Uncharacterized protein OS=Waddlia chondrophila (strain ATCC VR-1470 / WSU 86-1044) OX=716544 GN=wcw_0570 PE=4 SV=1

MKSSFFPSALCKGYFIFKKSTKNPYLFPTT

>tr|D6YVH1|D6YVH1_WADCW 4-hydroxy-tetrahydrodipicolinate synthase OS=Waddlia chondrophila (strain ATCC VR-1470 / WSU 86-1044) OX=716544 GN=dapA PE=3 SV=1

MPRLDGLKGVYTAIVTPFKKDKTLDEEGLRNNIQFQISQGVEGIAALGTTGEAPTLSEQE

KVRILQIAKEECAGRIHLMAGTGSYSTEQTIRMSKLAEELGADSCLVITPYYNRPNQKGI

YHHFKAVAESVHIPMILYHHPGRSGCRITTETLLKLSGIPNIAGIKDASGDLATQTCWLE

KVAPSFSVLSGDDPLTLPMLSIGASGVISVTSNLSPKKMINLVSAALQGDFQTAREIHYQ

LLPLMQALSIDSNPIPVKAAMNLINMAAGPCRLPLTPLEEHHVKFIKEIVHG

>tr|D6YSI2|D6YSI2_WADCW Early upstream open reading frame (EUO) protein OS=Waddlia chondrophila (strain ATCC VR-1470 / WSU 86-1044) OX=716544 GN=euo PE=3 SV=1

MSNIKEMCEEPKVVSITEAARINGVTRQAIYVAIKLKKLKAKKETTRWTIDVKDLDEYRK

NKYSRTKSMHEGELLFDNGRGYYSVNQVAEMLKVPAQKIYYATRVGMLKAHRRGAAWVIH

VEDVNKYKEEHLSKKGGRRRAV

>tr|D6YSG6|D6YSG6_WADCW Segregation and condensation protein B OS=Waddlia chondrophila (strain ATCC VR-1470 / WSU 86-1044) OX=716544 GN=scpB PE=4 SV=1

MTHKITGVPEEVVEAETHAFAKRIIESLLFSSNEPVPLRKLKEILQTVYPYSTEQVKELI

YALREDYAEQNRAFQVEEIAKGYLLRTCADYHPYIHRLLKHTRPDKLSQAALEVLAIIAY

RQPITRPQIDEIRGVDSSGIIHTLLDRLLIEQSGKLEVPGRPTLYCVTSHFLQHFGLKDR

EEFLRACRQFK

>tr|D6YX02|D6YX02_WADCW Putative membrane protein OS=Waddlia chondrophila (strain ATCC VR-1470 / WSU 86-1044) OX=716544 GN=wcw_1311 PE=4 SV=1

MGSLSYLDLAIAPSRQVSTLGLEVPTIEVPAIAATVHHRIARGPLEAIKDAYKRLEDLYH

QAFPITFQCRFAQIQLGLLKGGTLLSSLYKVLNPVMSIFRNTISTGEQSVFKETIWLVKF

AKKTRLLSIFSLPASLVQVVKGGYECVLSVKKKERSEALEQALNVADSLGDLSHGISTFI

DCLMDAAVVNSSAALTAASTYLTGIGVILSVASIGLQCKFIYESVKLKNRITRLFDSKGK

ADFQSVVEEIDKLSHSRLARTVGVSDGEKLKARLHAIYERNSDPLGNAEHKKNLVSTVKA

LKNRIHWNNIGRYLKITAAVISIIAVSILLFSPVAPIGYLLVAVSAAIGLAVLLIDYKAE

QDLNCHLKALAPKDSREMWKLYGKKQYRKKLKNDPMLRIWDLSNLGKDVQREALKRDELL

RELQEQRRLRFS

>tr|D6YTB3|D6YTB3_WADCW Putative membrane protein OS=Waddlia chondrophila (strain ATCC VR-1470 / WSU 86-1044) OX=716544 GN=wcw_1975 PE=4 SV=1

MKGIPQSRMILYLLVIGFIPIFFTIFYLSGQLTEVENLKNSLTLVEAQAFSREKKQALNM

AVKEHFKGADHFYVDKYLESQTFLSPEIESLQKLVNNRYFAGDESVRKRLDYITSQKNSL

LFSESNVQTYPFFQETTATLVHPIEININDLKRILSLVEGRTIGTNKPGPNRPQMIILDF

KIDKKEASENNEVLLLNMKILKREYL

>tr|D6YTJ4|D6YTJ4_WADCW Uncharacterized protein OS=Waddlia chondrophila (strain ATCC VR-1470 / WSU 86-1044) OX=716544 GN=wcw_0080 PE=4 SV=1

MHYFIDGYNLMFRLSMMNDSLQAEREQFISNLYAKIQVVGLDVTIVFDAQHQEGLGSRTH

LDFLEICFTDEGETADDYIIRELSSVPNPREEIVVTSDNKLAWLARRMHAKSEQVETFVR

WLDHRYRKRKKELPKDFVIKEPPKISLPEELPVSQEKSTADSYYLNAFEKKFKELEPERS

LPKPKKKTSSKKAGESEMERWQRLFENRDLDEDTPNVL

>tr|D6YUX3|D6YUX3_WADCW Putative pyrophosphohydrolase OS=Waddlia chondrophila (strain ATCC VR-1470 / WSU 86-1044) OX=716544 GN=wcw_0564 PE=3 SV=1

MTFVTKKLQDCKNQLEIFNHPPTDFAPQIEVAACYLLYSNEMLLLKRSYGKPEEGLWGVP

AGKIDPGETPLEGALRELKEETGIGLPPEKFIEKGKRYIRKPAIDYVYHMFLILLDAKPE

VNINSEHLEYQWIPPSQADILPLMAGAKAVLKAAGVIDEL

>tr|D6YS32|D6YS32_WADCW Uncharacterized protein OS=Waddlia chondrophila (strain ATCC VR-1470 / WSU 86-1044) OX=716544 GN=wcw_1528 PE=4 SV=1

MILPNMAVYTESTSRIGILTKSCLEFESGEDGAEATQPE

>tr|D6YU79|D6YU79_WADCW Adenylosuccinate synthetase OS=Waddlia chondrophila (strain ATCC VR-1470 / WSU 86-1044) OX=716544 GN=purA PE=3 SV=1

MPGVIVVGAQWGDEGKGKIIDILTSKAKHIVRAQGGNNAGHTIIIGEDEYKLHLTPSGIL

HPHTQCYIGAGTVIDPEVLVFEMNTLQSRGIDLTGRLWISPAAHIIFPYHRKLDLLLEQK

KGSQAVGTTGRGIGPCYADKAHRLGIRMAELMDPNLFPSLLKNALELKNEEITKLYNSDP

LSFEEIFSEYTRYGQYLAPHISHVEDKIQAALQQQENVLFEGAQGTFLDTTMGTYPYVTS

SSTISGGICTGSGIGPSQIDHTVGVIKAYTTRVGHGPLPSEVKENDQFLDHFTAREFGTT

TRRKRRIGWFDAVLAQKAAKINGLNSIAITKLDIFDKVDKIKICVAYELDGERVESVPYL

AEQFAQLKPVYEELPGWKQKTTEITSYDALPSEAKQYIQRLGELTGVEISMVSVGPEREQ

TITLMDLFKSKESV

>tr|D6YUU1|D6YUU1_WADCW Antitoxin OS=Waddlia chondrophila (strain ATCC VR-1470 / WSU 86-1044) OX=716544 GN=wcw_0531 PE=3 SV=1

MDTVAASEAKDKLQEMIKEAVRNRQQYKIIGNEGSVVLLPQETYDNILVTLELLSTPGLL

DQIKLQEVEEEFSKAFNH

>tr|D6YWS1|D6YWS1_WADCW 50S ribosomal protein L13 OS=Waddlia chondrophila (strain ATCC VR-1470 / WSU 86-1044) OX=716544 GN=rplM PE=3 SV=1

MTQKKRKIEQKSFFQKKEEVERNWFVLDAEGKTLGRFASEVAKVLRGKHKPTFTPSTDGG

DGVIVINAEKIAVTGAKEAQKSYIYHTGHVGGQREIPYRVMQARKPEYIIEHAVKGMMPK

TVLGRKQMRRLRIYAGAEHQLHAQKPVTANI

>tr|D6YT77|D6YT77_WADCW UVR domain-containing protein OS=Waddlia chondrophila (strain ATCC VR-1470 / WSU 86-1044) OX=716544 GN=wcw_1939 PE=4 SV=1

MEIHPRKENVHPDRPLECSECKNPITVHYTEIVGGKCTNTGMCDTCPQLQKKLKGIPFEE

HESPTKEGTGLVCGECGTTLADVKVSHRVGCTNCYSVFGDVLINELIAAHALFPGIVKQG

KSVPMHVGRAPGETHEISPSLKLIALNEALEETLKKEDYEQAALLRDQIKELTKESKKTE

DKND

>tr|D6YUG3|D6YUG3_WADCW Uncharacterized protein OS=Waddlia chondrophila (strain ATCC VR-1470 / WSU 86-1044) OX=716544 GN=wcw_0402 PE=4 SV=1

MKREEIHIMKKKDSSICWVIDFKKSDSKRSGTLLFEFYTPQEPSAFPKPKRKKSQYKALE

FLQQIQDSCESLKEECRETSESGQSESYEEQITRITEDIGYLFGMIKEFTQLMQKLQRPP

EDSSQKEEKNP

>tr|D6YSG1|D6YSG1_WADCW Uncharacterized protein OS=Waddlia chondrophila (strain ATCC VR-1470 / WSU 86-1044) OX=716544 GN=wcw_1662 PE=4 SV=1

MNQPIDSHIQLKQCGKSKKKLQKQLLLRLQQLPSLI

>tr|D6YU74|D6YU74_WADCW Uncharacterized protein OS=Waddlia chondrophila (strain ATCC VR-1470 / WSU 86-1044) OX=716544 GN=wcw_0310 PE=4 SV=1

MNIDFSYTHGASDWLHEKKANLFYSISDGYPKEQDNSYDGYKNVVGTKWTYLSGFPKGAH

CALLSIAARITAVGECLIKGVGNVFGSPFSKKFSVSTGVKQLGVDLPLSIFKLIFLMPVE

VIADAVISPFAVMIDKNYAEARGDFERNFFPDAWYDESSKSEFSEDEEDIDLRKSF

>tr|D6YVW2|D6YVW2_WADCW Uncharacterized protein OS=Waddlia chondrophila (strain ATCC VR-1470 / WSU 86-1044) OX=716544 GN=wcw_0911 PE=4 SV=1

MIPISSSCISYAFKEGFRLSLFNEKETLVKQTVLRCLKNSNIEPRKNRKRKRTYYAYSSN

ITISRSSEYPHKPGIRPSDSEFKQWRNVVIDHAYRRMQIFYYQLLFYFQEHCSVMQEKTL

SQHGKSSCCCCSAAHSAILPNLKDEKRLMSQKLHLYHTMNSTIEMPAIVNAVDCVIENQI

RSTALDLIHLTSLNNLHPYAALTLFAEKLAEFFALGKKETELRLSLLDKIEHLEEKIHCL

ETKEPAVPVDWMRNYARLVYDLQKCRKAFLENKNYTLPISLEKYDLSRRNIFSCICGSCQ

NEEKLLNSIKTHQQLKKYLSKREGFPSPLDKKLSKKKKVVRKFIASMHPVDLWLIPEKKA

LLLYQKVSRLLPLIKPISKTAISDARTQLNDSKRILELQEKGSVCPPFKYLSGHQTDGRR

LAYTIEQLLEQKKMLQTSD

>tr|D6YW73|D6YW73_WADCW 2-succinyl-5-enolpyruvyl-6-hydroxy-3-cyclohexene-1-carboxylate synthase OS=Waddlia chondrophila (strain ATCC VR-1470 / WSU 86-1044) OX=716544 GN=menD3 PE=4 SV=1

MTTLSNFFQSVSKPLVIVSTIPMKDQEVIADFLVRLNAPVYLEGVSGLRENPRLQHLRIT

YLKDICVDAVFRIGGVPTHRIWRDLEEKKGQLRLLSFSHLPWPGVSWGEVIEGDYQQLAE

ELFHYEKAWHVEDTQLKEISGKLFRQYPKSEPALIGRISSLIPEKSLVYLGNSLPIREWD

LAATNENRKYEIYASRGMNGIDGQISTFLGMTQPYRSNWCILGDLTALYDLAAPWILEQL

EPREITIVVINNGGGMIFSRMSERECLQNKHTIDFEYFAKFWKMNYFNEEGLSLKPEGVR

LIEAVPCAKQTNQFWNAYDKLLLNNYF

>tr|D6YUM1|D6YUM1_WADCW Thioesterase OS=Waddlia chondrophila (strain ATCC VR-1470 / WSU 86-1044) OX=716544 GN=wcw_0460 PE=4 SV=1

MFVGKNQVRMHDMDMAGLLYFPRQFRFVHDTLEDFMSQEGMPFETLFYEKDFMFVIVHCE

SDYFHSMRLGDDLEVHMVCKEIGRTSFTLSYQIYREDGLEVGRAKTVHVTIDKKNRKKIP

IPKLLKDVLMKHYSRDS

>tr|D6YWR3|D6YWR3_WADCW Uncharacterized protein OS=Waddlia chondrophila (strain ATCC VR-1470 / WSU 86-1044) OX=716544 GN=wcw_1217 PE=4 SV=1

MAANPYLGAPSNYVYIPLSDIYLDQIAKTNFSEGISGSSDTSKLSDPSQTQTDFPPSNAR

DGNSMVCLEQALLEAVGQGELGGIARDQKLAELRNQGIEIKGSLLKAGGDFNSPIVTDTY

TDSEGNRFYKFHVTWELKIKQSEGSDITITKRQWIITGVPQIDRLDDTSSVLFQHHQALL

AVKCHIFIQKNGFDKTQKQHDRILRCINDVRRTNLVGVQGFLKENRLTFTTDILDVDCKK

DPDCSYQLDSIISKKQPATALCIALRNASDKKVCVYIDQIFTGRKLNDNGTKYDKVPYDP

DHPTPRTIPLCVKRQRDAHGMIVRGNKDLSDHIRVLHAGPDELDELLSQSFEGRNARGMT

FAEVTKERYKARLASLENAQENVEQAFEQLKSMIKSLYGKQKGFGASLKVSLLGGKLEKM

AEKLDPQKLKADQCDQLTHAFETFQTAVSHLKTEDLCVYKLGSKCGMRLTAPKDSNYKKY

DDLTKLFQNNL

>tr|D6YUR6|D6YUR6_WADCW Uncharacterized protein OS=Waddlia chondrophila (strain ATCC VR-1470 / WSU 86-1044) OX=716544 GN=wcw_0506 PE=4 SV=1

MNLSEDLQEVIEKKSQKEGIDVKFEYRDRFIIP

>tr|D6YWE3|D6YWE3_WADCW Uncharacterized protein OS=Waddlia chondrophila (strain ATCC VR-1470 / WSU 86-1044) OX=716544 GN=wcw_1097 PE=4 SV=1

MMEIIAKEVGLRAVFNETVARKANIVHVKILKETSEDLL

>tr|D6YTI4|D6YTI4_WADCW CDP-glucose 4,6-dehydratase OS=Waddlia chondrophila (strain ATCC VR-1470 / WSU 86-1044) OX=716544 GN=rfbG PE=4 SV=1

MPQFPKDAFKGKKVLVTGHTGFKGTWLSLWLNKLGAHVIGYSLAPSDLFSLVGLENRITH

AQGDVRDLAQLSEIVQTTNPDAVFHFAAQSIVLNSYKSPQETFSTNALGTVNVLEACRRS

PSIRAIVIATTDKCYENRSWVWGYRENDRLGGKDPYSASKAMAELAASSYRESFLKGNIS

VATVRCGNVIGGGDFSPHRLLPDCFRALINQQSISVRNPKSIRPWLHVLDALYGYLKIAG

ELLTTGEIFSGSWNFGPFEKNAVTVQEMVEYAIETWGSGNWTDNSCKNAPEEMETLKLNW

EKAAKELSWSPRYCWKEAIEKTASWFKAYDTQSNMFETCMQQIEKYENDTSPARGRFSHQ

S

>tr|D6YWB8|D6YWB8_WADCW Putative membrane protein OS=Waddlia chondrophila (strain ATCC VR-1470 / WSU 86-1044) OX=716544 GN=wcw_1070 PE=4 SV=1

MKFTGILVIIYSLLVLSGGLIGYLMADSLPSLISGVAFGAALFTCGMGILRANVTALLIS

VALSGVLAVFFAYRYWLSMKLMPAGMMAIISSLIFLLLITTRARR

>tr|D6YW26|D6YW26_WADCW ATP-grasp domain-containing protein OS=Waddlia chondrophila (strain ATCC VR-1470 / WSU 86-1044) OX=716544 GN=carB PE=4 SV=1

MNSLLGEWFNLTEYSFVPNVFISSISKKIPLIQAARDALNRFSPNSLIIGGDLDPKCLGQ

YFVDRFWQMPSIKELTIHELIAYCQKEDVQFIIPTRDGELSFYAEHQMTLQKEGVGVMIS

PGTSVALALDKYAFSQKIERSIPTFLDACQLTFPCVAKERYGAGSRAIGLRLQYEQAVEH

GRQLENAVYQPYIEGSEISVDLYVRKDGGIHGMVCRERNVIVNGESQVTTTFRNEKIEAL

CISAAKQMKLYGHVMFQLIREAISGSVFLLECNPRFGGASTASVKAGLDSFFWFYCEAKR

RELPRCMRLEKEIKMVRHAEDMFIEL

>tr|D6YUE9|D6YUE9_WADCW Putative membrane protein OS=Waddlia chondrophila (strain ATCC VR-1470 / WSU 86-1044) OX=716544 GN=wcw_0388 PE=4 SV=1

MLPVESRDIELGIIPPSDDVLESSSAPLEERNQNFCGRIVKDWTQVVGGFLTLAGLVGGG

VGVGLEDYRVMLGASVVILASSVLLCARISCLKPEKELERQVAYFSGEVDRLTRNEAELK

TSRDELRKVLGEAETSIQELALAMKVPVDEIEGLSGKFEAIEEKLHVLIDLYHRYKAVAK

AFKDDLAIFRKSQKIADENLSRLGEETKKISSFEEELEEYQKAKEFHRQKNEELQEILSS

FQEDFVDVQKRFLLMRGELEELKQHVLKLDEVDDKFSSGGADFEEGIGQARSELLPKLKE

IVERLDKAVKELGDD

>tr|D6YV18|D6YV18_WADCW Uncharacterized protein OS=Waddlia chondrophila (strain ATCC VR-1470 / WSU 86-1044) OX=716544 GN=wcw_0611 PE=4 SV=1

MNFNLEVSFVQRGVENLISFRSRHESNLGKISTVFGGIGLGLAGLIETISSFALSLLTLP

ARLVGSDLSEKFYSRACSGINMTAISATLLQYYNIFGTARDFQTE

>tr|D6YWV5|D6YWV5_WADCW Uncharacterized protein OS=Waddlia chondrophila (strain ATCC VR-1470 / WSU 86-1044) OX=716544 GN=wcw_1264 PE=4 SV=1

MIVHYHPSGLFSQSRCSLENWKSFQYDHINDKNPKTNKTGIRCHHFIIALILRFFGKIHI

EKTKEGKSVYLNRKSFKKWIKWRGFSDQTATSTTTVEKIEQMRCHVVKTEPKNTSPLSTA

ASDFKQAIMDHCSEDLGKIWECLLACIPPDDIIQFESIEENTFTLKFSRPIALWLDSRSQ

DGTLSEPKGGTIVLLGHNERQTLEVLTDPLLKTIKYSTGMQFYCDTNTTGIGTKILNVIS

MSDKDEESVAIKAGCKVLFVFKAKEKIQPKKTLLKVWKQAAHLDREKDYRKYLERKMEGV

SEP

>tr|D6YSW1|D6YSW1_WADCW Putative outer membrane factor (OMF) family efflux porin OS=Waddlia chondrophila (strain ATCC VR-1470 / WSU 86-1044) OX=716544 GN=wcw_1817 PE=3 SV=1

MLRLNRLTSKYFSMLSFCLAGCAARDPFVLTPSTPCREWVYCLEGTPIPHCQLIDDDQLI

DEERVWHLPELVDLGLKNSRQTQRSWAETRIRASEYGLSLADFYPEISFSGYIEAVRATT

YFGSSRISELPGIQDIVVNEFREYAPSFSLAYLIFDWGTRNARSETFRQRVLSANWEFNR

EIQTVIRQITGDYYSYVGNKGLQEAAEANLKDAQTLYDATHKKYSLGIVDKSTDLIALTQ

VSKQQIQFLQAQQLLETSYAQLVADLGIPSTIELNIFGQFEAGEVIFPAECTVEQCVEEA

LMSRPDLFASYSGMQSTEAAVSAAKRDKLPKVSLQAAGSRIYYQDGENDGNDYGAMISLD

YPIFKGYWYENRIRSASSRYCKAKADFEQLQIEVVKEVVIAHRDLEIAVENLKVNRVYVD

AAAESYRATLMQFEAGVVDITTVVNAFTSLADARYSLVEAQKEWYTSIANLAYAIGILGK

GTR

>tr|D6YRS9|D6YRS9_WADCW Nucleoid-associated protein wcw_1424 OS=Waddlia chondrophila (strain ATCC VR-1470 / WSU 86-1044) OX=716544 GN=wcw_1424 PE=3 SV=1

MGKGFQKKKKQARQMQEQFAQLQEQMQNVEAEGQAGNGLVTIKLNGDFEIKSLRIKPDCV

DPDDVEGLETLIKAAHKDAMENVRKTMPSMGGGMPDLGSLGFGF

>tr|D6YVG2|D6YVG2_WADCW Conserved putative membrane protein OS=Waddlia chondrophila (strain ATCC VR-1470 / WSU 86-1044) OX=716544 GN=wcw_0756 PE=4 SV=1

MTVKSGKDWYRLFFYGACMGAADLVPGVSGGTMALICGIYEELIVSIKSFGTVDALSLFW

LDFKRFNQKVGWKFLLTVLLGILFSLVIFSRAIHFMLGDPVWRTYLYALFLGMILSSIVI

CIWRVPGWSKRLWWGLAVGAASTLFFTGFRSEPLLDQQTYQVKLPFKLGYQGDKQLVNYD

KETGLLKGLDAQILEAMWAKGYLNSDSMVAEQSSQRLLRLGDLMQKGEESWFDPWLFFCG

AIAVSAMLLPGISGSFLLMILGAYPVVIAALAELVKGWTAFTWDGEAFTVLINLSLGIGA

GAVIFSRAIDWFLKKDHDLTIAVLIGFMVGSLRVVWPYRTYAWGLDPLKLKNGPQLIPEG

FYQPSIWDAMYWKSAIFMVAGLGIVFLIYTMSENVKKR

>tr|D6YU04|D6YU04_WADCW Uroporphyrinogen decarboxylase OS=Waddlia chondrophila (strain ATCC VR-1470 / WSU 86-1044) OX=716544 GN=hemE PE=3 SV=1

MNTLLLEALACENQSRPPVWMMRQAGRYMPSYRKIREQYAFVEMCRNPEIAAEVTMLPIH

EFGMDAAILFSDILMIPDALGVGLRFEEGKGPIIEKPVKSCEDVAALKKPVVEEALGFVA

DAIRLLCNELSVPLIGFCGAPFTVASYMIEGGTSRDFKQTKQWMYRDPESFHALLALIAE

ATIDYVDMQVAAGAQAIQIFDSWAHVLGFRQFKEFSLFYMNAILEGICKPNPPVIIYCRG

ASVFAQELANIYPQAISLDWNAHLPSLRNLIPSRIALQGNFDPDLLYAPFSTIEEEVKRM

LCEMDGDPGYIVNLGHGIKPDMSPEAVKAFVDTVKAYSPITQAVE

>tr|D6YU14|D6YU14_WADCW Hydroxymethylbilane synthase OS=Waddlia chondrophila (strain ATCC VR-1470 / WSU 86-1044) OX=716544 GN=hemCD PE=3 SV=1

MPFHVSKGESKRPCTWDLNGASSPKETLRVFPPNYPNRSTSKELKLSSKPFPTLSTNRIT

VAARSSPLSEAQVDEILYEAQLIQPTLEFEVTLVTTLGDRDQESSLRTLEKTDFFTRDID

QLLLNGYCRIAVHSAKDLPDPIPNGLNIIAVNQGLDPSDSLVMRPGDTLSSLPKGAKIAT

SSIRREKAVHSLRSDLEVVDIRGNIGQRLQKLNDREVDGVIVAESALIRLNLYHLNRIHL

PGETAPGQGKLAVVAREDDEEAAVFFSAMDQRPITVYLGLKPPKDTAIKKFHHCPIIEIV

PKIRSTRPLPPFTHIVFTSKSAVKIFCCYFDPKGLMDKTVIAVGTQTAQGLIDCGVDVDY

MPEVESSEGLIALLETLNLQDAQIFWPHSALSRPVITDYLTNKQIPFTEWVIYTTIPREP

TPLPEEFHELVFTSPSTVDAFLLFFDEFPPDVKLTAIGPVTEQYLERVVKTE

>tr|D6YU08|D6YU08_WADCW Uncharacterized protein OS=Waddlia chondrophila (strain ATCC VR-1470 / WSU 86-1044) OX=716544 GN=wcw_0244 PE=4 SV=1

MSSFQSDFRACSHKYENHLSVGSENKQWDFAIGVKKGVVLSSDRSEGRSIIQQVSEDALF

HLSGARKRNAQILITSIEEKEVVPGYEPIAEPEDGSVEKDEEILKKRIKVRVIHYQLCVD

GKVEKHRTDEPRITNSVVKESNNHRVLLERFSRYDKNEEGAFARIKRALIQFFAQLFEPF

ESLSQLWEELCGRSEKEMVCDINLLAEVTGRPMTAEPLTLSQSLKYMIKLLSREKEVTAD

EKLHNRLKNALAISRKTKQLRKKFKRKDFGKLVKRVQADIRALPSDGSDKLLIPVGYKQD

GKLIEMLLEVGKTGEKTCSVALISQSKETLGLFDREEGVETLSRSLKREIHDVNISELQA

RIPIFMEMQTTPELLKFEEAGNVFLQQIHFPNSTVAVSKATEKTIDRSSSGHVSEIMSYV

KSELLSDGDVADAKRFETAARLRFFLDFCQRDKLWLRDPASRELVRTTAYQLIDIVETNR

RELIGDELLEQGLEMTKISHELERVLALFDETLPRTPDLSKKVALQTGLGKIDVEAAVET

PELEDIPMIADPFKEVDPPFTGFDPGKPVDSINAFAKRCEQLMEIGEIKRASYEAQTMVK

ALPPPGDALWNEIDPKAEEILQAFQTMGVAITKQSLQESHSSFNEALTLSMLNLYAYGII

SRKYPGKKEGLSVLAFKARKNLTELSNSRISLQDKARITQFDEIMKDAEIQEGHIYKLKG

DYEAILEGISQPLLNLCEYSAIAAGDLLYKTIQGKNVWNDSINYEEIDFETSQRVRSQLR

GKEPLDFSLDFNWNGLPYTRCEGVSEWKRDVSKFDSKISYTAPSYAKNVAEELANRYTTE

LCPLGCSKDNCENYGDTSGDPKEARKGHQYFPLYMLYHFTGEYCENLLAERKLTPDQVRD

LLLLQQTNRRADNLCSSHHKERGSRVGNHFHYTDGFTEDDRRAQVISSLNIYLKHPNFFK

IPSLRWFFETKLFNHHAFELLLSKENYEEHKPFLISTIKLLNREVSLAQINQEMETAAYL

VYLLGNIKDIIVEASSLNPDEKKLLSNLITVNPEKTIIQWMIDSIGQTSKREEKKQKSLL

PYVVNHYLELYNKNSDHTDQDFNLMAYAMERFQFLYEPGDSVDPKLKESFELLKCVMMPK

LRARAEKDDGSKFINGLILRFNPEIAGKKLDWKESQFPIYEASDAESRTYQFNLETGMIY

AGGERIERLPSFLKEEPEIVELYGSKLNDAWQIKGSPNLEDEQFRVTAYSHEKFPGQRIV

LRQKVDKEGIPLDGKPEVVIERMIEENSVSKWVSHHRFESQDKIAKGELLTGSDLPPKVA

AIIGERSCWVDRELDRIYVFDQEDSNPYATIFLGRDEGGGPLQIKEFRFADSNQLLAISG

RNLIQYSSIEDKDFILTAGSSGVVKRLEYPRLELASSGARLSYKITENGAVSSSFPGWTL

APLGLRPGIRRPIDRVVPLPETFDHFQLLQKDGAQKVLIPSRQFKQLRNRIGESIPKYQS

VCPEGFEPSAVYEFTVNTETNRLQAQSADAYAYLAYACFTHKDYESAKYYLEKAETSTGY

SPKYNELFDWIGKWPDNSPNGKAIKLHAAIFQDKIFEDHRLENIRRGGKKEEFEEPVQRM

ERLVELYHSYRTSLKDREIGLSGSDPGLDLTPDSELRALKLTREFIRANAETFVLQPKVE

ETKFVRALQNVEEIDLREPSEEEFFDYDLDALYLWACIGSSENIPEVTFGRPEWVLDHFG

YVFDQLMTLDPESVEFKQLVEKVRFISLNPPSEKLSPFAGDAIKIGQSYLLRVAELAKEG

KLEKLKNVVNGKFPKIKGSASTREKRFDNANNLAALLFDIDESTSAKAKKSLEERIGNVE

DRMSEVMEAIVKDGEHSFAEKMVVSKIARTEFESQLKMLRRMVKCCEDYDRRVEKIKQKN

ESLLLPSFEEEYQRVANEQLYGSGSHQTIQRIGSILQVLKEVEISSGEVEISSGEEKMQK

REVSDVEELPQKTVGDVYGELFENPPDDLKDAVESLLKGEIELYTQKAAQKKEIEKLEKL

IGETPEPEKIKKVVTPEEGKHAILGDARLHDYQRYYTVSSIDPSEISEEVFDRLAKSGEK

AVKRLADVYRKDMRAYKADLRAIKITRGQAKKLRAHLVDQKEELESRKSRLRKDLLQQVE

RFNTPAGTLMMRRLTGKAAKPNLDYLINLWRRGELTKQPWDENPLKQLGIGRMEEAELVK

LDVLITSYLDVSTTLQHMDNVVHSVDAYIATCGSRSSNEGDQQIAQALVQSLEAKRNYTL

FVPKRAITITDLAMVLHVSDIKKHLETQLDGKESRLAGQLRKLDPVKNKEDIIDLFANPD

HHKVLREILEEDKGLTNLVLSKGSDPDFRNLLFMEHTDRIILRSEQISTIREMIDDPNAA

RQLIMGAGKSKVLSPLLAYGKATGTNLVMLMLPEALYETNCRDLDATNRELFGQKIFRFE

FNRHSDRSVEALQETYIQLLETVRNKGFVPTTKSSMLSFRNAYFELLNQLSQIPLEQRYI

QEEKISEIMGQLRIMSKIMKLFHDRTDVLADEIDACLDVRKEVNFALGEPQKINPTIYQT

GSELMEILLNADEGTPLGELKTALLNNTNASIPPERLHELMKALGGAYYDENGEALGKIG

RNEFIQYLFDDDSLKGEIPQFIRSLEAQNPEMYQKVAAAKAFLHRGYGNTLSRIGNVNYG

RDPGSIWTIPYKASMAPSVGSEFDDTVERISFTLQDYVLYGVSYEQVYKAVAALHNMAIQ

QLRAANIDQTINIDETDAGKEFKELMKKLDLEGVFGKDPSLAAFAAEEKVEALKNVINQS

PLGRLSFASRYVLSEMTQAPNRVNSVSNDAPAMVRSFSGFTGTPWNLHTFHDKIQAERSP

GTDGRTWALLLERNVKVKTFVYDPKKPMESLIDGAGIVSEHYQATIDTGAYLRGQTNVDY

VTACLKKAEAEGLEGQSGVYFDESGKIVKKMGSEGKPLPIEVAEQGDLMQCHTLYDQAHT

VGADIKQGRKAKAIVTVGETTFVRDLFQAVWRLRQLHEEQDIDIVVSEDVKKLILGDEDN

RDLTIEDILAFCLTNEARRESEDNFRAEKGKIQGRPSQEILKSCAEIIVDEKNSDDDIHR

IASVLGRALTKRRPQDEIFDEYAQVRIQEDPSKILEKTRENTAQKAQEMAEALGKKASSK

LAGQLKKISKEIRDRKDPPSDWMPDQIDSAAVEGGQQVEVEAVAEVEMEQMVQLDAEVKG

EVAIQAEKVIQAGKPVSEGSGGTVSPVTKDNLIDLSLRGFVSGNLRQMSATIGAFDPEIY

VSSGVERQMVKSDHAEKYQVENSLDFDRVANTIFYTYRKTVNDVVIVKGQKGWRMIIPTI

HEAQSGCRTFIREANKKGWQAVQVNIGTAKPNIVFKTGDDRSDVLPFTEEEDLQKFYRLY

VQAKFFNGEINYGSEEEKEALRGWLRDKGPEDCQRLFETKILPAKPQRYTKNYTESDLYK

IFEELTG

>tr|D6YSG0|D6YSG0_WADCW Uncharacterized protein OS=Waddlia chondrophila (strain ATCC VR-1470 / WSU 86-1044) OX=716544 GN=wcw_1660 PE=4 SV=1

MDYPIIWKTRLGESKNDFLKVKEAARCMFSSP

>tr|D6YUV1|D6YUV1_WADCW Short-chain dehydrogenase OS=Waddlia chondrophila (strain ATCC VR-1470 / WSU 86-1044) OX=716544 GN=wcw_0541 PE=4 SV=1

MQRIIVVGGTGTIGREVVNVLSERHEVIPVGSQSGDFQCDLTSTESIEKMFSDIGTFDAL

ICCAGKVHFAPLKELTEENYYVGLQSKLMGQVNLVRLGLQTINEKGSFTLTSGILNREPI

ASGTPAAMTNAAIEGFVKGAAIELPKGCRINAVSPTILVESYERLENYFRGFIPVQAYNV

TQAYVRSVEGSQTGKIYHVDR

>tr|D6YSJ5|D6YSJ5_WADCW Uncharacterized protein OS=Waddlia chondrophila (strain ATCC VR-1470 / WSU 86-1044) OX=716544 GN=wcw_1696 PE=4 SV=1

MKSYLKILFVAFFIVQAVFVLNLKAENGDKIDLSPIFACKDCK

>tr|D6YRY3|D6YRY3_WADCW Glyceraldehyde-3-phosphate dehydrogenase OS=Waddlia chondrophila (strain ATCC VR-1470 / WSU 86-1044) OX=716544 GN=gapA PE=3 SV=1

MSINVAINGFGRIGKLVYRIATSRDDINIVAINDLVPCENLAYLLKYDSTHGRFDKELKI

EGNSFLVEGMKTIILNEKDPEQLPWKELDVDYVVESTGLFTSPEQANKHLKAGAKRVVIS

APAKGEVPTFVMGVNHRTYNPETDRIVSNASCTTNCLAPITKVLLDHFGIEEGLMTTVHA

VTATQPTVDGPSKKDLRGGRSATQNIIPASTGAAKAVGLCIPEVQGKLTGMAFRIPTADV

SVVDLTVKLSRSTSYGEICSAMKQAAEGEMKGILQYCDEPVVSSDFIGSSYSSIFDKGAG

IALNDTFYKVVAWYDNEMGYATRVVDLLAFLASKEHALAG

>tr|D6YWR2|D6YWR2_WADCW Uncharacterized protein OS=Waddlia chondrophila (strain ATCC VR-1470 / WSU 86-1044) OX=716544 GN=wcw_1216 PE=4 SV=1

MKWIKQAIFILFAGFLLFGALLQLDSVRSRMKTFLIESIESGTGYTVQIGQIRLFPSFQM

TAKDIVFMDGEVPLAAFNHLHLGIYPLDLLKGKLRFSTLHIEGVDVLNLPKETLKSEGKN

STISMIAIEDFFIEKIHWSIKGINPPDYPISLKGSLIVDQQHDLIYSNFNVFSPQSSGVS

GDLRYEKKNGTLLLQQEDGFRASLAFVMDESRGVRVPRFLATWHETTIDGKFSVTQTGGI

EDSAFFLSIDDLSQMVPISGALLGEAVISGSINSPKIDLSLSSNQLSGFGQTVEKLKLHL

STSGKGSFSLAFVKNEQDYMFSSALTWDQKTPWFPTQLDLRLPLEKVAQLANWDVADIDG

QLLINVRYQNQTLTFKAELLEALVESFSMGSRFINIHGIIEGDLKTLKLVHFTAKDTDNG

SYSGNGRIELDQKKNFPFNFVFQLEEAKPFQSDIFKSATSGELTFKGNLSKAILKGDLSG

SEARVQIPEKMPESSDSIKVTYINQPEDEPPPTKASIFSLDWPVELDVNYTIADQLTIKG

GNLSSTWIGKVNIGGTLDKLKTKGEVKLDKGRFILRGKTFEFVQGAITFNGDPEKNTHLY

LSAEMDLIDLTIQVVLNGPILNPSITLTSSPPMSQRAILSWLLFGKGISDINPMEETQLS

RSLRSLLDKVGDKPDVLTRMGGVLGLDQVEIGANPGGETGDLTVKVGKYLSEGTYLSISR

NISRGKDPDKNTSCFGIETKLGRHFRFRAEGDTETNGRLNLLWKNDY

>tr|D6YRK5|D6YRK5_WADCW Uncharacterized protein OS=Waddlia chondrophila (strain ATCC VR-1470 / WSU 86-1044) OX=716544 GN=wcw_1349 PE=4 SV=1

MNLNLKFSPIATSEQGLLPCLAGNLNFREIDSV

>tr|D6YWN1|D6YWN1_WADCW Holo-[acyl-carrier-protein] synthase OS=Waddlia chondrophila (strain ATCC VR-1470 / WSU 86-1044) OX=716544 GN=acpS PE=3 SV=1

MIKGLGTDIIEIDRIEKVFNRYGQKFLDRILSKSEQEYCLKYKNPVQHYAGRFAAKEAIV

KALGTGIRKAVSWTDIEILNNNQGKPQVYLSPEVRSHFSDPIIHISISHSKKYATAVAIM

EK

>tr|D6YU35|D6YU35_WADCW Putative membrane protein OS=Waddlia chondrophila (strain ATCC VR-1470 / WSU 86-1044) OX=716544 GN=pmp PE=4 SV=1

MKKRLLLIIPLVLLAFAGIFRHAILGNVLTYTANRYTLNKFGVPLEFEKISKKGRLWEIR

SLAIKRSNLSLTADKLEVAFEWHPLGGYFFVKILVRDSEIAINEQAADFSSLLADLLPNR

LPIWMLNVQTVCEISNGRVSWIAKGGEAESAVFELQASSGGSRQDLELSVWLDEEVRGRN

AFCLSLTKDDFKGLSTGLTFYQVECGKVARGFEALGRPLKGWEIQRGMIDGNVELAWPSS

HAPYAIGKASVTDLRFDHPESKTTGEVREAVLDLHMRHIKGEMPSLEGKVEFTGGTSLAF

QRKGEPFWKIDNLSGGLYFEPERAVMINMGGKCSHELCDFCLSLDGKAQLVDKQKASLDL

AVRLFSEGGKEAAVRFIAEQLGPKEHQAELFVENVGPAEFAFVQEALARYSDDWNGFHIQ

KGLLDAEGLVSMVGWQLTDLDIRQLKVEGMEVDVPSLDLSANIEKVTGGLAIHLLEEEPL

RTIDADFFIDGGCLVSQTLSGSLCCLNDVRTELKVRQGVIQQSEMRGEFAGLKGTVGIDW

TAKDKIVQAYFSGPSERLAPFLPAHFRSPFLQSFSSNDLEIKAGVVLKNEKLAVKGEARM

AEQTVAFGFHLEKSSEKLWKSWPANHLAASYWDNVGAEVMKTVVPPIASPAVLFEANWIR

AETGIAGLVLRNGWFKAKEVDLERFVAPFLFSSSQMQLSGRGSFIGGFNQNVLTVEYEAQ

NVVMGNRFMEINAEHIDRGVHYFDFKKGVHFGSIPLSQAVYTEKNTGLVFTPVDMQVAVE

GEKMHLAQIDAECCGMRMAGEIDIDFGKPDDGVFDLEIRVRNVKGGLKNVQDLLARFHPD

LWLLQLPVDGQFELKDEGAFFKLSFTPEDYAVEAVLKGKIQEAFVASGPYGRGGLSGLQL

QFGYDHLNRLLEISHLQADIYVDDQFTGYRLYGDHLSVDDLEEGRSSFDFWVGDQNRDLI

RFVGRTQQEMDPYERPYIAVHIDHHLTHLGNIHPSSFLLRLKSPTVVDQFQIGMQVDLEA

LFNDLFAVSCSGGGSALEQLGKEIQWMHNPAGTLDVQLHYDLRSNSLIYRIDGSGIEMNQ

LTAKKMKLAGSKSQSKWIVDQLQWDEKVIAGEFMPEGERWKIEGFQFRDQDILALNFDGY

FIPARKKLDARVNEVQIDLAQVSGTAEIEQFRRENDPHGKLTGYGNLSVEWGSSRVPWKF

DAILDVALEQWDLKGIRFDDAHNISCHYISGQGMTMRKLRTKMLDKASTEVLAQVDIEKI

QYDFSTGEVLFDELAFAVPADRLPRFSAQLERSFPNFVTPVMAEIICSCKQEGVLKGVMQ

YELTPPYTAMKLTLEDGIYNFLNAAHKISGFVMDYDPFEFSVVSGYRLAGRDVWLYARSS

SPSLAYGELVLTDQDPEKVSQQRPKEALYFDWENNSETGICFTHIEGSYQGLQMELEKDP

TQAPSKEALFLVGTVGIHGQKAKEFFPEQMADKFVAWQVGEGYSLQGKWRFLKNYSENYG

DKLHFVGMLKGSDFHLKGYQLDSLQAHLEYTPTAIRINDLVINDRCGVLTSDRIDILKTD

LGTWAFAMPLMIVNQFRPSLLQEEGFPRPTARKPMVVQELILEKTQGNLSDSRTMIGRGS

LYFTNRSKKFLQDTIFQIPSDILSRIGLDSAVLTPVSGTIEYEIHDGRIYLTRFKEMYSD

AKLSKFYLASSPSSTVDFDGGLNVQVRMKQYNLIFKLAELFTFNIQGDLKNPVYSIQKQ

>tr|D6YSR0|D6YSR0_WADCW Uncharacterized protein OS=Waddlia chondrophila (strain ATCC VR-1470 / WSU 86-1044) OX=716544 GN=wcw_1764 PE=4 SV=1

MFFHRRTIPGLAIHSYLIGNESSGECAVIDPVRDVEEYLQLTKENGLEICYILETHVHAD

FVSGSKELKKASKGKAQIYCSGLGGEAWTPSYADCVVKDGDEIAMGNLVLKAIHTPGHTP

EHISWLLSEDGEEIKLFTGDLLFVGAVGRPDLLGKEEMDRLSHQLYDSVFKKLSQFSDFV

EVRPAHGAGSLCGKALGSAPYSSMGIERSHNLYLKEKPENEWINALLDEMPLAPHYFTRM

KKVNIKGAEFVEKVTKDLKPLTVKEVKEKSAEGAMFLDLRSKEAFSSAHIPGSINIPISP

QVSTWAGWVLPYPTPVVLILEEDEHLEPAVTQLLRIGYDCILGFLKGGIREWETSGSKLS

ELNILSVQDLIKNLGDTFVVDIRTKSEWDRGHIQSAHFIEGGQIAERINEIPKDRQVAVI

CGSGFRASIIASLLKRSGYDKISNVFGGMQAWIQEGYPVSGQ

>tr|D6YWU9|D6YWU9_WADCW Uncharacterized protein OS=Waddlia chondrophila (strain ATCC VR-1470 / WSU 86-1044) OX=716544 GN=wcw_1253 PE=4 SV=1

MLNKKDGEGNTALHLQKNLSIIEELVKKGAQFLPNSSELFPLHTCQDPKIAKYFIDRFKG

EMDILNLSNDDMSAPIFTVPPAVVRVLIEEGADVTVEDKEGNTILFRFTEEPFSSDPQLL

EWILSKIENADVKEKFIDLICNEYSILNTLIQSDQANVDIVKMLLNAGASYPVLHEWNSP

LQVVKVPEVAACLIDHYGKDIMERKNEEGVSPLIQAKHNLEVFKIMIEKGVKVTEAIDSG

GNTILHYFSESPLKDSPELIDLVLEKIEDADAKAIFVNQKNLERMLPLEYTLPLQAIKAL

VEAGAKCPFVRSLSFYKDPEAATYLMEKFRDQFNEQLTHILSPLLVEKEDGSIALSELCS

SKGFEVLKALIEGGLNVHTTDGKGLEPIHYLKKEDVELAKLLIGNRRSDHDWKGNNPSIM

DNEDFVNWLKDREKDI

>tr|D6YTT3|D6YTT3_WADCW Macro domain-containing protein OS=Waddlia chondrophila (strain ATCC VR-1470 / WSU 86-1044) OX=716544 GN=wcw_0169 PE=4 SV=1

MKLDFSNFFTHLIFHPFDADTKNERKFALVSSIALGIFSLFTVHLGCLIYGFFARKVDHP

SPSDESVSKVAKEHGIGTQKPQAQKFEPVLLAEKTFGSARLQIYQGDLLEQKVDAIVNPA

NTKLRGGGGVDGIIGRAAGKSIYDECVEQLKAKKLDSCNIGDAYITGSGKLNEKGIKHVI

HAVGPTGSTPGGDQLLKAAYMNSLLKADEKGLESIAFPAISIGIFNFNPKHAAELAFEAV

RDFFSEHPDTSIKEVRLCYWTSSKDVASKNRMLELLS

>tr|D6YWS9|D6YWS9_WADCW DNA repair protein radC family OS=Waddlia chondrophila (strain ATCC VR-1470 / WSU 86-1044) OX=716544 GN=radC PE=3 SV=1

MTIRSLPDQEKPRERLQRLGADALSSTELLAVILGCGTRGCSVLELSKELIARFGGLRHL

ADATIEELLQMRGVGTAQAIKLKAAFSLGKRAYHPGQLRRYRVQTPEHAYAYVKDFLLQE

TKEVLISLLLDTKGDLITQKIISIGTLSHTLAHPREVFFDAVRHKAASLVIAHNHPSGDP

TPSRHDIEMTAQLIKAGELMGIPLQDHLIIGSRTFVSLREIGLFNKEAIAK

>tr|D6YUL3|D6YUL3_WADCW Uncharacterized protein OS=Waddlia chondrophila (strain ATCC VR-1470 / WSU 86-1044) OX=716544 GN=wcw_0452 PE=4 SV=1

MGAGCYLKTHEPIPILLFVQMFFLFEKFSSTASL

>tr|D6YU10|D6YU10_WADCW PTS system IIA protein OS=Waddlia chondrophila (strain ATCC VR-1470 / WSU 86-1044) OX=716544 GN=ptsN2 PE=4 SV=1

MVKISDYLDKQFITFLGVETRDEALLAMVEVLAASGKIDHKDQFFQALVDRETIVSTGIG

MGVAIPHAKLPSYDDFFIAIGVLKNGVDWDALDGAPVRLIFMIGGPDDKQTEYLQILSGI

TVAIKDEERRKKMLTLKSPTSMIKLFKDF

>tr|D6YTY5|D6YTY5_WADCW Uncharacterized protein OS=Waddlia chondrophila (strain ATCC VR-1470 / WSU 86-1044) OX=716544 GN=wcw_0221 PE=4 SV=1

MKSPWPYQIIHSVLQAVKKINTDKVGIAKTYLVKGNIREIKSRLDKQYIVKSCSSMRSIA

ATEQDYNSWEMQDLKNVPVMFQEYIDGTDYRIHFMDDYTWTLSVISKDRADYRYSSKHSL

EYRLETIPSELDDFCKDLSKIEDNRFIGVDLIKTGDRYVCLESNPGPDWSTFYHPSRDQF

NHFFIQTLAQEEKGYHV

>tr|D6YUU6|D6YUU6_WADCW Nicotinate phosphoribosyltransferase OS=Waddlia chondrophila (strain ATCC VR-1470 / WSU 86-1044) OX=716544 GN=pncB PE=3 SV=1

MNSLSPFRYHQSLALLTDLYQITMSYGYWKNGLQDKEAVFHLFFRKCPFRGGYAIAAGLE

LVIDYLNNFRFDDSDLNYLAGLKGADGNPLFEEGFFDYLSKMEFSCNVDAVPEGTAVFPY

EPMVRVSGPLIQAQILETPILNLINFSSLIATKASRICAAADGDNVLEFGLRRAQGIDGA

ITGARSAYIGGCSSTSNVLAGKLFGIPVEGTHSHSWVMTFEDEQDSFESFADALPANCVF

LVDTYNSIEGVKKAINVGKKLKERGKRMLGIRLDSGDLAYLSKVSRQMLDEAGFEDAVIV

ASNELDETIISDLKKQGAKIAVWGVGTNLITAKDQPALDGVYKLSAIRNPGEEWQFRLKL

SEQMVKISNPGVLQVRRYFNQKEYVADAIFNEGDDLSSGCFIVDPLDSTRQKRIDPNLLH

KDLLVPIFRNGKCIYTLPLLQDIRKSVRSELSRVHEGVKRFIFPHQYVVGLERSLYDRKL

ELIKQIRRIS

>tr|D6YWH1|D6YWH1_WADCW ATP synthase subunit delta OS=Waddlia chondrophila (strain ATCC VR-1470 / WSU 86-1044) OX=716544 GN=atpH PE=3 SV=1

MTKASTKYAGILFFLALKKQELETYLFQLQEIKKITSNTHLQEAFASPLVPLQAKKNVLE

LLFKDKVKEEILLFLKILTEQKKISLITEVIEEFSIKMKKQMGILSIKLISAEKIDEENK

LLLQNKLEAKYNKKIEFYEEQSSKIIGGMILLFPNGKILDKSLKTCLEHLRKHLKKGKRH

AA

>tr|D6YVN7|D6YVN7_WADCW Arginine N-succinyltransferase beta subunit OS=Waddlia chondrophila (strain ATCC VR-1470 / WSU 86-1044) OX=716544 GN=astA PE=4 SV=1

MHNSRRDAAMFVIRPAKLEDIEALKEFAENAALGIISLPKNPELLKKKIAFSLSCFDKQV

QNPGNEIYCFVLENLETGEVGGCCAIYAKTGIKEPVYIYRLETSYPSSKTLPVPCQITVL

KPMKLSEGPAELSMLFLKKEWRKEKLGELLSLSRFLFIANHKHRIETTICARLRGVVDSK

KNTSPFWDGIGRHFVAIDFSKVCELQQEDYPYIEEFLPRFPIYTHLLSKQVQAMIGRIHE

TTRPALHMLKKEGMSLSSDIDLFDGGPILQGDISTIRTVVKSRRTTVAKVDTVPINSPLF

LISNCLLDFRCCFARALFKEREGITLAYEDAKALQVSPGDDVRFIPNKLQE

>tr|D6YVI3|D6YVI3_WADCW Uncharacterized protein OS=Waddlia chondrophila (strain ATCC VR-1470 / WSU 86-1044) OX=716544 GN=wcw_0777 PE=4 SV=1

MQVRIHNKETIDKVCWPETDNGKLARQFLVPMIKEGTRRYFKNVETELRLLEIDKIFLPI

TINDNVAPNAYVCSTYTHYVSYALEEIERVKNRWLQKLAKPVIRLFGAFLKTGKVDKTVF

VNNWLLPTNLYPSLTQDQLSRITKALVNHFPKHTIAFRSVNDHAPGGLKNHLRSLNYDFL

LCRDIYYTDTQSPEPFKARMTKSDLKLLNNTEYSIVENDKIPEFSLERIASLYQMLNIDK

YSACNPQYTSELLNLMRSISGFNLKAWMKDHEVHAVLGYYTQDGIVTSPLFGYDTSLPQE

TGLYRQISAALLKDAKNNAHFLHQSSGAGHYKLLRKAVKDLEYTAVYIKHLPKARQLPWK

ALLVGMNKLGKNFL

>tr|D6YT59|D6YT59_WADCW Uncharacterized protein OS=Waddlia chondrophila (strain ATCC VR-1470 / WSU 86-1044) OX=716544 GN=wcw_1917 PE=4 SV=1

MVSQYDCLLPEECLQIQQQLHSLKDKWEPRLGDLPFYTLGAASYLDAGKKGEFFYKMKAK

RFNPLLKEHFDSLYQKVAEVLEKETGKPVVYEENYGYPGFHIFLFSEVFEFNIASVHFDL

QFQELSWRYKNVDQLHPLSITLPIALPGSGGGMFCWDIHYRDVKELPRSELEKRSQEEEP

VLIEYSPGQIVVHEGLFLHQIAPAEEMKENDERITLQGHGLFCDGAWHLYW

>tr|D6YWY3|D6YWY3_WADCW Uncharacterized protein OS=Waddlia chondrophila (strain ATCC VR-1470 / WSU 86-1044) OX=716544 GN=wcw_1292 PE=4 SV=1

MRTFHFLIVLCISSILHLSAVEEGDERQLLPDISYLVADLKFNDQQGVQICEVQQGRGSR

FIGYDFAHEGTGLIVRNLAEALKEYHNRGWFLKNDLCDPLTAGALTAIGWRGFETIEELI

NDPEFQLVASLNHEKAVSISDFGGILFARHSSLNPFAQFKEKYPNVLIIDEATRSYCGDK

LRMTLLFTKAELERFKPSWNLYPKEYSPSLCNQIMEEIASEYVVIKPRRAAKGNGVIICS

SSELDETLDYILNGGENLFDDPDPSYRYWAYDQSGSFIVEAYALSNPTPVEHLDNRLFDP

TMRVVYLLAHSDDTIHLRFLGSYWKLPEMSLEQTGSLNCLHKSCGKIPYFSVVDPDVDGE

VQRQIHDCLHQLYESMLKGEQKLLLENTP

>tr|D6YV09|D6YV09_WADCW Putative secreted protein OS=Waddlia chondrophila (strain ATCC VR-1470 / WSU 86-1044) OX=716544 GN=wcw_0602 PE=4 SV=1

MKKSFLSICLFVLTVCLPAQAAVEKPLEEPGTVTFVPPKNWRMVDAKQLKGNVRFMVIGK

GKYEFPPSINLSTEEYKGTLREYLAIVKEINSSQGSEWKNLGMIKTEAGDASLSQADAIT

EWGPIRMMHVILLRDDVIYILTAASLKKEFPTLYQTFFKSFKSLKITNIAPHH

>tr|D6YS80|D6YS80_WADCW Uncharacterized protein OS=Waddlia chondrophila (strain ATCC VR-1470 / WSU 86-1044) OX=716544 GN=wcw_1577 PE=4 SV=1

MNKDNIRLGREYLYQQYNNSQYESHVKPAIDRYRDLSDLFSKNMDLCRNGAKLFYDSKSH

KFFQGFGNCSAQNEFVWRRDHILYRIREFSAALFGSDAEKQECVKAYLYHKLGVQSEQDY

IHSGEVNDDKIWKLFGKLEEKRFLTCEEKERKQAYLASDEYTIKMLTEELEEIQKGIQEK

EEYFFQTLINEEMLNGIIKRIEERLKKNISPNGRESLQNVLQAIKEIRKGRENNTSLSEI

KFQMLFENGNNYRNCLNKYDDIDRIYECLEELDEKFLIRRNEINEQLSDLQEKPLVHDAP

DLDLVEDLVDDHQLLGLNGAQKIEEEEELNDLQEKPLVHDAPDLDLVEDLVDDHQLLGLN

GAQKIEEEEELNDRREDPVEVNAERKKKISWFSGVINAIGGFFRSAWNGFLNLFFTDKV

>tr|D6YRQ0|D6YRQ0_WADCW Quinolinate phosphoribosyltransferase [decarboxylating] OS=Waddlia chondrophila (strain ATCC VR-1470 / WSU 86-1044) OX=716544 GN=wcw_1395 PE=3 SV=1

MYLQKEIERLIDISIEEDLRSGDITSNILVPPEAITSGRIVLKQAGVLAALPFLQILFQK

IDPSIEVILSVDEGSYQKAGTTIGKVTGPARGILSGERIALNLIQHASGVATTTSEYVRK

LKGLHCEILDTRKTLPGLRALEKYSVNIGGGSNHRFALDDRFIIKTNHLYFLKGTSKYPI

KDAIERVKKHRSDLSIEVEITRYEQLEQALHPYVNGIMLSRMMPIEIKRCVDRIHQTDKK

VYLESLGTITLDTVRAYAETGVDGISIGSLTHSVPALDIVMRFK

>tr|D6YVW0|D6YVW0_WADCW Uncharacterized protein OS=Waddlia chondrophila (strain ATCC VR-1470 / WSU 86-1044) OX=716544 GN=wcw_0909 PE=4 SV=1

MVYFQFMILPMTRSFYFLLLVAFFAWKQAGAQAILPYENGPLHEAFATPISINLLLDAIE

LEPPQSINERIPKQLDIQAEWISGYWQWDFNANDFVWVSGVWRRPPPGHQWVSGLWKKYG

SEWVRVPGYWSKVPEQSADFISVPPPDPLDENTASPPSSNVFWVNGHWYFMFNLQEFHWV

PGHWDEFDPQWVLVPAHYVWRPGGYVYVPAYWDWPIEERGTAYTSVKIDPDYRYHVVFEP

TSILKSEIIVKQLFLHYPDYLCFFHHHYHYHLDFWKAFCCYPPWWGWDTWWGFTWHDHWA

LWWWYTHPGYPQPLWMTKEISSILPVPLSDLLSMFSYVNAPLIVTPNGVASRMTFLRALN

RVVENDLPVVPDNEKKLKRIQELAKPVSIDPYNILKPLGPRLPIDPNAVRPNVRKPVVNS

QPFSGVQIIKEDLKPHFPFKPRIPSHRSSPIWKPQESSSTSEPLPVPKPTWTPKSPNSYP

PVQTYPDKKKPSDAESSQWKPRHHWGEPPVQRPPIYRPRNRHSSVKTWKEMDERNREE

>tr|D6YVF6|D6YVF6_WADCW Uncharacterized protein OS=Waddlia chondrophila (strain ATCC VR-1470 / WSU 86-1044) OX=716544 GN=wcw_0750 PE=4 SV=1

MPKEMEEKIVSIRQMISCFGPKRIKHFYDIPYSLGAIQRVIRSHGLTRKRKKTYQKRRDM

RAVKAKRASMC

>tr|D6YSL5|D6YSL5_WADCW Transport permease protein OS=Waddlia chondrophila (strain ATCC VR-1470 / WSU 86-1044) OX=716544 GN=ybhR PE=3 SV=1

MLRKLHALIIKELIAVWQDKRSRMALITPPIIQFFVFTFAATLDVHNISLGILNRDEGQY

SREFIYRLSGSPEFTQIIYFDSDQELKEAVDAEKVLAAIQIDANFSRKILSDETGEILLI

ADGRKSNASQIAGGYISNIAQAYNAFLAKRKGMPLPLSELKPRNWYNANLNYTWFTITGL

IGTLSMLTSLSVTALSISREKEMGTFEQLLVSPLDHRMILLGKAIPALMIGVVEGSVMLL

AGFLFLDLPIRGSLLMFYPSMAIFVFAIVGIGLFISSLSQTQQQSALGVFLCVSPLVITS

GFATPVENMTQGLQILAMLNPLKYFLIIVRGICLKDISAGTVFHNTWPMLLIALGTLGTA

SWFFKRKIG

>tr|D6YRI4|D6YRI4_WADCW Uncharacterized protein OS=Waddlia chondrophila (strain ATCC VR-1470 / WSU 86-1044) OX=716544 GN=wcw_1327 PE=4 SV=1

MTSTGGIPPDGGSIHWQSSSTSGSDETERTTRAATFKADGKEYTVKVSYSRQAVKEKYGI

SDDAVDATMEEVISKIGVEQLKNLAGHSISASLRGKGGEQIQYKANKDSNYQIFSDALKT

KNLPLFQTISTVRTIFQLNTGALKRVPSSSGEVELDIKEETEKLRDSSIRFKEVPNEVFA

FIEHLSSTNPIASDLEKIKKIIEADSADSADEVKKILQDPKLNLTKEQQNEVLKQLKDVD

FADEIRVAIGLQKEGDVFLDVSQGGIQIENQPEEVGKMACASICGQAILWMESNNEYLVT

REDFEQVIKDGIDRHVGKERVENPPEGKEFNGVFKGISEDFESEDYKFEHSFITEHFDDE

VELMTSSDYLVVTGYSSAWGEELGSILLFKVDDDESSAQWGVMDSHGLTENKDTKDEITR

GASLRFFDTAQKALDYVKGHVKMGEEDSARVDKVSVVDRQS

>tr|D6YSN6|D6YSN6_WADCW Uncharacterized protein OS=Waddlia chondrophila (strain ATCC VR-1470 / WSU 86-1044) OX=716544 GN=wcw_1740 PE=4 SV=1

MKAFVEGGLNVHTTDDEGLEPIHYLKEDDVELAKLLIGNRRSDHDWKENKPPVMDNEDFV

NWLKDREEEDVKEEEIV

>tr|D6YV97|D6YV97_WADCW Uncharacterized protein OS=Waddlia chondrophila (strain ATCC VR-1470 / WSU 86-1044) OX=716544 GN=wcw_0691 PE=4 SV=1

MNWEKIHELADQMVENQRSHLLKLGRQVIPSLTSEDVLQPNDYPELEENPVFRYEEGILA

GLQGLQMALKSLDKSA

>tr|D6YUW4|D6YUW4_WADCW Putative transposase OS=Waddlia chondrophila (strain ATCC VR-1470 / WSU 86-1044) OX=716544 GN=wcw_0555 PE=3 SV=1

MNPSCPKCESTAVKKNGHIHNGKQNHRCLVCGRQFVLDPQQKIITDQTKSEVRQALLERV

SLEGICRIFSVSMPWLLSFIQQIIYELPDDLNATVVKDYKDFEVAIIELDEQWSYVGNKK

NQQWLWLAFHLASRQVLAMHVGKRDKRAAEALLAKLPEDLKKKPSFTLISSLCTTKSFLG

SNTRQSEKIQEKRVTLKDLTTP

>tr|D6YT32|D6YT32_WADCW Uncharacterized protein OS=Waddlia chondrophila (strain ATCC VR-1470 / WSU 86-1044) OX=716544 GN=wcw_1889 PE=4 SV=1

MIAETENLFNKDWVMLIDKEINRLLQELSHAEQAQIYLNGFAVSVHMLECGSRLTLSTPV

YCGGNYIPNSVRESLRKKAPFDRLDIQTFLEIDEEDFSIMLIYQGECAPSGYGGIVDLLE

DFSWLAEKWRDYLDEQDKNDLIYVTKKL

>tr|D6YVT0|D6YVT0_WADCW Putative membrane-associated protein OS=Waddlia chondrophila (strain ATCC VR-1470 / WSU 86-1044) OX=716544 GN=wcw_0877 PE=4 SV=1

MIKPNLSITNNLSQFIQKKHESKDFVQREIVTRVAGVALPIFSLIDVVIHSAGMLESCIK

GIAIKPGDFTDFHRHSKCVKIFLRLFIASSIYGAFIPSIILETFEDGTIPCVNVLLLSKY

PEYSENCVVNTSKIVKYIKKHVHQMKEEDLQGMENTLKLLGDAEKYLSRKNLQEIKKMTQ

APFTHGLTDLIRKLRTAQGSFVKTAFFRHCLTRLLSLGLSLTCALDLIFNTFALSFILLA

AASGKLLIGKSFDISKTDALNYIITLFRDQLVHLAGTLTGSIVGLIDPDTALHLTDTRSS

WYQKFQCKGERYTEAMLEDLERMQEGESLLIPLSFQTEKNGHIVYCLTDKTSSGYNFTTL

NTGYGSNYAEIKLKLITCIKNGLSKEEAEALRRNGISKELDPREKHLANYTFKHLTFSQL

SSHVRAIHEFAELSFNEIQKKANEKKISTDQYTYETIYPSIISSDEGKVRPEYGKLDSQG

FPDLTFNPQLMFSSLQTIGDCPKSSLLSALNYHSSHRSKEPNHQPFDRWIRKLQDKVLDQ

DGYLLDISFILKNHNRQAKKTAKTHLEERWRSYTSKYLMA

>tr|D6YVC5|D6YVC5_WADCW Isocitrase OS=Waddlia chondrophila (strain ATCC VR-1470 / WSU 86-1044) OX=716544 GN=aceA PE=4 SV=1

MYTKEDVASLEKEWETSPRWKGVVRQYSAEQVIKLQGSIAIDYPLARIGAEKLWNLLESE

TFVRALGALSGNQAVQMVQAGLKAIYASGWQVAADANDALQTYPDQSLYPVQSMPHLIRR

INNALKRADQIQHMQNKVNGDWFVPIIADAEAGFGGNLNTFELIKAMIEAGSAAIHLEDQ

LSSQKKCGHMGGKVLVSGDAFIEKLMAARLAADVMGIPTLIIARTDAHSAKLIRSDSYSV

DRPFLTGERSVEGFHYIEGGIPYAIARGLDFAPYCDLVWCETSTPDLGEAREFAQGIHEK

YPGKWLAYNCSPSFNWKAKLDDRSIRDFQEKLGEIGYKFQFVTLAGFHTLNASMFDLARQ

YKEEGMAAYSRFQEREFELERQGGFSAIKHQQFVGAGYFDMVTMTLSQGQSSTTALAGST

EEEQFNL

>tr|D6YU80|D6YU80_WADCW Elongation factor 4 OS=Waddlia chondrophila (strain ATCC VR-1470 / WSU 86-1044) OX=716544 GN=lepA PE=3 SV=1

MYTYDIKKIRNFSIIAHIDHGKSTLADRLMELTQTVSKREMQEQLLDDMELERERGITIK

SHPVTMFYQAKNGEFYQLNLIDTPGHVDFTYEVSRSLAACEGALLVVDAAQGVQAQTLAN

VHLAIDRDCEILPILNKIDLPAADTEEAKRQIEEVIGLDASHAIGCSAKTGVGIEEILER

IVQDIPSPKTMEDDLLRALVFDSFYDTYRGVMVYIRVVSGEIKKGSQIKMMETNRSYEVL

EVGVFSPKEKPTSSLRAGEVGYLIGNIKNTSDVKIGDTVTLQKFPAKEPLPGFKHIKPVV

FAGIYPIDSSDFQNLRDALVKLQLNDASLHIEQESSLALGFGFRCGFLGLLHLEIVFERL

QREFDLDIISTAPSVIYRCIMNDGTVKEIDNPAHYPDPTHIDTIEEPWIKCMIMAPSEYL

GAIMSLGLEKRGNCVKTENMNDKHLLLTYRFPLNEIITDFSDKLKSVTKGYGSFDYEPDS

YEKGEIIKLEIRVNDEPVDAFSCLVHRSKAESKGRAICEKLKDVIPMQLFKIPIQAAIGG

KIVARETIRALSKNVTAKCYGGDISRKRKLWEKQKKGKKKMKEIGKVNIPQSAFMAVLKA

ESD

>tr|D6YRM1|D6YRM1_WADCW Putative rhs family protein remnant OS=Waddlia chondrophila (strain ATCC VR-1470 / WSU 86-1044) OX=716544 GN=rhs31 PE=4 SV=1

MHIVDYDFLKRGVSNTAPPKLAGRVNLGKSWMIISNNCPLMKHGAEILFFGRKQGDSNYS

LTNVTFGNGCHHYYAYKAILRSMSIGYSLTTPRGL

>tr|D6YU82|D6YU82_WADCW Putative oxidoreductase (NADPH), flavoprotein OS=Waddlia chondrophila (strain ATCC VR-1470 / WSU 86-1044) OX=716544 GN=wcw_0319 PE=4 SV=1

MMNQNYSRLNPYLAKIIERVPLCSIDSGKEIYHIVIDIKGSGISYEVGDCLAILPSNHPE

RVQKVLSVLGLRETDRLIDTRANEDVDAIELFSKRLNINDCPLALVKKAAESSTDEDLLA

IAQDKQQVKELDLVLFLERCGIGSLTAQDVADLLRPMMPRFYSIASSMDVVGEEVHLTVA

LGKCVIDGIKRHGVCTDYLCHQAPVERPELPVYLHPHRGFTLPENPETPIIMVGPGTGIA

PFRGFMQQRERRASHHKHWLFFGDWTKKSHYYYSDYWENLSSKGMLKLDLAFSRDQEEKI

YVQHLMRQNGRELVDWLREGAYFYVCGNASKMAKDVDLALHQVLEDHGNLTAEEARAYVK

QMRADKRYLRDVY

>tr|D6YVT8|D6YVT8_WADCW Putative O-methyltransferase OS=Waddlia chondrophila (strain ATCC VR-1470 / WSU 86-1044) OX=716544 GN=mdmC PE=4 SV=1

MNPYHEKAKEIQHYLNALFAPEDSFLCEVKHQMEINHLPPISISPFIGKFLALLAGLVNA

KRILEIGTLGGYSAIWMGRTLPENGELISIEINPKNAEVARKNLKDAGLESKVDVKVGNA

LRIIEEMSDPFDLIFLDGDKPNYPHYLEPMIRLCRPGGLILADNLIRRGKAVNPSPTDEQ

ANAIATFNRLIASHPKLESVIIPTLVGYIGGDLDGLSISRVKS

>tr|D6YTW3|D6YTW3_WADCW Uncharacterized protein OS=Waddlia chondrophila (strain ATCC VR-1470 / WSU 86-1044) OX=716544 GN=wcw_0199 PE=4 SV=1

MFTPTFLRALSLACCFHLTGFVLFHVQPFRIAQTSTQFPPVQVNIEMTPPNSAIYANVEV

ESSRRRTLPEPVLPKPFIPRLSLNSPSRAEVLPSEKPFPMTPIIFEEPDALIDEWIAFDP

APSSKIKAPQIRLLGALSNRLPILNTLPVSTFPLKKSSLKRLTYSVKIDDRTGTIFWYSA

QSENGFTDEAEAILKSIRFEPLANGFVTEGQIELLGVSND

>tr|D6YSP6|D6YSP6_WADCW DnaK suppressor protein OS=Waddlia chondrophila (strain ATCC VR-1470 / WSU 86-1044) OX=716544 GN=dksA PE=4 SV=1

MALKKSEVAQFKKTLEELKNQLTRILQGSTAEIKKPDEATGYSQHQADQGTDDFDRTINL

EVTGNEYEILRQVERALEKIEDNTYGVCDITGEEIPKARLEAVPYATMTVKAQEQMEKGL

L

>tr|D6YTR3|D6YTR3_WADCW Uncharacterized protein OS=Waddlia chondrophila (strain ATCC VR-1470 / WSU 86-1044) OX=716544 GN=wcw_0149 PE=4 SV=1

MLNILCALHCEAKPLIQHLRLRKLQESAPFPVYENEAMRLAVCGIGKIAAATATGWLGAR

ENTCSAWLNIGVAGHADLPIGYQALALSVTDAGSSKRFYPDISLIKGLPMHGVTTVDKPS

FSYPDKDLVDMEAAGFYQAAQRFATVDRIHCLKIVSDNAWHPTEKINKEFVRDLIGQSIT

KIESLIGQILALIPNPPAIDDSPYVALCHWTETEKHQLERLLIRWKAMEKSPPLCQHLKT

SREIIQFLEHTLNQLPLCFTPSTTKKK

>tr|D6YSP9|D6YSP9_WADCW Uncharacterized protein OS=Waddlia chondrophila (strain ATCC VR-1470 / WSU 86-1044) OX=716544 GN=wcw_1753 PE=4 SV=1

MNPYQYLNKTYLTSTFFSICESISSISCSSYEEPLQKDDSQQLPSFSSSDILLNFFKNRP

LPSTSLPPFTSPSAVIDLFNSVEKENQPLLLVLRSLFKEINQFKLPQTSMIFQGPLLLHL

LNQAGYLHDLFNRQGLPLPLEQKDLLDDPELSLLFAECLITVQNTTQLIQLAHFLNVQFH

IESTISKNTLTAEFKKEDGTCVILKITTEKHPLQNAIALDLQDHFANQSDSMPLLVQDSN

PWDRLVLELTRCMQPKNWIDCILQYSYGKRAPDQQGINAIKQDYVNRLPCDNRGAQCAQD

LIQVCRASQISNGDAFFTIALQLLVSTDLNESEKNTCLKKLIFHLETLSPITDPTLVSLL

KSCRNKNPFSIIQSFMEIAAIQAALRHSDRYRTTLTWSGEELHLRLSFGKTGAFILLPFD

PKGALMNLTDRQLSSNCYTLFLELTESLGKPRIKDLQADFILSSSALAETLDSLARTENP

LLCHLALHWACFQGKELPFNRNHLAKILIAGFSSPMNRKASLQLLEALSSSQISLPSNLT

KTLEPLLKSTKYPSIKIIRNCVKTAFLNDKTVGFLTYDLWKEEVYEAETLSNSQFDEGKK

LLQSLLSSCQWRQTIEILGILSKKTPQWTNDVIEIYIQIFNLLPAVTSAAEKDDSMTRLA

RQLENTLKKHKEAVKTPSSLSVPLREMIKHCIGIGENKIACNFLMYAIEWNLFDDHFASM

TEKVLRILYKKGGIDHFILAAKILKSSMAYLSSHDLKTCGKLIASWISKMEFNSDSTPLF

IEAIEKEGLLSSFHSSVSQKQKSEFYETIYKALAESADPPKLLKAMIAWMAHSAEHSIDL

HSPKGYLEKLEQLLLQLKRCTCYNTLESTLFLLKKLPNEKKLTDSEQKKRAELHFQLLTR

AYLIAPDTLSPKAHLSYFISQTAVFEGYGELVQLLKKAQK

>tr|D6YU00|D6YU00_WADCW Uncharacterized protein OS=Waddlia chondrophila (strain ATCC VR-1470 / WSU 86-1044) OX=716544 GN=wcw_0236 PE=4 SV=1

MSVDKIILRSTAKFASTPTPLPQICRVTFLAFHKVHRLWRWVRLGNIYSNPNNFAQLAAG

HGINYIIGDSTLVRISAISVLISTRILQAVSEYEKLQDAWLKMKDAFQHHYPEPVRCSWD

QTHNFLSLSSIIWIKTTTKTTWCRVRLIAIAIFRVGKHFILLSLRMSDAVEAFTLKPEIR

EESINLMFVNTATCLSKLVDNKDFLLERLESNQKIIEKVLKGLGSQLTSDALIDTVEEAL

EKTSSIRRTTSSINDHIGEFISACGKKWTYEFLREVGLRHLVPSTLLPPSMPPWEEPKRR

ELKQYPPDSWLKRPNIKIQEKKKSFSSQNPLTGFVKKRENRTWV

>tr|D6YVZ0|D6YVZ0_WADCW Uncharacterized protein OS=Waddlia chondrophila (strain ATCC VR-1470 / WSU 86-1044) OX=716544 GN=wcw_0940 PE=4 SV=1

MKKIITAFSMALIAFYSNSLSASVEGTELIVEMIDLNNVNKSQVDPIFLELLEKYKRGVH

IAVENLPDQLSEFNVIIDRPLFKEGFLGYSYDKLIRKYSVTKEELLENQKQFSSSHPIVD

SFVCAGYLPGEEINLSLLTNENKLIANTSFIPNPLEKTFSDGKAKISLEFSGGQASRYKV

FLSGFKDNEPITFQSISGTEEIPPQTFRIKKNIGFFYSNGVVGAYGGKSILSFQRESGEK

AVFILPWGNQLLPYLEGKVAFGEIP

>tr|D6YUN3|D6YUN3_WADCW Methyltrans_SAM domain-containing protein OS=Waddlia chondrophila (strain ATCC VR-1470 / WSU 86-1044) OX=716544 GN=wcw_0473 PE=4 SV=1

MNQNTLFLPLITDNEDKSFPLKNRIRKNYRHLRKWANRTKTNCFRIYDRDIKEYPLAIDF

YDGRFCVQFFSFDHDNDNAPEELVKEINDVLNSIFGTAPGLIYWRSRIKRRKFEQYEKQA

EEKDFFTVLEYGAKFKVNLQDYLDTGLFLDHRETRQMAASLSKGKRLLNLFSYTSSFSVH

AALAGAAFTKSVDLSNTYTEWSKNNFILNDIAAKNHPIVRADCLKFLEEEVRSNNRYDVI

VIDPPTISRSKKMGKMFDVQKDYTFLMTMGLKLLSKEGVILFSTNSRKFLFDSSLFPHCF

IQEISHKTIPLDFHQKKIHRCWKLSTSCI

>tr|D6YWN8|D6YWN8_WADCW Putative Skp-like protein OS=Waddlia chondrophila (strain ATCC VR-1470 / WSU 86-1044) OX=716544 GN=ompH PE=3 SV=1

MKTLTKFVITGLTALLFCASTPKLEAAAANIGTVAFRTCVEQSKYGAREQKNFEDLKKQM

ESVLEEKEKALTEISNKFNDPDYLDSLSNEAEAELKHKFRTLSGELQQHQQQYMQILQQT

NFKIIQGITEKVNEAAGIVAKQKGLDLIVNDESTFFHAASLDISQDIIKEMDKMFEKEAK

KS

>tr|D6YS16|D6YS16_WADCW Uncharacterized protein OS=Waddlia chondrophila (strain ATCC VR-1470 / WSU 86-1044) OX=716544 GN=wcw_1512 PE=4 SV=1

MYFIHLIKELEMEFNLGIKDPSTEAICLKINEEYHLSFYSKDNGQSVLIESDIYELESSL

GKDFYQHMLSANFEGVLPWETYFGINPETNHALLMKKVLLEHIDYDEFLIQLNQFITLYE

ELQQQLYHWKIESFFHNKPTFEQKSENQNKLFI

>tr|D6YU94|D6YU94_WADCW Uncharacterized protein OS=Waddlia chondrophila (strain ATCC VR-1470 / WSU 86-1044) OX=716544 GN=wcw_0331 PE=4 SV=1

MDIGRRALYNLIRMNWINDPTTPYERWQVEDYRSLSDSQLFHLLQGLGIPWDSEAFLLYA

QSVESPEELTDLLLKDADLEPAEQDRIYLVLFEIWRRLLPDRMSLSLFSDELDHQIFMYD

KGDVSTAESIQDAIANLQDLMDDHVDEGESHQQIFQLISEACANDLESFLYDYIAEQIDN

DDSGYAAELVEGLDPYIGKSMWFELLKVRLLELEDPEASQEELRKLLAKAMREKELTFNL

EVLAFVSQCGEKKDFNKMVRASIPLLNIEEDFQELLSICEDYYRCLDEDGKEQAVQEILN

PRSYYDLSENLDPDDPGLKELLDLIK

>tr|D6YVK2|D6YVK2_WADCW Uncharacterized protein OS=Waddlia chondrophila (strain ATCC VR-1470 / WSU 86-1044) OX=716544 GN=wcw_0796 PE=4 SV=1

MEEDKQTKVSGRYFFHQKEAYSNPDADNVALQERLLDVCAGITGISFS

>tr|D6YTZ0|D6YTZ0_WADCW Uncharacterized protein OS=Waddlia chondrophila (strain ATCC VR-1470 / WSU 86-1044) OX=716544 GN=wcw_0226 PE=4 SV=1

MCPVSAANHLELIAKIYFFDKKLHFLYQEGSTKRSNKVAL

>tr|D6YUU9|D6YUU9_WADCW SnoaL-like domain-containing protein OS=Waddlia chondrophila (strain ATCC VR-1470 / WSU 86-1044) OX=716544 GN=wcw_0539 PE=4 SV=1

MGKKRVKEALEAILTDLGASVSLIEEYFHPDYVQKVDGKTLDFEGFVAHMHSLRSKVDRL

KLTFEKLFEEGENVCSVHIVDVAKKDGSNARMKVIAHFKLSGGKIIYCDELTFLIAGEAQ

DAQLGSCFS

>tr|D6YWH2|D6YWH2_WADCW ATP synthase subunit alpha OS=Waddlia chondrophila (strain ATCC VR-1470 / WSU 86-1044) OX=716544 GN=atpA PE=3 SV=1

MQLNPKEISWVLTQEIESFEDHVKLESVGRVIQVGDGIAQIWGLNDVMVSELVEFPDGTQ

GIALNLETDTVGVIILGSGQEIKEHDIVKRKHAVVSVPVGDELLGRVITPLGQPLDGKGP

INASQMRPVECTAPGVTQRMPVCEPLQTGIKAIDAMIPIGKGQRELIIGDRQTGKTTLIL

DTILNQKNQGVHCIYVAIGQKLSTIANFVSILEHHGAMEYTTIVATSAADSASLQYLAPY

AATAMGEHDMYQGKHAICFYDDLSKHAQAYRQLALLLRRPPGREAYPGDIFYLHSRLLER

AAKLNKELGGGSLTSIPVIETQASDVATYIPTNVISITDGQIFLESDLFFAGVRPAINVS

LSASRVGGKAQTKAMKKVAQSLRLDLALYRELAAFVQLGADLDETTKAQLIRGERMVEIL

KQNKLKPLPLQNQVIEIFIGTKGFLDDIELHLVDTFIKELMSEIEKEHPALLQSIAESQD

LEPKVAETIEHTAKAFKEQFIARQRKG

>tr|D6YTC6|D6YTC6_WADCW Mutator family transposase OS=Waddlia chondrophila (strain ATCC VR-1470 / WSU 86-1044) OX=716544 GN=wcw_0010 PE=3 SV=1

MSNFNYENALAKKAIHEIYMAPTKEDGLAAFEVFLKTYRDKYPKACACLEKDKAQLFTFY

NFPAIHWQHVRTTNPIESTFATIRHRTRQTKGCGSVPATLTMVFKLATTAEKKWRKLKGC

EMIEKVINGVVFKDGEEVLEKEKVA

>tr|D6YTN0|D6YTN0_WADCW Putative thiol:disulfide interchange protein OS=Waddlia chondrophila (strain ATCC VR-1470 / WSU 86-1044) OX=716544 GN=dsbD PE=4 SV=1

MVTQIKKYIPVFLTLFAIACFAQESFDHFQDPVQAELIYESRSIRPDTPFWVGVRLKLDD

GWHAYWKNPGDAGMPPQIEWTLPEGFSISDIQWPSPKKFTAMEAIGFGYEDEVILLAKVT

PAKTDHSEIQIEASISWVVCDSSSCLPGSANVKSTLAVSTDKIQVHDVHAGHFEKARSKL

PKAMNSLKGERKNGLICIPIETSEKIVSANFYPEQEERIDISVDPVIADDATILLKEQGS

SSKDPLKGVLVLNGKDAYEIYVSLDPHHSTDVAMNLNNASYPAASGDAHKFSGSLWLAIA

LAFLGGLILNCMPCVLPVISFKILSFVKMAGESRRLIFQHGLAFSFGVLLSFWVLAGVLL

MLQSWGQSVGWGFQLQEPLFVGILAAIILIFGLSLFGVFEIGTGMASAAGQATTKSANGL

TGSFLSGILATAVATPCTGPFLGTAVGFAVTLPIFQALTIFTSLGLGMASPYLLLGAFPN

LLRWMPKPGNWMITFKEIMGFIMLATVLWLIWVFGAQTDTMALFILLIGFFFLAIGCWIW

GKWGTPVMKKRTRMIGLAAASATFIIGGYAITKASSMSGNSPAPLTNAEIAMAEASPSEL

TDRWIPFSPELLEKLQDQGIPVFIDFTAKWCLICQANHLVLEVDNVEKKFLQKGVVRMIG

DWTKSDPVITEWLKKFGRNGVPLYVLYNEKREVEVLPQVLTPDLVIEKLDALNL

>tr|D6YRU3|D6YRU3_WADCW Putative deoxyguanosinetriphosphate triphosphohydrolase OS=Waddlia chondrophila (strain ATCC VR-1470 / WSU 86-1044) OX=716544 GN=wcw_1439 PE=4 SV=1

MKELPSFYPLNVAENVSPLLEKCRKKAQENEKANMHPMAAFSDSHKRYKLSEVDHRLPYK

RDVDRIVHSKAYARYIDKTQVAYLIENDHITHRGLHVQLVGNFARGIAEILRLNLDLVEA

VSLGHDVGHPPFGHEGEGYLSELTQEYGGYAFAHPYQSCRLFSKIEPLNLGLTVYDGILC

HDGGMRKTHLEPRYGKTWDDHFKDLETKLKYPEETIIPATLEGCLVKLCDTMSYLGKDIE

DAINLGIIERRDVPETLLGKSNREILKALACDIISNSFEKNYIAISDEAFEALKILRVFN

FERIYQDPSLKKESAKVKRSYRILFEYLLQDFERQQENSYLWKNFLENKPEAYLKESSPI

QKTADFIAGMTDSYFIRTLEKVFVPTRIEWRSC

>tr|D6YVV7|D6YVV7_WADCW Uncharacterized protein OS=Waddlia chondrophila (strain ATCC VR-1470 / WSU 86-1044) OX=716544 GN=wcw_0906 PE=4 SV=1

MCSFYSILDAAPKKYTLSTVCMFNNEAQYLKEWIEYHRLVGVDHFYLYNNNSNDHYKEVL

KPYIQSGIVDLINWPSPPETLYVFYQKDAYNHCVKHYGKNSLWMAFIDTDEFIVPVNHSS

IPEFLKDYRRYGGVYISWQCYGTSHLSHIPEGKFMIESLTLKFPWNHKKNLFFKTIAQPE

KIKECFIHDCSFKKGHVAVSPSFVKGHPRSPDIDKIRINHYWTRAEDFFFNVKVPRVESY

SKKPMTQEEIDKILIESNSIEDLSIFPFVNDLREVMGDE

>tr|D6YUK1|D6YUK1_WADCW Uncharacterized protein OS=Waddlia chondrophila (strain ATCC VR-1470 / WSU 86-1044) OX=716544 GN=wcw_0440 PE=4 SV=1

MRKSLSILFLAIFVELFSFPAAKIERVGNSEFESFTTFKAHSCIAQHEWQEERSQPKRQG

NSPTGKDAQDLPFILHHEKFLTELLVISLHFHPVSPSYDEAKLEVLKSRSHPPTKLLF

>tr|D6YV95|D6YV95_WADCW Uncharacterized protein OS=Waddlia chondrophila (strain ATCC VR-1470 / WSU 86-1044) OX=716544 GN=wcw_0689 PE=4 SV=1

MSNHPFYGKEIYRDREEKYIKTLLKKYRKEPASEELKETIWNELMWEKHLGNITIPFKVV

IRKDSSNQYPEIVEVILDTKV

>tr|D6YSY6|D6YSY6_WADCW 7-dehydrocholesterol reductase OS=Waddlia chondrophila (strain ATCC VR-1470 / WSU 86-1044) OX=716544 GN=dhcr7 PE=3 SV=1

MAATTTNVQTRNWGRAWETTWLSLFSTIALLATAPMMVLYCYIACVRFRGSLIGPAYALA

SGAVSLDSLFPSFEVGIFALYLGWFAFQLLLYLGLPDLLHRILPRYRGGRQEGAVTPAGK

QLVYQINGLQAWLISHLFFGIGAYVLGWFSPSIIAENWGGFLIVTNVMGYLTAIFVYVKA

YRFPSNAEDRKFSGNPLYDFFMGIEFNPRIGKFDFKLFFNGRPGIIAWTLINWSFAAKQY

ADLGYLPNSMLLVNVLQAIYVLDFFWHETWYLKTIDICHDHFGWMLSWGDLVWLPYMYTL

QGLYLLYHPVDLSTGFALFVLTLGVVGYAIFRSANHQKDHFRRVQGKEPIWGKMPEFISC

QYTAADGSLHHTKLLLSGWWGRARHMNYTGDLMLSLAYCLACGFSHLLPYFYFVYMTILL

VNRCYRDEHRCENKYGDAWRKYCRRVPYRLIPGIY

>tr|D6YTM6|D6YTM6_WADCW Putative iron-containing alcohol dehydrogenase OS=Waddlia chondrophila (strain ATCC VR-1470 / WSU 86-1044) OX=716544 GN=wcw_0112 PE=4 SV=1

MSDHYVFEFPTLINFGQGVSNLLGNYLDQQAIRRPLFVTDANLSKQPFFPNLIEQSGGSI

IYDHIPSIPTKQSVVNGKEVYNKHGCDAVIGVGGGTAIDAARAIALSIHHPGDLFDYSIE

KRGAEKISKKIPPFITIPTACGTGSEVSRGCVITDKATSEKAAIYSPRLIAQAVFADPEL

SLELPPSITASTGIIALSNNIEAFLAKNFHPMCDGVALEGVRLVFEHLPKAVKKGDMESR

AGMMMAALMGSVAQQKGQGVIHAAAHSLSSVFNTPYGIANAIMLPHGLEFNIPQSPRKLQ

QLSDTIWSHDFIRSTKELAETLELPRSLKEIGVSEGDVAHLSALAYQTPYHSYNPRPVQE

KDFADLYLKALKS

>tr|D6YUB7|D6YUB7_WADCW Putative N-acetylmuramoyl alanine amidase OS=Waddlia chondrophila (strain ATCC VR-1470 / WSU 86-1044) OX=716544 GN=amiA PE=4 SV=1

MIKRWIKNVWVGLIAVVFAGCASKSPEWERSDHEVAAVRVRREAVRPVIVIDAGHGGKDL

GAKCPDPQTEEKALNLQTALLLNQFLQQKGYQTILTRGEDFFVPLKMRADFANSNRATLF

VSVHYNSAPNKAAEGVEVYYYDNQDDLVRTSRSKILAQKVLDRVIASTKMKSRGIKNGNF

AVIRETRMPAILIEGGFMTNEKELARLRDPAHIQRIAESIANGVQDYLRS

>tr|D6YW17|D6YW17_WADCW S-adenosylmethionine:tRNA ribosyltransferase-isomerase OS=Waddlia chondrophila (strain ATCC VR-1470 / WSU 86-1044) OX=716544 GN=queA PE=3 SV=1

MLDKLESYQFDLPEDLIAKVPVTPRDHSRLMVIDRSSGKIEEIPFYELPQFLGDGDRIVF

NNTKVIPARLIGRKETGGQVELLLLEEKTEGVWLTMARPARKLKKGTKVIVSDRLCAEMI

EELDEGMRLVRFDYEGLFWEVLAAHGEMPLPPYLQRDELPKLDRERYQTIYAKHAGAVAA

PTAGLHFTKTLLEELTEKGVDIVEITLHVGLGTFRPVTAKTITEHKMHEERYWITQEAAS

RLNRPAKREICVGTTCCRALESAVNEQGNVFSGEGRTDIFIRPGYQFKKVTSLLTNFHLP

GSTLMMLVSAFAGYDLTMNAYKKAVLDKFRFFSYGDAMLIL

>tr|D6YWF9|D6YWF9_WADCW Mutator family transposase OS=Waddlia chondrophila (strain ATCC VR-1470 / WSU 86-1044) OX=716544 GN=wcw_1113 PE=3 SV=1

MKNDVIFLDKFHATSEMNSLLEQTLREGARLLLQQAIENEVNEYLESMKGRRDFEGRKQF

VRNGYLPEREVQTGIGPISVKQPRIRNREECSEGYSSAILPKYLRRVPSLDAVIPALYLR

GISTSNFQDALETIMGKDAKGLSAANITRLKQSWEQEYKDWNKRSLEGKRYAYIWVDGIY

FNVRLGDDRICFLVILGALPNGKKELVAIHNGYRESKISWTEVLESLKRRGLCTAPELAI

GDGALGFWSAIEEVFPKTKQQRCWVHKTANVLDKMPKSIQVNAKKAIHEIYMAPTKEDGL

AAFEVFLKTYRDKYPKACACLEKDKAQLFTFYNFPAIHWQHVRTTNPIESTFATIRHRTR

QTKGCGSVAATLTMVFKLATTAEKKWRKLKGCEMIEKVINGVVFKDGEEVLEKEKVA

>tr|D6YT58|D6YT58_WADCW Putative membrane protein OS=Waddlia chondrophila (strain ATCC VR-1470 / WSU 86-1044) OX=716544 GN=wcw_1916 PE=4 SV=1

MMSDKKEHEEEIIVEKETKEESGKKFSETIENLKKNKNIESILEYARNNTKDTVAFVLLI

IGILWMLGQPFNGGILVGLVVGFYFSKEFISYVKDFNSHLEEQGLAKTVILGGGALALFF

LAPGIFIGVAVMAALKYLLKSE

>tr|D6YSN9|D6YSN9_WADCW Uncharacterized protein OS=Waddlia chondrophila (strain ATCC VR-1470 / WSU 86-1044) OX=716544 GN=wcw_1743 PE=4 SV=1

MGSTVSTLLDSHHILHPREENHEIGARNEPTIFRRILGVVLVALSIPFTLGIATGLYIYW

AHRKVKYIEERDYSELQARTNKVFGEMIQSEAPSSEQVQFEAALKAIQQGGKIQQLPEEY

DKRAIALLDAALAYDNTSAVTALMLKYEDSVNDIHLANVKSVKMLHAILERVPEGEEREN

LLNKKDGEGNTALHLQKNLSIIEELVKKGAKFSGNSLELSPLHTCQDPKIAKYFIDRFKG

EMDILNLSNDDVPAPIFTVPPAVVRVLIEEGANVKARDLKGNTILFRFTEKPFSSDPQLL

EWILSKIENADEKKDFICLVCQEHSILNKLIQSDQANVDIVKVLLNAGARYPKLQKGDSP

LQYVKVPEVAACLIDHYGEDIMERKNKDGVSPLIQARSNLEVFKIMIDKGVKVTEARDKD

ESTILAYFSVEPLKNHPELIHTILEKIEDTDEKKAFVNHKDTKGLAALNYGLHPLQVVQA

LIEAGSDASPGVRALKTCKDPELARYLMEKFGNQFNEKLTKGVLSPLFVSKKDGSIALSE

LCSLENMNLSN

>tr|D6YVY1|D6YVY1_WADCW Uncharacterized protein OS=Waddlia chondrophila (strain ATCC VR-1470 / WSU 86-1044) OX=716544 GN=wcw_0931 PE=4 SV=1

MPMAGRRVKAAVSPIEKFLAFSKRREICYDGR

>tr|D6YX07|D6YX07_WADCW Uncharacterized protein OS=Waddlia chondrophila (strain ATCC VR-1470 / WSU 86-1044) OX=716544 GN=wcw_p0002 PE=4 SV=1

MNRYKLYETDEYLEWLATQTLKSKKQIQSRMLKIEDEGYFGHHKYLESADLWELKFNDGR

RIYYVLVPESKVILLLGGNKNGQNKDIKQASNILRKLVKS

>tr|D6YRY4|D6YRY4_WADCW 50S ribosomal protein L17 OS=Waddlia chondrophila (strain ATCC VR-1470 / WSU 86-1044) OX=716544 GN=rplQ PE=3 SV=1

MRHLKDRRKLNRTSSHRRCLMANMLKSLIVNERIETTVPKAKCLRRYADRMITLAKKNSL

ASRRRAISQMMIRFNSLTPKQARAAKNGDTSSYNDDRLVIEKLFSELGPRFTSRQGGYTR

VLKSDRRVGDNAQKCIIEFLES

>tr|D6YTE5|D6YTE5_WADCW Uncharacterized protein OS=Waddlia chondrophila (strain ATCC VR-1470 / WSU 86-1044) OX=716544 GN=wcw_0029 PE=4 SV=1

MLIQATATLSFGAQMSKWKGWIDGTRKKPDWGVFNRTYEYRLSTEREGL

>tr|D6YV69|D6YV69_WADCW Putative transposase OS=Waddlia chondrophila (strain ATCC VR-1470 / WSU 86-1044) OX=716544 GN=wcw_0662 PE=3 SV=1

MYYEVLPWKQHQAVGKNSGKTSYIERFNNTLRQRCSRLVRKTLSFSKKLANHIGMIQYFI

CDYNKRMALHV

>tr|D6YWA3|D6YWA3_WADCW Uncharacterized protein OS=Waddlia chondrophila (strain ATCC VR-1470 / WSU 86-1044) OX=716544 GN=wcw_1055 PE=4 SV=1

MLQVPLIASSHDYLMTVSRKDLCPLDYQVKVNLLERVWKAFLKILRNFLRYFSLSREAMP

FDLSKRQIGLIRKAREVRVGSSPLASRAVVPRNVAGPIEEIAETRIAEHDPTLEKLKEFL

QKFSGIAVNDCAYQETISPRFPEWKEALTTLVKEIPDWNETLIKLKESLTPLMNGVKKAD

DPAITLIIQQLLKHYIQLDKKTVKTELLENLENQKLAGNIDEIEFERTVAYLDPVLSWLY

HPNNWVVQSAQPVNLHNFISDSFRFLDTLSAKIRNGDLNVFFKNMISFIENDFENSLKQA

FEHNTSLIARLFSNRLADLIEHLPYSETYADILRKIVEHMEGWISANEMKKEQERLVDDA

IKANNAHATSVSEIDRQRTAQEFLDFVREDGGRDNYLHKEFLHKFSQHEACHDEIKKIID

SDSPEEAEKIKKKAYENMVELLFPIFLPDEKQALPHGLETKVNGISSVLGRLIFPEKIEM

LKNEAMRVFEEILTASEVKDPENFRVYFYSLIHMIAVQYVQTKTKSVLASELQKLMTRLS

TKEYLDYLAVEYIFPSLIRKTLEGFVRAHIERKSNEIAIAFYEMFNQDQKIDLLLESLYQ

SVCKNLIDFNMDEAEIGFSEFESIVKPILLEIHDFIENKRRESGDEMLSLNQVKNDLNEY

LKWETVPVNHDYGKLIMHALFKIGSFGGWFSEKLIGWFQGTLSETTSQTMHPISNHYKLL

VDSIVETASQNFLSVEFVKEYLFSEELSVEEKRRQKEKVERDLPKQIRRISALAHDTIYR

SLEARSIPFIKYSTPKTENIETIIQNVFTRIFGDEKLNESLFLSSVEIIGKSFQSSVQRI

E

>tr|D6YWT6|D6YWT6_WADCW Citrate synthase (unknown stereospecificity) OS=Waddlia chondrophila (strain ATCC VR-1470 / WSU 86-1044) OX=716544 GN=gltA PE=3 SV=1

MSGVTSEVLFEITKDQLETGMRGYPVGYCTTSSVDPEKGLFYVGRPVSELSKNTPEEVIY

LLYHGKEGSQQELSAFKEELCKRSIISDQLIESIRALPRQAHPMQLLVCALSLTTSFEGA

GDWKEDGLNLVAKIPVIVAHLINHHAGWGECNPSKPEMGYMENFTQMLNVPNADLKQLTE

VMRLFNVLHYDHGGGNLSAFIGKGIASGLQDMYGSLAGAMSALAGPRHGKANQDCLEFVK

DVLGNLGENATAGQVEQLIRDRLANNQLIYGFGHAVLRVEDPRASVFYEVAKKLFPDHPL

VKIAKLLRTEGTKVLKENPKISNPYPNVDAISGTVLTAAGFPYPEYYTVLFGLSRVVGIS

RQLIYERVEARGGKGTPIIRPKFIYAGQ

>tr|D6YV44|D6YV44_WADCW Putative dihydroneopterin aldolase OS=Waddlia chondrophila (strain ATCC VR-1470 / WSU 86-1044) OX=716544 GN=folB PE=4 SV=1

MQGTIGFNHLRINCIIGDLPEEREKVQEIEVSVKVACDFYACSLSDDLADTVDYVSLAAA

CRREAEEGRYHMLETYASRTLDKLLEEFPIDYAWIQVKKASGLPDADCSFVELSKKKKYP

G

>tr|D6YS78|D6YS78_WADCW Transposase OS=Waddlia chondrophila (strain ATCC VR-1470 / WSU 86-1044) OX=716544 GN=wcw_1575 PE=4 SV=1

MPMELATTNIDHLGLVAGMCDEIGLVEQVDKACGDQDPNKNLTFGQCVKCMIMNGLGFIG

RTLYLYSEYFEDKPLEHLIGADVSPEQVDNNVLGRTLDKLFSLGVTDLFSAISLQAIKTL

GIKISSLHLDSTSFHVDGDYNSDLEQDDHRIKITKGYSRDHIPFMNENFTCC

>tr|D6YVY8|D6YVY8_WADCW Putative membrane protein OS=Waddlia chondrophila (strain ATCC VR-1470 / WSU 86-1044) OX=716544 GN=ompA5 PE=4 SV=1

MKRHIAKFLIFIFLSSINVEADCCKNITYTKPPPPIRPCCCSDDCYSEPPRTYDPYCYAC

DQFCPGECHFFFGAEGLWWTACENDLDFAVDFNTEQTEILGIGNTHFASYDWDWGVRGWL

GWNWCCGWDTVVSYTWFKTEGNQFIDTTDEDISLKASLLHPNTGLSDADTATAKLDLKYQ

ALDVLFGRTIVYCEDTVQLHPFFGFHGIWLKHDQSYLYEGGDFGTGSLAAPARVTWNSTL

KGAGLIGGADVNLKWPCGIGIFGSMGGSILASKTDIEHLQEILDISGNITSTDIDLKEDQ

CVCIPGLHLKTGLSFQFACGQCLLMKFHLMYEFNNYFNTPHLRRYSYNNEGVSSSSASGN

TALQGITVGGEIFF

>tr|D6YTE4|D6YTE4_WADCW Peptidyl-prolyl cis-trans isomerase OS=Waddlia chondrophila (strain ATCC VR-1470 / WSU 86-1044) OX=716544 GN=mip1 PE=3 SV=1

MTSTKRYLFSLLVMVITFFSYCFSESSQKTTPSDLIAYEIKKNLPDQKYLDAIIEGIEAS

SHNVAVSEEEYEAFFSAGLQERQWKSQQNLIASEKWMLEKSNEKNISIITPSELFYQVVK

EGDGQTLLSSSVRVRINYLIFKFNNLFPESCGKKQVFELSHLVPGLSRGMTEMKEGEIRK

IYIHPKYAYADSNTFDPNCAIEIIVELLNILPGSGDIASLAAVPTTSHSVLSDNHLKESL

TKKYYVIGWRLWNHLKYGYHLFNKEELMDSLRKEQLLENFSEIDREVNRIHWLIYQQRIQ

DQGL

>tr|D6YSK1|D6YSK1_WADCW Pyridoxal 5'-phosphate synthase subunit PdxS OS=Waddlia chondrophila (strain ATCC VR-1470 / WSU 86-1044) OX=716544 GN=pdxS PE=3 SV=1

MAEEKIQNSFEVKVGLAEMLKGGVIMDVTNAEQAKIAEEAGAVAVMALEKIPSDIRQEGG

VARMTNPLFIKQIREAVSIPVMAKCRIGHFVEAQILEALEVDFIDESEVLTPADENYHID

KHGFKVPFVCGACNLGEALRRIGEGAAMIRTKGEPGTGNVVEAVRHMRTINQQMRALAAK

DTAELMTEAKEMGAPYHLVKMVAETGKLPVPNFSAGGIATPADAALMMQLGAETVFVGSG

IFKSEDPKNRAQAIVAAATYYDDPKMLLEVSSGLLSGMKGVDIRKLRHDELMANRGW

>tr|D6YUS4|D6YUS4_WADCW NADP-dependent alcohol dehydrogenase C OS=Waddlia chondrophila (strain ATCC VR-1470 / WSU 86-1044) OX=716544 GN=adhC PE=3 SV=1

MLKDKKKEAPVSKTVKAYAAPTAEKPLSQTTINRRSPTSKDVVIDIHYCGVCHSDLHTAR

NEWQNTTYPCVPGHEIVGIVVETGSGATKYQKGDKVAVGCLVDSCRTCSSCKENLEQHCE

NGATMTYNSPDKHLKDTTYGGYSEMIVVDEDFVLKMPDNLDFAAAAPLLCAGITTYSPLR

QYNVGPGQKVGVVGLGGLGHMGVKLAAAMGAEVTVLTHSDNKREDALKLGAKDVIYTKDQ

NQLEKAVNRFDFILDTVSAEHDLNLYLNLLKCSGAMVLVGAPPEPVPVAAFSLIFQRRKL

AGSLIGGLKETQEMLDFCGKHNIVCDVEMIHIDQINEAYERMLKSDVKYRFVIDMKSLKG

>tr|D6YUP1|D6YUP1_WADCW Uncharacterized protein OS=Waddlia chondrophila (strain ATCC VR-1470 / WSU 86-1044) OX=716544 GN=wcw_0481 PE=4 SV=1

MYKKQHCEICKKPFFLRDLYPAVLIRDNTFEAAKKKCPEMDRKGYICFPDLRKIHASYYE

EILTQERGELSKLEKEVLSSLEQHDILSENINEEFDATRTLGGRIADKVASFGGSWTFIF

IFGFVLLSWMAINSYQFFFEKALDPYPYILLNFFLSCLTTIQAPIIMMSQNRQTAKDRLT

QENDYQINLKSELQIRQLNARLDMFMRHSWQKLNEISQIQEEILEILENLGR

>tr|D6YWL4|D6YWL4_WADCW UDP-glucose 6-dehydrogenase OS=Waddlia chondrophila (strain ATCC VR-1470 / WSU 86-1044) OX=716544 GN=ugd PE=3 SV=1

MDILVVGIGYVGLVTATCLSEMGHHVTCLDIDKEKIQRLNDGVIPIYEPGLEEMVKRNVA

AKRICFTTDYEKGVNGSKVCFICVDTPIDSNGHASLNAVKSCAQSIAKRMSGYKIIVNKS

TVPPGTARQIHQWVQEILDQRKMIESFDIVANPEFLKEGCAVTDFMKPDRVVIGIDNPRA

TQIMKDIYSPFMLSRQRLILMDTVSAEMTKYASNAMLATRISFMNELSGLCEKTGADINK

VRIGMGADERIGYSYLYAGVGYGGSCLPKDISALKSHGNSLEYPMLLLEAVHEINNRQKL

VLSNKIKEYFEGKGGISGKTIAVLGLAFKPDTDDMREATSLILIKQLLSIGAIVKTFDPV

AMTNAKSLLPDTPNLIWCNDEYTCAENTDAVVFVTEWKQFRFLDFKKIQLSMRGCGLFDG

RNQYHPEEMVHLGFDYHGIGVPSKTGDFS

>tr|D6YVL6|D6YVL6_WADCW Putative carbohydrate kinase, PfkB family OS=Waddlia chondrophila (strain ATCC VR-1470 / WSU 86-1044) OX=716544 GN=wcw_0810 PE=4 SV=1

MTKIREVLGVGTPILDHLISVDPSFLKTIQGKPYGMELVSYDEMISIIENSGSIPLQIAG

GSCCNTIKGLASLGHACALTGKIGLDLTGEKVINELKKSRVEPLVCYNGSPTAHVACLIT

PDGKRTCRSFMGAGGEMTPEDLDPKYFQGVKLVHIEGYSLLVPGLTKQAMKYAKEAGALI

SFDMGSFEIVKEFQSSLYELLSEYVSILFSNEEETRSFTGKDPKEGCKVLSSLCQTAAVM

VGKNGCWIGSGEELFHSSAFPTVPLDTTGAGDLFASGFLHGMLKGLPLKECARYGAITGA

AVVQLIGAEIPSSSWPEIVERMTSS

>tr|D6YWM4|D6YWM4_WADCW Uncharacterized protein OS=Waddlia chondrophila (strain ATCC VR-1470 / WSU 86-1044) OX=716544 GN=wcw_1178 PE=4 SV=1

MLVNSLKKNKINLSDYNYEKDIECRKLMSDLSVFEVNTLTEIVHGSLNTTISQIAESVDA

TEEDVLPALEKLKKLKLFQINGDRINISKEMRKYYESQIPKFDNDFRADMEFVKGFLSKV

PIHVLPIWYSIPRTSDDIFGSIIEKFLITPKIYQRYLDEISFDDPILNAIMNDVYSHTDY

KIRVSEVIEKYSLTREQFEEHMLLLEFNLICCLSYNESDDHWVEVITPFYEWRQLLRERR

KNQPQPINHLLEIERTHPHDFGFVMDTSAITSTALNSPIPIHEENGEWTLPQETARHILG

RSITPQYARLLLKILIELKLVEIADHQIVSKDLSEDWLKKHLQEQAITIYRYGVHQGAPV

PGTYINRDLREIETCLKRLMNKGWIYFDDFLKGCCAPIGNNNPVTLVKKGKRWKYELPAY

SEEDIKTIHQYIFQTLFAAGFVATGTHAGKECFTITPFGRMSMD

>tr|D6YVF8|D6YVF8_WADCW DNA repair protein RecN OS=Waddlia chondrophila (strain ATCC VR-1470 / WSU 86-1044) OX=716544 GN=recN PE=3 SV=1

MLKQLYLKNIVLIEETVIPFKKGFNVISGETGAGKSALMAALQQIHGFRADLQLIRHGFD

KGIVEAVFDIDELPKVQQMLNESGIDHETGDELLIRREISRTGKSRLFINSQLTHLSTLQ

AIGGNLFKIVSQRGSQQLFDLDHHLELLDSYGNLKGLRAEYQAAFDKEMDTRKKLERLKN

SEQKRIREQEVCLRELEELTAADLQEEEEEELFAEYTKLTNTETLLQLSELITSTLDDQI

LPALRSVKQSAERLLSLDSLLAETHEQLASSVLEIEELTRSYHDYSNSIEHNPYKTETIN

QRLTLINKMKKKYGPTIEEAIRYKHSREELLASLENAETEIEELTHALQEQEKGSQELAE

KLTKERNRASLLFSEAMTKELRDLNMPHSIFSVKVEMMNRGKTGQDKVEFYFAPNKGEKT

VPIRECASGGEISRIQLSIQTLLAGKESIPSLIFDEIDANIGGQTAAIVGRKLQQIGKNH

QLLCITHFPQVADSAVHHIQISKKEKDERTVSVINVLRQEGKESELLRMAGKAI

>tr|D6YRL0|D6YRL0_WADCW Uncharacterized protein OS=Waddlia chondrophila (strain ATCC VR-1470 / WSU 86-1044) OX=716544 GN=wcw_1354 PE=4 SV=1

MRQYLQACSIKDVFFPLIIPYLSTIQIMRFIVDFKKNNYVIIGEEEIEMALDSGFSASSG

GGAASVREIFLKYVHNPALAQAQGPGDSAFDVVAPAVGAWSRMEGLVD

>tr|D6YSP7|D6YSP7_WADCW Transcriptional repressor NrdR OS=Waddlia chondrophila (strain ATCC VR-1470 / WSU 86-1044) OX=716544 GN=nrdR PE=3 SV=1

MKCPYCGQKELKVIDSRESLDMNAIRRRRECLDCLRRFTTFETIELSVQVLKRNGMYEDF

QQQKLINGLDAASRHTTISHDQVIALASQVTEELMAKQVREVSTTELGEIVMKKLQALDP

IAYIRFACEYRRFKDIDELIEEIQSIELYRS

>tr|D6YUA3|D6YUA3_WADCW Aspartate carbamoyltransferase regulatory chain OS=Waddlia chondrophila (strain ATCC VR-1470 / WSU 86-1044) OX=716544 GN=pyrI PE=3 SV=1

MPTLEKTLPVSAIQNGTVIDHIPAGKALKIVKLLNLSTERLQTTLGLNLPSGILGLKDLI

KIEGFVLSSESASKTAILAPLATVNIINNYEVQEKFQVSLPKKIQSVISCPNTLCISSQE

KVDSIILVKERWKGNIQLQCHYCRKQFSHQDI

>tr|D6YVM0|D6YVM0_WADCW 50S ribosomal protein L25 OS=Waddlia chondrophila (strain ATCC VR-1470 / WSU 86-1044) OX=716544 GN=rplY PE=3 SV=1

MKLKFEQRTAEKKSDSRSLRNLGKIPAVLYVRGKDSEAIAVDAADFETVLRTVKKGRLST

TKLALVDGTGKERNVLVKDIQYHVTTYNVLHLDFEELLDNVKIKVKVPIECVGEVDCVGI

KLGGVLRRVIRYLRVHCLPKDLPEFFKMDVKTLGLKESRKLSDLEIPETVRPLMDLNEVA

VTIAKR

>tr|D6YWY4|D6YWY4_WADCW Uncharacterized protein OS=Waddlia chondrophila (strain ATCC VR-1470 / WSU 86-1044) OX=716544 GN=wcw_1293 PE=4 SV=1

MSLSLDRLNSSLQPLIFATNKKELLECYQYAKKATESKNLSDLINKIHTALKENKNIDSL

QRLSKLEKGLRCLRDYHKIVSHSEKKLLTRLFIWIFPKSERAQKIMEQDKSTFQHSELLL

HVVEGKEEKNRLKQFSLKIHLGTKKDPRKAFISSLIRNPTFQENAGIAITKLINPESDQK

SALKDLHKMMLTLFQRHGGENIPKADLSDIYAAAIIASEHYLSSYKVGQLVELAYTPMDL

DPLSEENNALSQLLLVFATVDGEETRKIMDNTTPLSSYFLAKKTPSLKKMLSEAYNNIWK

AKSDRHNQFILRLIDDKEFQEISKQVINDLTNPKRPIEERLQKIAEIMNAQEDKYLQPRK

GEDKLREEDRIEYHLDFYLAMWIAARDQVDAKALFGAQTLLAMRLKIEPTGKESNTQATM

STALTFVEEMSKSKWLERQE

>tr|D6YU36|D6YU36_WADCW Uncharacterized protein OS=Waddlia chondrophila (strain ATCC VR-1470 / WSU 86-1044) OX=716544 GN=wcw_0272 PE=4 SV=1

MTKKILMFAALGIAMLSVKGLSADDRYVRHLDANGNGIYEEGEVDPCREYRSGPMRCTYP

VTKFKKKTYCTKRCVKEPYTVRKKCVRYKPEYYTKTYCRQVPETYYTCETRYRDRWVTDE

RCCYEPYTYYKTECINNPPADEGCPSDGCPTGGCPINNRSAAHYNHATQSQSDETPISNA

QTRKSRHY

>tr|D6YVD7|D6YVD7_WADCW Riboflavin synthase, alpha subunit OS=Waddlia chondrophila (strain ATCC VR-1470 / WSU 86-1044) OX=716544 GN=ribC PE=4 SV=1

MFTGIVKGTFPIVDIIRERGFSCAVLLPSELREKLAIGASVAVDGVCLTVVEILGDRVYF

DLIEETLKVTTFNAMEIGRLVNVERSLKFGDEIGGHLLSGHVMATAEIFEKQTRNEETIV

KFLVPERIEKYFFKKGFVAIDGISLTIVEINPLSVHLIPETLRMTTLGLKQPGDRVNIEV

DYQTQVLVDRHSM

>tr|D6YUA4|D6YUA4_WADCW Aspartate carbamoyltransferase OS=Waddlia chondrophila (strain ATCC VR-1470 / WSU 86-1044) OX=716544 GN=pyrB PE=3 SV=1

MAYKSRDIIHIADFSRDEILHILDTAEKLKRSPQSDLLRGKILGSCFFEPSTRTRLSFES

AMHRLGGSVIGFDNASNISTRKGETLRDSMKMLDYYADIAVIRHPLEGSAQLAADSIEIP

VINAGDGSNQHPTQTFLDLFTIRETQGRLESLSIALAGDLKHGRTVHSLAEALISFQCRL

YFIAPESLEMPKPLCDHLKENGIKFSFHRSLEEVMPKLDILYMTRIQEERFQDRKQLQTV

NQSIKATLQLLEQSRPNLKVLHPLPRMQEIDPQVDNSPKAYYFQQAGNGLCTRQALLALT

LLGEI

>tr|D6YWL8|D6YWL8_WADCW Putative secreted protein OS=Waddlia chondrophila (strain ATCC VR-1470 / WSU 86-1044) OX=716544 GN=wcw_1172 PE=4 SV=1

MTIQRFAFMAVCGMSALFSPASVSATEISQGKMMSYFPEKFVAQTLERYKVPQGQRIAIQ

NALIDKDQEVISLVEEKAAKMNPNPLRDPKMRQEAVKIFRDSLYEVFSKVMQAHGVTDKD

ELHAMLDDIQRQKAEYFAQSIEEQKEEAKRQREPSSHSLQRQPRGIKSSNDL

>tr|D6YTP7|D6YTP7_WADCW Methyltransferase OS=Waddlia chondrophila (strain ATCC VR-1470 / WSU 86-1044) OX=716544 GN=yhhF PE=4 SV=1

MLQIIGGKLKRKKLKSPKGLNTRPTSSRLRETVFDICQQEIERARFLDLFSGSGSMGIEA

LSRGAGSAVFVDHDRGSIRCIQENIQELGLADCARAVIGDVIKLLPKLGTFDVIYVDPPY

FEKNRDFSHSAEVLKAIDQSDLLAHGGMLFIEDSRSWEPDSGSLKTLRLKSSRKVGRSML

HQFER

>tr|D6YV21|D6YV21_WADCW F-box domain-containing protein OS=Waddlia chondrophila (strain ATCC VR-1470 / WSU 86-1044) OX=716544 GN=wcw_0614 PE=4 SV=1

MIDFESISWIVPKEQDHQSLSESPFSMIPEPIILKIFKKIGEENLNRASSVCHLFHRISY

DRTLIVKDVRAAVALLKYLFSIIQEGNTLSGELERLMESKDLLPLKLMKLIKWSEHLLLN

LPFDAAVKWSPPVSSHRVQAFTNNTYKQFWNVFIEKNLKEYRSINLNAFFQDWAFDQCLE

ALIEKKKVPGLLVGELLSKQHVEKLAEAIESGCLEVGQCSFFGAALLEALLQALSASSAL

KKLSFSMCNLWDADIKKIAQLFQQHESLEEISIKKFAFSLNGIKNLLKMCRLAPSLSVLR

LEECSDEELRALVDRHGKSGLRELHLSCLSLSLQTESAIIEKFKEASTVDRFILYYRDYS

ARFSFERMEKCLKGSFYYRVKERGSHQIEIAKL

>tr|D6YRR8|D6YRR8_WADCW Putative membrane bound phosphohydrolase OS=Waddlia chondrophila (strain ATCC VR-1470 / WSU 86-1044) OX=716544 GN=wcw_1413 PE=4 SV=1

MTSQNENTDASEFKFSNEQGYFDKSLGIKLIIGVIFALSLFSILHFREVRVEVLELGSIA

PSYIVAQTDLEFYDEEATIILRQEAVRDIGKIYEISDKQIKLVRGEFENFLIKDESWRLE

LGDTAFDEMYKGADALDRALVSIRFTDSRTLQKMNELSLPTSSYIIYTPLQTDAPVSLPK

STWHLAAQMAFPPEKFHSDTDDFIIDYFKKRTWELIEDIPQQRAVRTRIQKSVPEKYTKI

SAGSRIIDQGDRVTSRHVAILQAMKNAMNEQRNLWHPSTLAGSLMLSLLLTAICAAFVHV

HYPEIMASNRKLFLIVTVIVLTFIIAKSVEFFFLTNKSNLIEVIRYPLFVPFSAILLCSL

LNPAIATFTSAFLTIILMMGLAFERQGFMILNLATALIAILSAKGLRQRREVFVVCGKAW

LCSMVVIISMHLYQSTDWNGFWVDIFSAGGFMLLTAVLVVGLLPLLESGFKIMTDVTLME

FMDPNHDLLRRLSIEAPGTYQHSVVVGNLAEAAALAIGANGLFCRVATLYHDIGKMATPQ

YFSENQHGEGMNIHQLLTPQESAQVIIAHVSEGVAMARKANLPEQFIDVIKEHHGTTLVY

FFYRKMLKMNQEGAEVVNLGDFRYSGPEPRSKESAIIMIADSLESASRSLDEFNKETVWE

LTSRIIKEKSEEGQFDHTCLTLQELATVKETLVKTLVAYGHSRVKYPTREIGEETLKSEV

NEF

>tr|D6YUG1|D6YUG1_WADCW Glycerol-3-phosphate acyltransferase OS=Waddlia chondrophila (strain ATCC VR-1470 / WSU 86-1044) OX=716544 GN=plsB PE=4 SV=1

MDFQQRLQEIRSDFAYPKILLDSLEAFYSSYAQAVSQNGVDVADCQEPLLTFLDLLEAQL

KSPFIFEPFHKKIRKPKDLYRFGIDFIRPLVQLEKSKILGLDIVEEIETGMKRGENAILF

ANHQIEPDPQAISLLLEKTYPDLVDRMIFVAGHRVTTDPMAVPLSMGCNLLCIYSKNYID

HPPEEKPRKIAHNQHTMKTMRDLLTEGGQCIYVAPSGGRDRPNAAGVVEVAPFDHQSIEM

FHFIAKQSKTPTRFYPLALNTYPLLPPPDEIKKSLGEKRLPNTTPIHLAFGKEILFEQLP

ISINLDKKAQRIERARCIRSLVDQLYQLIV

>tr|D6YTQ8|D6YTQ8_WADCW Uncharacterized protein OS=Waddlia chondrophila (strain ATCC VR-1470 / WSU 86-1044) OX=716544 GN=wcw_0144 PE=4 SV=1

MKGKAMAAALAAAMVFSPLMAKERSYSNKYQQWGESHQQCGLFKGLQKKPMTETTLLLQL

DKEGRQKFKNLPSQTREEVLQTVNQTGTSDYKKIINEAIESQRLKAGIEQERMLHNGMDR

IQR

>tr|D6YSH5|D6YSH5_WADCW Uncharacterized protein OS=Waddlia chondrophila (strain ATCC VR-1470 / WSU 86-1044) OX=716544 GN=wcw_1676 PE=4 SV=1

MIQDKHQTNGHELENLIDSAIKKVGGKKENDICRYLPTGSGGYIHHFTMRKMKHENPKKL

VTMIRQHILNEEKPTAVPPKPRAARGSRKRRDQFLFTKEDIERMLNMARLAGDKEMIRKL

TPRKDLKQIKRELISSIRHNQIDHELWNCYVETVTTAEAISSNSQ

>tr|D6YT24|D6YT24_WADCW 1,4-alpha-glucan branching enzyme GlgB OS=Waddlia chondrophila (strain ATCC VR-1470 / WSU 86-1044) OX=716544 GN=glgB PE=3 SV=1

MKEAVAVYSLLSEKDIYLFKEGTHDRLYEKLGSHVVEVDGIKGTQFAVWAPNAESVSVIG

DFNDWDRKRHQLHVRWDESGIWEGFIPGAKQGDCYKYHIASKVNGYRVEKGDPFAFFAEV

PPKTGSIIWDLAYEWKSLTEERSRSDKPISIYEMHFGSWRRDEKNTPPSYREMAEWLPPY

LLDMGFTHVEFMPMMEHPFYGSWGYQKTGYFSPTSRYGSPQDFMFLIESLHQAGIGVFLD

WVPSHFPSDQHGLAFFDGTHLFEHEDPKKGIQPDWNSYIFNYGRNEVRSFLTSSANFWCS

RYHIDGLRCDAVASMLYLDYSRKNGEWIPNAYGGNENLEAIDFLKTLNTTIYQNHPHVQM

IAEESTAWPMVSRPVYLGGLGFGWKWNMGWMNDTLEYFKKDPVHRKYHHNELIFSILYAF

TESFVLSLSHDEVVHGKASLLTKMPGDEWQKFANLRLLYGYMFTHPGKKLLFMGSEIAPW

TEWHHEAGLEWHILDYERHAGIQRWVRDLNKAYCTLPALHQLDFSEEGFSWIDCSDWENG

VLSYSRKGFDPKDTAVVILNLTPVPRENYKIGVPFSGNWQEVLNSDSHYYGGSDKGNFGR

VASYPIAMHGHDYALSLTLPPLAMICLSPE

>tr|D6YWF2|D6YWF2_WADCW Cation efflux transporter OS=Waddlia chondrophila (strain ATCC VR-1470 / WSU 86-1044) OX=716544 GN=czcD PE=4 SV=1

MKSLSLYRKSLDERTMSQFPDPVPLPENVPKARKNRNSHLIRAARWGVLIRLIIIIIELL

GVAHFGSSALLMDALASSLDIACSLILIVFIKLAAKPPDEDHPFGHGRYEPLAGLQLGVF

MVLVGLGMAGQQLFQLMEDVPSSPIDSNVWLIPLAATLLLEACYHVISRVAIQQNSPALS

ADAAHYRMDALTSLFAAVTLIIAAWFPDWSVVIDHIGAIFIAVLMVAIGSFAARSNLHQL

MDKVPSDNYFEVVKKAARAVDGVLETEKIRIQLYGPDAHVDIDVEVSPELPVDQAHRISQ

KVRRNIQKAWPAVREVTVHIEPFYPDDH

>tr|D6YU65|D6YU65_WADCW Putative biotin-[acetyl-CoA-carboxylase] ligase OS=Waddlia chondrophila (strain ATCC VR-1470 / WSU 86-1044) OX=716544 GN=birA PE=4 SV=1

MLYKTFHFPNIDSTNTWALSNTHLFPRDKITLVYAKKQSEGRGRHKNQWTSPNANNLYAT

FGVFIPLEQGVQPNLPQLIALATISVMEKMGVQPKIKWPNDLLVQQKKIAGILTETSIDD

QHIFHAVGIGVNLNSSPDLPRPVTSLSAEIEREIDLEEFLDQLKETFSRFLSVFLDAGFS

PYLPAFKAHLNHHENQELSFHEKDCQWKGKFHSINDDGTLNLKLETGEVRRFIAGEILD

>tr|D6YVH8|D6YVH8_WADCW ATP-dependent RecD-like DNA helicase OS=Waddlia chondrophila (strain ATCC VR-1470 / WSU 86-1044) OX=716544 GN=recD3 PE=3 SV=1

MDQIKGYIERITFQSRENGYTVVKMQQPGQSDLTCVVGFMPSVQPGETISCTGSWKKHPA

HGLQFEAKEYRTEAPADLNGVKKYLGSGLIKGIGPVYAERIVSTFGTETLDVIDLEPERL

LEVPGLGEKRVDMIRQCWEEQKSVRDVMIFLQAHNVSPVYAQKIFRVYGKKCIQVVSEAP

YSLAKDIHGIGFKTADKIAEKIGIEKNSPQRIEAGILYVLSELSNDGHVCFPRDPFIEEA

AKILEVDSSLIIKNIENLQEESIVIQEQVHHGALQTFLWLKPLYIAEKGIAKELLRVMQG

ASHLRRIDGEKALEWVQEKLSITLADNQKTAVFLALSEKAQIITGGPGTGKSTITNAILT

ISSKLSDKILLAAPTGRAAKRMSEITGKKAQTIHSLLEFDFRAGGFKRNGKNPLDCDLLI

IDEASMIDTLLMYSLLKAVPDHSRLIFVGDIDQLPSVGPGNVLKDMIASRTLAVTQLNQI

FRQASGSRIVTNAHRINKGIFPELYNGKESDFFFIAEEDKDALLNQILTLAAQRVPRTYH

FDPFTDIQVLAPMKKGVIGTDNLNHLLQETLNPSQNHIYKYGRRFAKGDKVMQTRNNYDK

KVYNGDVGRIEKIDETDQKVYVKMEDLLISYDFADLDELVLAYAVSVHKYQGSECPCIIM

PVHTSHFKLLHRNLLYTGVTRGKKLVILAGTKKALAIAIHNDEVKSRYTGLLASLLEKHN

PALD

>tr|D6YTE9|D6YTE9_WADCW DNA helicase OS=Waddlia chondrophila (strain ATCC VR-1470 / WSU 86-1044) OX=716544 GN=recB PE=3 SV=1

MVRAGKAVVFPADCPVERKEEMNRFDILNRETKIDSHIILEASAGTGKTFSIENLVVRLL

LEGEHPLRIDEILIVTFTKMATSDLRVRVRDTIENVVNALEKGVLGRFDYLEPIDRDERK

KRPAIRLLERALIGFDEAQIFTIHGFCYRMLAEHGMDGCVHPDPKNEGKGIREDTYKKCV

MDYFRTGLSDERIGLQHRNFALSSQRGSVERLEKTLGKLIAEGVEIEKTPTRNEFFIHFK

DRIERLKKVHCLEPIKLREDLEVFSKSMKVNGFDKSQLIILPEILKKNGVDESDFECLVE

NGHGLLHFLNPANKKVKFPADDQFHYPNLPEILNDELCQFKYKNYGIARMAYDCKQLMKR

QFEEEELHNFDDLLELMRQKLKEEKFLKDVRSRYRAAIVDEFQDTDPRQWEIFKKTFPPD

DSGWGRLYLVGDPKQSIYAFRQADIYTYLEAADAIGRENLYSLDTNYRSQPSLVKALNHL

FSAPFSEGWMPLPRLNTYMNVPTVQWDDSKIDRVFQDNLSALHWCIHQTEKFSVTNSEDE

AFFPFIVQEIQRLRKEHSIPLNSFAVLVADRHQAKRVSEYLKRWNLPSQRQRVEPISESM

VIPSMRELLYGIMHHRDESSMKVALGGPFLRWTADEILQLDEDSVYAEVQARFRELDEIW

KSDGLSVCMEKLFMSRWKDSSKTVLETLLEDENGSQFYADFQHVMELLLDSPKTPSEAIS

FLEKFLFDVSKSEEELKRRLDTGQDCVQIITIHSSKGLEYDIVFALGLVNRPKKDDLYYI

ADGYLRFVLDQESADFVKYRDEKDAEKMRSLYVACTRSKYRLYIPYIDCKSNGASGTLSP

MELFVGQFSKPLESFLKESSENRLLSYTVLNDVDFDLLKEEDLEAPKLMAPTEVVVRAVS

KMMTSFSGLANVVASDQSGAPHQFDNGIKSCHTLPSGSATGTLLHTLLEEIPFEVICHDA

VRSITPFIKGTQYEEWKDVLAEIVFHAVSTPFIKGCALKDIDPNQCKFETEFLYSYNLES

YMKGFIDLMFAHEGKFYLLDWKSNWLGTSSEIYREDVLVDSMNHHDYFKQAEIYTEALKK

YLEISHKKPFQEIFGGVYYIYLRGLPKYGVKFIPPSFFYS

>tr|D6YUJ5|D6YUJ5_WADCW Putative tetratricopeptide TPR_2 repeat protein OS=Waddlia chondrophila (strain ATCC VR-1470 / WSU 86-1044) OX=716544 GN=wcw_0434 PE=3 SV=1

MFTQIFYLTVVLLIISFSPQHQQPPLTLSPLPAFLLGSAAYIFTLILIALQNILFSKWLR

NTHHILLATANALLILFLLAIHSLLIFPSILLVSAASLTLYFGGLYVFHRSAYPRIPAGS

RGLASSANLYAINHLRFLLPFCLPFLLIACIMDAITLIPADKVELSRSYSALFTIGLLAL

LLLIVLFYPPLTIRIWKCRDLSSSKLKQNLDALCVKAQFKHAGIKTWTVLNHSYTAAIIG

ILPRFRYIMFTKRLLNELSPRSINAILAHEIGHCAHKHLLYYPAVIFGILVLFALGTPLL

EPIASSFTFFALYSLVFFIYLRVVFGYFSRLFERQADLHVYQLGIPVEDMQLALDEIGHA

TGGTHQVPCWHHYSIQKRIDFLEATKKDPLLIAKHHQHVRLSLILYFILLGVGIIFTYKS

FF

>tr|D6YSI0|D6YSI0_WADCW Single-stranded-DNA-specific exonuclease RecJ OS=Waddlia chondrophila (strain ATCC VR-1470 / WSU 86-1044) OX=716544 GN=recJ PE=3 SV=1

MISWNFEPDNPMWVYPKMDKRWQDDIIKEFKIHPVTAQLFVSRDFTTVDQIHQYLYAKLP

DLHDPSLLQGMDKAVERIVKAIENEENILIYGDNDVDGMTGTALLTDYLRKVGANVYFYI

STPGTLRQNLIIEAIEFATKNQCKLLITVDCGVTAATEIAKVVEKGIDVIITDHHEPTDQ

IPHCIATLNPKLLNNSYPNRDLTGVGVTFKLVHALTNYLVKQGKIDSKKIDLKRYLDLVA

LGTVADMGQLLGENRILVRYGLEQLKRTKRIGLAKLISVCDMEPKQVSTFTIASKIAPRL

NSLGRIADPVKGVELLLIRNAKQAETMAQELDLNNLERQRIEKTMTADVDQIISLHPDIL

QKKAVVIHSDKWHPGVIAILCTRISKHYNRPTVMIAIENGIGKGSLRSIHEFPLLSVLRD

CSDLLVNYGGHDYAAGLTIKEENIPEFKRRFIEAANRKLSTHDVVTKLNLDSEVQFSDLT

FDLMESVKLLEPFGNENPQPLMYTRAKQAWPPKIVGKTHLKLYLEQGDRMLEGIAYGKAH

LSPLLRKKNIILEVAFTPQVNNFLGPSIQLLIRDFRIIEESQPNIPN

>tr|D6YT23|D6YT23_WADCW Maltokinase OS=Waddlia chondrophila (strain ATCC VR-1470 / WSU 86-1044) OX=716544 GN=wcw_1880 PE=3 SV=1

MTNWYKDAIVYQLHVKCFRDSNKDGIGDFKGLTKKLDYLQNLGCTAIWLLPFYPSPLRDD

GYDIADYLNVHPNYGSLKDFRTFLNEAHKRGLKIITELVINHTSDQHQWFQQSRKGKPGS

QWRDYYVWSDTPDKYSEARIIFKDFETSNWTWDPVAEAYYMHRFYSHQPDLNYENPAVHQ

EIFNILNFWMNMGVDGMRLDAIPYLYKKEGTDCENLPETHQFLKKLRRFVDQNYPDRMLI

AEANQWPEQSAAYFGNDDECHMAFNFPVMPRLYMALQMEDRFPIIDIMEQTPKPPPSCQW

GIFLRNHDELTLEMVTEEERDYMYRSYAIDPQARINLGIRRRLAPLMNNATQKIHLMNVL

LFSLPGSPIIYYGDEIGMGDNYYLGDRDGVRTPMQWNTDRNGGFSDGNPQKLYLPLVIDP

EYHHAFVNVENQEKSPSSLLWWMRKMISVRKRYKAFSRGDIHVLSPSNSKVLAFIREYEK

ESILVLINLSKYPVYVNLNLSQYSGGKLKDIFGNQEFCSIKEGSVGLTVGGYGYYWLAIF

PSDLELPHGDHLEELQLKKSWQDILLPEYEHTFCNRILMNYLPLCRWFQRKADKIIDISI

SDKTAFDSAIFLHLKVDYASHETEYYQLPISFKPGIGHEDYQHGVIAKVRFENTEGLLID

GIFDEEFRSHLLNHLILEKKQLKSRKGSITIQTTAALNKLGKAEGSQFSRVYSAEQTNNS

ISYGDAIIAKFFRKVENGLNPDYEIARHLTQSAKFKQTPRLLGSIQYTIKGKAPSILAMI

QEFVPNGGDMWQHSLDTIRLFFENALIREPGTNFDALEESIGTHFLQTIKLLGIRTAELH

LALSNDYGKEEFKPERFTWMYQKSLFQSIRSQIKKALYLLGQRYQEFDEADQRLAINLLD

KQSEFEKRIADLQTRKIEAKKIRIHGDYHLGQVLYTGNDLAIIDFEGEPVMPLSERKLKK

SPLQDIAGMVRSFHYASIWGFQLFEQYRGDSSSAVLPYRSRWYTTLKDLFISSYSTRLSD

AEPGMIPTNPEDFQTLLFAHLIQKAAYELSYEMQSRTGKVMIPMMGLTELLEEKSG

>tr|D6YRZ4|D6YRZ4_WADCW 50S ribosomal protein L5 OS=Waddlia chondrophila (strain ATCC VR-1470 / WSU 86-1044) OX=716544 GN=rplE PE=3 SV=1

MSRLKKYYREKVKEELQKKFDCSNPMTIPTLRKVVINMGIAEAAKDKNAIQDCINELSLI

SGQKPILTKAKKSISNFKLREGMPIGLKVTLRGDRMFDFMDRFFNIVCPRIRDFRGFNPK

GDGSGNYTLGLDDQQIFPELNLDEVKRTQGMHITFVTTAKSDEQCIELLRLLGLPFKKLP

VSVTI

>tr|D6YUV9|D6YUV9_WADCW Mutator family transposase OS=Waddlia chondrophila (strain ATCC VR-1470 / WSU 86-1044) OX=716544 GN=wcw_0550 PE=3 SV=1

MKNDVISLDKFHATSEMNSLLEQTLREGARLLLQQAIENEVNEYLESMKGRRDFEGRKQF

VRNGYLPEREVQTGIGPISVKQPRIRNREESSEGYSSAILPKYLRRVPSLDAVIPALYLR

GISTSNFQDALEAIMGKDAKGLSAANITRLKQSWEQEYKDWNKRSLEGKRYAYIWVDGIY

FNVRLGDDRICFLVILGALPNGKKELVAIHNGYRESKISWTEVLESLKRRGLCTAPELAI

GDGALGFWSAIEEVFPKTKQQRCWVHKTANVLDKMPKSIQVNAKKAIHEIYMAPTKEDGL

AAFEVFLKTYRDKYPKACACLEKDKAQLFTFYNFPAIHWQHVRTTNPIESTFATIRHRTR

QTKGCGSVPATLTMVFKLATTAEKKWRKLKGCEMIEKVINGVVFKDGEEVLEKEKVA

>tr|D6YRX0|D6YRX0_WADCW Threonine dehydratase OS=Waddlia chondrophila (strain ATCC VR-1470 / WSU 86-1044) OX=716544 GN=ilvA PE=4 SV=1

MNIRLFKEARERIAPYIIETPLIQSLEFPGLYLKCENFQWTCSYKPRGAVNAALQKIQPG

VGIVARSSGNFAQGIAYAGNRLGFPVAVVMPEHAPALKVKKTEALGAEVFLHGTTHAEGD

EKVAELVCEKERIMIHAFDDPDVIAGQGSVALEVCDQLKKPHFFFGPIGGGGIMAGCSCV

IKQISQHTKVIGVEPEGAARLTASLEKGERLHLPSTKTIADGLLSPSVGKHNWPLLKKYI

DSTVKVSEQEIIEAMKILFNVFGLVAEPSGAVSFAGFLKKRPLSGSVVCVITGGNVDRQK

FLEWLHG

>tr|D6YU77|D6YU77_WADCW Putative phosphatidate cytidylytransferase CdsA OS=Waddlia chondrophila (strain ATCC VR-1470 / WSU 86-1044) OX=716544 GN=cdsA PE=4 SV=1

MLASFPPIFQRVIASTIATAFLLFALYFSFTPYLSLLVPILAIVCFGAALKEYYSIARRK

GFKPREKTAYSLGLAYILAIYLGSLSGHQDSFPLYALAIGLFVLFSAFFFDGKNPLVNIA

ITLFGIVYLIIPLSCTLQINYFFPPESAEDGRWWVLYLLCTTYLTDACGLFAGQTFGKHK

MAPVISPKKTWEGAAGGLLASIIASLIFSLYAPISLSVVQSIILGACIGTIAQVGDLAES

LLKRDVGVKDSSKIPGLGGMLDVVDSLVFTAPLLYLYLKA

>tr|D6YSP3|D6YSP3_WADCW CinA-like protein OS=Waddlia chondrophila (strain ATCC VR-1470 / WSU 86-1044) OX=716544 GN=cinA PE=3 SV=1

MKVELIVIGSEVLSGHTVNTNLSFIGQTLDRAGYTLSRETVLPDNHEDLKSGFAEALKRS

DLVVAAGGLGPTIDDVTRHAAAEVFESSFHFNETVAKHLRERFGEKFPTVEDQAAVPEKA

IPLLNNVGTAPGLIFQEGKGTLILMPGIPAEMCPILTEQVLPFLKKKFPLKEQIYRRSLH

FFELSENVVDGVLRTLVQKYPKVDFGIYPGMGVVSVTLAAKAKSASDADLLLRKPYSALE

EHFATNCFKSDSGQIEEAVQKLFIEKGWTLSCAESCTGGAVAARITQHSGSSGYFLGSIV

SYANELKTAALKVPAELIQEKGAVSEEVVSSMVKGALDLTGSDFALAVSGIAGPLGGTPE

KPAGLVWCAVQHKDGEPHLWKLNHHLPRQLVILRSVNSLLSNLILYSKQYNV

>tr|D6YSR9|D6YSR9_WADCW Replicative DNA helicase OS=Waddlia chondrophila (strain ATCC VR-1470 / WSU 86-1044) OX=716544 GN=dnaB PE=3 SV=1

MAPQNLKVKIPPNSKEAEMMVLGCMLTSINALNIAADKLHDFDFYFTEHKLIFNSLKSAY

KSDKPADIHIICEDLKRQGQLEAAGGAAYLTTLAQFAGTSAYIEEYCRIVHSKSVLRRII

NTSHIIEKNALEEPDDVENVLDEAQKLLFEIGKSANSGDAVLLSEIITGTKSESGVHYLK

ELQERQEKYQERGDEDPGITGIPTHLADLDKMINGFNNSNLMILAARPAMGKTALAINIA

ENICFKNKIPVGIFSLEMSAEQLVHRLICSQAEVESDKIKTGSLDGHDYQRIVETINHIQ

DHPLLIDDQPGLSINDLRARARRMKETHNIGFLVIDYLQLLSGSGNNKNGDNRQLEISEI

SRNLKNLARELNLPILCLSQLSRKVEERPGHRPMMSDLRESGSIEQDSDLIFFLLRREYY

DPMDKPGMAELIVAKNRHGSIGTVNLTFRKEIAQFANYTPIGMDHEPQGGKHPYEGSSNY

ATF

>tr|D6YTK0|D6YTK0_WADCW FeS assembly ATPase SufC OS=Waddlia chondrophila (strain ATCC VR-1470 / WSU 86-1044) OX=716544 GN=sufC PE=3 SV=1

MLLEIKNLSASIDGKPLLKGVDLEVNPGEIHAIMGPNGAGKSTLAKVLAGHPSYEVTGGE

VWFKGQNILEMEPDERAQLGLFMSFQYPVEIPGVSNMQFLHASYNAIKKASNQPELEEGD

FEKLLDEKMKIMDIRPEFKQRNLNEGFSGGEKKRNEILQMAVINPSLAILDETDSGLDID

AMRTVAGGVNHLMNDDMGLILITHYQRLLDHIRPHKVHVMVNGKLIESGGPELAIKLENE

GYDWLVKNIQEEMAG

>tr|D6YSS0|D6YSS0_WADCW tRNA uridine 5-carboxymethylaminomethyl modification enzyme MnmG OS=Waddlia chondrophila (strain ATCC VR-1470 / WSU 86-1044) OX=716544 GN=mnmG PE=3 SV=1

MWKYPAVYDVIVMGGGHAGCEAALASARMGAKTLLLTMNLDTIGKMSCNPAVGGIGKGHM

VREIDALGGEMGKVIDCTGIQYRMLNATKGPAVWAPRAQADKAAYQFEIKHRLEKQENLE

IHQGTVEEIFVENDTVQGVATKEGIYFTAPTVVISSGTFMRGLLHIGERNFSGGRAGDQP

SVGLSGCLKKLGFHLDRLKTGTPPRINKRSINLSLTEEQPGEEGIRFSFDDEGKRKLPQV

SCYITYTTQETKQIILDNIHRSPLYSGKIQGVGPRYCPSIEDKVVRFSDKERHQLFLEPE

GLQTEEIYVNGISSSLPLDVQYAFIKSIPALRNAEITRPAYAIEYDYVTSGQIKPSLESK

KVEGLFLAGQINGTTGYEEAAAQGLLAGINAASKVAGREPLILKRSESYIGVMIDDLITK

GLDEPYRMFTSRAEHRLLLRQDNADLRLRKYGYAYGLIDQKRWETLCLKAETIEWEMTRF

EKTFKQVNGKGFSLAQLLRRPEMTYESLLETYPEAVVDHGEEINFQIELNLKYSGYISRQ

NTEIERISQVENLRVPEHFDFNQVSGLRNEAKQKLCRHNPLTLGQASRISGVSPADISVL

IVELTKRERQTIA

>tr|D6YWC2|D6YWC2_WADCW 3-deoxy-D-manno-octulosonic acid transferase OS=Waddlia chondrophila (strain ATCC VR-1470 / WSU 86-1044) OX=716544 GN=gseA PE=3 SV=1

MYDILLCLVSLIALPKLLYQMAFHKKYRNSLKQRLGIGFPEIEKGNKKLIWVHAVSMGEA

KAVAALARELKKRSDNDAILLFSTVTETGLAEGEKELPEADYHVFLPLDFSWIIRPIIRR

VRPDQVIVCETDYWYNFLSSSKNAGARLSVVNGKISERSMHRLLKFPKFTKRLFCLIDKF

CVQSHHYRERFLKLGIPEEKIVITGNIKFDNSFPKLTEEELVEWKSKFGIRPEDHVLVAG

STHDPEERIILDACFEVWKDDPHLKILIVPRHPERFNEVAQLLKKRGVEFSRYSEGVSHE

APVILVDAMGVLLQCYQAATLAIVAGSFTPKVGGHNIVEPCWYGVPVLFGPYLYSQPELL

ELVQEYGAGVQVEPEHLSDEIKGLLSEPKRRKALGAAGIHLADSLQGATERTLVEIA

>tr|D6YWB3|D6YWB3_WADCW Uncharacterized protein OS=Waddlia chondrophila (strain ATCC VR-1470 / WSU 86-1044) OX=716544 GN=wcw_1065 PE=4 SV=1

MNIPVQLALTIGQEMRNDALFKEIPGKGRHFDKNRIKALNLADRKLCYQALTVIEHAKLD

DFNSESPPENLSKRVREVTAAVTGKLSIHETPSPVWYKRPFLWIARCIHSLVLWFKNTFL

GRTSSSKLHNKVETYLNDYNRAQRRIALLAADEGLITKAEGKLNQCADEQKTFLESFNIL

LEVHETLRLIDFIIEKNEESKAKGTENFRREIQNAWIVDKINSDVIPLTHFKEVIVRKVS

EKTQEVKEYLKDLVEAAGQEDRDDEDVLNLLEQAGKRVTEEMKEQFKTGNQHRMHVEIAH

KHLCELKGELELLLKKYPK

>tr|D6YUQ9|D6YUQ9_WADCW Uncharacterized protein OS=Waddlia chondrophila (strain ATCC VR-1470 / WSU 86-1044) OX=716544 GN=wcw_0499 PE=4 SV=1

MSIQGDYPFSMSPLLDSGFKTWTVDFEETIQYQMVYVREFSEDKKQVMIRTLDWENDECL

TKARNLVANRLIGALQTNKEGVVLWVSDKLKPNFEKGRELKVVYQGKDTSTDICDLRGIA

FLNETKFAETTPWFREFCIKSAQRRTEKSSV

>tr|D6YSS8|D6YSS8_WADCW Putative succinate dehydrogenase cytochrome b-558 subunit OS=Waddlia chondrophila (strain ATCC VR-1470 / WSU 86-1044) OX=716544 GN=sdhC PE=4 SV=1

MAVETSVPKAFFWRRIHSLTGIWLVIFLLEHLLTNSQAALWIGDDGSGFVHMVTKIHNLP

YLPVIEIGLLGVPILIHGIWGIQYLRDASINSFKTDGSRPSLHMYKRNKAYTWQRITSFV

LLFGIIAHVVQMRFVNQPISATVDGQHHYMVRVGFDEGLYTLSKRLDFKVYNEQAVEVER

KMAPDVKEFLGDNWLSFFDFRKIPEGYELLERQKVLEKDEFVRALTARPLENGEVIVAAH

DFATAMLLVVRETFKWPLMMVLYTIFVLSACFHAFNGLWTFLITWGVSLTERSQKLMSGI

ANTLMIVVAFLGLAAIWGTYWVNLRF

>tr|D6YUF0|D6YUF0_WADCW Putative 4-amino-4-deoxychorismate lyase OS=Waddlia chondrophila (strain ATCC VR-1470 / WSU 86-1044) OX=716544 GN=pabC PE=4 SV=1

MFGDGVFTSVKVENGRLLHWDRHCERVTAQCEALHIQPPDIKQSKVEELIERNGAEQGVW

KLKIVITGGIDSRLSLPTRTYGTYLVTLEKYEMPEGKAKLCRYPYPIESPASSLKTLAYL

PRLMVKQYALERGFDDAVVCSSEGWVLESAFSNLYWEEGGAVFTPMSTLPLLAGTYLTSI

DHLKEERITYEQLKEKQAVFICNAMGSRLSEII

>tr|D6YVA4|D6YVA4_WADCW 30S ribosomal protein S16 OS=Waddlia chondrophila (strain ATCC VR-1470 / WSU 86-1044) OX=716544 GN=rpsP PE=3 SV=1

MALKIRLRQQGRAKRPFYRVVVTDSRSPRDGRYVETVGWYNPLESEAEKNLNIKGDRVEH

WIGQGAILTEKAHALVARANPEIVKTIKDKELARKQKTVAKRKARKAAK

>tr|D6YVQ3|D6YVQ3_WADCW Putative type III secreted protein SctW OS=Waddlia chondrophila (strain ATCC VR-1470 / WSU 86-1044) OX=716544 GN=wcw_0848 PE=4 SV=1

MFGGGAGGIQGPHARQTLEAMKEVGKETAEEVRQEQKVAAQSAQASNALTAFRPKVKTEK

KKLKQISTTVAKMAKMKKEKKLLPIQQIAKKAEEHERKNPELKAKVLKLLRERIKPDSSS

EDIQEILDEFYPDPTLADDAMEFLLDTTEGQLHQQVQEAKEKLNEDKGREITAGRNISKQ

ARQASDKGLGTPTSLREMYRDITGNPRDSNTMFQELSEKYAFKEMHKVVKFLLHSLGSDM

KSKGPSISRGELHRLITETRSLQAILGVYRFFKTRMGLMEKMFAKSGLDMPKKLNFELMS

KCFMNLAGERYPSSDKVLAQAKRLGIEDWILAKIIAFSQFRDSIKEMAMSQIYRSLQHRD

ELLFAIIEALEELEDQLEELEEQDEEEEDWEEDEEEEEK

>tr|D6YTP2|D6YTP2_WADCW Two-component response regulator phoB OS=Waddlia chondrophila (strain ATCC VR-1470 / WSU 86-1044) OX=716544 GN=phoB PE=4 SV=1

MQKSRLLLIEDEEDIAALIKLQADISGYKLHVEVDGLNGLLAIEREKPDLVILDIMLPGQ

SGLDVCRKLKNNPETKDIPVIMISAKSEELDVVLGLELGADDYVTKPFSPKILFSRIRAV

LRRGKEPEKGPKIIKFGHFALDTESYQVRKHDKQLHLTLSEFGILKRLVSNPGKVLTRNQ

LLDDVQNEEAFIVDRNIDVHIASLRKKLGPNFHWIETVRGVGYRFKEEEED

>tr|D6YST5|D6YST5_WADCW UDP-glucose pyrophosphorylase OS=Waddlia chondrophila (strain ATCC VR-1470 / WSU 86-1044) OX=716544 GN=glmU3 PE=3 SV=1

MDKRHAFEKLHSIGQGHLIQDYDQLSKHQQQGLLEQIERLQIPVFRKQQLLLMPTPHSPI

RFLDPVLNSAAYGENSWSEQGKGAIKQGLVGALLIAGGQGSRLRFNGPKGCFPVSVIKKK

SLFQLFAEKTLAASIQANRPLPLAIMTSPLNTQATISYFENHRYFGLEASQVSFFAQELL

PFLDDQGNLVPDPMGNIAEGPDGNGSCLRNFFDSGIWDIWYGSGVRLVNSVLIDNPLADP

FDAELIGYHLDENADVVIKCTTREDPKEKVGLIAKHNDRIEIVEYTEVPEEVRNKKNDQG

GLLYNLANLSLFSFSMDFIKSAAHKDLPLHRARKSAPTAKDPSPEKPNIWKFETFIFDTL

QYATKIKTLIYPRDSSFSPLKNRNGRDSLETVQQALLQRDRKIFQQITGTKPPDKPFELS

QQFYYPTQEFAAQWAGKSFPNAGYIE

>tr|D6YW28|D6YW28_WADCW Putative ADP-L-glycero-D-manno-heptose-6-epimerase OS=Waddlia chondrophila (strain ATCC VR-1470 / WSU 86-1044) OX=716544 GN=hldD PE=4 SV=1

MSILQMKGKILVTGGAGVIGRPLVGKLLQNGAELSVIDLAERPKEWPKEVCYLQTDINQL

DKDQLLKINPDYCFHLAASFERSEESPEFFQKNFYNNILLSHALLYHLQRCDCLKKIIFA

SSYLVYNPEDYFFKTPPKKRVNLSEVSSLSPRNLCGNAKLLHENELNFISRHSSFKAVSA

RIFRVYGKGSHDIISRWTRLLIAGKAIKIYGKESVFDYIYADDVAEGLIRLAESEIEGVV

NLGTGKSRRVAEVLAILKEHFPEMDVEDEEHSSLYEASQACMEEFVKAVKWRPTTTLEKG

IPKIIAYEKTTGLK

>tr|D6YSP5|D6YSP5_WADCW Lipoprotein signal peptidase OS=Waddlia chondrophila (strain ATCC VR-1470 / WSU 86-1044) OX=716544 GN=lspA PE=3 SV=1

MKKKTKRKKKTPQERSISLPWIPLLVGIAILAADQLTKYFVHTEMPRMTHEAQWYPYNGV

GVFENFLGVEFSIVHAVNFGAAWGMFSNFQVPLLILRVVLIMGLCLYLILFNKNKSLILP

FLCVIAGAAGNVIDYFIYGHVVDMFHFVLWGYDYPVFNIADAFICIGMAFLILIPWCQSK

GYTLCKS

>tr|D6YTM5|D6YTM5_WADCW Putative membrane protein OS=Waddlia chondrophila (strain ATCC VR-1470 / WSU 86-1044) OX=716544 GN=wcw_0111 PE=4 SV=1

MCFSAEASFTVGAILSVVGITTLKQVKKQKYFLVALFPLFFASQQLIEGIVWLNMHPSFV

STPLSQAAVNLYLFFAWLFWPIFVPIAFFVAEKEKWKKILFLTVFLIGLIISYIDIIYLI

NYRITPQIVGRSLDYGFTPLYGNILYGLIVFIPIFLSSVPRMKIFGISLLIAFIISQLIY

TYAFTSVWCFFCAAISIQLYQILQRASQEKKVS

>tr|D6YVT9|D6YVT9_WADCW Peptide methionine sulfoxide reductase MsrA OS=Waddlia chondrophila (strain ATCC VR-1470 / WSU 86-1044) OX=716544 GN=msrA PE=3 SV=1

MKRYHPLNPEEEKIIVHKGTEYPGTGKFEQTKESGIYLCRRCDAPLYLSSDKFSSGCGWP

SFDDELPGAIQKQVDADGKRTEILCKRCGGHLGHVFLNEGITKKNTRHCVNSISLDFIPA

FTKEGHGKAIFAAGCFWGVEHLFKDLPGVIASTVGYTGGKVADPTYQEVCSDRTGHAEAI

EVIYDKNITTFEKLTKFFFEIHDPTEQNRQGPDIGSQYRSEIFYLTDDQKKASEDLIKQL

KNKGFDVATRVTPASLFYPAEKDHQHYYDKTGKAPYCHFRTPRFE

>tr|D6YT10|D6YT10_WADCW Poly(A) polymerase OS=Waddlia chondrophila (strain ATCC VR-1470 / WSU 86-1044) OX=716544 GN=pcnB3 PE=3 SV=1

MTNVESGALKIIQTLRNAGYAAYYAGGWVRDFLLDHPSDDIDIATDAKPEKIVELFQKTI

EVGISFGVVVVLMDGHQYEVATFREDLGYEDGRRPTQCAYTNAEGDAKRRDFTINGMFYD

PLEKNVIDFVDGRKDLEKGVIRAIGDPYERFKEDRLRMIRAIRFTYRFGYALDPDTEQAI

KDHAHCLFPAVAMERVWHELQKMARYPSFGEALLKMHAVGLLAEVFPPLKKVPEIELLKR

VEAINRFPLECPAVLQVMELFPNSPLEEQVAFCYYLKVSNKEIKLVEYTHQVRTQVSDQD

ADWVPLYAHPQAETVLQVVCARKNGGQFQVHRERMDRLRVHINRIVNQSPVVSASMLLEE

GIVPGRQMGELLREAERLAISHDLHLPEEVLLLLKKQTVWSKE

>tr|D6YTX9|D6YTX9_WADCW Putative chaperone protein htpG OS=Waddlia chondrophila (strain ATCC VR-1470 / WSU 86-1044) OX=716544 GN=htpG PE=3 SV=1

MTKKTLTIHSENILPIIKKWLYSDKDIFARELVSNACDAIHKVKILRDQGEAKALDDEFR

IEIKIDKEKRTLSFIDTGIGMDAEEVEKYIAQIAFSSAEEFLEKYQSKDEGDQFIGHFGL

GFYSAYMVADQVEINTLSYKEGAEPVFWSSDGKADYTVEKGTRTTRGTEVILHVDKNSDE

FLEESRLKQILQHYCAFLPYPVYLGENHINTHEPLWIKSPSECTEKDYLEFYRYLYPMQE

DPLFWVHLNVDYPFNLKGILYFPKLRRELDLNKNTVHLYCNRVFVNDNCKDIIPNYLMVL

QGVIDSPDIPLNVSRSYLQMDRTVRQLSSHISKKVSDSLSSLWKSDREKYTRCWKDIAPI

VKLGVLEDDKFYDRIKNLLIWKIAGGAWTTVQEYLERNQEQTKDTVIYTKDEGQMPHFVE

MYNKKNVEILLAPDPIDSYVFQQLEQKLAPVKFRRIDAAVDDHLLDKGREKSVLDAEGKT

EAARLADFFRSKLADQKVEVEAKSLAHDETPGFVMISEEQRRMRDYMMALNQDQEMDDVH

QMALRTFVINTNSPLINRIKDLELKDPDLAKEMAQEVFELSLLSQRELAPKALNQFIHRS

TAIMAKLLEVQKN

>tr|D6YWI5|D6YWI5_WADCW 50S ribosomal protein L33 OS=Waddlia chondrophila (strain ATCC VR-1470 / WSU 86-1044) OX=716544 GN=rpmG PE=3 SV=1

MASKREKIKLKSSKSSHHYYTFKNKTSTPDRIVLKKYDPTIRQRVEYKETK

>tr|D6YWE4|D6YWE4_WADCW Putative amino acid ABC transporter ATP binding subunit OS=Waddlia chondrophila (strain ATCC VR-1470 / WSU 86-1044) OX=716544 GN=wcw_1098 PE=4 SV=1

MISIKNIRKTYDNGKTYIVDNISLEIPEGKTVILLGSSGCGKTTLLKMINRLIESSSGEI

IIDNQNIRDYDPIILKRTIGYAFQGVGLFPHLTVKENVTIVLKLMGMATKQIEQKALDLL

KTVNLEPKIFSSRFPDELSGGQQQRVGVARSLATHPKYLLMDEPFGALDAINRDAMQEEM

RVIRDKFHTTVLFVTHDIFEAIKLGDLIAVMNKGKIEQTGTPAELINHPQTSFVKNLFQK

PLEQLKLYKEELKT

>tr|D6YT82|D6YT82_WADCW RNA pseudouridylate synthase family protein OS=Waddlia chondrophila (strain ATCC VR-1470 / WSU 86-1044) OX=716544 GN=wcw_1944 PE=3 SV=1

MKKMIDQDQPLLEALRLLYPDSSKTTLRSLLKEERIKVDGQTCKLGTLSLKKGQILEVVK

KQNVIAEGVKILYQDPHLAVVFKPSGLLSVATDFEKNLTLHKILKEHFQPKTVEVVHRLD

QDTSGVIVFALDKQTCLKLKILFEKHEIERCYTAIVEGKLTPRSGSWRSYLHEDENYVVH

STDNARKGKLAITHYVVEGYSKKFTRLRLKLETGKKNQIRVHCSDAGHPIAGDTKYGASS

NPGKRLMLHADHLGFIHPVKRKKMQFTIDPPESFDKVVLKKRNP

>tr|D6YW07|D6YW07_WADCW Uncharacterized protein OS=Waddlia chondrophila (strain ATCC VR-1470 / WSU 86-1044) OX=716544 GN=wcw_0958 PE=4 SV=1

MKDVTQRKKVLKEMLKSKNFEQLDVFQDSSQWEKMQADERELLGLLYVMQGEKQLSKGDN

KVLDSFRRANLVAPNCPRIMYRQAVAFAGETHNLYCLKAAKKIFYRVISLQTGSFDSWYG

LANTCSLLAMHSNIEKNYLEAQKAFEKAEELAHFQPKEILAALYRDWGVLWFHYGKFSGE

AIDFRNGAKCYKKAVSMGMKTSDLWNDYGNCLTELASLLRKPDLILESINMYWRAVRLKE

DFFEGWMNLSAALKVVYEIQPIEAYYKLANEGFERASKLDPQNGPLWIKWGQMQVYHGKI

LKSIEALCDSIGKFEAASICEPDHPVVLCSWAESLLCLGEWTEDLQLLREAEEKIIKSLE

MQRDQPRIWCLYGDCLAEIGRYFDEEEYFFQAIERYRTGLRLSPQDPVILHGLAMTYLTL

AQATQDLNWLDKASDCCRQSIENGGHGQAQYWNDWGVVLMKLGTAADDRELVNSALNRFE

QALRICSQTQKQQEIEPEWLYNYGCALDFLGDYDDDLSMLEKAIHVLKKVIEIDPGFHHA

YYNLAISLAHFGDLEAEPEAFRESLKYLEAYVSFESEDEHVWNEWGLTLIDLSQLIDDPV

RKKQTDALLQEAEIKFLHARDLGSLTSFYHLACLHCLKKNAETAFYYLRKAKEAGALPPI

EDLVSNHWLEAIRHTPEFQRLILES

>tr|D6YVC8|D6YVC8_WADCW tRNA (guanine-N(7)-)-methyltransferase OS=Waddlia chondrophila (strain ATCC VR-1470 / WSU 86-1044) OX=716544 GN=trmB PE=3 SV=1

MKPKDLKFPFSWEARQVLLKDRVLYVPEYYDNYDQFTFPGWEAPSLFGNSKSVVIEYCSG

NGAWIVEKAKSNPQNNWVAVEKRFDRVRKIWSKIKNEHLDNLVVFCGEGFRMTQEYFPSG

AVDRIYINFPDPWPKKRHAKHRIVQRPFVEQISRILKPQGVLTLVTDDAVYSEQMIEVLN

MQEGMCSAHPEPYYVNELEGYGTSFFDALWRDKGRVIRYHQYRKQELMHAVD

>tr|D6YW93|D6YW93_WADCW Uncharacterized protein OS=Waddlia chondrophila (strain ATCC VR-1470 / WSU 86-1044) OX=716544 GN=wcw_1045 PE=4 SV=1

MLEPIRIFKFDIFSFFLALFSDRFLQSDRKNLSKRKKICTNSNIRIGSWKTK

>tr|D6YS08|D6YS08_WADCW Uncharacterized protein OS=Waddlia chondrophila (strain ATCC VR-1470 / WSU 86-1044) OX=716544 GN=wcw_1504 PE=4 SV=1

MSTSTIHRSNTAVEALGYTASHTDTLNKFAKTVTQGVALAKIVTSLVPGYEGVSSGLGLL

AGELKTASSVINATNILERGSEWGTKSTRDSILSRWQKTANRVALTVAQFFETILFIDKC

SLSFFYSAAMIVCHIPILEMVKNCLYMTSAVFGLWYVGQDLSKATSSMSSAKEKMRKWTA

FDVESKNQDLKLKYQKKLEGKGATSEKLSEEVQKLRAEIADNKKKLERLSGEYAKQLKAK

IKEDGNALTTNKVCLVSVLKYESYLQAINEGEVKKIKEYKIEKYETRIANCKKIREKSWL

SIAVDIGKIVMISLGMFVAAFSLTFPLLTLPTAALVITSMSLVSNALGLTKNIYSAVGPK

VQKEPVFG

>tr|D6YSD0|D6YSD0_WADCW Serine--tRNA ligase OS=Waddlia chondrophila (strain ATCC VR-1470 / WSU 86-1044) OX=716544 GN=serS PE=3 SV=1

MIDIKLIRANREEIEEKLKRKEPGISLFPILELDQKIRGLKTKVEQLKSERNHVSKKIGE

MKRKGEDSSAMMSEVSGKSAEIHAIDHELGPLEEKFRLELAKLPNLPMDDIKIHDDPAEN

VMIKEFGEKREFDFPFKNHVELNEQLKLFDFVRGAKIAGSGWPIYRGWGARLEWALLHYM

MSVNIKNGFTQWMPPALVRPEVMFGSGQLPKFANQQFAIHDEEYNLFLIPTAEVPLNGLH

ADEILEEEELPLKYCAYTPCFRREAGAAGSNERGLIRTHQFNKVEMFCFCKPEESGKIFD

EMIANAEEILEGLGLHYRNMLLVTGDMSFASARTVDIEVWLPGQDRYYEVSSVSNCTDYQ

SRRSQIRFRRKGEKPELLHTLNGSGLATSRLMVSLIENNQNSDGSINIPTVLQPYLDGKT

VLN

>tr|D6YS91|D6YS91_WADCW Putative membrane protein insertion efficiency factor OS=Waddlia chondrophila (strain ATCC VR-1470 / WSU 86-1044) OX=716544 GN=wcw_1590 PE=3 SV=1

MKHLLSFLITLYQWCIRPLLGQNCRFSPSCSEYGKEALIKHGAIKGLWLILKRLIKCHPR

HPGGCDPVP

>tr|D6YUU8|D6YUU8_WADCW Pyrazinamidase/nicotinamidase OS=Waddlia chondrophila (strain ATCC VR-1470 / WSU 86-1044) OX=716544 GN=pncA PE=4 SV=1

MKRAFLVVDVQNDFLPGGALEVKEGDKVIPLINELLKLPFELVVASKDWHPVNHGSFADT

HGKGVGDFIKLRELDQILWPVHCVQDSLGAEFSDRLDQSRFDKVFHKGTDEGIDSYSAFF

DNGHLKATGMGDYLKEEGITDLYIAGLTTDFCVKYSVLDAVNLGFRTHVVRDACKAVNLS

PDDEEKAVEEMKKAGAQVVASEQVIKEFDG

>tr|D6YUL7|D6YUL7_WADCW Putative rhs family protein remnant OS=Waddlia chondrophila (strain ATCC VR-1470 / WSU 86-1044) OX=716544 GN=rhs15 PE=4 SV=1

MAERRRSCLVLPGKGLNRAARWLNFAEKEVKAACKMEGMAAKISREIAPKIERPVKDVVS

NGGKGQIWSETKKRSSVTNAYKHWKDHGNEFPELNNAKEYVDRAHNFLRDPSALSRTRPN

GEILKYDSRSNTFGSYTKDGIPKTLFKPDPSKHRHSTNLEYFYGQ

>tr|D6YUJ8|D6YUJ8_WADCW 1,4-alpha-glucan branching enzyme OS=Waddlia chondrophila (strain ATCC VR-1470 / WSU 86-1044) OX=716544 GN=wcw_0437 PE=3 SV=1

MTVSAIEKIDSFSSFFNFDARKNEMDLQMKICVDLSSDTQAIAKSKELVKKVKLNKLQVL

ILKIINFVMKIFNAEFNKIDGLLTSNRCAKLLHRKYTEILNPEKMDYPAQLKHEQIETWV

QSGLTYLQQVQFQKLESQLAAFEEKTADATPEQLYKEYKSLPKFLRELLRKVVRERYAET

VKENPLLLLKKEENSTTAIEEIARIIHSQADAYSKLNALDAQEDQRDLQTVIAKNDLIFS

RRRNKEKVIYDVSTEGVLGDLSETLLEKPITVTMVGVEYAGLVKQGGLAEALEGLSRGIK

EQNEQNRVKLIFPKYSHLPKNIQEQLHDPVVHSGKNGTFNVYRLDINGVECYFIEDPSFV

LSEEKPDIYGPDMQTQATRFAKFSELAAELIWEQKDTDVIHLHDWHVSGVGLKLKQDHQE

EWQSGEIPPVLFTFHNNNRCAQGRILLGAYNYDPVVKGFQDSGITSKNDNLFVSTLMSAD

AMTTVSEMFGLESQQEKFGEGVSFAVRQAAKVGKLVGIVNGTNTDRWDPKTDPLLVKWKD

LKSGEPLNLSYGPDDEDILEKKGLAREQLQLWTEKYMPESKIDFSKPLVTFIGRFDSYQK

GVDKFEEAIEATLENGGQFIAMGMGEDPEAARILDKLEKKYKEGVLFVRDYKDPDGRIHF

QQGNAERPGMGSLLRAVSDFLYVPSRFEPCGLVQFEGWLFGSLAIGSNTGGLADTIISPE

KEENAFNGFLFEREGSVDNSAAAVVAKSLKFWGEQSDSSKRAIMSRLMKEGKKYGWHSSP

RGFTPAEKYRFSYENAKRRIGNRGRQENSVYRIDAVAREIVVDPAKKAETFPEESYMQQY

YQSGLDSAELYQTYSTVPTDWRIAVPSPYGKHVNHTRYNEYGAFYREDETTFRVYAPHAL

GVKVRLYDEQENLFCEAPMKKNSKGEWETIFGQIKQGQRYHYVVDGKIKIDPYSRSYTSF

SNKHQAPYSIVTHSTFEWNDKEWMTQRESDKGKPKPMSIFEFHPTTWKRKERKPLNYREL

ASELVKHCKKVGYTHVEPMGILEHFYEESWGYQVSGYFAPNSRMGSVDDFKFMVDHLHAN

GIGIIMDWVPAHFAKDDYGLMDFDGSNLYEASGLKYQLSIRKLAFSYGCKHFDYSKKSVR

EFLISSAAFWLKEMHIDGLRVDCVRSMLNSEDQQSANQFMRDLNAVVHEHCPGAVTIAEE

YSGDTSVTKPVWLDGLGFDMKWHVGWLKGALSFFKISPKNRSKRYEELKKAIQSDNFHKQ

VMALSHDDFHSKGLIALTPDLTEEEKLANLKAMLSFMMCLPGKKLLFMGSDSGNENSWLE

AFASHESFADPSREKEEILEMIARLNTLYREHKELWESDNNGHDLEWIEDPEKKVHAYRR

KSSSGDSCVCLHNFTDKERTFTIKCSANSIEAPLEIFNSDAVEFGGSGQINLAVDIVSGS

KGKSYKVTIPPLTTVMMKEVHHG

>tr|D6YWA9|D6YWA9_WADCW Uncharacterized protein OS=Waddlia chondrophila (strain ATCC VR-1470 / WSU 86-1044) OX=716544 GN=wcw_1061 PE=4 SV=1

MADKLKEMSDLMKETLHLMVKIQKTYPKDPMKCFDYVEKLVDNFEAIKKINQDDENFETD

EISNEFIESLEKFIEVTEKYRQDESSPYHNAMNKEKVLQILVDLDRRYNHLTE

>tr|D6YRU9|D6YRU9_WADCW Ribonuclease Z OS=Waddlia chondrophila (strain ATCC VR-1470 / WSU 86-1044) OX=716544 GN=rnz PE=3 SV=1

MSVRDITILGCSSQQPTRFRNQGAYLVRWNDEGLLFDPGEGTQRQFIFANVSPTCVSRIF

ISHFHGDHCLGLPSMLMRLNLDKITHTVHCYYPASGQKFFNRLRRGTIYHETIKIEEHPI

HESGIVVDDGHFKIEAAFLEHGIDNIGWRITEPDTRKFDDRQLQNFGISGKMVRELKEKG

AITINGARIALDDVSWIRKGDALAVVIDTRKCPQAIELAKGAKTLICESTYLDEHRKLAH

AHYHMTAKEAAEIAKEAGVQHLILTHFSARYLDSSAFEKEAGEVFPNVSAADDLKKFPFP

R

>tr|D6YVL3|D6YVL3_WADCW Putative disulfide bond isomerase OS=Waddlia chondrophila (strain ATCC VR-1470 / WSU 86-1044) OX=716544 GN=wcw_0807 PE=4 SV=1

MHHDTLRRSLFEMKGYLFLTIFLLAALPILESSEQKNGIAWHTNYEKALQESKATSKPIF

LFFTGSDWCGWCKKLENEVLNTQEFAEAIGNKMIFVVLDYPMKKNLDAKTREQNQTLKER

YSIKSYPTVVLINGNEELIGTTGYLSGGGQKYAQHLIKMVQEFTAYKQKMRRPSLREYSG

KELKRLYQKSQELGLAADSDKLVQAGIHSDLPHFFLTERYRILLKNGLLHSEEAAAIKEQ

LLVNDPNNKHLTHYQVAIAEFECLSKELESKKVNADAVVYPLVNYINQFKEKDIENTWKL

DMVISQVYLDENQLEKSLKFAQDACAHAPPAIQKEISQAVNKIQEQISEVR

>tr|D6YSU9|D6YSU9_WADCW Glycosyltransferase, family 2 OS=Waddlia chondrophila (strain ATCC VR-1470 / WSU 86-1044) OX=716544 GN=wcw_1805 PE=4 SV=1

MLFKLKKKQPERQRVSVIIISYNNESQVAKCLDALRQQTVSPHQMILVDTGSKDRSYLEK

YREECYVIEAEPHCGFCVGNNLGYLAVDPEADAVLFLNPDAFLFPDFLENGARYLSHHTD

VGAITGLTLGYDFEMKKPTGFVDTTGIFSTWYGKWYDRGQGSIYEPVNSPGHGDIPAICG

AVFLGRKKALDQTRINREVMDSRYFMYKEDIDLSLRLLKKKWKLRYVPELKAYHCRGWKK

NRKEMPRQYRLYSARNELRVNWGRKHLIGSLYSLTKYLTVKVFNC

>tr|D6YWY7|D6YWY7_WADCW Uncharacterized protein OS=Waddlia chondrophila (strain ATCC VR-1470 / WSU 86-1044) OX=716544 GN=wcw_1296 PE=4 SV=1

MASIGPRDLFSVGFSEPVKIRSSELDKYVSAFRELTNQVRSEIAETKGVSTDSVDENSIL

LSTMVSQKEGGVGRLVLENCSNMNNILSACQEKQAKIDQKTLARLDALLNAALDEKLQVI

YKEQEINVDTGQKQELMILSHFATGEAPQLKSLEVMFGVGNPESSTTVTSAQFSENYKDK

LKVLQGLWVNSPTQDVEEFGLKGEIEEPKIIGLKDNPEGSADDEGERDRLDAKENSVNRS

RIEDDAKKRLHEEEERKNRQNDAKDRMIERNQ

>tr|D6YT21|D6YT21_WADCW Glycogen phosphorylase OS=Waddlia chondrophila (strain ATCC VR-1470 / WSU 86-1044) OX=716544 GN=glgP PE=3 SV=1

MNQPSKARKIAYFSMEIALESSLPTYSGGLGVLAGDTIKSAANLEVPMVAICLLYRKGYF

FQRIDSEGRQTEEPVEWEIENFLHKRPETLTVTIEGEEVTINLWEYKAIGLKGFQIPVYF

LDSDHPSNSEWHRSLTDVLYGGDEKYRLCQEVILGIGGVRALRALGYDNIERFHMNEGHA

SLLTIELLREECKGKSINDASEEEISQVRQQCVFTTHTPVPAGHDQFSWELAQSVLGEMT

PTALKDLACCEDKLNMTYLALNLSHYINGVAKKHGEISNLMFAGHKIEAITNGVYAPFWA

SAPFQKLFDQYIVNWREDNFSFRSALQLPMENIWETHIQAKKLLLDYINQKENYSMDLQT

FTIGFARRSATYKRADLIFRDLERLKKIVKEKGPIQIIFSGKAHPKDAQGKILIQKVYEA

KKQLENELKIIYLPNYNVQIAKLLVAGVDLWLNNPRAPMEASGTSGMKAALNGVPSLSVL

DGWWIEGCLEEVTGWAIGDEQEEAKFNFSQEEKEKIDQSDAKSLYDKLENRILPLFYQQP

NQYRSIMRNCIALNGAFFTTQRMVQQYVVNAYFR

>tr|D6YSM3|D6YSM3_WADCW Na_Ca_ex domain-containing protein OS=Waddlia chondrophila (strain ATCC VR-1470 / WSU 86-1044) OX=716544 GN=wcw_1727 PE=4 SV=1

MRRQEDIAVGNVVGSNIFNILGIIGASSIAAPIHIENINWIDFSYMTALFIGLWVIIQKG

SCITRREGSLLFSSYIVYLCYLLYF

>tr|D6YWS6|D6YWS6_WADCW MiaB-like tRNA modifying enzyme OS=Waddlia chondrophila (strain ATCC VR-1470 / WSU 86-1044) OX=716544 GN=wcw_1230 PE=4 SV=1

MLYYLAMNTKKRQKFRVVTLGCRTNQYESQAFRDQLLQMGYEPAEGKEDADLCIVNTCTV

TESADSRSRYEIRQLAKKNPHAKIIVTGCSVERQPEKIEAIEGVAQLVKNADKEILLEKV

FPGEDLPEFSIRNFDAHTRAFVKVQDGCNSFCTYCIIPYVRGRSRSRTMGQVLEEVKDLI

ANGYKEIVLTGINIGDFDGGADEPRRLAELVKEVDAVPGLERLRVSSIDPDEVDDEMLET

IINGKKTCRSMHIVLQSGSNVVLKRMNRKYTRQMFMDTVSRLRSACSDFTFTTDVIVGFP

GETERDHQETLEVLQEVQFAKVHMFPYSPRPRTRAALYSDRVSADVIQRRKQEVLRLSEE

IAFNLRSSYVGRRMSVLTENYDDLHTEMISGHTDNFLPVMVPKAALRPNTMIEVECRENT

AAALIGKVVGIGS

>tr|D6YTP4|D6YTP4_WADCW Conserved putative membrane protein OS=Waddlia chondrophila (strain ATCC VR-1470 / WSU 86-1044) OX=716544 GN=wcw_0130 PE=4 SV=1

MHLVVLLYAFFASVFTISKVGLQYTQPLFLVGTRMVFAGVLLLFYLFIFHRRQFSFSKSA

LWGLLQLAVFNIYLTNVFEFWGLQYLTSFKTCFIYSLSPFFSALISYFSLNESLSPKKWL

GLAVGFIGFFPILLTESVAEEGVRHLFFVSWAELAVMLAAVTSVYGWIILRRLVKDEGVS

PMMANGTSMVIGGAMALANSYLIEDWAPFPVTDMVSFAECMILLVVVSNMVCYNLYGYLL

KRFTATFMSFAGFTTPLFTAIYGWFFLSEVISSAFYLSAAIVFIGLFLFYQEELKQGYTI

PEPV

>tr|D6YTU1|D6YTU1_WADCW Uncharacterized protein OS=Waddlia chondrophila (strain ATCC VR-1470 / WSU 86-1044) OX=716544 GN=wcw_0177 PE=4 SV=1

MKLNEYLLKEKFLEILNESITFEEGAFWASEMMAKNEIGELEYDSSDDISKLFSALTFLA

GLSTEISPRTYLYTIEDVKIEYDQLFSRS

>tr|D6YRN3|D6YRN3_WADCW Glutamine synthetase OS=Waddlia chondrophila (strain ATCC VR-1470 / WSU 86-1044) OX=716544 GN=glnA PE=3 SV=1

MQRFRAIEAVASHNHRIAERAYSTQRASDEFGIHVFTRSVMQKMLPREIYCNVIDAMEGK

DKIRDEYADPIAVAMKEWAISLGATHFTHWFQPLTGASAEKHDAFIEWESPDRMIEQFTG

KQLIQGEPDASSFPSGGLRSTYEARGYTGWDPTSPVFVWRGGDGVTLTIPSVFFSWTGDV

LDSKIPVLRSEKKLSDACLRLLKLTGIDAERVFTTIGLEQEYFVIDQSLRNLRADLALAG

RTVFGAPSPKGQELQDHYFGSVKERILCFMHDFEKAALRLGIPVKTRHNEVAPAQHEVAP

VVERASLAIDHNIQLMELMRKIAIRHNLSVLLHEKPFEELNGSGKHCNWSVATDTGINLL

DPTETPENSLHFLVLMTAILHAVFQHSALLRASIGSASNDRRLGGHEAPPAIISVYLGQA

LESLLENIEKQGVHKSASNRSKYDLGIKELPDLTKDNTDRNRTSPFAFTGNKFEFRAVGS

SQNPATAISVLNAIVATSLNEMLDSVEKKLGGKRPKNREELFEAVIPVVRKFLENSKSIR

FSGDNYSNEWVKEAKKRGLPNLEKSLDAFKSFTSKTTVRAFEGILTAQELKSRYEILAEN

YGLTVNIEANLMIDMFKTQVLPAALKQLNLFAESIHHVSRYVQKSSTKNLQKMLTDLNAL

VEKALVCQEKLIAAKEKAKKLSSVKQAEEFCRCVQPEMEAFRKVVDSIEGYVEDSQWPLP

KYRELLFFV

>tr|D6YSG9|D6YSG9_WADCW ABC-type transporter, permease subunit OS=Waddlia chondrophila (strain ATCC VR-1470 / WSU 86-1044) OX=716544 GN=wcw_1670 PE=3 SV=1

MHAINPYMGQNFLSFIAVFAMRMRLFFTGQLGIQELVSDEIQVFVLAGVAVSSSLLGCYL

VLRKMTMLANALSHTILLGIVVVYFFSVYMLPGSDGYAHLHLSIRALLAASLLMGIVTTF

LTEFLTKSAGLQEDASIGIVFTSLFALGIILVTLLTRNAHIGTEIVMGNADALRLQDLKL

VLAVLFLNIALIGLFYKEYAITTFDSGLSKTLGFSPVFLNYLLMTQVSATAIGGFRAVGV

LMVLALMTGPSLIAQLLTNHLRSMLLLSAAIGIFSAFVGVALSRHFLSVLGLAFSTGGVV

VCVIIALFLTVLLMKRPGL

>tr|D6YTQ9|D6YTQ9_WADCW Uncharacterized protein OS=Waddlia chondrophila (strain ATCC VR-1470 / WSU 86-1044) OX=716544 GN=wcw_0145 PE=4 SV=1

MSIDGYRSSDTLAKIQQSTAAKDVKRTETKEESGKTMVSYDVKLQGRSFKVTVGFDKTQL

EAHYKSQLDELPEGQKNVLTVVDKIVSGIKAEDLIAMSGTRISTSIADHADAQHTKVHLQ

GEKDPTCLSELDGKGKGELAKSITNVIQTFSKQEVRQLIQNAKESTSSLSPTHQVQIPTV

SFQESSDPKVEEEKDDPLIFSVSVQRENPESLPSDEQSPAKRTESKVQAASTAAFRLPDE

NKTSDKPLMTGSTEDPGKKIFQRWSDKQGFVADETIERIDTVEKLQKNASQEIGYLKQKL

SDCNPEEKTKIEARIDQLEKIRVKCQQKGASWANSDYFRDMLRGNSDLRSAENKEGIRGA

IQEFSKVMDEEYYAKAPPVNMRYHKCDVRGKTEKEATSTGWIRVGVVSCMDNGFVNQSSM

RELQNALKEGKEDLATEKRNEMTQGIFEVWKEQSKKGNSNIDASAGYALTQLGYSLEQVE

TIADIIKKGGDFSDIPGDVLKIGKENIAQVAEGVDAVMEKRNQLMANQFLQIVMEQLEHS

SPEDLAGNQLRMLHVGLLNHQSRSVDGTGWYHNEDQEMQDMADIFDQFDQAKLICDGKGP

YIDSDGNIHLPSMSQLPEGVQELSLRAIYLNQSVQGHTENDGTQRELNEKALKKLKGMGI

DDETMKGLEQKLTGKKSGYTSAADTVNLGLKARFKVSTGCLSAKDRTGFVSALVTKRKME

EKRFPKSTVRRVMRGQLGTSSPAVRVIKDNTGTRIMKITPFKIEGLTKDEYSPTNFAARL

IVYANQGIEIMKERKRIAKYEKLGKASAAA

>tr|D6YVS3|D6YVS3_WADCW Biotin carboxyl carrier protein of acetyl-CoA carboxylase OS=Waddlia chondrophila (strain ATCC VR-1470 / WSU 86-1044) OX=716544 GN=accB PE=4 SV=1

MELKQIKDLMAAMGRTRLKRLKIKNDNFELELEREEKVVKQVVEHMPEAYARAETEIPRV

KAPDIPASLHPPVDHDSAVREEKGTFITSPMVGTFYSASGPDEPFFVKVGDRVTEESVVC

IVEAMKVMNEVKAGVSGVISEVLVENGHPVEFGTKLFKVS

>tr|D6YUF5|D6YUF5_WADCW Proline--tRNA ligase OS=Waddlia chondrophila (strain ATCC VR-1470 / WSU 86-1044) OX=716544 GN=proS PE=3 SV=1

MTQRREKTAITPTRQEDYPEWYQQVIKAADMAENSPVRGCMVIKPWGFGIWENIKSTLDQ

MIKETGHENVYFPLFIPLSFLEKEAAHVEGFAKECAVVTHHRLEEKDGKLIPTGELEEPL

IVRPTSETIIGDSFSRWVESYRDLPLLINQWANVVRWEMRPRIFLRTSEFLWQEGHTVHA

TKQEAKEETLKMLEVYRVLAEEVLAIPVILGEKSAGERFPGAESTYTLEMMMQDRKALQG

GTSHYLGQNFARAQGIQFSGKEGELEYAYTTSWGVTTRLIGGMIMCHGDDDGLRIPPRVA

PKQIVIIPVIPKPELEEEVLAFAEKVKQQIISQMFYGKPISVHIDKRDRRGGEKNWEWVK

KGVPVRIEIGPRDIESDSVMLARRDRGHKEKEKVSLETLGAIIPNLLNEIQKNYFEQAKA

FREMHIQRGVETLEALREFFTPKNPDKPEIHGGFVLAKWSEDPESEKILDEMKLTIRCLP

LSQSGTKGKCILTGKDAVKDVIIAKSY

>tr|D6YWP6|D6YWP6_WADCW Uncharacterized protein OS=Waddlia chondrophila (strain ATCC VR-1470 / WSU 86-1044) OX=716544 GN=wcw_1200 PE=4 SV=1

MSFDVARKPIDNTENIDVIQKKSTIGESVSQAIAKVAKVALKAIVALGIALTLGLPLLSK

SVRLFTADLFFGPSLERELEKAKINPKKAVEKAISSLKDAQEAAKEEGKQAPKPEDLEDQ

KGNLKKTLLGIEGLEQNRQKIDEYHRQIADTEAVDFKKEKAEKDQFLSEYAPKFEEARLE

KIKLDNERKIAIGTEGKQGLVLPKITPDILEKAKAKKAEAEKAEAEKPTYMEKTTKKVAT

HVENNSGRYAIALGVGATLLMAVSRLK

>tr|D6YUL6|D6YUL6_WADCW Putative rhs family protein remnant OS=Waddlia chondrophila (strain ATCC VR-1470 / WSU 86-1044) OX=716544 GN=rhs13 PE=4 SV=1

MLEQGEIRQLRILGDPVEEIGSAVLFEIDGIKYVPQHDLRGNVVLLLSPAGKAEVYRYSA

FGEELFQETVSPWRFSSKRVDEETGWVYFGRRYYAPSWGRWTTADPAWFADGPNLYAYVH

NNPLRYVDPDGLSAIEHQQMNRPGTQGTFFGSFSRGILDDTSWGASSWMLGDYVCDNWQS

SLGYGMGTGVSMMTGLVYGGTEAKLLGVAGKGPEPGGALA

>tr|D6YVJ9|D6YVJ9_WADCW UDP-N-acetylenolpyruvoylglucosamine reductase OS=Waddlia chondrophila (strain ATCC VR-1470 / WSU 86-1044) OX=716544 GN=murB PE=3 SV=1

MNDSIRMEAGRSLRHLCTIGIGGPAKWYLEVRSIEEMQEAFKKASKLNLRTFILGKGSNT

LFDDKGFDGLVIHNKISFCNEISDGMFHVGAGFSFSRLGAQTARQGWSGLEFASGIPGTV

GGAVFMNAGANGSETEESLASVDYVTEKGELKHFDRHEITFGYRSSSFQSLHGAIVGATF

SLTPSCNAKSKQLSIIEYRTKTQPYGKKSAGCMFRNPPSQTAGALIEACGLKGSVQGEAQ

VSSLHGNFLINTNQASSADVLALVRNIQTQVKEKYGIELESEVRYIPYRDETISS

>tr|D6YRS0|D6YRS0_WADCW UDP-glucose 4-epimerase OS=Waddlia chondrophila (strain ATCC VR-1470 / WSU 86-1044) OX=716544 GN=galE PE=3 SV=1

MKQTILVVGGAGYIGSHVNKQLHEAGYQTVVLDNLSTGDRKSVTRGSFIKGDASSSKQLD

EIFKSQKIDAVMHFAAFTDIGESVANPYQYYRNNVCHTLNLLHAMERYKVDIFIFSSTAA

IFGLPQTNKIAESHPKNPINPYGKSKLMVEQMLSDAESAYGLRSCALRYFNAAGGDPEGE

IKNHKKKETNLIPILLRSLKSGDHSITIFGTDYPTPDGTCVRDYIHILDLGQAHIRAMEQ

LFNGASSSQYNLGNGQGFSVKEVISAVECVTKIPVKKISGERRLGDPPLLLADSRKAEQE

LGWNPRFPSLEEMIFHAWNSLN

>tr|D6YSJ7|D6YSJ7_WADCW ADP,ATP carrier protein OS=Waddlia chondrophila (strain ATCC VR-1470 / WSU 86-1044) OX=716544 GN=ntt4 PE=3 SV=1

MLCMMFLICFNASILRCIKDSVVVTASNAEVLPFIKVWAVLPMAVVLTLLFAKLNNRYSQ

ERVFYLMISGFLAFYALFAFVIYPNSDFFHPHATADAIQAALPRGFAGLVSMFRYWTFSL

FYVLAELWNSIVLTVLFWGFANEVTKVHEAKRFYAVLAIGSNIAAAFAGQAANSLSVGGV

YNESLPFGSNAFEQTLMISIFVIICTGIITMLIFRWMNKRVLDDPQFDDLHFTRFELKKK

KKMSVRESFSFLSNSRYLVCIAMMVVGYNLVINMVEVVWKNQLREVYSSPSDYYRFMNNL

TTAFGMVSTVTALFLPRIIGKFGWTSTALITPAIMLVTSLGFFGFLLFGDNLGGAWLAAL

GMSPAMLVVMFGALQNCLSKAAKYSVFDATKEMSFIPLDHEVKLKGKAAIDGVGSRFGKS

GGSLVHQGMLMVFGSLSTSAPYVAAILMAMIAFWIGAVRSLGRQFQELTHQGEPEKASDL

VKEEPVVA

>tr|D6YWJ9|D6YWJ9_WADCW Peptide deformylase OS=Waddlia chondrophila (strain ATCC VR-1470 / WSU 86-1044) OX=716544 GN=def PE=3 SV=1

MKLELAYYGDPFLRKKCKPVEEINNEIRELVENMVETLVEYNGIGLAAPQIKQDLRLFIT

AVPKELPNGDWEQGELIVFINPEIVSYSEETEDRQEGCLSIPKLYGNVNRPVRIVIKATD

MEGNVFERKFEGLQARCCLHENDHINGVLYIDRIRGKERKLLEPKLREIKKKFS

>tr|D6YS10|D6YS10_WADCW Uncharacterized protein OS=Waddlia chondrophila (strain ATCC VR-1470 / WSU 86-1044) OX=716544 GN=wcw_1506 PE=4 SV=1

MAPTSFPSYVIGKVSLPDVIRPAIKLTERVFQLTLKHFGLMQSTSVFGFGLQEIGLEYLS

ANRNTDAFSESRQQQIRNIKKIQAKQSLVHGLCYLGSGVLCTLGEMERVSWLNLDGTGNA

FIQSGWTLFLFANLFSLDQNIKLYHASSRFEGTFGHRLKMSAILGMINNLGYVVGTIFSF

FEGTAAIAIFLAVLSMMTGTVKFFYDYFFNLK

>tr|D6YV88|D6YV88_WADCW Putative permease, YjgP/YjgQ family OS=Waddlia chondrophila (strain ATCC VR-1470 / WSU 86-1044) OX=716544 GN=wcw_0682 PE=4 SV=1

MFPIIWRYLLSQYFKVMGLCVAAFIAVLLTSRLDEIAHFAVLSPTPALAAQFILLQIPYI

LPIVIPVSCLISSILLVQRLSKSHELTAFRSCGISLKDFLTPILFSSVLVAFINFYIISE

LSTYSHYQTIIWKEELRSTNPLHLLRNKHLMGAKGGFFNSLGDSKLGKNAGEVILALPNK

NQSRITLFLAKELISDDNFFEGTDVTMITAMPSGDPDSFDHLLVENAAKTKTLTDDFSQL

MKQKQWKIGNDYLTMEQLLAKLYEEQANGFSVNDINCCYSEIIRRFSLGFSAFSFTLMGL

AFGISISRTHSLKKILWVVLLAGGYITTLFIGKSAGQNLSLSLALYTIPHLLIISLSAAV

LYRVNHGIEGA

>tr|D6YVS5|D6YVS5_WADCW Uncharacterized protein OS=Waddlia chondrophila (strain ATCC VR-1470 / WSU 86-1044) OX=716544 GN=wcw_0870 PE=4 SV=1

MKFEIVTKWMLGFALMSAPLMASDEEPEEHIQVGSRALGHAERVLIQEQKYELIRKLENG

DELTEEESECVLAEEEAEDGEVKSKLIKKSAGTSTTYYTSHEGATHHPIAVSLMGDTVQL

EDGSIWIVSSEDRYQTFDWMTGDTIVIVPNHTWFSSYNYCLVNLNTGAKVKVNLSLGPIY

NGIYTHWILAIDYSNREVYLEDGSVWKMSWWDSSIVNQWLPNDTVIIGINDGWFSGSNPN

MLINVNMNDHAIGNCIF

>tr|D6YVS9|D6YVS9_WADCW Conserved putative membrane protein OS=Waddlia chondrophila (strain ATCC VR-1470 / WSU 86-1044) OX=716544 GN=yqfU PE=4 SV=1

MIPSMKAQTSPPKSITSQLGGYLWIILGAFLAAVSVEVFLIPKNLIDGGITGISMIFAYV

FGNNLLPFFLIAFTSPFLYMGYRYIGKVFLQHMIAAVLAFVGWIFFIHQYTDWVFIGESL

EVVVIGGAILGIGVGLIIRYGGCIDGTEILGIIINRSTGITVGQVVLACNVFIFTAAGLV

FKDWHPPILSLITFMVATKVMDAVIIGMEETKSVWIISPKSKIIASRIIHELGLGVTILY

GRGGYSGEEKEILYVTAERLQLAELKELTYSEDPTAFVAIENLHEVSNGVQRERKVSSQK

KAEKMMAKIFRN

>tr|D6YRL7|D6YRL7_WADCW Uncharacterized protein OS=Waddlia chondrophila (strain ATCC VR-1470 / WSU 86-1044) OX=716544 GN=wcw_1361 PE=4 SV=1

MKLDDFYQFIFHPWSSNHSLKKQISATIVDIALTIFSGLLFLIPFAYFQWKDRHVKVVYS

STATSKSAEKILKSSKEPSQKLSPKAQKVKNKQYWQLKQFEKWAAEGQWNKIHQAHYDWW

MYPISRSSQGQGTTYAVNSKEIAELKADQEFMQNYLRGVELGAKAWGWDIHLKKPVDHPS

KDQKWQNWDVRLGKMADSLHLFGQHELRDSMRTYALNKNLTLEEWVWKTLEPAIEP

>tr|D6YWC7|D6YWC7_WADCW Pyrophosphate--fructose 6-phosphate 1-phosphotransferase OS=Waddlia chondrophila (strain ATCC VR-1470 / WSU 86-1044) OX=716544 GN=pfkA PE=3 SV=1

MKEHTALEEKRLEYIPKLPAILHDLRKLKTVNLKNSPGKASEISDFFPLTIGQTALTFTI

DQDHEKTPLKVGVVLSGGQAAGGHNVITGLFDALKELHSKSQLFGFLNGPSGIVNNQTIE

LTEEILHSYRNQGGFDLIGAGRTKIETNEQFQGTLHTVKALDLDGIVIIGGDDSNTNAAL

LAEFFMKEGVRTRVIGVPKTIDGDLKNAYIDLSFGFDTAVKTYSGIIGNIARDSLSAKKY

YFFIKLMGRSASHIALECALQTHANYTLIGEEINEEKATFQQITNRLSDVICRRAELSKH

YGVILIPEGLIEFIPEFRTLIAELNEIKIDSELSKDERIQLAMRSISSESLECYKSLPRL

IQEQLMLDRDPHGNVQVSKIETERLFIEAVKQELKRRKEKGEYTGSFNAQPHFCGYEGRS

CLPSNFDSQYCYALGHVAALLIDANATGYMSCVKNLSRPIEEWQICGIPLTSMIHKEMRK

GKLKPVIAKALVDLQGAPFQYFKEKRLQWEDEDDYRYPGPVQFFGPSEITDAVTLTLELS

QNTEAILN

>tr|D6YTR0|D6YTR0_WADCW Putative ribosomal RNA small subunit methyltransferase B OS=Waddlia chondrophila (strain ATCC VR-1470 / WSU 86-1044) OX=716544 GN=rsmB1 PE=3 SV=1

MDITKTKNPREAAYLALLSSAREESFLIDSLAQWQAKCRPSIQDYHLAREVAYGTTRMAL

ALDHLALQLADKKKLALKLREKILFRMGLYQFHYMDKIPLYAIVDETIKIAKIHCHESFV

KFLNACLRKVEEIDPQLPKGETVPELSIRYSYPAYYVQELVQNFGLEKAEEIMEAGNRPS

QTMFRVRPQASRPKEANGEIDFLAGTRCGMGVIRDPSLISNISSSSDYYIQNATPAELMS

SLAQDFGAPKNILDLCSSPGGKLILAHDLYPDAELSANDVSSGKLKPLSENCAKYGISTT

LSSVRGEEFPLAQKFDLVILDVPCSNTGVLNKRPEARWRLSQKTLEQLEEIQLQLLRRGI

ELLDKGGELWYLTCSVLKRENGRLIDKVCQELSLETRLKEAILPNADGWDGGFGCALRKI

G

>tr|D6YRZ6|D6YRZ6_WADCW 50S ribosomal protein L14 OS=Waddlia chondrophila (strain ATCC VR-1470 / WSU 86-1044) OX=716544 GN=rplN PE=3 SV=1

MIQQESELLVADNTGAKRVKCFKVLGGSKRRYANVGDIIVASVQEAEPNGSIKKGEVVKA

VIVRTKYYIKRPDGTKLKFDTNACVLIDDKKNPKGSRVFGPVARELRDKNYLKIVSLAPE

VI

>tr|D6YTY0|D6YTY0_WADCW Na(+)-translocating NADH-quinone reductase subunit F OS=Waddlia chondrophila (strain ATCC VR-1470 / WSU 86-1044) OX=716544 GN=nqrF PE=3 SV=1

MTLFGIDLLLCLYAMIAFVAIGVALAGMILFTKAKFVSTDLCKIYINNDDGLTKSVPGGG

TLLQSLMSQGITIPSPCGGKATCKQCRVQIVEGVDPPLETDKSTFSRKELKEGWRLSCQS

KLKHDISVHVEESCLDVKEIDAKVVSNENVATFIKELVVEVPEEIPYRSGEYFQFHVPPF

KTNTSDWKETMEKKYFEDWEKFKMFDTEIDFSTLGEGEVIRAYSMASYPAEGKKVMFNIR

IATPPFEGKELSKTIPWGICSSYTFGLKPGDTVRLSGPFGESYMIDDNRELVFLIGGAGS

SFGRSHIMHLFKTENTKRKVTLWYGARSLKENIYEDEYRQLDKDHENFTYHLVLSDPQPD

DIKSGWPKDDPIKTAFLFKAFEEGQLKKMDYPEECLYYVCGPPLHNSSVMRLLDDYGVPR

ESIILDDFGS

>tr|D6YV24|D6YV24_WADCW Translational regulator CsrA OS=Waddlia chondrophila (strain ATCC VR-1470 / WSU 86-1044) OX=716544 GN=csrA PE=3 SV=1

MLVLTRKSEEKVTIGKDKKVVITILKIQGDKVSIGIEADKETPIYRNELINEENEQISRV

ELEAS

>tr|D6YRJ7|D6YRJ7_WADCW Uncharacterized protein OS=Waddlia chondrophila (strain ATCC VR-1470 / WSU 86-1044) OX=716544 GN=wcw_1340 PE=4 SV=1

MESFCFVSTIIEKSHLFRHNTKIFLMQIFFQTHAL

>tr|D6YTI7|D6YTI7_WADCW Putative methyltransferase, FkbM family OS=Waddlia chondrophila (strain ATCC VR-1470 / WSU 86-1044) OX=716544 GN=wcw_0072 PE=4 SV=1

MSCKYHLTENLAIFCRSSIEKFRCETFWTKEPETLEWIGRFNSKAIFIDVGANIGLYSLF

AASIHSKMKIYAIEPLSGNYQSIKENIQLNGFDQVIPLKIAVSDREGDVPFHILNEESGS

SGSQIIEPVKESGESFKPVKEEMIHCVTVDNLCERFNIRCDYLKIDIDGREWQVLKGAEK

SLESSVQSVLVELNPFYVALDSVHAWMKDRGFSIDGILQSLANHSNNRRSPQGPLNFIYT

KTDS

>tr|D6YVF3|D6YVF3_WADCW Uncharacterized protein OS=Waddlia chondrophila (strain ATCC VR-1470 / WSU 86-1044) OX=716544 GN=wcw_0747 PE=4 SV=1

MTHMQMDVKYLTDIPNYWEQLKPLGLPKSV

>tr|D6YW68|D6YW68_WADCW IstB_IS21 domain-containing protein OS=Waddlia chondrophila (strain ATCC VR-1470 / WSU 86-1044) OX=716544 GN=wcw_1020 PE=4 SV=1

MQFPWGEKGSTIVTSNLNFSQWDKSLSGDQALTSALLDRLLHHSHIIPIKGDSYDTLGDR

RRCHSRAI

>tr|D6YSW5|D6YSW5_WADCW Putative glycosyl transferase OS=Waddlia chondrophila (strain ATCC VR-1470 / WSU 86-1044) OX=716544 GN=wcw_1821 PE=4 SV=1

MKKLVMLTSFFPFAHGEEFLETEIEYLTKYFDQIHIFPKNFDKNRRPLPPKVQISHLFFS

NKILKKFTILRKSLFSSMFWIEFFNHPFNYIKWKPLKHLIFMTGESKKNLNKFSSYIQKN

GLEEALFYSYWCDHSPLILMLLKKKHPKITIISRAHGGDLYHERMPAGTKFLRQRLLSNL

DYVFTISNHGRDYLLSNYTLKSSKIIVSRLGVNPSKKKTSPSSTEEFFSVVSCSFLTPVK

RINLLIKALAIVAKRLPEVKFNWSHLGDGPLRNELEQHAKESFPSNVSFCFYGYIKNKKI

TEFYYKNSIDLFINLSVSEGLPVSMMEAQSCGLPILATNVGGVSEIVNSSNGYLLPKEIT

PEVIADKMISIKSNTKELNQKKISAYHHWEQHFNALKNYESFAQKISRLRSQS

>tr|D6YWK2|D6YWK2_WADCW Exodeoxyribonuclease 7 large subunit OS=Waddlia chondrophila (strain ATCC VR-1470 / WSU 86-1044) OX=716544 GN=xseA PE=3 SV=1

MTSKLSQDLPILTVSQLTNAIKHQLESTFPSIWLQGEVSNFKKHSSGHLYFSLKDGQAQI

SAVMFRGHVQSLKMIPKDGDQVVVYGGINVFAPSGKYQINVQTLRLAGVGELLLKLEELK

KELHQRGWFSQEHKKPLPKFPKKIGIVTSPTGAAIRDMLNVLNRRHGGYHILLNPVKVQG

EGAASEIARAIEQFNEHKMVDVIIVGRGGGSIEDLWAFNEEIVAKAIFESSIPIIGAVGH

ETDHCIAEYVADVRAPTPSAAAEIVGGEKAQQIEFLIQAQKQLTQNLRRQLRQWSTRLQQ

ILKTPLMSSPYALIGPWLQSMDDARLNADRAIKHVIRNQKVVLKSRFQMLQSLKPTAKIL

HFRQRLESLTKQCDLNISGLLKQREERLKRVASALQSIDPKNLLKKGYSILFSKKTGSVI

TTIKAVNKGDTVKVLLSDGEALAHISHSISSNQSKRL

>tr|D6YRM8|D6YRM8_WADCW Uncharacterized protein OS=Waddlia chondrophila (strain ATCC VR-1470 / WSU 86-1044) OX=716544 GN=wcw_1372 PE=4 SV=1

MIGSESNAAISIHPPPSSDPVSFIEHMIEKAKYVQEGSLHAKELIRDFLELQKHLSMEAF

VRFTKSWSRFTAYHLQERVARALSNS

>tr|D6YVW9|D6YVW9_WADCW Putative lipopolysaccharide heptosyltransferase I OS=Waddlia chondrophila (strain ATCC VR-1470 / WSU 86-1044) OX=716544 GN=waaC PE=4 SV=1

MRALIVKTSSLGDIIHAFPVAAYLQDRCPGILIDWVVEQPFSELVRSHPYVHSVYTVHTK

KWRKGRGWKEIAAIRKQLKSQSWDVVFDLQGNSKSAILTHWANSSDKVGYGDRLVFEKPN

LWVTTHQYDPPPEANVREENLFLVRSYLQDKAPFQSKAVYLNISTEEEELVDQIDRRLPN

QRKVMVCPGSAWLSKQVEQKALVCFLKQIPKTHFLLIWGSDSEREAVDFLHREIPNSSVM

DRFSIPVLQNLMGRVDEVIAMDSLPLHLAATIDVPTYSVFGASSAQKYKPFGKKHRAFQG

VCPYGRTFSRRCPILRTCETGACIRSLTGDELFEHYSSSKI

>tr|D6YUX4|D6YUX4_WADCW Putative Zn-dependent oxidoreductase OS=Waddlia chondrophila (strain ATCC VR-1470 / WSU 86-1044) OX=716544 GN=wcw_0565 PE=4 SV=1

MKVIQIRELKRTVEEAIHSLEVVEKPIPDPKPGQVLVKMAAAPCNPSDLLFLSGKYGVKK

SYPAVPGWEGAGIVVKSGGGALGWWLKGKRVACGGQSKLDGTWAEYYIADAKACVPLRDE

VSFEQGATLLINPLTAVGMMEEVLKGKHKAVVQNASLSQVGRLLRKLAEIEGIPLIDIVR

RSEHERQLRQEGARHVVNSSEENFRDQLKKLCDELSATIAFDAVAGEMTGDLANAMPEKS

FVFVYGALSGKPSAGVTPYSLIFQSKCVRGFWLSKWIKEKGVLRTLLAIRKVQQLMGSGG

FHTTIREVVGPEKWSKALLEYSRSMSGGKILLSFQNV

>tr|D6YWB9|D6YWB9_WADCW Transposase remnant OS=Waddlia chondrophila (strain ATCC VR-1470 / WSU 86-1044) OX=716544 GN=wcw_1071 PE=4 SV=1

MRSEIVDYFCDETGLKGQVYYREDIAKKKPAVLVAHAWKGRDQFALDKAKMLAEMGYVGF

AADLYGEGKVVESNEEAFELMLPLFLDRKLLRNRITAAYRAVSEHDQVDPQKIGAIGFCF

GGATVLELIRSGVDLQGVVSFHGLLGMTLGEQKAEMAPTAEKLFGSLLILHGYQDPMVSR

EDVVNIQKEFSAKGIDWQMHTYGNASHAFTNPDANEPESGLIYDSIAEKRAMQSMKNFFS

EVFV

>tr|D6YV60|D6YV60_WADCW DNA-3-methyladenine glycosylase I OS=Waddlia chondrophila (strain ATCC VR-1470 / WSU 86-1044) OX=716544 GN=tag PE=4 SV=1

MKRCFGNGPDKEFYAEYHDKEWGIPVHDDTRLFEMLILEGAQAGLSWETILKRRKGYREA

FHNFDPVKVAAMNDQELDQLKSDQRIIRNRLKISAARKNACVFLEIQKEFGSFDRYLWKF

VNNKPIVNKWKSMQDVPTTTPISDAISKDLKKRGMSFVGSTIIYAYMQAVGLVNDHLADC

IHWNH

>tr|D6YS03|D6YS03_WADCW 50S ribosomal protein L2 OS=Waddlia chondrophila (strain ATCC VR-1470 / WSU 86-1044) OX=716544 GN=rplB PE=3 SV=1

MLKKYRPRTPGTRQLILPKREQLTRPNKNNRKVVEPTKSLLLPKKRTNGRNNLGRITCRH

KGGGHKRKYRIVDFKRDKWNIPAKVASIEYDPNRSAYIALLNYVDGEKRYIIAPQGLKEG

AVVMTSDKPPYSVGCCMKLKDMPLGSVVHCIEMIPGRGAKLVRSAGMSAQLMARSGGYAT

LRMPSGEVRMVNESCKASFGSVSNPEHSLRVEGKAGRNRWKGIRPTVRGTAMNPVDHPHG

GAEGRNKGNIPQTPWAMYTKGYRTRSISKSRKLIVKDRRKK

>tr|D6YTZ8|D6YTZ8_WADCW Alanine--tRNA ligase OS=Waddlia chondrophila (strain ATCC VR-1470 / WSU 86-1044) OX=716544 GN=alaS PE=3 SV=1

MNTQEIRRKFINYFRENGHTHVPSSPILPHDDPTLLFINAGMNQFKDIFLGKSVRDYKRA

VTSQKCIRVGGKHNDLENVGHTLRHLTFFEMLGNFSFGDYFKKEAIRFAWEVSTTVFELD

PERIWPTVFREDDEAYEIWKEYVPEEKITRFDEKQNFWSMGDTGPCGPCSELLYDRGPKY

GNAKTPLEDEKEERYMEFWNLVFMQYNRDEEGVMHPLPKPSIDTGSGLERVVSIKMDVES

VYDTDVLRALITRVEEISGIGYDGADKEKAPAFRVIADHIRCLAFAIADGVQPSNVDRGY

VLRKVLRRAVRYGRMLGLDRPFLADLVPLLIELMGSDYPELKQSENRIGEILTTEEENFI

RTLRRGGNILNQIIDEAKKEHRQITGEEAFKLKDTYGFPLEEILLIALDDELEVDTKRFI

ELEEEAKERSRKTRKSTQQLASENLFEGFASCEFLGYEETEAEGKVAAIIVDNAFVDQLE

AGQEGMIVLDRTPFYAEKGGQVGDKGLLTGENSLFHVSDCQSPYKGIIAHLGKLERGAIR

LKDPLMARVDLERRQSIANHHTATHLLHWALCKVLGDHVKQAGSVVAPDRLRFDFSHHKG

LTQKEIRDIEDLVNAKIRENIAVKSYELSYEEAIQREEIKQFFGDKYDNIVRVIDIDYSK

ELCGGTHVKQLGSIGLFRIAKEGSIAAGVRRIEAVTGSWAEQRSREEENLLLSIADSLKT

QPQKLIQRINSLLEENKELAQELKTLKSAHLKNIVADLINKAEEVQGIRFIAQEVALSSD

EMRQLADDAVNALESGVVVLAASLDGRCQLMARVSDDMIDKGIKASDLIKTLAPIIGGGG

GGKAHFAQAGGKTPEKISQALSTVRTLLSS

>tr|D6YSQ3|D6YSQ3_WADCW Uncharacterized protein OS=Waddlia chondrophila (strain ATCC VR-1470 / WSU 86-1044) OX=716544 GN=wcw_1757 PE=4 SV=1

MPKVVDSLIEGIRERALGMVFQPTSSGVSGFTFICRIPNRQLSLHLDSSDSFSFISKWVS

KGYAKSEQVFSKIFRQCKVSAPEIRLDEECLEEKDLLQKFHRLAQSKKPAMGGAEMYDLM

FMNCFDAMTLDSFIREGHLKRLSEKDQETFFQKIGQGVILDLMIANDDRILSYDYESQDF

LNTLSPKFNFGNIMVEYPFMDNEQSRFLKEVHFIDNNSSVYLMDKKIIDHSDIVEIGSLF

GVEEGDNGSGALSSGIEKATDSEKDEFSPDDHSEKFSEAFKHLTANREVLASYVCQIFLN

EEQELSSNKVLIKSALCQGMEEIIRIIETQEIPDDEETSSPYLSRSMELIRRNIQFLKEI

QYVNER

>tr|D6YU70|D6YU70_WADCW Elongation factor G OS=Waddlia chondrophila (strain ATCC VR-1470 / WSU 86-1044) OX=716544 GN=fusA PE=3 SV=1

MPRSEQDKLDKVRNIGIMAHIDAGKTTVTERILFFTGRTHRIGETHDGTATMDWMEQEQE

RGITITSAATTVHWNGHPIHVIDTPGHVDFTVEVERSLRVLDGAVAVFDAVSGVEPQSET

VWRQAEKYQVPRIAFINKMDRMGADFFDAIESMKEKLHANAVPVQIPIGAEADYKGLIDL

ITMKAIVFKDETQGIEWDEIDIPAEYLEKANELRAQLLDELAVIDEENETFMAKVLDDPD

SLTPDEIHQVIRKGVCENLFNPVLCGSALKNKGVQQLLNAIIKWMPSPIDRGAVKAHLPD

SEDEILLEPSDDAPLGALAFKVMTDPYVGRLTFVRIYSGTLTKGMNLLNSTKDKKERISR

LIEMHANTREERDEFFTGDIAACIGLKNTTTGDSLCSPDKPYILEKMEFPEPVISMAIEP

KSKADREKLSQALSALSEEDPTFRVSTDEETGQTIIAGMGELHLDVLHDRMKREFNVEAN

VGKPQVSYRETITIPGGTDTKFVKQTGGRGQYAHVVLEIEPNEKGKGNEVESKIVGGVIP

REYIPAVIKGVEEGLLTGVLAGYNLVDVKVRIVFGSYHEVDSNEMAFKICGSMAVKEAAR

KCKPILLEPIMKVQVTTPETSMGDVIGDLNRRRGQIMGQETVKGGAIVNAEVPLSEMFGY

STQLRSLTSGRANYSMEPSHFAPVPTKIAEEIMKK

>tr|D6YTW1|D6YTW1_WADCW Thioesterase family protein OS=Waddlia chondrophila (strain ATCC VR-1470 / WSU 86-1044) OX=716544 GN=wcw_0197 PE=4 SV=1

MMSIWKSPITIESISAIREKTMTEHVGIEFTEVGENFLKGKMPVDDRTKQPFGIMHGGAS

CVLAETLGSVAACHCVEIDKQVCVGLSLNINHIRMARSGWVYGIARPVHIGGKTQVWDIA

IENEEGDLVSSTRLTLAVLDRR

>tr|D6YU24|D6YU24_WADCW Uncharacterized protein OS=Waddlia chondrophila (strain ATCC VR-1470 / WSU 86-1044) OX=716544 GN=wcw_0260 PE=4 SV=1

MGIPEVKNSDYSSQFPIISEENTEAKTVSSVIPSDIVERLSTEASSTRSLDQIDVQPEPL

KQEVYQAKIREKKLVFQAMQDKSKKVGTKSLNNQVEITSKNLKSMAILRGMIHQKMAREN

VDQSVIGNINEALKNESSFKGGAKKLATLLSDNGLDEEAAKKIAKEVIAEIAPSNGKPEK

HARELAKHTDKIARLLSKENPTEKELKSINKELIFTLRKAFECPAYQALKECEDNPAIHY

LHEINLAMYTEVHLASVYSEGKLAEEIVKDEKGNDRFSVGEGQRMGDAVHDIRNEAVKKR

KLFSSHSCLAYVRKHFKQFFGAFTSQKFGSIFGKYDPHGDLENNAGALYREELTVNGKKA

TALDVRTPSPTIGDKVSPEFRAALQAIENQRVFELKEGKDRGTQLKVGGRPTCWIYTNYQ

EMTNTSNGEAGRSKAIMQLNKEFPLSFRGLTLSKDSRYFKDGIGHGGSKMWDKVESEGKP

LGKEDVEGFVEELKEKLSDDSHFTLEKRTQSKHHGHGIYYPAEKEEMEEVLSAIAKEAGK

LLKEIIPEGEVLKGKEAWFAKAAAKEFAYAAIQRHFMSKELAALEEQGVSMNVITTSACK

EDIDRGFSDHIKRMWMLRAGKEKLLAKITNMPALMARYRVILEDRMQPLVGVMNLVSREK

AKEHLNNLSELNGVASEKAKVLGVEES

>tr|D6YW83|D6YW83_WADCW Uncharacterized protein OS=Waddlia chondrophila (strain ATCC VR-1470 / WSU 86-1044) OX=716544 GN=wcw_1035 PE=4 SV=1

MRRSICYCEPNQALAGEIYTWQFIYTTASPLPKGTLLRFDLMSDGRDIDWELPSVNIKDG

ANVIYAMLENGKTLNAKEVEVPDAWAPLYEFELPAKVNSGESFTIVIGSSKISEKLNINN

GTRAQTYSQRRRPFLLYIDTTGKRHFEEPEVFSIDIKGNILTQIKVLTPSFVQRNKRFDV

VVRFEDDYGNLTSNAPEETLIELTHEHLRENLNWKLFVPETGFITIPNLYFNDEGIYTIQ

LKNSYTGQVYRSSPIKCFSENGKSLYWGLLHGESIRVDSTENIDSCLRHFRDEKSMHFYS

VSPFEEVEETPNETWKLISQNVTEFDEQERFTTFLGFQWQGAAKSEGVRHFIWSKDNKPV

LRKKDAKYSTLKKIYKSFTPKDFISIPTFTMGKGFEYDFKNFDPEFERVVEIYNSWGSSE

CTKKQGNLKPIKGPVKKGVQESAEGSIVDALKSNKRFGFVAGGLDDRGIYADFFDGDQEQ

YSPGMTAIISKEHSRSSLYESLYHRSCYATTGERIILGIYIAGLPMGSEINTSDKPGLVL

NRHIAGYVAGTDKIKSIEVIRNGDVLQTFNPDDYHYEFEYDDMANLKEVCIDSKDKESPF

VFYYLRVVQEDGHMAWSSPIWVDFTDDIGAKKKAK

>tr|D6YS11|D6YS11_WADCW Methionyl-tRNA formyltransferase OS=Waddlia chondrophila (strain ATCC VR-1470 / WSU 86-1044) OX=716544 GN=fmt PE=3 SV=1

MKVIFFGTPHFSASVLRYLLEHGIKIVSVVTRPDKPKGRSNKLISTPVKEVAIEHGLPVY

QPEKASSSEFANVLPPYEADLFVVVAYGEIVKEHILGMPRLGCINLHTSLLPKYRGAAPI

QRAIMNGEKETGVSIMYMVKKMDAGDIIQTQSLVIDENETFGELEERLCQKGAEMLLQTI

RKFENGPVEGMPQNDDEATFAPKVELEDCEIDWTMPARRIHDLVRGSQPYPGAWCWVDVK

GEKKRMKLIKSVLGASVNSVPRKIIAVNEEGVEIGCGEGSVKIQKLQLEGKKAMAPVELF

RGTEVVF

>tr|D6YW47|D6YW47_WADCW 2-oxoglutarate dehydrogenase complex component E2 OS=Waddlia chondrophila (strain ATCC VR-1470 / WSU 86-1044) OX=716544 GN=sucB PE=3 SV=1

MKEEIKVPAMGESITEATVGQILKPSGSHVKMDEEILELETDKVNQVLYASQTGVLTLTV

ETDDVVKIDQVIGLIDSDGGKPEKKEEKASAPVLKKEEKKPEKGIRHSREAFVAEIGKQE

KSAPPPTMKKERGETRRRMTKIRKVIAKRLVEAQAATAMLTTFNEADLSQVMKLRTKYKE

AFIKEHDAKLGFMSFFVKAVVSALETFPDINSYIDGDEIVHRDYYDIGIAVGTERGLIVP

VLRDCDQKNFADIEKGIIEFAEKARAGTISVDDLQGGGFTITNGGIYGSMLSTPILNHPQ

VGILGMHNIQKRAVVVNDEIVIRPMMYLALSYDHRIVDGKEAVSFLVHVKNCLEDPSRLL

LGV

>tr|D6YVN4|D6YVN4_WADCW Succinylarginine dihydrolase OS=Waddlia chondrophila (strain ATCC VR-1470 / WSU 86-1044) OX=716544 GN=astB PE=3 SV=1

MNAPAYEVNIDGLVGPTHSYGGLSKGNLASIENAGNISNPKIAALQGLNKMKQMADYGYR

QLILPPHERPHLPTLRALGYTGVDNRIPGKVYQDNPELLYQYSSAASMFAANAATATPSI

DAADNRLHLTPANKAATPHRIIEAETTLRLLRTIFPNPTFFTIHPPLPFHPLFHDEGAAN

HIRFCTDLRYVGVHLFVYGKANEMDDLPEERIYTPRQTLEAQKAIARSHRLDPSQVVYAM

QSTEALNQGVFHNDLISMGCHDLFIYHELAFENPEAVLDELKNTFNEICDQPLKTIKVAN

NEIHLKAAIKTYFFNSQIIKLQDGGFVLFCPKQCQNHQDVNKYLTNLLKDPKSPIADIHY

IDLDQSMRNGGGPACLRFSTVLTDIELEQVNPNLFLTDKLYDRLSEWINIHYRDSLKLED

LADPSLVDETQEALNVLTQILDLGRIYDFQQ

>tr|D6YU34|D6YU34_WADCW Na(+)-translocating NADH-quinone reductase subunit B OS=Waddlia chondrophila (strain ATCC VR-1470 / WSU 86-1044) OX=716544 GN=nqrB PE=3 SV=1

MLRKFMDYQLSFVEKGKPLHFLRPLITAGDTFFYEAPINTKEGPHIRDAIDIKRWMIMVV

IALIPCILWAFWNTGLQAIVYSSGDFKLMDNYLAATTSFSDYFAFVGKDNLWMKILAKGF

SILFPVILISYAVGGMVEALFAIVRGHEISEGFLVTGILYALILPPTIPWWMVAVGVAVG

VVFAKEVFGGSGMNIVNPALACRAFLFFSYPGKMSGNVWVGNNPATVRESLLKMNQESGL

GPLDGYSQATKLARFNVTPEIKRVHVDAIATNNLGDQVGTFETIKAQFAKWTSAADQTAS

LGELTGEQMQNFVTTPLAEGGLGLSPGYYEDAYHFASLNYGFGHNGDWNLFFGDRLGSMG

STSVLCCLLGALFLIWVGIASWRTMAGMFLGAFLTASAFQLASTYFGADFGAWTPAQFGF

PAYKHLILGGLAFGAVFMATDPVSSPDMHAAKWIYGLFCGVVTVMIRVINPAFPEGVMLA

ILMGNVFAPLFDYYAAIYYRKRRVRRVRTAAAA

>tr|D6YTX4|D6YTX4_WADCW Uncharacterized protein OS=Waddlia chondrophila (strain ATCC VR-1470 / WSU 86-1044) OX=716544 GN=wcw_0210 PE=4 SV=1

MKRLLMIILLFPVLLPGTDMPETGTLVVTYQTDNKGDRLNRVRFWLTCISHIKNGSHKQT

LYPQGATYVDDANTQTRMVVIEDLPPGDYSIDFVVPNLDDRFSPVPQRKANIASGETTKI

DQKIKFNQKNPASTPEHSPDIAQNLPDETERQKEADVFYGKLIVSFDLPDTPKRISDIHF

RLTSPFGETTAHPIPEDTVIGLETGKMVMIPKLPAGNYTLEFFVEGSQEALAKKSFAVQA

DRTRSVHQVLTIAPTDAKTEIAEQASPSLILIANTPSAEFELLALESGKSYQEKGEKASF

NEIPTGVYELRFQSRDPFFIPPSTEKIEIRKEGLMLKEISYTPLGKIDISTNVNEAKAQI

TPIEKGLHPYKKEIREGKATLYLPEGRYRITFQPVKGHEDPEPVDIDVRPLQTQQINVHF

>tr|D6YUY8|D6YUY8_WADCW Proline amido peptidase OS=Waddlia chondrophila (strain ATCC VR-1470 / WSU 86-1044) OX=716544 GN=wcw_0579 PE=4 SV=1

MDFPDKLKEVQKYLFEKCLDGWLLYDFRNQNDLATRFLEIPDSKMLTRRFFYWIPAKGEP

QKFVHAIEADNLSHLPGKQVLYSTWQKLEEILKKALSGKEIILMEYSKNNAIPYLSKVDG

GTLELVKGCGVEVESSGTLLQRYTSVWSKDQYADHVEAASVLSKAAEDAWRFVGEKLKNQ

EQVNEYQVQQKILEVIKSCGCQTEEPPIVAVNAHSADPHYLPCSDRWDPIKRGDFLLIDL

YCRKNKERAPYADITRVAVAASKPQEKHREVFEIVRRAQAAALELVRQRFQEKKKLMGRE

VDRVCRTLIDASGYGQYFIHRTGHNLDVQLHGPGANLDDFETRDERELLPGTCFTIEPGI

YLPGEFGIRLEYDVFVHLDGEVVVNGGMQNEIACLL

>tr|D6YTN9|D6YTN9_WADCW 2-deoxy-D-ribose 5-phosphate aldolase OS=Waddlia chondrophila (strain ATCC VR-1470 / WSU 86-1044) OX=716544 GN=deoC PE=4 SV=1

MAMQSKIKSRYAFQQIASLCDHTYLKTVEFYRQVSRIGQSPILRRQEEFYRFLRETIESP

LTPYGICVGAEDVPHVRKFLQHHKKDSIALVATVGFPDGSWHQKHYKFFEAQYALSEGAD

EIDMPVNWKALKQGNHAEVLEELQTVCGLVRQKKGVFKMILETSLLNDRQIIEACKLARH

AGVDFVKTSTGFGAHGVGPRQIELILDHWDGGLKISGGVNAGNVAIYLDQILKRLKGPDH

LLNPMKVRIGESDLLSDLM

>tr|D6YTL7|D6YTL7_WADCW Uncharacterized protein OS=Waddlia chondrophila (strain ATCC VR-1470 / WSU 86-1044) OX=716544 GN=wcw_0103 PE=4 SV=1

MNDIENLMNREHLEEIVNHYSVEDLIKLLSFKKAMALSKLLLENENFDFDIQEYALNLIK

KIRQVYPNKWDKDWKHEAYLGYAYGILGCDIEQEFDAYSIAAKKAVDPPLEISMHMALLW

SYPGVYKLKMDEENAIKILENVASQIPYMEAVGGLIRLYEETKQVGKIAYWKEVLRESEK

KNLCDRYLYLDFF

>tr|D6YV47|D6YV47_WADCW Uncharacterized protein OS=Waddlia chondrophila (strain ATCC VR-1470 / WSU 86-1044) OX=716544 GN=wcw_0640 PE=4 SV=1

MHCKELALLAGRAWQSKETRFVHYCYHSDSRDPIPLKENFCFVLALLRSRTAENILEAKE

LLEKLLSYQVDGNFPIYLHEFPTCYDRFRAAEVLPSLIWMARGFHHVLGKELKDKLELSI

REAVKQSLAVLESYQVDELTALRIGCSTLAAGCYFGCDHWKELGSKLTAQAEALGIQPSW

FVSEMLGHRLASLNQIDVWPRLWQHCNEMYHEKLAAYCGPHLSEKQWLAGPEPNLFNLYM

KDSKFQERLHPSYLLGALVHPFEKQEYLPQYQTGSLLGRKWEVYKTDCWAVSLLEQEMSV

NQMFSPVYLIWNGGETLQSLVAQGGNVEQSRFIWKDREIELLFYLGPEADNDHPKDRVEA

AFFCSRESTALSIEGKKATVFRLGEKVEIKSLLPFSIEFSLLEGSGDFTGHISMGNRPCQ

IFSEGEARDWKIAIRSVRRTPDCILKAKLTING

>tr|D6YS34|D6YS34_WADCW Putative tRNA (cytidine(34)-2'-O)-methyltransferase OS=Waddlia chondrophila (strain ATCC VR-1470 / WSU 86-1044) OX=716544 GN=wcw_1530 PE=3 SV=1

MVKVVLYQPQIPQNTGNIVRTCSVTGSDLYLVAPLGFQITDRWLKRAGLDYWEGVNMQVL

ENLEEVLESQQTPFYFFSSHAKRPYSKISFTTDDWLVFGSETHGLPKTFVEKWPEKFISL

PMIEGKRCLNLATSVGIAVYEAYRQNQFNFFKGD

>tr|D6YRL3|D6YRL3_WADCW Uncharacterized protein OS=Waddlia chondrophila (strain ATCC VR-1470 / WSU 86-1044) OX=716544 GN=wcw_1357 PE=4 SV=1

MSEFNHLIALTKLHISQHYGEKSWIYTDPDTLANYREFAQRSKKAAPKQLPEKSKPLPRI

AEPVRKQPIIKKTEPPALELPKEVEQRITPKPVNEVDFSDLIKIVKTHFPAQKILDSQPD

DARAKETAQKWKHPAIPPEVWILDSSRAPEERLFLENIAQAIDLYFYPAAVLPISKMDEE

PAPRLILGTKDLLNGIKAPSIAMESISFYLETPKEKSRLWKDLKNTLQSS

>tr|D6YW01|D6YW01_WADCW Uncharacterized protein OS=Waddlia chondrophila (strain ATCC VR-1470 / WSU 86-1044) OX=716544 GN=wcw_0951 PE=4 SV=1

MLPKPRFLKSLEEIFIHSWWVILFSLLCYTWLEHENRQQRVAFHELTQQLMELKNHQIEA

SKQRIYLLNQINSQSDPAWVELTLIKILGLIPENQKKIYFSHENE

>tr|D6YVX2|D6YVX2_WADCW Spermidine/putrescine transport system permease protein potC OS=Waddlia chondrophila (strain ATCC VR-1470 / WSU 86-1044) OX=716544 GN=potC PE=3 SV=1

MIRSRLCFGVTIAIFIFLYVPILLLMINSFNASKYGTVWAGFSLKWYYTLFSEPDLWKAL

VNSMIIAVSSAATSLVLGSLAAFTIYRYRTPLQKVHYGLIYAPLLLPDILMGISLLMLFV

AWNVKLGLFTIYIAHTTFCISYVTMLMLSKLQNFDYSLVEAAHDLGAGAFETFKKVIFPL

ITPGLVAAVLLSFTLSIDDFVITFFVAGEGSTTLPLYIYSMIKYGSTPIINALSTLILLG

TFLLVFVYHTFAGEEQI

>tr|D6YWT2|D6YWT2_WADCW Uncharacterized protein OS=Waddlia chondrophila (strain ATCC VR-1470 / WSU 86-1044) OX=716544 GN=wcw_1236 PE=4 SV=1

MQEPTAAQLIKRLFSVIGLGVAAAIGVAVFFIYFFSPSGSYPISELLLNPQVAKELRYQV

DGKTYVFSRVELLSYNEAGNTWETKQVSLDQYQKFYSLIQSDKSEVIPDDAVKNAFYQSN

PAILSIVVRKEGVNTDQVFQEVQISTGGDAYRVSLREEQALPQWAYFTHADIYQKTLEAF

GWDL

>tr|D6YTV9|D6YTV9_WADCW Putative serine/threonine protein kinase OS=Waddlia chondrophila (strain ATCC VR-1470 / WSU 86-1044) OX=716544 GN=wcw_0195 PE=4 SV=1

MDMTIKVKERIAFYESLKGASEKKAGKSIHHLQSSGKSKKVNLETIDKVDEYAKEVIGEY

RVYHREDSNKTYLIHSRVVERDPLVSEVIRKAKRVSQKQKIEPDERKRKIDEMLREITKR

LVIKDGRLMRPYFESGATKYYDTGMTPKSLSVLLEQVCRVEDVETRVFHLIGEESEVKGK

FLSISRNESSKDRCIVHIGVQIGRGGAGKIHKVWNLNNNRVLVLKKSFGQDLQKILERKT

SLDIEASHLKKINPKGRCNGLQRAPYLVFRMSSRSSSFVGLLSARYDSDLSAALSPVDME

GNENKNKRELNLEQMIDGAHQIFSGIIHLNRSKILNADIKSSNIFIRQRSNGKLKFHISD

FDQAVFLNDDLYRSEHFARDPVGGFSVDSLKINHQRWAEKTAKAIVLRLEALQMSSKQRA

KVRKKLKKNKVKSKLEKEKIEVYKAKTKVLEEELIELKRSFSEIAFDIHTVSGGHILFQM

LSSELSYDIDDIDFAKLERKVNFSDKERDLYRQLVDLCVRCLPEIEEAPEVVNLEHFYHP

IGISVDECLNTLEEISGLR

>tr|D6YSL7|D6YSL7_WADCW ABC transporter, ATPase subunit YbhF OS=Waddlia chondrophila (strain ATCC VR-1470 / WSU 86-1044) OX=716544 GN=ybhF PE=4 SV=1

MSAVVIQNLTKTFDKGKRIALNNLCADFPKGKISGIVGPDGAGKTTLLRILAGLMQIDSG

QCLVWDFDTEKEPEKIQESLGYLPQKFGLYEDLTVSQNLSLYKDLQEINENDPVFAKLME

FSGLASFTERLAGNLSGGMKQKLGLISILLRKPALLLLDEPTTGVDPKSQNDLWEMIAEL

NNQGMTIIASTSYLEEAEKCHYTFLLNEGQVLYRGTPSSLKETMKGKTFYFEGVGDVKRT

VLDSLIGKNGIVDTIIEGSKIRVTFNAANPDQNPERYGLNEKAKLLPRDPYYEDAFISIL

GGIPKRPALKLTKSPQAEMETDLMIQAVDLSRSFGDFKAVNCISFSVKKGEIFGLLGPNG

AGKSTTFKMLCGILPATDGEALVGGKSLRQTPDEARGLIGYMAQKFSLYNNMTVAQNLRF

FSGIYPVKNRKKTREEMIAVFNLEEYLDVLTVDLPLGYKQRLAFSCALMHKPQILFLDEP

TSGVDPLTRREFWHQINLLADAGVSILVSTHLMDEAEFCDRIGLIYKGSLRIVDTPEGLK

KRVPKEISEKPGLIDAFLYFCSEREER

>tr|D6YU63|D6YU63_WADCW Nitrogen fixation protein NifU OS=Waddlia chondrophila (strain ATCC VR-1470 / WSU 86-1044) OX=716544 GN=nifU PE=4 SV=1

MTFELLTMSFPWNRYSRKLSAKIENPHNVGVFDPEESEARGMRLVIGIEGEIRDGNCVQI

YWLVDKEDGTIVDAKFQVYGQSALIGAAEVACDLIAGKNYDQAKRIGVDLIDKQVRDRSD

EPAFPQETAPHLNLVLDAIDHAADQCSDIPLPVAYAAPPAPKQFGEVLEGGYPGWEKMST

AKKLAVIEQVLNDEIRPYIALDGGGVEVKELKENELVIAYQGNCTSCFSAVGATLSYIQQ

TVQARVHPDLRVTPDINI

>tr|D6YRH3|D6YRH3_WADCW Alanine or Glycine Cation Symporter (AGCS) family protein OS=Waddlia chondrophila (strain ATCC VR-1470 / WSU 86-1044) OX=716544 GN=agcS PE=3 SV=1

MTWLDTIYQWVWGIPMLILLLGNGLLLTVLLKGLQFRYLPYALRLIISSDKEQDSKVKGD

ISNFESLMTALAGALGIGNIAGVSTAFAVGGTGALFWMWVMALIGMATKYGEAILAVKYR

VMDDRGEMCGGPMHFIERGLGWKWLAGAFALFGAIASFGGGNMIQANSVADVMMNLYHIP

SWVSGVILALITGSILIGGIKSIGRFAAFMVPFMAVFYIGGGCLILLKSYDRIPDAILSV

FQHAFTGQAAFGGFAGSTLLMTIQVGMSRGLMTSEAGLGTASIAAAAAKTDFPGRQAMIS

MTGSFIATIVLCTVTGLVLGVAGVQGALRPDGVPLNGASMTMAAFESAFPWGGYIVTIAL

ILFALTTLLGWAYYGEKCLEYLFGTFSILPFRLIFTLIIIPGAILDLEVVWKVADITNGL

MAYPNLIGLTALSGVILKETKTFLQVVKNETGSSSSNDFHCA

>tr|D6YWE0|D6YWE0_WADCW Uncharacterized protein OS=Waddlia chondrophila (strain ATCC VR-1470 / WSU 86-1044) OX=716544 GN=wcw_1094 PE=4 SV=1

MLIDAKIINDPEKKPRYSVEIPLVDALTQGRTKKEALEMAVDAIECLLDAYFEEGSGKRA

KVTASSLKGNHFFIKAEDQNLLTSLILIRQREKHKTTYSELTKRLKAGSTFAYRRYEKGN

VSISVKKFYDLMKAVAPEKNIVLRME

>tr|D6YUT3|D6YUT3_WADCW Uncharacterized protein OS=Waddlia chondrophila (strain ATCC VR-1470 / WSU 86-1044) OX=716544 GN=wcw_0523 PE=4 SV=1

MNSLSDFSVSSSSSSSIVVDGGLESPRSVFKKHPFTLAEGKLKPTNYINPGIGKRLEESY

RDVREINKKIEKSMRKLNPEDLSVVNQKALERLGFLIRKQQEAYGRYCHNMSMLHIGTDY

VEREQGKKFPFFLFCRETFFQNTDEKLREYLNKTIVLDPSKPFHKKQRFDLVHPKWLQEL

FPKEKKEQTVEVDKKKRELHLLVLYKEALCYFCHRLKNEQASLKKTSEFANVDQVDQMIG

KIKKILEKNKDVQKLLELVSEEVSIYLEGSFQQQIFDKAAEAFLHALEKSYQREVAYCNE

SICKAVLEAFNSEKNRPLAVESSRAVIGGIITFLEKRGKHDKILLPPFKGPPMDAEGDAD

YEKLMERKVQEYVVECQKLVGEKVFCDDQVVINFSMLLPQFASQECPGPENESALSAQER

IFNFLMTLEEDQGLTKKFRAFMNAELMRSNRKCMKRLGEKNKDCMEIRKYLLYFYRAPFQ

TYLNEPVKALKEIDERIFKEIYKTSIFVQLKDWKDDETKKIIIGFDEEVVNFQFLRRGAI

GLASENPAGVLVQDLSIMVSRPRKYETEIKDPVKLRFYCEKRKDLTREEEKELNRYLGRI

AFILKMLKFPPLEIREIEDES

>tr|D6YT08|D6YT08_WADCW Uncharacterized protein OS=Waddlia chondrophila (strain ATCC VR-1470 / WSU 86-1044) OX=716544 GN=wcw_1865 PE=4 SV=1

MINTSYFSFWKGITGNSKKPADSERELSEQDNKASKYSMKIHIWQTDAEEGRCGHTSVSL

HEEKDGHSEEEYGGMWPDKKNIILLSHPFAYLLASVKGEIHPKRSHCEEREGDDEAMRPD

SVYTIDVTESKYRELKEKMSKDKGDVDKGMVLYSLFQKVNLLSVVKLCANESFMRNVMDG

DFLEEYPEVLENAASIRNLRSEHCTSFAKSYVETAGFKVRESFSPWGISPLGLNSQLAKL

SEEHAEIQAEHYSRTPKFRDRYSDDL

>tr|D6YTU9|D6YTU9_WADCW Uncharacterized protein OS=Waddlia chondrophila (strain ATCC VR-1470 / WSU 86-1044) OX=716544 GN=wcw_0185 PE=4 SV=1

MNLSQSKPERQAVHLEMQAKLKSSDNKPIQS

>tr|D6YTN2|D6YTN2_WADCW Putative biopolymer transport protein TolR OS=Waddlia chondrophila (strain ATCC VR-1470 / WSU 86-1044) OX=716544 GN=wcw_0118 PE=3 SV=1

MARRGIRHRREEGERPAVDLTPLVDVVFSILIMFIIVAPMLNLDRVELAEGAKEAKSSDV

SVRDSSLVTIHVREDNSIWINQKLVDAVHLPEILKQERQRHPDARPQLYHDRRAQFGTYQ

IVKNALESAGFEQLDIILKPV

>tr|D6YTZ5|D6YTZ5_WADCW AMP nucleosidase OS=Waddlia chondrophila (strain ATCC VR-1470 / WSU 86-1044) OX=716544 GN=amn PE=4 SV=1

MAQIGEHVFDPREEKIAIDCLERYSGSIVSSYQSFLLLTNFPRYVDEFAKSRQIPVHEGS

MFKSAHSEKEGISILDFKIGSPAAALVIDICAHLPIKAALLLGMCGGLRRQYKVGDYFVP

VAAIRGEGTSDFYFPSEVPAMANFMVQKVATDQLTAENASYHIGITHSTNKRFWEFNEKF

RQRLIETRPQAIEMECATLFMASYYNKLPLGALLLISDLPLEPDGVKTKRHAEKVYLDYT

ADHVEKGVRILQSLRESIKFESKGVYRGARKRFEDAD

>tr|D6YTK5|D6YTK5_WADCW Putative type III secretion inner membrane protein SctT OS=Waddlia chondrophila (strain ATCC VR-1470 / WSU 86-1044) OX=716544 GN=sctT PE=3 SV=1

MTDNYWEFFLNSSLSIEDPGAVFILLFLFLARILPIIALSPFFGARVLPHPVKLAFAISL

FAIFVPHLLVVTQSKLSFTPLSLALFAKELFVGYLIGFLISIPFTIVQTAGMLIDHQRGG

ASLMVNDPTIQNQSSPLGTMFNMVLIYIFFMIDGPFYFIDAINTSYSAIPPDRFFNPQFF

DKSSAFSEIAFQLLNNAMVLAIQFASPALIAILMTDVFLGIANRLATQVQITFLGLPLKS

LLGLTLITIGWKVLINQMAEDSHRWMNVIDRVLIIFDIGRQ

>tr|D6YTM0|D6YTM0_WADCW Protein translocase subunit SecA OS=Waddlia chondrophila (strain ATCC VR-1470 / WSU 86-1044) OX=716544 GN=secA PE=3 SV=1

MISFFKKLFGTAQDRIVRRYSKLVSKVNEWDEKYKSLSDEQLQAKTDEFKQRLKSGELLD

NLLPEAFGAIKNACRRHVGTEVHVSGYHQQWDMVPYDVQIIGAISLHNGNISEMHTGEGK

TLTAIMPLYLNALTEKPVHLVTVNDYLAARDCEWVGSILHWMGISTGALTNDTPLEERRE

LYKKDVVYGTASEFGFDYLRDNSMAKRKEELVQRGHYYAIIDEVDSILIDEARTPLIISG

PAPESRQMYDELKAGVSELVRRQRDLCSKLASEAKKVIDLGDRSEAQDKSQTKEEKQREE

EAFRKLWLVSKGTPRNKILKRIRENPDARAAIDEWDLYYYSDSNKEEKAEKLSELYVVVD

EKSSEYELTDRGIAMWHEFTHGDGQGEDFVMLDISEEYLKIDLDNSLTDEEKMQKRLEIQ

EEDAKRKERAHNLRQMLRAHLLMEKDVDYIVQDEKIVIIDENTGRPQPGRRFSDGLHQAI

EAKEGLKIQKETQTYATITLQNFFRMYEKLAGMTGTAITEAGEFKQIYKMEVLEIPTHRP

CIRKDFNDEIYMTEREKYNAILKDVKEIHELGRPILIGTESVEVSEKLSRIFRQNKLEHT

VLNAKNHMKEAEIIAEAGRRGAITIATNMAGRGTDIKLEKGIAELGGLYVIGTTRHQSRR

IDRQLRGRCARQGDPGSSRFYVSFEDSLLRLFASPRMTQILKKFRPPEGEPISATILNKS

IETAQKRVEQRNYTIRKHTLEYDDVMNKQRQEIYEFRNDILQTDLIEEVACELIEHVCVD

AAEEHFHSRTDEQGWDPEGFRNWIMTQFPVSFEEGEFDDDHSDTEELAQKAVNVIIDAFR

KRLENENAKVAYDLPEGVQAPSKPANEALRHLMIRKIDKDWKEHLLTMDHLRSDVNMRAV

GQRDPLMEFKHEAFRLFDLFGKKVRKEITHDLFRFEIIAPEAQEIEQLLNRLQMERNRSF

LSDFGEQVPKSITEGAPSPMPYEAMKPTEFNQQEVEKELPVTVPPKTGRNDPCPCGSGKK

YKKCCGIHQADE

>tr|D6YT72|D6YT72_WADCW Penicillin-binding protein OS=Waddlia chondrophila (strain ATCC VR-1470 / WSU 86-1044) OX=716544 GN=pbp2 PE=4 SV=1

MPMPKRRRRARPRLTIEEKAAKVLNLVFIGFLLITLRSWHLSVILHEEKLEEARRPQKRV

VIESSKRGTIRDRFNIPLAINKMQYNLAISYAQIRQIPGVVWEKENGKKVKRYIRREYIE

KLSAVVGEELHLDPDYVEDLIYSKAALFHHLPYVVKEDISEGQYYRLKMLERDYPGLHTQ

SVPKRYYPYGKVGGEMIGYIGAISRQEYESVVQEIKSLEEWLGKYEMGQDPELPEGIETV

EGVEKRYKEMVEHAYSINDYVGKMGIEGKFEEVLRGYHGKKAFASDAQGNIIQELLEGKE

PQSGSRVLLTISQELQEYAEKLLIQNEAVRVPRVSRVNAQSRKKLEEKQHWIKGGAIVAM

DPFSGDVLALASYPRCDPNDFISSGNGEERARKTANIRKWFETEEYIADVWNQKRPLDRE

FFDLKTEQIAEEAIWVDWQTYLEMILPIDSPIIEALNRVGSVKNAVIIQKHLEKLLVFSP

SQSAYALFNQLYSDPPHQLYGRRLPAVQQEHLEEAVEKHRETVQFHKKALDPFFNGLESN

YDKVMFLDLVRIVVDPERISDTLLKEIGSQSLVEYRNAQSAFVLIEETVRQMIWELFREV

HFKRWRDLYQKEFLKQKRREEKINKVRYAKPYLDLLEQQELLMFQEFWEQHRYALLATFM

TGVSFQDYPEIKPYQEMLASWEKELKGGAHQALSWSRSYWKLHQSVDGLSPEMVQDYLAG

LRGFDRLNRSLLGRYRHLRSQDGQQLEKHLAAGFYPNYGYGFARSHAYRQAAVQGSIFKI

VTAYEALVQTFNAFQGKQAGEIHLNPLIMVDDIYKSGKATFVGYDKNGKPIPQFYKGGRI

PRSHRSGMGKMDLVRAIEMSSDPYFSLLAVDVLANPEDLARAARDFSFGSKTGIDLPAEI

PGQIPYDLSVNRNGLYAMAIGQHSLVVTPLQTAVMLSAVANGGKVLKPKIVNMIVGKNEK

NEGTILSFDPVVNNRIFMPDAVREVLLEGMYRAVFRSQTASLGSLSHLYENYPEAISDFI

ELKNQLVGKSSTAESMEQLDLDQNLGTNLYNHIWFGGILYTPDQKSEPKTFVFNDRFGTP

ELVVVVYLRFGAWGKDASPLAAQVAQKWREIKSKHLSRS

>tr|D6YUL5|D6YUL5_WADCW Uncharacterized protein OS=Waddlia chondrophila (strain ATCC VR-1470 / WSU 86-1044) OX=716544 GN=wcw_0454 PE=4 SV=1

MVKIMVMLTLDSVRKVFLDVIEEKKSFGEASRWASEMIEKDERGLLEFDPKEDISTIFSG

LTYLLGVDLEESPGVYFHSIQNVKDEYNELFY

>tr|D6YSZ6|D6YSZ6_WADCW Uncharacterized protein OS=Waddlia chondrophila (strain ATCC VR-1470 / WSU 86-1044) OX=716544 GN=wcw_1852 PE=4 SV=1

MKKKEIPMEAQRSIKADATGRINLGKECAGRLFTISKEQDKIVLEPARIVPEKELKSHET

IENIILDENEWFRFQEIMESDDEPTGNLRRLMKDSD

>tr|D6YW24|D6YW24_WADCW Uncharacterized protein OS=Waddlia chondrophila (strain ATCC VR-1470 / WSU 86-1044) OX=716544 GN=wcw_0975 PE=4 SV=1

MAENKKTIKTQPSKKSITSKKKSTSDKTVESERLLTAEGWKRRRRKEMK

>tr|D6YUM6|D6YUM6_WADCW Pyruvate dehydrogenase, E1 component, beta subunit OS=Waddlia chondrophila (strain ATCC VR-1470 / WSU 86-1044) OX=716544 GN=pdhB1 PE=4 SV=1

MPEMTIIQSLNHTLHEEFARDERLISFGEDAGAFGGVFRVTEGLQEKFGEERVFDTPLAE

QGIVGFAIGIAQNGLKPICEIQFADYIFPAYDQIVNEMAKMRYRTANQYSAPVVIRTPCG

GGIHGGHYHSQSPAAQFLHTPGLIVICVSGPYDAKGLLTAAIECNDPVLFFEPKRIYRSV

KEEVPLERYTIPIGKADLARQGKDITLIGWGAQHHQNMAAAEELAQEGIDVEVLNLRTLN

PLDEPAIVASVQKTGRCVVADEAPKTMSFAAEIAATIMEKCFLSLEAPVERCCGLDTPFP

HTLEHEYLPDAYKVRQAVIDTLEY

>tr|D6YSA2|D6YSA2_WADCW Uncharacterized protein OS=Waddlia chondrophila (strain ATCC VR-1470 / WSU 86-1044) OX=716544 GN=wcw_1602 PE=4 SV=1

MTFLLPVKIDGAEYQAHAEYTSNAVGEYYKERLKPFADVFNLGLNDTSPDTDAIRALIEN

GTYHTEMETALQELLDLAQNGITDPNDPVDPNRKHYLSVEMSASLNQLIKTLKAAGADID

VSTGTVNITANNVVEWKSLSVNSDAIRQVIAFTLDTAGDQNRTLQALVELIYVKTGNEIL

TQNLNDLETALSTTKDSLKTLTQLQDLHNKIETTKQEQSFVSFASQNYSDGNNSRPTAFV

EGSGFDGIAGQASKFFQELQPTVDVNNLTNADTALFLELRNDLVQQISILTQTTPGAGTS

GEAGSLLDKLKTVKSHIDMAIEKAGGVGDTDQIKRALRYWILDSQTDNAEIDGIEPGQIQ

REITAALSAGQSLNDSQKEEVRRYLFVFEEYYKSAAAILNKITQLLERMAQGIAR

>tr|D6YT09|D6YT09_WADCW Putative rRNA methylase, SpoU family OS=Waddlia chondrophila (strain ATCC VR-1470 / WSU 86-1044) OX=716544 GN=wcw_1866 PE=4 SV=1

MRTLSSLQHPLIKRLTALRKERKMRQQEGSVVIEGKKLVRENVRLVRTLLTTSPSTEFSL

PESAEVYAVSPEMIQKISGVKSPEGVLAEVKMPSFKLPARLQRLLVLENLNDPGNLGTLI

RTSLAFGWDGVFLLGNCCDPFNDKAIRASKGAIFKLPIVVGDLESLKTLLIDHQMSVFIA

DLEGKKIGAIPKVSKMALVLGSEAHGPSKEVISIGERLTIPISSGLDSLNVAVAGGILLH

HFQ

>tr|D6YTU8|D6YTU8_WADCW Serine protease OS=Waddlia chondrophila (strain ATCC VR-1470 / WSU 86-1044) OX=716544 GN=htrA1 PE=4 SV=1

MGGTLKDISFNTYAPLAGGFSESQVFITWELFDTLNQNVIFEFSTYGYAKCDGVGGQAIF

NAFKHALRELMANDEFVKLTTDKSSEKSDEFQYKHSQLINIEAEHQSFKLPEELEKVLDA

VVLIKAGQTHGTGFAISKDGYLLTAAHVVSGLDKVHIVNKLGQTCEAEVLKLDKINDVAL

IKTQDCIFNPLELELENRTSIGAEIFAIGTPLNENLSWSTTKGVLSGYRNIDGKRCIQTD

TSLNPGSSGGPLLNKEGKVVGVVSWKISGTEVEGISFGIDIEAVPYVLGLSFN

>tr|D6YVB3|D6YVB3_WADCW Uncharacterized protein OS=Waddlia chondrophila (strain ATCC VR-1470 / WSU 86-1044) OX=716544 GN=wcw_0707 PE=4 SV=1

MDIIDHLTNEKISKNFNNQFDLVNYAIKLAANMIQTGREPRVKMNTENPALLILEEIIEG

KDTFVEVSAKKEQKNFKEIELERVKEKVEEEADDSELLEDEEETQEVLS

>tr|D6YRZ3|D6YRZ3_WADCW 30S ribosomal protein S8 OS=Waddlia chondrophila (strain ATCC VR-1470 / WSU 86-1044) OX=716544 GN=rpsH PE=3 SV=1

MAVTDPVADFLTRIRNGLKAQHRYVDINWSKMKQSLADILKNEGFIENYLVKKDNNDRGT

IRVFLRYGAYRQPAIKGLKRLSRPGLRKYVKHNEIPKFYGGLGVSILSTSSGILSGNEAQ

NKKVGGELLCLIW

>tr|D6YWL2|D6YWL2_WADCW Uncharacterized protein OS=Waddlia chondrophila (strain ATCC VR-1470 / WSU 86-1044) OX=716544 GN=wcw_1166 PE=4 SV=1

MLLQCHGAAGQYNAQPSQHNWNRPMGLSKLAAGCLLFLCSLVQAEPNRTLYLLRQGHFQS

AIENYEQHVFETGKHDYQILQQIGLTLIELGWKCSDPEIKLLSLFGAGISAHDQAQHLLE

DAIRANDPRFQLAALNFLSKNYTDDSSRFLNLAMKSDFLPIRLEAVFHMAQKQHPKTAYQ

IESLMQKVPPEVLPLFPQLLGASGDRDSMIMLRRLMSHPLLAVRLEAILSAARAGRDDLL

PEIRGISSHHQIEEQEAAAVAIGLLKDEESVKRLTELKQSKAKNVQIASLQALYRMGRED

AALPLIEMARQGNLFAISVLSELNQSENTLYELCCTEDLNIRINAALSLLERRDPRCLQT

LLELFLRDPRDLALSQTHSAGKGLAAYKIVPSAQQNFESTPLLYEQTLNLKEAALQKTLE

LPEKDFFAFARLLFDTRQNDLIPALTHLLEECRSPGAVEILKTYREQIGAPLIRYWCNLA

LYRMKEEGPYTPTLKFWVKEYQFHDMIQFRPMLPWKMRSANASQYQLTPQETSRLLVETY

ESLVQLQDEEGIALILEAIKSGNRKNRYALAGLLIRAAN

>tr|D6YV59|D6YV59_WADCW Putative nucleoside-diphosphate-sugar epimerase OS=Waddlia chondrophila (strain ATCC VR-1470 / WSU 86-1044) OX=716544 GN=wcw_0652 PE=4 SV=1

MNGIIFGAGYVGCACIKEWPNKQDSLIATTTKEEKVAELRKLTPRVEVVKGSDRKKVAEL

IKEADFAIICAAPNHWNQYTETYLETAEGITQALSDRSRSFYLLYTSSTSVCGNHNGATI

DESSPLLADSENGKILIATENCYLRAASPMVSICILRLGGIYGPGRKIIDRARRFSGKTM

TGRDSPTNHSSLPLITKGIIWSVHHRLTGIYHLVEDAHPTKNELYGNLLETLKLPPPVWE

QPHTGGAAVSNRKFFKTGFQ

>tr|D6YVC2|D6YVC2_WADCW Putative membrane protein OS=Waddlia chondrophila (strain ATCC VR-1470 / WSU 86-1044) OX=716544 GN=wcw_0716 PE=4 SV=1

MNSDYPLSLGGGDTSFSSFESIDDADPSLDTLLLPLTPERSSVNDIKTAAKRMRGAPVGS

LYKNLGEAVTRQLTGFSPKEIKKSFRESWAKDKNLGPVLGRLKMAGRCLGFAIGTLVNVA

SKIAALSVALIAATTSGMLQFIFTPIKSFKKPHTVMNNIIGFGTTSGVLAGSGISFLGMK

LSQCVAGIKLEPQDEGSSTSTFDRAVEDSSGAGLIGMVIGAGIACLYPAMWQLPFRDSGE

NQPSIEEIEARMQEILAARKADSLSSD

>tr|D6YST4|D6YST4_WADCW Putative lipopolysaccharide heptosyltransferase II OS=Waddlia chondrophila (strain ATCC VR-1470 / WSU 86-1044) OX=716544 GN=waaF PE=4 SV=1

MTKKWPKQKPKNILVRMPNWLGDLVMATPVLKDLRDAFPKAEITAMCQTKSCALIQHDPN

IDEIFCFQKPSGWVHRRHHLEIIEPLRQGKYDLGILLTNSFSSAWWLWRGHVQNRIGFKD

HLRSFLLDKAVDYPSNKESQHLVYTYKALLEPLGILNTHSTPHLYLKDSEIESAKEFLRK

YNIDPDKHKVVGINPGAAYGSAKCWPPQRFESVTRKLLESPNHRAVFFGDLGGAELVNDI

CRNLPERAVNLTAKTSIRELMALIKLCSVFLTNDSGPMHIAAALKTPLVAIFGPTSDLKT

GPFGHGKVIHKHVECSPCYKRVCPIDHRCMTQIEDREVYQEIMSMLEKSGQTARV

>tr|D6YV53|D6YV53_WADCW Putative metallophosphoesterase OS=Waddlia chondrophila (strain ATCC VR-1470 / WSU 86-1044) OX=716544 GN=wcw_0646 PE=4 SV=1

MKQALVIFLLSMVVGPLFSQEEGEVRVALVGDGIGQGSEGVDAESFARLLAKVAEQKPNV

VFFLGNLVDGLEQSTAPESIKKLKNHLEQFTRLTDTYLGKQVKIYPVIGNHTFVNTQAAQ

LFKEHFKIKDTAPLESYQWAYAVNIEQTQFIVLASGLFERKYRGYRQEVLTMPLLDWLEK

ELRTNADSIRYRFVIGHMPAFSSRATEGIYSGLDKDLERRDAFWQVLKNNDALGYFASHE

PLYDRLNRDGVWQIISGGAGMMENGDGSSSVFQHFILISIPKNKAKNPVLESIDIKGKIW

DEFEIVPLDKPVHQLRISNRG

>tr|D6YVE5|D6YVE5_WADCW Aspartyl/glutamyl-tRNA(Asn/Gln) amidotransferase subunit C OS=Waddlia chondrophila (strain ATCC VR-1470 / WSU 86-1044) OX=716544 GN=gatC PE=3 SV=1

MAHLDKQMIEYLSDLSRIDLSEEEQQSLLEDLEKILAYIDLLNEVDTEGVEPCNHVLADM

RNVMREDEVGETMPREAFLNNAPAQIGGMIRVPPVIKGK

>tr|D6YVA5|D6YVA5_WADCW tRNA (guanine-N(1)-)-methyltransferase OS=Waddlia chondrophila (strain ATCC VR-1470 / WSU 86-1044) OX=716544 GN=trmD PE=3 SV=1

MRMDILSLFPDYFKGPFDESMIRRAIDAGILDIRLVDIRDFAEGKHRRVDDRPYGGGPGM

IMMPEPAVQAIRSVRQPEAKVIYLTPQGKPLNAAKCRELAEESHLIFLCGHYEGIDERVI

DIEVDEEISIGDYVLTNGCAAAIVCVDAIARFVPGVIGHESAADEDSFENGLLDCPHYTR

PEVFESLSVPEVLLSGNHKKISLWRREKALEKTQRIRPDMADRRCKEK

>tr|D6YV15|D6YV15_WADCW V-type ATP synthase subunit I OS=Waddlia chondrophila (strain ATCC VR-1470 / WSU 86-1044) OX=716544 GN=ntpI PE=3 SV=1

MRVDVKKFLFVGFRGALQAFFEKAQEAGLVHFIDPRRLKAKEVPQKIQDIVNAIKVVREL

PTLKQEEPEKFSEVNVIAEKILSMKHDIERLEEEKRTLKLEISRVDVFGDFSLEDIQHIE

KETGRTLQFYFGKKGTVEEELPDEVIYIASKHGLDYFMAVNKELKHYEQLVEMKIDQELH

VLRSRLEEVQNDIVRLEASLKKYNKYNEFLHYALTVKYNAHELDKAASYVEEPIEGQLFS

VEGWVPVNRVEELKHDLADTEVHLAEISLNEGEEPPTYLENKGYSRIGEDLVHIYDTPSN

TDKDPSLWVLVSFAVFFAMIINDGGYGLLFLAGALYYRFKNGQLKKAGMRVWKLLVVLFG

SCVVWGLLTNSFFGVSIGPDNPLRKVSALHWLVEKKAEYHLKQKDEVYKDWVKKFPGIAN

AEDPQAFLLGAKKESNGKTAYEMIDKFSDGILMELALLVGIIHVCISFIRYLGRNWAGLG

WVIAIIGSYLYLPLFLGATSLATYGFGLNREEIAQGGLYMIYGGIAIAVILGIVKDKWLG

LLEVTNVIQIFADVLSYLRLYALGLAGAIIGQTVNDIAGSLMYLPALILIGIGHGLNMVL

AVVGGVIHGLRLNFIEWYHYSFEGGGKLFTPLKKLETD

>tr|D6YRZ1|D6YRZ1_WADCW 50S ribosomal protein L18 OS=Waddlia chondrophila (strain ATCC VR-1470 / WSU 86-1044) OX=716544 GN=rplR PE=3 SV=1

MKTVEYKQNQKRKRRVFRVRKQLRGTSLKPRLCVVKSNQNIEVQLIDDNKGITLASTSTR

SKDLKNTEFNKCNKESARKLGEIIAERAVKQNVKEVVFDRGAHKYHGILKELADAAREAG

LKF

>tr|D6YVE6|D6YVE6_WADCW Uncharacterized protein OS=Waddlia chondrophila (strain ATCC VR-1470 / WSU 86-1044) OX=716544 GN=wcw_0740 PE=4 SV=1

MMNIASDFSAFHDALINSPAKISTDNQGCWKEVPFLKRQLYSLECFEKRRLSSLAANMVK

EFDRMEKVPVKFNGISEQSEQFKKYFEAASYIKNQMKIFSSSKKIFAQCQLLKQRAAALK

YRIEQINGGLDKGEVSSEATDELKQLAVEWKGSCLLYQDESLSKSEVDKLREASRYPKFA

KLLKVDPEIRNQFFRWAIRDNNPVDTFVEFPSTCCRLKSALLSGRIGRFANNFRFLAKKN

GEKEFRLPFQVELEDGRLKTKQISILSENRLVKLRGGYQLTIREIFNTFKRKNLSPGQLE

CFGSCGISNWHAHELGWLNPKTDTCERIDLDQENSEWWKQMPVFEHLSDGEVCRRFGIDS

LQPGQWVAVAKSTRESLSLDIDRSHGYFEIVVPNKDEGGYDLYPLGKFAREFPKNIIEKF

LFVTNTVESRIEYPDENPFYSHRQHASAPFVLSEADGIALMEMIRKDLKAAREGNVVFQF

AWENCAWWPQERLENLLGKKEKEAEAKAPNLFVSPLLESRPEVQPLKGILKICRALPEKL

QMIAVKISAFILGSWRGVWVVEEGKRVFKSMKNSRFKKECKMYHPALLHERIQNGSVNGV

VTMGHHFLPQYS

>tr|D6YSF2|D6YSF2_WADCW 2-Hacid_dh_C domain-containing protein OS=Waddlia chondrophila (strain ATCC VR-1470 / WSU 86-1044) OX=716544 GN=wcw_1652 PE=4 SV=1

MRTNIEDRFMQIVLLQNRLTDGEVAELIDEFPQFLFLAAYEIPFNQLSKEEWERVEIIYG

NRLSKENLQFAHQLRWIHSPVPNLNPLCLDQLEKKGNVIVTVTREENAYQIGEYVMCGIL

AFAKNLFHWMKAGESPSLLWNSKWRESMWTLKNKIFIQIGLGKVGTEIARRARQLEMKVW

GVQEKRSFHPYCHKVFDMEELNAILPEGDVVCAALPRPQQKRTLFGKQEIKLMKEGSIFV

MIGSKSLIDEGSLVNEKDANKFRGILIDAFYQVPIPPTSKLWKLPNAVITPEVAPRPKSE

GKEAYSLFRYNLRQYTHGNFSDMRNAVGTKKTLIT

>tr|D6YUM8|D6YUM8_WADCW Uncharacterized protein OS=Waddlia chondrophila (strain ATCC VR-1470 / WSU 86-1044) OX=716544 GN=wcw_0467 PE=4 SV=1

MKIHLHRIYEEDGPHGYRVLVDRLWPRGISREKADLDAHWKELAPSDHLRKWFGHDPEKW

KGFRKEYLSELSLLKERAKELLKNIPGHQLVLLYAAKDKKHTHALILKEYLEKLE

>tr|D6YV40|D6YV40_WADCW Uncharacterized protein OS=Waddlia chondrophila (strain ATCC VR-1470 / WSU 86-1044) OX=716544 GN=wcw_0633 PE=4 SV=1

MIDFDNLPADITNHIMLFREFLNISWDHVIYKIMDEHNWDDDSDFIDDWMQINWELLVER

ELLENRFTLAQFSTTHLGENILNPGKLPDFMVVGKSDHLELKDIVSSTVISEDKILRLFT

FNTWSLKSKALAFGPPFEVAGLVDIETNEIFHVLVEHLSFWLYKIQTHTYMKGMCKFQKY

VLDNN

>tr|D6YRH9|D6YRH9_WADCW Multiple resistance and pH homeostasis system, subunit C OS=Waddlia chondrophila (strain ATCC VR-1470 / WSU 86-1044) OX=716544 GN=mrpC PE=4 SV=1

MKFLLAIVVGVLFGSGVFLLLRRSLFKLVIGLILLGHATNLLIFASGKLTLGRPPFVEQG

QNSLMLADPLPQALILTAIVISLGVTAFALVLAYKFYGVTKISDLDDLKEN

>tr|D6YWJ5|D6YWJ5_WADCW Uncharacterized protein OS=Waddlia chondrophila (strain ATCC VR-1470 / WSU 86-1044) OX=716544 GN=wcw_1149 PE=4 SV=1

MLKYFIFIRISIYLGFLNKHLELLMAIINDLAADFKAAYTGNSNFKNAGNLSTACQWGKV

ATVALIVATVASALFSGPVGLFVMGLTALALFDCYRMLDNVQHIADRNVGGRLLSEASDF

AQSIKPHEKIVRGTIFARPIANLIA

>tr|D6YVJ2|D6YVJ2_WADCW Putative cytochrome d ubiquinol oxidase subunit 2 OS=Waddlia chondrophila (strain ATCC VR-1470 / WSU 86-1044) OX=716544 GN=cydB PE=3 SV=1

MSEVFTLEFSWFAIFVVLLTGFAILDGFDLGVGIFHLFSKSDEERRLMLNTIGPVWDGNE

VWLVTAGGALFAGFPDVYATFCSAFYIPIMLLLSGLIFRAVAIEFRSKQPMAWWRWMWDL

LFSAASLVIALGLGIVVGNLIVGIPLDHQKEFIGSFESLLHPYALLVGVLVVALFSMHGV

IYVLMKTEGTLHDKMREWVNPAIILFIMLYAFTTVATLIYYPHMVEAIKERPFFLLVAFI

NMLAIANIPREIHHNRDGRAFLSSVLNIVCLMALFGIGTFPNAVRAVNDPVQLSMTIWNS

ASSHKTLAILHLIALIGIPMVLGYTIAIYYIFRGKVKLDSTSY

>tr|D6YWG1|D6YWG1_WADCW Putative hydroxymethylglutaryl-CoA synthase OS=Waddlia chondrophila (strain ATCC VR-1470 / WSU 86-1044) OX=716544 GN=wcw_1115 PE=3 SV=1

MSTDVKVGISDIAIYLPRLKIDLETIIKKRASESDGKILEKVLKRALEKTGVLSMHFPES

WEDSVTMAAQAALKLINRKRFNLKALRYLVSGTETTVDYSKPLSSYVIGILKKAGISLPQ

SISTFQTQHACAGGTVALMAISALLGFTQSSGESGIVLCSDIARYGKYTSAEMTQGAGAI

ALLTETDPKLIELDLKTAGYSSKDVDDFFRPLGSEIAKVKGGFSVRCYMEAMDSALVDLS

SRSGKTPKEMLTSADMYALHVPYPKLPTNTMEYLLGKYVEMDKEAIEKILKEKGFYEMTA

PASKSGNIYTGSMFMSLAFLLKNRYNQLGNAIVGKKILMGSYGSGNTMVFMSGIIAPSAP

EVIKNWDLEEIWDHQKSTIQDYESWIESNGKTSEQYASLLESKRSSIQPDMFFLDRIRED

GYREYSYSSQHS

>tr|D6YWU0|D6YWU0_WADCW Uncharacterized protein OS=Waddlia chondrophila (strain ATCC VR-1470 / WSU 86-1044) OX=716544 GN=wcw_1244 PE=4 SV=1

MLAINPVGSKHRVPFNEQTNQAYTHGLEQIYALETIDPSKIKKIELPKSVEKEMIPVGFS

HREDLWNGQYQLDLGDEYRGWMPPIFLKEPVQMLGLSQRAEMVCLDRGAERLMDLISLDF

NDWIGEKGVGQGHIDEIQQKLRQYIDGRSLERSFYIEWKALLRSLTVGMERKKVHLLLQS

FDLEWILPLTPVERMETLNLQDQTKKIWQNEALCALQCFEKTSYVKEMLRQISSVFLSPW

IRARGGVARLCEAAERTERVSQEREIVPRVMRFLQNIYGFKKHPLVEFLDEVEEELYCPD

RYTAEAYFAICQRAQSYFYHEGVIYLFKELTSLLAREFGAAWEGFPELLVEASLKKSCCF

QVRKGESGQLVIYRASCLSMQASHIPHALPAEGL

>tr|D6YW48|D6YW48_WADCW Dihydrolipoyl dehydrogenase OS=Waddlia chondrophila (strain ATCC VR-1470 / WSU 86-1044) OX=716544 GN=lpdA2 PE=3 SV=1

MEDLIVIGSGPAGYVAAIRASQLGLSVAIVEKYSTFGGTCLNVGCIPSKALLQSSEHFAF

IEKHADEHGIQLKNATVDFSKMMKRKEEVVASLVGGIEGLLKRNKVKTVQGSARFVSPEE

IEVNGKKMRAKHFLIATGSKPIELPFLPFDEKVVVSSTGALSLPSVPKKMVVVGAGVIGV

ELASVYSRLGSEVAIVEMLDRITPAMDNQIGKTFQKILSAQGLTFHLGAQVTDAKVSKKG

AELSVRIKGKESTLKADVVLVAIGRKPYSEGLGVEEIGIAKTKQGFIVVDGNFCTNHPNI

LAVGDIIDGPMLAHRASEEAVAAVESLAGKSSHINYMAIPNVIYTMPEVAAVGLTEEEGK

EMGLELITGVFPFKGNARARCAGETEGIVKVIGEKNSGRLLGVHLVGANASEMIGEGVVA

IEKRATVRELAYTSHAHPTLSEAIKEAALAACEKPIHL

>tr|D6YTA4|D6YTA4_WADCW Putative membrane protein OS=Waddlia chondrophila (strain ATCC VR-1470 / WSU 86-1044) OX=716544 GN=wcw_1966 PE=4 SV=1

MRGDWYQISLIFLAVIVTAFFGGFLYKELFPEYKIYQNTYIALEEFRSSYTGQPPPPFES

KIKQIVLEKPDNGPPVIDRCTSCHVAVQFSHFSPTQQAADINGNPVYDESGLPVLEPNPS

YIWTKLDQKIDTLKKEGNDKEAENLQALKTVKVGEHVYDMTKVLRAHPLIGRETRPFEYH

SIDEYGCTVCHSGNGRGLTTEKAHGPVFDGQYEAEYMGPEPEFLEKDPLNDPQFSKVFNH

KPGHELLFQTTPIYVGALLQANCVQCHNSTSEALEDAARETSIVEKRREEKSKIIKDAYE

NEKKALVSLIALKKELEKSGWNQTVEYLTQGTQDFRSSDEQIAAVKSQLEFVRKAENTEK

AISAIDERMEEILGSQKLVEQIAQNSGSIDTFLEKHSGSGTLFVKKSALKKDKVLMAYAE

SAQSTIRETVADENIRGVLKSEIDMLISDFHRGENLYFSQGCYACLRIDGLARGGVGPEL

TNIGNFYPWYIKESMVWPQADLKTSTMPNFHLDHEELEDLTTFLLAQKGRRPATSEPAYK

TRLMEWESGYKLPWEKPINPANLNNLRYSMTVFATEGCAACHRLKGFTSNIGFKIEKGKT

PSFETLFKEREWFRKTIAEEISGSELAKILDKKSDEIDSRIVNDVRSGSLLEELESTHPQ

LIESYNTAFKYAKRAKNHHYQEKLSQASDPSEKKRIKAEWDQWQQRVNRVLMMYVQEYGL

GRLVGPRPNWSGIYRSDEWLIEHFRKPSKHVARSIMPVLPFDDSKFYALTYMLDRIAVEN

RDEVRKIWDTFGFDPAMAYDLHCSQCHGDHKRGNGPVAEWIYPIPKNLTNADFLRNLTRD

NAVISITHGVLGGPMPPWGEVADGKKIIGSVPVLTESEIDQLVDWLYAPLEGASIYQTPE

EVDKWQYQPGDLLKELKKERGKLRPVAKEERVDEVFDVVSNPYKEGENNLYYIKKRYYTQ

HNLEEGREFFELNCAVCHGKEADGQGYRAGTMFDAKPRMLTNFEWINTRDDLRLLRSIKY

GVPGTSMTPWGDQTSALQRLQLVMYIRSLSNEQKQRDRLTTVIYEMYEEEGLVLEQSRTD

QYQRVNELKNAIKETKRALLTGTLSEAEAADAYKKELSLSQKMQMLQAVDQLVVDMREKL

QKESDILRNVGVQLLGIQEEKALFDQYLQLISLNSIHYSLADGQLKLSHDAEKELQIQTL

SKKMLQEVLETLQTLEKEKILVEGRLPSNERSESLEDLNGKILKYKLLQKALASGQEEAK

RLRDQQIELYANYEKKLKIWKKRNDIGNEIQ

>tr|D6YW10|D6YW10_WADCW Putative membrane protein OS=Waddlia chondrophila (strain ATCC VR-1470 / WSU 86-1044) OX=716544 GN=wcw_0961 PE=4 SV=1

MTISTANHAENTKVLDTLNAELSRMDQLLEDPNCDHARVLEQAFKVIQLMSRMLGNMEKE

YVLDQESVLFVKAKELKGTYNTWSVLALTIVSSSVTIGGGFVGMGAAIPGTTLGQTLGNA

SPNALGWLGNAAWAKKLGSIGQGMGAVGQGSGGFTSLMNNSNEAKRTVFQVVIEEVKRKR

GDRDDAARQMRDQSYSAANNCRQAIQAIHQAAQQILSARG

>tr|D6YSF3|D6YSF3_WADCW Uncharacterized protein OS=Waddlia chondrophila (strain ATCC VR-1470 / WSU 86-1044) OX=716544 GN=wcw_1653 PE=4 SV=1

MFPLDHAIFSDSDSIDLDFWDLGYTDDLLGRFQGPMEILQTHEQPRNLAEARYDLARKQY

SHLYQCRELISGYPDIKDILVVEMDYFEVVSVKEEKYLSTVGLGPCLGICARGWTEEGEC

FLGLAHLSIQSPRLCLEQLTDKLKGQGCLDHSIQFFVIGGMKSICEEEGSGSLESETEIL

ELSNEFNIQGVLFNLVEREGEPFLSVIISETCVYYTRDQSFAEKHYRISDTKLIGCSSSD

DEMSFESCSSSSKCSC

>tr|D6YWS5|D6YWS5_WADCW Uncharacterized protein OS=Waddlia chondrophila (strain ATCC VR-1470 / WSU 86-1044) OX=716544 GN=wcw_1229 PE=4 SV=1

MKIRIAERLRPFSHRPGTRLILPRSDHVLTLYPTLFKVNDRSIPLELSGPVKEFTVQLDL

EKGCVWVWGFYREGYRRFCLWSESGEFRMAGTRGGDQAAAPLFHSKPFERLSLGSHKKQD

WCLVKRRGDLKEIVPHWMRLAQLIPQVQPHQEGALGFMLQIQKNKPTLHDEIWQLYDVGF

DGIFHPRLEDEDHLGISLPPVSRKCSPLALIAEGAKFLRSLFIESGSQISILPCLPSAFH

CGRMVSIDLGTAGLLDMEWSKKMIKRMVFYSAVDQEQTFLFQKGIKNFRLKRESEKGEKI

ISTGSALSFAKDSTYYFDHFQK

>tr|D6YSB9|D6YSB9_WADCW Putative type III secretion translocase SctQ OS=Waddlia chondrophila (strain ATCC VR-1470 / WSU 86-1044) OX=716544 GN=sctQ1 PE=4 SV=1

MRKDPQPYAWVKKIPESIAKADTIPLLGDLPPFPWEDLAENLKNLLEFQSLEIVPSEFVW

AEEGAATKGMGSPLSIVKFSIASLEGNVYIAIPQEEIRKIMDLLLLKGSEPAPIDDDFQI

AFLDFLTLEVMHAVRQIDYPPNLLPRLEKNAEMPNTPMLVMNLTVTADSFVFSPRIMISE

EMNRSLKEHYVKSKENAVFKRPLSDQCDVIVHLEAGKTTLSQAQWKEVKPGDFILLDRCS

LKPGENSGTVTLTLKGHPILRGNIQDANIKILENPLFREDH

>tr|D6YW55|D6YW55_WADCW Membrane protein insertase YidC OS=Waddlia chondrophila (strain ATCC VR-1470 / WSU 86-1044) OX=716544 GN=yidC PE=3 SV=1

MDRRTIFFVLSLSLTLLAVNMFFEHRNRDHLKEWHAQEKAKKQQKMIELQAEVANKTVSF

QEMPTVSLASNDGKPLAKALRIGDAVITTRWTSQLPETVLVEDEELKLIFQSAEGPVVYA

KNDRQLLKTADLPYFGNYELQLVSFNPDQVLLGSYIDGAFSIPVVELQSLKADVEENVEE

VKLPHSAIALMKSGNAYLPVGLYLSKGNALIQLEQFKGLATETKKPMVPELQQEVEEAYY

VLENDYQQLVFSTTGGALVEINLPFETGENQKSVVKEIEFDKEIEEQHPYDARFPLQSYY

TPGESPTGPFVENSKGRLGGYYPLLRRDLIQKGAKPSIQVLPENYALNIVSKYPEIATLP

YRVTHFDKNKIVFEASQNNRKIIKTYSFDEQKNAPYIVNLSITVEGDSRGLWLTTGVPDV

EWISNAPAPALKYRLTRQGKVEVNNLDKPKDSLTMSTVVPDWICNSNGFFGIILDPLSPI

DDGLRVLHIDGEEMPSRLVLVGEEYDRFQASKLPGYLMMLPLNSKGGTMNFRIFAGPFGD

KTLTTVDHLYSNPETGYNPDYKGSQTFHGWFSFISAPFSKFLFVLMKFFHHLTGSWGISI

ILLTIALRVMLYPLNAWSTKSMLKMQQLQPEVQAIQEKHKKDPKKAQLEVTNLYMSRGVN

PISGCFPMLIQLPFLIGMFDLLKSTFELRGAPFIPGWIDNLAAPDVLFSWKTPIFFIGNQ

FHLLPILLGVVMFIQQRTMSNAPKDPNLMTEQQRQQRTMGTMMTAVFSVMFYHFPSGLNI

YWLSSMLLGILQQWWTQRKFKANPIEIQTVPLAKGKQKRKKR

>tr|D6YTW2|D6YTW2_WADCW Uncharacterized protein OS=Waddlia chondrophila (strain ATCC VR-1470 / WSU 86-1044) OX=716544 GN=wcw_0198 PE=4 SV=1

MIELTPITALMLYLCITLATLLGIWARYHLKSRKQKVIIVEQELLVCEYCHFAYLDQIGK

EVTSCPQCGSYNKKNQYGDS

>tr|D6YW67|D6YW67_WADCW Uncharacterized protein OS=Waddlia chondrophila (strain ATCC VR-1470 / WSU 86-1044) OX=716544 GN=wcw_1019 PE=4 SV=1

MQLSYEQLESLAFDAVSIAAPVARAKGAKAVADAKAASVAEKVEKAAKSTPVVGPTKQSL

PTKLDLDALSKAGQVHDRNGLTLAGRSLDKHGNRVGSPFPLATGNPQLKNIQGQFHLDDI

LTHPESKFINHGLKGFEIYSPDGRGVHFRADGRFRGFVE

>tr|D6YU68|D6YU68_WADCW Uncharacterized protein OS=Waddlia chondrophila (strain ATCC VR-1470 / WSU 86-1044) OX=716544 GN=wcw_0304 PE=4 SV=1

MSTAPVNLAQSINQSWVSIEDTFVLDCPVLPEKAPPNSLLLSELSYVGEPEVKSSNEVKK

MCIVTKKNQGTIVYFKKCSDAYPPILAQIESAGSASMRLTRGANAARVRPVVDAEGNVVG

AASYEILTFVSLSKESLSVGVMVRMGIIDELVARYVRMEDDLHGDQIGIAKDLGIVGLDY

DELWYSDITVEIKGARAINNGILAPLPKDLFPVTDKDIDAFPNIRDAQPCHWPTKIPNNL

AIWKRFPNRAEFVKLAEDSMAIRQKYYAFLREILIDPEDHLQVMIPSFSRDEEGVAMIRK

MQSCIEKRWNQQLLEGLIKNQGFRRFVIRNKTSILEILDHFKAYNCEFAENDATIDLVKV

EKRFQYIVKKCMGKDLTMVLYDLGCTLRVDRKQWETYHPSYLKLIDVCEQLESSDHSFPE

AFFLFECELCNIALMFRERMDEWNLLIKNITQVVENYRGLVTEKIGTPIHSILIDSSHQQ

VSFMDKLLDKEKCLAKALMELLENHAKHEEIVDEVQKVFNEYAPIGRSSSLGSVNFFTYT

RTRSDDIEQLLKDLKDRPGERAQLISGFFNEGKWNISGYVRSGSANVMLVRRLVEKALDD

FKNEITLEQLRKHELVEICFAIEQKMLDIEASAQKIATLFQKMMKKKATDS

>tr|D6YRJ8|D6YRJ8_WADCW Oligoendopeptidase F OS=Waddlia chondrophila (strain ATCC VR-1470 / WSU 86-1044) OX=716544 GN=pepF PE=3 SV=1

MTVAREQVLQTDCWNVEALYKNFSEWEKEFNARFAEKESPRWPELQQFRGKLSEGPHTVK

KCLELLFKTQRVIEKLYTYAHLRHDEEITEDAYKTAYQKMIGIYHQFAQEASWFEPELLA

LPDNILNEILNADEIAPFKFHLEKIVRIKDHTLTPENEMLLALSGKALQTAHKAFSAIND

ADFAFGAVKDKNGEDREITHANYGLYIREHDRILRENAFRSYHGKYQRYENTLAELLSGQ

TETHVFNARARNYSSSLEAALFPKNIDVNVYHSLIKAVNDNLGALHKYFALRKKVMKVDK

LHLYDMYVPLSDDVEISMDFDKAIDIVTESVSPLGTAYQNALRSGLTKERWCDRYENRNK

RSGAYSSGCYDSMPYILMNYKDKLRDVFTLAHEAGHSMHSLLSHQNQPYHYGDYPIFLAE

VASTFNEDLLNRLLLQQTDKKEEKIFLINQQIEDIRATLFRQTMFAEFELKIHELAETDT

PLTPTLLKKEYKELNDRYFGESTFVDEEISIEWARIPHFYYNFYVYQYATGISAALALSE

QVNAGSMEEREAYLSFLKGGCSRYPIELLKIAGVDMTTPKPVEAAIKKFSNLVEELDKLL

AL

>tr|D6YRV9|D6YRV9_WADCW Ferrochelatase OS=Waddlia chondrophila (strain ATCC VR-1470 / WSU 86-1044) OX=716544 GN=hemH PE=3 SV=1

MHARTGVLLVNLGTPDSQKPADVKKYLLQFLTDARVTDLPWFKRQLLVRGIIVPKRYKES

AKSYSRIWTEKGSPLLVLGKQVKALLQQSLGDHFQVELGMRYQNPSIESALLSLKQCFKL

VVVPLFPQYASATTGSVHEEVMRVIGKWNRIPELQLINSFPVHPKMIEAFCVRASNFQLD

SYDHILFSFHGLPEKQLRKADFYNHCLQSKECCATLNEKNGQCYSAQCYATAQAIVKKLN

LPSTRYTLCYQSRLGKDPWIKPYVSDIIKELAQKGAKKLLVFSPSFVCDCLETIDEIGNE

YLAEFKSYGGEALDLVPGLNDHPLWVETLKSLVS

>tr|D6YW52|D6YW52_WADCW Phosphatidylglycerol--prolipoprotein diacylglyceryl transferase OS=Waddlia chondrophila (strain ATCC VR-1470 / WSU 86-1044) OX=716544 GN=lgt PE=3 SV=1

MHLAWLHWDPPRFIFTLPFIHHPIAWYGVCFAIGFILGFFIILPMIRQKLSQTEKILPRD

IASWPHVVQSLRQVSGKKEHPLYPIYQKLSQKTRENLNRLKIKQEPVELTKNDILQAVNH

SYLAYGREKLEKLFGKGLLTLKELAVLLTDRLTWYIVIGTVVGARLGHVFFYEWPRYQQN

PLEIIKVWEGGLASHGGALGVILALFLYQRSIREKFPEFNFLCLLDILCVPTAMTAVWIR

IGNFMNQEIIGPVTTAPWAVVFAHPLEAEGGLPRHPTQLYEAFCYLITFIFLYTLWSRKR

NQLKPGVLIGLFMILVFGSRFFVEFVKVSKGLMIDESFLLTGQYLSLPFIILGIFLLFCG

KKLSKSSIF

>tr|D6YWX5|D6YWX5_WADCW Uncharacterized protein OS=Waddlia chondrophila (strain ATCC VR-1470 / WSU 86-1044) OX=716544 GN=wcw_1284 PE=4 SV=1

MYFKKLIFLSFSVLAFYSPQLQGEVLEVFISWNSYLCDDRCAELIKKRFESLKQVESIDV

NAKAGSAKIKWNPKSTFTYQAIKTQLQLVGVGLSELRVRVRGRGVPQGEGAALISLGDNT

RFNLISPIQVAPGEYAALPDGSQLSLAEGLKEKILSSAREKKVIVIEGPIYQGHRSPPLY

LIVSRLQIEKK

>tr|D6YTV2|D6YTV2_WADCW Putative alternative thymidylate synthase OS=Waddlia chondrophila (strain ATCC VR-1470 / WSU 86-1044) OX=716544 GN=thyX PE=4 SV=1

MLLEDYEDFTESQRKVLEKYVTNTSSHVFALRNLPEVIKGALFSRYSRSSLGLRSLLLKE

FVSNTDESGFNTIVSGAGSSEDSDAQVLAIKRAQNFYDRILDGYGDDSIGELGGAHLAIE

NISMLAAKVLEDSRIGGSPLEKSTRYIYFDQKVKGEYLFYREPIIMTSAYRDVYINTCNL

LFETYSKMIPPLTARLEEQTPHDPNTSKVAYAASLRAKVLDCLRGLLPAGTLTNMGLFGN

GRFYEQLIHKLHCSNLAELQEIGRSSFDELNKVIPSFIRRSDLSHHTHQNYAQYYEAMQM

EVAMVTAKNMIFSERSMDRGVRLIAHDKDSVIKVAAAMLYANSDRGLEDLYLYCQQLPME

DLERILDAGCSSRENRRHKSPRALEHAVFTFEILADFGVYRDLQRHRILTQERQLLSCDY

GYFTPPEISGMEFESDYHAAMQKAKETFDTIATELPEEAQYVVPMAYNIRWYYTMNLRAL

QWLCELRSQPAGHPNYRYVAQEMAKEVIRAFPAFERFLKFVDYDGHDLGRLSQEQKKIDK

QIFSSL

>tr|D6YS41|D6YS41_WADCW Uncharacterized protein OS=Waddlia chondrophila (strain ATCC VR-1470 / WSU 86-1044) OX=716544 GN=wcw_1537 PE=4 SV=1

MIIDEFKISDFSSHLLLIHCTPPKCFHLVCKKDAIRMVKKTPSSSAILAAPEGSFTGIEC

FSDYQSIQRI

>tr|D6YUX6|D6YUX6_WADCW Putative membrane protein OS=Waddlia chondrophila (strain ATCC VR-1470 / WSU 86-1044) OX=716544 GN=wcw_0567 PE=4 SV=1

MQLSEKDFIHETKSSEGWKVWLGLGIVALAFILLVFSSKWMLDQTHQKINKSPFLQVTNR

EFSLFLWQNPEFMRSNRKKKTGYLSGFHSYPKASPIPEQADEWVSAPPDVLFLYHTWKRL

LSSDRFSRSVSIVEFHEFLDDAKEWQPIYWKGASPEYTQLVSRLLQLDQIDIRDQLPEPV

LQAFIGWKNYYKEGKKINDSAYTSAYVASFLKKHPAYASNYWRPMYPDYLRSLDPSSRET

IPSSEIPSFLKAALYNSKDPSEKIPD

>tr|D6YUS0|D6YUS0_WADCW Uncharacterized protein OS=Waddlia chondrophila (strain ATCC VR-1470 / WSU 86-1044) OX=716544 GN=wcw_0510 PE=4 SV=1

MIESWKKGSSIFAVIRFNNQFLKKKSLPFQENLLSL

>tr|D6YW59|D6YW59_WADCW DNA gyrase subunit B OS=Waddlia chondrophila (strain ATCC VR-1470 / WSU 86-1044) OX=716544 GN=gyrB PE=3 SV=1

MSKEYNASSITVLEGLQAVRERPGMYVGDTSTSGLHQLVYEVVDNSIDEAMAGHCSEIYV

IINADGSITLEDNGRGIPIQKHEKESQKQGREVSALEVVMTTLHAGGKFDKETYKVSGGL

HGVGVSCVCALSSKMIVNVYKDGRVWEMHFSQGKVIKSLADMGATSKKGTRITFWPDETV

MTVTEFDYDILAKRLRELAFLNRGISIFFQDERHDDKDDVNFRYDGGLKSFVSYLNENKN

AIFPEPIYFYGTREGDDGPIDIEVAMQWNDGYNETIFSYVNNISTAHGGTHLTGFSTALT

RVLNNYIKTHNLLRSDKIGISGEDMREGLTAVISAKVANPQFEGQTKQRLGNSDVGSVVQ

QITGEELGIFLDEHPSIAKTISDKAILAAKAREAARKARELTLRKSALDSARLPGKLTDC

QERNPELCELYIVEGDSAGGSAKSGRDRRFQAILPIRGKILNVEKARLEKVLQNTEVGTI

ISALGCGIGTEGFDLGKLRYHKIIIMTDADVDGSHIRTLLLTFFYRHMPALVENNHIYIA

QPPLYLVTRKKSKQYIHSEKEMDEYLLKLGISDIVLKKETDEQPMDKESVEALMNVIVDL

ESFLTRIEKKGMSFREFLEAKNEEGQYPRFQIETVEGFRFAYSSDEFEALKTADEEAQKL

QHEQKLASMPEAEITDDMRVFKAKRLHFIELYEPENLEKLINRIKPFNFSFENYLLVNGP

LLTVRTENGEDVLCHTLREVIDLIRDNGRKGIEIQRYKGLGEMNADQLWETTMDPEKRTL

IKVTLPDAVAADHMFTMLMGDEVPPRRAFIEQHALSVKDLDI

>tr|D6YUE0|D6YUE0_WADCW Uncharacterized protein OS=Waddlia chondrophila (strain ATCC VR-1470 / WSU 86-1044) OX=716544 GN=wcw_0379 PE=4 SV=1

MRPNPLINICRPSTAFVRGLGRNQGLKKNGRA

>tr|D6YSD5|D6YSD5_WADCW Glutamate dehydrogenase OS=Waddlia chondrophila (strain ATCC VR-1470 / WSU 86-1044) OX=716544 GN=gdhB PE=3 SV=1

MTHPGKQAAINEQLSLAIQNETEKFREYYLWLEKAMPPAFFEDVSHENILLIVHNLIGFN

LQDYFTTIHLRGAAIVICLDSADADLRILKNYAMHGIKNYQAYVSTAPPPFPGVKAHLRI

AQTFFTQAIECIEESFPEKSKEELRALVKQRNPNVTDSEFEKIINSINIRFLRSLTIDRL

ILALDMFFRAKTRDNCQYEVRYNENWEEKGIASMQVVLAWRNTPKHNFLYRMARIIHRYN

LVMKKVNAAYIDPYSKRSILVMALGLHGSNGKAVWDVADIPDFLRELATTKYFASFDPID

SLLIKTGIIVGKLGNFLRSAVNFAHQALVHIDPNLYTLEHIELDLCRHPELTSQICRAFE

LKFDPDYCDYQKYLEVRDQCLVDIENLDTGHPGNDERRKNVLKQALNLIHYSLKTNYYRL

NYTAASFRLDPKYLDDIPFNRKEKFPELPYGIFYMKGMHFFGFHIRFKDLSRGGLRTVYT

KQPEHLFSERNTVFSECYNLAYTQQKKNKDIPEGGSKAIIFLQPFERMESEAAILKNELE

ESGIDPKEIENKLQSFRDEQSTEFLYQAQRAFIESLITIVNCDPSGEIRAKNIVDYWKRP

EYIYLGPDENMHDSMIQWISSFSQKYDYKPGSAFISGKPQVGINHKEYGVTSLGVNVYMH

KLLLHLGIDPEKDPFTIKISGGPDGDVAGNQIRNLHKHYPNTAKLVALTDVSGTISDPEG

LDLSILVELFHQCKPIKYYPPEKLHEGGFLVDKEAKKQQTAFVQQTLCWKMKNGSLIEEW

LSGSEMNHLFRNNVHQTKSDIFIPAGGRPRTLNHQNVKDFLDAEGNPTSKGIVEGANLYL

TSEARRFLEEKGVLIIKDSSANKTGVICSSFEVLCGLVLPDHLFLKWKSTLIEEILERLK

LCASNEADLLLNTLKTNRAFLTEISDKISARINQYTYELLDFLEEVPLSNHPSDPLIKYF

LDYALPSLVSEFQEELLEEVPDHHKKAIISSHIAAQLVYKKGLDWRPSIVDILPVILEKQ

I

>tr|D6YVB8|D6YVB8_WADCW Uncharacterized protein OS=Waddlia chondrophila (strain ATCC VR-1470 / WSU 86-1044) OX=716544 GN=wcw_0712 PE=4 SV=1

MGKMNYSKVEEALTEGLYKMKVNQILESSKNKDDPKALHPAQKFIAVVQELKWIKKTSPD

IYKKIKISKKEISSLAEIIKKRRDSLKESEVKRIDELLEIIKKYKKKEMPQIDDEDLIEE

ENDRHHYKRHNVSEKWIPLDVPRN

>tr|D6YWZ7|D6YWZ7_WADCW Uncharacterized protein OS=Waddlia chondrophila (strain ATCC VR-1470 / WSU 86-1044) OX=716544 GN=wcw_1306 PE=4 SV=1

MLRFFLCFFYPFVLFAKTIPITIETADTDEKRIWGLMGRDRLPKNHGMLFIYDKQRYLSV

WMFNTRIDLSAAFIDNKGIITEIRQLQAYPEIMDPDRPVLSLNDLRLYPPQDPIVLFFRK

KRATSLHQVRYVLEMQMGWFKKHGIRPGDRLQWNPGENKATIQLQKRTKRDILSPKGE

>tr|D6YTT0|D6YTT0_WADCW Sodium/proline symporter OS=Waddlia chondrophila (strain ATCC VR-1470 / WSU 86-1044) OX=716544 GN=opuE PE=3 SV=1

MKTQYLLSFITYFTILLGIGYISHRKQTSNAEFIVGNRSLNFWVTALSAHASDMSAWLFM

GLPMAIYFQGLSGSWIGIGLLIGMFLTWQLIAKKLRVSTEKYNSYTLSTFFEKRFEDTSG

TLRILTAIILLFFLTFYIGAGLTAMGLLIESLFNIDYWWGLTFALCVVVAYTFGGGFITI

AWTDFFQGLFLLFMISIVPILMLLKFPDIWTTIKQLDLLEPNYLSLMGDMSIGSVISSAF

AALAWGLGYFGMPHVITKFLGIKNADEMKKSRNVGMTWQFLALTASIAVGVIGFAHYQGT

LSNPELVYVEMVKLLFHPFFAGFVLCGVLAANLSTMDSQILVCASVIGEDLFKRIAKKEL

SPQALVRASRCGVIIIALFALIIAYDRQKTIIEAVFYAWAGLGSAFGPLVLMSLYDSKAN

KYGAVCGIIVGALFGSLWPFINPYLFAYQLPATIPGFSLGLLSIYGISRITNKQIIAQ

>tr|D6YUB3|D6YUB3_WADCW Ribosomal RNA small subunit methyltransferase H OS=Waddlia chondrophila (strain ATCC VR-1470 / WSU 86-1044) OX=716544 GN=mraW PE=3 SV=1

MIPHLSVLLEEVAESFSDVDLKRFVDGTLGAGGHSAEILSAHPEIQRFLGIDQDPDALEI

ARERLSKWSEKVMFQRGNFSDLKEHLAQNGLEKVDGILLDLGVSSMQFDRPERGFSLQQD

GPLDMRMNPEEPLTAAEIVNTWPEAELGDLFKKYGEEKRWRAAARAVAAARPISRTLELV

EVLTSVLGRPRKGKIHPATLIFQALRLAVNREIERIEEVIPQAIGCLNPGGRLAVISFHS

LEDRIVKRQFRYLADDKESTRGVGGVFISKEPVVNLITRKPIVPGKAEVERNPRSRSSKL

RVVEKR

>tr|D6YTC7|D6YTC7_WADCW Uncharacterized protein OS=Waddlia chondrophila (strain ATCC VR-1470 / WSU 86-1044) OX=716544 GN=wcw_0011 PE=4 SV=1

MSGQKEGVYKLTEWRSCNASFSELGGFASTSPVIFKKVKNIAKSNSEPKSLKKKNLVERI

RNTTFENISPISLYRTLHSLITKKQKDRQITHRLQCYSGAF

>tr|D6YU20|D6YU20_WADCW Uncharacterized protein OS=Waddlia chondrophila (strain ATCC VR-1470 / WSU 86-1044) OX=716544 GN=wcw_0256 PE=4 SV=1

MKIVKFAFILIFCAAATIRLEAATLHTILVGDTMDESIGDSTVMDLFKMRRQMEKVAKYS

GMEHHQVKISGYEVLADNILGALDLLQCGEDDVVVFYYSGHGYRTESKEGNPWPNLYFSI

ESKGIDLSHIRDLLEAKNPRFLIVIADVCNSFVPDEFAPPLMQKFWSCSAIEEIAAANYR

SLFLETSGTLMISSSEVGEYSWGTMKGGLFTVAFLQNLEKTAESDSYPEWELILDKTAQA

ISDVQHPQWDLNLRIGSKEDS

>tr|D6YUJ4|D6YUJ4_WADCW Putative peptidase OS=Waddlia chondrophila (strain ATCC VR-1470 / WSU 86-1044) OX=716544 GN=wcw_0433 PE=4 SV=1

MKTLDQLRHSYKQSEAKALEAFQKFLSFPSISSEAEYTPHLLECAGWLREKMNSLGLTTQ

LWKTSGHPILFGEDLSAGPSKPTLLIYNHYDVQPVDPLELWKSPPFEPAIRNGQIYARGA

QDNKGQCFYVMQALKLLREKNGGYPINIKWLIEGEEETGSEGLEGILAEKSQELKADYLA

IVDLGIPGPNIPAITLGTRGILTMDIVVKGSNTDLHSGVHGGIVFNPIHALVHILSSCRD

KEGKITVPGFYDSVSPINDREKERLFLDFDEKEYHQAFGALPTGGETRLTPFERAWTRPT

LEINGIHGGYTGDGFKTVIPAQASAKISCRLVPRQDPKQIGPLVAEYLKSLAPEGIEVEV

NVHPGSGSACRSDIRSKGVQAFSKAYEEVFGQPISYTYEGGSIPIINALSEASGSEVILL

GLGLADDYMHAPNEHFGIDRLEKGALIMALGILNI

>tr|D6YVQ8|D6YVQ8_WADCW Putative membrane protein OS=Waddlia chondrophila (strain ATCC VR-1470 / WSU 86-1044) OX=716544 GN=wcw_0853 PE=4 SV=1

MMKYQEIGREALKYAFNGLAGFGVGVLLGKALQGPSMIVGGILAIGFVALKALRTLVRNF

AIRQDMHLSNYQIVKNITGTVLNLAVNAALYAFGIFGTNSFMVISGASLAVCALNIGLGL

YIKYSEQDELMSDIVVGEKSPWYLHNAYA

>tr|D6YSV1|D6YSV1_WADCW Uncharacterized protein OS=Waddlia chondrophila (strain ATCC VR-1470 / WSU 86-1044) OX=716544 GN=wcw_1807 PE=4 SV=1

MKLDLIMGQGQYIHPVSFKKMTLKSFKEKKQ

>tr|D6YUA5|D6YUA5_WADCW Uncharacterized protein OS=Waddlia chondrophila (strain ATCC VR-1470 / WSU 86-1044) OX=716544 GN=wcw_0342 PE=4 SV=1

MSRIFFLFFLPAFCLGTEFQYWQIAYFNQKITDKWSFVFQTEERWKGVYSNLVRHQNDIG

IAYTFNDHWNIHVYYRHKVRNSDHGWATYPEQLIDFSGKWNVGEINISNRHRFVIHYYGN

HWVYRNRISLAYPIIPYCKKGDLYFSNEFFFRNLQKFFENRFGAGIQLSLSKTGNKVRCL

YLMQHINMATGWAHTLNILLIESKIYF

>tr|D6YWC0|D6YWC0_WADCW Uncharacterized protein OS=Waddlia chondrophila (strain ATCC VR-1470 / WSU 86-1044) OX=716544 GN=wcw_1072 PE=4 SV=1

MKTKCAAMFCFLIAVFQLSAFEAVNQPSLDAAKQWLELVDKEDYSKSWQTGSLTFKLTIS

EQHWNQLMKAIREPLGSVISREPLEQRPAANPKGLPLGEYMVVFFKTQFQKKENAHELVT

LVQESDGNWRVLTYQVQ

>tr|D6YWL6|D6YWL6_WADCW Galactosyl-1-phosphate transferase OS=Waddlia chondrophila (strain ATCC VR-1470 / WSU 86-1044) OX=716544 GN=wcaJ PE=3 SV=1

MEPSFSALKIRHLPVKRVFDLFFSLMALSFGLPFFLLIAVMIKCSSKGKIIYSQTRVGRG

GKTFHCYKFRTMYQDADFRLQTLLKQNPSARKEWGKTRKLKNDPRITRLGKFLRRTSLDE

LPQFYNVLKGDLSIVGPRPVVPEEINRFFGVKAHKVLSVRPGLTGIWQVSGRNNTSYSHR

VHLDETYVDTRSLWLDLKLICKTVPCMIFAKGAY

>tr|D6YVR2|D6YVR2_WADCW Ribosome-binding factor A OS=Waddlia chondrophila (strain ATCC VR-1470 / WSU 86-1044) OX=716544 GN=rbfA PE=3 SV=1

MTKQRIDRLNSLLKEVISEVIRKDVDNPEVGEFVSVTRVDISKDLRHAKVYISIIGDEDT

KQKTLKALTTASGFIGINASKKVVMRFFPALRFIIDDTVDKVMRIEELLQEIHEEQDSRI

PPKAADDTLF

>tr|D6YS74|D6YS74_WADCW Putative sensor histidine kinase OS=Waddlia chondrophila (strain ATCC VR-1470 / WSU 86-1044) OX=716544 GN=atoS PE=4 SV=1

MSTNESEEKAFQEAASLLIHRLRNPLGGIKGFASLLKRDLEGQPNLQKMAAMIVEGADNL

DRIMTAALDFAEPLNLDKEYCNLIPMIKEVFDHHQISWTTSIDTANVYMDKSQMRKALSY

LSDFAAHPQEKGSFPEAQVNVTDSNIAITLVDQGTVIPKDRVSKVFSPFFVAPWGGEEGA

LAQAKKIICSHGGTMEIDSSDNGTTIIAKIPR

>tr|D6YWB7|D6YWB7_WADCW Putative phospholipid N-methyltransferase OS=Waddlia chondrophila (strain ATCC VR-1470 / WSU 86-1044) OX=716544 GN=wcw_1069 PE=4 SV=1

MKQLVNVGKSRLKKMLPVDTALFLFQFFKHPNDIGALAPSSKALAESMTRFVRSDALAPR

RYLEVGAGTGAFTKTIAGKLGDQDQLDIVEINSRFCSRLEEKYADNPNIRIYQTSVLDWE

PESQYDVIVSSLPFNAFPSDFVKQIFDHYRKISKHGGIVTYCEYMALPGIRKLFISPKSR

RALQATLDMTNLFEKEYEIQTDKVFANFPPALVHHCKISSRGD

>tr|D6YTZ6|D6YTZ6_WADCW 2OG-FeII_Oxy_3 domain-containing protein OS=Waddlia chondrophila (strain ATCC VR-1470 / WSU 86-1044) OX=716544 GN=wcw_0232 PE=4 SV=1

MRFLNKKTIAAFSHEDFRRQWPFPYAGIKGILTEEGFAALIQEFPSLDLFAYHQDVKKAY

GQRPHNRYYLAYEETHFKRGDSYDREATDKDLPESWVSFIHELQKDADYQQLIKRALGVD

EWEVRFAWHVGQKGSEVSPHLDNADKLGTHIFYFNTEEDWKEEWGGQIVALGGMMTASGN

PEYEDFADQKEIPIINNRSFLFKNTLEAWHGVRPLRCPEGKYRRLFNVIYQSASSNRLRA

PL

>tr|D6YTF9|D6YTF9_WADCW Adenine DNA glycosylase OS=Waddlia chondrophila (strain ATCC VR-1470 / WSU 86-1044) OX=716544 GN=mutY PE=3 SV=1

MGSPCAGTSKTAPPSHRKSPCITQMILTLNCDSLKRWFLEYQRDLPWRDSRTPYAVWVSE

VMLQQTQVAVVIPYFLNWMEQFPDIPSLAKADQQEVIKAWEGLGYYSRARNLHAGAQYVL

EQFNGVLPSDPQLLSKIKGLGPYTIGAIRSFAFQQKTAAVDGNVLRVIARYRMLNEDISK

QKTVKNVNDWLEKQLPDQEHWLINEGLIELGATVCKRKPDCCLCPLRAGCQSYLHGRQHD

FPIKENRVSSTSLYRLVSVIHCNGNYLVKQAAKGEIMQGLYEFPYFETGPRGWSEKKALR

EIQESMGLKCQLIDCQLPIVKHTFTRYRASLTPFLFDTNHLKEVDRCQWLSANQLKNQAF

SSGHRKICDYILSA

>tr|D6YRR6|D6YRR6_WADCW Uncharacterized protein OS=Waddlia chondrophila (strain ATCC VR-1470 / WSU 86-1044) OX=716544 GN=wcw_1411 PE=4 SV=1

MVNFQNIQEALNFPLGQIKGAKVEVTIGDYLDVIDSSNAFTRLLTQVSLYIQGKGWITQA

TAEEMIKSVDLETLNAITNICKKVITRLSEVNLMKKDSIGFKLSKVDEQTVLKGIKSLGD

HHIDRSVQTVIQEMQLLRGWVGAERVNRHQHLVQLMGHWNVDHFEMIREDGKLKSIFLLF

VNWANQWGFEHPPGTLGEAVKYLNNNQSNKHYIDGNFDMSTFDTSQLATRASILTLISGF

KEWLAWSLETGKFNESKKQEIIDSLCKINPGNQDQIRKASADLPSSKD

>tr|D6YWL1|D6YWL1_WADCW dTTP/UTP pyrophosphatase OS=Waddlia chondrophila (strain ATCC VR-1470 / WSU 86-1044) OX=716544 GN=maf PE=3 SV=1

MIILGSQSPRRREIFNLFRIPHKAVTPHFDEASVPFTGDPEEYVCSIAKGKAESLARISP

ANLPILTADSIVFKEGKIFGKPANEENAFQMLQELSGSWHTVYTGLALYHQEKYQTMAEA

TEVLFNPLTSEQIRSYHRLVDWRDKAGAYAIQMGGGIAIRRITGCYYNVMGLPVNTMRNL

LNTIGIDLWDYLS

>tr|D6YSI3|D6YSI3_WADCW Tail-specific protease OS=Waddlia chondrophila (strain ATCC VR-1470 / WSU 86-1044) OX=716544 GN=tsp3 PE=3 SV=1

MRKFLFLALTLNLVFHSIDAKLPEIDSPKVLEKVNEIIKSHASHTKLTPEIMKRVISNYI

DELDLNKIYFIESDIDPWIHPSETLLETTLQQFNNGDFSLFFKIRDQMLKAISRRKLLDM

QVTASELPEEVDPKEFKDMDWVKSEEELVIRLGRIKALQIETSRKLNEELREKSLQRIEK

HQAKKEEEIANPDPKHSEQFVLSSILKATASALDTHTVYFTPGEATQFMINVQQRLFGIG

AQLRDDLNGFTIVKIIEGGPASESKQLKVKDRIVAVDDEPVVGMDILSAVELIRGKEGTP

VKLTVIREEGDDENKKKEELNITIIRGEVVLKETRFETAYEPFGDGVIAYLKLYSFYQDP

ESSSASDLAKEIEKLKENHRVKGIILDLRDNSGGLLSQAVSVTGLFITKGVVVGVKDNTG

NVQYLRDLDGKMMWDGPLAVLVNRASASASEIVAQTLQDYGRAIIIGDDHTYGKGSFQTF

TLNVGQEKPVNPEGEYKVTRGIYYTVSGKTPQLTGVLSDIVLPGPLSEIEIGEQYAKYPL

ENDSIKPNFDDDLSDIPYSQRSRIRLLYKHDLQKKLDTYTAYLPFLKSNSAYRIENNKNY

QALLKEIKKENLLEDESKEQFGQNDLQLTEAYNIMKDLIILMDTCKEDAI

>tr|D6YSI9|D6YSI9_WADCW Cytosine-specific methyltransferase OS=Waddlia chondrophila (strain ATCC VR-1470 / WSU 86-1044) OX=716544 GN=dcm PE=3 SV=1

MSSLCDDFLVKEELLADILSVSKRTLKKWKKEGVIVSVGNSDNGVYNLKDLKSFEPIKLM

LDSSWESETKTVPKRDFLSVELFAGAGGLALGLEKAGLCAQALNEIDKSSCMTLRKNRPS

WNVIEGDIKSIDFRQFKGIDFLSGGFPCQAFSYAGQKKGFEDARGTLFFDFARVIKETKP

KVFLGENVRGLLNHDNGNTISVIKSVIEDIGYTILDMKVLKSIFYQVPQKRERLFIVGVR

NDLVKKAKFKWPSPFKKVLTLKDALKQGSLYDSDVPTSNGQLYPQRKKEILSHVPQGGYW

KDLSEELQKEYMQNSFYLGGGKTGMARRLSWNEPSLTLTCSPSQKQTERCHPEETRPLTV

REYARIQTFPDDWEFSGSMINQYKQIGNAVPVNLSWAVGRSIVRLLNSLE

>tr|D6YTR4|D6YTR4_WADCW Uncharacterized protein OS=Waddlia chondrophila (strain ATCC VR-1470 / WSU 86-1044) OX=716544 GN=wcw_0150 PE=4 SV=1

MKRLYIGTAGWQYPEWKHSFYPRGMPTSQFLTYYSEIFDAVELNSSYYRLPRPETIIEWK

MRTPSDFKICPRLIQAITFKNKLKKGDNLLKAFLDHFLPLEERLGPVVIEIPENIYFTDE

NVEPFLDMLGRFSHHYTFVIEPRHVSWMDPNAGKMCEERELTWSISDFCGRIPTIHKTIG

SKIYLRFHGTHDLKNSEYTVDTLQYCAAKVQHWLEEGKTVYVFFNNHFGGFALENAKEFK

KLIQVPH

>tr|D6YSL3|D6YSL3_WADCW CaMKII_AD domain-containing protein OS=Waddlia chondrophila (strain ATCC VR-1470 / WSU 86-1044) OX=716544 GN=wcw_1714 PE=4 SV=1

MFAKYLSICAVLGALLFASSCDRKEESVAPKMQKEVEVIEGKSKMYIDAVNARDLDTIVD

QWSERAVYKNPYTEELVNGRKGIRSEFKKIFSNNNQAKIHVDEKTIRFPIEEKAVEEGVA

TIIVPGKNPVKRKYKMIYVNENGNWQILHTNRLGFGLED

>tr|D6YSX4|D6YSX4_WADCW Uncharacterized protein OS=Waddlia chondrophila (strain ATCC VR-1470 / WSU 86-1044) OX=716544 GN=wcw_1830 PE=4 SV=1

MTRNIPSEKKVYIGTGIRQGDIVSFVLIDQRAPNSPSVQIYKIEDDTLSGKWVHLGRTKK

GSETLKKIN

>tr|D6YSJ6|D6YSJ6_WADCW Ferric uptake regulator protein OS=Waddlia chondrophila (strain ATCC VR-1470 / WSU 86-1044) OX=716544 GN=fur PE=3 SV=1

MKRMTKQRMAILQCLSKAGRPLCVKEILTYATREIPQINLSTVYRTIKILIKEEKIDLIE

LPGEKSCYEIRQKNHHHYFLCNSCDKTYFINNCPKGLLEIVPRGFHLLGHSITLNGLCKE

CHSSSL

>tr|D6YWM6|D6YWM6_WADCW Beta sliding clamp OS=Waddlia chondrophila (strain ATCC VR-1470 / WSU 86-1044) OX=716544 GN=dnaN PE=3 SV=1

MKFVISTQEFNYLISKCQHVVPPNPTMPVLSNILIEAKNGELILTATDLTVGVRCFTEAK

VLEEGSTTLPVKKLASLIRELTSMNVEVTTSENHVTEIISDTSKFKLNGMAGGNYPELPS

FADATPIKLTQQQLKEMFFRTSFAVSKDDTRYALTGVFLHIENGRACFVGTDGKRLARTR

LDLEIDPSFSGGYIVPIKAVDEILKNLKEEGDATLYLLNDKIAVETENSLHVTKLLSGDY

PDVNRVIPDRSGQSLTLHREELLTLLRQVSLFTAEGYHSVRFSFGNGEVKLSANTKDVGE

GNVSMPVNYHADQIDIAFDPTFFMDILKHTRGERVTLGVTDSFNPGVITDTEEGTEDSPL

FVLMPMRLSENAG

>tr|D6YV76|D6YV76_WADCW Putative replicative DNA helicase OS=Waddlia chondrophila (strain ATCC VR-1470 / WSU 86-1044) OX=716544 GN=wcw_0669 PE=4 SV=1

MTISLSDLLTKEPPFYKRVIKRKNHYAEHNSDIPSRYNAIPTGFSDIDQLCGLVEHGVNV

IASVPAMGKTALSINIALNIASLKKSGLLKDINKIKYFNFESTKEQFILRLLSNKSDIES

DKIIKGILDKDEIIEIENAVEDLQALDFEVLQELPRNEEDVLEYLDQTCNEGDVIFLDYL

NLIVNNIRSEPYTSGVDFIRHLKLLAINRKVTFVILSQLNKKVDERPGHRPMMQDLPYGV

LSNHADLIMFLLRREYYDPMDKPGMAEIIIGKNRNGCLGCVNLVFRKEISQFCNYIPINT

FE

>tr|D6YVN2|D6YVN2_WADCW Uncharacterized protein OS=Waddlia chondrophila (strain ATCC VR-1470 / WSU 86-1044) OX=716544 GN=wcw_0827 PE=4 SV=1

MFNKIIFFIFILYQFNTFSQECNTPLLLLHLDVNKTLIAEDQTAGQSLEFMIASEIAEKT

VHQWEPNLPPMSYADYVRKILVPGATTKEIKEKRKKILRNFINILENSDHPDKNAIIAQY

YTCIEKMEGRYLIPSFVKFLCILKEQKAAFRIILRTYGNDIRLGKVTKEIESVLDGDRFF

YRGSFKKGTLKIKGMDSMRKAEEIYLFFRDTIGHVAIQDDWKTWLKDNKRRRSGKPFIFD

PADKEILSLFFDDNINSDPDSEFGIVNLLPINESEVSSSLLHKLYLRKVETIEAILDEDY

FLKLIKI

>tr|D6YVY9|D6YVY9_WADCW Putative membrane protein OS=Waddlia chondrophila (strain ATCC VR-1470 / WSU 86-1044) OX=716544 GN=ompA6 PE=4 SV=1

MTRRLLLSIFCALICTNPFLEAQCGKSWLSTSPCPPVTTHTYWDYSPCNTASNQWKSSPC

EYGCNNRCGFNAGIDYLYWTVCQDNLDFSVDHTIADDPPTLLGTGKTHFLKYGWSSGVRA

YLGLELLGFEIRGQYTWIENKAKETKDYTDTDAVLVASLLHPNTGLANARIAQGFQDLWY

ETADLLFSREMEFFDYKLILRPFIGAKWMRIKQKLKVTYEEEDFAENPEQIRWNSTLSGP

GAHAGFELFYRWVSGLGMYGQLAGSVIGAVVDNHHIQVSLDENGADVTPPRIQLKETQRL

CVPGYQLEAGITWDMCCLNNYNIRFRFCYELNQWLNTPEVRRYHYENEGVSSAATDGSIA

LHGATFGIDLRF

>tr|D6YTN1|D6YTN1_WADCW MotA_ExbB domain-containing protein OS=Waddlia chondrophila (strain ATCC VR-1470 / WSU 86-1044) OX=716544 GN=wcw_0117 PE=3 SV=1

MFVKNLIILGSTSPFLQAYQQSDLLGKGIYLAIVAASVICWTLMLYKTWQIREARKNSLR

FKKKVDLRKGNLLAQEAEGERKERLPNPYYEIYVILKKHTVDLLRKNHHVMGQKEGGSYL

SPTDIDCIEANLATAIASQTKEMDRHLFILSTIVGLAPLMGLLGTVWGILISFSQLQSQA

AAASNQMVLGGIALALTTTVLGLLTAIPALIAYNYLKSSIRDFETDMEGFTNEMLSSVEM

RYRKVDIKG

>tr|D6YWS8|D6YWS8_WADCW Putative membrane protein OS=Waddlia chondrophila (strain ATCC VR-1470 / WSU 86-1044) OX=716544 GN=wcw_1232 PE=4 SV=1

MSDYIGYEVAANVLEAGFAASSGALAVLALGSCVDPAVGAFGAVVGWLAGKPVQYLTTKI

FNTDNPAATMISRIAQFACSFFANAAIFMWSVGRLGVSLSFAASCGLAGTAIGIQMAFIL

AVSTFATLWLKGSQV

>tr|D6YS28|D6YS28_WADCW Putative phosphoglycerate transporter, major facilitator superfamily OS=Waddlia chondrophila (strain ATCC VR-1470 / WSU 86-1044) OX=716544 GN=glpT PE=4 SV=1

MNSILSLFKPAPYIDEIKDPEVVKTKYNYWRIRIIYSMFIGYALYYFTRKSFTFAMPGLI

EDLGYDKSQLGLLASILSITYGVSKFASGILSDRSNPRYFMAFGLFMTGVFNICFGLSSS

LLMFAVFWGLNGWFQGFGWPPCARFLTHWYSQSERGVWWSSWNVSHNVGAFLIPWVVGAC

LYFGWRYAMFLPGVLCIFGSFFLLDRLRDTPQSLGLPSIEKFRNDYPPNAKKDGAEENLS

TKEILVDYIFKNKYIWLLAAAYFFVYAVRTAVNDWTALYLLETKGYSRIGANGCVSMFEV

GGFFGSLFAGWSSDRLFAARRGPVNVLFTLGMLLSLSFFWVIPEGFPLLDSMAMFFVGFS

IFGPQMLIGVAAAELSHKKAAATSTGFIGCFAYMGAAVAGYPFGKLTQEHGWEGFFIGMV

VCALMAALLLLPLWGVFKYEVSVEPEPATA

>tr|D6YTY8|D6YTY8_WADCW J domain-containing protein OS=Waddlia chondrophila (strain ATCC VR-1470 / WSU 86-1044) OX=716544 GN=wcw_0224 PE=4 SV=1

MNIEFKKNAYLESIKKEDYLNSEEKIFDYAKNNLQFNYGNVGFREFNDTYASVEGWLGRK

VIALPVFFCLGVVKTINHFATIILQGIVYGTLAGAIRVGAIKIELNNQKVAPSLLTSLFL

SVVFERFKKESFYLVRDLQEAFGWLATIFSDRYGQYHVQESQFHKSCYGCESADDSSENY

SVLGLKAQDLKGLDSETLEKKVNHEYRRHSLKHHPDKIYRKSDESKADFEKRKQEQVERF

IKLQDARDGILNQKETSKDVNYNISFFGRSVMSSAT

>tr|D6YWC9|D6YWC9_WADCW Uncharacterized protein OS=Waddlia chondrophila (strain ATCC VR-1470 / WSU 86-1044) OX=716544 GN=wcw_1083 PE=4 SV=1

MRKILSFFLSIQLLCASQIFAQMTDNSKFYPNPGIVAQRGGSWVGSDHLYNLTSNIDILV

EIFKPKNIDIPISEEIIRSKVADIFTKGGITPTAEEVPGEPPLPFFHLLIMIYPIEKGYV

AYCEGRLFEKVSLERIKLDDQTVLQGVTWESQNLIVSPSDQIAEQIIKSVNEIAETFVER

YRFYDEIRRKIQKN

>tr|D6YSB2|D6YSB2_WADCW Putative needle chaperone SctE OS=Waddlia chondrophila (strain ATCC VR-1470 / WSU 86-1044) OX=716544 GN=sctE PE=4 SV=1

MFGLEGHKKGKKREFEFELEKELLDPKKHKELKQHVENRIQEIKKILRDGENKKEFERFG

LILHGYTSLLKVFSRFDPK

>tr|D6YSK3|D6YSK3_WADCW Probable GTP-binding protein EngB OS=Waddlia chondrophila (strain ATCC VR-1470 / WSU 86-1044) OX=716544 GN=engB PE=3 SV=1

MNYREAVFVKTSVSGSDYPRLLDDQGNPLPEIAVLGRSNVGKSSLLNHLFQRKGLVKASS

KPGKTAALNFFSIENFCAFVDFPGYGFADVPLHVKKQWGPMVQSYLENRKELKLVLFLFD

IRRTPNEEDLALVQWLLNEQVPTILVLTKSDKLNQSEKHQNTKKILEAFGFEKFSYLLYS

VPKNRGRNTLMKLIEETL

>tr|D6YSH6|D6YSH6_WADCW Uncharacterized protein OS=Waddlia chondrophila (strain ATCC VR-1470 / WSU 86-1044) OX=716544 GN=wcw_1677 PE=4 SV=1

MPFCRFVWRTIFFSGKKPLKKIEFQSVQNGNSRLIEST

>tr|D6YWG5|D6YWG5_WADCW Putative phosphomevalonate kinase OS=Waddlia chondrophila (strain ATCC VR-1470 / WSU 86-1044) OX=716544 GN=mvaK2 PE=4 SV=1

MRITVPGNMLLFGEYAITYPDGLGIAAATKEKLIIDILPASQLTISGEYGNETYRWTKGD

PFPSRLIEHIVSTLNLSPSLHIHLDARQFYYPDGTKKGYGSSAAVTIGLLSALLEGQIGS

IDQLAWHALQLHRSFQGGRGSGYDIYASCLGGVGLFKNSNPPEWQPLNPDLYREIYLLRG

AYSCDTRSVLKELKHFETAHPLQIKKYIKTSNALIQQLSLSLRLFHRASLLNRWINRHLA

QKIQWEEISVKPLGAGGEIGAALTPMEHSKPLHISLEGVQCLQ

>tr|D6YSI5|D6YSI5_WADCW Uncharacterized protein OS=Waddlia chondrophila (strain ATCC VR-1470 / WSU 86-1044) OX=716544 GN=wcw_1686 PE=4 SV=1

MMKTLQADTVVDLDRHLASSQLVIVTSPLFSGSLRIPLKLESADNLTIVSIGNGSRGACL

DYFTCFHEITSNFLTIVKCRSGYNVLCCLSHSGVAASLEGDGKDCLLCLRGVSAEPVPVL

IGVHGSDLHQTVGLAIRMGVKKALRAGKFGEEKPPIPHWLKTLGWESGASFGRIPTHDKI

LGAVWALRQEGIQPGYVLIDEGWQRVERRGGKKVLSCFEADAERFPMGLSGLVQELQRAG

VHHVGVAHSIFGCGGGISDSLVGKYQLSTKENEKGYLGYDLGKTFQFYHDYYKSLSEEGI

AFVKVKRQVDAAGFIGNPGLMTRIYSHLQSAIQASSGLFFEASHLNSECLNNESLISGIA

ATDDDLETAQTLAGVKKMIRSLLVNACWMQNFFSSWITDFPYSHLLAILHALSSTAHVIG

DPPGKTKIELLKKCVLPSGRLIQADYPLTLCSSSFFLNPLTTHALYCAFSFKGESGLLAL

FNFTRKKKPLQEDVSASLIEGISGDRFAVYSHTNGYLGVFEKNEEFSVAVKQNEADILTF

APVRNGVALIGCYAFYVPNGPIQEITIEQESMHISSIVTSPMLMYSEKSVMEIRRNGKVI

PWDYDQEKKLLVIDSRQSQSEIPTVYTLNFE

>tr|D6YWK5|D6YWK5_WADCW Uncharacterized protein OS=Waddlia chondrophila (strain ATCC VR-1470 / WSU 86-1044) OX=716544 GN=wcw_1159 PE=4 SV=1

MKNPSAIDTFEMKSKEKVFYSHILVVLNLRKNTFYFIKKN

>tr|D6YWQ3|D6YWQ3_WADCW mRNA interferase OS=Waddlia chondrophila (strain ATCC VR-1470 / WSU 86-1044) OX=716544 GN=mazF PE=3 SV=1

MALKKNHPLQGEIWLFDPDPVKGNEIGKKVRPALVVSNNLMNKGASGLIIVVPITSKDKK

IPSHIRIDPPEGGVNLSSFAVCEQVRSISKNRLVKRLGKIQSVTVLKEVGSWLNDLLWMD

I

>tr|D6YWU8|D6YWU8_WADCW Uncharacterized protein OS=Waddlia chondrophila (strain ATCC VR-1470 / WSU 86-1044) OX=716544 GN=wcw_1252 PE=4 SV=1

MRSTVSTLLDSHHILHPKEENHEIGARNEPTIFRRILGVVLVALSIPFTLGIATGFYIYW

AHRKVKHIEERDYSELQARTNKVFGEMIQDEAPSSERVQFEAALKAIQQGGKIQQFPEEY

DTRAIALLDAALAYDNTSAVTALMLKYKDSVGDIHLANVKSVKMLHAILEGFLKGRNGKI

C

>tr|D6YW90|D6YW90_WADCW DNA polymerase III, chi subunit OS=Waddlia chondrophila (strain ATCC VR-1470 / WSU 86-1044) OX=716544 GN=wcw_1042 PE=4 SV=1

MRFFKMNIRFLKTTDNVTKLRRISASVEHYFLKKKRVLITVPSETAATYLDDFLWKQPKE

GFLPHSVSTQACSDEIVITTKQENLNQAEILINLCSEFSPIAGQFKTVFELWDETNALRR

EQSEKKFKAYEASGMKVELFRC

>tr|D6YVK6|D6YVK6_WADCW Conserved putative membrane-associated protein OS=Waddlia chondrophila (strain ATCC VR-1470 / WSU 86-1044) OX=716544 GN=wcw_0800 PE=4 SV=1

MNIGKLIHFFIAAMLSIFFILGGITCLLTPWSSAIRTHIILFIIENTTLISSFGLLFIIL

GISLFINVIISSQHRYYSIKSKKNAIDVDENLIQQLVDCYFKELFPEKEIPSAISLKNNK

IHITVSLPYFPSFEQKFFLESIEHDLAETLGNILGLKEEFYLSASFKT

>tr|D6YTY3|D6YTY3_WADCW Uncharacterized protein OS=Waddlia chondrophila (strain ATCC VR-1470 / WSU 86-1044) OX=716544 GN=wcw_0219 PE=4 SV=1

MNFNLMKKTFIPKEFRPSVPGSSLKSLNRSVFEFPRNSENRPEKYVILSSQGDLVAKKVW

ESLKSNKTAIHFYLDELGYTWDFSYSNERLTLLTPSGKITPLSIYHRHPGVAQDHPFYSK

HISLFEVMDIWKGNSIGQKRDHFHNTSKPYQQGNFMKNT

>tr|D6YW13|D6YW13_WADCW Uncharacterized protein OS=Waddlia chondrophila (strain ATCC VR-1470 / WSU 86-1044) OX=716544 GN=wcw_0964 PE=4 SV=1

MSDSAIKDVLLDAVNFDKDGIDLENLSLEALILLINTERLKQLQEKTEKEFKELRERQEK

VKELHDILKAINAATKDDGTLDISKNQELKTKIERAKELGADIKEGQTTFNKEERDRIVE

NIRMTIEDMNVQNDMQLQTISRLTNERYESYQMARSILKPLHEAKSSLARGIRGAS

>tr|D6YS49|D6YS49_WADCW Nucleoside diphosphate kinase OS=Waddlia chondrophila (strain ATCC VR-1470 / WSU 86-1044) OX=716544 GN=ndk PE=3 SV=1

MLKKLIFTAAILFAPLFAQVEQTLSIIKPDAVQGHHIGEIIQIFEGNGLQVAAIKMVRMS

KNDAMEFYEVHKDRPFYEQLTNFMHAGPVVAMVLEGENAVAKNRQLMGETNPENAKPGTI

RYRFAKSVQSNAVHGSDSLENAKKEIAFFFNRQEIHTR

>tr|D6YTF5|D6YTF5_WADCW Putative peroxiredoxin 1 OS=Waddlia chondrophila (strain ATCC VR-1470 / WSU 86-1044) OX=716544 GN=wcw_0039 PE=4 SV=1

MAEEEQAGKQKEIQIGDKIPPFLAKDHEGFDVTDEDVIGTPLVIYFYPKDGTSICTDEAC

SFRDSMAVFDEKQALVIGISPDGVDSHKKFLTENKLEFSLLSDEKKDMFRSFGALKGDEI

IRTTFVVNSEGEVKWMEKPVDVKGHVERVIKALEEHCSDEVVSFDDYDRDYEEFMGKALG

DAPDEEQIRKDILKKFNLEESDLKE

>tr|D6YWR8|D6YWR8_WADCW MlaD domain-containing protein OS=Waddlia chondrophila (strain ATCC VR-1470 / WSU 86-1044) OX=716544 GN=wcw_1222 PE=4 SV=1

MQDQMKNMLIAIFIITALAIIVFMVIFLHPYTGDEGQILHVRFTDIDKVSVGTRVTFAGK

PVGEVTKISVLPEVRLGRTEKKGDVYVYELTLAVDSNLKVYNSDKVSLRTSGLLGEKSIS

IDPEPIKPGEKLRLVNDEIIYAEGTGSVEDTFNEFKEVADKFDKVLELITEALQELKDRG

MWENIANTADNISEITARLSERWNDVDDTIVSLSEAASNTKNITERIHTGEGTLGRIVSS

DDLYLRTNSILSKGETAMDDINHYGILFHLDKGWQRLRARRLNLMQKLRTPQEFRNFFND

EINQISTSISRVSMVLDKSECYPCLMDDCEYQKVFAELMRRVESLEENVKMFNIQTVDCE

VKKTELSECYY

>tr|D6YSU7|D6YSU7_WADCW Uncharacterized protein OS=Waddlia chondrophila (strain ATCC VR-1470 / WSU 86-1044) OX=716544 GN=wcw_1803 PE=4 SV=1

MKTILKATAITAQSEKIEQSSKLKAVNNIANQFLSSLQYKSILFVFLLLIGCGERVSEDQ

KISPLEKKIFSSIERGKLGQSTSPIIITPRKLSISEIGHHLDMDEETVLKHLKATNIHGY

YSLSARNLPPEGIFTLYHINFEGKMLQDKKFFVNGNGILVTKLDDQFIEINNNLLFFSNY

LPGEPVNFALVSEDQKLIATTKIVPNPIEKVDNHKHRISLEIASPDKRTYVIHCSGLRPF

GTYLASISFENERFAYPFEANSKGEAFLRTGPSAPWITEGEGTLELRGDQITNPLFLEFF

WGS

>tr|D6YVI1|D6YVI1_WADCW Conserved putative membrane-associated protein OS=Waddlia chondrophila (strain ATCC VR-1470 / WSU 86-1044) OX=716544 GN=wcw_0775 PE=4 SV=1

MAQHLLFYDGKCGMCDHLVQHVLKADRRGLFLFAPLDGKTADKVLKEIPNEIKQADSLIL

IENFQQRDSSLWMYGRGALKVAWLLGGWWVLLGWISFLPPFLYDWGYRLVAKNRRKLFSK

NSCRLPKSFEKERFLP

>tr|D6YSZ9|D6YSZ9_WADCW Trk system potassium uptake protein TrkA OS=Waddlia chondrophila (strain ATCC VR-1470 / WSU 86-1044) OX=716544 GN=trkA PE=4 SV=1

MNIVILGAGGVGRYIAQVLSKEEHNVILIDPNEKKLEKASWEMDVAIRQGSGTDWQLLDD

LLDLSPDFLIALTSQDEVNLTACAIAKHLGYPRTIARVRDNRYLNRTRLDFSHIFEVDYF

IGPELLVANDILKYMVSPGSLAVENFAHGAVQLRTLAIPEKWRKSDVPLQHLTLPKGVII

GLIRRDIPDAEEKGAKEVIFPHGQDVIKPGDEVTFIGETEAISELHRFLGIKQQRISSVV

IIGGSLTGLNLARLLEGREVDVRLIEKDYDRCRYLAEKLPNTTIMNHQLNDIDYFKSEKI

GTAEVFVACTESDEMNMIAALLGKEVGCDDVVVTLSNTNYAPIANQLGINHALSPRISTA

DHILSHILGGTVTSLVSFYDNQAEILEINVSMESKLVGIPLSELGSLLPKDFLIAMIQNR

GRIMVANGNRIISPGDTVIVITSPKHIPELEKIF

>tr|D6YVQ4|D6YVQ4_WADCW Type III secretion inner membrane protein SctV OS=Waddlia chondrophila (strain ATCC VR-1470 / WSU 86-1044) OX=716544 GN=sctV PE=3 SV=1

MNFITNILDGITQRLGGEKSLSNISKGSDIALALLIVGILGMIILPIGPDVIDYLIAVNL

SASVVLLMVSLYIPSAVHLSIFPSLLLITTLYRLGTNIASTRQILLNANAGEIIFSFGNF

VVGGNFVVGGIIFIIITLVNFIVITKGAERVAEVAARFTLDAMPGKQMSIDADMRAGILD

SNEARAKRLAIQKESQLYGAMDGAMKFVKGDAIAGIVITLINVIGGMTIGMAINGMPAQD

AISTYAILSIGDGLVSQIPALLISITAGIVTTRVSSEKEANLGKEISGQLLKQPKALMIA

AGFCMGLAILPGFPKAPFMILALTFGIMGYALWSGEAGRAAGGSSGGGGGGAMAGGISSA

AEEESSTIETAVKGHSAITGGGVDSYSLTLPVILECGNSLSSKIQKAQRGKSFIDVMIPR

MRQALYADLGVRFPGVHVRTDSPILEPDEYSIHLNEVPIIRGKVLAGHVLTNETEENLNR

YNLSFTAYKNSLGMPSLWVEDKNKQILEKAGIKSWDSLEVMILHLSYFFRHYANEFVGIQ

EAKSMLEFMEKSFPDLVKEVTRLIPLQKMTDIFKRLIQEQISVKDLRTILESLSEWAQTE

KDTVLLTEYVRSSLKRYISYKFSQGQSVLSVYILDPEIEDMVRGAIKQTSAGSYLALDPD

SVQLILQGIRNTVAPPPPGGQEPVLLTAIDVRRFVRKLIEMEFPDVSVVSYQEIVPEIRI

QPLGRIQLS

>tr|D6YRL1|D6YRL1_WADCW Uncharacterized protein OS=Waddlia chondrophila (strain ATCC VR-1470 / WSU 86-1044) OX=716544 GN=wcw_1355 PE=4 SV=1

MEKKHPLWNKPADIVALAEKPEQPPLPKGGIPKQWVPAWIRWPIRVLFLPFVLLDLFMQK

IAGYFFKTPYKQEGKCYQRGNCCFYIIIPAPDSFLTKIYYFWNTQINGFYPRHPQPVEVE

GEKVMVMGCRYLRKNGTCGHYRLRPTVCRQWPFIEYFGVPRILKGCGYHAVPRDKNFDPY

PEETNSTKNKLNIIK

>tr|D6YUB2|D6YUB2_WADCW Uncharacterized protein OS=Waddlia chondrophila (strain ATCC VR-1470 / WSU 86-1044) OX=716544 GN=wcw_0349 PE=4 SV=1

MATIDQLDISIYNLYAIRTRMMEQINQDLRLGEAGSIPPQTQLIDTMPKMTELDILLGVA

PVVTPWAYFYPPKRYRFIRRSPFAFFRIVPSLGSLEEHTEHEEKAMSVSTNSPEEEKEKG

AILALLDQIDKINNWMSFIVGRVGQFLQG

>tr|D6YX24|D6YX24_WADCW CbiA domain-containing protein OS=Waddlia chondrophila (strain ATCC VR-1470 / WSU 86-1044) OX=716544 GN=wcw_p0019 PE=4 SV=1

MKTIISCLNLKGGCGKSSTIVNLGGVFSENKKKPLLIDLDEQQSSTHWARQGGDKFPFPV

IPLSIETAGKVKSEIERLANEHKADTILIDTPPQLEDDALITALLSDIVLIPVSPSPLDL

WAAQQAVNTIKDAREERGGKLPKAILIPSRVMNRTTLAKDIKGSLKAFGEPISPAISLRV

AIAEAAIAGLPIGHYAPNSPGHKEFTNLWKFVNSQIRK

>tr|D6YV52|D6YV52_WADCW Uncharacterized protein OS=Waddlia chondrophila (strain ATCC VR-1470 / WSU 86-1044) OX=716544 GN=wcw_0645 PE=3 SV=1

MLEIKTDCAPKALGPYSQGIKVGNFLFVSGQVPLDPQTGNLVEGGIAKQVTQVIDNLESV

LKAGGADLSKVVRVDIFLTDLSNDFPIVNEIYSSRFIGSVKPARQTVEVGKLPAEALVEM

SCVAYSEEA

>tr|D6YVP2|D6YVP2_WADCW Uncharacterized protein OS=Waddlia chondrophila (strain ATCC VR-1470 / WSU 86-1044) OX=716544 GN=wcw_0837 PE=4 SV=1

MGDILSIEGAMVKHIDKDIREKLAKKHACYVLVTCGQPSEEGKINVEMSYEGDPALASFL

LQGAQEYLMEQDSD

>tr|D6YUW7|D6YUW7_WADCW Putative drug resistance permease, major facilitator superfamily, Bcr/CflA subfamily OS=Waddlia chondrophila (strain ATCC VR-1470 / WSU 86-1044) OX=716544 GN=wcw_0558 PE=3 SV=1

MKKIAISLCLIIGLVSLPQISETIYTPALPDVAKGLKASVHAVEASLSVYFLGFAFGVFL

WGGCSDLIGRRKTLLIGLTIYIISCVACLISHRIDLLLVWRFFQAVGASVGSVITQTILR

DVYEGKERSRLFATISAALSLSPAIGPVIGGLLSEDWGWRSNFAFLIALGSVLLAWAYLK

LPETRPSHVELPGKREYQNLCRRMFSSTALWGHVLLIGATNGILFCFYQEAPFIFINQIG

LSPKAYGLLGLVVASATVFSARLTYRLAGKYSQECLISSGAFTATIGAIALILLQQAGYF

QIEGLTSVVFALFVTFVGVGMIIPNSLSIALKDYQAMVGTAGSIFGALYYLIISLATWGM

SILHDGSALPLPLYLTLLCLLLLIGSFFIQQTLTVEEVIDQPEQSA

>tr|D6YUF3|D6YUF3_WADCW Uncharacterized protein OS=Waddlia chondrophila (strain ATCC VR-1470 / WSU 86-1044) OX=716544 GN=wcw_0392 PE=4 SV=1

MNKFKKVKFPRRIFVIVTKKFYNRFIKTAITT

>tr|D6YU95|D6YU95_WADCW Ribonucleoside triphosphate reductase OS=Waddlia chondrophila (strain ATCC VR-1470 / WSU 86-1044) OX=716544 GN=nrdD PE=4 SV=1

MVKPTARAMATSLRTYHRPIKEGESVLESWDEVVGRVISHQRWLWERALGRTLSERESHE

LEECRQLITNRQMAPAGRTLWLGGTELSRKRESSMFNCSFTFVETVYDLVDVLWLLLQGC

GVGFKPITGTLNGFRRPLQEIKVIRSNRAGKGGVVHNVETYDPETHTWTIKVGDSAIAWA

KAIGKLVAGKFPAKSLILDFSEIRPAGTRLKGYGWISSGDEQIAKAFKAIAQILSDRSDQ

LLTRMDILDIVNWLGTILSSRRSAQIALFEYGQPEWEDFSLAKKEWWITGNAHRQQSNNS

LLFRQKPTREELENVFEIMLDAGGSEPGIINAEEAERRAPWFKGCNPCVEILLGNKSFCN

LTEVNVLSFKGDKVGLERALFLAGRMNYRQTMVDLRDEILQEAWHLNNEFLHLCGVGLTG

IRARSDLTAYDYKRMRNITVSAAYSMANELDSPLPKNVTCIKPSGTLSKIMGTEDWGEVP

EGVHMPLGKYLFNNITYSRHDPLVGKFRVAGYSVIEKPYEPESVLVKFPVKFESVPFARK

TVTRKNGKVEEVDVNADTAVEQLEWYKLLQETWCEQNVSNTISYDPSEVPQIIDWFMENW

DSYVGASFIFRNDPTKNAEDLGYAYLPQEVVTKEAYEEYVSQLKEIDYSGLEMRDEELQE

EMCLNGACPVR

>tr|D6YSZ1|D6YSZ1_WADCW Uncharacterized protein OS=Waddlia chondrophila (strain ATCC VR-1470 / WSU 86-1044) OX=716544 GN=ybbC PE=4 SV=1

MDSKLKQLLFVFFTCFLLPLSAKVSLGIDQLVSGKYDSLLKGKRVGLITNHTAVNKQMDS

TIDLLKKHAKKGGFSLVAFFAPEHGLTGASHANTLIQDSRSPEGIPIFSLHGKTKRPTKE

MLSHIDLLIFDIQDIGSRSYTYISTLFYAMEEAAKHNIRVIVLDRPNPINGITIDGPMMK

EQWRSFVGYIDVPYCHGMTVGELARYFNEEYKVGCRLEVIPMQGWTREMTFRDTELPWVP

TSPHIPEANSVWYYPTTGILGEISMVSIGIGYPLPFKMIGAPWIDADAFASQLNAQHFSG

VRFHPFHFKPFYGLYADQDCHGVLIVITDDRQFLPVTTQYLIIGMLKSLYPKKFKESMQK

TAHRKEMFSKVNGTDEVWRLMQEEPYIVWRLKELDRAKREQFKQKRERYLLYR

>tr|D6YWM3|D6YWM3_WADCW Uncharacterized protein OS=Waddlia chondrophila (strain ATCC VR-1470 / WSU 86-1044) OX=716544 GN=wcw_1177 PE=4 SV=1

MQLMLGVLMNTPIPPVGGPAGQSPIDPKIGSTRRTEIEPGLEILPDKPGNPVSSNEVKAN

CDWLEKSSLPDILGKVVSAAQDAEGRIPIGPLVSSARNPEVLAAMFATLSNRE

>tr|D6YUH9|D6YUH9_WADCW Uncharacterized protein OS=Waddlia chondrophila (strain ATCC VR-1470 / WSU 86-1044) OX=716544 GN=wcw_0418 PE=4 SV=1

MPKKPQMDADEHGLETAKGQFLVYQAEDGRLKLDVRLEGETVWLTQPLIAELFQTTQQNI

SQHILSIYEEGELAPETTHKKFLSVRSEGKREVKRLLDYYSLDMIISVGYRVKSHVATRF

RIWATQQLTEFVKKGFLLDDERLKNPDQPFDYFEELERRIQDIRISERRFYQKITDIYAT

SIDYDPTLDISIEFFKTVQNKMHWAITGQTAAEIIHSRADADKPNMGLTSYR

>tr|D6YWV0|D6YWV0_WADCW Chalcone_isomerase domain-containing protein OS=Waddlia chondrophila (strain ATCC VR-1470 / WSU 86-1044) OX=716544 GN=wcw_1259 PE=4 SV=1

MNYCRLATYGFTTLIGAFILINSANAAITDKSTGESFPDTETVKFNEKNHRLEATGISTR

KKFFVKVYSVAHYMEDPQKGEGNAAFDAILNSDKPKQLFLKWVRSVSGKKVQDGYKESFD

KVANREQRRDLQVDIDKYMSFFSRGVSEGDTHQLSWLPDGTIEVVINGNQVGIISNRDFA

KTLWSIWFGPKSVVNRNDLISRIR

>tr|D6YRQ9|D6YRQ9_WADCW Uncharacterized protein OS=Waddlia chondrophila (strain ATCC VR-1470 / WSU 86-1044) OX=716544 GN=wcw_1404 PE=4 SV=1

MSSLPRLPNDPVKVKIDGTPYYRTQLIFKYFIIYPFFTYF

>tr|D6YWG6|D6YWG6_WADCW Putative mevalonate kinase OS=Waddlia chondrophila (strain ATCC VR-1470 / WSU 86-1044) OX=716544 GN=wcw_1120 PE=4 SV=1

MSAVGSASAKLLLFGEHSAVYGFPALGIPLPWKTTVKLVRAENMQWKVAPRYLPRLNPLL

SKLPEIFPELHSLSPLEIIVHSETPIGVGFGSSGALCVALTRAVFSQIGQHAQAKEIWSR

AHALEAYFHTNPSGIDTGISTMEQGGYLQKKSSILPSFQPFKKKLPCLIVGAFPRSMNTS

DLVSGLRQKMETASEPMEHIEHLGEIAKLSLDQLDCPATFGELATLAHVHLKALGISIPE

LDSLLEKALTLGACGGKLSGSGGGGAFYIVCENRNSAEKIIRQLEFPNLYLLLDSSFEKI

RQ

>tr|D6YRX6|D6YRX6_WADCW Putative 3-hydroxyacyl-CoA dehydrogenase OS=Waddlia chondrophila (strain ATCC VR-1470 / WSU 86-1044) OX=716544 GN=wcw_1472 PE=4 SV=1

MLNEKLTSVAVVGAGGKMGSGISLLLLREIACLEASKEGQVGSGAYTLTLIDSNVKSLSG

LRNYLRAQMTRFAEKQINDLREYYKNDQRLVSNQEMIEAFVQGAIDCLRFEEEIAQAKNA

HLIFEAIVEEMDVKTRVFSELKKTASESALFFTNTSSLPIGVLAEASGLKGRMIGYHFYN

PPAVQKLLEIIPAAENGESFAALADELAKRLGKIIVYSKDVAGFIGNGHFLREINEALKE

VSELESSYTLVEALCFIDQMTRDLLIRPMGIFQLFDYVGIDVLFRVMQIMAHYLKIEFHS

GLIDQMVAAGCTGGQYSDGSQKDGFFQYRGHTIEKIYDLKAKAYVDLPNDLIGELPKGHL

PWKVMGKEKNREELLKRYLRNLFELDTLAARIASSHLLRSRDIARKLVADGVARSIDDVN

TVIVNGFYHLYGPDDEHLPQEVYK

>tr|D6YRK4|D6YRK4_WADCW Putative addiction module killer protein OS=Waddlia chondrophila (strain ATCC VR-1470 / WSU 86-1044) OX=716544 GN=wcw_1348 PE=4 SV=1

MDKIEIELYETAAGKRPFEVWIKEIKEIHTRAKILTRLDRLKLGNFGDCKTLQEGVCELR

IHYGPGIRIYYGKIGNKVILLFCGGDKGSQDRDIAKAKEYLKDYQSREIGHGKK

>tr|D6YV50|D6YV50_WADCW Uncharacterized protein OS=Waddlia chondrophila (strain ATCC VR-1470 / WSU 86-1044) OX=716544 GN=wcw_0643 PE=4 SV=1

MKFMQGKYLRIFLFVFAVSVALYGGGRLWFHFTDGFRIAHITSNFKPDPRWEIRKLNLEE

EKEVEAALSQSYIYLGKGCQSYAFESEDGHYVLKFLKYKRYRPQFYFYLFTFFPEFQKYL

DRKIEEKKKLLDVLFTSWSICFDHLPQETAMVYLHLNKSNHLNKRIVIRDKIGREHQLEM

DNMEFMIQRKGEMLCPAIDRMMAEGRAEETKKMLSGLFQIILNEYRKGLADMDYALMQNT

AVVDQKAFQLDIGQFVADPMVSNPDFYYQEIFNKYDKFRKWLGKNHPSLVEHVNNELVQE

MGEKFHTMRYMPNPNQF

>tr|D6YVK8|D6YVK8_WADCW Putative membrane-associated protein OS=Waddlia chondrophila (strain ATCC VR-1470 / WSU 86-1044) OX=716544 GN=wcw_0802 PE=4 SV=1

MEFSTQYTILTIIISALLGFAVSAMADNRGRDGRIWFFLGFCFGLLALAALFILPNLANQ

EKEEGVEELLLSESAVEEAESKQWYYLEGEASQIGPLKFEELHKLFQDKKISSTTYVWSE

GMEEWKTVEECRLFTS

>tr|D6YT93|D6YT93_WADCW Uncharacterized protein OS=Waddlia chondrophila (strain ATCC VR-1470 / WSU 86-1044) OX=716544 GN=wcw_1955 PE=4 SV=1

MSMNQPRKARKIAYFSMEIALESEIFSNVVFRILKRTLI

>tr|D6YWP0|D6YWP0_WADCW Putative secreted protein OS=Waddlia chondrophila (strain ATCC VR-1470 / WSU 86-1044) OX=716544 GN=wcw_1194 PE=4 SV=1

MNKIILISISILFFFYLFLCANPLEQEIGQLFISLSERPLPLGGGYKAKNQGLPKKAGSD

FQYTFVLIPKSVRVIGAYDESHEAQRASIRILN

>tr|D6YTL2|D6YTL2_WADCW Uncharacterized protein OS=Waddlia chondrophila (strain ATCC VR-1470 / WSU 86-1044) OX=716544 GN=wcw_0098 PE=4 SV=1

MSQPFEEFPLQSATDALILMHRDVHFGGSFNVMLNYYEGGGKGISDMFEISQIRRLAQLE

QASGSNIAPSILNGADAEKIAEAKKAYQQLKDLFDKRGNEFHYPQLIASLILSEEEEPAE

EIEAIVKEKNAIVPSLLEVLKSEKFYDPLFPGYGLAPSLAAKCLGLIGDKRAIISLFEEI

GRRDFDHEDSLLQAIKKIGKPAKEFLITVVRGMPVNEDNEHAAIALAAFKEDPDVAEACY

DLLHQINFDPLLPLTHYLIFACEGLQDEEKREFLRKMMNTAGVSKDLALDIRSIADKWS

>tr|D6YS06|D6YS06_WADCW 50S ribosomal protein L3 OS=Waddlia chondrophila (strain ATCC VR-1470 / WSU 86-1044) OX=716544 GN=rplC PE=3 SV=1

MALKLMGKKRGMTHLFDEKGNVVVCTVIEMEPNVVTQIKTQGTDGYDAVQVGFDKVTTKD

PRTMDQRVKKPLRGHFKKAGVEPRRHMLESRLSDVTGYQVGQEFDVTAFNEVKFIDATAI

SIGKGFQGAMKLHGFSGMPASHGAKKNHRSLGSTGGRSTPGRCFPGGKRASQMGNRQVTI

QNLEIVQIIPEENVVVVKGSIPGPKNGVVTMRQAVKKAGA

>tr|D6YTL5|D6YTL5_WADCW Uncharacterized protein OS=Waddlia chondrophila (strain ATCC VR-1470 / WSU 86-1044) OX=716544 GN=wcw_0101 PE=4 SV=1

MKHDWYKSGKIVPKNANVIPHVMQKKHAWDKLVKLSGNKAEDFAKVSSLLEESGILSEKN

ILRTREFYNGKIIRSDYKIIINNFEVQAVFETQVGNELFFLKDAWVAIK

>tr|D6YSA7|D6YSA7_WADCW Uncharacterized protein OS=Waddlia chondrophila (strain ATCC VR-1470 / WSU 86-1044) OX=716544 GN=wcw_1607 PE=4 SV=1

MGFPVGGTGVGGSPVPPGIYESQTSAFNEVMKDVIGMLPFDTSVIIPFTQQEYDTVFHYR

SEPGKPSLHPLLFLRTSSEYERTEEDTFNPIYQELLNHLPPDMAEWLSSQMQQPFAERDP

DAVALHHTLSTAASALGWLAVVTAPIAPNSAKAANLLRNIALPYVAFGEALGQAQFIVGK

AFGFLDQAGPNNRYFDFFNNSLTEIQNEIVEAMAAQREIEKGLITEDIRQRLIQSAHLAH

EIASRLQSLSTENTLTILGIQMEALATATAALALSHGTPSLALGSAVALIGINRHNSVLG

PFGLGLDTIMDSVVDGILHSFVYGPEAEISELTALYHDLLSLRDSG

>tr|D6YUS6|D6YUS6_WADCW Probable membrane transporter protein OS=Waddlia chondrophila (strain ATCC VR-1470 / WSU 86-1044) OX=716544 GN=wcw_0516 PE=3 SV=1

MIIEYLALSLIGIIAGLLGGLLGIGGGIFTVPAFLFFFHVFEFPATYTIHVAIGTSLGVM

IFTAISSSGAHFLKKGIQWDYLRYYAPGIICGTVFGALIAHTLPNRKLVTLFSTYIFCFG

IYFIYTAFKKENPYQFKDHKKPPRAITTGGVGLIGGTICSILGVGGAPITIPFLTSHHVQ

LKHAISTSAFVSLLVGTVGTLSYLYFGLGSQVSNGSLGYLYLPGMLIAGLSSVLSAPFGA

NLAYMLPTRILRAIFGIFLVIVSFVLYS

>tr|D6YSL8|D6YSL8_WADCW HlyD family secretion protein OS=Waddlia chondrophila (strain ATCC VR-1470 / WSU 86-1044) OX=716544 GN=wcw_1719 PE=3 SV=1

MQIACTLLRTKIEFAFSSLMKKTQWLPLTIIIAILALAAVIGYALWEKNHEENSLTLYGN

IDIRQVDLGFRVSGKLKTMLYEEGDVVHEGDLLAELDDAPYKQSFLESEAKFTSIRESLA

YAETQLKRRSPLVQEKTVSQEDYQNAYYNQKILTANLLEAAASMENSRIRLQDTRLVCPS

DGVVYTRIREPGTVLSIGEPVYSVAVNTPIWARTYISEPDLGRIYPGMAAEVYTDTPENP

VYEGRIGFISPIAEFTPKNVETPDLRTNLVYQLRVIIKNPDKGLRQGMPVTVKLKS

>tr|D6YT20|D6YT20_WADCW Uncharacterized protein OS=Waddlia chondrophila (strain ATCC VR-1470 / WSU 86-1044) OX=716544 GN=wcw_1877 PE=4 SV=1

MGDRACTNDHFEEQLFLEKHISLIPKRRKDLKRQHTAETKQNRNSFQ

>tr|D6YVH4|D6YVH4_WADCW Putative class-C beta-lactamase family protein OS=Waddlia chondrophila (strain ATCC VR-1470 / WSU 86-1044) OX=716544 GN=wcw_0768 PE=4 SV=1

MERSIFLLFVLFFNTLTSLEHVSRQHVQKAIDQIDRLVIESMDRLSVPGAAVAVVYNGEV

LLLKGYGVREWGKMDPVDNDTVFQLASLSKPITSSVLAAVVAEGRVRWNSPVISLDPSFR

LSDAWVTQHVTVADLLSHRSGLYEHAGDLLEDLGYDGKTIIHQLRFIPRLNPFRASYAYT

NFGFSEAAYAVANFFNTHWNTLANQKLFAPLRMKHASFRYLDYQNAVNKAVAHQVKEDIP

ILKHVRNPDPQAPAGGASMSVKDFAKWMIFSLNGGTYKSYRLVPETVLLESQMPYIVSAV

DLLDDTINFYGLGWGIKYNRFGNKVLDHSGAFSLGIRSQVVLIPELKVGIAVLTNSYPHA

LPEAVTQSFMDLIYTGKIEKDWLPIMNEKFIKVMGDEHRFYKAPQMYVPHIELERYAGDY

YNEFYGGMRIVSEEDRLVLVIGPKSYRFPLKHYNKDAFMMETKGENSVGETKVVFEFSSD

GSLKRVVVDYLNNHGLGVFLPVKE

>tr|D6YT98|D6YT98_WADCW Uncharacterized protein OS=Waddlia chondrophila (strain ATCC VR-1470 / WSU 86-1044) OX=716544 GN=wcw_1960 PE=4 SV=1

MTELPDNGTLTALYIEGHWEDIVKHHPIAAGGIVFEWQDEYWKAGNKHQQTPSKAVNIDF

PGGCWTKKGLES

>tr|D6YTK8|D6YTK8_WADCW Putative type III secretion protein SctL OS=Waddlia chondrophila (strain ATCC VR-1470 / WSU 86-1044) OX=716544 GN=sctL PE=3 SV=1

MSKKFFSLIHGGNIHIAPETKVIPAEALSTLVDAQEVLAAVEKDAELYKKEVVTEIEKLK

AQAQQEGFEQGYSEWMEKIASLEEEIRKVRSDMQKVIIPIALKAAKKIVGKEIEASEKTV

VDIIANSLKAVSQHKRIKIYVNAKEFDTVEKSREQIKKIFENLEALSILPQDDVDSGGCI

IETEGGIINAQLENQWMILENAFQKLVNTQPTAVGITGDSEMQKAKDAK

>tr|D6YW31|D6YW31_WADCW Uncharacterized protein OS=Waddlia chondrophila (strain ATCC VR-1470 / WSU 86-1044) OX=716544 GN=wcw_0982 PE=4 SV=1

MFKYFLYLSLMFFSALCAKTTYLFQKDFDQGTYLIDQPGTYILAEEITFNPQHGRKPYEV

AQVSPSQLKSNGGKYDDQAFGLGFFAAIVIQASDVTLDLNQKTIQQSKEHALLQRFFSVI

ELANVPFIPGEGPHHFGAKIVSASKVLIKNGKIGRSSHQGIHGNGNRFISIEDVDFEDFE

VAAVSLNGVDGLRIKNCTAKSREDVPVLGIFATTQFILPYLDYLVESGSQTTLRVDGKEI

SPIEMRNKLRQAILNVHEDLIVKNKPTIDKFAHPEEYELFHNAAGVIDGNCYGFLVNFYG

QAVGGFPIYHQIPEDKRAKNITIQNTHVKRLKGKINEIVAINHENDALVDPLNAIFQIHN

THPDTKENLSILIDADGNFLYKGNMASNAQALVAKALKNGDFKQSKLDLSRSNITPEVID

WIESGTHSTQKLTFICNSDSQFHLNKGVIGFKIDGAYQATLDNTTAENIINLSERGSSLC

GMYTKSNPSATLDGCGGPRTRGYSIAGSNHVSLRNCSASHLVALCGTATGFDIINDSSNV

LLQNCAVTDVLAGEKFERNHAPNEPPMAIGFRIGPSAHDIALIGVHASKMKGFSGEEIVL

DESSRAQLK

>tr|D6YTA9|D6YTA9_WADCW Acylphosphatase OS=Waddlia chondrophila (strain ATCC VR-1470 / WSU 86-1044) OX=716544 GN=wcw_1971 PE=3 SV=1

MHAIVHGRVQGVFFRDTTKRKASEMGISGTVENLPDGTVEIFAIGKREDLENLIEELSGK

KGPGYVKNVDISFREPAHVYDGFRIIFS

>tr|D6YSD1|D6YSD1_WADCW Uncharacterized protein OS=Waddlia chondrophila (strain ATCC VR-1470 / WSU 86-1044) OX=716544 GN=wcw_1631 PE=4 SV=1

MTSPQIDLINGSGIYLIPMSSDGSADDSIFGELISLMQGAIDPDRLVIQENALEVIGGFL

NLGVQVGWKEYGNDVRVGAAFIRCMLTEYSQSVSKLEGESDENEKILKRVSDCFANIGDL

IQSPFWEKQPEEGYRPKTNRVRYDADVKGWQKHAVCRMVGNIASNYHSDYPFFEILVNSS

KESIDSEICKPFYVTIHDNEKVLDKRRAVLSGLKLFPGEVAHPIYYSSSLKNPEVFLEKT

SLRIGPNARRILEGLTFLSNVKLYTQKTGNCWIKQPMRCLLASLYLEILSVKQDVSPQEA

WKAAKALYQEIQKSAGVTLIRELMSETTLSDSQIRTIDREISKRFSL

>tr|D6YUM3|D6YUM3_WADCW Fused isobutyryl-CoA mutase OS=Waddlia chondrophila (strain ATCC VR-1470 / WSU 86-1044) OX=716544 GN=mcm PE=3 SV=1

MIYRILTASSLYDGHDAAINVFRRLFQKAGLEVIHIGHNRSVEELVKTAIEEDVNAVCVS

SYQGGHMEYFRYLRQRLNEEGGEEIAIFGGGGGTILPSEIKQLESEGITRLYHPEDGQKL

GLRGIIKDAISRMKPKKREADTLAKALTRIELGESVSVPKKDVQAVVIGLTGPGGAGKSS

LMDELILRYLYSTPSGRVGVLAFDPTKRKTGGALLGDRIRMNSIYNERVFLRSMATRGSG

TEMSSVAKNALELMKRSGFDLIILETTGIGQGDAEIVDLADFCLYVMTSEYGASTQLEKI

DMLDYADVIVLNKFDRMGSEGAFEDVLHAYADSRGVRVTEKNRDKLPVFGTIASDFNNPG

VTRLFIRLMQILKEKGLLTSTGNQELDLEKTRELNNSYTFAIIPSEKTFYLGKIASALRN

YHRETKQKGEIASEIYKLRTTGRDAEKLEKELGADNQELLEQYRQLQTKYREEELGYEVR

GKTIKRPLFSKSLSGLFISRIALPKYRDWGDILNFMRKENLPGHFPYTAGVFPLKNVEEE

PKRQFAGEGAPERTNKRFHYLTRNDQAHRLSTAFDSVTLYGEDPRNRPDVYGKIGNSGVN

IATLDDMKALYKGFNLIDPLTSVSLTINGPAPMILAMFLNTAIDFEMEKQGQEPYEKVKE

RVLQVVRGTVQADILKEEQAQNTCIFSSKFALKMMGDIQEYFIDHNVRNFYSVSISGYHI

AEAGANPISQLAFTLANGFTLVEYYLSRGMKIDDFAPNLSFFFSMGMDPEYSVIGRVARR

IWAIAMRERYGGNERSCKLKYHIQTSGRSLHAQEIAFNDIRTTLQALYAVNDNCNSLHTN

AYDEAITTPTEESVRRAMAIQMILNREYGTTKCENAIQGSYFIEELTDLVEEAVMEEFDS

LSRRGGVLGAMEKQYQRHRIQSESMKYELMKESGELPIIGVNTFTSEAGQPDYEHMEVIR

ATEEEKQSQIERTQKFIERAGDRNAEALERLRHTALSGENIFAELMETVKYCSLGQITRL

LYECGGQYRRSI

>tr|D6YUZ2|D6YUZ2_WADCW Elongation factor Tu OS=Waddlia chondrophila (strain ATCC VR-1470 / WSU 86-1044) OX=716544 GN=tuf PE=3 SV=1

MAKETFQRNKPHVNIGTIGHVDHGKTTLTAAITNVLAKKMGGKARSFEEIDNTPEEKARG

ITINSSHVEYETPNRHYAHVDCPGHADYVKNMITGAAQMDGAILVVAATDGAMPQTREHI

LLAHQMQVPKIVVFLNKCDMLGEGDQELLDLVELELQELLEAKGYENAPIVRGSALKALE

GDAEWEEKILELMSTVDDNVPEPQRDVDKPFLMPVEDVFSISGRGTVATGRVERGVIKLN

DKVEIVRFGDKKESVATGLEMFNKLLDEARAGENVGVLLRGVNKDEIQRGQILAAPGSCK

PHKKFKGPVYVLTKDEGGRHKPFFTGYRPQIYIRTTDVTGTVKLPEGVEMVMPGDNVEIE

VELIYPVALEKGMRFAIREGGRTIGAGTVSEIIE

>tr|D6YSJ9|D6YSJ9_WADCW Phosphoglycerate kinase OS=Waddlia chondrophila (strain ATCC VR-1470 / WSU 86-1044) OX=716544 GN=pgk PE=3 SV=1

MTIALKELPLKGKKILVRVDFNVPLDKKGRITDDTRIVASLPTIRYILEQGGNPILMSHL

GRPKGRQDLSMSLSVCARRLAEMLGKPVIMAPDCIGNQVEALINKLRPGESLLLENLRFH

PGEENPTEHPEFVNALAKLGEIYVNDAFGTAHRKHASTYFIVPFFKGRAAPGFLLEKEIE

FLGNEISKPKRPFFAVIGGAKISSKIGVIKALASKVDSLLIGGAMAFTFLKAKHVNIGDS

LFETDCLDTAKAIMETYEKAGVRILFPVDHVIVTDIDQDQFPKIVDNQSGIPKGYKGVDI

GPRTIEIYSSELANGKTIFWNGPMGIFEHQEFAKGTKAIAEAIAKVKGTKIVGGGDSVAA

IRSLNLAHQFTHLSTGGGASLEYLEFGTLPGLEVLKNIP

>tr|D6YS67|D6YS67_WADCW Glutamyl-tRNA reductase OS=Waddlia chondrophila (strain ATCC VR-1470 / WSU 86-1044) OX=716544 GN=hemA PE=3 SV=1

MQIGVVGVNHKLADLKLRESFAKVCNEYFRDTFARHGKHNTLLLTTCNRTEVYFSSDVLS

ESHSYVLNILRENLPVSDETFDQKLYTYFGHDCFIHLARVTAGLDSAIVAETEIQGQVKI

AYEKAADAAILPRELHYLFQKSLKIGKSIRSELGLGRGVPNLEHAVLNAGFHFFDKPEKA

NILFIGASDINCKILSFLQSKGCKHLTLCNRTKSNALEASKKYHINILDWEQRKMWTAFD

WVIFGTKAGDHIISKSDLHQKPASDLLLIDLCVPRNVDPKLGREESITLLNIDQINRMLK

FRKKRLNNHLSKAEKIVYTSTKRHIDLFHSKEERKLQLLATG

>tr|D6YVI5|D6YVI5_WADCW Uncharacterized protein OS=Waddlia chondrophila (strain ATCC VR-1470 / WSU 86-1044) OX=716544 GN=wcw_0779 PE=4 SV=1

MKKGVLGCFLINAVALSASQAVFYTEQGEEYLCDIQTPATVWEIKESLEDVLPFTKEEML

FITEGAFLCEEEYFLPFDGQKIWVTSLDFDQPVPKKTYAGYRNYDRKAAESEKKDLRFIL

KTLATKSIAGLLKYRSELEAAGDRIDKLHPLRFLETAFTDEELKAYMHNIRQRGGWIWGE

FIKGIKGSLQDEADIGNLTDDMILDFSCQVEIDLSKIEGLIHREKWEDFIKALIIHIPRK

GDSDRYDQ

>tr|D6YVD0|D6YVD0_WADCW Uncharacterized protein OS=Waddlia chondrophila (strain ATCC VR-1470 / WSU 86-1044) OX=716544 GN=wcw_0724 PE=4 SV=1

MNIYLADTLPVEVPAGFEAVSITLDAGLKSLLEWRKELLEADRLKKKGFKLFWNLDFDLQ

LTCTEAQVSSLRLAVEHFCSAVWEKFREETAGVCLYLGGDLLNDEQIRVLEILAGGLPDE

VEAFIMLDVSSLSSPTEISRAISKERFPHFTLVVKGVENPLPEFGWESVCGSRGMIGRHL

VENAIVEPTIGLCIPEKGASPSLDEIALWLKSKDLPFRMIPETLLTSEWQGLDDVIVDSE

TVASLCKRRLMGFCAAGGTIVTIGKSLGLPIEVSCEEWKDSLRLKQDLSKSRLLS

>tr|D6YWY5|D6YWY5_WADCW Uncharacterized protein OS=Waddlia chondrophila (strain ATCC VR-1470 / WSU 86-1044) OX=716544 GN=wcw_1294 PE=4 SV=1

MRVSEYFNPFSSQFPNTVQHFKNLGFWSKAAVVGLTALAGTVSFFALGIGGVAMFRFTVK

VFSGGELPKEAAKVDEVRERVLAPETPEEERDSLADSQKIKVNNKTQHDLEDGSTGIYLK

IAKRLLEASPDQVNADLIDEIPVEREEDYDIESHESGEFGLKRWKFDEKEGFSYGKPPYS

GTVNQFKEMIAKAAQDLPKPTALIIKSVFKTALVVIRPSGEFWLFGSCGSDGTYVRKFSS

EDQFVKKLGDFFPFVDLEGDQQDSLPINFFNAYAFVENKMPEEEQDSLAAFSRIEANITQ

YDPLLDNAGKSACTCICLKAAEMLLEASPDQVNAGLIDDILVEGVADYNRFKVGGVVEHT

SVENYELNTFELKRLEFRDVDNPFSAEGNPYAGTLDSFAKMMEKASDSKDLPKPVALVMT

KSNMTITIVIRPDGKYWLFDPHGTNGKGAYIESCNTDELIKKIKEIFPKTSYPGMTEDEN

LGFNSFEAYAVRRS

>tr|D6YW99|D6YW99_WADCW Uncharacterized protein OS=Waddlia chondrophila (strain ATCC VR-1470 / WSU 86-1044) OX=716544 GN=wcw_1051 PE=4 SV=1

MHWRREMDLLDLKKLALLGIAGGVLMAASGHHPVFADGQGGSGGIDKEANGCSGGNGCEG

VEDDSPDDGE

>tr|D6YRU1|D6YRU1_WADCW 30S ribosomal protein S21 OS=Waddlia chondrophila (strain ATCC VR-1470 / WSU 86-1044) OX=716544 GN=rpsU PE=3 SV=1

MTTVKVRPGESIDKALRALKKRLDKEGVMKSVKAHRFYMKPSIKKRAKSKAALKYRR

>tr|D6YT18|D6YT18_WADCW Transposase OS=Waddlia chondrophila (strain ATCC VR-1470 / WSU 86-1044) OX=716544 GN=wcw_1875 PE=4 SV=1

MLVYENHKNFRAKIFSEKEYHGFTASKNQYFFGIKVHMIVDTEGVPIEFSFTPGTQIAT

>tr|D6YS86|D6YS86_WADCW Uncharacterized protein OS=Waddlia chondrophila (strain ATCC VR-1470 / WSU 86-1044) OX=716544 GN=wcw_1583 PE=4 SV=1

MESEPFDSILDQIAELLEVADENKKEAIKGKVDKDLMNQLDFLEMKVQFFRNVTDQALKM

SGITDEELGSHIENFSDNPTTKSQKMIARADQLKKRLQVLEKQYELRFRAAKMQKKQEKS

TGKKRKKKFKKLGGQGWLPL

>tr|D6YUQ6|D6YUQ6_WADCW DNA recombination protein OS=Waddlia chondrophila (strain ATCC VR-1470 / WSU 86-1044) OX=716544 GN=rmuC PE=3 SV=1

MDVIFHFFLSYLPWILTSVMLFACTGVLGWLLWRKEVRLRTFQEENRQLLSRRAELEATL

EHERRAHGEKVSLLNEAQTKMSHAFKALSVDVLKNNSESFLEIATAKLEKLQEAAKGDLS

VKHYAIKESLEKVNDKIHMLEKARTDAYASLTEQVKLLAMTQTRLQSETSNLVKALRQPV

VRGRWGEIQLRRVVEMAGMVEHCDFLQQESVQSENGRLRPDLIIRLPNNKQVVVDSKASL

SAYLESLEIDSEKEKLEKLKDHAKQIRHHITQLSAKSYWDQFQPAPEFVVLFIPGETFFS

AALEQEPSLIEHGVEQKVILATPTTLIALLRAVAYGWRQEQIAENALAISELGKTLYERI

RVMGEHIEEIRKGLERSVNGYNSAVGSLEGRVLVTARKFKELGASAGKELPVLKNVDLSL

RKLSEEVSDSEYITD

>tr|D6YUJ0|D6YUJ0_WADCW Uncharacterized protein OS=Waddlia chondrophila (strain ATCC VR-1470 / WSU 86-1044) OX=716544 GN=wcw_0429 PE=4 SV=1

MNHVESLGSNPVKNSVQTADLPVIEEIVEKTHTLGERTIKAGQLALWNIGTAFLINLDLL

KEDSWLRKEYQAVWNGRTIEVIKTSQSPSQQVEQSLVPKVSTDLATRSNIGALEENPKTK

ESSLVGKKFTIQLANGVKGELEILDLVTTLISSPNNFTTSHLPQLEHQSSSSALSRLWNG

AGQAFNQVFGFRGQLSEATVQQSSTMRLTIDNIETIFKISFNVPAERLTPQFMNSVAGNI

FGQIEGSSQPNFSVSKKISSPPQPLMLEGSRPVMKTSISKCSPSLFLTNQEAPKHLGIPP

QVQNKRQPVVKTEERQQAPQRESERPKPKYTQTPPLVGFIMYWAMMHMRGANMGADRFTA

KTPEKSQPWYSRFYSVFTKAIAYIGQSARTYSSKLWWQNATPIDTRIINVGNVPVKVDLL

GIRLNDNRELD

>tr|D6YUF8|D6YUF8_WADCW Putative tetratricopeptide (TRP) repeat protein OS=Waddlia chondrophila (strain ATCC VR-1470 / WSU 86-1044) OX=716544 GN=wcw_0397 PE=4 SV=1

MKQLEKDQLFSKSILWDLQLAVYREFGPSAWAEKGVPFYLTSNPLLARQFVSVIESYFFE

MPASNPSEPIYVFDLGAGSGRLAFLILKDLIKLRDRVPFVYVMTDMVEENLKFWRQHPRL

KPYFEKGVLDCASYKNDQKEPLKLQVSGKILDVVQNPMVLICTYFFDTIPQDLFRVKNGK

LEEGRISISVPAGSTQSIDPDWINSLEESYSFQPVDNASKYYPNEAANQILCEYVKEMEG

ATFLFPSGSLLTLEYFQKLSKGKMLLLAADQGVCSKDQIMRWGIPHISRHSSFSIAVNYH

ALARYFELHDGLGFLPSLPDHRFALIAGILGEGVYNQACLAFYKEMDVLQPVEYWHLTGI

SDEQADELSLKQLLILVKMGNNDPLNMHCFFKQIRDKLSEADEETLDFLKSVVKKCVGNF

YPVSPSEGDFVQNMGVLLFEAKDYESAAQVFAYAASIKGLDAQLMQNLALCKAKIHSHL

>tr|D6YVD1|D6YVD1_WADCW Putative K(+)-stimulated pyrophosphate-energized sodium pump OS=Waddlia chondrophila (strain ATCC VR-1470 / WSU 86-1044) OX=716544 GN=hppA PE=3 SV=1

MVPLLIITFISLAGLLFAAYLANWILSHEEGTEEMKSIARAIQEGAMAFLHREYKVLAIF

ILILTPILWYSLDEAGSSVNQGKYTAIAFVIGALCSGGAGYLGMRIAVQANVRTANAAIK

SLHEALQLAFSSGAVMGLCVVCLALLGLSSLYLFLVPIMGMSHESAIHIIKGFGLGSSSI

ALFARVGGGIFTKAADVGADLVGKVEIGIPEDDPRNPAVIADNVGDNVGDVAGMGGDLFE

SYVGTIIASLTIGAFTFHSFPAIIFPILVAGIGIVASILGSFFVTKGRRSEQPLVHGAFR

NGLFFASTLVVAGVWLLSRTCLPESFELLGASYTQNGVFFAIVTGLIGGILIGLITEFFT

SSSYSPVQEIAQGASTGAGTNIITGLSVGYRSVVMPIIVICCVIYLAFLLAGLYGIALSG

VGMLSTLGISLAVDAYGPVADNAGGIAEMAKLDKVVRERTDTLDEAGNTTAAIGKGFAIS

SAMLSALALFSAFSIAAKLENTSITNPTVLIGLFLGAMLPYLFSSLTMKAVGKTAFKMIE

EVRRQFKTIDGLMEGKTRPEYGKCVDIATQAALKEMIIPGIIAIASPIAVGLFLGNEALA

GLLAGSLISGFLMAVMMANAGGAWDNAKKFIESGELGGKGSDSHKAAVVGDTVGDPFKDT

SGPSLNILINVMNIVSLVFVPVFVEYGGILTR

>tr|D6YUI8|D6YUI8_WADCW Uncharacterized protein OS=Waddlia chondrophila (strain ATCC VR-1470 / WSU 86-1044) OX=716544 GN=wcw_0427 PE=4 SV=1

MIKRLLFKKAAFYFHYLAATRVISNRSRSGYNTPYSSYDETCNRIGRENANAKNCQCNPN

N

>tr|D6YS05|D6YS05_WADCW 50S ribosomal protein L4 OS=Waddlia chondrophila (strain ATCC VR-1470 / WSU 86-1044) OX=716544 GN=rplD PE=3 SV=1

MTELKKYNLEGKETGSVEANAKITESSIHSQLIKEYIVAIRKNARQWSANTKGRSEVKHT

TKKPHRQKGTGRARQGSLVAPQFRGGGVVFGPKPKFDQHVRINKKEKRSAIRFLLGEMMR

ENRMIVLENAMMDKPKTKTLAAFIKKANLGKRVLFLAEGKHEEIQTAHKTVAVSVKCNDH

ENFRKSLNNIPKTQFALAKNVNGYDLMLADNIVVTEAALEEINHWLA

>tr|D6YUS5|D6YUS5_WADCW Putative cytochrome c family protein OS=Waddlia chondrophila (strain ATCC VR-1470 / WSU 86-1044) OX=716544 GN=wcw_0515 PE=4 SV=1

MKLSYLSGGIFFILLGGIYFYFLRHPVDDIARVHSYNVPDKYDLYDPEMAPEEIRDQVVS

GFQMMIETKNQLPQYVGNDMNCRNCHFNGGNSMGGRSCGFSLVGVVHKYPRKLPSGKEYT

LEERVNSCFMRSMNGKPLPVDSKEMRALVTYMEWISNGIPKLESYPWLGAKKLTSSHVPD

PKRGEKLFAMKCAPCHGENGEGQKRPYQLDYPPLWGNGAFNDGAGMSKLDMFAYFIYKNM

PYNDPRLSVEEALDIASYVTKQPRPKLENHE

>tr|D6YUH5|D6YUH5_WADCW Putative acetyltransferase OS=Waddlia chondrophila (strain ATCC VR-1470 / WSU 86-1044) OX=716544 GN=wcw_0414 PE=4 SV=1

MIEIRNATPEDAEQIAKVHVKSWQTTYQKIVDEEVLSAMNWEDRVENWRETIKSLGSQAT

ILVAVENDRIVGFLSGGQARQTLENCDAEIYAIYLLEESRGKGIGRNLVNKYFKWLTENG

FHSCFVWIAKENPYQSFYLSVGAEKTDLEGISRVRSNKIPTVAYIWKTVDNLTNFP

>tr|D6YWU1|D6YWU1_WADCW Uncharacterized protein OS=Waddlia chondrophila (strain ATCC VR-1470 / WSU 86-1044) OX=716544 GN=wcw_1245 PE=4 SV=1

MLNQERKLQFLEVELTPYTFGRESLTPIPNRLEPYSSEGSALQNCLAGFLHGFPLIRSGT

VNSDSKVKQLLSLAKMLHKKQQNSLPLRVVFDQSQSVEFEWDMIHEEHQSIVHRSEHTQD

SIHHLCSAADPLVSENPPSFKVKSTLDILDQMPKILNVEAWADFCLWFGLRLQEILKEFP

YDAVDAYTLEKHLQDFLHETHSPFLKRVRQHLLMLQLLMGYPEEPAQASFNQEKEVIKAR

YEMQKLIRDHYKVLVKIVHVVEMIGHEYPELEPVLRAGIYLRELIGPEVNDENQAPVSWG

RGQLLKQLFCAEFNAIPVISSRKGLGRTHFAFAIRAAAMTMRERLRWPELKHFVLNWNEM

TVLVNRLAAKQGKNGIEDPSLNLSARHVIEFRVLVFNYLRNFCLPISSWNRKGDFLQLTG

NEFIDPGFLNFLPAFREDRPLITYDYASGFPSGLTEEGLKFYAEIN

>tr|D6YVS6|D6YVS6_WADCW Uncharacterized protein OS=Waddlia chondrophila (strain ATCC VR-1470 / WSU 86-1044) OX=716544 GN=wcw_0871 PE=4 SV=1

MTFNPSGHVPVGTGNQYPVTSYTPQTTVIATQQGQQARHVHVIAHQHVGAPGYSQVPLRN

VPARQLPNTGTTTVVFNQRAYRR

>tr|D6YVT6|D6YVT6_WADCW Uncharacterized protein OS=Waddlia chondrophila (strain ATCC VR-1470 / WSU 86-1044) OX=716544 GN=wcw_0883 PE=4 SV=1

MSKVGRNDPCPCGSGKKYKQCCWNKRLPLGKRKFKASVIKSGLMKEFPDLMERTFGEAIK

GEEGSNLKEALSSIVPKPEEMPEIDANQSLKEYLEASEEAKKKRSEDDSA

>tr|D6YTN8|D6YTN8_WADCW Glutamate racemase OS=Waddlia chondrophila (strain ATCC VR-1470 / WSU 86-1044) OX=716544 GN=murI PE=3 SV=1

MNAIAVFDSGLGGLTVVKALCEHLPQEEIIYFGDTARVPYGGKSRETVIRYAREISAFLL

SQEIKALVIGCNTASAYAADLLAAELELPVFNVIDPLIEEISACQAEHIAVLGTAATVRS

GIYQARLAQKIPNVKVTGISCPLFVPIVEEHFQNHPAARLIVEEYLFRVKDERMDTVVLG

CTHYPLLYCLIREYLGDEVRIVDSASACAKQIQAALFSHPFNNSLSVKGKLKYFVSDDPE

RFKHLGEQFLGVEIGPVECVDLNSVLQIPQNFAKSG

>tr|D6YTA2|D6YTA2_WADCW Putative cytochrome oxidase, cytochrome c subunit OS=Waddlia chondrophila (strain ATCC VR-1470 / WSU 86-1044) OX=716544 GN=fixO PE=4 SV=1

MSGKQGNFFYKIEWSAWLTIIGILILFSSAVIVTLIAPRHIDYTWVQPTSDYQVQMYEVA

DPNIYFSSHDSELQAVYHLQDGYSLTAFKESKTVRFVAPPHLQKYITFYGDDALKLSSRL

ILLRNPESALAGNPFDGAKRAEALQKELQASWEREHSDWRDQGLQRPFFHILELFEPEGS

EAFAIAPIDRVQESWVDENFIILDEEKKQPWHSSKGVVYINNPHEYRVKYFESGGKEEWR

FDPSGESIKNLEELQGNLLGFRSRKNLIQLGEDLYRIEGCWYCHTDQTRTLIQDVVLNGT

ESYPAPPSSANEYIYERITFLGTRRIGPDLSRVGIKRPSRDWHQSHFWSPKTESKGSIMP

AFQHFFDDDPRGTLGRGVSFPNYKFEAIFQYLMTKGTRITPPTEAWWLGKDPVQTMEIIQ

GQYNKDESVK

>tr|D6YSA5|D6YSA5_WADCW Putative membrane protein OS=Waddlia chondrophila (strain ATCC VR-1470 / WSU 86-1044) OX=716544 GN=wcw_1605 PE=4 SV=1

MGGVTGSNDPTVANWGNIGFQGAHISGGADARRFEGFQDGTSSLNGVWGNTPYLQDIGSP

KLQQVILGLSDQPNGTHGETQYTPTHAYYDSFSENLEQLIDKYGLSEEQINQLVMAHLTD

TPADDNFINSVLNELDSAVAGDLAQLFELPSTADQASNLRERAGNHSSALKGQISDLQAY

ADSLPEGSEKQAIQEKINELTQNLDSFNEHLASLPENPSREQLAKLATELGMSGENLRSI

AGELSELAGDSPELKSLLANFQHSVNSSTTTLDGLLQELYVSWEISTGDFSSLTNSFKET

FANKLTQEFDKAFEAELTKFTDDPELQAQLRFVHYNPDAEIPENIKSMTQTVEQNAINQM

NAEGWNIPQSYTPPSNGLSYNMRMQNSADEMFEGMLQNWDPPLTPDQQKALRNMYYGVEK

PAGDLAAVLQQIESGVAAELAAAFGLPDGFPVPKGSFSHQGNINGQFQMKFLELLNALPA

DQKAAVLQAINDPMNPAISAETKALLNKLFNQAAGSIRAQFGLPEGWTPSASVLREIGTM

SPENQSIATQISEMETEVALAISYVEQWPNSPTKAVLLNVLKIVSEAIATLKAQLAIIMQ

KDAELATKLGQAQLDTALLKVHENLKKLEEIKNKQAKMKALGPLFKIFKVISFIFTAVLC

LAAGPVGWAILGVLIADMAINGLDSDKSLIGKAFEAVSEAITGLLESWGFPPELAQMCAL

VVNLMICVAISMAGSPMLGMQIFFEHSGIIQSFFTDVCGCDPMVGEIIAMVTQMVVEIVV

MILLTIVTGGAGATLLVASVVGRVAMMVGKVVQKIVTIIMRVAQLVMKIAQAAQKFARLS

QTLMKISMELMNFALKVNKIATKIIQWAQKTLRLTKQVTQSMGKVKWTKSLKNAFSKTSK

TGVQQFDDAVNALRKQLDVAKWLFRGIGVTFGVLQTTVVVAQFRNSLLAAEIARIRGDME

AMMTELEAFIQVLKKMVAKILEALSGIGEWIGQIGQQQGSMWKEASETMDAVAASNQAS

>tr|D6YWX0|D6YWX0_WADCW Permease, major facilitator superfamily OS=Waddlia chondrophila (strain ATCC VR-1470 / WSU 86-1044) OX=716544 GN=wcw_1279 PE=4 SV=1

MLRDRKNLLSWALYDWANSAFSAIIQTFIFAVYFTQQLTSDPAIGSALWGGINGTAALII

AILGPILGAVADHGGKRKAWLGLFTLLCIVCTGTLSFAKPPIGNLFSLLTVLTTAIAASE

LAFVFYNAMLPNLAPHSHIGRWSGWGWGLGYAGGMLSLLISLLIIQFGYPVQLTFFFCAA

WYLIFSIPLFLFTPPTSGLGKPLLKAVFYGMKQLIETLRQMKQYKEIVKFLIARMFYMDG

LTTLFAFGGVYAATTFNMPQSEILLFGIMTNISAGIGAVGFAWIDDRIGPRKLILISLAG

ITLPTLWILLIPYKLLFWILALFAGLFVGPLQASSRSLMAHLAPKHLYNEMFGFFALSGK

ATSFLGPWIVSWLILEYGSMRIGLAVVVAFYTIGGILMLFIED

>tr|D6YX19|D6YX19_WADCW Putative helicase OS=Waddlia chondrophila (strain ATCC VR-1470 / WSU 86-1044) OX=716544 GN=wcw_p0014 PE=4 SV=1

MLTLRKYQRECLDAIASNYKNGNCRQLVSLPTASGKTVVFASLIKEFEGKSLVLAHTNEL

LEQAREKIQMIAPNLSVGLVNADSKEFDFPVIVSSIQSARQPNNLVELQAQNFKLLVYDE

CHHAASKTSRNILNALGFGCKTDRLLCGFTATAFRQDGKGLKEVFDTVAYQRTIKEMIEE

GYLCPPKGIKVSTDIDLSKVKMGDGDFQAESLAKVMDIPEIRQIVFDAYQKEGEGRQTIC

FGVNIQHAYNLSCLFNCCGISSDTIHGRMSKSERESVLKRYRSGQIQVLCNCQVLTEGFD

APETSCVIVARPTQSKGLYQQMAGRGLRLYPNKRDCIIIDLCAKHHGLCNTVTLLEDSEK

INEVEKLEKSDQPGLVESFPANLNQKLKAALIRFDPLGQEFTWTCNESNIYVLKGDNIRL

GIVPINKDRYRVVLASEKGSQTISDDLNFEYSFAVAEDFARSNRDVFIVSDREAKWRNFP

ASAKQIALIRSKGYRAGLDKLTRGQASDIISSGTLRGGSGCYVEK

>tr|D6YTT5|D6YTT5_WADCW Uncharacterized protein OS=Waddlia chondrophila (strain ATCC VR-1470 / WSU 86-1044) OX=716544 GN=wcw_0171 PE=4 SV=1

MKLRFLGAAGTVTGSKTLIETNDCAILVDCGLFQGYKELRGKNWSGFPVSIEKIHSVILT

HAHIDHSGYLPILVRDGFKGDIYATPATRDLCEILLRDSARIHEEDARRANKYGYSKHKP

ALPLYTEEDAIRCLRQFKSLDLGIDTPLSRSLIVHASRAGHILGSAMLTFRTNEETLVFS

GDLGRSKSAIMPPPAQIQVADDLILESTYGNRLHPDEDDEQKLGEIIRKTAKRGGVVLIP

AFAVGRTQIVLYLIHQLKAKKQIPDIPIFLDSPMAQDATDIMLNYSNEHTLSPDLCRKVC

STAQYVQSAQDSKQLHGKHYPMIIISASGMAEGGRVLHHLKKYAPDHHNSIVFVGFQAPM

TRGDRILQGAREIKIHGNMVPVRAKIELLETLSSHADYQETLSWLKGFVKPPRQVFLNHG

EPAALGALKDKIIDTFGWRVTIPSYMDLYEL

>tr|D6YUP2|D6YUP2_WADCW Transposase OS=Waddlia chondrophila (strain ATCC VR-1470 / WSU 86-1044) OX=716544 GN=wcw_0482 PE=4 SV=1

MKFIQKNDIIFHERISYKIYFLFQNFIGYALFISNTTFDHISLYYRCLKQLVRFAKRFNI

ELRQSYVFLSKKTLRRVSQYVHSRKMKLAKRETKRLKTYFGRVLREIERHVEKDSELKRM

TSEWLSILRGIFEQKRTDSPKIYSVHEPQVECISKGKAHKKYEFGCKVSIVTTHNQGFVL

SSEALHGNPFDGHTLKQAIVDAEACSQKEIRRLFVDKGYRGHKVKGKEVFISGKRKLTLH

FKKMLRRCQAIEPTIGHMKSDGKLNRNYLKGRVGDCLNAILCGIGHNIRLILNHFDRKMQ

LV

>tr|D6YS70|D6YS70_WADCW Probable endonuclease 4 OS=Waddlia chondrophila (strain ATCC VR-1470 / WSU 86-1044) OX=716544 GN=nfo PE=3 SV=1

MMQCLKTTKKSAMLLFKKDNNMSKNLLIGAHTSAAGGVFNALLEGKRIGATTIQLFTSNQ

KRWEGKPITEEIKKKWNEALSETGLREIMSHDSYLINLGAPSVDVLEKSRKAFKEELQRC

LDLKITYLNFHPGAALKTDPQECLDRIVESLLETEPMVDGGPTTLLLEATAGQGSAVGYQ

FDQLGYIVKRVEKRIPIGVCIDTCHIFAAGYDIRDQVGWDQTLAEFDDKIGLSYLRALHV

NDSMKDLGSRVDRHQPLGEGKIGMECFKVMMRDPRLKEIPKYLETPGGPDAWIHEIELLR

EFATRGIGS

>tr|D6YVR1|D6YVR1_WADCW tRNA pseudouridine synthase B OS=Waddlia chondrophila (strain ATCC VR-1470 / WSU 86-1044) OX=716544 GN=truB PE=3 SV=1

MKSKTQESLLKLQTTPSSEGILPINKPVGITAFTLVRKLRRLLGVKKIGHAGTLDPFATG

VMVMLIGRNYTKFSDHLMGQEKEYVGRVHLGIVTDTYDSDGVKLQTHSLIPSIEEISSAL

SHFQGVIEQIPPMYSAKKINGQKLYKLARQGEVIERKPVKLHVQTDLIAYEYPYLDIRVT

CSKGTYIRSIAHDLGQSLGCGAHLATLTRTRSGSLTLADCFDGKQLDAPDLDVNELCSKI

LNHDRYLSKNRRF

>tr|D6YWT9|D6YWT9_WADCW 7-cyano-7-deazaguanine synthase OS=Waddlia chondrophila (strain ATCC VR-1470 / WSU 86-1044) OX=716544 GN=queC1 PE=3 SV=1

MKAIVLLSGGIDSTVVLAMAQSLGRECTALSFDYGQRHKVELEHAAHIAHYYKVPQIIIN

ISPACFENTALVNALNVPKNRTLDEISKGGIPSTYVPARNTLFLAYAAGQAEIMNAEEIH

CGPNLLDRNCYPDCRPEFYSAFQQVLASATKQGAEGNPPRIVTPLINWDKNRIVQEGRKL

GAPLDTTFSCYSPSAGKACGMCDACILRQEAL

>tr|D6YS77|D6YS77_WADCW Transposase OS=Waddlia chondrophila (strain ATCC VR-1470 / WSU 86-1044) OX=716544 GN=wcw_1574 PE=4 SV=1

MKILLVAKSTSQHAVSMQFLTKQEREQLQAQHRFERDRRICDRIKAILLYDKGWTFPEIA

EALLLSEGAIRNHIKEYQSHKKLRPEGGGSMEKLSDSESSELEKHLLEYTYLHVTSIVAY

VNVRFGHHYSIPGMTSWLKRHGFSYKKPKLVPGKADKDEQQKWIDAYAKFKANLPGDETI

CFTDGVHPIHNVQLGYGWIKKGVDKLIPANTGRSRLNLTGSIDILSYEVFLQEDKTLNAD

ATIRFFQSLEQHYSGKTRIHVFCDNAPYYRNREVTKYLKNSKIQLHFLPPYSPNLNPIER

LWKWMKETVVYNTYYSDFYEFRQAIFGFFRTLSGLDPGSSLGSCFRSRVGDRFRAMGAPA

TP

>tr|D6YUW8|D6YUW8_WADCW Uncharacterized protein OS=Waddlia chondrophila (strain ATCC VR-1470 / WSU 86-1044) OX=716544 GN=wcw_0559 PE=4 SV=1

MKIPEVIDLSHEVHPNIPTWSGQCGFRITVEMDYDEEGCRVMHYDMESGIGTHMDAPSHF

NPNGENISDLDVSIFVAPLCIIRVDSDSFPDILVTPQNIKDYEAKHGKISPGSLVVADTG

WAKHWNNPSKYRNKTHFPGFSVESAEVLLKRKIVGIGIDTLSPDGRNMNFPVHHLLLKKG

KYIIENIANLPSVPDSGALAIALPPKIRGGTEAPIRLIALVDQ

>tr|D6YUF2|D6YUF2_WADCW Uncharacterized protein OS=Waddlia chondrophila (strain ATCC VR-1470 / WSU 86-1044) OX=716544 GN=wcw_0391 PE=4 SV=1

MKEFFKGFAASAAQVGAACVVKWAIVGAVTFACPPAGAAVSSAMTAYSVGMTAYSVGKCA

YDNYDTLQEIGDAALAGDLGQIAAWGIDAVCSMSYEQLGSLAFDAVSIAAPVARAKGAKA

VADAKAASVAASAEKVAKVAPVVGSTKNSLPTKLDLDALSKAGQEWDRGGLTRAGRALDK

HGNRSGSVFPRAFGNPEFKNVQGQFHLDDILTHPESKFINCGLKGFEIYSPDGRGAYFYK

DGTFRGFLKYRELGD

>tr|D6YTC4|D6YTC4_WADCW Uncharacterized protein OS=Waddlia chondrophila (strain ATCC VR-1470 / WSU 86-1044) OX=716544 GN=wcw_0008 PE=4 SV=1

MTKTTKNVPIPKQVSLLAEAIQCADPEAARSLVKKAFDVMNCNDDLLSGRRIDPIAIDSV

IALIRGVNPQDTVEMILASQFVATHFQAMNKMASDRDTDTSHGMMLMRLSHQALETLQKY

RQKGSNINVNYWVHNEGQAVLQTNIGKNLGKKGE

>tr|D6YW35|D6YW35_WADCW Putative glutaredoxin family protein OS=Waddlia chondrophila (strain ATCC VR-1470 / WSU 86-1044) OX=716544 GN=wcw_0986 PE=4 SV=1

MLKFLNIFFLIFCFATVSAQDFSSKSANPVLYYNPKCPHCKTVMKYLDSQNISVQMKNTS

QASYRDELNRMGQRGVPVLVVNGKAIAGSTSIINYLKQHPDIFSKHY

>tr|D6YVE7|D6YVE7_WADCW Redoxin domain-containing protein OS=Waddlia chondrophila (strain ATCC VR-1470 / WSU 86-1044) OX=716544 GN=wcw_0741 PE=4 SV=1

MPYTLQIGENAPDFMLPATDGETYRLSDFNRFDTLVVFFTCNHCPYVIGSDHVTKQTAEK

YQDQGIGFVAINANSKNTYPEDSFEKMVERMKIHQFPWLYLYDASQDSARAYGALKTPHF

YVFNKNRKLIYTGRGVDSPRDTSKMTVNDLDRVLFEHTAGKEISTPVTNPIGCNIKWEGK

DPKWMPGEACDLI

>tr|D6YSP4|D6YSP4_WADCW Uncharacterized protein OS=Waddlia chondrophila (strain ATCC VR-1470 / WSU 86-1044) OX=716544 GN=wcw_1748 PE=4 SV=1

MSIPPPLQASKWISVQVMVSPSEMRSLLEELGEFTILMTTPAKSPEVSKERFLEIYENYC

TQLQKKELPDPAEYRACFSTFWTRSCDAYVQVPVGEMTLCRAVKPVVQLQYHLLGFSPFD

KKFRPMVLGKGSMPWGIQFSYPTLFQDRETQQVLKVGTEFPNTFLFQAFRKWVRRYTVPT

PFVVDGETINAPVRIGREARQWINVHPQFCEYSIAVREAE

>tr|D6YUS8|D6YUS8_WADCW DUF853 domain-containing protein OS=Waddlia chondrophila (strain ATCC VR-1470 / WSU 86-1044) OX=716544 GN=wcw_0518 PE=4 SV=1

MEQTEQFIKEMEAAYLPKGESITLGAGLLDNKCLKKARVNIPLKTLNRHGLISGATGTGK

TKTLQLLAGALSSKGVPTLLMDIKGDLSGIAKAGESTPRIEERQKTLGIDFKPEQYPVEF

LSLSEEKGVKVRATVSEFGPILLSKILELNDTQASLVSIIFSYCDNHHLPLLDLKDLKKV

IQYMTNEGKEEIESQYGAISNRSMGIILRKIIELEQQGATRFFGERSFEVKDLARIDEEG

KGCISILRLTDIQDKPNLFSTFMLSLLAEIYGSFPEEGDLEKPKLVIFIDEAHLVFEEAS

KALLDQLDSIVKLIRSKGVGMIFCTQLPTDIPSNILSQLGLKIQHALRAFTAKDRKAIKL

TAENYPTSHYYNTTELLTSLGIGEALITALSEKGVPTPLIATLLSAPRSRMGVLTLDEID

EIVSKSHLVQKYNAEIDSESAYEILNAKIKAFNKKEKQDEIQQDFKKAKKEKSMLEKIVD

SSAARQIGRTIAREISRGLLGVFGLGGRRR

>tr|D6YVG1|D6YVG1_WADCW HTH cro/C1-type domain-containing protein OS=Waddlia chondrophila (strain ATCC VR-1470 / WSU 86-1044) OX=716544 GN=wcw_0755 PE=4 SV=1

MTTEKVKSLGELLKQKRKERNLSLKEVENATSIRMNYLQAIEEGDFKQLISPVYAQGFIR

QYAAYLGEDGEAIIRKHPEAFSRVESQEFSYGIGTLEARGNPGAHVKSLPNIIWAILFVF

VAAVAWYLAKFLEVI

>tr|D6YTD4|D6YTD4_WADCW Uncharacterized protein OS=Waddlia chondrophila (strain ATCC VR-1470 / WSU 86-1044) OX=716544 GN=wcw_0018 PE=4 SV=1

MWFDIREASGGHVFVGAHRSCEGMKINHKKVYRIGF

>tr|D6YTF2|D6YTF2_WADCW Uncharacterized protein OS=Waddlia chondrophila (strain ATCC VR-1470 / WSU 86-1044) OX=716544 GN=wcw_0036 PE=4 SV=1

MPKQIQEMMKNLLPLLLIFAVSFNLEGASPPASFTFMIADLKHNDEEGIKICELQAGSLS

AFRGYDWLTGEPGAVSKKVMKMLNQFGHSLWVLSGSISDPSIQKEMKTQKVNVTKNLSEL

YKNKTFIQQANLPVKDPYDLSSYPVLLIASPKRFQNNLESFQEQFPNVLILDLPSYPYWK

NKYEMSLLLRETPELEQIKPHWELFSRAMTSEEIQSSHTHFSSEYVVIKPLNAFLGNGVI

LLERTKLPEILQLIWKSPKKLPDSSDKGYSYWKKARDKHLIVEEFHLSDPVAVSHLKGKE

YDPTLRVVFMLWHEHGEIQMDFVELHWKLPKKSLSEKGSFSEKHKSYGQIPHFALVEPER

EEAIKHQLRETMPLLYAKMLGWEDLNHD

>tr|D6YTB2|D6YTB2_WADCW Uncharacterized protein OS=Waddlia chondrophila (strain ATCC VR-1470 / WSU 86-1044) OX=716544 GN=wcw_1974 PE=4 SV=1

MTTEKPTSSHTTGLEIDEKEIRAVQIKWKKGRLSTGKILSIPLDLEPPNHVKPLYTEKSE

KQLRSLLNKDLIVTGMPASDVMVRNLTVQLSKEKDIDQVLEFQTEPLLPFPVKDAIVDRM

ISGKGLDHTDLTVCAVKNELISEHIAIWHEVGVEPEVISCSQAALSTFSKTFSPIDGLHY

LLHLGREETICLLVNNGILLNSQTIGSGIKDLEEAFAVDSGLIDKKLEKELKNCDANAVT

TTSHPKLFQKIQKLSIEITKCLYALAKQHPAQEIDHLLLTGNSSALINLPDRLNQSLKKK

LHTPKNTKGFSLTPLELQQFALPIGLALQAVSGSSHQINFRQKEYLYPYPWKRIKKPIAI

YFAFCALLSGVILFFGNAYIAYKEDSLRKEYLNLLELMNKPFNEMEAEIKKKTPYIKTFE

NGSDVLLKKMSQSDIAYRIQLIENEIKETPDTFPLLPNTPNVSDFLAWLSNYPHIKPSKE

KNGSDTISLESLSYKMVKRPDINKKRERYQVKVELEFSTPTPRYAREFHDILLAPNEMVD

PNEDVKWSAERGRYRASFFLKDRTLYPTGKRK

>tr|D6YSF6|D6YSF6_WADCW Uncharacterized protein OS=Waddlia chondrophila (strain ATCC VR-1470 / WSU 86-1044) OX=716544 GN=wcw_1656 PE=4 SV=1

MTVANEGGLGGVDPHRFIESSEDAKDEAKIADAKDKVNALPELEELSEEEEIHMIADDRE

IPRPSPQDGEQRTLQATVDRGGMTGSDVAIGGSVMASAVIAANNQEAEVGSCRGHVDRGD

DGSIRITVTDPEGVTREGGIDEDGHQYLRTDLEDVGPSTVRFSEVDDGEMTVSVEPDDGS

GPHELTLEVDEEAEDRSKFNKEDLDKAEDDEQSWVQMLEIVNSVMKHMEEWIAQGWEKGK

PNI

>tr|D6YTK4|D6YTK4_WADCW Putative Chloramphenicol 3-O phosphotransferase OS=Waddlia chondrophila (strain ATCC VR-1470 / WSU 86-1044) OX=716544 GN=wcw_0090 PE=4 SV=1

MKGAIIILDGSPSVGKSTIAKEIQEIAEEPYYYLPIDEFVNKLPSRWIHFTDDLTDIEGI

GFHPTTDSEGNAEIKFKAGPLGEKLIRGYINAVKGFSKAGNHVVADAIITDLKWLDWLTK

QLSQYPVVFIGLHAPLKVLEQREKERFELQGTARGRYQEVYGMKKPYDLEIDTSLISPKE

AAVKILNKSKKS

>tr|D6YRH5|D6YRH5_WADCW Multiple resistance and pH homeostasis system, subunit G OS=Waddlia chondrophila (strain ATCC VR-1470 / WSU 86-1044) OX=716544 GN=mrpG PE=4 SV=1

MMTDWVSGFLLMSGSFFMLVASIGVVKFPDVYARIHAITKATSLGMILLLAAAVVLFPMP

LVFLEVVATLLFIVMTAPVGSHMISRIAYKMKVSKKGMPDIGDEVDS

>tr|D6YU17|D6YU17_WADCW Uncharacterized protein OS=Waddlia chondrophila (strain ATCC VR-1470 / WSU 86-1044) OX=716544 GN=wcw_0253 PE=4 SV=1

MQERQIYLEMTPILKEMEIMKISVDLKQREVYLEEGALLLEEILTSDQQEKIFKEIEMVQ

STSGRDLWRKNHRLRQLPVLRPVVNVASQLLGENLIRLGYDQYLPHLNGIWMDWVHGKAS

LRDLGSYRGEVCSVLLPLTGRGDALYVRPEWKLDLAEWVDTAKPHYLVLYSTLKAQYTLN

KKDPAAAEMVRLGYNNGDRLREEINPVVHR

>tr|D6YS20|D6YS20_WADCW Uncharacterized protein OS=Waddlia chondrophila (strain ATCC VR-1470 / WSU 86-1044) OX=716544 GN=wcw_1516 PE=4 SV=1

MNRSIFLTLVAALFMLGQLAAVSANSMFLAQPKHIFVNNRILAKINGQPISVIDVMKQMD

MFFYRQFPQYANSPEARYQFYNMSWKRALEDLIDKELIKADAKEVNLPMTHGEVRQEIEN

IFGPNIIANLDKAGLTYEEAFDLTKEDILFKKMMMARVNSIVLRSITPQMVVDHYKTWAE

QNSQPEKWIYQVISVRGGSNEASSTIANTVHHLLRDKDVELSQLKSLLHPDEFKLCSVSQ

EYSLSPSEISDSYREILSLLKPGAFSEPIAQKSRASKTDVYRIFYLKEIEKGGAPSFYEI

QNRLRDQLVEKEMATEQAKYLSRLHHHFKVHIDDILNEIPDDFKPFTLK

>tr|D6YV90|D6YV90_WADCW tRNA(Ile)-lysidine synthase OS=Waddlia chondrophila (strain ATCC VR-1470 / WSU 86-1044) OX=716544 GN=tilS PE=3 SV=1

MNLYTSHFLPFLKRCTPFNAPVLIACSGGPDSMALLRMMVEYRKTHSVRFGVAHVDHRWR

KESADEAETLRALCREIDVPFHLKEIDPEAMQGNLEEACRHFRQTYFLQLCFDHGYGSVM

LGHHLDDQAETVLKRVFEGAKLEKCGGMQEISVYETIPFWRPFLQVRKQKLIAWLNLRNF

PYFIDPTNNDSSFLRSKIRGNILPEISRYFGKEVSPGLAFLGREAHALKEFMGSHIYKWL

ALKEKTPWGTVLDLSCQNPSHLFEARCLISELLPQASREIAYSAAENLIGGAANKCYHAG

GKTLYIDRKRMFLTERLHADLPHQETSLKKCGSIGSWNYRIQSASNKRLSGWKQVLYGVL

QWPVGGNFEKLRIGSPATVHCQDELNRMRRQARVPVFMKNWAPVITRENEIVQDFLSGKV

EQQAADAPLEVVLTKAMD

>tr|D6YWL3|D6YWL3_WADCW Uncharacterized protein OS=Waddlia chondrophila (strain ATCC VR-1470 / WSU 86-1044) OX=716544 GN=wcw_1167 PE=4 SV=1

MKKILFFFLFPLSLLQGIDIDIENPSYENGKLTTEDGGVITAPNFRLQALRICYDSSNEQ

SLLEAEKNIILEFGEYFFVGKQLVYDFKTKTGVLYHGRTAVDQWYIGGEEIHLCADGSFY

IRNGFATTSENARMDWQLETGDAHLKDHHMLTAKDVKFRFIALPIFWMPKIHLDLNALFD

PPVRFNIKVGGTRGSKVGFIYKLYSTPYWQTNLKVDYRVKSGFGGGIESYYRSPCKCTSF

SSINYISDDKTRFDSKTRTRYRLEGNYHTQLNHGKTTVDATWDKLSDYEMASDYDDADLY

LVLANPTQLYIRHQESNWIASILTRVRINQFQTLKQELPTLYGAYRPFEIGSTGILSAGK

FEASYLELEYTNNSEPDINDYNSPRYEASQSFWKPLKAGAFTMTPEVGMTAIYYGNSKKR

SDRWMAIGQFELDMRADLYRFYGDCKHVLTPYLRYQYYTFPTTSPDDHFIFDINDGWYRL

NALRLGLLNNFYCKSSPCCIYRKLYLDLYTYAFFDTKTIEEIIPKTYARVIYNFTDRMRH

SNLFVWDFQHQIVDQFNSLLEWTINTTMAVALEYRHRSPYAWRKVDATNFILDSFRSVDD

LEKTLLSDRRDTFLYHFYWKFHPTWAFAFQSRNGWNRKDEVGYKEFQVTLLGTFRSSWNV

KMDYKHREHDDRFSIHFSVGLNRPCYDYNACRVPCLEF

>tr|D6YWJ4|D6YWJ4_WADCW L-threonine 3-dehydrogenase OS=Waddlia chondrophila (strain ATCC VR-1470 / WSU 86-1044) OX=716544 GN=tdh PE=3 SV=1

MKSILKKKPEVGLWMEDSPFPTCGDHEVLIKIRKTSICGTDLHIYQWDQWAQKNVPVPLI

VGHEFVGDIVEVGKAVKGLSIGDRVSGEGHITCGHCPNCRKGLKHLCINTRGIGYHKTGC

FAEYFALPAENVFVLPEKVEDRIAAIFDPFGNAVHTALTFNLTGENVLITGAGPIGVMAV

AIAKKAGARNIIITDINDYRLDLAKKVGADAAVNVLNTSLEDAMAALEIRHGFTVCLEMS

GNPQAFATLLHHARHGANIALLGILPPNTVIDWDLVIFKMLTIKGIYGREIFKTWFQMVD

LVASGLDLAPIITHEFDVDDFEKGFEAMQSGNCGKVILNW

>tr|D6YRI9|D6YRI9_WADCW YkuD domain-containing protein OS=Waddlia chondrophila (strain ATCC VR-1470 / WSU 86-1044) OX=716544 GN=wcw_1332 PE=4 SV=1

MSFPKLLAIITVLLFGSIAIAAIFKKKEISDPLAETEIVLAPVSIEEDPLVTDAVKEEKS

LELLAAASTEETAQEVPEADRIDEFFNTRGQKFPIVETITYKKKVPWLKGRAAWIADYAS

HYKTSRHFIARSLNGKPLYDKQDVVDGDRFNVFKEDKNLSFYLLVDLQRSKMWFYYLDEE

SNDRVLVKTYQVGLGRLEKDYESGSLTPLGKYSLGEKVAVYRPKTMGFHQGDKVEMVRIF

GTRWIPFEEELDHCTAPAKGFGIHGLPLMPNAKGELVENSDTLGKYESDGCIRLATKDME

ELFSVIITKPAVIEIVKNFHDAKLPGKESRG

>tr|D6YW14|D6YW14_WADCW Putative sodium-dependent transporter OS=Waddlia chondrophila (strain ATCC VR-1470 / WSU 86-1044) OX=716544 GN=wcw_0965 PE=4 SV=1

MRKRAHWGSRVGFILAVAGSAVGLANIWRLPYVVGENGGAAFLIVYLICLALIGFPVFMS

EIVIGRTTQLSPGKAFHQIGGSRFWSWIGYGTIITGFLVSSFYSVVAGWILGYLVESIRG

NITSLSYASEAIDHYNGLIENPFWGVGFHALFLVICVGVLFLGVKRGIEQGNKVMMPSLL

IILILLVIKGVSLPNASEGIRYLLSPDWSELTPKAIMIALGQAFFTLSLGQGTMVTYGSY

LNKEENLVTSCFPVVIVDTGVSLLAAIAVFSIVFAGGMQPDSGPGLIFHTLPLIFSQIPG

GYLFAVMFFLLVLLAAITSQISAMEPTIAYFQDELGWGRHLSTTVCGALVFLAGIPSALS

YSMMRSCTFFDCNFLDFMSMLCSSFLIPLGGFFAVILVGWVWGTNNAIAQIKQGASDLFQ

RKPWLKTYFRFCFKYAAPILMLFVFLNALGLFL

>tr|D6YRW0|D6YRW0_WADCW zf-RING_7 domain-containing protein OS=Waddlia chondrophila (strain ATCC VR-1470 / WSU 86-1044) OX=716544 GN=wcw_1456 PE=4 SV=1

MHEALKDILPIQEFDMQMIQLMKLKKERTKELNHINGIKSDLRQKTMIKEGEIIELRKNI

RLLEGEVKEVAEKIKKLEGQQSQVRKVDEFNALSHEISQAERERVAKDQRLSDLYDQVAA

EEEALKSLNENLELTVENSKVLENEIKESIERINEEGKAIQKQRDELVGSADPELFRIYE

MLLKNKKDRVVVPIENRCCSGCHITLTAQHENLVRKGERLVFCEHCSRIHFWQESEALEG

TAVATKTRRRRRTTTKST

>tr|D6YUX1|D6YUX1_WADCW Uncharacterized protein OS=Waddlia chondrophila (strain ATCC VR-1470 / WSU 86-1044) OX=716544 GN=wcw_0562 PE=4 SV=1

MKTYTLSLEITTPAADQSSFLHVSHLSEPKRVHPIWKVGLAGTLGSPVRFYIDEPRVLHL

SGHLPAALYRDLTPFSAHTVHLFEDKASIWSLSFPPAFEEIDHTADIAFAVKGEDLEQLH

YHALAALAFYEPQMIPFIPIPRSSADLDTIIIHLNSLITKIDTESGCAFKAVSFHGRIKE

MQGFLQWEMMIDV

>tr|D6YWN0|D6YWN0_WADCW TRAM domain-containing protein OS=Waddlia chondrophila (strain ATCC VR-1470 / WSU 86-1044) OX=716544 GN=wcw_1184 PE=4 SV=1

MMNACTIFFKLIFSFLSVLFFTLFTATTFSYGFTLYSAVIGVAGGALFSMLVFASDIVFK

RVSLKQMNVLTLGLLFGYLLGQSITLILTAVIDLAALTVWPETIALTKAGIFLFCVYFGI

TFTAKASEEIHASIPFIKFKPSAQRKKDYILDFTALCDTRLIDLAAAGLLDNHLILPRFI

LKKIYEKAESQQDPANVKARRALEVIKKLEELPELHLKTVETDFPEIKEIQGKLVRLARL

LDAYMLTAEANQIEQSSVEGIRIINMHNLAKALKPLTQTGEFISIKVQRYGKEARQGVGY

LEDGTMVVINGGAEFIGEMIKAQVLSVKHTSSGRMIFCNAFEDEEGYAEERMAIPDKDDS

SAKKYFAL

>tr|D6YUL2|D6YUL2_WADCW Metallo-beta-lactamase superfamily protein OS=Waddlia chondrophila (strain ATCC VR-1470 / WSU 86-1044) OX=716544 GN=wcw_0451 PE=4 SV=1

MVKTMKFFAIEGNRQHLDGGAMFGNAPKAMWEKWVYPDDLNRIPLATRALLVRLDDGRNV

LFEVGVGAFFDPELKKRFGIEPEEDLLLKNLNQSGVGEADIDIVVLSHLHFDHAGGLLPE

YGDDPPKLKFPNAVYYVGAEHWEYAQNPHPRERASFIPLLHELLHASDRLVLIDKPEHPD

LNFGVTFRFSEGHTRGMMLSQLATDDGPLIFVTDLIPGNAWVHLPLTMGYDRFPELKVDE

KRELYESLLEKGARFFYTHDPNIACAALKQNEEGRYFGEPVEL

>tr|D6YSL9|D6YSL9_WADCW Uncharacterized protein OS=Waddlia chondrophila (strain ATCC VR-1470 / WSU 86-1044) OX=716544 GN=wcw_1723 PE=4 SV=1

MFKPKARPFPPGTFVPTPLRVVSIIHLCIAFSALLFDLGYPFMGKLFEDKRLNSIYESVI

AESALYQQLSFEKQTEIQAAHQALLNRMHQPFSTKFDHAMRILLIELPPFQKAWIFFSII

IPILILMKIDGAHRAAWILPIITLVFIANQFTFPTVPEKWKENALFPTEQMLLDQYLGEP

LANSISDQREQLLKGWHLYLIKEWSKEEPSQTESDFKRQLSKGLFAFNLARIDAIQQDSL

FPSYLRNKEPFLLVMFLLWNLFFAWFANRRKWFIL

>tr|D6YVP6|D6YVP6_WADCW Uncharacterized protein OS=Waddlia chondrophila (strain ATCC VR-1470 / WSU 86-1044) OX=716544 GN=wcw_0841 PE=4 SV=1

MIQFRLKKHIFIFIMNPSQYSNITFERSRESAVSENGVRKQAFERFTEWFVSLMREFLTS

IEELFAYSEQEWIPLKDSQFQEILEHIRKSHLQCFVLPEDYVLGKKSPEKLTRQERKALL

DRVKEVKRDFYHSCMDNVSGIPSNLDGNTAGNSLLAFLFDLHYLKKDHPDDTSLHEMIKT

FSETYKFVVEREAVERLVNKKEQAFRKQEYIGKVEAKLRSLNPGERFVYQMSVSHHAVLF

EFKLKEVEGKQVLDIKLLNSGDGVDHHYSKSFFADLNPFAKFQTYLIEGVEKDTFLSSDF

VSKLIELEVPGEVKGRHIPVFGYLFSLVDSIHHMFFGVGKIYRLLNVHAIHDGCGKKVIS

DDPRLHHYTQSKGTCSRRIYEYWMRENLASDSEFQNYLTKTAEYGVARLKMAEALENHLK

GRSVKVDSLKDRLFVQPHWFSKGVACLRNSLKTRTMILIGEKIVQKRQSGSLDC

>tr|D6YV08|D6YV08_WADCW Uncharacterized protein OS=Waddlia chondrophila (strain ATCC VR-1470 / WSU 86-1044) OX=716544 GN=wcw_0601 PE=4 SV=1

METAMDFPTTATITAIATIIRNLIPCLLAVSCQSSPPSWKVSETKSRHPSYQACSISQKP

DNLYRGMELEFVRTSSRTHAYLNVFSLTFPSDRLDENKTKVVIKIEDEEQIFLAHRLEGG

QRLLLPEDARDLLVQALLENKPVAMIAGRFDVKISPGNFSKKHCQKINLYEKR

>tr|D6YSX2|D6YSX2_WADCW Acriflavine resistance protein E OS=Waddlia chondrophila (strain ATCC VR-1470 / WSU 86-1044) OX=716544 GN=hlyD PE=3 SV=1

MNKWILPVFALLSSCGSGNVQPRHPPPVDVNVMTIETVDAPVVYQFVGQAKSSLQVEIRA

RVDGFLDKLAYEEGEMVEKGQILFELDKKPYEAALQKAKGELALQQARLDTASANLKRIR

PLAEQDAVSKKDLDDAIGSEKAAQAAVLAAAGTVDEAQLNLGYATIYSPLKGLASKTDKQ

VGSYIPTGQDSLLTYVAQLDPIWINFSISENQALQFNQDVASGTIIPPEEMKFEVEVILA

NGTSHPYWGTITFSEPNIDPNTGTFLIRAELKNPEGNMRPGQFVRVNLHGAKRPNAILVP

QKAVVQGSKGHFVWVVGKDNHPQVRSVEVGPWQGNNWFIEQGLQPGDLVIIDNLMKLNPE

KPIKVNMGS

>tr|D6YVS0|D6YVS0_WADCW Uncharacterized protein OS=Waddlia chondrophila (strain ATCC VR-1470 / WSU 86-1044) OX=716544 GN=wcw_0865 PE=4 SV=1

MFPAPKNIENIANPVDRVLDFIFVDMILLSLKF

>tr|D6YTS7|D6YTS7_WADCW GtrA domain-containing protein OS=Waddlia chondrophila (strain ATCC VR-1470 / WSU 86-1044) OX=716544 GN=wcw_0163 PE=3 SV=1

MRHHFRENQAFWGDVVRFCIAGALSVSMQYFVLVTLVEVFHLNPTFSSATGFISGCVVNY

LLLYFWAFMSSARHHVALIRYLMVMSGSMTINVVIFWALTERIGMWYPISQFFATCSSSC

FSFLANRHFTFADRSGR

>tr|D6YTI9|D6YTI9_WADCW Proton-translocating NAD(P)(+) transhydrogenase OS=Waddlia chondrophila (strain ATCC VR-1470 / WSU 86-1044) OX=716544 GN=pntA1 PE=4 SV=1

MESIGLLLFVFVLAVFTGFELISKVPSQLHTPLMSGSNAISGITIVGAIIATGYLGDSLF

AIVLGLLAVIFATINVVGGYLVTDRMLKIFKHKNQK

>tr|D6YTM7|D6YTM7_WADCW Uncharacterized protein OS=Waddlia chondrophila (strain ATCC VR-1470 / WSU 86-1044) OX=716544 GN=wcw_0113 PE=4 SV=1

MIRMTFLISLFLAASLMASADEKEVKYEEDIIIQEPECLQLMTVGRGSGSDYVIKFYNSC

PKRIWASICVEERPGKFKLHESSTRIPKYGYMDIYTYEGTAPVSVNWISGSSRPFRPPGQ

CGTDVEK

>tr|D6YVQ2|D6YVQ2_WADCW Putative type III secretion chaperone SycE OS=Waddlia chondrophila (strain ATCC VR-1470 / WSU 86-1044) OX=716544 GN=wcw_0847 PE=4 SV=1

MVEDLFESLLQEMGKAMNIPDLHADSNNSCLIAFDTGIEVQIEPYERGEFLLIVCDLGEV

PPGRYREDVFREALKSNGLPHPRPGIFAYSEQSNHLIFFGLLSLKELNGEKIASFMYPFM

EKAAAWKNTLERGDVPLADTMTTSHAAAPGGLFGLRP

>tr|D6YS66|D6YS66_WADCW FlaA1, SDR superfamily member with inverting 4,6-dehydratase activity OS=Waddlia chondrophila (strain ATCC VR-1470 / WSU 86-1044) OX=716544 GN=flaA1 PE=3 SV=1

MEHFKDKRILITGGTGSFGRTCAKKLIINSKCKKIIIFSRDEWKQWEMKQSDPVFEDHRI

RFFLGDIRDHTRLMRAFNEVDIVIHTAALKQVPAAEYNPTEFVQTNVIGAMNIINAAIDC

KVRQVIALSTDKAVNPINLYGATKLCADKLFVAGNAYVGTQGFPCFSVVRYGNVLASRGS

IVPFWKRLIDEGAKSLPITDSRMTRFWITLDQAVDFVIQCSSLARGGEIFVPKIPSMKIT

DLADAMAPNLPQENIGIREGEKLHELMINEENSRHTLEFNDHYLIVPEIFMNTAKLLEKF

LSGREGSPLPEGFSYRSDTNTKWLSAEELRLLLSTIL

>tr|D6YRU4|D6YRU4_WADCW Uncharacterized protein OS=Waddlia chondrophila (strain ATCC VR-1470 / WSU 86-1044) OX=716544 GN=wcw_1440 PE=4 SV=1

MKIASYFCPFSHKFQAREDFFQLTRWEKLKTITVTALAALATLPLLGLGGIAVFRAMVRH

YAPINLRKPSDSDSPQESSASKVDTIVQPVFGKELKSERYLQARKQLVQCNKELETLAKP

PLGVEGMLETIKNIEKDIHAFMHEASSMKNESDLALMQEMGDRLVEVSKAIEEKLELESD

ESELNQALEIIEALIEELPKKEEVESTKTFWERLGGAFGQSQEVKDLAWKVIKYETQVLE

LKKHIEKAFPFVLQDKDLVSSRLFVGNHRDAVQRLYDILCEVEIDTTDDSRFSQDYLGQV

RSLINEQFIPVLRKELKSDSENSSLHQIAEIMFTHVSLEKQCFDTLALFNQMGADALGQL

PEEVDDTVHGEHPFVKWINTIYHRISAVPYSAKAPLFNQWANSARGQWNRNFDPYRQGNP

THHFWSLRVGKKEVKMLGMGTPTNEDSWSQAHMNEEFIALMKRYKTVGKKHLFINNQNLI

PKTGWTASLINGDETARCKLILDKQDDEDVKGAYFAIALSKNSTFYEQKGEWKERDLAET

FKGNVFEQVCKGKRHITGSYIPETIRELIPEFDEKARNVIEQIHEKVFEGKENLSVEERR

WFIELFYDNLVKMIIVEGDFDSVNGSCKDQIDRGAGTNAQLAANCYIVSQKDGKLTSEQQ

GHVLMLMMVRALLVRKRPPLTERVERFVEGMEMSFKHVEQLKALHKTLFLKTPFVPLRVG

GE

>tr|D6YU21|D6YU21_WADCW Uncharacterized protein OS=Waddlia chondrophila (strain ATCC VR-1470 / WSU 86-1044) OX=716544 GN=wcw_0257 PE=4 SV=1

MSAIASPVCVQSDPIFKSRSFLSKHRKWCVRVYRHHSDLIASLGIFTLNTILLASKIFKE

IPHLIHRSCYTALSFSGMIWMNIQIRDLIKNSDDLYHNLKAKDWEGIIFTAAKVAVKSLN

ILLTGALLTASIFSLFAFPEIAFAMYAAMRPFSLFSLGTGILTEVYDYKKNSSLCARIKK

VSEKQEVMHHFLKRLYHEKDLAQESSNKLLARHVFKQLDHYVMESMRKNLANKYGCETAD

HLMNKLDENDLNALFQSLKRGLKQKQQYIQANLGLITAGYISMGICKMWPDSLIQSSVTW

IMSLLYTSKMIWQKSLHSGWSAKT

>tr|D6YSI6|D6YSI6_WADCW Putative membrane protein OS=Waddlia chondrophila (strain ATCC VR-1470 / WSU 86-1044) OX=716544 GN=wcw_1687 PE=3 SV=1

MESLILFATEHAEAAHWFFALLILLGGLNVPISEDVVMISAGALASTLSPEIAAYQYSAL

FFVCWFASWETYWIGRLLGPRLYDIRWFSRILTPKKIEKLHHYYERFGIWTFIIGRFIPG

GVRNGLFMTAGLGRMPFKKFITRDLPACFLSTLFLFALGYFFGQHSQEVIATFHAYHRSI

LFFIAATLFIYAILRYLRSKQIIIDDFYRHLQ

>tr|D6YVB0|D6YVB0_WADCW Uncharacterized protein OS=Waddlia chondrophila (strain ATCC VR-1470 / WSU 86-1044) OX=716544 GN=wcw_0704 PE=4 SV=1

MSLGEFEYNPRREELLKQTESEKPCKIRYESVEQSGKRKNLSLTQKEFIASVINNNVSLS

TNKDHHMRIEVLGEYEFSVVIDHADKFHIVKREKEEDSLVVVQERIKTKSFVNSIRDFFQ

AIANLFYQPTYPSKELKQRTHRETVKQDFRALRKSLENGKQWLSSAGSIRKGEKKELEKE

IGGLIINAKKIEHALDSKNQTKALDELSERFAQQIFDLNKESSFTLATGYRNQDGALQPV

MLHFSNTKEGGLILEIYADTHEGKGKIAPLHRRKFHSNSVTQPHIKAVLDEAFKPLISPK

THTKALKKAVKPSNTFGAITRSQIEPRLQEEIKKVSKRKETAEERFESPQEERVTSLTFE

RFMRSVDTSMKGTWDSVEIGEQKMKRQPETPSERVAGWFNHLLGEQKLHKNEKLNLLFFL

TEDYAQDQMRQLERKPPRFRKPSPEKQLAVYKNVAEQIAHMRERLAVSINGDHMLDTRGI

PQSLKNTEELCQKKIAHLESKIRDSKRAGMSKILSKTQSSKLSGITVGSVNLEPPVDPAA

TMMDASLAVNLDEAEWMKEWQSKARSLEELLSPEKMVQQRNWQIVREFNDVKKRSIQRFE

DAQDQLEELKEEAKDYDDLEPEKKDELIQKIMNSPVPLSEKDKKTLEIPNNLNADSIQEI

IENLISKSENLLESLEISSLIKEDKVDEESLDKIIQAIFGSMDKEALAFLGTSRQAGINQ

DQLASIIRQLIICPQMLPTIDTPNGKSILLQEYLEVIYRILDPQLASGSPYASITDDLRA

LINIRHLLPRKKMGENYQKLWEEQLELKKKKSEEKVEHKTADLIKATKENVDTFSKKLEE

LNQSEERNPQEIAKTEKALREYEMMLKILEPLSEEKGIFKKFEKLKTQIESIKEEVKDTD

QEEIFKEILTNLKNSEGLYTFLEIEKQFLKMIDSHPAIPETLTPSNLEEIDESPAPDLEK

LEPILEAFGETVNAMAAHLQQLSKDISREPDPFKKQAMYEEIKTRSLELLAMLPPPGTDG

AHGTPSIWRSLTAEQRVEMRRNVQQLEHHIWEAQMRLSKTDLSGKEKFLMIKGQAIGMAL

IREDVKEEQKKIEKFLTDLYTKTPEEFKTFANMEGIDVLESEGRVIINLEKLDPKTIKDL

ARFIAETREQFSWVEDLNNAKIQDRDKDSMIDLLKQCLIKNNQDSFKQNGINWNADKKEL

IISWNEKELDWGFMKKVLENNTILMGDLKINPLLLLLDARTLDTHGLNCVLTQDLTTCLS

LDPKLEREVQAVYHFVVTDQNRGKSDRIYRQNNKATRSPLLRQSATVVVNPLLRELSLLN

ILSNGNKRQVESKIKELQKTHNIDVSKLKAIKWDEKNKNELLIDWDNPELEKDLAAFAEH

DSQIKMALDFYTAVNQAAKNELYFNTASDPDFTLYAEESEEPSGRSFFQKRPNLLIGKQS

PIDLHIHPSQQGLYTIKDESGKVAAEVHIYGKQGESDRRSFGAIEEEINEDQLHLAMKPK

KPFLPTTNQPDPFGDPDGFGSNEDVREIRVLKSYDAWSEYVKEIAGGQVLSIPTQVLGKL

FQIRQAPNDSRQGIVVASYSSDTALLALDFLTDISNQPHIQHDFVQNFLLESLFGPMVLQ

QSLIDHPEAFISHIKSLEQAILQAQTSRNHEMLGFYTLILNRLEGHINEARNQLQNQGVM

AGSLSKLPVFRDKHGLGAIYRHVQQATELTHVINREDLDTEAIIQPLTAKLYFLELCHME

ISSIERDSAITVKNILLSGEKGQRPIDKITNPEAKRRAYTHLLQYYYSMGIGSLDDDDYL

EILKGYELLQSPSTEAASSLISLEQEAASWIRQDVLPAIQSMEEDKRDGLLKQIVNAKLK

AEKKDPLSDEARFKATEGKINQFELSTGVQEITIHLSTMEIGGIELTKETLGSAAIPEKI

LKRKDVQQALGTSIIKGKITKTERGKTSTYTWEHQGQEFTLRTDGFKVDIKRKISSHSTL

PSDEYTFYPVQLENIETHAEHLLNANGIWKIDKQPKIGYVFGSGMELPTTETIYQATMND

TNNISFLSTLGGARVGSATRLNGPSPLLFADTKHTLILVDPQTGRPNELRIPGLNVAMRK

TENRWQLSQSGKPLGMLKVPGRDEEKFLRTAFGEHWDQFVVPVEREVDKIFMKKGEKITT

KRTETVYLMIPYRQQVDRKGQLSVDQSMLDTIPGMHELRMTEEGKVQGTMAANVYLAHRF

LLQAGKTSNPTIARNLMMKAQEHLERVQGQPAPSNPQEVENLRFVLAEIAATPAVSLSPP

PTLEALSMALKLELFVQHIRSQAVKQKIKTLQSGSEQELNELFKIAQMYQAYTVLEETAA

KKGKLHRDMFALTDKEDAALIGINKRMLQGLSSAMENQELYFGTTGRLATTVSLDRPKTI

DPQFLLALLRMAKPRNPEITLKGVNAPLPAANLIENFWSYLTSIKTNRLTTEDLMFLFEE

SILPPTDSPEQEQQLRELDLQARQFLLTMADLIEQAGGSTDIMGDAEKFLEESKAHILTT

EQKDLGSLLSNYKGMTGIKTNFDSGLKGIEELKIDPVPDTDPPIPDLDKLIYDLNNKVKT

PLGNFKDQLEDALNIANKQISEIRARDEKLQEEIFTAKKSLEKAIDDHKEGVDELGEEEY

EEKIHKLQEAVKNAIKEHLNYTEEEILDPLTGEKLPRSAFVSNEYAESVFLEDLKKVAEQ

TRSSIEKLDDAIQEIGEKAGLLKSWNELEECMRDIETVPFKRNDWFDMPEAGAAKAFIEY

QLQQTGEEPSLFSAGISLIRQLGPVTAIRLARSTSEYETLSTRLKETDKELRTDNLSSER

KKELQGKRAKIMQKLLSNPQGNLEAAGMALGSLVTAGAILDFKPIEYKPETIKKVGEQTV

SEEILNDPFFKKCFTDQEREQIASNLSRMGGYEKRTYVKQLQAALNMQASLLNASLGLQS

RIESINKSLGKVLKGTHEGKSDPTPHSFKGITGTSLEQRYADHYTSKTGFVAKEKLAALF

KKLEETTKRNEDTLDFLKNRAENLADPLFSISLEEVNDTINEIRKRIQPQPIPIEIEPYI

DALALIGKAAVYKIDGAKNFADQIRKISTGEELLLAIQTAHDQLAPLVAKHEHEALISEI

IDQTMPKDSHYADEYSKGLERIEKNNPLAYSTVIGEDQVEDVEELIATDVAQLQEQVKQQ

QDEIIRRLEAVSLGNLPKSLQKIRMRRGSSQELLEAAYKEYRKGSFQESKPTIPDGAFKE

LDLDTLIGKTLFDDTRRQVLQGSRFNAFTSIQNLYVLREEKKELMRLRAIASQYTKEKLE

NKIKKFVELAKESTSKQENEELRKEIEGLRNALLHYDDFNTLLARIESKWQKESDKIRDF

TGRCQSQPHLQKDLGSLAPYTRHINYLQQRLGLVLREDQVQTLQEIIDNPALLKQLRMGL

GKTSVILPFALMILSAKGYNAIGMVPKALFDTNFDEMDETTRAVFEMAGSRFLFSRQDAN

FPLDKLSLNELSTKCAEFFDALNREEYILTTIESKASLDNKIIEVERSQAFIQSRINDIK

ENEDISEEDKQKRIEAFYPSLYNHQMALDMLYRVKAVFEAKHTRLIIDEVDSVAKSNKTV

NSEIGRKDPIAPAIQNVASEIFDIIRENPELEGLLEQIQSNNQFTLNESQVNDFIKKIGE

VLIQNIDLQGHKKDEVLNWLTGETACPFDAKELKNLGAAADKLKIQRKALNSALRSSLAL

KAGLSGDFDPTHGAIGVPASQGVTSKTTKYSDPLMQVVLTQMIALYKPQGEAYLNAVAKE

VIADMKSDLKIVEGQLKEASSDSLKQKRDELKEGIKKLSKLAIPEILRNELKQELAAKEG

SVGSDLEKEIENLEEYIDTLSPDSPPSYQNAIKGSDPLNTFLRLRFARAAAKRGMIYVSN

NEVSRPNQHALRGCHVIGLTGTATENTSHVITGTGHQEAMKGITETGRDSTAEVVYRFVK

ALKKGSETPAEILKRPVETYSLDTKKALEQFINIAQKESGYRFLINQAGACDELTLREIT

QQLHVRCERPIVYLDMDEEGRTEKVALINGVRRPLKDLSREEKQQVEEEGFTYYHTPHVR

GTHFDLPGSKGAIMLSPTVNANDRDQAFYRARGLGEEHTVVPFISEKQSAEFKEENGEEM

KLRDLLKIQHEQTREEESQEDLAAYVLHIKGQVILAADRAKERIQLKDGSIKSFSDWTEA

DQEKITARAKVNEIVERLFIRETGNSAYLSSLNSETRQGGTMPTNEYLTTVVIEQELARI

DRFLKEIDEIKTENDRELKTALDIVTQELSQAKKKLEEEQKNIEENWEETLSRKLPTETS

SAPATQETSETEAEAEAEAVAETTSESTSDTERKKRTVQTRNILTVPDHDLLNSLQQSPK

KFMTYQYLPDFRNTHEGWKSGIRITENLKHAIEIAGGQGKGIEIKALIIDEPKEGEQAII

LSNAADIDNLIGHMSILLNSSSRETSGFSVKPWFNEEGEMELQYCDTSLTGYSLRDEFDE

KKELKGEIYLSFIHLGFSPISEKGWEDIGAYWTSLDKDAQDEVKNSLEKTLTSSNPSLLD

SAAKHMWNKPKSEAISLGGSKASVDKISDIDEAKKQIKEAVEDPYAKTKYNAAQKWIDRN

ADDLIKEELEKWAGEEY

>tr|D6YUU2|D6YUU2_WADCW Putative SAM-dependent methyltransferase OS=Waddlia chondrophila (strain ATCC VR-1470 / WSU 86-1044) OX=716544 GN=wcw_0532 PE=4 SV=1

MDSTEKKCRHGCFLMTEKNKIHQGNVIDTIDGIDVIECTVCGFKHVNPLPSQEELETLYK

KEYYSLDKPQYLKDSEEDQEWWEMTYRQYFHLFDKHLKKEAPSLFEIGSGPGFFLKVGKE

HGWDVLGIEPSKQAVAFSKQFDVPVIHDFFHEETAKKLGTFDLIFMDTLLEHVPNPSSMI

ALCSSLLNPGGLLCVISPNDYNPLQKILRNQKNYPPWWIVPRHHLNYFDFTSIQSLFKQH

QLQVVESLGTFPMEFFLLCDDNYIENHALGREVHGKRKTLEKLMLANNPKLLESFYQWLG

MQGLGRSFVVIAKKPS

>tr|D6YSK5|D6YSK5_WADCW Uncharacterized protein OS=Waddlia chondrophila (strain ATCC VR-1470 / WSU 86-1044) OX=716544 GN=wcw_1706 PE=4 SV=1

MTKVIISEEAKSLLDKFLEENRPAPLVNTYLFYIENKFNLQPVLFPKEKMIYQSAEAAIR

RLEEEEKIWHQTEIKITFGETSVNEQTAKIYICPFTGKVFGDNTHPNPQDAIYDWVSKCP

ENTERSGGLRVKRFFVSEDPEVIKNYMNKEQRKDPITKTVYSSVLSGKLFNSKEAVIKDF

RKNYLKKLTLVEVQNQNKFSIEESFLEFIQQQLVEDKITGFVEAMAEVPEFLPVVESWLA

>tr|D6YVJ6|D6YVJ6_WADCW Putative Fe-S-cluster redox enzyme, Cfr family OS=Waddlia chondrophila (strain ATCC VR-1470 / WSU 86-1044) OX=716544 GN=wcw_0790 PE=4 SV=1

MIPILSHTRESYSQAVAKALGKGLQHAALLYREWMRTGNVSSKHPAFYNAPLLYEEILQV

TDFSLPEISHQLTDGDVRKLLIRFDDGNVVESVVIPMQFGLSLCVSSQAGCRMGCTFCQT

GRIGLKRHLRSEEIVAQGFIARHVLRLPIRNIVFMGMGEPMDNFDAVSQAIKVFSDQGGM

AFGMRHLTVSTVGRVDGIRRFVSEVNPAVNLAVSINAPNDALRKTLMPLTRKYSLGTIKE

ALLDYCSHPRRSVLIGYVLIKGVNDSLELADELAAYIKGLRAKVNLIPYNPQESDPYETP

ESESVNAFAARLRAQGVSTLLRQTKGDEMMAACGQLGGVNKSVHSSQKSPFRPSFQELS

>tr|D6YUF6|D6YUF6_WADCW Ankyrin family protein OS=Waddlia chondrophila (strain ATCC VR-1470 / WSU 86-1044) OX=716544 GN=wcw_0395 PE=4 SV=1

MPNYKFILEIVMFKTSDFDENINKTQKEINELEIRNGQIDRDYSDLLSKLQITSEQLSRF

IEKKENFTEKNWEQLQERKKEIEQKLATDLTNIRDPLKSKKALQDRNVGSHWLFIR

>tr|D6YTE0|D6YTE0_WADCW MinD/ParA chromosome partioning protein OS=Waddlia chondrophila (strain ATCC VR-1470 / WSU 86-1044) OX=716544 GN=minD PE=3 SV=1

MATRKKIAIAISSFKGGTAKTSTALHIGSALSQFHKQKTLLIDFDAQANLTTGLGFDPDE

HDSLAPVLQGNKEVKEVILPTNVKNLDIIPADTWLERVEVTGSLAADRYSHERLHDIIEP

LDYDVVIIDTPPSLCWLTESAMIAAKHTLICSTPEFYSVKGLERLSQFIESIGQRHPLNV

LGVVLSFWNQRGKSNTTFLDVIEKTFPQKVLKTKVRRDIAVSEASIFGKPLFETAPKSRA

AQDYKTLSKEILDRL

>tr|D6YUK9|D6YUK9_WADCW Uncharacterized protein OS=Waddlia chondrophila (strain ATCC VR-1470 / WSU 86-1044) OX=716544 GN=wcw_0448 PE=4 SV=1

MRVKIGNAQGFWGDQAGAAARLLALQPDLDYLTMDYLAEVSLSLLAIQKERIPSLGYARD

FIEELRGLIPHWEKGSHVKVVVNAGGLNPIACARSCAEMLKNSKKIAVVIGDDVLEIIAR

GGEYLNLDTGEEVESVRPLLVSANAYVGAAAIAEALNRGADIVITGRVADPSLVVACCIS

HFNWKLDAYDQLAQATVAGHLIECGTQVTGGISTHWLDIENRENLGFPIVEVEANGTFVV

TKPEGTGGVVNEQTVKEQLLYEIGDPDRYLSPDVEVSFLELSLEERGENRIQVSGAIGRS

PPSTYKVSATFRDGFAAEGMVALFGTYLREKASLCGQMVFERVKQAGFVLESTHVECIGL

GDVVKGVMQPIDDRNLREGMLRIGAKDSRREAIDCFAKAIAPLVTSGPQGVTGYFHARPK

TRQVFGFWPCLIPVDLVHLSVEWVEA

>tr|D6YS02|D6YS02_WADCW 30S ribosomal protein S19 OS=Waddlia chondrophila (strain ATCC VR-1470 / WSU 86-1044) OX=716544 GN=rpsS PE=3 SV=1

MAKRSLKKGFFVDHHLLKKVDDQNEAGTKKPIKTWSRRSMIIPDMVGHTFEVHNGHKFLT

VFVSENMVGHRLGEFSPTRVFKGHPVKK

>tr|D6YW98|D6YW98_WADCW Uncharacterized protein OS=Waddlia chondrophila (strain ATCC VR-1470 / WSU 86-1044) OX=716544 GN=wcw_1050 PE=4 SV=1

MRNRDIPKLGIGLGLRIPHYTDILTKRPPVDWFEIISENFMVEGGKALVNLEKFLETYTI

VQHGVSLAIGSPTPLDFDYLKKLKALTKMTRTPWVSDHLCWGQTPGGHYHDLLPLPYTKE

VADYVAERARIVQDYLELPFALENLSSYVSYQIDEMPEWEFYTRIAEKSDTFFMLDVNNI

YVSSRNHGFDPREYFNNIPLDRVLQIHIAGHNDKGEYVLDTHDHPVRDEVWKIYADVWPK

TGGVSTLLEWDNKIPPFQKTWEEALKAKQFQRNLEMEFA

>tr|D6YU23|D6YU23_WADCW Uncharacterized protein OS=Waddlia chondrophila (strain ATCC VR-1470 / WSU 86-1044) OX=716544 GN=wcw_0259 PE=4 SV=1

MSSIRGFVQRLIGGYSSNDQKIQHPNKSKKAAPIGGEKNRQGISRKGGDANISR

>tr|D6YUB0|D6YUB0_WADCW Uncharacterized protein OS=Waddlia chondrophila (strain ATCC VR-1470 / WSU 86-1044) OX=716544 GN=wcw_0347 PE=4 SV=1

MDLQTIAHYTFPASARDIGTGVYHLAKGNATKEQCQKLVAGAIRVTIAALAAISLVAASV

LLPSPLNLLGSLIIVIPLRIVDAETATLLSGTTGIVSGLRECALGISAIAQGIHSGPPKL

LLGTLETGFYWLITRMPRDIQIYRGRFAENIDDFAKSWGSQLHLKLA

>tr|D6YS61|D6YS61_WADCW Uncharacterized protein OS=Waddlia chondrophila (strain ATCC VR-1470 / WSU 86-1044) OX=716544 GN=wcw_1558 PE=4 SV=1

MMFSACLFLGFPVDPLFAEKLKKVNPALLNAFIQEGGEDLCEINYQQMRYIGKRLGSIIS

LDNLELLEKNIYSFLKRLVPDFPYEEAPLYLVSLLDDHS

>tr|D6YWQ1|D6YWQ1_WADCW Proton/sodium-glutamate symport protein OS=Waddlia chondrophila (strain ATCC VR-1470 / WSU 86-1044) OX=716544 GN=gltP2 PE=4 SV=1

MFFKKLSRQSQSLAAAILLGLFAGYANHPTMDAAASVVSEIFINLLKLVSLPIIFLSIVS

TASGMDSIDEIKSIGKKVIKYTLLTTVIAATFALIIFVTIDPVRSVVSKAPPVDLESGSL

PGYMHYLINIIPSNIIQPFSDNSVIGVLFLAMLLSFAILSLPQEKRKLLNSFFSGLYAAI

MKITTWIVQLMPFAIWAFITLFVKDVRSGMEMKSIALYLACVLIANLVQAFVVLPILLKI

KKMSPVKIAWGMMPALSLAFFSKSSSAALPMAMKCAEERVSVSRKVASFSLPLCTTINMN

ACAAFILTTVLFVSMSNGITYTPAELVFWIFIATIAAVGNAGVPMGCYFISSAFLAAMNV

PLNILGVILPFYAMIDMLETAINVWSDSCVTTIVQKEMDQEESAAAIVSAV

>tr|D6YS22|D6YS22_WADCW Uncharacterized protein OS=Waddlia chondrophila (strain ATCC VR-1470 / WSU 86-1044) OX=716544 GN=wcw_1518 PE=4 SV=1

MRPELEAIKSQLLSGDLSIQEIEEHRDKRDAFIPVFTTCRVFTLYKDNSVIGYYKEEKYG

LFGGAIMSSLAYEMSEILGVSSRLIPSMKMEPFSIEGKQYQGGAIQQAQEGLTFKNYLNH

PEKRKSTILKDEYVEALIDSVIIGMFDAHANNILIDSNGNIHFFDLTRSFPHSNAFLDRG

GYLSSPYRGGLLFGKESSSILNLTSKKAFLERLGAMKSTLPQVKQYLLSESVRKQARELP

ENWFNAEKLFQSLEERIEKLIGGFKNHAIKQARDIVFQSFPYYRLICLLNYCMLLDWRAH

AKNAYLLNRALHDIELPLFYELLPIPIEGMIKECVKLGLDPLAIKKHAEKESLMQAMSGI

VHDVQEKLIHPSSTKEMRFLTQREELLKSELKSMAEIDYKDWGENYPEEDFFTQPNS

>tr|D6YUE1|D6YUE1_WADCW Uncharacterized protein OS=Waddlia chondrophila (strain ATCC VR-1470 / WSU 86-1044) OX=716544 GN=wcw_0380 PE=4 SV=1

MTIGSIQSGISEAAAKVFTPIKNAAAWAGHQITKGFDKFSELIKTAWNTVFPFLKNLAIR

TTDFLKTAPGMGLVLGASSLALGYAAHTNHEKKWVSASLQAASIVTAIGTGIILGYGYAI

GFHAPLI

>tr|D6YSG3|D6YSG3_WADCW Uncharacterized protein OS=Waddlia chondrophila (strain ATCC VR-1470 / WSU 86-1044) OX=716544 GN=wcw_1664 PE=4 SV=1

MVSYLFKAGLILLMSVAALGVAAVYDAGDSDLEKGKEHKWDDKAGWNKTDEPLAVEIDIE

KDSLDLTDKEDLESGVLEIAILGSEDFDVNHIDPSSLSIGNVDGAEKFMVFDQNNDGIED

LVAGFSISDLNLKEGVQELTLKGKTKDGKMFKGRDQITVTKAY

>tr|D6YTM2|D6YTM2_WADCW Phosphatidylserine decarboxylase proenzyme OS=Waddlia chondrophila (strain ATCC VR-1470 / WSU 86-1044) OX=716544 GN=psdD PE=3 SV=1

MKHAIRYIDRDTGKLEQEKVFGEKAIHFLYNHAVGSKLNRWIARSSLVSKAFGWWQKRLW

TKSRVIPFIESYKIDASEFLHSPETFSSFNDFFIRKLKPEARPIDQRKETGVMPADARYL

FFSNIAQTDGYVIKGRKFNLQALLQNGELAERYTRGSMVIARLCPTDYHRFHFPVDCIPT

PPSLINGHLYSVNPLALRKNIRIFTENKRMITHLDSQTFGKVLFIEVGATNVGAIHQTFT

PGRSYQKGDEKGYFSFGGSSIIILFEPETIIFDEDLVKASQQRIEIRCLMGQSMGIASNG

SSLC

>tr|D6YWK1|D6YWK1_WADCW Triosephosphate isomerase OS=Waddlia chondrophila (strain ATCC VR-1470 / WSU 86-1044) OX=716544 GN=tpiA PE=3 SV=1

MPREVIIAGNWKMYKDRREGKDFLSKFASAVSDAPHTVYLAPPFPLIAFLSRQAEGTNLL

IGAQNMHDAEEGAYTGEVSAKMLLDAGAEFVILGHSERRRCFGEDNAFINRKVKRALESG

LRPIVCIGETEEERSCGKTEEVLHEQLSDSLAGLSSEQVSRSVIAYEPVWAIGTGLTATP

GQAQDAHAFCRSVVKMLWGEEVSEGISILYGGSVKPDSARVMMEQSDVDGVLVGGASLDP

ETFAQIVNYQTMKG

>tr|D6YWB5|D6YWB5_WADCW Uncharacterized protein OS=Waddlia chondrophila (strain ATCC VR-1470 / WSU 86-1044) OX=716544 GN=wcw_1067 PE=4 SV=1

MKPIIRSVNKIWENAEHNALTDLIIFQKKFYCVCRESDEHVFGRNGIIRIIQSIDGKEWR

AHSVIRKEGVDLRDPKLSITPGHQLMLLVEEVVYEGEKALSRFSSISFLKKDWTPLQKIC

RPFDWLWRITWHKGTAWGVSYKPDKDKWRVWLWRSETGTEWRQALEWKIPGNPNETTLRF

MDDETMVALVRRNFKTNGYAWIGTSKPPYTEWRWNETQHHLGGPDFIILPDQQMWAAGRI

VERTPYGMFQKTALFTMSLDKIQPALLLPSGGIDCSYPGIVFHDQLLWISYYSSHEEKTA

IYLAQIELFE

>tr|D6YVP4|D6YVP4_WADCW Uncharacterized protein OS=Waddlia chondrophila (strain ATCC VR-1470 / WSU 86-1044) OX=716544 GN=wcw_0839 PE=4 SV=1

MKKWLVFFCVCVLSLLSASEKSDYFAKLTPQEAKDIQYIVTTLGNTSAIGLLFKKKSLEQ

AGARIDDVHPLRFFGYVMTNPQLKASFDKIKGVAWSRFKEGMAGSLEKADSRDHLNAEVI

DDFSSESHLDRSKVQAYVDRKQWEALIDFMRR

>tr|D6YUU7|D6YUU7_WADCW Uncharacterized protein OS=Waddlia chondrophila (strain ATCC VR-1470 / WSU 86-1044) OX=716544 GN=wcw_0537 PE=4 SV=1

MSKYMNLSAYSNLFSDRFLQSFSKLYLRSDRKNLSKRKKICTNSNIRIGS

>tr|D6YV86|D6YV86_WADCW Uncharacterized protein OS=Waddlia chondrophila (strain ATCC VR-1470 / WSU 86-1044) OX=716544 GN=wcw_0680 PE=4 SV=1

MYKTILSFIFLIFAGMSSLQAEPLPLSLRLKEGQKIKQTVSLDFSCYLEASEQFSDQIKK

MHETQPTEETVLYMHALNQLTVDKPVFTGNVKTTVQSTILKDHGQYYTISTSSVGNKFSI

QENLINQFSLAYDADNPKNNFSNLPFPIDFKQIDEAMKQTEAITARVTKDGTVIAFDDLE

QKISTQAKKNGLPSNQLAQISETIGSISQLTPQFKLPSYLTGIRLPNTLMKIGESLNQPY

ITIPDEIKASIPQEALPLLALLEQKITLIEKNGEYAKFSIEMPLTQPPAIPVSDNDQITF

KGTLFKGNMTINVNTGLLVKSNLATDFSIIFSSKEESQGTMSDEITLRFVSRGQTETESG

S

>tr|D6YWH7|D6YWH7_WADCW Ras-GEF domain-containing protein OS=Waddlia chondrophila (strain ATCC VR-1470 / WSU 86-1044) OX=716544 GN=wcw_1131 PE=4 SV=1

MTPLTSTIKDIQVPEASPSNIQTFATQAVACFEAIQNACQGIVKNAERQQKYFKLEELRK

QIKSLDTSQVKELSQLNQKIRREMRALRNQYQTNSHWLVLTQKQSHLVEIEPSISLVPRV

KIHPDDLQASFKEIAALRRGVEKVRALQHLYQKAASLEESDSSELLIEIQRFSDSNLSLS

EKILALKQIKWRDKDDKQLDELKALIDPTGKDFVQYFLRGDLKPKDAVNYITVVLKDRLM

YNPSVSPLELLKSWPTSIEKKDSDQWIAALMKMESKKSIQDSFTNFTISYLLNCIDPSGL

QRCAELNKKRLSSASFAKEVSEVIDTLETFGLDSKEIYQRYRFTTKENLPLQEMEKKMMS

ALLSESEIKQFAKALKSAVARDWLTFDMKTFMSGSKSFEQLIGNRLKIYDDLAEKYKNAL

KALSDEKEQAQMISNMLLLCYHLMRSGEFGLSFTLFSSGVDMTKNSCQKGWDIALKKHQK

HHDFLEKVCDPVKNLKNLRKLISNRESEKIACVPFFAIVLKDIIQFKEQISQPRDNLIEQ

LFGDHGHLAKKIVDLLAKGKIEEEEKDWAKSHFFKDIQKLFRIIEMTDAPYAEKNLVRKK

IVDILQQERELQLEILKRKRALNQGISLLSLYYSNQTEEESLSSLL

>tr|D6YSY7|D6YSY7_WADCW Putative membrane protein OS=Waddlia chondrophila (strain ATCC VR-1470 / WSU 86-1044) OX=716544 GN=ompA10 PE=4 SV=1

MMKILKNLVFGILLTSAHFTHAFDGYDHCDPCYDQNTCDFLCVDFCNMEFSVYADALFWH

LHSTDLVLGDGSFDKLTPGNDWGWRVGGSARWNVWDLSLRYTSFRSKADKEAHFDQVPIV

AKYAFDYRVFDAELGASCCICDGLLFRPFLGVKFANIDVTDDHVSELERDFTQGLDGSGL

YIGASSHWKLCSFNACGCCIPVAFVCRMSTGILDSDFTRTEPPAPTEKERRESKAFRFIP

VHEVYAGLEFRMCVLCDADAFFQIGYEAQYWGWKGIAEDALSHAYLGVGGLVLRLGANF

>tr|D6YSA0|D6YSA0_WADCW Uncharacterized protein OS=Waddlia chondrophila (strain ATCC VR-1470 / WSU 86-1044) OX=716544 GN=wcw_1600 PE=4 SV=1

MKICFKNFPYRVLVLDTQNLNPEKKRKYSLFTTPKFRCCLEGKDPYDIGFTEQIAIGIEK

NSEPIGLALASLFKGLDYAEIHTINIDIHYKKPNILKNFISEIEKKLFSQNANYITYRFS

PDTPIVNSLVPVLEENSWASPKLVMIRYFYDAQAFNPPWYDRPPLLPSGYKTFKWETIRD

SELEKIKKDYKQGYFTDHVSPFWEAEKIEPVNSIGLRYKNQVIGWMICHRVAHDTIRYTA

FYVKHQFAFTGYPLRLLVDSMKLHQTSTIKWALFELNFQDPPTSWINFVLKRLAPLAQAQ

IPVYETWKQRETGS

>tr|D6YSR7|D6YSR7_WADCW ABC-type transporter, permease subunit OS=Waddlia chondrophila (strain ATCC VR-1470 / WSU 86-1044) OX=716544 GN=wcw_1772 PE=3 SV=1

MISFLEALYANPLLLSAVIAGLLASIVSGIIGSYVVVKRIVFISGGISHSVLGGIGLCLW

LERAKGVSWATPLLGALTAAIVSALIIGWIHINWRQREDSVIAALWSVGMAAGILFISQT

PGFNVELTNFLIGNILWVSTTDLIILGILDVIILAIVFCMHKRFLAICFDEDEARLQGLH

VNALYLLLLALTAVAIVLLIQVVGIILVMTMLTIPAAIANTFTSRLSRMMVISVILSSFF

SFFGTAFAYHVDWPGGATIALLAGVGYLLAMWLSPLLKRFSYSRTL

>tr|D6YRK3|D6YRK3_WADCW Uncharacterized protein OS=Waddlia chondrophila (strain ATCC VR-1470 / WSU 86-1044) OX=716544 GN=wcw_1347 PE=4 SV=1

MARSKKYQEFLLEHLADHDEAVAYLNAALEESLKGDEESQHLFLIALRNVAEAQGGVGAL

AKKAHVGRESLYKTLSGTGNPKWHTLVSLCVAMGLNLRLS

>tr|D6YWQ5|D6YWQ5_WADCW Pyridoxal phosphate homeostasis protein OS=Waddlia chondrophila (strain ATCC VR-1470 / WSU 86-1044) OX=716544 GN=wcw_1209 PE=3 SV=1

MSVIDNYHALISDIGNTVRKAGRDPSEVSLIAVTKGFSLEHVLPAYKAGARGFGENRVQE

ALPKIEKAPKDIEWHLIGTLQKNKVRKVIGKFSLIHSVDSLELAQKISETSLVCNTVTPV

LLQANTSGEQSKHGLQSEEWIANWDSLLELEGIDLQGLMTIAPFVDDEKVIRRCFRALRQ

LRDRLQAMAGNKCTLKHLSMGMSHDYRLAINEGATQLRIGSAIFH

>tr|D6YW23|D6YW23_WADCW Putative serine/threonine PP2C protein phosphatase OS=Waddlia chondrophila (strain ATCC VR-1470 / WSU 86-1044) OX=716544 GN=wcw_0974 PE=4 SV=1

MDVDPENALRSAKIFYDKKDYNKAMEFFVKAAFSLIYNSKEHACTKTAMEKAIDCYLKLN

PAYTIAPHDNYIQQYFTVVRHWQDHRKWSEEQICQELFKEIPCSLADDSREELPMGATFS

RQLNSSSFDAAIAAGQGIREEMEDAFVTEYFSLETASIDLFAVFDGHGGKTCAQYTAEEL

PKQLKNRFNQLAELNDEHIYQTLIETCVSIDESWKQLSFQVEGWKDISGTTAAIALYINQ

KELWVANVGDSGAVINLNGKAIQLTEAAKPTHPRYYQEIYMRGGIVSYGRVDASLDMARS

IGDLPHPSVSARPTVKKIEIDSKDHTLIIACDGLWDVIEGQTAVDSIKGKNSLEAAEHLR

TLAYQRGSTDNVSIIVVINQ

>tr|D6YUX7|D6YUX7_WADCW Uncharacterized protein OS=Waddlia chondrophila (strain ATCC VR-1470 / WSU 86-1044) OX=716544 GN=wcw_0568 PE=4 SV=1

MSITPILHAAEGRREVRKLYKNEERKAHLKEAIKTVYSTPLAVFLIQAQKPDGLLYDYNI

HSWRKREDSDTDEKLDRHKASIDFLVDRVEKLAKKFVEGTEYSEENVRSWLQYKLGLGQF

KNTSDFIEKGQATFTQNHIKLSRALTKIYENRFLGKKEKAQKKNIDEIISNSADVVEDSL

PLDSHIASNSEKLSVEATDQTQKKQSAIVRFFIRIGNGIKQSFISIWNFLRRIFRVV

>tr|D6YV87|D6YV87_WADCW Putative permease, YjgP/YjgQ family OS=Waddlia chondrophila (strain ATCC VR-1470 / WSU 86-1044) OX=716544 GN=wcw_0681 PE=4 SV=1

MLTKIWERYFIKETLKTFFFISFCFYGLYVLIDYASHSGARHHQSMMEWSELIKFYANEW

ILRADVLVPFALMIATIRTLCDLNIHNELVALQASGISLKKILRPFIFLGLLFTLLIYAN

TEFLLPNAMAASRHLRNQKEIQKNNKNKKFFVQHVGLKDGSTLLYQHYNFAERSFFDAYW

VKSFDEIWRFQHLSPYEQPPKGIGVGHFQRMPDNTLHKIESFESRSFPEMKFNQKILLDT

LTPSRELSLSTLWKRLPHSKEIESEKEAEFAASFFHKSALPWLCFFAVIAPAPFCVRYGR

SHPIFFIYSLSLFFLVGSYLILNAALIISERQVLSPEVAIGVPFMLFSSTTLYRFLYRS

>tr|D6YTG3|D6YTG3_WADCW Transposase remnant OS=Waddlia chondrophila (strain ATCC VR-1470 / WSU 86-1044) OX=716544 GN=wcw_0048 PE=4 SV=1

MGNKKGGEAAGVVLSLVQTCRAMGINPREYIEDVMRRLMSHNAQKLQELLPDFWLKAKEC

PAA

>tr|D6YWJ3|D6YWJ3_WADCW 2-amino-3-ketobutyrate coenzyme A ligase OS=Waddlia chondrophila (strain ATCC VR-1470 / WSU 86-1044) OX=716544 GN=kbl PE=3 SV=1

MNQGFLDQISNQIEELKGSGLYKEEYEMTSPQTSAITVTGEKKVLNFCANNYLGLANHPE

VVEAAREGFAKWGFGLASVRFICGTQTIHKELEASISNFLKTEDTILYSSCFDANGGLFE

TLLGPEDAVISDSLNHASIIDGVRLCKAKRFRYKNNDMADLENKLKEAEAQGVRYKLIAT

DGVFSMDGIIANLKAICDLADQYEALVMVDDCHAAGFIGKNGRGTHEHCDVIDRVDIITG

TLGKALGGASGGYTSGKKEIIEWLRQRSRPYLFSNSLCPGIVTASLKVFDILSSSSHLRE

KLKKNSIYFREKMEALGFTLVPGEHPIIPVMLGDAKLAQEFAARMLDHGIFVIGFCYPVV

PKEQARIRTQMSAAHSMEDLDKAIKAFEIVGKELNVI

>tr|D6YSV4|D6YSV4_WADCW Uncharacterized protein OS=Waddlia chondrophila (strain ATCC VR-1470 / WSU 86-1044) OX=716544 GN=wcw_1810 PE=4 SV=1

MCLKKFISTSIFSLSLITTAADASTNKVSQYNAEIERLEQLENELHKLIKEAKNVLSEHS

TSKQTESIQEIAAAEPQAETVFAKEESSVAKAESEPQHSFRFQGKETPWVTSFYENNNNL

FVPQENIEESCASTPCYQPKRDCCRDSILLYADARLGNRPCFDGSIFTGGLMYFENSFFR

NGITLFLDVNGSSFEHGGSGVSAGIGARFCLPCTNTLLGINTYFDYNDTRTCVDGDRFEC

IRESSGIEVDKVRRSHSFRQASVGLEWIKCAYSIRGNAYFNAGDDRAHCRCKIYDVYEGP

YEVECKSWTQALQGYDIEYRQHICLPCNLWAYPAIGGYYLTGGCCSKLGVMGGLAVSWRN

ILSVEGIFYYDKYNESKGEAYLSINLPLDFFCNPCCRSYCPDYSKSRVRRRNYIPTEEVC

RYTKNY

>tr|D6YU28|D6YU28_WADCW Glycine cleavage system H protein OS=Waddlia chondrophila (strain ATCC VR-1470 / WSU 86-1044) OX=716544 GN=gcvH PE=3 SV=1

MKYTESHEWIDLEGSIGTVGVTDYAQKELGDIVYVELPEVGKEVNAGDEIAVLESTKAAA

DIYAPVSGTIIEVNEVLNDQPEEINISPMDDGWLFKIELKDKDELDQLMSQSEYLETVQ

>tr|D6YRZ8|D6YRZ8_WADCW 50S ribosomal protein L29 OS=Waddlia chondrophila (strain ATCC VR-1470 / WSU 86-1044) OX=716544 GN=rpmC PE=3 SV=1

MIMKPQEMRDQSIEELVAKLEESKRELFELKNEMKRSKKLEKPHLLREKKKDIAKFNTII

REKQLANR

>tr|D6YSG2|D6YSG2_WADCW Uncharacterized protein OS=Waddlia chondrophila (strain ATCC VR-1470 / WSU 86-1044) OX=716544 GN=wcw_1663 PE=4 SV=1

MKNFEDILQNLSANQKKMIREASEEGAQILFNIKGMIEILRDNKPFEFIEMGYGDIEKLR

EKGLISAVRTKEEGHVLQGELYELSDTGKKVAELLLNP

>tr|D6YUU5|D6YUU5_WADCW Glycerate kinase OS=Waddlia chondrophila (strain ATCC VR-1470 / WSU 86-1044) OX=716544 GN=glxK PE=3 SV=1

MKVMVAPQAFKGTLTVFEVGEAMMRGVQKALPGCECVFVPIADGGDGFMDVLFYYRQGTL

IQQQVTGPLGERILVEWGIDEASQTAVIELSKFSGLSLIPEHRRNPLAATTFGIGEAILL

ALDKGIRHFLIGIGGSGTNDCGAGVIEALGGRFLDEWGNPLPRGGAALKELKTIDLKGLD

DRLLESTFIVGYDVKNPMTGPEGASLVYSSQKGADPVEAERLEQAVLRFVEVIQSQLGIE

LNAIEGSGAAGGAGGGLHALLGARLISGIDLVLDRAGFDRLLKDADLVVTGEGRIDRQTT

YGKGPIVVAHRAKARGIPVYAFVGSVGEGYEAVYDHGITKVIPL

>tr|D6YV06|D6YV06_WADCW Putative membrane protein OS=Waddlia chondrophila (strain ATCC VR-1470 / WSU 86-1044) OX=716544 GN=wcw_0599 PE=4 SV=1

MKLWKKTAILLTCSSMLCLNTSEGIEYCTDTGGCAYEDAYQSCCIAPAIAFALVAIAGII

AVGVHNRSSSSHTTNAHTQ

>tr|D6YTV1|D6YTV1_WADCW Uncharacterized protein OS=Waddlia chondrophila (strain ATCC VR-1470 / WSU 86-1044) OX=716544 GN=wcw_0187 PE=4 SV=1

MEIKKFIELDKRGLIPGPGESEEAFAKRAAYCLSLKDFLKSTLEGKIPFDSKEAPLDLSE

QDMERVDKQYGIRPDWVPVFFSNTRLTPWHGGCAWIFQMDESSPTAAFLQLRKSFFFSDQ

YLKVLRKEEVVAHELAHVGRMCFEEPKFEEFLAYQSSSAVWRRYLGPLVQSTWESMLFVF

LLFVIFLFDLFLFWHGDYGSYQNWMWLKTIPLVLAFIGVVRLVVRHWQFKKCSNTLSRLI

SNPCTVKQVLYRLTDQEIISFGSMDKQSLISYIKEMEASALRWRVLVKRYFSDIY

>tr|D6YSM8|D6YSM8_WADCW Tetraacyldisaccharide 4'-kinase OS=Waddlia chondrophila (strain ATCC VR-1470 / WSU 86-1044) OX=716544 GN=lpxK PE=3 SV=1

MINELHFYYIQVIRGKRTGWLPTLIKGLAWVLSLPYRWVMSLRNWLYDHEWLRQYDAPVP

VVMSIGNLVTGGTGKTPVTKLLAGFFYDDYKIAILSRGYRSPAEKLRAPVILSSGKGPLH

SAAYAGDEPRLLAENLPKAWVVVGKDRVMSANLVAKQGVDLILLDDGMQHRRMARDFEVV

VLDAKDPFGQNYLFPRGLLRESPEGLRRADLVILNHVRDAEDYEDSKKMVEKYTNAPVIG

IHYDRWKAMDLEGNELAPLEGRKVAIFCGIAQPEQFASTVREMGAEIVARKYYPDHFHYD

VEELSELAARWKEMGAAMMVCTEKDKVKLPEIHDLLLPVVWIKIQPEVIEGTDELKAFID

KVKAKIA

>tr|D6YT99|D6YT99_WADCW Uncharacterized protein OS=Waddlia chondrophila (strain ATCC VR-1470 / WSU 86-1044) OX=716544 GN=wcw_1961 PE=4 SV=1

MLDEEGFGIMSVKLKRNSSLDWPYPFVPDERVPRAQYETLKMLGKE

>tr|D6YRU5|D6YRU5_WADCW Uncharacterized protein OS=Waddlia chondrophila (strain ATCC VR-1470 / WSU 86-1044) OX=716544 GN=wcw_1441 PE=4 SV=1

MTIKNLFNQLTSYLCPPTEHVYSSLYDSLCDVQTALDQQRNFQEVRQDGIEQLAEKLNGI

VDEVIKVESSSSFDQDDLHKLYEFSGSLLTIDPVNAALWERLAKPVQEFICSLHPLFQPP

TDKIDLAMRIPRTSRLGKGETKTLMFGDRHIFRATFLKTSFSRLSFSIAKQKDLKLKATA

SLDSRIQEKVQEYTAHLPYGINIFFHIDKLLKTIRQFGNQPNLKVQIVSQLTHQLFIAKQ

TYLNMMLHAVGMPAKYPLEYALQGEWIIEPRETSESPQKIADQIAQFLILEKTLGIYLLG

DSESVLDPEQTALFEVKENLLYCSIPRKRIEFLRMPFEQRLKKVNDFLAQSHPDFARCIP

QIEEAVGKVPSPNKIIEMIREGFASGRLERDHPLNTAIVSVCLQLPEAFFSPPIEWDQFQ

SPKDFFLYCKLQLETSGKSLEIPEQQTFLQEHLILISAWPEDNPEEKQLRLEKLLFENLA

QFQKRRIASIIKNALKN

>tr|D6YW22|D6YW22_WADCW Aspartate-semialdehyde dehydrogenase OS=Waddlia chondrophila (strain ATCC VR-1470 / WSU 86-1044) OX=716544 GN=asd PE=3 SV=1

MKRKIPVAILGATGSVGQSFVRLLKDHPWFYIAELVASKRSAGKKFGDFVPNTHLEDHFI

KAVSDSLSSKIVFSALDAQVAGEIESSLARKGHWVISNCRNHRYDPDVPLLIPEVNPEQL

HWIKRQQFGGGVIVTNPNCSVIGLALTLKPLVEAFGVEQVHVVTLQAVSGAGFRARTILD

IDDNVIPYISGEEEKIEKELDKILCIKRVSAQCNRVSVSDGHTQCVSVKLSQTASLEEIK

SAWTAFRSPVLPSSPSKPIHYFNEESYPQPKLHRMLEKGMAVSVGRLRKCSLFDIKYATL

SHNTIRGAAGCAIMNAELLIDHHYNGDIVGASSLIGQCP

>tr|D6YT15|D6YT15_WADCW DUF58 domain-containing protein OS=Waddlia chondrophila (strain ATCC VR-1470 / WSU 86-1044) OX=716544 GN=wcw_1872 PE=4 SV=1

MSETSLEVVKKIRHIQMTTTHLANDIMAGAWHSAFKGQGMEFEEVREYQVGDDTRSIDWN

VSARMDHPYVKVFGEERELTVFLVVDVSASSRFGGKHQLKQDLIAEVGAVLAFSAIKNND

KIGLVLFSSEIEKYIPPKKGTRHVLRCIRELLAFKPKHKGTDLEKALEFLGTAQSKKAIC

FLLSDFLCSIPEDSLSVIAKHHDLIAISFRDPYEMELPNLSLVTLEDLETGKQATIDTGN

AKMVQQFNQHSQMDLERLDKIMKKIHAGTIHLKTDSSYLKPMRKFFKQRGVRH

>tr|D6YRS3|D6YRS3_WADCW Transposase OS=Waddlia chondrophila (strain ATCC VR-1470 / WSU 86-1044) OX=716544 GN=wcw_1418 PE=3 SV=1

MPDDLNATVVKDYKDFEVAIIELDEQWSYVGNKKNQQWLWLAFHSASRQVLAMHVGKRDK

RAAEALLAKLPEDLKKKPSFTLIGSLCTTKSFLGSNTRQSEKIQEKRVTLKDLTTP

>tr|D6YUQ1|D6YUQ1_WADCW Metalloprotease OS=Waddlia chondrophila (strain ATCC VR-1470 / WSU 86-1044) OX=716544 GN=wcw_0491 PE=4 SV=1

MALNKIGDTYNAFKLKRLVPLPEINCLLREIEHEPSGAKVLHIENNDPENVFCLSFRTIP

ETSNGVAHILEHTVLCGSKKFPVKDPFFSMTRRSLNTFMNAFTGDDFTCYPAASQVPQDF

YNLLEVYLDAVFHPNLKEYSFLQEGHRLEFSIPEDPSSPLEYKGIVYNEMKGAMASPDQR

LYNAIDHALFPDLTYSVNSGGDPMVIPELTYEELKAFHSKFYHPSRCLFYFYGNLPVERH

LDFIQKNILSGVEKVEPIPHLSRQKRFQEEKSKEIGYPFSRDEDPSEQAMAAFGWLTCGI

LEQETTLALEIITLVLFGTDAAPLKKALLKSGLCKQVFASVGDDNSEIPVSIVVKGCNPD

NIQKLKILIKEKLQEIAEQGLSDTLIQSALHQVEFHRSEITGDHYPYGLSLFLRCGLLMQ

HGGNPESSLLVHSLCDDLLAKLKKNPRYLSELITKHFIHNPHFVSILAAPDPELNNKERE

LEEKKLKEKEIALDDSQKEFLVKRAAELSAFQKEQEEINIDILPKISLKDVPKNSMNFPL

EQEAFNNFTLYHHDCFTNEIIYSDLVFPLPRIEEQELPFLRLFTLLLPQLGCGGRSYIEN

LEYIQAHTGGISVSETLNAQISDPSLLDPYLTIEGKALKRNQGKLFKLLREMTTSTDFTD

ASRIKEVLVKHFTGMQSKFTQNALRYAMGLSTSPFSPANRINQVMGGLTYYHFLKELMEN

LDSRLESLITSLQSLQNRLLGAGSPHLVLSCSASDYEALKKEHCYGLCEIETKEAAHWIT

NHQIPKVESQGRIIASPVAFTSTATRSIPYKHPDTPAITAAAKMFDNLILHPQIREKGGA

YGAGAINHATQGTFTFYTYRDPCIASSLDAFALSVQKIAEGSFSEEDLEEAKLEIIQGLD

SPISPGSRAYTAYSWLRSGKSLEIRQQYRNQVLSLTREQVKEAVKTHLAPQMQESVTVVF

AEKELLESENRKFQSLNKPLLPIFST

>tr|D6YVZ3|D6YVZ3_WADCW Putative rhs family protein OS=Waddlia chondrophila (strain ATCC VR-1470 / WSU 86-1044) OX=716544 GN=rhs25 PE=4 SV=1

MIRSLSLLSLFLFSQLQAFEKYDHLDGLGDPSIYVEGYVNALTGALVHSQADLVVDCPEE

LSFIRNYNCFNCYNNSFGSGFTHNHPTGSFRDEYDKKTDTRTIYVGEKSGNCLKYSDWKF

SKHSRNYYLDEEHREHDLTNTYSGKISGRHNLKNSYLTADGRQDRIRIREMKLGDGTVRK

YNDKNQLSTEIRPSGNQVKYYYSKDQLCSIQAFDRNGKACLGWINFYYNSGMLSSIKTSD

GKIAKYTCEMKRIKKHCKEKLPLLKKVQLFDQPKEILYSYDENVIKIKPEDGSDSFRIYE

RKISGHSGGMHRSKCEYGSSGKISKLTLFGPQRRGAVYYFCYPFYDRGTIVITPLKAALR

YTGSSVRVGSITTYLNGTFFANPKDLYRKQRFYWGEKEKTIDNRGNLTIKALEDSNEHIL

CAASYIYDHRGNVLKETLYGNLTGKCDFEPIKITKKNEVVGQESFHHTYTYTDDGRNLVL

THEDGDGVREEYEYVPGSNRVSQILTIVNDQVVKRQCYTYNSFMALTSVREDNGSSRDVN

DLSDVTSSTVQMHTLIEDLKTSGFGKPEETIEGYLNPLTGVLVQLKRQTYTYGKYNLVTS

QTVYDADDRLCFTLTFDYDSYGRCIRETDCFGVETVYKYNTHNCCVFSKKEGVGYSTSFA

YDSADRMIEKRELHESGEEFVYTFAYDVMGNLIEQTDAYGNVKKMTYDSLGRHIKTEYPP

VFLADGTTSAPTEEMVYDIFDRIVEEKDPLGNVTQKRYTARGQLAEIVYPDGTSERYVYN

LNGSLHQKYEKNGSYHTMVYDEFQQLVRDETYSSDGNFLRKKEFVYDQGLLIYEIDASGT

VTEYRYDFAGRKSAKLIHGEEMTAMETYAYDSLGRLCGTKKWLSEEKGEYLNYIQEFDCL

NRVTLNKIEDHHGKTISWTSYCYDINGNCVEERCQQTEKAVSVAKAEYASYSMPLVTIDP

MGNATCYHYDFSFENVHGQKVFRSTVINPKGVKMITTKDVLGRDVETHMENAEGVLLSKG

QSFYDLKGNKTLSLTDSIVQGVVNRTYEVKWVYDAMDRVVRIIEDPKEKFKETQFTYHPM

GMLETKRKADGVLLSYFYDDLGRESEMFSSDQSVHYTYEYNLRDQLIRSVDSITGKTIER

RYDSFGNLIEEVLPSGMRMRYQYDPFCQILKITLPDESAITYTYDLAEMTTVSRFSPDQS

PLYTHHYTSADMQGRSLKSVLITGDEVDFKWDLNGRPTKISSLHFTEEISEKGYDSVGNL

LQADFINGGKQYSNIYNYDELNQLIYESTTRAHDYRYDSLHNRLSKDSDDYALNSLNQVE

QCEAHQYQFDLNGNTISQISAGNEISYTYDALNRLRAVEREGIWKIEYQYDSFGRRISAH

HFKWAGENLLEEEREYLFQGIYEIGYRLPSGEFPELRVMGKREGPAFRTCIAFELQGETY

APLYDHIFNVVALLDLSSGDLKAAYRYSAFGEMQELISSSIICPWLFSNQRYTRESNLYH

FGKRDYRPAIGRWISPDPGDFIDGMNLYAYTRNCPTMHLDFYGFSTQPVTKRPIAEPVYG

GFVSCEIMDYLVKGAGKCCEVAAHNILPESTGRFAIEGIARVMQGESFVPDNGFVLPSVS

GIVPGRPLDGIRVRYIPGIGTLESGARATAEKISGYLRGAEVHWTCQQSRGFVQDLGLCM

AEMMHIRTPAIQHLSEQIRNDYWEMRAIRGDNFLLFYIDHSRGGLDFYEATRNLPREMRD

KMFVLALGSAKLFDARDYKLVKNYSNDYDIVNFIANLTCSHHENVNLIYTEYCSGSGNPL

SCILDHLIESGGYQDTLWREIQFLLENGI

>tr|D6YWG0|D6YWG0_WADCW Putative membrane protein OS=Waddlia chondrophila (strain ATCC VR-1470 / WSU 86-1044) OX=716544 GN=wcw_1114 PE=4 SV=1

MKISLSSDPHIEKINNNRIYVDHDLRYRFHWSNYCGMIPLVSTVTGSIRALIGLIHTIVH

LAKSIFDKKRRKDHLLEAALGAYSVIRGCVEAVPLLGNLAIIEFDVIRILSKKTKSVYIG

PSSEYRA

>tr|D6YS97|D6YS97_WADCW PPIase OS=Waddlia chondrophila (strain ATCC VR-1470 / WSU 86-1044) OX=716544 GN=tig PE=3 SV=1

MAIQVTPLASEAAYQKAIKKINKEVSLPGFRKGKAPAKMVEDNYSKYVNQEWQDIMVQAA

FQDAIKLTSIYPLSRETIQKPELQDASRKNGGQIVIEFESSPEIPEIEPKKLKLKKVEKK

EIKDEDLGEALKDIQLHHATWEEIDNRGVEKGDYIDVDIEDASAPGTFICKDTRFEVEEG

KMGAWMLKLVIGKKTGDVVEGTSERSEDLDPDTEFTPTECKITIKSIKTPNLPEIDEELA

QKIGAESVEDLKTKLTSNLNSQAEEEVRHKLRMQVENSLAKHFPFEVPESLILSETNNRL

NHLRHKLKNAGETEEAISSKLNEDREKIRDDAIRAFRLFFISRHIAEENKISVSQDELVR

ELVVQMYSPSSPIDTSLDPEEVRSKVYVNLLSQKVKDFLIEQAAMED

>tr|D6YU81|D6YU81_WADCW Thiamine diphosphokinase OS=Waddlia chondrophila (strain ATCC VR-1470 / WSU 86-1044) OX=716544 GN=thiN PE=4 SV=1

MTPRFTAPVALVANGPIGSGEVLKNQLARFKTIIAVDGGLHTCHKLGVRPNFIIGDMDSA

GSELLASYPEIPKKTFSPEKDQTDLELAILEIKKQKINRATLFCALKMRTDHSLYNLHLL

SRYKEILTIETDYETLFFVEGNCSIPCTPGQTVSLLPLGIPAKGVVSKGLKWELENATLN

GTFASISNICLGDAFSLSIKEGELLCFLIK

>tr|D6YUM5|D6YUM5_WADCW 2-oxoisovalerate dehydrogenase subunit alpha OS=Waddlia chondrophila (strain ATCC VR-1470 / WSU 86-1044) OX=716544 GN=pdhA1 PE=3 SV=1

MCLEQFSFLSWQLAKLKEAQSKNIQLLFIVVRIVKLNFNLQEGKQKSMKIPTIAYLSAEG

KLSSSCKTKIPEKSLLKGYRIMCLTRHNDERMITLQRQGVISFAMSSKGEECCAVASAAA

LKASDWMYPQYREAGIVFWRGMSVEDYVNHMFCNAKDIILGRQMPNHFGSRELNIVTVSS

PIGTKIPHAAGSAYAMRVKKEKNVAICYFGEGASSEGDFHVGLTFAAVRKAPVIFFCRNN

GYAISTPTSKQFGSEGVAPEGIGHGITTYRVDGNDFFAVYETVLNARKECIQGKGPVLIE

AMTYRMGAHSTSDDPSRYRDDKECRKWEKRCPILRLKRYLEKKKLWNEEEEKKLQKKIKQ

EVDAAIAAAKEVLPPSLETLIQDVYFAPTPRLREQLQELKRFFPGRQ

>tr|D6YVM1|D6YVM1_WADCW Peptidyl-tRNA hydrolase OS=Waddlia chondrophila (strain ATCC VR-1470 / WSU 86-1044) OX=716544 GN=pth PE=3 SV=1

MTEKEQKAVVFVGLGNPGSKYAMTRHNMGFLLIESFARLHGVILKEEKKLQAKTGKCQIG

EWTVHLLMPLTYMNLSGQAVGRFINYFKLTTDNVVVVTDDVAIPFGELRLREKGSAGGHN

GLKSVEQHLGSHCYTRLRVGIGRGVDPSQELSDYVLDPFNKSEMEQLPSLLNQGVKALEL

LLKDSLQNAMNVVNSKLKPQEEIQNG

>tr|D6YT74|D6YT74_WADCW Elongation factor Ts OS=Waddlia chondrophila (strain ATCC VR-1470 / WSU 86-1044) OX=716544 GN=tsf PE=3 SV=1

MTITPQLIKELRDRTGVGIGKCKEALEEANGDLEIAIENLRKSGMASAVKKEGRAANEGM

IAIAESGSRVALVEINAETDFVVKNERFQQFLQEVAQEIVETNPVSLEDFLAQKFSKDQN

MTIDEYRATIVQAIGENIQVRRLKVFEKSSDSSLGVYSHLGGKIVTMVKIDGSGDAENLA

KEIAMHVAAAHPEYIRPEDVPSKVIDQEKEIARTQMAGKPDHVIEKILEGKISKYFDDVC

LNKQFFIKDDSLKIEDLVKKHGDNLAISHFERWTVGQG

>tr|D6YUZ0|D6YUZ0_WADCW Cysteine desulfuration protein SufE OS=Waddlia chondrophila (strain ATCC VR-1470 / WSU 86-1044) OX=716544 GN=sufE PE=3 SV=1

MFESCIKKQQNVKKLFESCRSKEEIYQKIIEIGRSSLGLEAEYKIPANEVQGCQSLMHMR

AFLKNGKLFFEAESEALISSGLAALLTHVYSGEEPIVILKCPPDYLEEIGVSSSLSPNRA

NGLYHIHLRMKQIALETFMEEGS

>tr|D6YSY3|D6YSY3_WADCW Uncharacterized protein OS=Waddlia chondrophila (strain ATCC VR-1470 / WSU 86-1044) OX=716544 GN=wcw_1839 PE=4 SV=1

MTRYLGLLLIIAAFCGCGTSQPPKMEKAIDATEVSRQILTYSEKLRFQKHLQLEDSVVYY

NNKINRIRLDFSSMDVLDIWEARALLVDLVDEFLERINGNAIIYRDLGTFPFTASDLEVY

IYFKSFYNHYVKLDSVGMISLKQGIASYIASDGFDCEGPCWRKRNEYYFQSRNFITFKRQ

GEALYKPDTESDFNIFGDERYIDPETRKYLNRISPRIKTNRPR

>tr|D6YX15|D6YX15_WADCW Uncharacterized protein OS=Waddlia chondrophila (strain ATCC VR-1470 / WSU 86-1044) OX=716544 GN=wcw_p0010 PE=4 SV=1

MNRTNKNRISKLESGITEVGRHKSALVIYDPNVDYEKDLAKIDADVVLALPDNGRKDLKN

GVVIGSCVVSYC

>tr|D6YTT6|D6YTT6_WADCW S1 motif domain-containing protein OS=Waddlia chondrophila (strain ATCC VR-1470 / WSU 86-1044) OX=716544 GN=wcw_0172 PE=4 SV=1

MNQTHIAKISSELNLSIGRVGAALKLLEEGNTIPFIARYRKEMTDSLDEVQVREIKERSE

YLKGLDDRRLAILESIESQGKLTEELRSKIEACETKSALEDLYLPYKPKRRTKATIAREK

GLEPLAALIIAQGVDGNPEEDAAAFINAEKGVESAEQALEGARHIVAEWISEDAENRAFL

REAYQQDALLVSQAKEEWKEKDSKFKQYYDFSELVRKIPSHRYLAIRRGEQEMVLSVRLE

VEEEPILRELEKRARVSSSSPYSKQLKMAVEDSYRRLLSTSIESDVRVDLKMSSDRDAVE

IFEQNLRHLLLSPPMGNRSVIGIDPGLRTGCKCAAVSTTGKYLDSTTFYLTKGEKGLDQA

RNDFYRFVKKHTPFAIAIGNGTGNRETETFVKNLLSQSEMKEIIVVQVSECGASVYSASD

VAREEFPDLDLTIRGAVSIARRLQDPLAELVKVEPKSLGVGQYQHDVYQALLEKKLHDVV

ESCVNQVGVNLNTASPSLLSYVVGIGPSLSKKIVQFREERGEFKSRQQLMEVSGLGARAF

EQSAGFLRIQGGENPLDSSAVHPERYELVKQMAKDLGVTVEELVGDPKRAEAIDLGRYQS

GEVGFETLKDILDELKKPGRDPRKSFEPPKFRDDVTKIEDLKAGMELEGVVTNVTAFGAF

VDIGVHQDGLVHISELSDQFIKEPSDVVKAGDRLKVRVLDVDQKLKRISLSAKSKSKGTA

PIAKKKREQAPNQKKGAFSHHPFSSL

>tr|D6YVN5|D6YVN5_WADCW Succinylglutamic semialdehyde dehydrogenase OS=Waddlia chondrophila (strain ATCC VR-1470 / WSU 86-1044) OX=716544 GN=astD PE=3 SV=1

MIRSINPATGEILWEGLPAGQAEVDAAVSLAAQAAPLWSGISFREREALVRHFGELLEKK

REAIALTISQEMGKPLWESKTEVGAMIGKIETSIAAQKERCPEKTSESARFRTCTRHKPL

GICAVFGPYNFPGHLPNGHIVPALLAGNTIVLKPSEITPFVSEKIVDIWKEAGLPKGVLN

LVQGDAETGQYLAHHPLINALFFTGSWQTGKILSELFGSRPDRLLALEMGGNNPLVLGDI

EQVDAAAYTIIQSAYLTSGQRCTCARRLIVQEKHQPIIDCLLEWLPKLKIGAYDSTPEPF

MGPLISQAAAEKVLNAQSNLLKQGAKALLKSSKINPDLPMLSPGLLDVTTASNVPDEEIF

GPLLQLVYTDSFEASVQEANRTAYGLAASLLSESQEEYTHFYRSVRAGVINWNCPTTGAS

SLSPFGGVGQSGNYRPSAYYAADYCNYPVASLESTTLTLPESLSPGIPR

>tr|D6YTT4|D6YTT4_WADCW 50S ribosomal subunit assembly factor BipA OS=Waddlia chondrophila (strain ATCC VR-1470 / WSU 86-1044) OX=716544 GN=typA PE=3 SV=1

MHQSDKIRNIAIIAHIDHGKTTLMDGLLKQSCNFRENQDVPERVMDSYDQEQERGITIFA

KHTSIPYEDYKINLIDTPGHADFSGEVERILGMVNSVLLIVDAQEGPMPQTRFVLSKSLK

MGLKPIVFLNKIDRPHADPERVLNETFDLFVELGANDEQLDFAYCYGSGIQGYAVQELGD

EKKDFIPLMDLIVRRTPVPSGEIEEPFLLQAVTIGYDDYVGRQACGRILRGKIKKGDQVV

HINREGAQSRHQITKIEGHSGLEKVEMPEAGVGDIVLLSGVPEVTIGETLCSPEKVEPLP

LIAIEEPTVSIDILVNNGPFVGKSGKHVTMNKIRDRLQREKRSNISYQITEPADDQKRVT

VSGRGELHLAVLLEAMRREGYEFCVSKPRVIIKEVDGARHEPIDNVHIEVPEEYSGAVIE

QLSKRKGEMQHLHTNEHGITHMDFLIPTRGLMGYRNDFLTTTRGLGILTAIYDRYAPWKG

EMESRKNGSLVSMLPGKANAYACFNLESRGILFVAPGDEVYEGMIVGENARENDLVVNMI

KGKQLTNVRASGSDENIILTPPRKMTLENAIGYINSDELIEVTPDTIRLRKVYLTENERK

RNKL

>tr|D6YWM8|D6YWM8_WADCW Ribosomal RNA large subunit methyltransferase H OS=Waddlia chondrophila (strain ATCC VR-1470 / WSU 86-1044) OX=716544 GN=rlmH PE=3 SV=1

MYHIKIFSVGKTKEQWLTDAVNEYEKRLCSTVKFECIWAKHDEQLKALSAKEKHLVLLDP

NGKLMTSEQFSEFLVDQLECGGSKLTLIIGGSDGIPKELKGKGPQLSLSPLTFTHQMTRL

ILIEQIYRALEIEKGSNYHRA

>tr|D6YSQ2|D6YSQ2_WADCW Uncharacterized protein OS=Waddlia chondrophila (strain ATCC VR-1470 / WSU 86-1044) OX=716544 GN=wcw_1756 PE=4 SV=1

MSMNANVGCIAGSGSCVVPIYEVSSDIDDQQKMIYSRIRKLISNSGEDSLHELADLVEAN

KEKISKPYCHLINSIILLGEVIYKQIPEEFNQTDLQTTYFTLQYLLLKKLPE

>tr|D6YUR0|D6YUR0_WADCW DUF3566 domain-containing protein OS=Waddlia chondrophila (strain ATCC VR-1470 / WSU 86-1044) OX=716544 GN=wcw_0500 PE=4 SV=1

MKKQLSGIGIKQSAKFMAVLYFILSAIFCIPVGILLIIQEGFQERGYILLVPIVYGLLTW

IFTAFVAFIYNIVADMVGGLELTLKDVDETI

>tr|D6YW50|D6YW50_WADCW Putative ferredoxin [2Fe-2S] 4 OS=Waddlia chondrophila (strain ATCC VR-1470 / WSU 86-1044) OX=716544 GN=fdiV PE=4 SV=1

MAKLKFETTGEEVELPDNSAIQEVCEEAGIPFACTEGVCGTCVIEIKEGGENLSDPTQEE

IDFLGDDPQDERLACQCKIKQGEVTVSF

>tr|D6YSB3|D6YSB3_WADCW Putative type III secretion system needle protein SctF OS=Waddlia chondrophila (strain ATCC VR-1470 / WSU 86-1044) OX=716544 GN=sctF PE=4 SV=1

MSRQEMKFLVWIPSQTYSLKTNKLKSLGGLVMTATVNPGDAANRDFIGSVKQSGFSVQNL

FSLVNIATTSAKAKLLEIQQRRSAISIGDMFEMQMLMNHLSQLSEMSTSVVSASNTAISS

MARNVKG

>tr|D6YVL5|D6YVL5_WADCW D-alanine/glycine transport protein, sodium-dependent OS=Waddlia chondrophila (strain ATCC VR-1470 / WSU 86-1044) OX=716544 GN=wcw_0809 PE=3 SV=1

MFSFLEFFENFIWEYLGVPAVVILGITLTIQSRGFQLRNLPTVLRRFFSYFFMKTEGKGV

HPIKAFFACVGGCVGVGNIVGICTAIQIGGPGALFWIWITAIIGMVVKYAEVYLGVKYRK

DHGDGTYSGGPMFFLKRATAQSWIPVTVCLLLCIYGVEVYQFRVITESLSENFHLHPGFV

SAALIVCVIFAGSGGVARVGHIASYLIPVFVFLFLGMGFWVLAHHLHQIPIVLSQVFSQA

FTGNSAVGGAFGSAIMITVSQGVRRGCYTGDLGVGYASVIHSESEVESAEKQASLVIVDV

FLDTFVICTMSVMMILTTGIWQQPMEAGMLIQTVLRDYFPGMDYFMPLFLFLLGYSTISA

YFVVGLKCAEHVSKKWGVLMFNVYAVLALVLSVFFDTSQAQSVMAIAGGLLLLINSWGIW

TLRKEISYAIQEEVILSTISGQEEEGISAPIS

>tr|D6YT84|D6YT84_WADCW Uncharacterized protein OS=Waddlia chondrophila (strain ATCC VR-1470 / WSU 86-1044) OX=716544 GN=wcw_1946 PE=4 SV=1

MKTVTLSTQTEEGTALEATFLPDKGMTLASYKLGAIETIDQSTKEDFNQFRSGLGALIGP

HFYERAARLVPKINHEFAHEKAMREKKKSDVFSHGVGRYAAWNAEINGNSIHAELSGKDL

LEGIPLSEIEGQNFKMIFDILLDPKGLKLNLSVSSDTDSVVGFHYYYAIGKEESACVRAA

VQKTYLDNLNGNKQQPLPSDWNVDSQRVLTYPLAQETDCTFHPYPDLLAGKIVLETESHS

LTINTSCPSAENSWQVWHPKGASFACIEPISSQNPRRPNLTVSQISAALEISKKNRSNSN

>tr|D6YTK9|D6YTK9_WADCW Uncharacterized protein OS=Waddlia chondrophila (strain ATCC VR-1470 / WSU 86-1044) OX=716544 GN=wcw_0095 PE=4 SV=1

MQDRSPTYLRLLLNRYHKGPHEAVLAPFPKEKAKLVSELPLRTSDIEAILYAPLENLNRI

HYSWMLDTLKKIPKPSVLYLLAMIQKEKAEKLKAALGIKSKIPSLSPLMRKFILKYLAND

IPFKDTPPFSHILESELSTLAHLQKQELIQLIDFLGLYDLSEEIHNIVDKRLLEKIYNAL

TPKKKSYVKKCLLSKEKLVTNRLNLEYWDGDPEKLSKLIHHRGMVRLGYALCNQDNNLIW

HITHILDSGRGKKLLRYINDKEIPGVTKTLREQVLDVMAFFKKGSKQ

>tr|D6YSH7|D6YSH7_WADCW ADP,ATP carrier protein OS=Waddlia chondrophila (strain ATCC VR-1470 / WSU 86-1044) OX=716544 GN=ntt3 PE=3 SV=1

MFFWPVHSYELKKLLPMFLMFFCISFNYTILRDTKDTLIVTSSGAETIPFLKVWGVVPSA

VVFMLIYAKLSNTLSKSALFYVTITPFLAFFGLFALVLYPNKELLHPTELADSLQAVLPL

GFSGLIGCLRNWTYSVFYILAELWGSAVLSLMFWGFANDIMRVTEAKRFYNLLGLGANVA

LLASGTAIIHFSDIRKHLPADVDAWQISLNFLMGMVVLAGIVIIGIYWWMQKNVLTDPAF

YDPSDVKKKKEKPKMSITESFKYLLSSKYILCIAILVIAYGISINLVEVTWKNQLKLQYP

NPNDYSAFMGGFSRWTGLVTICMMFVGGYIIRTKGWGFAAAITPVVLLLTGIAFFTFVLF

RENLSTYIAALGTTPLFLAVVIGMIQNIMSKSSKYSLFDPTKEMAYIPLDQESKVKGKAA

IDVVGARLGKSGGSLIQQGLLVAFGTISAITPYVAIATIAVIIGWLFAVRSLNKQFVALT

SDAPTEEPKTPEKTSTAAAAAS

>tr|D6YUQ3|D6YUQ3_WADCW Putative LexA repressor OS=Waddlia chondrophila (strain ATCC VR-1470 / WSU 86-1044) OX=716544 GN=lexA PE=4 SV=1

MKGLTKRQREIFDYIQEYIKSNRYSPSYREIMEHFGFLSIASVSKHLGVLQSKGAITTKK

NSRRSITPCDSTPQQKESAEAELPFIGHIMGGVPIETFPLTQTLTVPKFLVHNVEKTYVL

RAKGDSLSQEMIQDGDLLLTEARSEALAGEIIIGALEDRRTFVKQYFPEGNSVRLITSLS

KEETALRHDQLTIHGVLVGLWRLFH

>tr|D6YWP1|D6YWP1_WADCW Uncharacterized protein OS=Waddlia chondrophila (strain ATCC VR-1470 / WSU 86-1044) OX=716544 GN=wcw_1195 PE=4 SV=1

MNRHKVRLSVDCTEDERMYIKMLAAKEKKTISEFLISLARNRMPQGKIPNKETQKILRET

EEGKNLESHESLRDFWKSMGIDPNAED

>tr|D6YUI0|D6YUI0_WADCW AAA_13 domain-containing protein OS=Waddlia chondrophila (strain ATCC VR-1470 / WSU 86-1044) OX=716544 GN=wcw_0419 PE=4 SV=1

MLTSNATGANRSSSISKLDSLLYEYESEYHYLFSLVYEAANSKEKAGLQQNYPLPNIARR

LLESFLAFRLPSKSGELRQQLDFIDFDVVKKTRILRFLHTYSHSGQISDSEHDPSILIET

KQVLNDLLCLIQKDDYRHFNQMKALVTK

>tr|D6YTU3|D6YTU3_WADCW Putative rhs family protein remnant OS=Waddlia chondrophila (strain ATCC VR-1470 / WSU 86-1044) OX=716544 GN=rhs7 PE=4 SV=1

MHFYKRVYDPAIGQWLTPDPLGFADGPNLYVYVAQ

>tr|D6YUP8|D6YUP8_WADCW Cytochrome o ubiquinol oxidase, subunit I OS=Waddlia chondrophila (strain ATCC VR-1470 / WSU 86-1044) OX=716544 GN=cyoB PE=3 SV=1

MMFGNLTLEAFQHDFVQNMAVLGGVVNGILLIALITYLKRWKWLWNEWITTVDHKKIGMM

YIVVVLIMFLKGFADAMMMRIQQATATGAANPFLTSEHFQEVFSAHGATMIFFVAMGFMF

GIMNLIIPLQIGARDLAFPFLNAVSFWLFAAGGMLILISLGIGHFSVSGWLAYPPLSEVA

YSPNEGVDYWAWSVQISGIGSTFSGINFLVTILKMRCPGMTLMKMPIFVWSSLCAMVLVI

FAFPILTATVGLLMLDRYLGMHFFTASGGGNPMLYFNLIWAWGHPEVYILILPAFGIFSE

VVPTFSEKRLFGYTSMVWAMVLIAFLSFLVWLHHFFTMGASPSVNTFFSVMTMLVAIPTG

VKIFNWLFTKYRGKVHFTSPMLWFFSFVLNFSIAGMTGILLAAPPVDFQVHNSLFLIAHF

HGMVIGGVLFGFFAGFTYWFPKYTGFFISERLNRWAFFCWFAGFLLAFMPLYMLGFMGAT

RRLDHYDPSTGWQPLFVIAAMGGVIIFIGALIQFVGLFWSIYKRKDNMDTTGGNPWNGRT

LEWATHSPPPIYNFAVIPTINQRDPLWAEKPATEEKPEYEDIHLPANTPISFYIGALSMV

LGFAMTWQIYWLGVLSFAGILGLIIARLSSKDEHFTISAEEVKKIEESYSKRQQIA

>tr|D6YU97|D6YU97_WADCW Uncharacterized protein OS=Waddlia chondrophila (strain ATCC VR-1470 / WSU 86-1044) OX=716544 GN=wcw_0334 PE=4 SV=1

MNGIISEIKNFYFYYHTAGIIKKEDPNELIVKTSLCTRMDFVSAVFDGLEFLIYNVAMTI

FSAIAVTATMGFAPKFKKCFYENSYEAIVHAGSIPVSIIGIIHPKIVNRHFIKQKLQELK

IERKPDTLSSIVNLVGTIFIRKRLPT

>tr|D6YWX1|D6YWX1_WADCW Uncharacterized protein OS=Waddlia chondrophila (strain ATCC VR-1470 / WSU 86-1044) OX=716544 GN=wcw_1280 PE=4 SV=1

MFMSEVLQKFEKLADALVKDLKDGDQSEMETFESYVQRTKHEIMDEMMAFQDSFTQGYEV

ILSELASNKKEGGLPPGAMRM

>tr|D6YVM5|D6YVM5_WADCW ADP-L-glycero-D-manno-heptose-6-epimerase OS=Waddlia chondrophila (strain ATCC VR-1470 / WSU 86-1044) OX=716544 GN=waaD PE=3 SV=1

MNVDDLIIVTGAAGFIGASVVRHLNDNGRKNIILIDNLEKTEKWQNLIGKSFIDIIPIEN

CFDWLKGKESEIGGFIHLGACSSTVEKNADYLLDNNYRFSVKLAEYALSHNHRFIYASSA

ATYGNGSKGFSDDHSKLETLEPLNMYGYSKHLFDLWAKKNGVLDRVVGLKYFNVFGPNEA

HKGRMASAIVHLLSQVQREGEVRLFQSSEPEKFADGDQCRDFIYIKDAVRMTCAFFNNDA

GGIFNIGTGKAGTWNQIAHALFKALDKEPKIEYIEMPEDLIGKYQNYTRADMSKTAGVLG

QSALCEPLEASVIDYVRNYLLTGKKW

>tr|D6YSN0|D6YSN0_WADCW Uncharacterized protein OS=Waddlia chondrophila (strain ATCC VR-1470 / WSU 86-1044) OX=716544 GN=wcw_1734 PE=4 SV=1

MFTSPINSPFGSYPQPFATTNSLASLVNQLFSTPENAKEILASYMGNGVMDELLSEIWNR

PLQSDEAKLHLIEKVAPHLSFVKSMTTLFEKDSLTALFITASLPSNQRKWDVLDSLLKDQ

TGYQTLLECWQLLVSSTWEDDALKQEPLELLFADLSEEERWQLFRLCCEQCSPSEQGQLC

ILLATPSYQAEFTSAWDDVFDSNPEIDTKLHQLICSMIANHRGNSLVPPILFHLLSPEDK

NNDFGKTAFENLDQFTDREQGIILSSLLPLSLSDLIKDFDPMADRGVLISQMEKILSFSE

SAEEKTKTVGIYLELIDDEETLIAWMTELQDRFSPAALYLWPALPNNLFAFVITDPATVS

LRSNLMRIATLTKANDLPELVSYCLKNTEKIEHLFSICTLDIVSQDQMQIENETFGRWMT

FSDFANIIQTGINTGNLSEQLNQIKEDFQKLPPLLLALGMTFPSHQAWIAKAALYCTDEQ

LEAIASLLSVDDMEILDRFAESLMTDQFKLTLKHLTSKTLEAFAKHKTAACTALLQDIQQ

VHRETQARLNDLESHEQLPETEVLDDLLVILGKQTGKIMQFSHKTLNDLVDRCLSEKFGQ

EIANLQRQSKAYEADLKFFFQRIDKMRSSLPIPQKINLDDLPKNLLAFAKAATWQETGIF

SEKDLQHLGYTGEEASSLEKYLSQPELLKIWKIFISKGLGNISSLIDSCVAEPEELFNLA

DIAKKLVIEF

>tr|D6YW27|D6YW27_WADCW Uncharacterized protein OS=Waddlia chondrophila (strain ATCC VR-1470 / WSU 86-1044) OX=716544 GN=wcw_0978 PE=4 SV=1

MQRICSLNFENNQDYSLRLLLHQDMEPIRRWRNSQIDILRQSSHLSHQEQEAYWKEVVAP

SYQMNHPDQLLVAFLEGSQLIGYGGITHIDWERKEGEVSFLLNPEEIESSKNYRKKFGIF

LELIKTAAFDKLNLIRLFTETYDIRPDHISELEARGFQLKKRLTDAIEVRGRKVDALIHE

YIADER

>tr|D6YTS3|D6YTS3_WADCW Recombination factor RarA OS=Waddlia chondrophila (strain ATCC VR-1470 / WSU 86-1044) OX=716544 GN=rarA PE=3 SV=1

MQPLADKLRPKKLEEIWGQDHLLGDGAFIPSAIRSQKPLSMILWGPPGCGKTSIARLYAQ

AFAMDFRSLNAIFSGVADLKKIVHEVKQTPLFSQNCVLFVDEIHRFNKAQQDAFLPFIED

GTIVLIGATAENPSFYLNDALLSRLRVLQLKALDHEALESILERCEEAIGPLKITEEGRK

FLLDLSQGDGRYLLNLVENVSVFSSDQLLNVEMLRSRLQKRAPLFDRGSDQHYNLISALH

KSVRGSDPDAALYWLGRMLEGGEDPRFIGRRLVRMATEDIGLADPQALTISMAAKDSYEF

LGSPEGELGLAQAVVYLALAPKSNAVYKAYQKSREAASKTSQLNPPKHILNAPTRLMKEL

DYGKGYTYDHDTLIGFSGQSYFPEEMESQEFYKPVERGFEREMKKRLDYFIKKRKSAG

>tr|D6YSF5|D6YSF5_WADCW Phosphoribosylaminoimidazolecarboxamideformyltra nsferase OS=Waddlia chondrophila (strain ATCC VR-1470 / WSU 86-1044) OX=716544 GN=purH PE=3 SV=1

MSCDLLPIRRVLISVTDKSGLKKLIKGLQDHTKQLEIIASEGTAAALSEKNIPFTPLRDY

TGFPECFGGRVKTLHPRIAGGILFRKGQDEQEAEQLGIGPIDLVVCNLDDFQKASLRANP

STAQLLESMDIGGSTLIRSACKNYSNVAVIVDPKDYPSFVHELKINSGSVSLETREKLAV

KGINACADYEALLAKVLTKRIAKKDTQKPRLTRGKKLPYGENSSQKTWIYQFEEQEGLAK

AEILCGKELSYNNYEDAAVAYYAVQELLRLKAACGVAIIKHGSLCAYATGNTLNEAFEMA

WEGDSKSAYGSVIAITSPITDELRNGIKNKFVEVLIAPDFSPSFVEWASKNKKRLRMVKI

PNHLKSPFSSQNFDGKILAQMSKKILIPENFDSFFTKGENNQGIVTKATPSKELYPLFAF

GISAVNYAKSNAVAIVREAKPGCYQLIAMGAGQPNRVDSLERLAIPKAIENLQYEHGFNP

EYDPKVDLGKCVIASDGFFPFDDSIRFATSAGIKYCIQPGGSKRDKEVIEAADKEGMCMV

MTGARYFSR

>tr|D6YTB9|D6YTB9_WADCW Uncharacterized protein OS=Waddlia chondrophila (strain ATCC VR-1470 / WSU 86-1044) OX=716544 GN=wcw_0003 PE=4 SV=1

MRKDTVRLTFDFPSNLHTFLKMAAAKEGVSMRAYIVDSLMHKMDHEDKVDLDKDAFRKEL

AKMTKKDAKLMKDLSVR

>tr|D6YUT9|D6YUT9_WADCW Uncharacterized protein OS=Waddlia chondrophila (strain ATCC VR-1470 / WSU 86-1044) OX=716544 GN=wcw_0529 PE=4 SV=1

MNLLNSSTSIAQLTQRGLTLKTIQHFHLGWNPTTLFQPRTTWGLSEEIKENGNPRKLWLP

KGIVIPSFKKTIPMKIKIRRDNWTKQDPLPKYIEVSGSKPSPTIYGDTTKPIIIVESELD

AILAQQEASHLVCSVALGGVSKKPDAKLDTILRQAPLILLSLDFDEAGKKHCAFWMRQYS

NLRLWPCPFTKSLGDAFQTSPANLIRRPPFLKNKVLNTSLTIPLAVCRL

>tr|D6YSG5|D6YSG5_WADCW Putative segregation and condensation protein A OS=Waddlia chondrophila (strain ATCC VR-1470 / WSU 86-1044) OX=716544 GN=wcw_1666 PE=3 SV=1

MNQSSENTFSLDIFEGPLAFLLHLIQKSEINIHDVPIYEITKQYHERIQEWTSLSVDSGA

EFIGTTTQLLLMKSKKLLPKHDAIEEEEEDLDPHFEIIHQLLEYCYFKEAAKDLATREEK

QSTYFHRGVHNLPEVKKRLGIEHLSMEDLEALFEIALQKSNPQNKNIAEEEWRVADKISA

LRTLLKIQAKVPFRQLFSEKQSKPELIVNFLALLELMKTGECFVSKEGLSNQIYIYKGSY

DTQDNGSA

>tr|D6YUG9|D6YUG9_WADCW Uncharacterized protein OS=Waddlia chondrophila (strain ATCC VR-1470 / WSU 86-1044) OX=716544 GN=wcw_0408 PE=4 SV=1

MKLLGRSGIGQGIERGAGVVVFDLAMLVSIGFMVWGLTIAFSEVFLAKSAPENSGHLSEW

PISIITNVFLISLIYSWTIKREQTLYKTKRIMVEHFHSGDIEEEGHGLQGESLNRLVGAC

NHLLDMLAEKCQFNTLPSQYRLDPDRL

>tr|D6YRY2|D6YRY2_WADCW Uncharacterized protein OS=Waddlia chondrophila (strain ATCC VR-1470 / WSU 86-1044) OX=716544 GN=wcw_1478 PE=4 SV=1

MDFTREPIIESVITPKEGCKLVIRSSKGAGQEEFFVDAVEVVSFGNTFFLRSMERPKAFL

VPASDYEILEVRETRMVLKNVGLDRSIKIGGGKETPKKTAVKESPKVEQEKTSSDQKTEQ

PKKREKRRHYRRRKGRDRISDETQDDGKIDLPEPKQVEDDSPQDLGEPLTPNSQIIGSLL

PPPPNLISETIERYKGNALFKDVFYSDSDSESETDEEEIEEEESFVELEESDPPKLENPT

EKNEEEAESSQETNSIS

>tr|D6YSP2|D6YSP2_WADCW Methyltrans_SAM domain-containing protein OS=Waddlia chondrophila (strain ATCC VR-1470 / WSU 86-1044) OX=716544 GN=wcw_1746 PE=4 SV=1

MCNFYQLIDSGHEKKLEQFGLYRLIRPAAAAIWHPKLPQEEWDKADGVFSREGKSHWKRP

IKKTWNVKIGGIAFKLAATDFGHIGVFPEHEEVWHWSADRIKPGDHVLNLFAYSGGATLA

CVKAGAEVCHVDASKGTVEWARENAALNNLDAAPIRWIVDDVVKFLKRELKRGRRYDGII

LDPPTFGRGSQGQVFKIERDLPALLGMLAQFKPRFISLSCHTGSFTPLILKRLLQDPFNG

GVFEEGELQLKGPGAYLPNGTYARWVRD

>tr|D6YTP8|D6YTP8_WADCW Uncharacterized protein OS=Waddlia chondrophila (strain ATCC VR-1470 / WSU 86-1044) OX=716544 GN=wcw_0134 PE=4 SV=1

MNCLSSLEAGMPVLGSLLPSEDAKCSSIDRLYVRTMIYQRALEALAAFETCRFQSLDVPA

GDQACQIRAFQLCMLNLKGLDVLKKTIKSRLDALADLEKIFASLDKECKVQVAQIEREKL

MPLVEENRLIGIERRKANLSKGDPLCEEFAQAFQAVKLKKQPILKEISELQQACRVKQKM

MIEDDGLEMTADPSAVFIIRSYLVTLAKVDVIREEMGIFTYQIHSSPKSLQEEGFVVPTS

QLIEIVEKAKKAICLDSIAFVQKEALLIAGNNGERLQKYTANLRVLESKERSELPFFYLT

QVIFLRAMAEEIPVLLKVRNIGSHPLEQENFACSTLFKSDGSSYKVSPVLSDDLNRRVIV

IEGFSKQAFEALQTLDYVEDTMEKSGGLLRLIDLNTAQHGQYTDTKSSKKMFRKIPGMVS

DEEETLLSLFEEAVSKGFSLDNSEQLCIDHVFCDLLANQRTG

>tr|D6YWD6|D6YWD6_WADCW Uncharacterized protein OS=Waddlia chondrophila (strain ATCC VR-1470 / WSU 86-1044) OX=716544 GN=wcw_1090 PE=4 SV=1

MKSIKMPFGLNSDGNLVHISEVVNGKKCDCICPSCKAPLTAAKGNIKQHHFKHAIDSGCE

SGLESSIHLAAKEIIKEKKTIKLPENVLVLEKKDSKGLPHHESTIIVEAGLLIKFDFVEE

EKIIDGMIVDLLAKKQEKQLIIEIYFRHNVDDEKIQKIKNSNISAIELDLSNLSPEDLID

RNTFWNYINNPERAQWLYNSTHQDEYLKLQKNLDQKIEKKEQEYIKEREKEMKRLTRSIE

EVKRIKEDSSLIINLDEFKNNFNLYSRNIPKFLNLNILPDLLNLSELPDFINLKVQDGDW

IYGSEGCVWQLIVYSMLYPRVGEIMTIKFTDKWLKKILVSKVHNPVKNISILRERFPEIV

SSNLPGDIPNTWKTLRTYFNYLCKLGMLSNMGHNCFFVEKNNSCKQGDFMKKI

>tr|D6YW96|D6YW96_WADCW Leucine--tRNA ligase OS=Waddlia chondrophila (strain ATCC VR-1470 / WSU 86-1044) OX=716544 GN=leuS PE=3 SV=1

MKYNHQAVEKKWQEHWEKEKTFKVDVDPNKPKYYVLDMFPYISGAGLHVGHVLGYTATDI

LARYKRQLGFNVLHPMGWDSFGLPAEQHAIRTGTHPAETTEINLANFRKQLKSLGFSYDW

DREFGTHEPTYYKWTQWIFTKLFEKGLAYEAEMPVNWCPALGTVLANEEVEEGKSKEGGH

EVIRRPLRQWVLKITDYADRLLKDLDLLDWPESLKRLQKNWIGRSEGAQVKFPVNGTKHL

LSVFTTRPDTLFGATYMVLAPEHPLVSEIAADSCKEAVETYQRQAAMKSDMDRTDFSREK

TGVFTGAYAINPANGREIPIWISDYVLIGYGTGAIMAVPSHDQRDFDFARAHHLEVIPVF

DPNIESHPELVPETLSHDELRELVIAGKHCWEGEGVCINSANEEVSLNGLRVKEAVEKML

AWLEKKGYGKKTVSYKLRDWLFSRQRYWGEPFPILHLADGTKRVLELDELPLTPPNLSDF

KPAGDGQSPIAKVDDWVNIADPKSGQQAKRETNTMPQWAGSCWYYLRFCDPHNHDEAWSR

EAENYWMPVDLYVGGVEHAVLHLLYARFWHKVLYDTGYVSTPEPFQSLRNQGLIVSRSFK

DSGNNYVPTALVKEKDGAYFHKETGEELTSQIEKMSKSKLNGVVPDEIIAEYGADALRLY

EMFMGPLDKEKVWNTDAVNGCRRFLQRFYDACASDKVTDIDHEDALKLGHRLVHDVSKDI

EQMSFNTSVARFMEFMNDLSKLEAYPRSVMKMAIQTLAPFAPHLAEETWEMLGFKEELSR

CLWPEVDPKYLKNDRVTYVVQVNGKVRGRLEMDKGLSKEDLMEYAVSDPHVVKFLDGKEI

RKVIFVPDKLLNLVV

>tr|D6YV99|D6YV99_WADCW Rhomboid family protein OS=Waddlia chondrophila (strain ATCC VR-1470 / WSU 86-1044) OX=716544 GN=wcw_0693 PE=4 SV=1

MIYSMTMTTSSFRLGPEFTPAAVRKLMIYTLCMSIGSVFFNPFFTQIFGIPGPQEWFSLS

WWGLQHYLLWQPFTYLFIHPIGYGGIGFSYFIGLIFNLYILWVMGSEICQRINEKSFTRF

YLICGIISGIAILFFMPVIGQYAVLTGPVSSIIAILIIWAFLNPEQELLLFFLLPVKAKW

LSLGILGVALLVSISGLQFISFFHYFFGAVSGYLYGLIIWGIHSPFPFMYKTELAIIRAS

QKVRQQILRKKSTSKSKVVNIQTGEPDDDEAFVDSILEKISKYGESSLSFSERKRMKAIS

EKKSKY

>tr|D6YWV6|D6YWV6_WADCW NADPH-dependent FMN reductase OS=Waddlia chondrophila (strain ATCC VR-1470 / WSU 86-1044) OX=716544 GN=wcw_1265 PE=4 SV=1

MNEHPKIIAMAGSMRAGSWNKKLVRIAAAGARKAGAEVTILDLSDYRLPLYDGDLEEAEG

VPDKAKALKKLFAEHDGFLFSSPEYNSSISGTFKNMIDWISRPEKGEPSLVAFKGKSAAL

MSASIGALGGLRGLVHVRAILGNIGVLVLPDQVAISSSQEAFNEDGQLKDPKKLEQTLGL

GRQLTELLKKTTID

>tr|D6YW69|D6YW69_WADCW Uncharacterized protein OS=Waddlia chondrophila (strain ATCC VR-1470 / WSU 86-1044) OX=716544 GN=wcw_1021 PE=4 SV=1

MGRYTACSRSFSSWGSKTSSTLGNAMKKNETDNFSWLQEWFYKNCNGDWEHNTNIRIVNL

DNPGWGVFINVSETVLENKKFQIIDDYRTENDWIYCVVENQQFKAAGGPFNLLEMLRVFK

DWAES

>tr|D6YW45|D6YW45_WADCW 3-deoxy-manno-octulosonate cytidylyltransferase OS=Waddlia chondrophila (strain ATCC VR-1470 / WSU 86-1044) OX=716544 GN=spsF PE=4 SV=1

MNMMEKVSIIIQARSGSSRLSQKAFAEVCGKPLLWHVIERAKLSKRISDIIVATTTRSED

RAILELAKSCNVHAYAGSEEDVLQRYYEAALKNGADVIVRMTGDCPLIHPPTVDAMIALL

QEKKADYVCPDPRHRSLETGLEVFTMKTLREMHEKAVENYQREHVTLYLREHPESFKIAL

HIPDQIFQRKDIRITVDYLEDLELIRIIYRELYREGEIIDLKKVVEFLDQHPEFKDLNIH

AKLSKANRLSISDAISEKIIRSVEKEKNG

>tr|D6YUD5|D6YUD5_WADCW tRNA pseudouridine synthase A OS=Waddlia chondrophila (strain ATCC VR-1470 / WSU 86-1044) OX=716544 GN=truA PE=3 SV=1

MKKYKLIIAYDGTQYSGWQMQPNALSIQEVLEEKLKVLTKTRTSLTGAGRTDAGVHAIGQ

VAHFKVEAPFNPSLLRLSLNGLLPKDIRIMDVEEVPLDFHARYSATNKIYYYHLNLGPVQ

DPFEKQYSWNITNSLNMELLKKGISYLIGTHNFSAFANEANQGAAKKNPIRTLKRISVVP

ERIGMRLEFEGESFLYKMVRNMTGTLIDVAKGKLSPEEIKKILESKDRRNAGRAAPAKGL

FLVRIDYVSNPLKS

>tr|D6YWW4|D6YWW4_WADCW Putative membrane protein OS=Waddlia chondrophila (strain ATCC VR-1470 / WSU 86-1044) OX=716544 GN=ompA8 PE=4 SV=1

MINMKKAVAAILLTFASLTTTAQADCWDSSCNDCCPDITVGVDWLYWSPCINDRHFALTT

DGVDVNTHYFCNEWDFGSRVYAKLGNWWNGFNGALIYTYINPKTSSSLKSDAIILSVAAP

DEIPSFNDLDAKWELQYQTLDAVLSYSIDVTQNRCFKIEVFSGLTWIDVKQKTEYVFDLN

EGGFTNRNFDRQNDFWAVGPCLGLNSSFSFFDCFKIFGTLKTNLVVGETASKDVLFRKVA

GDEKDSVKDEILTTCKVKDRCFCLPGLHLASGIEYEICLYETTFGLRLGWEYVQWINAPT

FPCYELDGSRVRSAPSTNNLTMQGIFFGLNSTF

>tr|D6YT37|D6YT37_WADCW Uncharacterized protein OS=Waddlia chondrophila (strain ATCC VR-1470 / WSU 86-1044) OX=716544 GN=wcw_1895 PE=4 SV=1

MISLGGSSLHIKPFNQTLQDFVRVVSCEKSNSINTTNASEQEHMLDYLIQLMQYCVRFDQ

NNIPKELHLHPEFFALPNVETFFTSLFDNLHESLFPKAKNVFLQGLSFIVKYIGLPKSRE

VMYGLMEKLQNRFPTLKEMHDKAIENFIEEFFRPTIGEFLDKYKVNKRLLQIAFPTIYEL

FVTPLDTVHDRKLFNFYCNPWIYQPIMDYLAKYPQSNEAPLNFLEDRLQAIHASTTTESW

EELSKKAHNLCTHSSNLEQFAIACCGLLGEIKTAAQIRSKGTVIFLPEKSDEGKNCDLLV

IDPIGKLELIECKAKTPRHGLEETTAGVTQIWDDFFTNFSLAIHSYVDYHQKAIQNPLGF

SECFPLLSAFEGSSYAQALPLIQSIPSTTGNVTLKKWTAEQKISHLLRALFLRPLVLDPC

CVPLPPEEERLTQRQQATETTIKNKEWVISIFNKATKQLEDTYHRLTAEGQTVNKLLVAL

DLELSYRLLHDHFSYNNGNIAEVAEQALYETFEPYRTTFAKKNLNLGLLLIQP

>tr|D6YT96|D6YT96_WADCW Transposase OS=Waddlia chondrophila (strain ATCC VR-1470 / WSU 86-1044) OX=716544 GN=wcw_1958 PE=4 SV=1

MNVRLGDDRICFLVILGALPNGKKELVAIHNGYRESKSIGEFEAARAVYSS

>tr|D6YV02|D6YV02_WADCW ADP,ATP carrier protein OS=Waddlia chondrophila (strain ATCC VR-1470 / WSU 86-1044) OX=716544 GN=ntt2 PE=3 SV=1

MQKFKSHYFPLVFVSFFLAFNYFILKALKETLLVTAPDAGAEAIPFAKMWLLFPISMVFL

AVFTWMATRISFRFAVSSIIVFFLSSYAFFTFFCYPLRESLHLNSVADQMQLILPAGWKG

LIAVVRYWSYSLFYVTAECWCTMVYSVVFWGYANAVTKFHDAKEIYPFLTLAGTTAALFA

GPIAIFLTSSRFSHLFANPFDFSLYTLTTLVLFCGIAALIIFLKYCPSVPSKETEAKEKK

HFLKTFIGVFKSRYLMILASIGLTYNLVINLSDVVWKNEVLKLYPDPQDFTSYLSYVTLV

TGLISTVFTLFICRPFLKHCGWTKTALLTPLVTLATAAVFFTALFFSERDPVVIPLSTVA

FLGSLHICLSCGGKYTLFEPTKELAFTPLSQSEKIHGKAAIDGVGARVGKTGSSMIYQAL

LIGLPTVSACAPVVGILVIIGISLCIRCVFLLGKKVDQQLAKT

>tr|D6YVZ5|D6YVZ5_WADCW Putative short chain dehydrogenase/reductase family protein OS=Waddlia chondrophila (strain ATCC VR-1470 / WSU 86-1044) OX=716544 GN=wcw_0945 PE=3 SV=1

MESQVVLITGSSRGIGRLTALELSRRGHRVYATMRNPEPIENVHVERLDVTDEKSIAEAV

SNLIEKEGRLDCVINNAGYGLLSPVDTATDDEIMKQFDVNLFGVIRVIREVLPQMRKQKS

GKIINISSVVGVVSNPAMGWYSATKHALEAVSASLASTVFPWNIYVSVVQPGSTATEFAA

NLKLEEGGGNSPYGDFSKKHQERMKNILTDGQPPEEVAELIADVVEDPKPHFRYQTSERA

KQIVSQFVVDPSGDQWLNQQKETFKGWINT

>tr|D6YSV6|D6YSV6_WADCW Uncharacterized protein OS=Waddlia chondrophila (strain ATCC VR-1470 / WSU 86-1044) OX=716544 GN=wcw_1812 PE=4 SV=1

MVMMDQIDNVYRHSFSIFKYKISLYNALFMLIFYKKLFKKLF

>tr|D6YVP7|D6YVP7_WADCW Corrinoid adenosyltransferase OS=Waddlia chondrophila (strain ATCC VR-1470 / WSU 86-1044) OX=716544 GN=wcw_0842 PE=3 SV=1

MAKIYTRSGDKGETSLFTGERVLKNDPFIEALGTVDECNSTIGLAIAHMPDNEIYSGLKQ

QLVMIQHALFDLGAAIATPRTRAKEDKINKTRFDEEEIEILEKWIDEMDLQLPKLKHFIL

PGGHPAGAMLHLARSVCRRAERQVIPLSRHSDVSDKITRYLNRLSDYLFMASRWNNHLAN

MPETPWKQHLAIK

>tr|D6YWK3|D6YWK3_WADCW Exodeoxyribonuclease 7 small subunit OS=Waddlia chondrophila (strain ATCC VR-1470 / WSU 86-1044) OX=716544 GN=xseB PE=3 SV=1

MTKKEPDFEKNFQRLEEILEAMNSGEAGLDKSLKLYEEANDLIIACGKRLNEAEQKIEKL

IKQRDGELALSDDGAPAVESFE

>tr|D6YTH0|D6YTH0_WADCW Uncharacterized protein OS=Waddlia chondrophila (strain ATCC VR-1470 / WSU 86-1044) OX=716544 GN=wcw_0055 PE=4 SV=1

MLIPFNFLTAELAPDQIQNLEEQKKAYGEIPEREIKYYEETAPQNADKQRNFYMHDHPLQ

EDSVPDDTVK

>tr|D6YV35|D6YV35_WADCW Putative rhs family protein remnant OS=Waddlia chondrophila (strain ATCC VR-1470 / WSU 86-1044) OX=716544 GN=rhs21 PE=4 SV=1

MFQESVSPWRFSSKRVDEETGWVYFGRRYYASSWGRWTTADPAWFADGPNLYAYVHNNPL

KYVDPDGLSAIEHQQMNRLGTQGTFFGSFSRGILDDTSWGASSWMLGDYVCDNWQSSLGY

GMGTGVSMMAGLVYGGTEAKLLGAAGKGLNRAARWLNFAEKEVKAACKMEGTAAKISRDI

APKIERNLPDVGKGSYVPGVGGSPKSMPEGHMWTSGSPFKNKTAEELHQMFVDKGYDYGA

RSPELLIRGKGNYINPKNGRQFHIDPKNSGRYREPNHVDVSRPEKYNGSLPKKRFSYLDD

>tr|D6YRK6|D6YRK6_WADCW Uncharacterized protein OS=Waddlia chondrophila (strain ATCC VR-1470 / WSU 86-1044) OX=716544 GN=wcw_1350 PE=4 SV=1

MFNFNFPISIGFEVDASDGSQRVYVTCTVKDLKNARMLERLFNEVIGGFETISSGNKARI

FCQLHQKDDRDATKGDRGNTIADAFIRVINVASRLFPHATVSSQASSVLENLVPTGDLYL

SRFEDLETLLPKPEEGDSLVSVFYQKFDKAELEKFQLSEEERGQAIEGAYVSINEAIEED

NKKVEEFKARQDVKISSEASEASHSLPKVFPASKKETQGVFSHISRLFVRFWKWFSAFFR

>tr|D6YTN3|D6YTN3_WADCW Putative TolA protein of Tol-Pal system OS=Waddlia chondrophila (strain ATCC VR-1470 / WSU 86-1044) OX=716544 GN=wcw_0119 PE=4 SV=1

MNKQRLWILAVSVILAHLCVVIWLHYGGVPESRFPLQEKLVVRTIHLTQEKKAETIPKKL

PAQIKKNAPKKKQPLLKKTEKKTEPRQEKGLRQEERMESLIAQARQQLSQVGKASKAMDQ

AKRVETLKIDGVEDHGYYSQLAACLKENLRLPEFGEVRVSLTLDCRGKVIQVKIIGAQNE

NNKRYVEKILPKVNFPSFDQAFKNEKEHTFSITLANDL

>tr|D6YU83|D6YU83_WADCW Integral membrane protein, TerC family OS=Waddlia chondrophila (strain ATCC VR-1470 / WSU 86-1044) OX=716544 GN=wcw_0320 PE=4 SV=1

MDWIADPNGWISLSILTLLEVVLGIDNIIFIAILVGRLPEEQRKSARMLGLSLAMLTRIL

LLFSLSFIMRLTSPFVSILDFSFSVRDLILILGGLFLIAKSTLEIHRKLEDHEQPAIAPK

YGQYFGVLIQIAILDIVFSFDSVITAVGMANELAIMVIAVIAAVIFMMFFVHSISCFVER

HPTIKMLALSFLILIGVVLVGEGLSFHIPKGYIYFAMAYSMLVEMLNLRMRSKD

>tr|D6YT80|D6YT80_WADCW Uncharacterized protein OS=Waddlia chondrophila (strain ATCC VR-1470 / WSU 86-1044) OX=716544 GN=wcw_1942 PE=4 SV=1

MANTLSVHNSLASALNYEYECHTKSRVLGKVHKFSGSWIEPRERFAVAAYEAQVAAIHSL

ASAIFHTLWSMTAGKLFTDKEARHFAVQSWKDFGDHLVSALKGFLGVISPTIADWADKKV

MTVRSNEEEGIELKDQRKIDEPSIEVNED

>tr|D6YV13|D6YV13_WADCW V-type ATP synthase beta chain OS=Waddlia chondrophila (strain ATCC VR-1470 / WSU 86-1044) OX=716544 GN=ntpB PE=3 SV=1

MKTVYDRINDMRGNLVTVTAEGVGLGELAQIQLKDGRSVYASVLRIDGKEVTLQVFQNTR

GISTEDKVTFLNHQMQATYGNSLLGRRLSGSGKPIDGGPSIIGEQIDIGQPSFNPVKRII

PREMVRTNIPMIDMFNTLVRSQKIPIFSVAGEPYNQLLMRIANQTDADVVVIAGMGLTFN

EYQAFIDNAEQAGSLNKTVMFIHRATDPAVECMLVPDMALACAEKFAIDDKNVIVLLTDM

TAFSDAIKEISITMDQVPSNRGYPGSLYSDLASRYEKAVEIEGSGSITVVGVTTMPGDDV

THPVPDNTGYITEGQFYLHGGQIDPFGSLSRLKQLVIGKVTREDHGDLANTMIRLYADSK

KSKERQSMGFKLSRWDEQLLSYSKLFEVQMMDWNVNLPLEEALDTGWRMLAQCFKPEEVG

IRDDVLKKFWPK

>tr|D6YSD7|D6YSD7_WADCW Protein GrpE OS=Waddlia chondrophila (strain ATCC VR-1470 / WSU 86-1044) OX=716544 GN=grpE PE=3 SV=1

MIKDDTTTPDEEKDVEITVEEASEEVDYKSKYLHLLADSENARKRLQKDRDEIVQYSLRS

LLQDFLSPIDHMENALNYTGQASEEVQNWAKGFQMILAQFKDVLASNNVKSFESVGKPFD

PHIHDAVEMKESAEHPPGTVLEETMKGYLIGDKTLRPARVVVSKVPVEELKEKEDKEVKS

>tr|D6YSX1|D6YSX1_WADCW Multidrug-efflux transport protein OS=Waddlia chondrophila (strain ATCC VR-1470 / WSU 86-1044) OX=716544 GN=acrB PE=3 SV=1

MFSHFFIDRPILSSVISIVIVLAGFMAMVNLPIAQYPEITPRQVQVTTAYPGASAEVVSN

NVAAPIEQQVNGADDMLYMYSSCSSTGNMTLNVFFDLSRDPDLAQVDVQNRVNLALPQLP

EIVTRQGVSVKKVSTSFMMIIAVFSPDGRYDNTYVGNYANLYILDAIKRIPGANQASVIG

VPDYAMRLWVKPDRMAQLGITSQDIIRAVQAQNEQFAAGRVGQPPNMDEVVMTFPVTTQG

RLTTPQEFEEIILRAGSEGAAIVKLKDIGRAELGSKDYNVTTHLNKMPATLIAVYQQPGS

NALEVSEAVTSLLKEMKKDFPDGIDYKISMDTTQFVRASIHEVVRTFFEAAVLVTLVVLI

FLGTIRATLIPLVAVPISILGAFIGMILMGFSINMLTLFGLILAIGVVVDDAIVVMENVE

RNMAAFKLSPREAARRAMVEVTGPVIATTLVVLAVFIPVGFMGGVTGELYKQFAITIAIS

VGFSSIVALTLSPALTVILLTPDMKKKGFFQWFDRKFDWINEKYASLVEFFLNNKLISAG

VFIVAIISSWWMFATISTSFVPEEDQGYLLSVYMLPDASSLDRTAEVGRQVEKIFLDQPG

VSDVATGNGYSLLDGQLKTNTGVAFVALDDYEKRKSPDLQAEHIMRATGAKMMQIREGVA

FPINPPPIPGLGSVGGFEFWVQSQGTGTYQQLGEVINKVVEKAGERPELGNLTATINTDS

QQLFVNLDRERAEVYGVPVQEVYDTLQTLFGSIYVSQFSKFSRLWQVIVQAESSYRTVPE

DINQVYVRNKNNDMVPLSSLVSMEYVAGPDIVTRFNNFPAAKINGNPAPGYSSGQALNAM

EEVAREVFPPGYGFAWSGQAFEEKKSGGTSAVAFIFGMVMVFLILAAQYERWSMPFAVIL

ALPFALFGALLAIWLRGISNDVYFQIGMVALIALAAKNAILIVEFAMQKYEEGFSAYESA

LIAAKLRLRPICMTAFSFILGCVPLAMATGASANSRHSIGTGVIGGMLGATVIAIFFIPL

FYLLLQKISGDHKEKSDEE

>tr|D6YW51|D6YW51_WADCW Ras-GEF domain-containing protein OS=Waddlia chondrophila (strain ATCC VR-1470 / WSU 86-1044) OX=716544 GN=wcw_1002 PE=4 SV=1

MSIDSSTGFDSGALTHSQFTFNEKDGTVKLAASGKKYTVQIGGSTLDRNNKELIKMITDI

LDQVGQQEEFEYKDLSQTTITDEGVKTDQKEEEPRTYKIETHDESYKKITEIAQKVLSKP

PVDSGRLVAEEGKGQNVEDLTKQSGSSSGFLEVEFEVLERGKLPETTEKVVSFSPVPQTK

REKLKQVKERVLQFVSRSANFLIEKFKKTDHKTKTPSIEIELDKIGKSDESDELDKEIGR

EFTKCPLSKNSETDEFDHTTLSLKALEKIASETVNIDGKVKIRKEKKQSFIRAGHRIGKL

KKRERGVGTGKKSLEAMKVVLKACQHHYSDVKEQHTLYSSVLEKFVKMEIKEEYFDIDQW

LSEIEKNVPEEIKKSQELQDLKTALEEYKEKKDLSKIKDAKKNLEKQLKTKLNSLNQESQ

KIIRIVTSLTKLSTWGKAIMENYNQTLKPIISEIQPLKSPKDKWLKKEKFFNQVFNPEEE

ELRSNFEINYRWMMSSSEYIDLLKNRYQSNEKKLKDEEILDKKYLKEEQEIILKMVFNWS

FSDKIAPSEIELLSDFVGLLDDRYQSNEEKLKDEEILDKKNLEEEQKIILEIKNKIEKKE

VPAVSDVTPEDATQDFQADLAGVAAGKIKSKKREELLNKYLADITAFTSNTYLKILPSEF

AKLAWSKSEKASTSPNLSKFSTQFNNLNQYFQYVILTVPDGKGGRKPSTIEEQARMIEFF

IDLQTKAVKQGDLNTVAIINSALDSSSIFRLKKAWNDVPIKKIAQQKRNASYTSPESSYK

AAREFQEGHPGGIPFMGIFLQDLTFFNDGNKLYQEDDSEVVNNDLIEKFSTTIKTALQGQ

QGRTMMRPKRNIEEEIKLASKQDDNTLYALSLQAQQRE

>tr|D6YWT0|D6YWT0_WADCW GTPase HflX OS=Waddlia chondrophila (strain ATCC VR-1470 / WSU 86-1044) OX=716544 GN=hflX PE=3 SV=1

MYEEIREKLGDEEASKERRALLVAIYRGTDQLPECKEHLDELELLCKTYGIETAEKLPCL

VRKFNASTLISSGKVEEIGETAKELGVDVIVFDDEVTPGQQRNLEKIYSIPVIDRSEVII

GVFAQRAHSKEAKLQVELARLKYLAPRLKRMWTHLSRQQATAGGGAYLKGKGEKQIEIDR

RLLKTKIFHLQKEINAVQAHRDTQRIQRERSEIPTFAIVGYTNAGKSTLLNALTEAGIFT

EDKLFATLDTTTRKFTLPNNQEILLTDTVGFIRKLPHLLVAAFRSTLEEAVQADILIHLI

DSSHPMALEQAEATVEVLHELGAKDKPVITVLNKIDRLEDRAILKKLRTKFTKTVNLSAL

YKQGFDHLTEQMTEELQKRRRVVNLRIPQKDYAVVSEVMRHGNILNQEYEENDVVMRVDL

PSRYLGRISRYIEE

>tr|D6YVJ1|D6YVJ1_WADCW Putative cytochrome d ubiquinol oxidase subunit 1 OS=Waddlia chondrophila (strain ATCC VR-1470 / WSU 86-1044) OX=716544 GN=cydA PE=3 SV=1

MDVEILARMQFAFTIMFHYIYPPLSIGLGLLLVIIEGIYMKTGDQSYLRMARFWTKMFAV

TFAIGVATGIVMEFEFGTNWASYSRYVGDVFGSALAAEGVFAFFLESGFLALLLFGWERI

SPRLHYFSTIMVCLGAHFSAIWIVVANSWMQTPAGFHIVGDGLSARAEIIDFWAMVFNPS

SVDRLLHTIVGAWLAGAFLVISVSAYYLLKGRHKKFAVNSLRVALMVAGVSVALQAVIGD

MSARGVAKNQPMKLAAMEGIYQTQKGAPLVLFGVPDSEAKLVKKEVSIPKFLSFLSFHDF

NAEIKGLNEVPRADWPNVKAVFTTYRMMLFMWGLMVVTVILAWWKWRKGTLAESKGVLRL

MAVSVIFPQVANQAGWVAAEMGRYPWIVQDLLRISEGLSKSVSAEHVLGSLIMFTAVYLF

LFFMFLYLFNEKIKHGPDEPEAETPYHSLHVLAERFDNE

>tr|D6YWF0|D6YWF0_WADCW Uncharacterized protein OS=Waddlia chondrophila (strain ATCC VR-1470 / WSU 86-1044) OX=716544 GN=wcw_1104 PE=4 SV=1

MTTQSVKEKDFVFSSSEFPRLSEGIPGVVQEIDQLHSAVLKAHTIEKVRILELQLGERIR

ELSELFTQLFDTANEVSKLALPLFNKNKGRKGAWQTEAEARKKAIEAHKELDALLKKQVS

YRLALSEVTVTPNYLPQVQDLSSSLIELSNSLQELMTEALNPSQQPDIEKIKLQLAEKRE

RFEELGAQVEGLEDPVARKAIALDKHIEETREHTGMIAINKAVCKWGLTRINIFVDILSN

IVDLQKKMAGLSRDAENLKTYLRENPYTSADPVKLCIRAILNDPGERTLKAVEKEMKELL

AQYPETISSGAKKALLSILDEKPETKITEARDLIEAVVKKLPDSPIDNATRDFAAIVRVY

NESKSQAEAYREVTEKAIDDLSKLIENEEKNLIATIKGIPFHDPSVLNQISKQMEEAISR

MQGERKTLLYKLKETWKAITEQLSTEQKGQLLYEINRLGYAIHENHGALPRDFTYGRLWS

INNPYVESMATKST

>tr|D6YSK7|D6YSK7_WADCW Arginine--tRNA ligase OS=Waddlia chondrophila (strain ATCC VR-1470 / WSU 86-1044) OX=716544 GN=argS PE=3 SV=1

MHKTLIQIFYSAFISAFPQLKEKSVEIDIAQSKQEKFGHYQCNTAMRLTKQLHMPPREIA

QRIVESVDKGDAIEKLEIAGPGFINIFLSPAYIEKKVQTMAASPRLDIALPEKTEKVIVE

FSSPNTAKELHVGHLRSTIIGDCLARVFEFLGHDVLRLNHVGDWGTAFGMLIAYLKLSHP

EILTGEEETDLPHLVSWYKASKKQFDEDPGFKKISQLEVVSLQGGESDSLKAWEIICEIS

RRSYQEIYNLLDVAIEERGESYYNPVLPELIEELDQKELIEISEGAKCIFIPEFCNREGS

PMPLMVQKSDGGFGYDTTDMAAMKQRVQEEKADRIIIVTDAGQALHFKLVYAASVKAGYL

DPDKVRFDHVTFGLVLGADGKKFRTRSGEVERLIDLIYTAIDKAREIMAERNPDMPQEQR

EKIARALGIGAIKYADLSCHRTGDYTFSYDRMLRFEGNTAAFLMYAYVRIAGIKRKIGAD

IDALQAAISLKHPSEMALGLHLAQFNEALQQVAEDLLPNRLSDYLYTLAEKFNAFYRDCQ

VEGAPEQNSRLLLCEATARILKQGLHLLGVETVEKM

>tr|D6YRK2|D6YRK2_WADCW Uncharacterized protein OS=Waddlia chondrophila (strain ATCC VR-1470 / WSU 86-1044) OX=716544 GN=wcw_1345 PE=4 SV=1

MEEPMFRLKTTLLTLLFFCMTISGFAATPQLLQTLGSTNLDLLKNAQYVTILAMYEKLPE

IHLTNDEIARLKRQLLDDHNYSFNQNQPCKFVPEISFKFQNTEGEAIHVFVSPSCNQILF

SMGGKSALLNYEPAHERLEHYFQQLIKNTRMKNKVS

>tr|D6YUZ7|D6YUZ7_WADCW 50S ribosomal protein L10 OS=Waddlia chondrophila (strain ATCC VR-1470 / WSU 86-1044) OX=716544 GN=rplJ PE=3 SV=1

MRSEKQLLLDEIKEQIEGHDSFVIMQYSGLDANRTSDFRRQIADHGGSIEIVKKRVLIKA

ANELGIELELDQLPGHIGIVFTGEDSIQATKAVFELRKATNNGVNVLGGQFDGRLLNAED

VETLSKLPGKDEMRAQLLGVFAAPMQQTVGAMNALLCSILYCLDNKVKKEEGSN

>tr|D6YVX7|D6YVX7_WADCW Putative (Di)nucleoside polyphosphate hydrolase OS=Waddlia chondrophila (strain ATCC VR-1470 / WSU 86-1044) OX=716544 GN=wcw_0927 PE=3 SV=1

MKREFTSTVYILENDKVLLIFHKKLQKWLPPGGHIDPNETPPEAARREAFEETGLHISII

PQENIWVDNWNAKSIERPYLCLLEEIPEHNGVPAHQHIDMVFLARPQGGKLIENPEETDG

LRWFSWDEVEALTSDQEIFAETKAVVKTLIGVSAYA

>tr|D6YUD8|D6YUD8_WADCW SWIB/MDM2 domain-containing protein OS=Waddlia chondrophila (strain ATCC VR-1470 / WSU 86-1044) OX=716544 GN=wcw_0377 PE=4 SV=1

MTKQKKPSAFMKPVGVSDALAEIVGKGPMARTEVTKKLWDYIKKNKLQDPNNKRNIVPDQ

KLAKVFGSTQAIDMFKMTSKVSKHLHEAEAAGAK

>tr|D6YSC4|D6YSC4_WADCW Uncharacterized protein OS=Waddlia chondrophila (strain ATCC VR-1470 / WSU 86-1044) OX=716544 GN=wcw_1624 PE=4 SV=1

MMAKTVLCCIFLHSALIAEDPWGKGCALVCKPYERSKKPRSFIGENLIRFHKRVISPADG

PRSHHKPNSSQYTIDAMRKYGFFQGYLMGCDRLMRENEDPWIYQTVMDEKGETFKWDPVK

>tr|D6YS37|D6YS37_WADCW tRNA uridine(34) hydroxylase OS=Waddlia chondrophila (strain ATCC VR-1470 / WSU 86-1044) OX=716544 GN=trhO PE=3 SV=1

MNPYRVLAFYHIVPLENPHNEVLRHKIFFKGREITSRIYISEQGINAQMCASIQDAEDYI

SWLQENPLLKDASIKIDEWHEQVFPRQTVKYRKNLVACDQSIDFSKRGEHISPEIWKEML

ESDEEFCLIDVRNDYESKIGHFEGAELPPCKDFREFEKYADELDRRHGSEKPPVMMYCTG

GIRCELYSSILKEKGYDKVYQLEGGVINYGHKVGNKHWQGKLFVFDDRLTVPVGDEETPP

ISHCQFCSKLSDHYYNCANMDCNTLFISCSECIKTYMGCCQKSCQNADHVRPYHQQNPHK

PFRRKHFYEKVSCSCSKAQSGDIPSDH

>tr|D6YST6|D6YST6_WADCW Uncharacterized protein OS=Waddlia chondrophila (strain ATCC VR-1470 / WSU 86-1044) OX=716544 GN=wcw_1792 PE=4 SV=1

MIIASCIYSKDWNNLFPAFETPREKYHLALNETSSLTDLVFSSIREKKNPSSEELQILRS

PTRLITESKEKIEDHIDKRIALFFQQLIVTYRVPFHFEIGKTQHQAPSTPTLSKVPFNSA

HSATIPCLYAYHLDDWKKWEADPQNTPKPAPFVYLKGSDTYNHHNATIGLIKLVNTADII

LDGTSQSADKLRIKSIGIIERSARGEIDPREGLKKFLTQAKKILIRQIISPDEKEEYRQI

LNIYLSRVENAIEQSKDESFFDQLINVKIDHESREASALRTLIYRNRFHIIRESQEMQSV

IAKKIHDVAASIIGIGRKPANFDFVMKKKILSLSSSQGKKQLSRYFGVSLEQITHDLQGK

RFAAAERKINEEEHKQKFDTLMRDIRTLCRDLVRREMAYRAQVTKDLRNHKRWRQKDLVA

EYRKVYADGPMSQPTVSRLENTIKPIERQLAHRLASIFNVDPGFFFPAIFTSVE

>tr|D6YSB7|D6YSB7_WADCW Uncharacterized protein OS=Waddlia chondrophila (strain ATCC VR-1470 / WSU 86-1044) OX=716544 GN=wcw_1617 PE=4 SV=1

MTIPEYPLEKVMDIKKKRVDEAEKVVAAKKRELEAEREKLRKCEEERDKAVQHKIDKLNQ

LRDELDRGTTTDKIQQMKNYLKVVEERILVEEKKVQEQEEQVNIAKRNLEIAQEELKMRR

QEVDKLQNHKKDWIKQMKRELELAEEREMDEIGQVLYLRNMRDAP

>tr|D6YT03|D6YT03_WADCW Thiolredoxin peroxidase OS=Waddlia chondrophila (strain ATCC VR-1470 / WSU 86-1044) OX=716544 GN=ahpC PE=3 SV=1

MNTLIGKKAPAINASAAAGKKTINNFSLEQYKGKYVVLFFYPLDFTFVCPTELHAFQSKL

EEFQKRGCEVIGCSIDSVFSHHAWLKTPVSKGGIEGVSYPIISDIHKTIAKDYGVLCEEE

GVAYRGLFLIDKEGCIRHQLVNDLPLGRSVDEALRMLDALIFFENHGEVCPANWNEGSKA

MKPTQEGLEKFFAAEVN

>tr|D6YVR8|D6YVR8_WADCW Uncharacterized protein OS=Waddlia chondrophila (strain ATCC VR-1470 / WSU 86-1044) OX=716544 GN=wcw_0863 PE=4 SV=1

MIMNKEPDFPLISTELDLTSENHNHLYYSSLVPRTLKQILPSEFIQFYGSHPDEKEKLFK

HFQSLLPFTYWKPTNSTPTNMSCYLICCYRPNAFKFFFEMISRWLIPGKRLNVLAMYASD

FTIPEFSEQVYTMCEMVIRIENAEEMEAVKRNLPIIESEVRLGVVSSYYARRILEVKGLS

NDEKTAMIQECIAWATARWPHNFSFDVFTEMQHVLVMCRDDFKLRRSVRHLSRLIGIQYL

FRKDLRQKIRKHPGKRHLNLKFFQTTLLLSDSEKKVLGVLLAVNFLRDKEVFEETHLIKA

IQNYIPEAVMIENSSFFNRRGAEPICTLYLEVEKQDGKLFTASEVTTLRNNLPKELGERV

EHLIQPVFMPRNEEEIMRNILTLGNQIKYLRDIPQVFITFDEQTHHHLCFNVIIVRVLKE

GDSLIEESFKNSSSRLEYIHDRTKTIGFLRKKYPKEATVCRLRLPKEEFLRRDHSIDLYK

AREAVFAEVSNALGEVRDYNGGMISKQNELLASVRKLLQGIVNYNDLLLEDFFYSLNPVI

MRTVLEPEALKTLFVMLLESISDGFFNEESYAMKVRAEPHFVYVMITAEHVELEELISEE

LALLDFSPSSLAMTQVNVYDILYLGYIFRSDDPQKQRKFCISIQHAIEKLEHQR

>tr|D6YUK7|D6YUK7_WADCW Putative Acyl-CoA dehydrogenase family protein OS=Waddlia chondrophila (strain ATCC VR-1470 / WSU 86-1044) OX=716544 GN=wcw_0446 PE=3 SV=1

MDEEEKKLAEELLFSEKKNPSFAKQLFLGKFDHSLVFPYPYPEKSEEEETARFVGKVSEF

ASVHLNAAEIDRQAQIPDEVLKGLADMGVLGMTVPKEYAGLGMSQRAYCKVTDRIARQCG

STALFINVHQSIGLKALLLFGTEEQKEKWLPDLAKGKKYAAFSLTEPNAGSDANGVETKA

VYDPEKKVYRINGLKQWTTNGSIADILTVMAKTEVETKEGVQERVTAFLVTPDMPGFIVR

DKALDKLGTRGAWTANLAFNHLEVPEENILGPLGGGLKVCLTLLDYGRTTFGAMCTGAAR

ELVQLGVKHARERYQFKRPLASFSLVKKKIAKICSLLYAMDASTYLTAGLIDSGVEDIML

ESAILKVFTSDALWEIIYEVMQILGGRSFFTDQPYERMMRDARLNMIGEGSNEVLRAFIG

AVGMRGLGVELQKGVFSNAGKLFSRLFLPAIPVRSQRLSKEAKELAAVLRKLHWYSAGLL

AKHGEDIVECQLQLNRIASIAMSVYTMSAVLSKLDTDKPSGRDLSVGIYFIRSQLALCRR

WFKGIGSNDDEASVKLADAITGI

>tr|D6YTM8|D6YTM8_WADCW Putative tyrosine-protein phosphatase OS=Waddlia chondrophila (strain ATCC VR-1470 / WSU 86-1044) OX=716544 GN=wcw_0114 PE=4 SV=1

MSINLLGETSCQSTVEDQLKILGSINDKSVQQFVRETLDSWNGRQISKGELNSTIDKITH

LVSKVLNSKKSISENGEIRLALEKLKKTKGETEIICTTFSSSTSKTVETILNELDEDHLD

SHPAYRMGWTQMTASNALKLPSIPIGSYVIVKEKKKLTMFIKGDSGPEQHSLEKKEGEAG

YTVFNPLSFFKSFEDSCSGEEDVTCFETNFSKKISIDKDEDTAPPAVLSMTVTPLRELKP

ETEALLKLCSQLSEVKFNFFFHWRSLKEKSDFIKAPNNRPQHKSSENHKKSSVDEINLAE

RTAKLLIRRDQPVFFHCNTIKVGKHQFDCHQLPTDYSRDDVWLSLWNDAGEADGRATIMK

LSTNHCPLSNDLPVIRDYWPVRKGDPSIKKFQNRCLLAEKTLKRIEKKDKIEELIAKISR

MEKEKAKTEIELNHLENELTEIDAAPQINKLKQNIKKYNEEIKKNKQELDILLIAAKEHL

AYVLKEKEAEESGTGPGVYIGSLTTIDEITVEHIDETLIEDFPGAILRQFKLTQPGETPP

RFVTQIDYPHWSDFSATNASVLTSLRDLYREVHGDNPAPMKVHCRAGVGRTGTFIVFCGA

MDMIEDAVETGKISGLDAKEMLFNIIADVRSQRDFQMVQTPQQGMQVISALELEILKIAG

KIQAD

>tr|D6YVV8|D6YVV8_WADCW Peptidase_M78 domain-containing protein OS=Waddlia chondrophila (strain ATCC VR-1470 / WSU 86-1044) OX=716544 GN=wcw_0907 PE=4 SV=1

MIAPHMRKTSADLPGAEATAKQVIIANRVSAPPVPVASFFTNDGFSVYAAEFPDQIIIAG

FIDLDKKQIILNESDDLEVQQFTLARALGHWIMHGDELDEIPELKVIYHQSLGGDLKNFY

EKEALHFALHLLLPEIFIVDDLRLADQAIAVKYQVPDFVVRLIRKEYLS

>tr|D6YW79|D6YW79_WADCW Putative membrane protein OS=Waddlia chondrophila (strain ATCC VR-1470 / WSU 86-1044) OX=716544 GN=wcw_1031 PE=4 SV=1

MAVSLDLDCGDVFNQFSKEGKFADVSKNFFESRSNYYKLTKHTVEYAFAIAEVTSIVPLQ

FIPIFEAIRSIVTVAFKALSIYHNVEKIKKSHQELALCKVKVEKWSQEHRDDAPFDRHSL

FQLRDRYLGKIQLLQEKPEKEAFDRFRKNRWQEYLKLIEDAIEGTDAKAMQVLQSKFAMR

KERKAASWQKKEHDLHLKIKKSYLEIAISIGGIALEAMTIISIAFIPLSAGSTFIAVKLG

SNILVGAMEYGKYLWWSTHKKENPGVIAIKSIEHIGTSVAKTKLLPYSSVMHPSLIKGAC

KTAFSLIRVFHSRYNYMDNHYAIGECKRNIEHLKKKNIVLMQASHKIASGDRKEMFRILN

KYETKKNGALSIIQDYEEALEREETTRNPDELIAMIGIMKADIKKWERYIEALRRALDTN

DLSDAKIAANCLCERNGRRISNWKLSEQLHQKNNLKEGMKTALSIVEIARFAFLITAISL

ALVGMGINLLPYLFASSLFANVLYMAYAWFTMKTNKEIDGIIDLWSRDEIAPSTF

>tr|D6YUW6|D6YUW6_WADCW Putative transcriptional regulator OS=Waddlia chondrophila (strain ATCC VR-1470 / WSU 86-1044) OX=716544 GN=wcw_0557 PE=3 SV=1

MSRLHQIETLASVGRHGSLAGAARELGVSSAAVSKQISKLEKELGIQLLVRTTRKLAFTD

IGAAYLEQVHRIIEELEASKALVSQMKAIPCGKLRVFCTPHFASRIVVPNIAEFLNLYPK

IELELEIGERIPHFEGEKIDVMMGNSMQAGSDLVHRRLLTTRYAICAAPSYLKRYGEPKH

FNDLKDHRILTHRMRCPDNLLYFKDKKKVAFAPYIKVNDAKILVDLAFQGIGIVQLHHYT

VKKYLQSGELQELFQNLSQDDVPIYVVLPPRKYTPRKVRALIDFIDEKLHSSDLSIRSCQ

RS

>tr|D6YSW4|D6YSW4_WADCW Uncharacterized protein OS=Waddlia chondrophila (strain ATCC VR-1470 / WSU 86-1044) OX=716544 GN=wcw_1820 PE=4 SV=1

MVLGELLEHINNPVLFLEELKNKYQAKVSKVIITVPNAFRISNFKFSLFNKELINSDHRY

WFTPYTLAKIIVESGLIMEDMHFVEGCLSRKSFFKNFLLKRKPLFCDTIVAIASL

>tr|D6YRW4|D6YRW4_WADCW Uncharacterized protein OS=Waddlia chondrophila (strain ATCC VR-1470 / WSU 86-1044) OX=716544 GN=wcw_1460 PE=4 SV=1

MLAKSKSKNSHPEAQNPLILRAHRLMEAFAKSDDERDFYLDKIEGFIIYVDLDKTQEELD

NLESELEKDPERYSPIPKLSFFESKKIMESFVNEKVYDIDTKEKLLDIIQSKEARDNFLE

FIYDHHMELEKWQQFYQERSRIRIIEWLRSNSFNFVFEEDLDLSTKAVEKLKEHLFDKKV

PKDVEVARKTLMTKAKSYYSNEALNPRPKRGRPPKQQEKVEMEPQVTVDIYTTVPSAVRA

FLFTPDITSIADVTFSSRFETGKELLDHLKNGTSPYEMEESMNNINAKLAQLRELSSNWM

NDDNEAPTSIKEKDSFVGDSYEGLGDEDKELFLKLEEKKNSIQKKKEEKKPVKKKIQKVP

EKKPVKRIIPKEPAAKKTKPTKRPIRKLIRTKPAESQSTKKKK

>tr|D6YVK1|D6YVK1_WADCW Uncharacterized protein OS=Waddlia chondrophila (strain ATCC VR-1470 / WSU 86-1044) OX=716544 GN=wcw_0795 PE=4 SV=1

MKALDCLFRFFIRFRHPFTLPEDIANSLGVNVSKFVTFDEFVERITAPECCPTRLHKLMT

RREAENAFCGAQRKEKFQRNTLVSYYFNEGWLEFNLKFDEDARLRRIYIQHKKIESDQGV

ELHLATD

>tr|D6YSU3|D6YSU3_WADCW Glycosyl transferase, group 2 family protein OS=Waddlia chondrophila (strain ATCC VR-1470 / WSU 86-1044) OX=716544 GN=wcw_1799 PE=4 SV=1

MRRFIPPLLLVFLVSFAGVFAAPTVCLNMIVKNETDVIERCLQSVLPIIDYWVIVDTGST

DGTQQMIQAFMDKHGVKGELHERPWKNFSHNRNEALQLAKGKSDYIFFIDADEYLMYEPN

FKLPHLDKDFYYITVLHSGSSYPKLQFAKSSLNWKWEGVLHEYLGCPGSKTFATLEGVSN

VYTTEGARSKDPLKYKKDAAVLEDALKDDPSNCRYVFYLAQSYRDAREYDNALKSYHKRV

SMGGWDQEVFYSMLQIASIKEILNYPKEDVLESYFQAFSNRPSRAEPLYRIANYYRRLGD

FKRGYQIASIAAGIEKPNDILFVEDWIYNYGADLELSVCAYWIDEFEKSRDVSLKLLDRE

DLPGNVRDCVKKNLEFANVKLAELSMR

>tr|D6YVC3|D6YVC3_WADCW Putative type III secretion chaperone SycD/LcrH OS=Waddlia chondrophila (strain ATCC VR-1470 / WSU 86-1044) OX=716544 GN=sycD1 PE=4 SV=1

MMDKMIFEIQDQLIKPLMEEIERMYLEHLQSQQISKGQLGASIPALKKNVEKEMQEFKNL

LLGREELSLMEKGFDLALQHLHKLPNATVIIEDLQQAGHRFISQEKPSNDEIPFFYDTLL

EMFGLSEETFNSFYTIAADFFHQKQYSNALSIFLLLTNLSHLTFEPWYAQGICWQKKGNF

SEAMRSFAMASLVNYDHPGPHLHTAEIYLSIGEHKLAAETLDHALKECDQELLKSQKEFI

RFLKKRQK

>tr|D6YSY4|D6YSY4_WADCW Uncharacterized protein OS=Waddlia chondrophila (strain ATCC VR-1470 / WSU 86-1044) OX=716544 GN=wcw_1840 PE=4 SV=1

MTACNLQEIYSACQSKKSGEPALVPSLISQRVYHSSYGWGRLWKWFYLAVQFLTGKDLKT

KRLIKIMQKMEKIFSKKLPQVIENAAAYQDYLEKRIREEEVDENEVHALRKNVRRWTRAT

APLSSIAGKKQNEKITSLFQTYYPDSIERGELPFSYGQGEVLLRETQLLIDLEGYLHSPL

PLALFKKLARKEDLSSNEQHELEKWIKILNKKKENIPVDLFIDCLRVLTNKPSFGGSLIE

LKVRLLQNNLELLRMKEEKHLSWRAALQPGDELKSGSHTYRLGEAIGVKSEGFDSTLIFE

IEGNEDHVIAVGMNRAYWSIKQKVANEFQWGIKMPEIKEISPDGRFAIIERLTPAISENQ

WESPENQPLVESDLSILDPISNLFKWWGKESVCPANFSLNRLMFNMDGELKYTHSLQPTA

FDFRLLEDLAYEVAQGHLNVYLHIMQQSKLSSHLTMNFYRRVVEASLKNESVKIRDLAAY

RKISDPLVIQRGRKLYKKIQKLRAKIIKTLNKEFDHIDQHSLLANTNKELKEWYEGTCSA

SRLWPSIEEAVTGNLRRPLQLGRNL

>tr|D6YVC6|D6YVC6_WADCW Putative membrane protein OS=Waddlia chondrophila (strain ATCC VR-1470 / WSU 86-1044) OX=716544 GN=wcw_0720 PE=4 SV=1

MRNSDSLGKITSYLPGVIAIFLWSTGAYFIFKMKNLPVSQILVVGQLVGGLISIYFEKSR

FSLHSMKNRIRKGWAILAIFWISQYGYIYAFQNAPPAQINLIFYTWPAILIIIKAWNITR

KLNKWDIAGIWLGFMGIAILLSPDLKQDAFSLRYLSGYLGSILGALGWVAYLLYTDQEKK

DKKGFTSIGEDILILGIFNFFHLMITEHWIQPNTNEWMMLGYYSVSMFGIPYYLWRITLK

KDPKMAGALSNATPVLSIIWLILAKITPFTIELAVSIVMVNLGIYCIDRSAKSTSSITG

>tr|D6YT81|D6YT81_WADCW 30S ribosomal protein S20 OS=Waddlia chondrophila (strain ATCC VR-1470 / WSU 86-1044) OX=716544 GN=rpsT PE=3 SV=1

MAEKEAKKKEKRSTAFKRDLQNRKKRADNRVFKSRVRTAIRRFEEVVAKNEQESVAETLS

VVYALMDKGVKKGLYKLNMASRTKSRLAAKAARAK

>tr|D6YRR5|D6YRR5_WADCW Uncharacterized protein OS=Waddlia chondrophila (strain ATCC VR-1470 / WSU 86-1044) OX=716544 GN=wcw_1410 PE=4 SV=1

MVQFNNIQKALHHQIGQSGITVGDCLHVIGSNFLGRTITQLSLLFQGKGWVNEKQALQKI

QSLDSISRQSIHSISGQIVLSLEDGYLKNRLNDDNPLRGDQKKISRKEEKQLLKDLELPT

DQTIREIIKEMKLLSELTELKYEGW

>tr|D6YWQ6|D6YWQ6_WADCW Uncharacterized protein OS=Waddlia chondrophila (strain ATCC VR-1470 / WSU 86-1044) OX=716544 GN=wcw_1210 PE=4 SV=1

MTLIELIGFMITMVAFFLLIAKQARDERRRRQNPEAYEEEAEQEHQETIKHFLRSLNIEI

PEDMPPPKRELPPVPKVLEPEEIEAPALKQKSLFRDPYALHKPFEYGEKRKKQQSRVSQM

LKGKDSLKQAVILKEVLGAPKAFE

>tr|D6YVZ2|D6YVZ2_WADCW Uncharacterized protein OS=Waddlia chondrophila (strain ATCC VR-1470 / WSU 86-1044) OX=716544 GN=wcw_0942 PE=4 SV=1

MKLFFLPFLITVFFLFSCQNKEEFNYAQYTYKEIDKFKKYAFKKYGVVLWGKGGGFMDQI

NEISLTFYIQKELKVKEMRELIINLSCDFLKQINSNNEIRQYLAEHPFTSKRLDLAVAIF

NSKKTYITNPGSTKEKLALGVLNKDNICYTIKNEEEQFLQKVYKETFQEAIEKVQISQIQ

LNK
[truncated: 563,434 more chars]
