# Supplementary material for: Pharmacophore modelling based virtual screening and molecular dynamics identified the novel inhibitors and drug targets against Waddlia chondrophila
Source: Sci Rep. 2024 Jun 12;14:13472. doi: 10.1038/s41598-024-63555-1 (PMC11169463; doi:10.1038/s41598-024-63555-1)
Supplement: Supplementary file 2 — Supplementary Information 2. [file 41598_2024_63555_MOESM2_ESM.docx]

>tr|D6YTC9|D6YTC9_WADCW UDP-GlcNAc pyrophosphorylase OS=Waddlia chondrophila (strain ATCC VR-1470 / WSU 86-1044) OX=716544 GN=glmU1 PE=4 SV=1

MEPFSPLELFDLSSYPHKALLENCTYAWEALLFLKKYLTQQNLGNLQGTISSNAYLVNKN

QIFIGKGTVIEPGAYIEGPCWIGENCTIRHGAYIRGNVITGHGCILGHDSEFKHSILLNG

SQAAHFAYVGDSILGNHINLGAGTICANLKLDKQPVEVKFESHRIHTGLRKLGAILGDNA

QTGCHAVLNPGTVFGKAALCYPCLNVSGYLSPGTKMKMEK

>tr|D6YWF5|D6YWF5_WADCW Putative pre-16S rRNA nuclease OS=Waddlia chondrophila (strain ATCC VR-1470 / WSU 86-1044) OX=716544 GN=wcw_1109 PE=3 SV=1

MKTRIIGIDYGMKRIGLAISDETHLIASAIETFEASKKMEETVAALVKRLEEHQKEKGYE

LKEIVVGFPLKMNGKAGFISDEVTLFAETLSKALSIPIIKWDERLTSVQAERAMMEGSYN

RKKRTKKLDQISAVIILQNYLDSKNLII

>tr|D6YRW3|D6YRW3_WADCW Diaminopimelate epimerase OS=Waddlia chondrophila (strain ATCC VR-1470 / WSU 86-1044) OX=716544 GN=dapF PE=3 SV=1

MQSSTPTKAFELRTSIPFSKYTGCGNDFILIDNRNQVYPSEESDLAEHLCDRQFGVGADG

VILLERSDRADFVMRIFNPDGTEAEMCGNGIRCLGKFLQELGIEGTRFTIEVMGKKYPLS

LHDDGSVSVSMQPAPEISWDIELSIGRKTFHVDFLDTGVPHATLSVEKLSAFDLDAIGPK

IRYHEKFMPQGTNVDVYHIDESDPSLIYIRTYERGVERETMACGTGATACAIIAWTKEQI

ANPIRIQVASGDILEFKISGSLGRIDQIVMKGPAEFIFRGNFSI

>tr|D6YRN8|D6YRN8_WADCW Single-stranded DNA-binding protein OS=Waddlia chondrophila (strain ATCC VR-1470 / WSU 86-1044) OX=716544 GN=ssb PE=3 SV=1

MVALNKVMIAGRLTRKPELRKTPNGASVTDLLIALNREFTTSAGEKQQEVCFVDVVVWGR

LAENCTNHLDISSPVLVEGRLQLDVWEGKEGDKRCKLRVAAERVQFLEKLDRKNSQEEND

ALAESYVLN

>tr|D6YSK9|D6YSK9_WADCW Putative chromosome-partitioning protein parB OS=Waddlia chondrophila (strain ATCC VR-1470 / WSU 86-1044) OX=716544 GN=wcw_1710 PE=3 SV=1

MSQEVVEVELSQIRENPYQPRKQFNREELEELAQSIQSVGVLQPPVVRKMENGGYELIAG

ERRFRAAEIAGLTTIHVLVSQKPGNVSAEAALIENIQRVDLNPLDIAQALRRLIVEFGLQ

QDELADKVGKKRSTVTNYLRLLSLPHKIQESLQFGEISMGHAKAILSVSGFEEQLYLHRM

VVEDGLSVRETEEAAAKLNVKPVKKKMRTLNENTVYLSDLEEKLQQRLGTKVTIASSGKR

GKILIDYYSLDDLERILEVISL

>tr|D6YRZ2|D6YRZ2_WADCW 50S ribosomal protein L6 OS=Waddlia chondrophila (strain ATCC VR-1470 / WSU 86-1044) OX=716544 GN=rplF PE=3 SV=1

MTRKAKVPVDIPNGVEVKVDQEKISVKGPKGQLEQKIMKGINVIVEEGKVHVQLDQNAKD

MKNFQGLYHALIKNMVHGASQGFEKHLEMIGVGYRAAVQGNLLNLQVGLSHPTNLTIPQG

LEIKVEKNTKIIISGPDKQQIGEFASTVRSIRPPEPYQGKGIRYSGEYVRRKAGKSAAKK

>tr|D6YRU2|D6YRU2_WADCW N(6)-L-threonylcarbamoyladenine synthase OS=Waddlia chondrophila (strain ATCC VR-1470 / WSU 86-1044) OX=716544 GN=wcw_1438 PE=4 SV=1

MLTLVVDVCSDRGIAAFIRNGKVGYFAGLPLGLHNSKFLMPKIEEGLKVLEIQSSQIELI

AVGIGPGSYTGMRVGSMVAKALSFAHGIPLIGVSSLEGYLSDHQGIFAAMIDAKMAGCYL

MKGRKKGNEVNYLTGPEVWKMEHIGSVLKDVDVIVSPHCRSLQEKFQKNDLNRQWEWQET

AINVEHFASRAEKKYRDGEFSIDGSLDLLYMRG

>tr|D6YW37|D6YW37_WADCW Thioredoxin reductase OS=Waddlia chondrophila (strain ATCC VR-1470 / WSU 86-1044) OX=716544 GN=trxB PE=3 SV=1

MEIKMKKTKVAIIGSGPAGYTAAIYASRANLEPVLYEGFQTGPAGGQLMITTDVENFPGF

PEGVMGPELMMTMRKQAERFGTEVITDDVVSVDFSEYPYKIKGNKHEHEAFAVIIATGAS

AKRLNIPGTREGEFWQKGVTACAVCDGAMPIFRNQKLYVIGGGDSACEEAIFLTKFGSEV

FIVHRRDELRASKIMQERAMNHPKITILWDSVLTKVEGDHVVKKVTIQNVKTKEENVHDA

GGVFFAIGHLPNTSFLNEQIELHDNGYIKVSPGTTRTSKAHVYACGDVQDFTYRQAITAA

GTGCMAALEAERELAEKGHLD

>tr|D6YVR4|D6YVR4_WADCW Transcription termination/antitermination protein NusA OS=Waddlia chondrophila (strain ATCC VR-1470 / WSU 86-1044) OX=716544 GN=nusA PE=3 SV=1

MNKDLVAIFEYLEKEKGIQREIVIHAIEESLQAAARKSVSGASNVTVQIDPKTGNINVFS

EKEIVDEVEVPAQEILLEEAREIDPDCEIGQFIDILATPKDFGRIAAQKARQIISQKLRS

AERDVIYEEYRHRVNEVVSGTVKRFVKGANLVVDLGKVEALMPMRQYPKTEKYKIGDRVL

ALLMEVNDTDSGGAEVILSRSSPEFVRQLMIQEVPEIVEGIVVIDKIEREAGYRTKLTVR

ATDQKVDPVGACVGMRGNRVKNVVRELNSEKIDIIPYSDDPIELLQNALSPIEIRKISIS

EDDRVISIVIDDDDYAAVIGKKGMNARLNSRLIGYELEVQRMTDYNKTMAIQRTELAESD

DPTLDHPLTGIEGVNKLVFEHLVAEGFNTPRTLLLASPEQLSNAASISLEMADHILEQVR

KQRL

>tr|D6YSE6|D6YSE6_WADCW Dephospho-CoA kinase OS=Waddlia chondrophila (strain ATCC VR-1470 / WSU 86-1044) OX=716544 GN=coaE PE=3 SV=1

MLRLKKVAVTGGISSGKSLICQYFSEFGAYVIDADKIVHQLLNPDTEIGQKVVALLGERI

LDKQTISRSRVAKLVFLNPRLLKSLENLLHPLVYEEINRIYKKVAHEKNPPPLFVAEVPL

LFESGGEAYFDQTIAVVSIQEKCWERYRASTGNEREDFNRRTACQLPQHVKAEKADIVIH

NEGSIESLKKQTKTIYESLRQCT

>tr|D6YRN2|D6YRN2_WADCW Probable nicotinate-nucleotide adenylyltransferase OS=Waddlia chondrophila (strain ATCC VR-1470 / WSU 86-1044) OX=716544 GN=nadD PE=3 SV=1

MKQIGFFGGSFDPIHFGHLKMAKELKEKKMLDEIWFSPARISPFKLDRCPESVENRLEML

RLALGGEPGFKIYEEESRRLGPSYSIETVEHLSEIPDCQFYFIISDESVPEFFHWKEAER

IVQLVPLIVGSRVGAEPPKKGNETICQAMERGWTPTQILDISSTQIRKFLKEGKDCTSYI

PRNVLDFIYQNHLYSSTNYE

>tr|D6YVL0|D6YVL0_WADCW 50S ribosomal protein L36 OS=Waddlia chondrophila (strain ATCC VR-1470 / WSU 86-1044) OX=716544 GN=rpmJ PE=3 SV=1

MKVKASIKADPSKGDKLVRRKGRLYVINKKDPNRKQRQKGPARKK

>tr|D6YTJ6|D6YTJ6_WADCW Transcription elongation factor greA OS=Waddlia chondrophila (strain ATCC VR-1470 / WSU 86-1044) OX=716544 GN=greA PE=4 SV=1

MSYLEEFQNQINNRDFHKFFQLWEEYCTNDEVDSDEFISLLDIIKNSDFNTLFGQFAETA

LPLWQCIKDDEESYQVLKRILDLQTSNSSLLASTAISMLKKRYGEDPKFKERLRQIGLRT

QANFQGAISNFELLNHMAKGKFVFHTSGWGTGEIMDISHIREQVAVEFENVTGIKHLSFE

NAFKTLIPLLDEHFLARRFADPDLLEKQAKKDPLEVLKALLHDLGPKTAGEIKDELCILV

IPEQEWSRWWQNARARLKKDTMVETPNSLKDPFILRKKELSHEEEMHREIEKQTSVDDIV

QTTYTYVRDLPHVLRRREVKDTLRDKLVSLLDEEQLSSAQELQICIFLETLFGHSIEGKS

VKDFIHSMENVEEVLNSMEIIAFKKRALTMIRENREDWAETFSSLLFSIHQNPLRDYLFQ

ELQSSESRELLMKKLNTLLRYPERHPEIFVWYFQKICKKNPGNIPFSNKEGQCQFFEAFL

ILLHRIEHENEWKDLVKKMYNTLTSKRFEVVRNLIENTTLEFIKEFLLLVAKVHVFSDHD

KKIMRSLAQVVHPSLAPSKSKSITDDASTLWTTEEGYRKTQERVKRIATVEMVENAKEVE

EARSHGDLRENSEYKFACEKRSRLQSEMKMLSKQLSAARVLTPEDVDSSIAGVGCIVEVE

EPSGAKEQFTILGPWEADIDNGIISYQSQIAQAMSGHKIGDSFTFKDGHYKITALKSVFE

G

>tr|D6YWX4|D6YWX4_WADCW Pyruvate kinase OS=Waddlia chondrophila (strain ATCC VR-1470 / WSU 86-1044) OX=716544 GN=pyk PE=3 SV=1

MLSAILSIVKRDRLMACRTKIICTIGPAVNTYDKICALIKAGMNVARLNFSHGSYEEHFS

VIEMLKRARKELQVPLAILLDTSGPEVRVGKIQDGEIKVNKGERLRLLDKEVLGGNGVIS

VNPPGILKGLSVGAQVLFDDGYISSRVKEVAEEWVELEIENYGVLKGGKGVNIPNVSLNL

PSVTEKDVKDIEFGCRNGIDWIAVSFVRTPENIITVKNLLESNRCSHVLVIAKIENHEGI

EHFDSILQISDGVMIARGDLGVEIPLSHVPRLQKEMIRKCYLAGKPSVTATQMLESMINN

PRPTRAEVSDVANAIYDSTSAVMLSGETAIGKYPIEAVEMMRDIISEAETDFDYRSLFEL

HSSISYNDVPSSVTLATVKTAYSSGAKAIFAFTSGGGTARLLSRLRPELPIVAMTPKENF

YHQLSLNWGVIPFLGKESKTFEEGFEQVSQFALKNHILSYGDLAIATAGSTFGIKGTTNM

MIVEHIGDVLVRGHLGEGEKVYGNVKFLRSPSEGLQPYHVRGALIVITKCDESYLPYIQE

SAGVILQNHIDDEASEMFAIEQAGLFHKPTIVRADAAAYILKEGQLVTLDPGKALIYKGV

VI

>tr|D6YVW5|D6YVW5_WADCW 50S ribosomal protein L35 OS=Waddlia chondrophila (strain ATCC VR-1470 / WSU 86-1044) OX=716544 GN=rpmI PE=3 SV=1

MPKLKTKKAVAARFKLTGKGKLLRQRPGLRHIMTKKTPKRKRQLAKPALVSDSQLKTYKR

LMCVS

>tr|D6YVL1|D6YVL1_WADCW 50S ribosomal protein L34 OS=Waddlia chondrophila (strain ATCC VR-1470 / WSU 86-1044) OX=716544 GN=rpmH PE=3 SV=1

MVKRTYQPSKRRRKSEHGFRKRMETASGRKIINRRRRAGRKALTRV

>tr|D6YWN2|D6YWN2_WADCW Acyl carrier protein OS=Waddlia chondrophila (strain ATCC VR-1470 / WSU 86-1044) OX=716544 GN=acpP PE=3 SV=1

MATEQEVIDIVVEQLGVDKGDVSLEKSFVEDLNADSLDLTELIMTFEERFGIEISEEEAE

KLKTVGDVVNYLEKTKS

>tr|D6YWM7|D6YWM7_WADCW SsrA-binding protein OS=Waddlia chondrophila (strain ATCC VR-1470 / WSU 86-1044) OX=716544 GN=smpB PE=3 SV=1

MMFAMGKKSSSSELVSNRKARHSYEILDTYEAGIALTGTEVKSLRDNGGSLQEAYIRVKG

GELWLLKCHIAPWKYGNIHNHEETRERKLLMHKKEIQRLKAATQEKGLTIVPLALYLKNG

KIKVSLAAAKGKTGLDKRKSLKEKDEKRRMQQAMKRDVSH

>tr|D6YWN1|D6YWN1_WADCW Holo-[acyl-carrier-protein] synthase OS=Waddlia chondrophila (strain ATCC VR-1470 / WSU 86-1044) OX=716544 GN=acpS PE=3 SV=1

MIKGLGTDIIEIDRIEKVFNRYGQKFLDRILSKSEQEYCLKYKNPVQHYAGRFAAKEAIV

KALGTGIRKAVSWTDIEILNNNQGKPQVYLSPEVRSHFSDPIIHISISHSKKYATAVAIM

EK

>tr|D6YWH1|D6YWH1_WADCW ATP synthase subunit delta OS=Waddlia chondrophila (strain ATCC VR-1470 / WSU 86-1044) OX=716544 GN=atpH PE=3 SV=1

MTKASTKYAGILFFLALKKQELETYLFQLQEIKKITSNTHLQEAFASPLVPLQAKKNVLE

LLFKDKVKEEILLFLKILTEQKKISLITEVIEEFSIKMKKQMGILSIKLISAEKIDEENK

LLLQNKLEAKYNKKIEFYEEQSSKIIGGMILLFPNGKILDKSLKTCLEHLRKHLKKGKRH

AA

>tr|D6YRY4|D6YRY4_WADCW 50S ribosomal protein L17 OS=Waddlia chondrophila (strain ATCC VR-1470 / WSU 86-1044) OX=716544 GN=rplQ PE=3 SV=1

MRHLKDRRKLNRTSSHRRCLMANMLKSLIVNERIETTVPKAKCLRRYADRMITLAKKNSL

ASRRRAISQMMIRFNSLTPKQARAAKNGDTSSYNDDRLVIEKLFSELGPRFTSRQGGYTR

VLKSDRRVGDNAQKCIIEFLES

>tr|D6YV44|D6YV44_WADCW Putative dihydroneopterin aldolase OS=Waddlia chondrophila (strain ATCC VR-1470 / WSU 86-1044) OX=716544 GN=folB PE=4 SV=1

MQGTIGFNHLRINCIIGDLPEEREKVQEIEVSVKVACDFYACSLSDDLADTVDYVSLAAA

CRREAEEGRYHMLETYASRTLDKLLEEFPIDYAWIQVKKASGLPDADCSFVELSKKKKYP

G

>tr|D6YVM0|D6YVM0_WADCW 50S ribosomal protein L25 OS=Waddlia chondrophila (strain ATCC VR-1470 / WSU 86-1044) OX=716544 GN=rplY PE=3 SV=1

MKLKFEQRTAEKKSDSRSLRNLGKIPAVLYVRGKDSEAIAVDAADFETVLRTVKKGRLST

TKLALVDGTGKERNVLVKDIQYHVTTYNVLHLDFEELLDNVKIKVKVPIECVGEVDCVGI

KLGGVLRRVIRYLRVHCLPKDLPEFFKMDVKTLGLKESRKLSDLEIPETVRPLMDLNEVA

VTIAKR

>tr|D6YVD7|D6YVD7_WADCW Riboflavin synthase, alpha subunit OS=Waddlia chondrophila (strain ATCC VR-1470 / WSU 86-1044) OX=716544 GN=ribC PE=4 SV=1

MFTGIVKGTFPIVDIIRERGFSCAVLLPSELREKLAIGASVAVDGVCLTVVEILGDRVYF

DLIEETLKVTTFNAMEIGRLVNVERSLKFGDEIGGHLLSGHVMATAEIFEKQTRNEETIV

KFLVPERIEKYFFKKGFVAIDGISLTIVEINPLSVHLIPETLRMTTLGLKQPGDRVNIEV

DYQTQVLVDRHSM

>tr|D6YRZ4|D6YRZ4_WADCW 50S ribosomal protein L5 OS=Waddlia chondrophila (strain ATCC VR-1470 / WSU 86-1044) OX=716544 GN=rplE PE=3 SV=1

MSRLKKYYREKVKEELQKKFDCSNPMTIPTLRKVVINMGIAEAAKDKNAIQDCINELSLI

SGQKPILTKAKKSISNFKLREGMPIGLKVTLRGDRMFDFMDRFFNIVCPRIRDFRGFNPK

GDGSGNYTLGLDDQQIFPELNLDEVKRTQGMHITFVTTAKSDEQCIELLRLLGLPFKKLP

VSVTI

>tr|D6YSR9|D6YSR9_WADCW Replicative DNA helicase OS=Waddlia chondrophila (strain ATCC VR-1470 / WSU 86-1044) OX=716544 GN=dnaB PE=3 SV=1

MAPQNLKVKIPPNSKEAEMMVLGCMLTSINALNIAADKLHDFDFYFTEHKLIFNSLKSAY

KSDKPADIHIICEDLKRQGQLEAAGGAAYLTTLAQFAGTSAYIEEYCRIVHSKSVLRRII

NTSHIIEKNALEEPDDVENVLDEAQKLLFEIGKSANSGDAVLLSEIITGTKSESGVHYLK

ELQERQEKYQERGDEDPGITGIPTHLADLDKMINGFNNSNLMILAARPAMGKTALAINIA

ENICFKNKIPVGIFSLEMSAEQLVHRLICSQAEVESDKIKTGSLDGHDYQRIVETINHIQ

DHPLLIDDQPGLSINDLRARARRMKETHNIGFLVIDYLQLLSGSGNNKNGDNRQLEISEI

SRNLKNLARELNLPILCLSQLSRKVEERPGHRPMMSDLRESGSIEQDSDLIFFLLRREYY

DPMDKPGMAELIVAKNRHGSIGTVNLTFRKEIAQFANYTPIGMDHEPQGGKHPYEGSSNY

ATF

>tr|D6YTK0|D6YTK0_WADCW FeS assembly ATPase SufC OS=Waddlia chondrophila (strain ATCC VR-1470 / WSU 86-1044) OX=716544 GN=sufC PE=3 SV=1

MLLEIKNLSASIDGKPLLKGVDLEVNPGEIHAIMGPNGAGKSTLAKVLAGHPSYEVTGGE

VWFKGQNILEMEPDERAQLGLFMSFQYPVEIPGVSNMQFLHASYNAIKKASNQPELEEGD

FEKLLDEKMKIMDIRPEFKQRNLNEGFSGGEKKRNEILQMAVINPSLAILDETDSGLDID

AMRTVAGGVNHLMNDDMGLILITHYQRLLDHIRPHKVHVMVNGKLIESGGPELAIKLENE

GYDWLVKNIQEEMAG

>tr|D6YSS0|D6YSS0_WADCW tRNA uridine 5-carboxymethylaminomethyl modification enzyme MnmG OS=Waddlia chondrophila (strain ATCC VR-1470 / WSU 86-1044) OX=716544 GN=mnmG PE=3 SV=1

MWKYPAVYDVIVMGGGHAGCEAALASARMGAKTLLLTMNLDTIGKMSCNPAVGGIGKGHM

VREIDALGGEMGKVIDCTGIQYRMLNATKGPAVWAPRAQADKAAYQFEIKHRLEKQENLE

IHQGTVEEIFVENDTVQGVATKEGIYFTAPTVVISSGTFMRGLLHIGERNFSGGRAGDQP

SVGLSGCLKKLGFHLDRLKTGTPPRINKRSINLSLTEEQPGEEGIRFSFDDEGKRKLPQV

SCYITYTTQETKQIILDNIHRSPLYSGKIQGVGPRYCPSIEDKVVRFSDKERHQLFLEPE

GLQTEEIYVNGISSSLPLDVQYAFIKSIPALRNAEITRPAYAIEYDYVTSGQIKPSLESK

KVEGLFLAGQINGTTGYEEAAAQGLLAGINAASKVAGREPLILKRSESYIGVMIDDLITK

GLDEPYRMFTSRAEHRLLLRQDNADLRLRKYGYAYGLIDQKRWETLCLKAETIEWEMTRF

EKTFKQVNGKGFSLAQLLRRPEMTYESLLETYPEAVVDHGEEINFQIELNLKYSGYISRQ

NTEIERISQVENLRVPEHFDFNQVSGLRNEAKQKLCRHNPLTLGQASRISGVSPADISVL

IVELTKRERQTIA

>tr|D6YWC2|D6YWC2_WADCW 3-deoxy-D-manno-octulosonic acid transferase OS=Waddlia chondrophila (strain ATCC VR-1470 / WSU 86-1044) OX=716544 GN=gseA PE=3 SV=1 (cytoplasmic)

MYDILLCLVSLIALPKLLYQMAFHKKYRNSLKQRLGIGFPEIEKGNKKLIWVHAVSMGEA

KAVAALARELKKRSDNDAILLFSTVTETGLAEGEKELPEADYHVFLPLDFSWIIRPIIRR

VRPDQVIVCETDYWYNFLSSSKNAGARLSVVNGKISERSMHRLLKFPKFTKRLFCLIDKF

CVQSHHYRERFLKLGIPEEKIVITGNIKFDNSFPKLTEEELVEWKSKFGIRPEDHVLVAG

STHDPEERIILDACFEVWKDDPHLKILIVPRHPERFNEVAQLLKKRGVEFSRYSEGVSHE

APVILVDAMGVLLQCYQAATLAIVAGSFTPKVGGHNIVEPCWYGVPVLFGPYLYSQPELL

ELVQEYGAGVQVEPEHLSDEIKGLLSEPKRRKALGAAGIHLADSLQGATERTLVEIA

>tr|D6YWI5|D6YWI5_WADCW 50S ribosomal protein L33 OS=Waddlia chondrophila (strain ATCC VR-1470 / WSU 86-1044) OX=716544 GN=rpmG PE=3 SV=1 (cytoplasmic and periplasmic)

MASKREKIKLKSSKSSHHYYTFKNKTSTPDRIVLKKYDPTIRQRVEYKETK

>tr|D6YVS3|D6YVS3_WADCW Biotin carboxyl carrier protein of acetyl-CoA carboxylase OS=Waddlia chondrophila (strain ATCC VR-1470 / WSU 86-1044) OX=716544 GN=accB PE=4 SV=1(cytoplasmic)

MELKQIKDLMAAMGRTRLKRLKIKNDNFELELEREEKVVKQVVEHMPEAYARAETEIPRV

KAPDIPASLHPPVDHDSAVREEKGTFITSPMVGTFYSASGPDEPFFVKVGDRVTEESVVC

IVEAMKVMNEVKAGVSGVISEVLVENGHPVEFGTKLFKVS

>tr|D6YWC7|D6YWC7_WADCW Pyrophosphate--fructose 6-phosphate 1-phosphotransferase OS=Waddlia chondrophila (strain ATCC VR-1470 / WSU 86-1044) OX=716544 GN=pfkA PE=3 SV=1

MKEHTALEEKRLEYIPKLPAILHDLRKLKTVNLKNSPGKASEISDFFPLTIGQTALTFTI

DQDHEKTPLKVGVVLSGGQAAGGHNVITGLFDALKELHSKSQLFGFLNGPSGIVNNQTIE

LTEEILHSYRNQGGFDLIGAGRTKIETNEQFQGTLHTVKALDLDGIVIIGGDDSNTNAAL

LAEFFMKEGVRTRVIGVPKTIDGDLKNAYIDLSFGFDTAVKTYSGIIGNIARDSLSAKKY

YFFIKLMGRSASHIALECALQTHANYTLIGEEINEEKATFQQITNRLSDVICRRAELSKH

YGVILIPEGLIEFIPEFRTLIAELNEIKIDSELSKDERIQLAMRSISSESLECYKSLPRL

IQEQLMLDRDPHGNVQVSKIETERLFIEAVKQELKRRKEKGEYTGSFNAQPHFCGYEGRS

CLPSNFDSQYCYALGHVAALLIDANATGYMSCVKNLSRPIEEWQICGIPLTSMIHKEMRK

GKLKPVIAKALVDLQGAPFQYFKEKRLQWEDEDDYRYPGPVQFFGPSEITDAVTLTLELS

QNTEAILN

>tr|D6YU63|D6YU63_WADCW Nitrogen fixation protein NifU OS=Waddlia chondrophila (strain ATCC VR-1470 / WSU 86-1044) OX=716544 GN=nifU PE=4 SV=1

MTFELLTMSFPWNRYSRKLSAKIENPHNVGVFDPEESEARGMRLVIGIEGEIRDGNCVQI

YWLVDKEDGTIVDAKFQVYGQSALIGAAEVACDLIAGKNYDQAKRIGVDLIDKQVRDRSD

EPAFPQETAPHLNLVLDAIDHAADQCSDIPLPVAYAAPPAPKQFGEVLEGGYPGWEKMST

AKKLAVIEQVLNDEIRPYIALDGGGVEVKELKENELVIAYQGNCTSCFSAVGATLSYIQQ

TVQARVHPDLRVTPDINI

>tr|D6YTN2|D6YTN2_WADCW Putative biopolymer transport protein TolR OS=Waddlia chondrophila (strain ATCC VR-1470 / WSU 86-1044) OX=716544 GN=wcw_0118 PE=3 SV=1

MARRGIRHRREEGERPAVDLTPLVDVVFSILIMFIIVAPMLNLDRVELAEGAKEAKSSDV

SVRDSSLVTIHVREDNSIWINQKLVDAVHLPEILKQERQRHPDARPQLYHDRRAQFGTYQ

IVKNALESAGFEQLDIILKPV

>tr|D6YTM0|D6YTM0_WADCW Protein translocase subunit SecA OS=Waddlia chondrophila (strain ATCC VR-1470 / WSU 86-1044) OX=716544 GN=secA PE=3 SV=1

MISFFKKLFGTAQDRIVRRYSKLVSKVNEWDEKYKSLSDEQLQAKTDEFKQRLKSGELLD

NLLPEAFGAIKNACRRHVGTEVHVSGYHQQWDMVPYDVQIIGAISLHNGNISEMHTGEGK

TLTAIMPLYLNALTEKPVHLVTVNDYLAARDCEWVGSILHWMGISTGALTNDTPLEERRE

LYKKDVVYGTASEFGFDYLRDNSMAKRKEELVQRGHYYAIIDEVDSILIDEARTPLIISG

PAPESRQMYDELKAGVSELVRRQRDLCSKLASEAKKVIDLGDRSEAQDKSQTKEEKQREE

EAFRKLWLVSKGTPRNKILKRIRENPDARAAIDEWDLYYYSDSNKEEKAEKLSELYVVVD

EKSSEYELTDRGIAMWHEFTHGDGQGEDFVMLDISEEYLKIDLDNSLTDEEKMQKRLEIQ

EEDAKRKERAHNLRQMLRAHLLMEKDVDYIVQDEKIVIIDENTGRPQPGRRFSDGLHQAI

EAKEGLKIQKETQTYATITLQNFFRMYEKLAGMTGTAITEAGEFKQIYKMEVLEIPTHRP

CIRKDFNDEIYMTEREKYNAILKDVKEIHELGRPILIGTESVEVSEKLSRIFRQNKLEHT

VLNAKNHMKEAEIIAEAGRRGAITIATNMAGRGTDIKLEKGIAELGGLYVIGTTRHQSRR

IDRQLRGRCARQGDPGSSRFYVSFEDSLLRLFASPRMTQILKKFRPPEGEPISATILNKS

IETAQKRVEQRNYTIRKHTLEYDDVMNKQRQEIYEFRNDILQTDLIEEVACELIEHVCVD

AAEEHFHSRTDEQGWDPEGFRNWIMTQFPVSFEEGEFDDDHSDTEELAQKAVNVIIDAFR

KRLENENAKVAYDLPEGVQAPSKPANEALRHLMIRKIDKDWKEHLLTMDHLRSDVNMRAV

GQRDPLMEFKHEAFRLFDLFGKKVRKEITHDLFRFEIIAPEAQEIEQLLNRLQMERNRSF

LSDFGEQVPKSITEGAPSPMPYEAMKPTEFNQQEVEKELPVTVPPKTGRNDPCPCGSGKK

YKKCCGIHQADE

>tr|D6YRZ3|D6YRZ3_WADCW 30S ribosomal protein S8 OS=Waddlia chondrophila (strain ATCC VR-1470 / WSU 86-1044) OX=716544 GN=rpsH PE=3 SV=1

MAVTDPVADFLTRIRNGLKAQHRYVDINWSKMKQSLADILKNEGFIENYLVKKDNNDRGT

IRVFLRYGAYRQPAIKGLKRLSRPGLRKYVKHNEIPKFYGGLGVSILSTSSGILSGNEAQ

NKKVGGELLCLIW

>tr|D6YVE5|D6YVE5_WADCW Aspartyl/glutamyl-tRNA(Asn/Gln) amidotransferase subunit C OS=Waddlia chondrophila (strain ATCC VR-1470 / WSU 86-1044) OX=716544 GN=gatC PE=3 SV=1

MAHLDKQMIEYLSDLSRIDLSEEEQQSLLEDLEKILAYIDLLNEVDTEGVEPCNHVLADM

RNVMREDEVGETMPREAFLNNAPAQIGGMIRVPPVIKGK

>tr|D6YVA5|D6YVA5_WADCW tRNA (guanine-N(1)-)-methyltransferase OS=Waddlia chondrophila (strain ATCC VR-1470 / WSU 86-1044) OX=716544 GN=trmD PE=3 SV=1

MRMDILSLFPDYFKGPFDESMIRRAIDAGILDIRLVDIRDFAEGKHRRVDDRPYGGGPGM

IMMPEPAVQAIRSVRQPEAKVIYLTPQGKPLNAAKCRELAEESHLIFLCGHYEGIDERVI

DIEVDEEISIGDYVLTNGCAAAIVCVDAIARFVPGVIGHESAADEDSFENGLLDCPHYTR

PEVFESLSVPEVLLSGNHKKISLWRREKALEKTQRIRPDMADRRCKEK

>tr|D6YRZ1|D6YRZ1_WADCW 50S ribosomal protein L18 OS=Waddlia chondrophila (strain ATCC VR-1470 / WSU 86-1044) OX=716544 GN=rplR PE=3 SV=1

MKTVEYKQNQKRKRRVFRVRKQLRGTSLKPRLCVVKSNQNIEVQLIDDNKGITLASTSTR

SKDLKNTEFNKCNKESARKLGEIIAERAVKQNVKEVVFDRGAHKYHGILKELADAAREAG

LKF

>tr|D6YVR2|D6YVR2_WADCW Ribosome-binding factor A OS=Waddlia chondrophila (strain ATCC VR-1470 / WSU 86-1044) OX=716544 GN=rbfA PE=3 SV=1

MTKQRIDRLNSLLKEVISEVIRKDVDNPEVGEFVSVTRVDISKDLRHAKVYISIIGDEDT

KQKTLKALTTASGFIGINASKKVVMRFFPALRFIIDDTVDKVMRIEELLQEIHEEQDSRI

PPKAADDTLF

>tr|D6YWM6|D6YWM6_WADCW Beta sliding clamp OS=Waddlia chondrophila (strain ATCC VR-1470 / WSU 86-1044) OX=716544 GN=dnaN PE=3 SV=1

MKFVISTQEFNYLISKCQHVVPPNPTMPVLSNILIEAKNGELILTATDLTVGVRCFTEAK

VLEEGSTTLPVKKLASLIRELTSMNVEVTTSENHVTEIISDTSKFKLNGMAGGNYPELPS

FADATPIKLTQQQLKEMFFRTSFAVSKDDTRYALTGVFLHIENGRACFVGTDGKRLARTR

LDLEIDPSFSGGYIVPIKAVDEILKNLKEEGDATLYLLNDKIAVETENSLHVTKLLSGDY

PDVNRVIPDRSGQSLTLHREELLTLLRQVSLFTAEGYHSVRFSFGNGEVKLSANTKDVGE

GNVSMPVNYHADQIDIAFDPTFFMDILKHTRGERVTLGVTDSFNPGVITDTEEGTEDSPL

FVLMPMRLSENAG

>tr|D6YS67|D6YS67_WADCW Glutamyl-tRNA reductase OS=Waddlia chondrophila (strain ATCC VR-1470 / WSU 86-1044) OX=716544 GN=hemA PE=3 SV=1

MQIGVVGVNHKLADLKLRESFAKVCNEYFRDTFARHGKHNTLLLTTCNRTEVYFSSDVLS

ESHSYVLNILRENLPVSDETFDQKLYTYFGHDCFIHLARVTAGLDSAIVAETEIQGQVKI

AYEKAADAAILPRELHYLFQKSLKIGKSIRSELGLGRGVPNLEHAVLNAGFHFFDKPEKA

NILFIGASDINCKILSFLQSKGCKHLTLCNRTKSNALEASKKYHINILDWEQRKMWTAFD

WVIFGTKAGDHIISKSDLHQKPASDLLLIDLCVPRNVDPKLGREESITLLNIDQINRMLK

FRKKRLNNHLSKAEKIVYTSTKRHIDLFHSKEERKLQLLATG

>tr|D6YRU1|D6YRU1_WADCW 30S ribosomal protein S21 OS=Waddlia chondrophila (strain ATCC VR-1470 / WSU 86-1044) OX=716544 GN=rpsU PE=3 SV=1

MTTVKVRPGESIDKALRALKKRLDKEGVMKSVKAHRFYMKPSIKKRAKSKAALKYRR

>tr|D6YTN8|D6YTN8_WADCW Glutamate racemase OS=Waddlia chondrophila (strain ATCC VR-1470 / WSU 86-1044) OX=716544 GN=murI PE=3 SV=1

MNAIAVFDSGLGGLTVVKALCEHLPQEEIIYFGDTARVPYGGKSRETVIRYAREISAFLL

SQEIKALVIGCNTASAYAADLLAAELELPVFNVIDPLIEEISACQAEHIAVLGTAATVRS

GIYQARLAQKIPNVKVTGISCPLFVPIVEEHFQNHPAARLIVEEYLFRVKDERMDTVVLG

CTHYPLLYCLIREYLGDEVRIVDSASACAKQIQAALFSHPFNNSLSVKGKLKYFVSDDPE

RFKHLGEQFLGVEIGPVECVDLNSVLQIPQNFAKSG

>tr|D6YV90|D6YV90_WADCW tRNA(Ile)-lysidine synthase OS=Waddlia chondrophila (strain ATCC VR-1470 / WSU 86-1044) OX=716544 GN=tilS PE=3 SV=1

MNLYTSHFLPFLKRCTPFNAPVLIACSGGPDSMALLRMMVEYRKTHSVRFGVAHVDHRWR

KESADEAETLRALCREIDVPFHLKEIDPEAMQGNLEEACRHFRQTYFLQLCFDHGYGSVM

LGHHLDDQAETVLKRVFEGAKLEKCGGMQEISVYETIPFWRPFLQVRKQKLIAWLNLRNF

PYFIDPTNNDSSFLRSKIRGNILPEISRYFGKEVSPGLAFLGREAHALKEFMGSHIYKWL

ALKEKTPWGTVLDLSCQNPSHLFEARCLISELLPQASREIAYSAAENLIGGAANKCYHAG

GKTLYIDRKRMFLTERLHADLPHQETSLKKCGSIGSWNYRIQSASNKRLSGWKQVLYGVL

QWPVGGNFEKLRIGSPATVHCQDELNRMRRQARVPVFMKNWAPVITRENEIVQDFLSGKV

EQQAADAPLEVVLTKAMD

>tr|D6YUU2|D6YUU2_WADCW Putative SAM-dependent methyltransferase OS=Waddlia chondrophila (strain ATCC VR-1470 / WSU 86-1044) OX=716544 GN=wcw_0532 PE=4 SV=1

MDSTEKKCRHGCFLMTEKNKIHQGNVIDTIDGIDVIECTVCGFKHVNPLPSQEELETLYK

KEYYSLDKPQYLKDSEEDQEWWEMTYRQYFHLFDKHLKKEAPSLFEIGSGPGFFLKVGKE

HGWDVLGIEPSKQAVAFSKQFDVPVIHDFFHEETAKKLGTFDLIFMDTLLEHVPNPSSMI

ALCSSLLNPGGLLCVISPNDYNPLQKILRNQKNYPPWWIVPRHHLNYFDFTSIQSLFKQH

QLQVVESLGTFPMEFFLLCDDNYIENHALGREVHGKRKTLEKLMLANNPKLLESFYQWLG

MQGLGRSFVVIAKKPS

>tr|D6YTE0|D6YTE0_WADCW MinD/ParA chromosome partioning protein OS=Waddlia chondrophila (strain ATCC VR-1470 / WSU 86-1044) OX=716544 GN=minD PE=3 SV=1

MATRKKIAIAISSFKGGTAKTSTALHIGSALSQFHKQKTLLIDFDAQANLTTGLGFDPDE

HDSLAPVLQGNKEVKEVILPTNVKNLDIIPADTWLERVEVTGSLAADRYSHERLHDIIEP

LDYDVVIIDTPPSLCWLTESAMIAAKHTLICSTPEFYSVKGLERLSQFIESIGQRHPLNV

LGVVLSFWNQRGKSNTTFLDVIEKTFPQKVLKTKVRRDIAVSEASIFGKPLFETAPKSRA

AQDYKTLSKEILDRL

>tr|D6YRZ8|D6YRZ8_WADCW 50S ribosomal protein L29 OS=Waddlia chondrophila (strain ATCC VR-1470 / WSU 86-1044) OX=716544 GN=rpmC PE=3 SV=1

MIMKPQEMRDQSIEELVAKLEESKRELFELKNEMKRSKKLEKPHLLREKKKDIAKFNTII

REKQLANR

>tr|D6YSM8|D6YSM8_WADCW Tetraacyldisaccharide 4'-kinase OS=Waddlia chondrophila (strain ATCC VR-1470 / WSU 86-1044) OX=716544 GN=lpxK PE=3 SV=1

MINELHFYYIQVIRGKRTGWLPTLIKGLAWVLSLPYRWVMSLRNWLYDHEWLRQYDAPVP

VVMSIGNLVTGGTGKTPVTKLLAGFFYDDYKIAILSRGYRSPAEKLRAPVILSSGKGPLH

SAAYAGDEPRLLAENLPKAWVVVGKDRVMSANLVAKQGVDLILLDDGMQHRRMARDFEVV

VLDAKDPFGQNYLFPRGLLRESPEGLRRADLVILNHVRDAEDYEDSKKMVEKYTNAPVIG

IHYDRWKAMDLEGNELAPLEGRKVAIFCGIAQPEQFASTVREMGAEIVARKYYPDHFHYD

VEELSELAARWKEMGAAMMVCTEKDKVKLPEIHDLLLPVVWIKIQPEVIEGTDELKAFID

KVKAKIA

>tr|D6YW22|D6YW22_WADCW Aspartate-semialdehyde dehydrogenase OS=Waddlia chondrophila (strain ATCC VR-1470 / WSU 86-1044) OX=716544 GN=asd PE=3 SV=1

MKRKIPVAILGATGSVGQSFVRLLKDHPWFYIAELVASKRSAGKKFGDFVPNTHLEDHFI

KAVSDSLSSKIVFSALDAQVAGEIESSLARKGHWVISNCRNHRYDPDVPLLIPEVNPEQL

HWIKRQQFGGGVIVTNPNCSVIGLALTLKPLVEAFGVEQVHVVTLQAVSGAGFRARTILD

IDDNVIPYISGEEEKIEKELDKILCIKRVSAQCNRVSVSDGHTQCVSVKLSQTASLEEIK

SAWTAFRSPVLPSSPSKPIHYFNEESYPQPKLHRMLEKGMAVSVGRLRKCSLFDIKYATL

SHNTIRGAAGCAIMNAELLIDHHYNGDIVGASSLIGQCP

>tr|D6YUZ0|D6YUZ0_WADCW Cysteine desulfuration protein SufE OS=Waddlia chondrophila (strain ATCC VR-1470 / WSU 86-1044) OX=716544 GN=sufE PE=3 SV=1

MFESCIKKQQNVKKLFESCRSKEEIYQKIIEIGRSSLGLEAEYKIPANEVQGCQSLMHMR

AFLKNGKLFFEAESEALISSGLAALLTHVYSGEEPIVILKCPPDYLEEIGVSSSLSPNRA

NGLYHIHLRMKQIALETFMEEGS

>tr|D6YT81|D6YT81_WADCW 30S ribosomal protein S20 OS=Waddlia chondrophila (strain ATCC VR-1470 / WSU 86-1044) OX=716544 GN=rpsT PE=3 SV=1

MAEKEAKKKEKRSTAFKRDLQNRKKRADNRVFKSRVRTAIRRFEEVVAKNEQESVAETLS

VVYALMDKGVKKGLYKLNMASRTKSRLAAKAARAK

>tr|D6YWZ1|D6YWZ1_WADCW D-alanine--D-alanine ligase OS=Waddlia chondrophila (strain ATCC VR-1470 / WSU 86-1044) OX=716544 GN=ddlA3 PE=3 SV=1

MLDLAVLFGGRSIEHEISVITGLQAILALNPKKYKILPVYFALNGKWYTGDPLLERSFYR

KLPAAYSDLTEITLLPDPSIGGFKDLALNRTYPVDRCLLCFHGQYGEDGCVQGLLELAGL

PYTGSRVLSSALTMSKSHTKLLLQALNIPCLPHVVVDKQEAVSDLAAVRKKILDTLRYPL

FIKPNHLGSSIGIAKATDKNSLDRALAKVFLYDSAAIIEPYLDNLLEINVSVLEGDPPRS

SAVEIPIASQEALSYEDKYLKGGAKSTGGSSQGMAGLTRMIDPQDLDLKIKQGVINHALK

AFKELSCSGVCRFDFMLDTSTETLYFNEANPIPGSLSFYLWDRTEPKLLYSELLDVLIER

CAHKSALQRSLKKDFGFQAL

>tr|D6YSE7|D6YSE7_WADCW Transcription termination factor Rho OS=Waddlia chondrophila (strain ATCC VR-1470 / WSU 86-1044) OX=716544 GN=rho PE=3 SV=1

MESEKNSPNVASAVPEEKKPLQKNGNEQERKAARTTKISDIQRMNIDQLNQLGKQLGIKH

TGSLTKSQMVFEIVKSISENPNEILYGEGVLEILPDGFGFLRSTNYNYLPSAEDIYVSPA

QIRRFDLKKGDTVKGTIRPPKEKEKFFALLRVDTINDQTPDQARERIHFENLTPLYPDER

LVMETASEKLSTRVLDLAAPIGKGQRGLIVAPPRTGKTIILQNIANAIATNNKEITLIVL

LIDERPEEVTDMQRIVKGEVISSTFDEPPERHVQVAEMAIEKARRLVEHGRDVVILLDSI

TRLARAYNTVQPHSGKILTGGIDANALHKPKRFFGAARNIEQGGSLTIIATALIETGSRM

DEVIFEEFKGTGNMELVLDRRLADRRLYPAIDLIKSGTRKEELLYHPSELEKIYLLRQAV

ADLTSVDAMNLLLGRLKKTKQNVEFLLSMKD

>tr|D6YRZ7|D6YRZ7_WADCW 30S ribosomal protein S17 OS=Waddlia chondrophila (strain ATCC VR-1470 / WSU 86-1044) OX=716544 GN=rpsQ PE=3 SV=1

MTAKGTAKRKTKTGTVVSNKMDKTVIVRVERTMQHPRYGKVIKRANKFYAHVENDQLQIG

DTVTISETRPYSKLKRWRVVEKN

>tr|D6YS13|D6YS13_WADCW 3-hydroxyacyl-[acyl-carrier-protein] dehydratase FabZ OS=Waddlia chondrophila (strain ATCC VR-1470 / WSU 86-1044) OX=716544 GN=fabZ PE=3 SV=1

MPSDTLDYPETLDIKEIIKILPHRYPFLLVDKILKVDMEKGYILGQKNLTINEAFFQGHF

PGAPIMPGVLILEALAQTGAVLVHLSGQKEKVGVLLNVKNAKFRNPVKPGDILYLEGEGI

HISSKGGKVRATATVNGKVAVEAEMGFALVDKSQI

>tr|D6YWZ5|D6YWZ5_WADCW 3-dehydroquinate synthase OS=Waddlia chondrophila (strain ATCC VR-1470 / WSU 86-1044) OX=716544 GN=aroB PE=3 SV=1

MIINKLDNWMPVIEHPVIVTDHIVEELYGASLAKRLNCELISIPPGETSKELERFYELTR

KMALLNCNRKTTVIALGGGVVGDLAGFAAATYMRGISYIQVPTTLLGMVDSSIGGKVGIN

LPEGKNLLGAFYQPQEVVVPLNCLETLPEKELRSGMSEVVKYGVILDADFFKWLEENIEK

LLNKDSDALKTAVKRCAELKMEVVNQDAKETDLRQILNYGHTFGHALEKITKYQEYTHGE

AVALGMRFEGALASVRYQFPAKLLERQNALLDKIHPRLSPPQIDDGELMEAMKGDKKSVG

GKTVFVLPQWLGKMVRETNGYGIVIPQKAVHECLRSHYSSYAS

>tr|D6YUZ8|D6YUZ8_WADCW 50S ribosomal protein L7/L12 OS=Waddlia chondrophila (strain ATCC VR-1470 / WSU 86-1044) OX=716544 GN=rplL PE=3 SV=1

MSTKTEKLVEELSKLTVLEMSELKKALEEHWGVEAAAAAVVAAPVAAAAGGEAAEEEATD

FEVTLTEVPADKKIAVIKIVREVTGLGLKEAKELAESAPKVLKESAPKAEAEEIKKKLED

AGAKAVLKGV

>tr|D6YWF3|D6YWF3_WADCW 3-deoxy-manno-octulosonate cytidylyltransferase OS=Waddlia chondrophila (strain ATCC VR-1470 / WSU 86-1044) OX=716544 GN=kdsB PE=3 SV=1

MTYRIIGMIPARYGSSRFPGKPLVEISGKSLIQRTYENAQRCKLLQEIYVATDDDRIFSH

VEEFGGKAIMTSSNCPTGTERLAEAIHLNFKNVDMIINIQGDEPLLEPYVIQKVGEILIN

DQSAVMSTAAVKISTEEEALSRSVNKCVIDAHGNALYFSRSLIPGGHSGKWEPDTTYYKH

LGIYGYRKDFLFHYAELETTPLQLAEDLEQLKVLEHGFKIKVAVVDSHSIGVDTPEDLIK

VERKL

>tr|D6YUZ6|D6YUZ6_WADCW 50S ribosomal protein L1 OS=Waddlia chondrophila (strain ATCC VR-1470 / WSU 86-1044) OX=716544 GN=rplA PE=3 SV=1

MGRLSKRFREIAESIDVQKQHTLEEAVELLKQLPPVKFDQSIEVALKLGVDPRKSDQNVR

GTVSLPNGTGKTTRILVFARGDKIQEALDAGADYAGDDEYFEKVGKGWTDFDAVVATPDL

MRDVGKLGKILGPRGLMPTPKAGTVTNEIGKAIQDLKGGKIEFKVDRYGVVNAAVGKLSF

TVQQLVENIKSLIDAIQKQKPASSKGQYMKSLVLSSTMGPGLKIELRKVETI

>tr|D6YVK0|D6YVK0_WADCW Transcription antitermination protein NusB OS=Waddlia chondrophila (strain ATCC VR-1470 / WSU 86-1044) OX=716544 GN=nusB PE=3 SV=1

MIQMAVPQQKIREIVFQLLYGHDWHSPEKEAFHTFLSKEAKISKKVILEAQERVDLIRGR

EKELDQLIGESAKSYAFDRIPRVERNILRLGIYELLFDEQIPPKVAIAEAIRLGRKFSTP

ESATFINAIMDELYQKRCTPQVKDE

>tr|D6YRP2|D6YRP2_WADCW UDP-N-acetylglucosamine--N-acetylmuramyl-(pentapeptide) pyrophosphoryl-undecaprenol N-acetylglucosamine transferase OS=Waddlia chondrophila (strain ATCC VR-1470 / WSU 86-1044) OX=716544 GN=murG PE=3 SV=1

MDRRKVVIAAGGTGGHLFPALSLAHQLEKRGDSILFAGGKLGANSLFDKGRFPFQEISCA

RPTFKSPLFPFKIAKGIVQSVNIFRTFKPDFLIGFGSYYTFPVLAAAKMMKVPFVIHEQN

RVPGRVNRLFTSSASFTAIHFPSVAGKIKGKCQLVEMPLRPGFEKRWDPVEAKREYGFSD

ELPVILVFGGSQGAEAINQLLYDSAELLTRFQILHFTGTVDGEKKLARRYGEAGIKAHVR

VFEKEMARAWSAADLAVSRAGAASIAEQLAAAVPGILIPYPYATDRHQDANADYLISLKG

AVKIPQSQLSPATFIEAIDSMLPKLESMRSALLGKETQQIRFIDCLEDL

>tr|D6YTJ2|D6YTJ2_WADCW Cell shape protein MreC OS=Waddlia chondrophila (strain ATCC VR-1470 / WSU 86-1044) OX=716544 GN=mreC PE=3 SV=1

MRSFSPKPFLQLLAVLLTLLSVSKSTSEKVQGYSIAILAPTWNQLAIIKQGIQHLSEETF

EEDGKTLYVHEEIQKLHIENQLLKNEIATLKELLKQEQKRDLVTSKELEAAPAQVIFRPG

TSWNTVLWINVGKEHNKQLGKQVISQNSPVVVGTNVVGLVDYVGNKQSRVRLITDPSLTP

SVRSVRVVDGRTWYLAKGELKGSSLSQYRTKEKLLIGTGFNYDFPDQEGPARDLRSGAVL

GDQSKHYPALDIIMPNDLLVTTGMDGVFPSGLCVATVTKVNMLKEGDYYYDIEAEPCCTN

LDHLMIVSVLPPQGFDPCDQPSPYQLPN

>tr|D6YVA0|D6YVA0_WADCW 50S ribosomal protein L31 type B OS=Waddlia chondrophila (strain ATCC VR-1470 / WSU 86-1044) OX=716544 GN=rpmE2 PE=3 SV=1

MKQDTHPEYQDVLFVDSSTGVKFICGSTLKSDKTETFEGKEYPVCNVSVSSASHPFFTGS

TQFVDAEGRVDKFKKRYAVTRKKKAQD

>tr|D6YW62|D6YW62_WADCW Thymidylate kinase OS=Waddlia chondrophila (strain ATCC VR-1470 / WSU 86-1044) OX=716544 GN=tdk3 PE=3 SV=1

MMFISVEGGEGSGKTTLIRNLAEYLESKGDQVLITREPGGTRFGESLRKVLLDPEFPAAF

NARAEMLLFLAARVQHIEEVIQPALRKGKTVICDRFNDSTIAYQGGGRELGVVRVEQLCF

EVCDGFQPDKTFVLDINPETAFQRLNRSKDRMEKESFDFHNKVRSAFYALKDKHPNRIHL

INADQSKEKVLADVLKYLQ

>tr|D6YWA2|D6YWA2_WADCW Chorismate synthase OS=Waddlia chondrophila (strain ATCC VR-1470 / WSU 86-1044) OX=716544 GN=aroC PE=3 SV=1

MGSNQFGQYFQVTTWGESHGKEVGLVIDGCPPNIKICEEQINDALKKRAPGRTPWTSPRK

ESDRAEILSGIFEGKTTGHPIAIRIPNLDVRSSAYQGMEKILRPGHANAAYLEKYGIYDY

RGGGRASARETACRVAAGAVAEQVLEGVEVVAYLKEAGSVTIGNVDESHLANSPIFCPDP

YTEASMISMIERTLEEKDSVGGVVEVIARNLPAGLGDPVYQKLEANLAHAMLSIPASKGF

EIGSGFQAARMKGSEHNDLYTSLRKTETNHAGGTLGGISTGMPVICRVAFKPASSIGKAQ

KTVTIDGEPAIYELPQGSRHDPCVAIRAVPVVRAMMLIVLADRWVKRFDKN

>tr|D6YRP5|D6YRP5_WADCW UDP-N-acetylmuramoylalanine--D-glutamate ligase OS=Waddlia chondrophila (strain ATCC VR-1470 / WSU 86-1044) OX=716544 GN=murD PE=3 SV=1

MNVLVIGLGISGRSAAAFLMKRGHRVAAIDQRAETLRSDPLVVPLIDKGLTLLNTQCPGK

IEVFGQVVASPGISLTHPAIAEALKRNIEVIGEVELACRHLDQPMIAVTGTNGKTTVTQL

IGHVLNRCGKPAKVLGNGGIPLTAELDALQKEMIVCELSSYQLETLNSRVVDVGAVLNIT

PDHLDRYLNMEAYAKAKLRLANCLKSDKKLFLSQQVINDFGYLISDLKENVELIVDSNYK

SRLTHDEENCLAAKRICLEMGVLPEAFDCAESEFQKPPHRIEFVRKRRGVSYFNDSKGTN

LDAVKRAVERMDGPVILIAGGKGKGISFCPWIKPFSGKVKAIVAIGEARSLLLKQLGGEF

EVIEESSLKAAVAAASSIANDGDNVLLSPGCASFDMFSNFEERGDHFKECVQLLD

>tr|D6YST7|D6YST7_WADCW Glycerol-3-phosphate dehydrogenase [NAD(P)+] OS=Waddlia chondrophila (strain ATCC VR-1470 / WSU 86-1044) OX=716544 GN=gpdA PE=3 SV=1

MKIGYLGAGAWGFALASLLASKKYEVVSWTISEELADRLNRSEEHPMLPGSKKFPNQRLT

TDLQDAVQGADLIVESVTSAGIRPVFEQLKKVELPSCPIVITSKGIEQDTGLILSDVISE

VLGEGVRCRIGAISGPSYAAEVVKGQPTSVTGSAYDPAVMETVCKAFNTEAFRVYPNHDM

RGVALGGALKNVIAIACGVAEGLNLGYSARAALMTRGLHEIRKLAVSMGANSETLYGLSG

MGDLTVTCSAMTSRNFKFGYLLSQGKGVKDAKKEIGMAVEGAYTCVSALQLSKKLNVPMP

ISECVYQVVFEGLPPKEAVVQLMKREIKQEHL

>tr|D6YWZ6|D6YWZ6_WADCW Shikimate dehydrogenase (NADP(+)) OS=Waddlia chondrophila (strain ATCC VR-1470 / WSU 86-1044) OX=716544 GN=aroDE PE=3 SV=1

MNVCVPITARTLPEALKEISFACSIAEMIELRLDYLDHPSPEGINELVQACEKPVIATYR

KKNPVPLLKAALDAGADYVDVEEINTSLPLERCIYSIHLQETPPLEELYQSMKASGAAII

KIVPTARSFIDNIAILNLLKKYPEDRLIAFCMGEKGLLSRYFAPAYGSFCTFGAIAEGKK

SAPGQPLATHLRKWKAQVNTTLCGVIGDPITHSLSPAIHQAAYEADGLNFLFLPFHTTKE

DLPKILDLMRQFPLRGLAVTVPHKESVIPMLDEVDVEAQAIGAVNTIINHGGKLTGYNTD

VFGAVQPLQKRTDLRGKRVAILGNGGAAKAFAYGLQKEGSVVTLFGRNLEKLKEQLMESE

ILIQTTPVGMAPNTDKTLVPPDFLHQNLLVFDCVYNPLKTQLLKDAEKAGCRTISGLEMF

MIQAREQYKFFTSQEANEEAMKKAALNAFPSSKTKF

>tr|D6YWN4|D6YWN4_WADCW Malonyl CoA-acyl carrier protein transacylase OS=Waddlia chondrophila (strain ATCC VR-1470 / WSU 86-1044) OX=716544 GN=fabD PE=3 SV=1

MKNIAFLFPGQGAQYPGMGKDFYESYQTARAVFEEANERLNRDLASIILNGPEETLTETR

NSQVGIYVVSIAILRVLSELFPEMEPSICAGLSLGEYTAATSAGYLSFDDGLPLVQARGQ

FMNEACESTQGAMAVILGLSPAQVEQIVKSVNLPNDLWAANFNCPGQVVVSGTRKGIDAA

AIAAKSMGAKRVLPLQVHGAFHSGLMLDAQKRLAEYVNGTAFGEPKAELVMNVTGEIVEE

ASVVRDLLISQVSSPVKWQQGIETIEKRGIDLYVEMGPGKALAGFNKRIGVQAPTISVEK

IEEIKQLEEALRCGNF

>tr|D6YS55|D6YS55_WADCW 2-amino-4-hydroxy-6-hydroxymethyldihydropteridine diphosphokinase OS=Waddlia chondrophila (strain ATCC VR-1470 / WSU 86-1044) OX=716544 GN=folK PE=4 SV=1

MKMDQAYVGMGGNIGDSIAILNQSLEEMRAIHEITNLRCSNFYKTSPVEAIPQADYVNAV

CTFQTSLKPIELHKRLEEIERKMGKLPKPKNHPRVIDLDLLLHGLEVVDEKNLKIPHPKW

NQRLFVLIPMRELVSRLAVKNKTGELMTVDLEKMIQHFDNVNNEQLEILR

>tr|D6YU85|D6YU85_WADCW UDP-N-acetylglucosamine 1-carboxyvinyltransferase OS=Waddlia chondrophila (strain ATCC VR-1470 / WSU 86-1044) OX=716544 GN=murA PE=3 SV=1

MLSDFKATEVMEVVGGKPLMGKIKASGAKNAMTKLLVASLLSDKKCTFFNVPNIGDVQIT

VELCREIGMDVKWDKEAGVMEVLTRTLRTSYIPQRFSGSNRIPILMIGALLGRTDEEIIV

PTVGGCVLGPRPVDFHISALRQLGATIEFRRMKREGAYFAHAHEGLKGTVIELPFPSVGA

TENTILAGVAARGTTEIRNAATEPEVVDLILFLQKLGANITLDVDRTIRIQGTRRFYEVE

HTVIPDRIEAASWGMAAIASKGRVFVEGAQHQHLLTFLNKIREIGGGYHVRHEGIEFFYD

GPLQGGIHLETDVHPGFMTDWQQPFVVLLTQATGTSVVHETVYENRFGYTDTLKEMGAEI

TLFRQCLGGKECRFSSQAFSHSLIVKGVSPLTGREINIPDLRAGFSYVMAALIPKEKSVI

KGLSFLDRGYENLDQKLLSLGADISRKSLKEKNSKAAEEQELIFSSFR

>tr|D6YRY9|D6YRY9_WADCW 50S ribosomal protein L15 OS=Waddlia chondrophila (strain ATCC VR-1470 / WSU 86-1044) OX=716544 GN=rplO PE=3 SV=1

MHKLNNLKNSSKVTKSRRRVGRGIGSGVGKTCGRGEKGAGSRSGYTRRLGYEGGQFRLFM

KLPIRGFSNARFQKKLDSINLGQIDKLYQDGDVVSIETLRQRGFINGKSHGLKILGEGEL

TKKVTIEADKISVSAKDKLQKANISFSLKSSDDE

>tr|D6YRJ0|D6YRJ0_WADCW Integration host factor subunit alpha (IHF-alpha) OS=Waddlia chondrophila (strain ATCC VR-1470 / WSU 86-1044) OX=716544 GN=ihfA PE=3 SV=1

MSFDDKKSTVTKKKLINAISQDKGIHPNDVRHVIQAFLDKMTDCLTNGERLEFRDFGVFE

VVERKQKIGRNPKNASVPIVIPARPAVKFTPGKRMRKLIEKTPEELASVE

>tr|D6YRT2|D6YRT2_WADCW HPr kinase/phosphorylase OS=Waddlia chondrophila (strain ATCC VR-1470 / WSU 86-1044) OX=716544 GN=hprK PE=3 SV=1

MYLVEDLYKRHGARLGLELISGSVGMKRRIKVPEAHRPGLSLSGYLKGHADKRILIFGKV

EIEYLRDLKPSVRVERLEGVLVTPTPAVIVARRFRPPKELIHLCEERGVPLFRASMSTMN

LLSKLTLLLNEEFALSMSVHGTLVEVFGVGVLIQGDSSVGKSEAALGLIERGHRLISDDI

VKVKKREGHYLEGSGAELTRHHMEIRGIGIINVANLYGAVCVRDYKSIDLVVRLESWDEQ

SFYDRVGLDEKFCDILGVKLPLHILPVKPGRDVVLLLETIALNHRLRGMGYHSAKEFNSK

LLNMINKKPQSRSFDSEISTKSPS

>tr|D6YVJ0|D6YVJ0_WADCW Dihydropteroate synthase OS=Waddlia chondrophila (strain ATCC VR-1470 / WSU 86-1044) OX=716544 GN=folP PE=4 SV=1

MAILKTQIMGVLNATPDSFYSQSRCFRNDLAIARGLELFQKGADIIDIGGESTRPYADPV

SEEEELERVVPVIKALKKQLTIPLSIDTMKAKVAKAALDAGASIINDVSGLQDPEMIAMA

ADTGARICVMHMQGTPKTMQANPIYPKGIIKELLFWFEDKLKALQKAGIKENNIIIDPGI

GFGKTVEDNYQILHNLQKFKELGFPVLLGLSRKSFMGKVLNKPPEDLLAPTIALGALAMK

ENIDILRVHDVEEHRSTAVIMEKFLSCSEMKGRHINA

>tr|D6YRZ5|D6YRZ5_WADCW 50S ribosomal protein L24 OS=Waddlia chondrophila (strain ATCC VR-1470 / WSU 86-1044) OX=716544 GN=rplX PE=3 SV=1

MQKKKKQNKKIRTGDRVMVIAGNDKGLVGTVQSRTEDRIIVAGVNVRKKHVKKSQLNPQG

GIIDMERPIHISNVTLCVDEEKPRKLRVGIDHEGNRILVYREGDTDATYRSVKKKGE

>tr|D6YVL2|D6YVL2_WADCW Ribonuclease P protein component OS=Waddlia chondrophila (strain ATCC VR-1470 / WSU 86-1044) OX=716544 GN=rnpA PE=3 SV=1

MRACFSKQSRLLKKYQFLRVVRYGKRHAGNFLVVESVKNRLNRSRLGITVSRKFGKAVQR

NRFKRLIREGFRQLGSLRNSGIDIHVKPRSKAKDVDFEMMRTELSALLKFVLESC

>tr|D6YSM5|D6YSM5_WADCW Carbohydrate isomerase, KpsF/GutQ family OS=Waddlia chondrophila (strain ATCC VR-1470 / WSU 86-1044) OX=716544 GN=kpsF PE=3 SV=1

MINSVIPELLEKERSYLNHFFDNIDMEAVDAVLQELVNCKGITVFTGVGKSGLVAKKMAV

TMTSTGTRALYLSPTNALHGDIGILKPDDLFIVLSKSGESDELMNLIPFIRNQGVKVVSI

VSNQDSRLAKASDIVLFISPERELCPFDMAPTTSTTIQGIVGDVLAIALMRLKKVSIEDF

VKSHPAGRLGKRATILVKDLMLKGDAVPVGKGDDKLVDSLVELSNKQCGCVIIVDDDRRM

KGIFTDGDLRRALQKYGVDALESPLERLMTKTPRSISPNMLAYAAVKEMESNQKSPIMIL

PVLDEEGRVVGVVKMHDLLQAGI

>tr|D6YUB5|D6YUB5_WADCW Putative cell division protein FtsI OS=Waddlia chondrophila (strain ATCC VR-1470 / WSU 86-1044) OX=716544 GN=ftsI PE=4 SV=1

MRDKGRRRLVAISLGVYFLFSLLIFQFFKLQIAEHEKWSETARKQHFFVVKEPFRRGTFW

SNTAIKKKHPEEPQKLVFDIQKHHLYIDPMSISEEHRDVIADEIARTLSLSADERKNIRD

AFDRRSRSRKLAMWLDQEEKDQLLNWWSPFARRRKIPSNALYFVADYKRSYPFGKLLGQV

LHTVQNQRDETTKQVVPTGGLELAFNKELRGKEGSRLLKRSPKHRFETGQVMHPSVNGQD

IHLTINHILQAIAEEEVEKGVKRCGAKKGWAAMMDPGTGAILALAQYPFFYPERYPEYFN

DPEKVGDTKVNAITDAYEPGSTMKPITVAISLQANDELRKRGEPPLFDPEEMMPTLDGRF

PGRQKVISDTRAHSYLNLNMALQKSSNIYPARLVERIIKRLGNEWYRSALTDTFGFGKPT

GIELPSESWGVVPRPGKLHPNGALEWSASTPFSLAMGYNLQATSLQILRAWAVIANGGYW

ITPHLVAGGGNLPSFRVLDQAVIDRTVEAMKYVTKTGGSGRRADIWGFTEVGKTGTAEKI

VNGRYSKKQTVASFVGFAPVKDPVFVLIVVMDEPECRFIPGVGSNLNGSIAAAPVFKEIG

RRTLEYLGIPPDDPGGYPYGDPRSDPENADWVKETRQLQEIYEKWNK

>tr|D6YWH0|D6YWH0_WADCW ATP synthase subunit b OS=Waddlia chondrophila (strain ATCC VR-1470 / WSU 86-1044) OX=716544 GN=atpF PE=3 SV=1

MNVEVGQIVAQVIAFLIILWVLQRYAWGPLLTILEERQERIRSELSAIDAEKLQVQQLRF

SYEDKLKNIDHLAQTRMQEEMEKARQITREIEKEAHQRAQEIINKAHIAAEYETNKVRSE

LKNDLIDLTIAATESVLKKELDEKHRKELIVKSIEELKL

>tr|D6YV22|D6YV22_WADCW DNA polymerase III, epsilon chain OS=Waddlia chondrophila (strain ATCC VR-1470 / WSU 86-1044) OX=716544 GN=dnaQ1 PE=3 SV=1

MPARPIFYDTETTGIKAEHDRIVEIAAYDPISGCSLEELVNPGIPIPKEASKVHHITDDM

VKDAPTFKEVGQKFIEFCSGDVILIAHNNDNFDVHFLRHEFKRHALEMPEWRFFDTLKWA

RRYRPDLPRHALQFLREIYSIEANNAHRALDDVIVLHKVFEKMSDDLDIEQSYSLLCMPR

DITVMPFGKYQGKPLQEVPEDYVKWLAKSEAFEKPENLSLKESFIKLGILK

>tr|D6YUZ3|D6YUZ3_WADCW Protein translocase subunit SecE OS=Waddlia chondrophila (strain ATCC VR-1470 / WSU 86-1044) OX=716544 GN=secE PE=3 SV=1

MIKMEKATVSAKIMDDKRSQSKPMAGSGAFNFLGEVKQEFKKISWTDKDELKVYTKVVVA

FTFIFGMSVLFVDVIIQQALAGLNGFIRLITG

>tr|D6YUZ4|D6YUZ4_WADCW Transcription termination/antitermination protein NusG OS=Waddlia chondrophila (strain ATCC VR-1470 / WSU 86-1044) OX=716544 GN=nusG PE=3 SV=1

MHNWFVVHVLSAQEKKVKKAIEEQRDLKGMTDLIDIVLLPVENISEVKKGQQKVVEKRMW

PGYLLVKMTLTDESWGYICDVPGVIGFLGGDQPTPLTPQEVDEILQDIEEKKEKVVQKHQ

FEVGDNVKIIDGVFVNFVGTVTDVQPDKGRLSVQVSIFGRETQVDDLEFVQVEEVTEESE

>tr|D6YVI0|D6YVI0_WADCW DNA primase OS=Waddlia chondrophila (strain ATCC VR-1470 / WSU 86-1044) OX=716544 GN=dnaG PE=3 SV=1

MRLFTQESLERLRSRVDLIDVLSSHMELKRSGSAYKGLCPFHDEKTPSFIVQKGERHYHC

FGCGEHGDAIKFLMTYLNLGFQESVESLAERFHVHLDCVEAKDQYTGPSKAAMKEAMEIA

SCFFHFILLHTPEGHEALHYLYSRGVCLEFIKQFRLGLAPKVSGMLHKVLHEKKIGDDVM

RGCGLLTRSDSGKTREFFSDRITFPIYHPSGSVIGFSARKYREETFGGKYVNTPETPLFK

KSRVLFGLNHCRRRIVKERRVIIVEGQIDALRLIYEGFNFVVAAQGTAFGIEHAAELAGM

GVNLIYLAFDSDDAGREAAAKVGDLFQMRGIEVRIVSLPGGTDPDTFLMEKGPEAFQKLM

EKGQGYLEFLVGHLSRGIAIDTPAGKNHLVKHVSEQLRKWEDPVMVHESLRKLARLLQVP

EEYLGVGQEYLPNTLIRKSASVGFEEVDPEKILELDFLRWLIVVGSQEFVEIAQFNLHPG

DLRHEGCRRLYQTYLTSLHNHLPSDLLSLIQTEEDQELVAELMSRKVNREKGKEQFAQSI

QRILDRNWMEKREEIRRRIHSGQLSDEEALSLLKTFDELKRSKPEVNHGTASPVL

>tr|D6YRI8|D6YRI8_WADCW Acetyl-coenzyme A carboxylase carboxyl transferase subunit alpha OS=Waddlia chondrophila (strain ATCC VR-1470 / WSU 86-1044) OX=716544 GN=accA PE=3 SV=1

MQSSPAKNLEVKLMKEMKLMKDILPHEKQIFEYIKTIDHLKKQNQNNPLFNSEIIKLEEK

LEELKEKVYGELTAWERVGICRHPQRPKTLDYIKNICDSFVELHGDRLYRDDPSIVGGLA

TINGMKCVIVGQEKGNDTESRVKRNFGMLHPEGFRKAFRLMKMAERFCLPIVSLLDTPGA

YPGLEAEERGQGWAIAHNLKEMSLIKTPIIVLVIGEGCSGGALGMGIGDVIGMLEHSYYS

VISPEGCASILWKDASKNVEASSALKLNAEYLQKFGMIDEIIPEPLGGAHHQPEVVYESV

IKFINEHYQVLKRIPSELLLEQRYLKFRNMGEFQKEELEEYDRSDEHLAAEEPA

>tr|D6YWD0|D6YWD0_WADCW 6,7-dimethyl-8-ribityllumazine synthase OS=Waddlia chondrophila (strain ATCC VR-1470 / WSU 86-1044) OX=716544 GN=ribH PE=3 SV=1

MKEIIGSFQAQGLRFGIVAAHFNEMITRKLFDGAVEALKRHGADVALSTAVWVPGSFEIP

VAAKKLAESGQYDAIITLGTIIKGSTAHFDYVAAQTASGVANVSQITGVPVIFGILTTET

IEQALERAGTKMGNKGFEAAQAAIEMANVMRELKAPVKQYAVSSS

>tr|D6YVW4|D6YVW4_WADCW Translation initiation factor IF-3 OS=Waddlia chondrophila (strain ATCC VR-1470 / WSU 86-1044) OX=716544 GN=infC PE=3 SV=1

MRINREIRAPKVRVISSTGEQVGIMSPRDALKRAEDEGLDLVEIAPNANPPVCKIIDYGK

FRYDQTKREKESKKASHQIKVKEVKVKPNINEHDLQTKMRHAKDFLEKGNKVKVTCMFRG

REMAHKSIGERLIQRIVEDLNEVAVCETPMKMFGRFLTVVLAPHKNKK

>tr|D6YTY1|D6YTY1_WADCW Glutamine-dependent NAD(+) synthetase OS=Waddlia chondrophila (strain ATCC VR-1470 / WSU 86-1044) OX=716544 GN=nadE PE=3 SV=1

MRVLIAQINPTVGDLEGNCQLILDGIAKAKSLKAHLVLFPELALSGYPPDDFLLLSDFTE

AIDRYLEKIVEASKGIAVIVGTPKMNPQGRHLKLCNSAAIIHDQTLIGLQDKSLLPTYDV

FDELRYFEPAAKTFVWQLCGKKIGVTICEDIWQHTPAARETSYHRDPVEDLAAQSPDLLI

NLSASPFRRGRSLERIETCQAIAKTLKCPVLLCNQVGGNDSLIFDGRSVAVNGDAELIDL

AKGFEEDFLLVDLSYPSEEISHDVDHTEELRKALVLGLRDYFRKQGFSKACLGISGGIDS

AVVAALAVEALGKENVLGVLMPSRFTSKRSIEDARQLVKNLGIDHREISIEEPFQCYLDL

LQPHFEGKQPDVTEENLQARVRGMILMALSNKHGYIVLSTGNKSELAMGYATLYGDTVGG

MAVISDVAKMDVYALAESYNAEEEVIPQTIIDRAPSAELREGQLDSDSLPDYEVIDTVVK

AYVEEHMCPQAIADKFDYPIDLVKDLIGRVHCNEYKRRQMAPGLRVSSRAFTIGRRFPIV

QKWV

>tr|D6YWN9|D6YWN9_WADCW UDP-3-O-acylglucosamine N-acyltransferase OS=Waddlia chondrophila (strain ATCC VR-1470 / WSU 86-1044) OX=716544 GN=lpxD PE=3 SV=1

MKGKEAFTLRELSLLTESKLFGDPDYTITGYADLENAQPHHASFLSNPKYTNTRYEGVMN

ASQAGVIFVAPHIARAQGKNYLVHEDPSRAFQTAIEALKGKIKQTGFESFHPSAVIHPTT

KIGLNTIIGPHAVIDEGVTIGKDCYIGAGAFIGPETTIGERCRIDPNVVIREHCVIGNRV

IVQSGAVIGSCGFGYTTNDQGLHERLSHIGNVILEDDVEIGANSTIDRARFTSTIIAKGT

KIDNLVVIGHNVKVGRHNIICGQSGIAGSSETGSHVVIAGQCGINGHIKLEDGVIIAAKS

GVTKSLSTGRYGGIPAQPLDQHNRTNVHIRNLEKYITQIRELNKKFK

>tr|D6YRY5|D6YRY5_WADCW DNA-directed RNA polymerase subunit alpha OS=Waddlia chondrophila (strain ATCC VR-1470 / WSU 86-1044) OX=716544 GN=rpoA PE=3 SV=1

MSVKYGRFEMPDEIVVDEETATGTFARFIAEPFERGFGHTLGNSLRRLMLTSLEAPAIIG

VRIEGVAHEYMAIDGIVEDMTNIILNIKGAKLRKLPMEDTPNARNQRLLTKVLEITQEDI

DNGKGQYEVRLGDIVEGGIFEVVNPNHHIFTVTVPMQKQVDLRVSFGRGYVPSERHVVND

KSTDEILIDAAFTPVKLVNYHVENTRVGQDTDFDRLVLEVTTDGRVTPTEAFGFASQIGL

KHFEIFKKVQTQMVSFDEIKEDSDSDDDEIMDKLALRIDEIELSVRSTNCLSGANIETIA

ELVSIPERRMLEFRNFGKKSLTEIKAKLQEMGLGLGMDLSRYGITAENVREKIKEYQALH

PKKEFTTFEE

>tr|D6YRS8|D6YRS8_WADCW DNA polymerase III subunit gamma/tau OS=Waddlia chondrophila (strain ATCC VR-1470 / WSU 86-1044) OX=716544 GN=dnaX PE=3 SV=1

MTEYQVLARKYRPQTFREVLGQDAIVATLKNGIKKRRMAHAYLFSGSRGTGKTTLARLFA

KALNCQAPSNDCEPCNACSSCKEIAAGSSLDVLEVDGASNRGIDDIRQINETVGYSTASG

RYKIYIIDEVHMLTKEAFNALLKTLEEPPEKVKFFFATTEPHKVLPTILSRCQRFNLNRI

PQDSIIGKLKRMTEEQGFQADEDALRLVANRADGGLRDAESLLDQILSFHEGGIIDSQSA

AKILGVMPQETFFRLDKAAQICNFAEAFEIAEEIFSEGKDLTHFVEMLSEHFRNILVVKL

SGADTPLLSLSENAKKQYIESAQKYRKEQCLELIDYLVKTQQEIRFAPSPRIALEAALLH

VIRTVHKIPIEVIVQRLAELENKALSIPQTASPPPVAQPAPKPKPKEAAQPKEKPKPMPA

KLIDPEKQAQYDTLLQFAAVELEGTLQKSSQR

>tr|D6YU84|D6YU84_WADCW 2-C-methyl-D-erythritol 2,4-cyclodiphosphate synthase OS=Waddlia chondrophila (strain ATCC VR-1470 / WSU 86-1044) OX=716544 GN=ispF PE=3 SV=1

MTVKSGLGQDSHRFLSTDSSKPCVIGGLIFDDAPGFNANSDGDVVLHSLCNAISSLTGEK

ILGGIAEDLCLKDGITDSEVYLKEALKTLGQQKITHVAISIEAKKPRFEEKILEMRENIA

RIMGLSISQVGITAISGEGLTDFGCGDGVQALSIITTTES

>tr|D6YVB6|D6YVB6_WADCW 50S ribosomal protein L28 OS=Waddlia chondrophila (strain ATCC VR-1470 / WSU 86-1044) OX=716544 GN=rpmB PE=3 SV=1

MPKCQVTGKKTTRGNKYSIRGIAKKQKGIGLNITGKTRRTFKPNIIEKKLWFPEEKRFIT

LKLSTSALRTIDKVGVGPIVRKLRAQGHKI

>tr|D6YS30|D6YS30_WADCW Histidine--tRNA ligase OS=Waddlia chondrophila (strain ATCC VR-1470 / WSU 86-1044) OX=716544 GN=hisS PE=3 SV=1

MNIKAPPGVFDILPIDPKTPWKESHLWIYVEKVIRETAAQYGFQEIRTPILEKTELFQRG

VGETSDIVSKEMYMFNDRGGRSLCLRPEGTAPVIRSYIENNLSHQVPIHKFFYIGPMFRY

ERAQAGRYRQHHQFGAEVIGTQAPEQDAEVIDLLYTTYRKLGLQNLKVMINSLGDKECRL

HFRHALKEYFSQHKKHLSEESRQRLDKNPLRILDSKSPQDQEFIEKAPSILDFLSHEVKT

HFERVQEYLTFLEIPFAINPHLVRGLDYYNRTVFEVAAEELGAQNSIGGGGRYDSLIKEL

GGPDQPALGFGTGIERIIQTLLKQEAPVPSAYRPTFFLIPLGEKAKRACFKVLHFLRENS

IDAQMDFTGRKLGKVMGYANQIGAKFVAVVGDQELETETVELKNMETGEAISAPLYHLSR

ILRIESAGEDLIRMWADFNTPFKEPLEAKFFHDKIKSSIDHTKKLTKELQDAMEKMESFL

>tr|D6YS54|D6YS54_WADCW 2-dehydro-3-deoxyphosphooctonate aldolase OS=Waddlia chondrophila (strain ATCC VR-1470 / WSU 86-1044) OX=716544 GN=kdsA PE=3 SV=1

MTVMSGPCVIESEEHCLKAAEELKKMFSGRPINFIFKSSYDKANRSSIHSFRGPGLEEGL

RILEKVKKELDLPVVTDVHSPEQATAAGEVVDMIQIPAFLCRQTDLLVAAAKTGKVVSVK

KGQFLAPWDMRNVIEKIKESGNDRVIAVDRGTSFGYNNLVSDFRAIPIMQELGVPVCFDA

THSVQLPGGMGDKSGGQREFIPYLARAAVAVGADCLFMEAHPDPDQAKSDAASVLRFDAF

PKLLDLLQRLRDAVDVL

>tr|D6YS46|D6YS46_WADCW Phenylalanine--tRNA ligase beta subunit OS=Waddlia chondrophila (strain ATCC VR-1470 / WSU 86-1044) OX=716544 GN=pheT PE=3 SV=1

MKLPLSWILEVIDLNLPSHKIANMLTMAGLEVDGCTPLPLAFENVVVGEVLRTERHPDAE

KLCIATVFDGEEQFQVVCGAPNCRPGLKTAFAKIGSVLRDEEGKAFKIKKTKLRGVESYG

MLCSEDELGIGDHGSGIMEFADRIQVGADISEIYGDTVLEISLTPNLGHCASVLGVVREL

AAMTGKQYRLPAIKIEENEEEAFEAVSVEVACFEKCPRYACRVIKGVKVGPSPDWLQKKL

LACDQRPVNNVVDATNYVLMEMGHPLHAFDYDLLEERRIVVRTAQEGEAFVSLDEKRRVL

TSDDLLICDGKKPVAIAGVMGGLNSEVHEGTVNILLESAYFEAAGIRRTSKRLALQTEAS

RRFEREADPNQVLAALDRAAVLIKEISGGEICRGVVDVKQKDFSPKEVKCRLSRVNAVLG

TQLGMSEVNDLFTRLEFGVKWDGKDTFTLTIPTYRADINSEVDLIEEVARIYGFDNIPKP

SSYFTSSDMPHAPIFVFEREIRRRLMAEGLQELLTCDLIGPKLQEAVNGPIMPPENIVKV

LNPVSVEQSILRTSLLPGLLQVAKHNYDHECRNLSGFEIGRIHFKEEGRFKEQSMAGVLL

MGNRHPDHWERKKDGADFFDLKGIVESLLLETGVLDYQFRSGNLHFFHPGRQGSLCIGEQ

EVGILGEIHPTILRRMDLSNKVYYAEIDLHLLYQMRQSRRLMEPMPVYPASDRDLTVTVE

ENVAIQEILEVISSVKSRLLENVSLIDIYRGEKVGESKKNVTLRFIYRNVKKTVSQEAVD

AEHARITSQIEARIRRY

>tr|D6YUG6|D6YUG6_WADCW Phosphate acyltransferase OS=Waddlia chondrophila (strain ATCC VR-1470 / WSU 86-1044) OX=716544 GN=plsX PE=3 SV=1

MGSESSPGVLFEAVLASASYLSKQDSLVVFLTRFAMESILRDSRFSCFLDEHSGLVDLHV

VEGEISMEDDPLAAVRLKKESSVVVGLRLLKERNIDAFVSAGNTGAIVAAAALILPKMGL

DRCCLLASLPTKKDPLAVLDVGGFLDPTPEILLQYARIGAFYQRALLGKETPNVGLMNIG

VESKKGTGEVREAYRLLKQEEGGQFHFVGNIEGREAFEGGVDVLVTDGFTGNVLLKVSQG

VSSFIFDYLKSEIKEILNDEVHQVIDDLHRHFRYDEHPGAFLCGVEGLVIKCHGAATVRS

MVQSILGAKRLVQINIINQIKRNFY

>tr|D6YUG5|D6YUG5_WADCW 50S ribosomal protein L32 OS=Waddlia chondrophila (strain ATCC VR-1470 / WSU 86-1044) OX=716544 GN=rpmF PE=3 SV=1

MAVPRSRLSNQRKNTRRAHHAKKPRSLIDCSKCGVKRLPHTICQSCGWYGDRSVLGGKEG

SQE

>tr|D6YW86|D6YW86_WADCW Endoribonuclease YbeY OS=Waddlia chondrophila (strain ATCC VR-1470 / WSU 86-1044) OX=716544 GN=ybeY PE=3 SV=1

MDVHIFDQQNDLSISVLQVEKLVIECLKLEGVECDEVSIQFVDTQMICDLHHRFFNDPTV

TDCISFPIDQDGDTGYRVLGEVFVCPKTGIDYAQEHLKNPYEEISLYIIHGLLHLMGYDD

IGEDEPRMREAEKRHLAHLSASGLLLKETTCTS

>tr|D6YSF0|D6YSF0_WADCW 50S ribosomal protein L27 OS=Waddlia chondrophila (strain ATCC VR-1470 / WSU 86-1044) OX=716544 GN=rpmA PE=3 SV=1

MAHKKGQGSSRNGRDSISKRLGVKRTHGEVIKAGTIIVRQRGTKWHPGTNVKRGNDDTLF

ALCDGVVDFRKTNKTVVSVKA

>tr|D6YUZ9|D6YUZ9_WADCW DNA-directed RNA polymerase subunit beta OS=Waddlia chondrophila (strain ATCC VR-1470 / WSU 86-1044) OX=716544 GN=rpoB PE=3 SV=1

MTQRPPNRVSFKESKEIIDLPNLIEIQIKSFNQFLQIDKYPEERDNIGLQEVYNEIFPIK

SYDEKTIIEFLSYSLGVPKYPPDECIRRGISYNVTLKVKFRLTDETGIKEEEVYMGTIPI

MTDKGTFIINGAERVVVSQLHRSPGICFEQERHPRGNILYSFRIIPYRGSWLEGAFDTND

MIHIYIDRKKRRRKILATTFIRSLGYSTNADIIEEFFSTRKMKIKSEKDFEKLVGKILAE

DVLDEESGLIFGKAGEKLTTAMLKRMADSDISMVRIAEDADETSPIIKMLAKDPTDSYET

ALKDFYRKIRPGEPATLSNARSAMMRLFFDPKRYNLGRVGRYKLNSKLGCEVNDEVLEVV

TLSKDDVINAVKYLIRLRSGDENAYIDDIDHLGNRRVRSVGELIQNQCRIGLARMEKIIR

ERMNLFDFTSDTLTPGKIVSAKGLSGVLKDFFGRSQLSQFMDQTNPVAELTHKRRLSSLG

PGGLNRDRAGFEVRDVHTSHYGRICPIETPEGPNIGLITSLSSFAKINEFGFIETPYRIV

RDGVVTEEIEYMTADQEENCVIAQASVSLDEFNMFEDEICWARYRGEPLEIESSRVTHMD

VSPKQLVSIVTGLIPFLEHDDANRALMGSNMQRQGVPLLAPEAPIVGTGLEARAARDSGA

VVIAEEDGTVQYIDGNKIVIAPKKNPMEKKTYLLKKFMRSNSGSCINQKPLCKVGEKIKS

GDVIADGPATDNGEIALGRNVLVAFMPWMGYNYEDAIIISEKLLREDAYTSIYVEEFELT

ARDTKLGKEEITRDIPNVSEDALINLGDDGIIRIGAEVKPGDILVGKITPKSETELAPEE

RLLRAIFGEKAADVKDASLVVPPGTEGVVMDVKVFSRRDRLSKTDEELVEEASRLKDLQR

EYKQKRSELKMERHERLGALLLNDKAPGTIVHRKTAEIVLEEGVLLTQDVIETFEDYNVD

DLIMPENQVYQTLKQVLHDYDVKLQTLETHFKTQLEHLKKGDTDLEAGVIRQVKVYVASK

RKLQVGDKMAGRHGNKGVVSNIVPEADMPFLESGQTVEMILNPLGVPSRMNMGQLFETHL

GFAAKLKGIKIKTPVFEGFPEEEIWKMMKEQGLPENGKFFLQDGRTGNRFDNTVVVGYIY

MLKLSHLVADKIHARAVGPYSLVTQQPLGGKAQMGGQRFGEMEVWAAEAYGAAHLLQELL

TVKSDDVTGRTRIYESIVKGENVLRSGTPESFNVLIKEMQGLCLDVRTESVEESEF

>tr|D6YS01|D6YS01_WADCW 50S ribosomal protein L22 OS=Waddlia chondrophila (strain ATCC VR-1470 / WSU 86-1044) OX=716544 GN=rplV PE=3 SV=1

MAENNAKAITKYVRISPRKARLAADLIRGIPVEDASLQLQYSNMKAGRLLKKTLDSAVAN

AETQLEARRENLIVSEVRVDEGPTFKRAKPRNRGGRHPILKRTSHFTVVVSAGEGA

>tr|D6YS73|D6YS73_WADCW 30S ribosomal protein S4 OS=Waddlia chondrophila (strain ATCC VR-1470 / WSU 86-1044) OX=716544 GN=rpsD PE=3 SV=1

MARYTGNRNRIARRFGVNIFGRARNPLIHKPNPPGVHGARRRKKSDYGLQLEEKQKLKAV

YGMISEKQLVNYYKKALKLKGNTPLHLAEMLECRLDNVVYRLKLASTIFAAHQLVSHGHI

LVDGKKVDIRSFQVKPGMVVSVKERSQKMKAVNEALDNSMKDVPEYFSLDRNKFSGQLLS

MPTLEQIPWPLEINLPEVCDFLDHTN

>tr|D6YRP1|D6YRP1_WADCW UDP-N-acetylmuramate--L-alanine ligase OS=Waddlia chondrophila (strain ATCC VR-1470 / WSU 86-1044) OX=716544 GN=murC PE=3 SV=1

MKRAFHFIGIGGIGMSSLARILLEKGEVVSGSDLAATSMTESLCSLGAKISLGQKAENIS

PAQTVVFSTDIKESNPEFQAAVKLRCEMQHRCGCLLNLMKNKDVLAVGGTHGKTTSSSLL

AWVLEVCGFSPSFAIGGIVANFKANGKAGEGSCFVAEADESDGTLARYRSFGAIVTNIGL

DHMDHHQTEERLLNCFRTFFSKVQNEAFCFWCGDDKRLQTLNPKGVSYGFHENNRLRVDH

FRQVGWTSIFDVVFDGKRFADIEVLLAGEHHALNAAAVFGLSLRLGAQESKIREALKTFK

GVARRCEVKGEKNGILFLDDYAHHPTEIRATLKAIRQASPDRRIVAVFQPHRYTRTRDCM

GMYGSTFHSADQVIVTDIHAAGESPIPGVEVCHILSELEKNSSVKCRYLPREDLSCKLSK

FLEKGDVVVTLGAGNITKVAVETLEAF

>tr|D6YSF1|D6YSF1_WADCW 50S ribosomal protein L21 OS=Waddlia chondrophila (strain ATCC VR-1470 / WSU 86-1044) OX=716544 GN=rplU PE=3 SV=1

MYAIIKSGGKQYRVAKDDVIDVELLHVDQGSAVEFSEVLFVNDGTEALIGEPVVAGYLVK

GEVVGTSAGPKVSSLKYRPRGHTQKHWGHRQHYTRVKITDIASSGKVEEKKAAPKKAVPK

KKKEEDK

>tr|D6YT75|D6YT75_WADCW Uridylate kinase OS=Waddlia chondrophila (strain ATCC VR-1470 / WSU 86-1044) OX=716544 GN=pyrH PE=3 SV=1

MGSQHFGISQEACEQLAKSIDKMRNSGLQVAVVIGGGNIFRGIHLENLGLERTPADQMGM

LATMINGIALQQALGQINCKAIVMSSLSCPGLVDNFDLRRAIDHLENENVIIFVGGTGNP

YFTTDTAAALRASEIQADIILKATKVDGVYDKDPMIHEDAKKFETLTYSKALSDNLKVMD

ATAIAMCRNSNIPIFVFNMKHLTEDKILSALSQEGLGTLVKGEM

>tr|D6YVR0|D6YVR0_WADCW Riboflavin biosynthesis protein OS=Waddlia chondrophila (strain ATCC VR-1470 / WSU 86-1044) OX=716544 GN=ribF PE=3 SV=1

MIGIYQKIEDFKPLDVPVVLTIGNFDGVHRGHHRVIEQSVSYARLIGGKSVVLTFSNHPS

EVLPNRTPVLPICTPLHKKQLLEKLSVDFLIQIPFSLEFSKQSAETFIQSIRKFIPFSHL

VLGYDARFGNNRQGDQEAIQQQAKIFGFTTDYIDCVDINGITVSSSNIRIAVKNGNFTEA

SKLLERPYSILSTVIKGEQIGTQIGFPTANLDVANLCLPPFGVYAVHLKCDQATLPGVAN

LGVAPTLKNTRLPTFEVFMLDGILNLLGKTVELIPLHFLRTEVKFSSLKQLKQQISEDIK

TAKIFFKHSKKDKDELLG

>tr|D6YT06|D6YT06_WADCW Small ribosomal subunit biogenesis GTPase RsgA OS=Waddlia chondrophila (strain ATCC VR-1470 / WSU 86-1044) OX=716544 GN=rsgA PE=3 SV=1

MCYHLNMEDQENPTVSKRWIACPIEEEYLGSDRKQKRKERKEKSSRDRSKFKKTDKSKYT

DSLQKEIKERVVDQELFFGRVLSVTSQGIVVDCDREIYTCMIRGLLKREKTQTKTLVAVG

DIVRFAKMPDNEGMIVDVEPRKSILSRADNLSRKKEHLIAVNIDQVLITSSVVIPPLKPA

LIDRYIIAACKGGMRPVIIVNKVDLLDSHDFDDEARKEQRDLLEKCRQAYKEVGISFFEV

SVESRCGLDELKEAMRDQTSVFSGQSGTGKSSLINELTGLNLPVGDTVKQTRKGAHTTTQ

ARLVPLDCGGFCVDTPGIKSFGLWDLAQEEIQQYFPEIEKISSFCRFPNCTHTHEEKCQV

RKDVEDGNISPLRFESYLSLIESVVQKHHRR

>tr|D6YU12|D6YU12_WADCW Acetyl-coenzyme A carboxylase carboxyl transferase subunit beta OS=Waddlia chondrophila (strain ATCC VR-1470 / WSU 86-1044) OX=716544 GN=accD PE=3 SV=1

MRFFSRDKPKIKVQSSKKDGFSGWLKCTHCTELIHTNELEKNLNCCPKCSYHYRLTAKQR

IEALADSDSFQEMFQEIKTADPLQFIDSEKYPERIEFARRKTDIDEAVIVGTCKIEGIEA

ALGVMDFHFMGGSMGSVVGERLTRMIEHAICHRLPVVIVASSGGARMQESTLSLMQMAKT

SSALARLSEEGLPYISILTNPTTGGVTASFASLGDIIIAEPKALICFAGPRVIEQTIGQQ

LPEGAQKSEFLLKHGMIDCIVNRHDLKNKLAECLFYLIPADN

>tr|D6YVH2|D6YVH2_WADCW 4-hydroxy-tetrahydrodipicolinate reductase OS=Waddlia chondrophila (strain ATCC VR-1470 / WSU 86-1044) OX=716544 GN=dapB PE=3 SV=1

MICGMLKIALIGYGKMGKMVEQIAVKRSHEIVAIIDPKTEDRETVQNSLKKADVCIEFTQ

PNAVLGNIRTALSLGKNIVVGTTGWNDALKNVEKWVAESGTGLFYASNFSLGVNLFLKTV

EKAADLFLNTGNYNVAGVEIHHQQKLDSPSGTAIAIQQTIARTGGKSPSFSSVRCGSVPG

THTVYFDSPSDTITLTHQARNREDFALGAVSAAEWMQGKTGIYTMEDMLCLA

>tr|D6YVM6|D6YVM6_WADCW Bifunctional protein hldE OS=Waddlia chondrophila (strain ATCC VR-1470 / WSU 86-1044) OX=716544 GN=hldE PE=4 SV=1

MRAVGGKCDRLRKKLPSNRKKMVNLFSSFRRLSKQSVMVIGDLMLDTYTIGKASRISPEA

PVPVLHVQAEEHRPGGAGNVALNLAAMGAETLMVGRVGADDHGKILIDSLKREGVQTHGI

WIQTGCQTPVKNRVIADNQQMVRVDYETALPLHEAIEQQIIEALPTLLESIKVVAISDYG

KGMITKTLMAAVIEECLQRNIPIIADPKGIDFTKYRKATVVKPNLGEVYAAANLPMDAPL

EAAAQRVLEITQAEVLMVTRSEEGISLFYQSGKREDFPVRAREVVDVTGAGDTVLAMLTV

ALANQLTVSDATRLSNVAAGVAVSHFGCARVTLAELARQLLSEDVGNKVFDEEHLFALQE

ALKGREFAILSLSSKGGIGTDLFEAIRKVAQKKSWDLVVHIEDENPSPIFVDVIASLHDV

DYIIVKRGGLEYVSKLLFADEIFSFS

>tr|D6YUH1|D6YUH1_WADCW Lipid-A-disaccharide synthase OS=Waddlia chondrophila (strain ATCC VR-1470 / WSU 86-1044) OX=716544 GN=lpxB PE=3 SV=1

MSHSLFLIAGEKSGDMLGCNLMKALKEQMPGTAFAGVGGQEMRQEGLDCVLRTEDFELHG

FSDIIRSVPKLIKQFKTIRNWILSKNPDAVIFIDYPGFNLRMAKSLRKKGYRGKLVQYVC

PTIWAWGRKRKQKMEEALDLVLSIYPFEPAYFENSPLKVEYVGNPVKKIVQNHKHDENWH

ALFGIKKMDHLIAIFPGSRKGEIQRNLPYQLKTCELMKKKNPNLVFAISCAHEKIMPVMH

PMLRNVSLKLHQDLFLLPKTYSYELMRDCRSALAKSGTVTLELALHQTPTVVLYKLTWLN

RFIAKYLLRLNLPHYCIVNILSNQTVYPEVIEKGLSAQNLYKKLMPLNGHTEERRQCIEK

CQELDQLLQNKDASRQAALAVRELIG

>tr|D6YV85|D6YV85_WADCW Alanine racemase OS=Waddlia chondrophila (strain ATCC VR-1470 / WSU 86-1044) OX=716544 GN=alr PE=3 SV=1

MDAFDLRNWSGFISAGGDSNRPALIDQIVIDTRRIYASNALFAALKGQRQDGHSFVSGSG

AKFALVEKHWEGPADGPSLLKVDDPLTAFQEIAGSYRSQLRGNVIAVIGSYGKTMVKDLL

RHLLSSSFTVASSPESFNSQVGVPLSLLGAAKAHEQVIIEAGFSHPGEMARLCKIIRPSH

IILTHVGNKHLHTIGSKEQIAQEMCHFSLSSDASWTLLPKDPILSPLPKPYYWDTPQPNL

PHASFHENSKAITVQFPKGDAHTILRPLGMSYILDLINIGLKAAYLLGVPEKQIVDALKT

YQPEPMSTEIWKSQNGATFINETYCEDPQSLDCALRRFDFHSPKGKKTLLFSGIKQNDPA

LDARIGEAIGNSMPDTLMLIEPSFQLEHAVKTIAPQTAIRHFSTVEAALKDHAGSMRPED

ILIVKGKKKLPFDQLTQTVQGSIFSNQCRINLAAIASNIKQIRSRLTPHTEIMAMVKATA

YGTQDALMAKFLKTEGIRRLGVSYIDEGISLRKEGVDQDIFVLNAAPYEIEKAVSWQFEI

AVNDKETIDQIGRMSSERGTITRVHLHINTGMSRLGCRPEEALPLARIIQAHPHLKLEGV

MTHFACSDNPDEDTFTLEQSRIFDHSIEAIEQEGISIPFRHACNSAAALRLHFPQYNIVR

IGMALYGLCPGGTLALSLHSRIVGINHCIKGETISYGRSYTVQREREKIAVIPIGYYDGL

HLNYSSKGKVMIHGAEAPIVGRICMDYMMVDITHIPDACIGDPVLIFGEDAEGNVLSPNE

LAEQGNSSVYELISCLGPRIQRVFIHEENDKIM

>tr|D6YWX3|D6YWX3_WADCW Multifunctional fusion protein OS=Waddlia chondrophila (strain ATCC VR-1470 / WSU 86-1044) OX=716544 GN=glyQ PE=3 SV=1

MITFQEIIRRLSAYWEEKGCIIHQGYDLEMGAGTMNPATFLRCLGPEPYRAAYVEPCRRP

TDGRYGENPNRVQHYFQYQVILKPSPSDMQQQYLDSLKAIGVDLSQHDIRFVHDDWENPT

IGAWGLGWEVWMDGMEITQYTYFQSVGGLSLKPVTGELTYGLERIAMYLQNVDNMFDLKW

NEHLTYGDIYHRNEVEFSRYNFEEATTEMWLTNFNFYEKEAKKLIGKKLPLPAYDFVIKA

SHAFNILDARGAISVTERTQYIGRIRDLAKMIAESYVENRKLQEFPLLEKFPSIKREMGS

LPPMNEALLKMPEGTKDHFLLEIGSEELPAAFVPIGLRNLESAIIELLNKENLSYEKLNV

YGTPRRLSVVVENLQLTISEAVEERRGPPLDRAFNEDGTLKPAGAGFLKSIGKELLDRTA

IENDEEIEIREVKGSAYLFAKVKTPGKSAARILADSLPSIILNLEFPKKMKWGDLEISYA

RPLQWIVAMIGTEVVPFIVGDLISGNASRGHRQLDPDPFEILNPQSYLETLREHRILADP

AERKQEIIKQLNTLENQLGLHVIERDAVIPQVLNLVEWPMVAKGNFDENFLKIPQEVLIS

EMVEHQKYFPVADENGKLKPQFVITCNTLPTENMIRGNQKVLSARLSDGVFLYEKDLGVP

FESFNEKLKAVTYLSGLGSIYEKVLRLEGHVEVLQEHLQISSLKNVHRAARLCKCDLVSD

MVFEFPELQGTIGRYYALAHGEKPEVAQAIEEHWMPKGENAPLPTSETGSVLCLAEKIDN

LLSCFSAGLKPTSSKDPYALRRQVLSIIRLVIAKKFRLPLKNILDKCTRNFDPMHLSSKD

QVIKEVMDFFTNRIKSIFQEYGFTKDEVEASIACGFDDIYDTFCRVQALHEFRASSEEFP

KLYEVYKRAKGQLANHSAVAFDKGLLLKDAEKELDKTLDSTKEKFEEALSHQHYAQAYQL

IAQMQPSLARLFNEVRILDEDEKIKENRIALLQRVFSLFEQLLDFSKIQD

>tr|D6YRN7|D6YRN7_WADCW Putative phosphoesterase, RecJ-like protein OS=Waddlia chondrophila (strain ATCC VR-1470 / WSU 86-1044) OX=716544 GN=wcw_1381 PE=4 SV=1

MWRETKKIFQEHGSFLISTHVNPDGDGIGSACALADLLSRMGKQVCFVYDGLFPEKFSFL

NFPGIKEIFYSEENYSDIEVVVMVDAHSADRLGRVAQIFQRPDVVKVVIDHHQPQEIPAG

HCVIDSNASSTGSMIYTLYKESGYELTKEAAEGLYVSVISDTGRFCYSSTDRKAHKIAEE

CMKKGVDPDWMYAHLYQQVSLPEFKVFAKALQHMEEHFNNRVIVQKILREDFVGVSDAVQ

EILQSDLEYFHEFNKIIVGVDCVMLLCERPNRTVRVSLRSSGSFRVDRIASQFGGGGHPK

AAGASLKGTVEGVKEKVLEKIQQELERLD

>tr|D6YUB6|D6YUB6_WADCW UDP-N-acetylmuramoyl-L-alanyl-D-glutamate--2,6-diaminopimelate ligase OS=Waddlia chondrophila (strain ATCC VR-1470 / WSU 86-1044) OX=716544 GN=murE PE=3 SV=1

MKLKRLIKDLPNAEIKGSKEIEITGICAHSKQVAPGNLFIAKKGRTFDGSQYIAEAVETG

AAAVLNDIFDPSLKVSQIIHPNVAVVEGKLVSAFYHRPGEELLMVGVTGTNGKTTVSYLI

KHLFDSLEMRPGLIGTIEYLIGDHAAPATHTTPDVATNHKLLREMVNQRSQAAVMEVTSH

ALHQGRVSEISFDVGIFTNLSQDHLDYHKTMDAYCEEKRKLFLSLDPEAKKRKNYPKAAV

VNIDDPWFEKIIDGCRVPIITYGIANHADLRAEEIQLGAEGIVYTLVHQGKRYPVSLPLI

GRFNVLNSLATIGCGISLGVPLNKVLDAVKSFPPVPGRLEYVPNKRGLKLFVDFAHTPDA

LKNVLNCLKELTQGKLIVVFGCGGDRDPYKRSQMGRIAEEFSDLAIITSDNPRSENPANI

MDQIVAGFNDPSCFIKIEDRRKAIEEAIHRATPEDVVLIAGRGHEPYQIFAHQTIEFKDR

EVVEDILHS

>tr|D6YW29|D6YW29_WADCW Putative DNA polymerase III delta subunit OS=Waddlia chondrophila (strain ATCC VR-1470 / WSU 86-1044) OX=716544 GN=wcw_0980 PE=4 SV=1

MKFGNRKAFDKHLKEAGPHHFASVYLLISADYFERKQLVTKLCGAVLNGSPLDEHHLKKL

DGAVHSVHELMRELGSFNFFAKKRVVIYENAGELKKEDAGVLKSYYEHPNPSVFLVLSSS

SVNRGSSFYKQTEKAGVILDIEPEKPWEKEAALSDYAAAWFKKNGKKTASGVNQALVKSV

NNDQAQLLQEMEKILCYLGENEEVSFEDLRVIGSGQTSYTVWQLGDAVFSRKGKEALEIS

NQLLGDGESLIGLLRALRMQFQTRLLGATAQPEEVQSRVPYLRGPLLHKQIQQALQYGAE

SCREGVLLIDEAERLAKNSGIADRCILETLMIKLAVL

>tr|D6YS00|D6YS00_WADCW 30S ribosomal protein S3 OS=Waddlia chondrophila (strain ATCC VR-1470 / WSU 86-1044) OX=716544 GN=rpsC PE=3 SV=1

MGQKVNPIGFRLVRKKDWRSKWFANKQEFGDFLVEDRRIREYLLKKSQLVGTSKIKIRRM

SEKIEVTIVTARPGLVIGKKGAEIDALKTELYKLTGKQVWVEVEEIKRPDADAQVVADGI

AKQLERRIPFRRALKKAIQSSLDAGAVGIKVQTSGRLGGAEIARTEWYKEGSIPLHTLRA

NIDYATSRAETTYGTIGVKVWINRGDDNYSKKENG

>tr|D6YVM2|D6YVM2_WADCW 30S ribosomal protein S6 OS=Waddlia chondrophila (strain ATCC VR-1470 / WSU 86-1044) OX=716544 GN=rpsF PE=3 SV=1

MGENKQNLYEGMYIISATLSEDARSKALERVQNEITSRGGEIHKVHEQGKKRMAYEIDGH

REGHYYVIYFSVSPAAISEVWNEYHLHEDLVRFITLRTEKVLEKIEFKALAESQ

>tr|D6YTZ2|D6YTZ2_WADCW Elongation factor P OS=Waddlia chondrophila (strain ATCC VR-1470 / WSU 86-1044) OX=716544 GN=efp PE=3 SV=1

MPQVSTSEFKSGMKIEVEGQPYVIVNNEFVKPGKGQAFNRVKLKHLLTARTIERTFKSGD

KVDLADVLETEMRMLYRDADGIVFMDDKTFEQITIPLDRIGDSDPWMMEDILYEVLIYNG

EPVSVEPPTFMELKITQTDPGERGDTASGKVLKPAETESGAKVQIPIFIEEGEVVKIDTR

TGEYVSRVN

>tr|D6YS27|D6YS27_WADCW DNA-directed DNA polymerase OS=Waddlia chondrophila (strain ATCC VR-1470 / WSU 86-1044) OX=716544 GN=dnaE PE=4 SV=1

MFVPLHVHSQYSILDASASVKAIAAKAKEFGMPAVALTDHGNLFGAVDFYKSCKGNGVKP

IIGCEVYIAPNSRLEKKKERGERAAFHQTLLAKNDEGYHNLCALSSRGFLEGFYYFPRID

MELLKERSRGLICLSGCMSSKLSQAILDGAGDECDRLADWYLEVFGEDFYIELQRHPMSE

ASLHEGGMYQESWLIQQYQGYIEKQNRLNDALLEIAERKGILYVATNDSHYIERSDWQAH

EVLLNVQSGEPVEIWEKDSLGNPKFRVPNPKRRTYSSHEFYFKSPDEMGELFKDLPEALS

NTLAIADKCTMELDFETKHYPVYVPPSIENTEYTDAERVESVEKFLRQLCAEGIPKRYTP

ERLEKVKEVYPDRDPLEVVHERLEEEMSVIAPKGMCDYLLIVWDFINWAKNHGIPVGPGR

GSGAGSIVLYLIGITDIEPLRFNLFFERFINPERLSYPDIDVDICMDRRGDVIQYTLDKY

GKDNVAQIITFGTMKAKMVLRDVGRVLSVPLAKVNEIAKLVPDDLNITLETALEKDHDLR

QLYEQDEETRRLVDLGKKLEGSIRNTGIHAAGIIICGDPLVNHIPVCVAKDAEMPATQYS

MKPAEQVGMLKVDFLGLKTLTAIQTCVNAIKERVGEQIDWVNLPLDNEKAFNLLNQGKTL

GVFQLESGGMQDLARQLHLDKFEEIIAVSALYRPGPMDMIPSFINRKHGREEIDYDHPWM

KEILAETYGIMVYQEQVMQIAQKLANYSLGEGDVLRRAMGKKDAEQMAAQREKFRLGALE

NGISENVSMNIFDKMEKFAAYGFNKSHAAAYSYIAYVTAFLKANYPEEWMAALMTCDRHD

LTKLSKFIRECQAMQIKMLPPDVNEAGTTFMASKEGIRFAMSGIKGVGTSVVEAIVAERE

KGGRFESLYQFIKRIDSSKVGKKVIENLVEAGCFDFTGWSRDQLRMSVEPMFESAMKEKK

EEKAGVRSLFELMGENNENLFAEPPKVNMPTPSRQIYLKEKELLGFFLTGHPMNSFKDIL

ERLSCIALRNVETLDHQTVFRSAFLVETVRTLFSSKSQRKFAILMVSDGIESFELPIWPE

MYEEKHQLIKENQLLYAVLEVDRSEGSLRLSCRWFDDLTQANEATVKECDQAFDRAKFMA

ARASHSKNRRSRSNQKKKEEPKVEIEKVSLKLDADKTRLSHILEIKKLFESSRGKNPVQI

DFMVDGRSLAALHIDQPWGVEWTPELKEKLEKFSWR

>tr|D6YWH5|D6YWH5_WADCW ATP synthase epsilon chain OS=Waddlia chondrophila (strain ATCC VR-1470 / WSU 86-1044) OX=716544 GN=atpC PE=3 SV=1

MFSLKILTIDKKVYEGKALSLTIPGTLGYFEVLNNHASLMTPLQPGKLAITFSQQEKSVF

AISGGIMEMHQNQVTILGDTIESAGEIDYDRAKAAYQKAYKLLEFPEEEIDQHEVTQALL

RAKNRMEIAASSNS

>tr|D6YV49|D6YV49_WADCW Enoyl-[acyl-carrier-protein] reductase [NADH] OS=Waddlia chondrophila (strain ATCC VR-1470 / WSU 86-1044) OX=716544 GN=fabI PE=3 SV=1

MLSINLKGKRAFVAGIGDDQGYGWAIAKALAEAGAEILIGTWTPLMKIFLTSLEKGKFDA

SRRLSDGSLMQISKVYPLDASFDSPEDVPEEVKENKRYKDAEGYTIAEVAEAINNDFGSI

DILIHSLANGPEVHKPLLETSRNGYLAALSSSSYSYISLLQHLGPIMNAGGSAISLTYFA

SERAVPGYGGGMSSAKAALESDTRILSWEAGRKWKIRVNTISAGPLRSRAAKAIGFIDGM

IKYACANAPLTDEMTSEDVGNTAAFLSSPLAGAITGTTVYVDKGMHAMGVAMDSQALN

>tr|D6YWB1|D6YWB1_WADCW Riboflavin biosynthesis protein RibD OS=Waddlia chondrophila (strain ATCC VR-1470 / WSU 86-1044) OX=716544 GN=ribD PE=3 SV=1

MDDRRWMEMAIAEGLKGRLDAPPNPWVGCILVKNGQIVGLGHHPECGLPHAEVYALREAG

QNARGATAYVTLEPCVHTGRTLPCIHALIEAGVARVVVGVEDPDPKVSGSGIAALKIAGI

KVTVGVEREAVELSLAPYLHHRRTGRPFVIAKTALSIDGRAAASDKTSQWISCEAARRDA

HEMRAESQAILIGAGTAVKDSPQLTVRQVNKLPKKQPLRVLLDPNGKVPHDSPLYDLDLA

PTLVFSEKMEPEKILSILGEKGVIQLLIEGGPTTITQFLQQHLIDRLIVYTGPCLLGSDG

LPGFGSMGICSMENALRLHLLESSAIGNSVKSVYAHTFQ

>tr|D6YTK1|D6YTK1_WADCW FeS assembly protein SufD OS=Waddlia chondrophila (strain ATCC VR-1470 / WSU 86-1044) OX=716544 GN=sufD PE=3 SV=1

MTLFEEQLKRHYQLLPGDAALETIRQKAWDRFQAVGLPQKNQEVWRSIKLRKLFEKSLTL

PSLGTRSREEIETLILPGFKKRCLVMINGQLDLELSSTEDLGGRLIVSHLERAAKSYGGF

LKPAWMQAIKEETDPFAVLNSALGAKGAFVYLPPNAIIEKPVQILSVHSSETSSLVQPRL

HLFAGKGSRITLVSTPHVFSSGSVCNASFELSIDENADVSLYQLNHRMPEAHWQLETTRS

FLKRDSRFTAVNLTNGSETVRSDYKAVLTGENGEVNLSGLWMLQGNREAHTNILIDHQAP

HCRSNQLFKGVLDDASRSSFEGKIYVRQAAQKTDAFQLNNNLLLSEKANAFSKPNLEIFA

DDVKASHGATVGQLDKEQLFYLKSRGYSQEMAQKTMIKGFYLEIIDQIKDSVVRDEITKG

W

>tr|D6YT87|D6YT87_WADCW RNA polymerase sigma factor SigA OS=Waddlia chondrophila (strain ATCC VR-1470 / WSU 86-1044) OX=716544 GN=rpoD PE=3 SV=1

MKNNSTPKEAISQQHQQKIDELVAIAKDQGFITYEEINDVLPMTIDTADQIDQILIFLSG

MDVQILNQSEVERQKEKKKEAKELEGLPRRVEGTPDDPVRMYLKEMGSVPLLTREEEVEI

SKRIEKAQHQIERIIMRFRNSSRETIAICSALINNKERFDKCITEKEIENKTEFLKLLPR

LSDLLQKDDQILESHLIELENPKLTKAERAYYLEEIEKARIRTQAYLRRLHLRHNIIDDF

GEVIMESYDRFLQLEEEILELTPRAERNKYAGLKLAAAKRKLKKKELAAGRSLEEYKKDV

RMLQRWMDKSQEAKREMVESNLRLVISIAKKYTNRGLSFLDLIQEGNMGLMKAVEKFEYR

RGYKFSTYATWWIRQAVTRAIADQARTIRIPVHMIETINKVLRGAKKLMMETGREPTPEE

LANELGLSAERIREIYKIAQHPISLQAEVGDGGESQFGDFLEDTGADSPAEATGYSILKD

KMNEVLLTLTDRERKVLIQRFGLNDGKPKTLEEVGVEFNVTRERIRQIEAKALRKMRHPT

RSKQLKAFLDLLEVE

>tr|D6YS12|D6YS12_WADCW Acyl-[acyl-carrier-protein]--UDP-N-acetylglucosamine O-acyltransferase OS=Waddlia chondrophila (strain ATCC VR-1470 / WSU 86-1044) OX=716544 GN=lpxA PE=3 SV=1

MSESKIHPMAYVESGAKIGKNVTIEPFAVVKGNVTLEDHVVIKSHAYIDGYTTIGEGTVI

YPNASIGTKSQDLKYRGERTFVNIGKHCEIREFVTINSSSGEDTYVKVGDNCFIMAYCHI

AHNSVIGNHVVMSNNATLAGHVTIEDFAIIGGLTPIHQYVRVGTYAMVGGMSRVPHDVPP

YTIGAGIPFKFGGLNLIGLKRHGFSLETRKALSQAFKLTFRSKLHLDEAIARIESELPLL

PEIENWISFCKRTKRGIIGLQGVINAEEEEIPFTDEEEEEVSVKETQAVTV

>tr|D6YSY9|D6YSY9_WADCW DNA helicase OS=Waddlia chondrophila (strain ATCC VR-1470 / WSU 86-1044) OX=716544 GN=uvrD PE=3 SV=1

MMYAAGLNEPQQDAVNTFNGPLLVLAGAGSGKTRVVTYRIAALIESGVPASQILGLTFTN

KAAGEMQERIRKIANSHVLISTFHSLGARILRESIHELGYRSDFTIYDEDDVLKLLKACL

EELKIRDKKMEPKVFRSLISKAKNQLLGPDQVQSSQSKDPADMALPMVYRKYQERLKEYN

ALDFDDLLFLVVKLFRECPEVLKGYQDRWSHLLIDEYQDTNHAQYEIVCKLVERTKNLCV

VGDPDQSIYSWRGANIQNILNFENDFAGAKVVRLEQNYRSRANILDAANHLIGFNQQRFE

KNLWSALGPGEKIKHYTGDSDRNESQFVADTIRYFHEEKGIPYQEMVIFYRTNFQSRAFE

DRLLSSRIPYVIVGGISFYQRKEIKDVLAFLRMTYSDSDFISFQRTINLPRRGLGNATIE

KIRLGATQEGMTILEYCQALVDGGVSLRMTTKQKEGLEGYLSMIRKLKRIYAECSLKELV

IAAVEETGYLNHLSEDPETVDDRKANLDELRTKAMEWEQERDSAELADFLEELSLKSSLD

EADAGLVDRVNLMTIHNGKGLEYTLVFLVGMEEDLFPHANSRGSQEALEEERRLCYVGMT

RAKEYLYITDARFRYLWGQARTQRPSRFLKEIPIEHLQRVRDGITIGGSSMMQTSEMETE

FESGDAVFHQEFGVGVVQQVYDSSVGMIYKVLFSSDCQVKDLAARYSHLTRL

>tr|D6YVR7|D6YVR7_WADCW Aspartokinase OS=Waddlia chondrophila (strain ATCC VR-1470 / WSU 86-1044) OX=716544 GN=lysC PE=3 SV=1

MKFGGASVATPKHFSRIADIILQRKQEYERIVTVVSAMGNTTDELISLAKQVHPNPPRRE

YDMLVTVGERISISLLAMALSLKEQEAVSFTGSQSGIITTDDHTEARIIDVRPYRLIPCL

QEEKVVIVAGFQGVSMNKEITTLGRGGSDTTAVALGIALEAECVEFYKDVAGIFERDPKK

FPESTCYSCLTYAQSRKIIDSGAKVLSIRCLDLAEKNGIPLWVRSFDDDSDDQGTRIFDR

NLVLPVQPIYEYTCQNMRETGRISNCV

>tr|D6YS14|D6YS14_WADCW UDP-3-O-acyl-N-acetylglucosamine deacetylase OS=Waddlia chondrophila (strain ATCC VR-1470 / WSU 86-1044) OX=716544 GN=lpxC PE=3 SV=1

MAVVAEGIATSTRTQNTIGEAVSFSGIGIHTGKVVSIRFVPSECGTGVVFKRIDLPGHPE

IPATLEYVCDTSRSTTIGISDVRIHTVEHVLAAVRACQIDNLIIEISSIEPPVGNGSSDV

FIEMIKKVGVVGQEGSAPIVYLDQPIYWSEEEVHVVAIPYDGYKISYTLNYPDSAILKCQ

YQSFEITEEIFIKELSQCRTFSEYREVEMLMKRGLIKGGSLDNAVIIKDEAVFSKGGLYF

QDEMARHKILDMIGDLSLVGFDFRAHVIAIRSGHQANVAFAQQLYQKLTRETL

>tr|D6YSK4|D6YSK4_WADCW t(6)A37 threonylcarbamoyladenosine biosynthesis protein TsaE OS=Waddlia chondrophila (strain ATCC VR-1470 / WSU 86-1044) OX=716544 GN=wcw_1705 PE=3 SV=1

MVSLNSEVVICRSPEETEELGFQLGKQLPNRSVVCFFGDLGAGKTTFIKGLARGAGGIDP

DEVNSPTFVYLNIYEGQLPIYHFDLYRLKDVQEFIRMGLDEYLNGEGICCLEWSERIEGH

LPPKTIRVEICHVDQSKREVRILQ

>tr|D6YWN5|D6YWN5_WADCW 3-oxoacyl-[acyl-carrier-protein] synthase 3 OS=Waddlia chondrophila (strain ATCC VR-1470 / WSU 86-1044) OX=716544 GN=fabH PE=3 SV=1

MLKARITGLGSYQPEKILSNQDLEKIVDTSDEWISTRTGMKERRIAASDEASSDMGIKAA

AKALLAAKLPADQIDLILVATSTPDHLMPSTAAIVQSAIGAVNAAAFDLLAACTGFLYGM

SAAKAYIESGMYRNVLLIASEKISSFIDYQDRTTCVLFGDGASAAVISCEGSGYAIENVC

LGTDGSLADLIMIPGGGSRLPLSEKALQERQQYFKMSGKEVFKHAVRKMSAAAMESLEKA

GLKEEQISWLVPHQANARIIDALAKKFQMPLERVGKTVHKYGNTSAPSVAMTLDELIVEN

EIRDGEHLLLVAFGAGLTWGASVITKMGGDR

>tr|D6YWD1|D6YWD1_WADCW Riboflavin biosynthesis protein RibBA OS=Waddlia chondrophila (strain ATCC VR-1470 / WSU 86-1044) OX=716544 GN=ribA PE=3 SV=1

MNSRLDKAFEVYRKGGFVIVQDDFDRENEGDLIIAAEKVTPEAIAFMVRYTSGIICVSLK

EERLKQLCLPQMVPENTEIYQTAFTIPVDYCQGTTTGVSAEDRAKTIRALVNPDSCPEDF

RRPGHVFPLRYREGGVLKRAGHTEAAVDLAILTGLQPAGVISEIVNQDGSMAKDDQLQFF

AKEHDLPLISISDIVRYRREKEKLVECISEARMPTRFGSFTAFVYKSLLDNIEHLALVKG

DVKGKDSVLVRVHSECLTGDIFGSKRCDCGNQLDLSLQQIDAEGLGVLIYLRGHEGRGIG

LGHKLRAYALQDQGRDTVEANLELGFPIDSREYGVGAQMLSDLGLTTIRLMTNNPSKYGG

LEGYDLKIVERVPLQAVCNDENRRYLTTKKEKLGHLLEV
